# Exploration for Asian longhorned beetle parasitoids in Korea using an improved sentinel log trap[Fn FN1]

**DOI:** 10.1051/parasite/2023062

**Published:** 2023-12-12

**Authors:** Seunghyun Lee, Duk-Young Park, Xingeng Wang, Jian J. Duan, Juli R. Gould, Il-Kwon Kim, Seunghwan Lee

**Affiliations:** 1 Key Laboratory of Zoological Systematics and Evolution, Institute of Zoology, Chinese Academy of Sciences Beijing 100101 China; 2 Insect Biosystematics Laboratory, Department of Agricultural Biotechnology, Seoul National University Seoul 08826 Republic of Korea; 3 Research Institute of Agricultural and Life Sciences, Seoul National University Seoul 08826 Republic of Korea; 4 USDA Agricultural Research Service, Beneficial Insects Introduction Research Unit Newark DE 19713 USA; 5 USDA Animal and Plant Health Inspection Service, Otis ANGB Laboratory MA 02542 USA; 6 Division of Forest Biodiversity, National Arboretum Pocheon 11186 Republic of Korea

**Keywords:** Biological control, Molecular identification, Parasitism rate, Trapping method

## Abstract

The Asian longhorned beetle (ALB), *Anoplophora glabripennis* (Motschulsky) (Coleoptera: Cerambycidae), is a destructive invasive woodboring insect pest, and efforts are being made to find parasitoids for ALB biological control. Through a four-year survey in Korea using a sentinel log trap associated with host chemical cues potentially important for host finding by parasitoids, two parasitoid species were discovered attacking ALB. One species is *Spathius ibarakius* Belokobylskij & Maetô, which is known to also parasitize citrus longhorned beetle, *Anoplophora chinensis* (Forster). The other parasitoid species, whose offspring were dead before imago, could not be morphologically identified at the adult stage. We attempted molecular and morphological identification of the larvae/pupae of the unidentified parasitoid; however, only superfamily-level identification was possible. The parasitism rate recovered in the logs was 0.3% by the unidentified parasitoid in Gapyeong-gun in 2019, while it reached 29.2% by *S. ibarakius* in Busan city in 2022. Future efforts for exploring ALB natural enemies in the pest’s native range may focus on parasitoids with high parasitism rates.

## Abstract (fr)

Le longicorne asiatique (LA), *Anoplophora glabripennis* (Motschulsky) (Coleoptera : Cerambycidae), est un insecte ravageur envahissant et destructeur du bois, et des efforts sont déployés pour trouver des parasitoïdes pour la lutte biologique contre lui. Au cours d’une étude de quatre ans en Corée utilisant un piège à bûche sentinelle associé à des signaux chimiques de l’hôte potentiellement importants pour la détection de l’hôte par les parasitoïdes, deux espèces de parasitoïdes ont été découvertes attaquant le longicorne. Une espèce est *Spathius ibarakius* Belokobylskij & Maetô, qui est connue pour parasiter également le longicorne des agrumes, *Anoplophora chinensis* (Forster). Les autres espèces de parasitoïdes, dont les descendants sont morts avant l’imago, n’ont pu être identifiées morphologiquement au stade adulte. Nous avons tenté une identification moléculaire et morphologique des larves/pupes du parasitoïde non identifié, mais seule une identification au niveau de la superfamille a été possible. Le taux de parasitisme observé dans les bûches était de 0,3 % par le parasitoïde non identifié à Gapyeong-gun en 2019, tandis qu’il atteignait 29,2 % par *S. ibarakius* dans la ville de Busan en 2022. Les efforts futurs pour explorer les ennemis naturels du capricorne dans l’aire de répartition naturelle du ravageur pourraient se concentrer sur les parasitoïdes à taux de parasitisme élevés.

## Introduction

The Asian longhorned beetle (ALB), *Anoplophora glabripennis* (Motschulsky), is native to China and Korea, where outbreaks have occurred. This serious pest has expanded its distribution from its native range, invading Canada, Europe, the United States, and other countries, including Japan. ALB-invaded countries have allocated large budgets to ALB control and eradication [22] and the estimated economic loss caused by ALB is tremendous [41]. Despite concerted efforts for eradication (*e.g.*, pesticide injection, infested tree removal), small and invasive ALB populations still remain in several US States and a few European countries [5].

Biological control could be a promising alternative to chemical pesticides for combating pests. Chemical pesticide use often enhances pest resistance, rendering them ineffective in pest control [51]. As an alternative, extensive research is being conducted worldwide to discover effective natural enemies such as entomopathogenic fungi, parasitic nematodes, pathogenic bacteria, and insect parasites for important insect pest biological control, including ALB [4]. Several studies have surveyed or investigated natural hymenopteran parasitoids in both ALB native and invaded regions. In North America and Europe, which have experienced ALB invasions, some potential larval parasitoids for ALB control have been identified [4, 13, 23, 25, 37, 50, 56]. Notably, some braconid species (*Ontsira mellipes* Ashmead, *Rhoptrocentrus piceus* Marshall*, Spathius laflammei* Provancher, *Heterospilus* sp. and *Atanycolus* sp.) have shown the ability to readily attack ALB larvae in laboratory tests [13]. In China, surveys have discovered several larval ALB parasitoids [18, 36, 42, 43, 47, 59]. Among them, *Spathius anoplophorae* Yang (Braconidae) and *Oxysychus glabripennis* (Pteromalidae) were the two most dominant parasitoids that were collected in the different geographical regions in sentinel log traps and exhibited relatively high parasitism rates on ALB larvae [36, 59]. Several generalist parasitoids, such as *Dastarcus helophoroides* (Fairmaire) and *Sclerodermus guani* Xiao & Wu, have shown effectiveness on various woodboring beetles including ALB in China. However, their broad host ranges make them unsuitable for classical biological control in non-native regions like Europe and North America due to their potential risks to attack native woodborers [21, 45]. Thus, specialized natural enemy identification, particularly those targeting ALB eggs or early-instar larvae, is essential for effective biocontrol program development in both native and invaded ranges [36].

Past surveys for ALB parasitoids involved ALB egg or larval collection directly from trees by felling and dissecting infested trees under natural conditions [17, 61] or launching sentinel logs already pre-infested by ALB eggs and/or larvae under laboratory conditions before log deployment [29, 36]. However, sentinel logs were usually deployed at the survey site after laboratory-induced oviposition, ignoring various fresh chemical cues produced during oviposition (*e.g.*, host volatile compounds, pheromones [15]). Moreover, previous field surveys in China were conducted primarily in urban areas other than natural forests, as ALB populations are extremely low in natural forests [36]. In low-density host populations, any specialist parasitoids are unlikely to maintain their populations in natural forests or are difficult to find. To increase the probability of finding parasitoids, it is efficient to target areas with high ALB populations using sentinel log traps that consider chemical cues that could be potentially important for host search by parasitoids.

This study aimed to discover native ALB parasitoids in Korea, including egg parasitoids, using an improved sentinel log trap deployed on ALB host trees in the field. Surveys were conducted over four consecutive years in natural forests where native ALB populations dwell and one year in urban areas where invasive populations are present in Korea. During our survey, two parasitoid species emerged from ALB eggs or larvae; however, one larval species died before imago. Therefore, we adopted molecular identification using public and *de novo* putative parasitoid adult data.

## Materials and methods

### An improved sentinel log trap

All previous surveys used sentinel logs infested with ALB eggs and/or larvae in the laboratory prior to field exposure and discovered many ALB larval parasitoids. However, these surveys failed to find true ALB egg parasitoids [29, 36]. It is possible that there is an overall lack of parasitoids attacking ALB eggs or egg parasitoids are attracted to volatile compounds released by plants in response to herbivore damage, as well as to volatiles from undamaged plants and different types of pheromones from host adults [15]. In addition, physical (*e.g.*, vibration created during female ALB oviposition pit preparation or mating [39]), chemical cues (*e.g.*, plant volatiles induced by egg deposition or volatiles on the egg itself), or contact semiochemicals from host adult footprints may play a key role in attracting ALB egg parasitoids.

**Figure 1:**
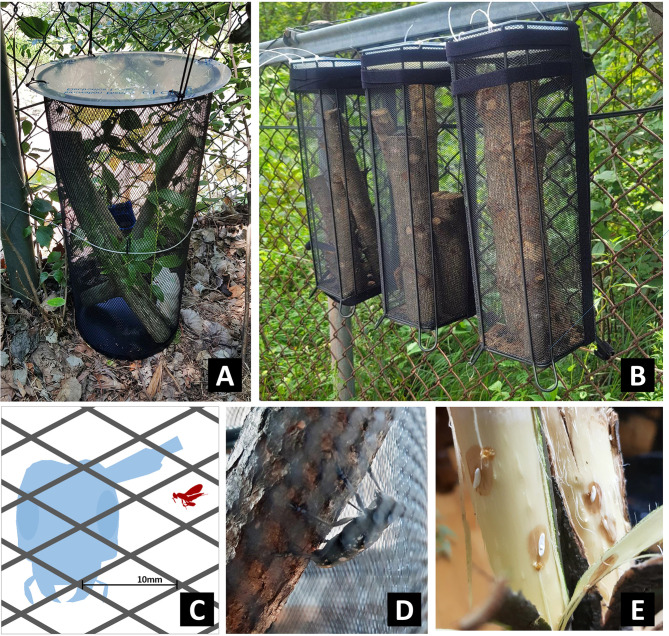
An improved sentinel log trap. A. Cylinder type, B. Box type, C. Size comparison of mesh, ALB and parasitoid, D. ALB in trap, E. ALB eggs on dissected logs.

Therefore, an improved sentinel log trap was designed to allow female ALB to oviposit on sentinel logs in the field. Two types of metal mesh buckets (box type L140 × W140 × H350 mm; cylinder type Ø240 × H390 mm) were used with a mesh size < 10 mm through which parasitoids could pass, but ALB adults could not (Figs. 1A–1D). Sentinel logs and ALB adults were placed into the trap, the top of the trap was tightly sealed to prevent adults from escaping, and they were subsequently placed in the ALB natural habitat.

### Field survey

Currently, Korean ALBs are divided into three geographical subgroups, of which two are invasive and only one is native [35]. Southern and western invasive subgroups show massive population levels [29, 35], thus, adults were collected from those invaded areas, focusing on the collection of as many females as possible. Adults were collected by hands and sweep nets in June and July each year in the urban areas where invasive populations thrive sporadically.

The traps were launched and collected during the summer months from 2019 to 2022. For sentinel logs, *Acer mono* Maxim, *Acer saccharinum* Linnaeus, *Aesculus turbinata* Blume and *Salix koreensis* Andersson were used, each approximately 30 cm in length and 5–12 cm in diameter. Three to five sentinel logs were placed into the trap depending on the log size with twigs for providing sustenance to adults, and 5–10 females and 2–5 males were subsequently placed in each trap depending on the sentinel log volume. In total, 12 *A. mono*, 23 *A. saccharinum*, 239 *A. turbinate*, and 114 *S. koreensis* logs were launched over four years (Table S1). Most traps were launched at a height of 1–2 m near the ALB oviposition site, and a few were mounted at a height > 5 m where oviposition pits were clustered at a high density in nature. In total, 12 *A. mono*, 23 *A. saccharinum*, 239 *A. turbinate*, and 114 *S. koreensis* logs were launched in Gapyeong-gun, Gyeonggi-do, where the largest population level within their native range was observed. However, only 16 *S. koreensis* logs were launched in Busan city, where there were highly ALB invasive populations to confirm the existence of ALB parasitoids in this invaded area. Sentinel logs were retrieved after they had been exposed in the field for 2–4 weeks for mainly finding the ALB egg parasitoids. All traps were examined for oviposition pits in sentinel logs before collection.

Most of the sentinel logs (82.4%, 333/404) were dissected in the laboratory to check for parasitism (Fig. 1E), whereas a few (17.6%, 71/404) were placed in a rearing cage without dissection to allow parasitoids to emerge naturally from the logs. Following outer bark removal with pruning shears, the number of ALB eggs and larvae was counted (Table S1), and all putative parasitoids at various stages (larvae, cocoons, and adults), if present, were collected and reared.

### Parasitoid identification

#### Morphological identification

Morphological identification for braconid species was conducted by referring to [2, 54]. One parasitoid species was found with only two individuals at the larval stage during the 2019 survey, and both died before imago during rearing. Therefore, identification of the larvae and pupae of this species was carried out using external morphology and molecular analysis by referring to [1, 6–10, 12, 14, 19, 20, 29, 38].

#### Molecular identification

The species with its larvae/pupae died before imago was identified as belonging to the superfamily Chalcidoidea based on morphological characteristics. Tree-based molecular identification was adopted for species-level identification [48, 49, 60] in conjunction with a BLAST search, which has been widely used in previous studies [53]. To enhance the molecular identification success rate, we made efforts to obtain numerous DNA sequences from Chalcidoidea specimens collected in the same or closely related regions where the unidentified parasitoid was discovered. Most Chalcidoidea specimens were sorted from unsorted Malaise bulk samples deposited in Korea National Arboretum entomology collections (KNAE). Among the Malaise bulk sample, putative parasitoids with sizes similar to the dead parasitoid pupae collected in 2019 were sorted. Total genomic DNA was extracted from the entire body of insects using the DNeasy Blood and Tissue Kit (QIAGEN, Hilden, Germany). After DNA extraction, all voucher specimens, except for the unknown ALB parasitoid larvae and pupae, were deposited in KNAE. All genomic DNA samples were preserved in the laboratory freezer of Insect Biosystematics at Seoul National University. The mitochondrial cytochrome oxidase subunit I (COI) gene was targeted using four primer sets for the unknown ALB parasitoid larvae and pupae (Table S2). For the other parasitoid adults, only one primer set (SLEPF/LEPR) was used. Both strands were assembled with Seqman Pro v.7.1.0 (DNASTAR, Inc., Madison, WI, USA) and examined and manually adjusted using MEGA X [31] with the amino acid translation option.

Our final sequence alignment consisted of 213 *de novo* (Table S3) and 8,210 public COI Chalcidoidea sequences (Table S4), and one outgroup sequence. First, all Chalcidoidea COI sequences were retrieved from GenBank (https://www.ncbi.nlm.nih.gov/genbank/) and *de novo* sequences were added. For genetic analysis convenience, perfectly redundant sequences were deleted using Jalview 2.1.1 [58] and short sequences (< 600 bp) were automatically removed using Seqkit v.0.12.0 [46]. All sequences were aligned using MAFFT ver. 7 online [27] with an alignment algorithm automated selection. Multiple sequence alignment was manually assessed in MEGA X [31]. New sequences were deposited in GenBank with accession numbers OQ134171–OQ134383.

Phylogenetic trees were constructed using the maximum likelihood (ML) approach. The best-fit substitution model (TPM2 + F + G4) was determined using ModelFinder [26] under the Bayesian information criterion. ML analysis was performed using the IQ-TREE web server [52] and the ultrafast bootstrap nodal support value was evaluated from 1,000 replicates.

## Results

### Discovery of additional ALB parasitoids

**Figure 2:**
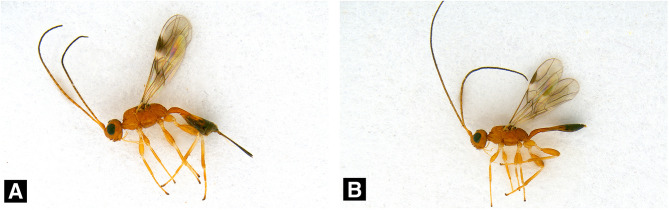
Habitus of *Spathius ibarakius* Belokobylskij & Maetô. A. Female, B. Male.

During the survey conducted from 2019–2022 using an improved sentinel log trap, two additional ALB parasitoid species were discovered. One species was identified as *Spathius ibarakius* Belokobylskij & Maetô (Figs. 2A–2B), which is known to parasitize CLB larvae. However, another species died before imago, making it impossible to identify them at the adult stage. Therefore, identification was conducted based on larval and pupal morphological and molecular identifications. Unfortunately, species-level identification failed in both analyses. However, its affiliation was identified as belonging to the superfamily Chalcidoidea.

### Morphology of the unidentified parasitoid terminal-instar larva and pupa

**Figure 3:**
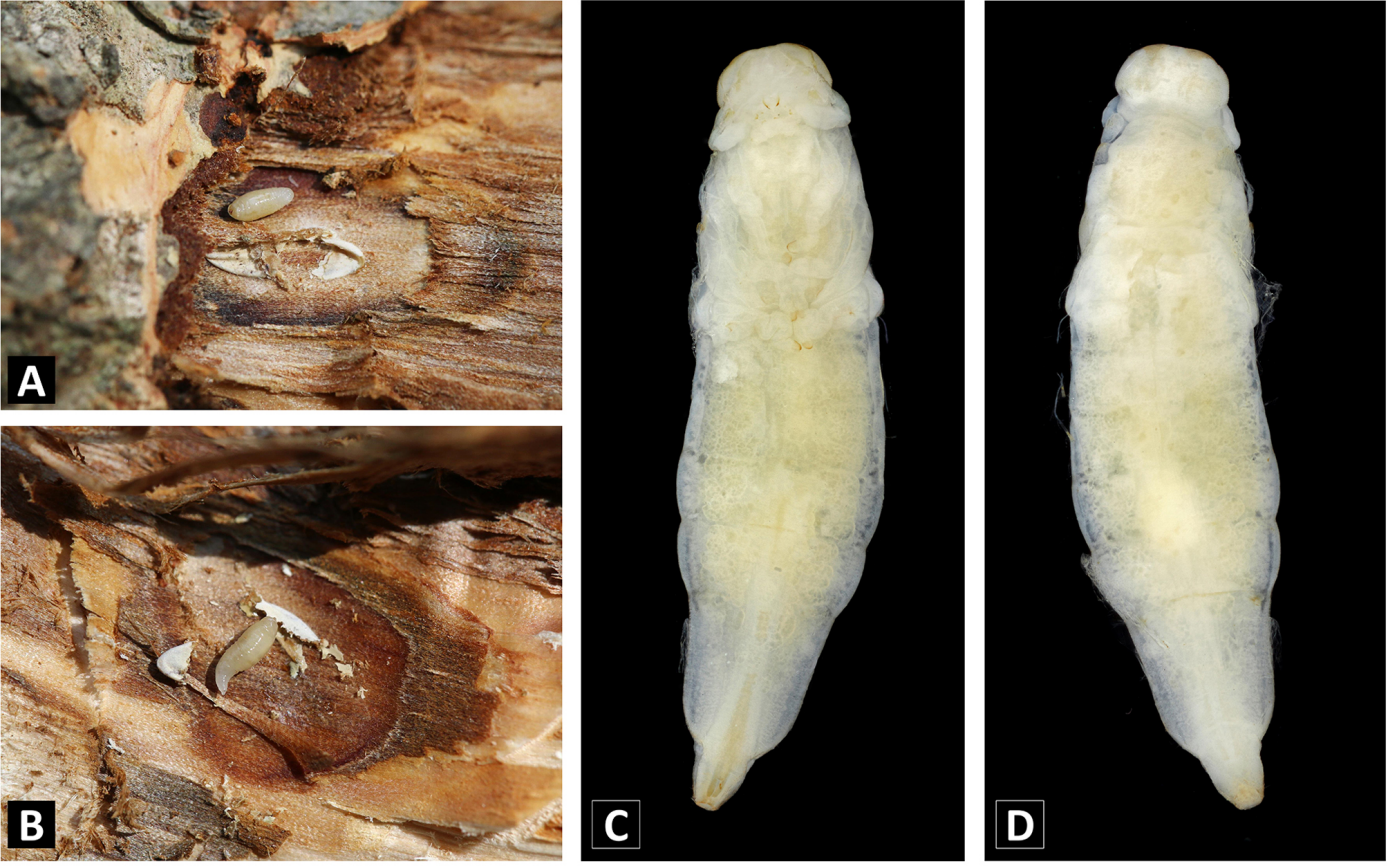
Laval and pupal morphology of the unidentified parasitoid. A. Larva 1, B. Larva 2, C. Ventral view of pupa, D. Dorsal view of pupa.

Larva (Figs. 3A–3B). Hymenopteriform. Body 2.40× as long as width; brownish-white; with 13 segments; glabrous; integument smooth and fusiform; anterodorsal protuberances absent.

Pupa (Figs. 3C–3D). Body 3.90× as long as width. Head 1.55× as wide as length in dorsal view; distinctly separated from mesosoma. Mesosoma poorly developed but posterior region combined with metasoma; propodeum indistinct. Antenna, eyes, legs and wings visible but less developed. Metasoma with 7 gastral tergites and 5 gastral sternites; 6th gastral tergite and 5th gastral sternite longer than others; 4th gastral tergite and 3rd gastral sternite broader than others. Ovipositor sheaths developed and protruded beyond the gaster in dorsal view; start from anterior margin of 3rd gastral tergite. (Measurement: body length, 7.48 mm; body width, 1.92 mm; head length, 0.72 mm; head width, 1.12 mm).

### Molecular identification

**Figure 4:**
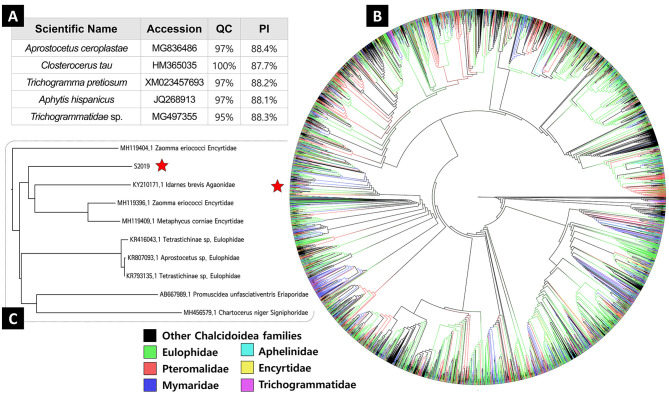
Results of phylogenetic analysis. A. List of Blast search, B. ML phylogenetic tree, C. Partial tree of closest affinity taxon with the unidentified parasitoid.

In the BLAST search, *Aprostocetus ceroplastae* (Girault) (Eulophidae) [MG836486] showed the highest percentage of identical nucleotides (88.4%) compared with the unidentified parasitoid [OQ134171], followed by species of the families *Trichogrammatidae* (*Trichogrammatidae* sp. [MG497355], *Trichogramma pretiosum* Riley [XM023457693]) and Aphelinidae (*Aphytis hispanicus* Mercet [JQ268913]) (Fig. 4A).

The phylogenetic analysis, not surprisingly because of their higher saturation [11], recovered serious polyphyly of all the major families of Chalcidoidea, which was divided into numerous clades (Fig. 4B). This serious polyphyly and low branch support results in true phylogenetic relationship uncertainty between most of the genera and families. The unidentified parasitoid showed the closest affinity to partial (Agaonidae + partial Encyrtidae), followed by partial (Eulophidae + Eriaporidae + Signiphoridae) (Fig. 4C).

Overall, both BLAST searches and molecular phylogenetic identification failed to identify the unknown parasitoids at the species level.

### Parasitism rate

The parasitism rate of the unidentified parasitoid discovered in Gapyeong-gun in 2019 was found to be 0.3% (2/592). Considering *S. ibarakius*, it was observed in both Gapyeong-gun and Busan city. However, the exact number of *S. ibarakius* parasitized individuals in Gapyeong-gun was not counted. The *S. ibarakius* parasitism rate in Busan city is shown as 29.2% (21/72) (Table 1).

**Table 1:** Number of ALB and parasitoids collected from 2019 to 2022.

Year		2019	2020	2021	2022	
Location		Gapyeong-gun	Gapyeong-gun	Gapyeong-gun	Gapyeong-gun	Busan city
Number of specimens	ALB eggs and larvae	590	380	1,691 + n/c	311	51
	Unidentified parasitoid	2	0	0	0	0
	*S. ibarakius*	n/c	n/c	n/c	n/c	21
Parasitism rate (%)	Unidentified parasitoid	0.3	0	0	0	0
	*S. ibarakius*	n/c	n/c	n/c	n/c	29.2

*n/c: no count.

## Discussion

Through this study, we discovered two additional parasitoid species that attack ALB in Korea. One species was identified as *S. ibarakius*, which is known to parasitize CLB larvae [28]. However, the other species died before imago during rearing, preventing us from confirming the identification based on adult morphology. To identify the unidentified parasitoid, we conducted identification using the morphology and molecular analysis of the larvae and pupae.

Some morphological characteristics of the larva and pupa allowed the candidate families to be narrowed down. First, the unidentified parasitoid larva has a head + 13 body segments; however, Platygastroidea have a head + 10–11 body segments [7]. The longest gastral tergite in this species is the 6th; thus, Platygastroidea, which has the same length for all gastral tergites or at most one of the 1st–3rd gastral tergites longer than the others [6, 38], can be excluded. Decisively, the pupa has distinctly visible ovipositor sheaths that generally appear in some Chalcidoidea families [8–10, 30]. In contrast, in Platygastroidea, ovipositor sheaths are inside the metasoma [1], and in Ichneumonoidea, visible ovipositor sheaths start at the basal margin of the metasoma [12, 14]. The aforementioned morphological characteristics combination suggests that the unidentified parasitoid is most likely a Chalcidoidea. Among Chalcidoidea, Eulophidae and Pteromalidae are the candidate families because the unidentified parasitoid pupal setae and anterodorsal protuberances are indistinct in body segments [19, 20]. Moreover, because this specimen has ovipositor sheaths ventral to the metasoma, it appears to be a female [32].

However, all families of Chalcidoidea were super-polyphyletic in both the ML and NJ phylogenetic trees. The polyphyly was expected because COI is generally a poor marker for a deep-time phylogenetic relationship, which is a fast-evolving marker with higher saturation [11]. A BLAST nucleotide search suggested that the parasitoid pupal sequence is closest to *A. ceroplastae*; however, the identical base pair percentage was too low (88.4%) to be of the same species (recommended threshold: 2–3%; [24, 40]). Hence, the second to fifth closest species in the BLAST results are all from different genera, and several *Aprostocetus* species in our phylogenetic tree are also highly polyphyletic. This severe polyphyly results in molecular identification failure at every taxonomic rank, even at the family level.

At the time of its initial discovery, the unidentified parasitoid was believed to be an ALB egg parasitoid. This assumption was based on the presence of ALB eggshell partial remains in the vicinity of the unidentified parasitoid larvae and the absence of feeding traces from ALB larvae. Similarly, [62, 63] reported *Callimomoides monochaphagae* Yang (Hymenoptera: Pteromalidae) emerging from *Monochamus alternatus* Hope eggs. According to their biological observations, the *C. monochaphagae* immature larvae, which had consumed all the host egg contents, emerged by piercing through the eggshell before imago. However, typical egg parasitoids are known to develop their immature stages within host eggs [3]. Therefore, it is challenging to consider the species we discovered as a true egg parasitoid, as it was found outside the host eggshell at the late larval stage. Based on the feeding traces on the ALB eggshell, two possibilities can be inferred: i) The unidentified parasitoid may be an ectoparasitic egg parasitoid. It is possible that the ALB eggshell was gnawed upon by larvae emerging from the parasitoid eggs, which were laid outside the ALB eggs. Certain species, such as *Aprostocetus gala* (Walker) within the family Eulophidae, are known to feed externally on citrus weevil egg masses (*Diaprepes abbreviatus* (Linnaeus)) [55]. ii) The unidentified parasitoid may be an extremely early larval parasitoid. This is suggested by the fact that the parasitoid larvae were found outside the host eggs, and no feeding activity by ALB larvae on the tree was observed. Considering this, it is likely that the parasitoid laid its eggs in/on ALB larva before the ALB larva emerged from the eggshell or shortly after the ALB larva emerged from the eggshell but had not started feeding externally. Conversely, it showed that *S. ibarakius* parasitizes young ALB larvae in a similar manner to its CLB larval parasitization [28], as *S. ibarakius* cocoons were discovered together with ALB larval shells.

We used an improved trap that took into account chemical cues in an attempt to find ALB parasitoids. In previous studies, sentinel logs were installed without considering chemical cues, inducing ALB adult oviposition in a laboratory setting, which led to the discovery of mostly larval parasitoids and two suspected egg parasitoids (*Anastatus* sp. and *Xorides* sp.) [36]. However, all *Xorides* (Ichneumonidae) known species are coleopteran larval ectoparasitoids [16, 44, 57]. It is unclear whether *Xorides* sp. is an egg parasitoid. Furthermore, *Anastatus* members are known egg parasitoids in several orders, particularly Lepidoptera and Hemiptera. However, the *Anastatus* sp. reported in early surveys has never been found again in recent surveys at the same site [59]. Conversely, several *C. monochaphagae* specimens, thought to parasitize *M. alternatus* eggs, were also found from naturally laid *M. alternatus* eggs in the field in China [62, 63]. This suggests that there is a correlation between the chemical cues during oviposition and parasitoid attraction, emphasizing the importance of chemical factor consideration in the search for parasitoids. However, despite four years of intensive efforts, only two parasitoid species were collected. While *S. ibarakius* was found every year, the unidentified parasitoid was only found once, with two individual larvae. This suggests that the ALB native population density in Korea may be low, or the density and diversity of parasitoid species specific to Korea might be extremely low.

Utilizing the unidentified parasitoid discovered in this study for ALB control may appear to be the most effective approach as it may prevent ALB larvae from causing damage to trees before their feeding stage. However, due to the low density, it seems challenging to use this species as an efficient control agent. Instead, [36] suggested using early larval parasitoids as biological control agents (BCAs). Among the larval parasitoids, *Oxysychus* sp. (5% in Beijing and Shanghai) had the highest parasitism rate compared to other parasitoids. However, Braconidae have high diversity within the ALB parasitoids. Among the known ALB parasitoids found in Korea and China, eight species belong to Braconidae (*Atanycolus* sp., *Bracon planitibiae* Yang, *Heterospilus* sp., *Leluthia honshuensis* Belokobylskij & Mateo, *S. anoplophorae* Yang, *S. laflammei* Provancher, *Zombrus sjoestedti* (Fabringer)) [18, 28, 36]. Furthermore, the *S. ibarakius* parasitism rate, discovered in this study, was remarkably high at 29.2% compared to the unidentified parasitoid (0.3%) (Table 1). Recent surveys in China also found mainly the two larval parasitoids *S. anoplophorae* Yang and *O. glabripennisi* Yang, that were most abundant and collected consistently in different geographical regions and years from sentinel logs, indicating their potential as BCAs for ALB [59]. In the United States, some North American native braconid larval parasitoids collected from other cerambycids were found to be capable of attacking ALB in laboratory tests, including *Ontsira mellipes* Ashmead, *Rhoptrocentrus piceus* Marshall, and *S. laflammei* Provancher [13, 18, 56]. Similarly, several native European woodborer larval parasitoids can parasitize ALB larvae [4]. These resident parasitoids could potentially attack ALB in the invaded regions.

In the current study, we conducted a survey to identify additional natural ALB parasitoids in Korea. Unlike previous surveys, our study considered various environmental factors such as chemical cues (*e.g.*, host volatile compounds, pheromones), which are necessary for attracting natural parasitoids in the field. Consequently, we developed a sentinel log trap designed to provide natural host chemical cues to effectively detect and monitor the parasitoids associated with other emerging cerambycid pests [33, 34]. Despite our efforts, we were only able to identify two new ALB parasitoids, and also failed to find true ALB egg parasitoids. One of them is *S. ibarakius*, known to parasitize CLB larvae. The other parasitoid remains unidentified but appears to be closely related to an ALB egg external predator or an extremely early larval parasitoid. Although the unidentified parasitoid was not abundant, our results suggest the need for further exploration of host-specific larval parasitoids for effective ALB control strategies.

## Supplementary materials

The Supplementary materials of this article are available at https://www.parasite-journal.org/10.1051/parasite/2023062/olm. *Table S1*. Dates and number of materials used in this study. *Table S2*. List of primers used in this study. *Table S3*. Information of specimens used for sequencing. *Table S4*. Species information and GenBank accession number. Abbreviations used in Supplementary materials AM: *Acer mono* AS: *Acer saccharinum* AT: *Aesculus turbinata* SK: *Salix koreensis* ALB: Asian Longhorn Beetle
