# Supplementary material for: Exploration for Asian longhorned beetle parasitoids in Korea using an improved sentinel log trap
Source: Parasite. 2023 Dec 12;30:57. doi: 10.1051/parasite/2023062 (PMC10714676; doi:10.1051/parasite/2023062)
Supplement: Supplementary file 1 — Table S1. Dates and number of materials used in this study. Table S2. List of primers used in this study. Table S3. Information of specimens used for sequencing. Table S4. Species information and GenBank accession number. [file parasite-30-57-s1.zip › Supplementary table S4.pdf]

Table S4. Species information and GenBank accession number

Order	Superfamily	Family	Genus	Species	Accession number
Hymenoptera	Apidoidea	Apidae	Apis	mellifera	MF100924.1
Hymenoptera	Chalcidoidea	Agaonidae	Agaon	sp.	GQ367956.1
Hymenoptera	Chalcidoidea	Agaonidae	Agaonidae	sp.	MK543425.1
Hymenoptera	Chalcidoidea	Agaonidae	Agaonidae	sp.	MK559431.1
Hymenoptera	Chalcidoidea	Agaonidae	Agaonidae	sp.	KY831422.1
Hymenoptera	Chalcidoidea	Agaonidae	Agaonidae	sp.	KY834713.1
Hymenoptera	Chalcidoidea	Agaonidae	Alfonsiella	longiscapa	GQ367962.1
Hymenoptera	Chalcidoidea	Agaonidae	Alfonsiella	michaloudi	GQ367936.1
Hymenoptera	Chalcidoidea	Agaonidae	Alfonsiella	pipithiensis	GQ367963.1
Hymenoptera	Chalcidoidea	Agaonidae	Apocryptophagus	agraensis	HM770659.1
Hymenoptera	Chalcidoidea	Agaonidae	Apocryptophagus	gigas	HM770636.1
Hymenoptera	Chalcidoidea	Agaonidae	Apocryptophagus	sp.	HM770610.1
Hymenoptera	Chalcidoidea	Agaonidae	Apocryptophagus	sp.	HM770616.1
Hymenoptera	Chalcidoidea	Agaonidae	Apocryptophagus	sp.	HM770629.1
Hymenoptera	Chalcidoidea	Agaonidae	Apocryptophagus	sp.	HM770630.1
Hymenoptera	Chalcidoidea	Agaonidae	Apocryptophagus	sp.	HM770631.1
Hymenoptera	Chalcidoidea	Agaonidae	Apocryptophagus	sp.	HM770632.1
Hymenoptera	Chalcidoidea	Agaonidae	Apocryptophagus	sp.	HM770635.1
Hymenoptera	Chalcidoidea	Agaonidae	Apocryptophagus	sp.	HM770637.1
Hymenoptera	Chalcidoidea	Agaonidae	Apocryptophagus	sp.	HM770638.1
Hymenoptera	Chalcidoidea	Agaonidae	Apocryptophagus	sp.	HM770639.1
Hymenoptera	Chalcidoidea	Agaonidae	Apocryptophagus	sp.	HM770656.1
Hymenoptera	Chalcidoidea	Agaonidae	Apocryptophagus	sp.	HM770657.1
Hymenoptera	Chalcidoidea	Agaonidae	Apocryptophagus	sp.	JN001508.1
Hymenoptera	Chalcidoidea	Agaonidae	Apocryptophagus	sp.	JN001509.1
Hymenoptera	Chalcidoidea	Agaonidae	Apocryptophagus	sp.	JN001513.1
Hymenoptera	Chalcidoidea	Agaonidae	Apocryptophagus	sp.	JN001514.1
Hymenoptera	Chalcidoidea	Agaonidae	Apocryptophagus	sp.	JN001516.1
Hymenoptera	Chalcidoidea	Agaonidae	Apocryptophagus	sp.	JN001517.1
Hymenoptera	Chalcidoidea	Agaonidae	Apocryptophagus	sp.	JN001523.1
Hymenoptera	Chalcidoidea	Agaonidae	Apocryptophagus	sp.	JN001529.1
Hymenoptera	Chalcidoidea	Agaonidae	Apocryptophagus	sp.	JN001556.1
Hymenoptera	Chalcidoidea	Agaonidae	Apocryptophagus	sp.	JN001557.1
Hymenoptera	Chalcidoidea	Agaonidae	Apocryptophagus	sp.	JN001563.1
Hymenoptera	Chalcidoidea	Agaonidae	Apocryptophagus	sp.	JN001565.1
Hymenoptera	Chalcidoidea	Agaonidae	Apocryptophagus	sp.	JN001571.1
Hymenoptera	Chalcidoidea	Agaonidae	Apocryptophagus	sp.	KC421097.1
Hymenoptera	Chalcidoidea	Agaonidae	Apocryptophagus	sp.	KC421098.1
Hymenoptera	Chalcidoidea	Agaonidae	Apocryptophagus	sp.	KC421099.1
Hymenoptera	Chalcidoidea	Agaonidae	Apocryptophagus	sp.	KC421100.1
Hymenoptera	Chalcidoidea	Agaonidae	Apocryptophagus	sp.	KC421101.1
Hymenoptera	Chalcidoidea	Agaonidae	Apocryptophagus	sp.	KC421103.1
Hymenoptera	Chalcidoidea	Agaonidae	Apocryptophagus	sp.	KC421104.1
Hymenoptera	Chalcidoidea	Agaonidae	Apocryptophagus	sp.	KC421107.1
Hymenoptera	Chalcidoidea	Agaonidae	Apocryptophagus	sp.	KC421108.1
Hymenoptera	Chalcidoidea	Agaonidae	Apocryptophagus	sp.	KC421109.1
Hymenoptera	Chalcidoidea	Agaonidae	Apocryptophagus	sp.	KC421110.1
Hymenoptera	Chalcidoidea	Agaonidae	Apocryptophagus	sp.	KC421112.1
Hymenoptera	Chalcidoidea	Agaonidae	Apocryptophagus	sp.	KC421114.1
Hymenoptera	Chalcidoidea	Agaonidae	Apocryptophagus	sp.	KC421115.1
Hymenoptera	Chalcidoidea	Agaonidae	Apocryptophagus	sp.	KC421116.1
Hymenoptera	Chalcidoidea	Agaonidae	Apocryptophagus	sp.	KC421117.1

Hymenoptera	Chalcidoidea	Agaonidae	Apocryptophagus	sp.	KC421118.1
Hymenoptera	Chalcidoidea	Agaonidae	Apocryptophagus	sp.	KC421119.1
Hymenoptera	Chalcidoidea	Agaonidae	Apocryptophagus	sp.	KC421120.1
Hymenoptera	Chalcidoidea	Agaonidae	Apocryptophagus	sp.	KC421121.1
Hymenoptera	Chalcidoidea	Agaonidae	Apocryptophagus	sp.	KC421122.1
Hymenoptera	Chalcidoidea	Agaonidae	Apocryptophagus	sp.	KC421123.1
Hymenoptera	Chalcidoidea	Agaonidae	Apocryptophagus	sp.	KC421124.1
Hymenoptera	Chalcidoidea	Agaonidae	Apocryptophagus	sp.	KC421125.1
Hymenoptera	Chalcidoidea	Agaonidae	Apocryptophagus	sp.	KC421126.1
Hymenoptera	Chalcidoidea	Agaonidae	Apocryptophagus	sp.	KC421127.1
Hymenoptera	Chalcidoidea	Agaonidae	Apocryptophagus	sp.	KC421128.1
Hymenoptera	Chalcidoidea	Agaonidae	Apocryptophagus	sp.	KC421129.1
Hymenoptera	Chalcidoidea	Agaonidae	Apocryptophagus	sp.	KC421130.1
Hymenoptera	Chalcidoidea	Agaonidae	Apocryptophagus	testaceus	HM770663.1
Hymenoptera	Chalcidoidea	Agaonidae	Blastophaga	esquirolianae	GQ367901.1
Hymenoptera	Chalcidoidea	Agaonidae	Blastophaga	psenes	GQ367895.1
Hymenoptera	Chalcidoidea	Agaonidae	Blastophaga	sp.	JN103270.1
Hymenoptera	Chalcidoidea	Agaonidae	Ceratosolen	armipes	JN103254.1
Hymenoptera	Chalcidoidea	Agaonidae	Ceratosolen	blommersi	GQ367916.1
Hymenoptera	Chalcidoidea	Agaonidae	Ceratosolen	blommersi	JN103311.1
Hymenoptera	Chalcidoidea	Agaonidae	Ceratosolen	sp.	JN103256.1
Hymenoptera	Chalcidoidea	Agaonidae	Ceratosolen	emarginatus	GQ367930.1
Hymenoptera	Chalcidoidea	Agaonidae	Ceratosolen	emarginatus	MK543332.1
Hymenoptera	Chalcidoidea	Agaonidae	Ceratosolen	emarginatus	MK543333.1
Hymenoptera	Chalcidoidea	Agaonidae	Ceratosolen	emarginatus	MK543334.1
Hymenoptera	Chalcidoidea	Agaonidae	Ceratosolen	emarginatus	MK543335.1
Hymenoptera	Chalcidoidea	Agaonidae	Ceratosolen	emarginatus	MK543336.1
Hymenoptera	Chalcidoidea	Agaonidae	Ceratosolen	emarginatus	MK543337.1
Hymenoptera	Chalcidoidea	Agaonidae	Ceratosolen	emarginatus	MK543339.1
Hymenoptera	Chalcidoidea	Agaonidae	Ceratosolen	emarginatus	MK543340.1
Hymenoptera	Chalcidoidea	Agaonidae	Ceratosolen	emarginatus	MK543343.1
Hymenoptera	Chalcidoidea	Agaonidae	Ceratosolen	emarginatus	MK543344.1
Hymenoptera	Chalcidoidea	Agaonidae	Ceratosolen	emarginatus	MK543345.1
Hymenoptera	Chalcidoidea	Agaonidae	Ceratosolen	emarginatus	MK559357.1
Hymenoptera	Chalcidoidea	Agaonidae	Ceratosolen	flabellatus	JN103267.1
Hymenoptera	Chalcidoidea	Agaonidae	Ceratosolen	fusciceps	JN103272.1
Hymenoptera	Chalcidoidea	Agaonidae	Ceratosolen	fusciceps	KX242193.1
Hymenoptera	Chalcidoidea	Agaonidae	Ceratosolen	fusciceps	KX242194.1
Hymenoptera	Chalcidoidea	Agaonidae	Ceratosolen	fusciceps	KX242195.1
Hymenoptera	Chalcidoidea	Agaonidae	Ceratosolen	fusciceps	KX242197.1
Hymenoptera	Chalcidoidea	Agaonidae	Ceratosolen	fusciceps	KX242199.1
Hymenoptera	Chalcidoidea	Agaonidae	Ceratosolen	fusciceps	KX242200.1
Hymenoptera	Chalcidoidea	Agaonidae	Ceratosolen	fusciceps	KX242201.1
Hymenoptera	Chalcidoidea	Agaonidae	Ceratosolen	fusciceps	KX242202.1
Hymenoptera	Chalcidoidea	Agaonidae	Ceratosolen	fusciceps	KX242203.1
Hymenoptera	Chalcidoidea	Agaonidae	Ceratosolen	fusciceps	KX242204.1
Hymenoptera	Chalcidoidea	Agaonidae	Ceratosolen	fusciceps	MK543347.1
Hymenoptera	Chalcidoidea	Agaonidae	Ceratosolen	gravelyi	MK543352.1
Hymenoptera	Chalcidoidea	Agaonidae	Ceratosolen	gravelyi	MK543353.1
Hymenoptera	Chalcidoidea	Agaonidae	Ceratosolen	gravelyi	MK543354.1
Hymenoptera	Chalcidoidea	Agaonidae	Ceratosolen	gravelyi	MK543355.1
Hymenoptera	Chalcidoidea	Agaonidae	Ceratosolen	megacephalus	GQ367933.1
Hymenoptera	Chalcidoidea	Agaonidae	Ceratosolen	megacephalus	KX242196.1
Hymenoptera	Chalcidoidea	Agaonidae	Ceratosolen	nanus	JN103297.1

Hymenoptera	Chalcidoidea	Agaonidae	Ceratosolen	sp.	JN103260.1
Hymenoptera	Chalcidoidea	Agaonidae	Ceratosolen	sp.	KU194650.1
Hymenoptera	Chalcidoidea	Agaonidae	Ceratosolen	sp.	KU194651.1
Hymenoptera	Chalcidoidea	Agaonidae	Ceratosolen	sp.	KU194652.1
Hymenoptera	Chalcidoidea	Agaonidae	Ceratosolen	sp.	KU194653.1
Hymenoptera	Chalcidoidea	Agaonidae	Ceratosolen	sp.	KU194654.1
Hymenoptera	Chalcidoidea	Agaonidae	Ceratosolen	sp.	KU194655.1
Hymenoptera	Chalcidoidea	Agaonidae	Ceratosolen	sp.	KU194656.1
Hymenoptera	Chalcidoidea	Agaonidae	Ceratosolen	sp.	KU194657.1
Hymenoptera	Chalcidoidea	Agaonidae	Ceratosolen	sp.	KU194658.1
Hymenoptera	Chalcidoidea	Agaonidae	Ceratosolen	sp.	MK241698.1
Hymenoptera	Chalcidoidea	Agaonidae	Ceratosolen	sp.	MK241700.1
Hymenoptera	Chalcidoidea	Agaonidae	Ceratosolen	sp.	MK241702.1
Hymenoptera	Chalcidoidea	Agaonidae	Ceratosolen	sp.	MK241703.1
Hymenoptera	Chalcidoidea	Agaonidae	Ceratosolen	sp.	MK241705.1
Hymenoptera	Chalcidoidea	Agaonidae	Ceratosolen	sp.	MK241707.1
Hymenoptera	Chalcidoidea	Agaonidae	Ceratosolen	sp.	MK241708.1
Hymenoptera	Chalcidoidea	Agaonidae	Ceratosolen	sp.	MK241710.1
Hymenoptera	Chalcidoidea	Agaonidae	Ceratosolen	sp.	MK241711.1
Hymenoptera	Chalcidoidea	Agaonidae	Ceratosolen	sp.	MK241712.1
Hymenoptera	Chalcidoidea	Agaonidae	Ceratosolen	sp.	MK241713.1
Hymenoptera	Chalcidoidea	Agaonidae	Ceratosolen	sp.	MK241714.1
Hymenoptera	Chalcidoidea	Agaonidae	Ceratosolen	sp.	MK241715.1
Hymenoptera	Chalcidoidea	Agaonidae	Ceratosolen	sp.	MK241717.1
Hymenoptera	Chalcidoidea	Agaonidae	Ceratosolen	sp.	MK241721.1
Hymenoptera	Chalcidoidea	Agaonidae	Ceratosolen	sp.	MK241722.1
Hymenoptera	Chalcidoidea	Agaonidae	Ceratosolen	sp.	MK241723.1
Hymenoptera	Chalcidoidea	Agaonidae	Ceratosolen	sp.	MK241724.1
Hymenoptera	Chalcidoidea	Agaonidae	Ceratosolen	sp.	MK241725.1
Hymenoptera	Chalcidoidea	Agaonidae	Ceratosolen	sp.	MK241726.1
Hymenoptera	Chalcidoidea	Agaonidae	Ceratosolen	sp.	MK241728.1
Hymenoptera	Chalcidoidea	Agaonidae	Ceratosolen	sp.	MK241730.1
Hymenoptera	Chalcidoidea	Agaonidae	Ceratosolen	sp.	MK241731.1
Hymenoptera	Chalcidoidea	Agaonidae	Ceratosolen	sp.	MK241732.1
Hymenoptera	Chalcidoidea	Agaonidae	Ceratosolen	sp.	MK241733.1
Hymenoptera	Chalcidoidea	Agaonidae	Ceratosolen	sp.	MK241734.1
Hymenoptera	Chalcidoidea	Agaonidae	Ceratosolen	sp.	MK241735.1
Hymenoptera	Chalcidoidea	Agaonidae	Ceratosolen	sp.	MK241736.1
Hymenoptera	Chalcidoidea	Agaonidae	Ceratosolen	sp.	MK241737.1
Hymenoptera	Chalcidoidea	Agaonidae	Ceratosolen	sp.	MK241740.1
Hymenoptera	Chalcidoidea	Agaonidae	Ceratosolen	sp.	MK241741.1
Hymenoptera	Chalcidoidea	Agaonidae	Ceratosolen	sp.	MK241742.1
Hymenoptera	Chalcidoidea	Agaonidae	Ceratosolen	sp.	MK241743.1
Hymenoptera	Chalcidoidea	Agaonidae	Ceratosolen	sp.	MK241745.1
Hymenoptera	Chalcidoidea	Agaonidae	Ceratosolen	sp.	MK241746.1
Hymenoptera	Chalcidoidea	Agaonidae	Ceratosolen	sp.	MK241747.1
Hymenoptera	Chalcidoidea	Agaonidae	Ceratosolen	sp.	MK241748.1
Hymenoptera	Chalcidoidea	Agaonidae	Ceratosolen	sp.	MK241749.1
Hymenoptera	Chalcidoidea	Agaonidae	Ceratosolen	sp.	MK241751.1
Hymenoptera	Chalcidoidea	Agaonidae	Ceratosolen	sp.	MK241752.1
Hymenoptera	Chalcidoidea	Agaonidae	Ceratosolen	sp.	MK241753.1
Hymenoptera	Chalcidoidea	Agaonidae	Ceratosolen	sp.	MK241754.1
Hymenoptera	Chalcidoidea	Agaonidae	Ceratosolen	sp.	MK241755.1
Hymenoptera	Chalcidoidea	Agaonidae	Ceratosolen	sp.	MK241756.1

Hymenoptera	Chalcidoidea	Agaonidae	Ceratosolen	sp.	MK241757.1
Hymenoptera	Chalcidoidea	Agaonidae	Ceratosolen	sp.	MK241759.1
Hymenoptera	Chalcidoidea	Agaonidae	Ceratosolen	sp.	MK241760.1
Hymenoptera	Chalcidoidea	Agaonidae	Ceratosolen	sp.	MK241761.1
Hymenoptera	Chalcidoidea	Agaonidae	Ceratosolen	sp.	MK241762.1
Hymenoptera	Chalcidoidea	Agaonidae	Ceratosolen	sp.	MK241763.1
Hymenoptera	Chalcidoidea	Agaonidae	Ceratosolen	sp.	MK241764.1
Hymenoptera	Chalcidoidea	Agaonidae	Ceratosolen	sp.	MK241765.1
Hymenoptera	Chalcidoidea	Agaonidae	Ceratosolen	sp.	MK543357.1
Hymenoptera	Chalcidoidea	Agaonidae	Ceratosolen	sp.	MK543358.1
Hymenoptera	Chalcidoidea	Agaonidae	Ceratosolen	sp.	MK543367.1
Hymenoptera	Chalcidoidea	Agaonidae	Ceratosolen	sp.	MK543368.1
Hymenoptera	Chalcidoidea	Agaonidae	Ceratosolen	sp.	MK543369.1
Hymenoptera	Chalcidoidea	Agaonidae	Ceratosolen	waliensis	JN103250.1
Hymenoptera	Chalcidoidea	Agaonidae	Courtella	bekiliensis	JN103268.1
Hymenoptera	Chalcidoidea	Agaonidae	Deilagaon	aulatae	MK543400.1
Hymenoptera	Chalcidoidea	Agaonidae	Deilagaon	aulatae	MK543401.1
Hymenoptera	Chalcidoidea	Agaonidae	Deilagaon	aulatae	MK543403.1
Hymenoptera	Chalcidoidea	Agaonidae	Dolichoris	sp.	GQ367869.1
Hymenoptera	Chalcidoidea	Agaonidae	Dolichoris	sp.	GQ367871.1
Hymenoptera	Chalcidoidea	Agaonidae	Dolichoris	sp.	GQ367879.1
Hymenoptera	Chalcidoidea	Agaonidae	Dolichoris	sp.	JQ256523.1
Hymenoptera	Chalcidoidea	Agaonidae	Dolichoris	sp.	JQ256528.1
Hymenoptera	Chalcidoidea	Agaonidae	Dolichoris	sp.	JQ256529.1
Hymenoptera	Chalcidoidea	Agaonidae	Dolichoris	sp.	JQ256530.1
Hymenoptera	Chalcidoidea	Agaonidae	Dolichoris	sp.	JQ256531.1
Hymenoptera	Chalcidoidea	Agaonidae	Dolichoris	sp.	JQ256532.1
Hymenoptera	Chalcidoidea	Agaonidae	Dolichoris	sp.	JQ256533.1
Hymenoptera	Chalcidoidea	Agaonidae	Dolichoris	sp.	JQ256535.1
Hymenoptera	Chalcidoidea	Agaonidae	Dolichoris	sp.	JQ256536.1
Hymenoptera	Chalcidoidea	Agaonidae	Dolichoris	sp.	JQ256537.1
Hymenoptera	Chalcidoidea	Agaonidae	Dolichoris	sp.	JQ256540.1
Hymenoptera	Chalcidoidea	Agaonidae	Dolichoris	sp.	JQ256541.1
Hymenoptera	Chalcidoidea	Agaonidae	Dolichoris	sp.	JQ256543.1
Hymenoptera	Chalcidoidea	Agaonidae	Dolichoris	sp.	JQ256545.1
Hymenoptera	Chalcidoidea	Agaonidae	Dolichoris	sp.	JQ256548.1
Hymenoptera	Chalcidoidea	Agaonidae	Dolichoris	sp.	JQ256550.1
Hymenoptera	Chalcidoidea	Agaonidae	Dolichoris	sp.	JQ256561.1
Hymenoptera	Chalcidoidea	Agaonidae	Elisabethiella	glumosae	GQ367938.1
Hymenoptera	Chalcidoidea	Agaonidae	Elisabethiella	platyscapa	GQ367964.1
Hymenoptera	Chalcidoidea	Agaonidae	Elisabethiella	reflexa	GQ367913.1
Hymenoptera	Chalcidoidea	Agaonidae	Eukoebelea	cunia	MK530774.1
Hymenoptera	Chalcidoidea	Agaonidae	Eukoebelea	sp.	HM770641.1
Hymenoptera	Chalcidoidea	Agaonidae	Eukoebelea	sp.	JN001573.1
Hymenoptera	Chalcidoidea	Agaonidae	Eupristina	altissima	MK543370.1
Hymenoptera	Chalcidoidea	Agaonidae	Eupristina	altissima	MK559358.1
Hymenoptera	Chalcidoidea	Agaonidae	Eupristina	sp.	JN103274.1
Hymenoptera	Chalcidoidea	Agaonidae	Eupristina	koningsbergeri	MK543373.1
Hymenoptera	Chalcidoidea	Agaonidae	Eupristina	koningsbergeri	MK543374.1
Hymenoptera	Chalcidoidea	Agaonidae	Eupristina	koningsbergeri	MK543375.1
Hymenoptera	Chalcidoidea	Agaonidae	Eupristina	koningsbergeri	MK543376.1
Hymenoptera	Chalcidoidea	Agaonidae	Eupristina	koningsbergeri	MK543378.1
Hymenoptera	Chalcidoidea	Agaonidae	Eupristina	sp.	KC311066.1
Hymenoptera	Chalcidoidea	Agaonidae	Eupristina	sp.	KC311067.1

Hymenoptera	Chalcidoidea	Agaonidae	Eupristina	sp.	KC311068.1
Hymenoptera	Chalcidoidea	Agaonidae	Eupristina	sp.	KC311069.1
Hymenoptera	Chalcidoidea	Agaonidae	Eupristina	sp.	KC311071.1
Hymenoptera	Chalcidoidea	Agaonidae	Eupristina	sp.	KC311073.1
Hymenoptera	Chalcidoidea	Agaonidae	Eupristina	sp.	KC311074.1
Hymenoptera	Chalcidoidea	Agaonidae	Eupristina	sp.	KC311075.1
Hymenoptera	Chalcidoidea	Agaonidae	Eupristina	sp.	KC311077.1
Hymenoptera	Chalcidoidea	Agaonidae	Eupristina	sp.	KC311078.1
Hymenoptera	Chalcidoidea	Agaonidae	Eupristina	sp.	KC311079.1
Hymenoptera	Chalcidoidea	Agaonidae	Eupristina	sp.	KC311080.1
Hymenoptera	Chalcidoidea	Agaonidae	Eupristina	sp.	KC311082.1
Hymenoptera	Chalcidoidea	Agaonidae	Eupristina	sp.	KC311083.1
Hymenoptera	Chalcidoidea	Agaonidae	Eupristina	sp.	KC311084.1
Hymenoptera	Chalcidoidea	Agaonidae	Eupristina	sp.	KC311085.1
Hymenoptera	Chalcidoidea	Agaonidae	Eupristina	sp.	KC311086.1
Hymenoptera	Chalcidoidea	Agaonidae	Eupristina	sp.	KC311087.1
Hymenoptera	Chalcidoidea	Agaonidae	Eupristina	sp.	KC311088.1
Hymenoptera	Chalcidoidea	Agaonidae	Eupristina	sp.	KC311090.1
Hymenoptera	Chalcidoidea	Agaonidae	Eupristina	sp.	KC311091.1
Hymenoptera	Chalcidoidea	Agaonidae	Eupristina	sp.	KC311092.1
Hymenoptera	Chalcidoidea	Agaonidae	Eupristina	sp.	KC311093.1
Hymenoptera	Chalcidoidea	Agaonidae	Eupristina	sp.	KC311094.1
Hymenoptera	Chalcidoidea	Agaonidae	Eupristina	sp.	KC311097.1
Hymenoptera	Chalcidoidea	Agaonidae	Eupristina	sp.	KC311098.1
Hymenoptera	Chalcidoidea	Agaonidae	Eupristina	sp.	KC311099.1
Hymenoptera	Chalcidoidea	Agaonidae	Eupristina	sp.	KC311100.1
Hymenoptera	Chalcidoidea	Agaonidae	Eupristina	sp.	KC311101.1
Hymenoptera	Chalcidoidea	Agaonidae	Eupristina	sp.	KC311102.1
Hymenoptera	Chalcidoidea	Agaonidae	Eupristina	sp.	KC311103.1
Hymenoptera	Chalcidoidea	Agaonidae	Eupristina	sp.	KC311104.1
Hymenoptera	Chalcidoidea	Agaonidae	Eupristina	sp.	KC311105.1
Hymenoptera	Chalcidoidea	Agaonidae	Eupristina	sp.	KC311106.1
Hymenoptera	Chalcidoidea	Agaonidae	Eupristina	sp.	KC311107.1
Hymenoptera	Chalcidoidea	Agaonidae	Eupristina	sp.	KC311108.1
Hymenoptera	Chalcidoidea	Agaonidae	Eupristina	sp.	KC311109.1
Hymenoptera	Chalcidoidea	Agaonidae	Eupristina	sp.	KC311110.1
Hymenoptera	Chalcidoidea	Agaonidae	Eupristina	sp.	KC311111.1
Hymenoptera	Chalcidoidea	Agaonidae	Eupristina	sp.	KC311112.1
Hymenoptera	Chalcidoidea	Agaonidae	Eupristina	sp.	KC311115.1
Hymenoptera	Chalcidoidea	Agaonidae	Eupristina	sp.	KC311117.1
Hymenoptera	Chalcidoidea	Agaonidae	Eupristina	sp.	KC311120.1
Hymenoptera	Chalcidoidea	Agaonidae	Eupristina	sp.	KC311124.1
Hymenoptera	Chalcidoidea	Agaonidae	Eupristina	sp.	KC311128.1
Hymenoptera	Chalcidoidea	Agaonidae	Eupristina	sp.	KC311130.1
Hymenoptera	Chalcidoidea	Agaonidae	Eupristina	sp.	KC311131.1
Hymenoptera	Chalcidoidea	Agaonidae	Eupristina	sp.	KC311134.1
Hymenoptera	Chalcidoidea	Agaonidae	Eupristina	sp.	KC311135.1
Hymenoptera	Chalcidoidea	Agaonidae	Eupristina	sp.	KC311137.1
Hymenoptera	Chalcidoidea	Agaonidae	Eupristina	sp.	KC311138.1
Hymenoptera	Chalcidoidea	Agaonidae	Eupristina	sp.	KC311140.1
Hymenoptera	Chalcidoidea	Agaonidae	Eupristina	sp.	KC311144.1
Hymenoptera	Chalcidoidea	Agaonidae	Eupristina	sp.	MK543379.1
Hymenoptera	Chalcidoidea	Agaonidae	Eupristina	sp.	MK543380.1
Hymenoptera	Chalcidoidea	Agaonidae	Eupristina	sp.	MK543383.1

Hymenoptera	Chalcidoidea	Agaonidae	Eupristina	sp.	MK543384.1
Hymenoptera	Chalcidoidea	Agaonidae	Eupristina	sp.	MK543387.1
Hymenoptera	Chalcidoidea	Agaonidae	Eupristina	sp.	MK543388.1
Hymenoptera	Chalcidoidea	Agaonidae	Eupristina	sp.	MK543419.1
Hymenoptera	Chalcidoidea	Agaonidae	Eupristina	sp.	MK543420.1
Hymenoptera	Chalcidoidea	Agaonidae	Eupristina	sp.	MK543421.1
Hymenoptera	Chalcidoidea	Agaonidae	Eupristina	sp.	MK559359.1
Hymenoptera	Chalcidoidea	Agaonidae	Eupristina	verticillata	HM582244.1
Hymenoptera	Chalcidoidea	Agaonidae	Eupristina	verticillata	HM582245.1
Hymenoptera	Chalcidoidea	Agaonidae	Eupristina	verticillata	HM582246.1
Hymenoptera	Chalcidoidea	Agaonidae	Eupristina	verticillata	HM582247.1
Hymenoptera	Chalcidoidea	Agaonidae	Eupristina	verticillata	HM582248.1
Hymenoptera	Chalcidoidea	Agaonidae	Eupristina	verticillata	HM582249.1
Hymenoptera	Chalcidoidea	Agaonidae	Eupristina	verticillata	HM582250.1
Hymenoptera	Chalcidoidea	Agaonidae	Eupristina	verticillata	HM582251.1
Hymenoptera	Chalcidoidea	Agaonidae	Eupristina	verticillata	HM582252.1
Hymenoptera	Chalcidoidea	Agaonidae	Eupristina	verticillata	HM582253.1
Hymenoptera	Chalcidoidea	Agaonidae	Eupristina	verticillata	HM582254.1
Hymenoptera	Chalcidoidea	Agaonidae	Eupristina	verticillata	HM582257.1
Hymenoptera	Chalcidoidea	Agaonidae	Eupristina	verticillata	HM582258.1
Hymenoptera	Chalcidoidea	Agaonidae	Eupristina	verticillata	HM582259.1
Hymenoptera	Chalcidoidea	Agaonidae	Eupristina	verticillata	HM582260.1
Hymenoptera	Chalcidoidea	Agaonidae	Eupristina	verticillata	HM582261.1
Hymenoptera	Chalcidoidea	Agaonidae	Eupristina	verticillata	HM582262.1
Hymenoptera	Chalcidoidea	Agaonidae	Eupristina	verticillata	HM582263.1
Hymenoptera	Chalcidoidea	Agaonidae	Eupristina	verticillata	MK543392.1
Hymenoptera	Chalcidoidea	Agaonidae	Eupristina	verticillata	MK543394.1
Hymenoptera	Chalcidoidea	Agaonidae	Idarnes	brevis	KY210171.1
Hymenoptera	Chalcidoidea	Agaonidae	Idarnes	flavicollis	KY210168.1
Hymenoptera	Chalcidoidea	Agaonidae	Idarnes	flavicollis	MG214353.1
Hymenoptera	Chalcidoidea	Agaonidae	Idarnes	flavicollis	MG547434.1
Hymenoptera	Chalcidoidea	Agaonidae	Idarnes	flavicollis	MG547438.1
Hymenoptera	Chalcidoidea	Agaonidae	Idarnes	flavicollis	MG547439.1
Hymenoptera	Chalcidoidea	Agaonidae	Idarnes	flavicollis	MG547454.1
Hymenoptera	Chalcidoidea	Agaonidae	Idarnes	flavicollis	MG547455.1
Hymenoptera	Chalcidoidea	Agaonidae	Idarnes	sp.	HM770611.1
Hymenoptera	Chalcidoidea	Agaonidae	Idarnes	sp.	HM770612.1
Hymenoptera	Chalcidoidea	Agaonidae	Idarnes	sp.	HM770621.1
Hymenoptera	Chalcidoidea	Agaonidae	Idarnes	sp.	HM770622.1
Hymenoptera	Chalcidoidea	Agaonidae	Idarnes	sp.	HM770623.1
Hymenoptera	Chalcidoidea	Agaonidae	Idarnes	sp.	HM770625.1
Hymenoptera	Chalcidoidea	Agaonidae	Idarnes	sp.	HM770643.1
Hymenoptera	Chalcidoidea	Agaonidae	Idarnes	sp.	HM770644.1
Hymenoptera	Chalcidoidea	Agaonidae	Idarnes	sp.	HM770645.1
Hymenoptera	Chalcidoidea	Agaonidae	Idarnes	sp.	HM770646.1
Hymenoptera	Chalcidoidea	Agaonidae	Idarnes	sp.	HM770650.1
Hymenoptera	Chalcidoidea	Agaonidae	Idarnes	sp.	HM770651.1
Hymenoptera	Chalcidoidea	Agaonidae	Idarnes	sp.	HM770652.1
Hymenoptera	Chalcidoidea	Agaonidae	Idarnes	sp.	JN001524.1
Hymenoptera	Chalcidoidea	Agaonidae	Idarnes	sp.	JN001525.1
Hymenoptera	Chalcidoidea	Agaonidae	Idarnes	sp.	JN001532.1
Hymenoptera	Chalcidoidea	Agaonidae	Idarnes	sp.	JN001538.1
Hymenoptera	Chalcidoidea	Agaonidae	Idarnes	sp.	JN001539.1
Hymenoptera	Chalcidoidea	Agaonidae	Idarnes	sp.	JN001542.1

Hymenoptera	Chalcidoidea	Agaonidae	Idarnes	sp.	JN001543.1
Hymenoptera	Chalcidoidea	Agaonidae	Idarnes	sp.	JN001549.1
Hymenoptera	Chalcidoidea	Agaonidae	Idarnes	sp.	JN001559.1
Hymenoptera	Chalcidoidea	Agaonidae	Idarnes	sp.	JN001560.1
Hymenoptera	Chalcidoidea	Agaonidae	Idarnes	sp.	JN001583.1
Hymenoptera	Chalcidoidea	Agaonidae	Idarnes	sp.	MG214354.1
Hymenoptera	Chalcidoidea	Agaonidae	Idarnes	sp.	MG547440.1
Hymenoptera	Chalcidoidea	Agaonidae	Idarnes	sp.	MG547441.1
Hymenoptera	Chalcidoidea	Agaonidae	Idarnes	sp.	MG547446.1
Hymenoptera	Chalcidoidea	Agaonidae	Idarnes	sp.	MG547448.1
Hymenoptera	Chalcidoidea	Agaonidae	Idarnes	sp.	MG547449.1
Hymenoptera	Chalcidoidea	Agaonidae	Idarnes	sp.	MG547450.1
Hymenoptera	Chalcidoidea	Agaonidae	Idarnes	sp.	MG547451.1
Hymenoptera	Chalcidoidea	Agaonidae	Idarnes	sp.	MG547452.1
Hymenoptera	Chalcidoidea	Agaonidae	Idarnes	sp.	MG547453.1
Hymenoptera	Chalcidoidea	Agaonidae	Kradibia	giacominii	JN103271.1
Hymenoptera	Chalcidoidea	Agaonidae	Kradibia	sp.	GQ367874.1
Hymenoptera	Chalcidoidea	Agaonidae	Kradibia	sp.	GQ367920.1
Hymenoptera	Chalcidoidea	Agaonidae	Kradibia	sp.	GQ367922.1
Hymenoptera	Chalcidoidea	Agaonidae	Kradibia	sp.	GQ367931.1
Hymenoptera	Chalcidoidea	Agaonidae	Kradibia	sp.	JN103275.1
Hymenoptera	Chalcidoidea	Agaonidae	Pegoscapus	aguilari	GQ367891.1
Hymenoptera	Chalcidoidea	Agaonidae	Pegoscapus	sp.	JN103284.1
Hymenoptera	Chalcidoidea	Agaonidae	Pegoscapus	sp.	JN103286.1
Hymenoptera	Chalcidoidea	Agaonidae	Pegoscapus	herrei	JN103251.1
Hymenoptera	Chalcidoidea	Agaonidae	Pegoscapus	sp.	GQ367911.1
Hymenoptera	Chalcidoidea	Agaonidae	Pegoscapus	orozcoi	JN103294.1
Hymenoptera	Chalcidoidea	Agaonidae	Pegoscapus	sp.	JN103282.1
Hymenoptera	Chalcidoidea	Agaonidae	Pegoscapus	sp.	JN103329.1
Hymenoptera	Chalcidoidea	Agaonidae	Pegoscapus	sp.	MG214355.1
Hymenoptera	Chalcidoidea	Agaonidae	Platyneura	mayri	MK530779.1
Hymenoptera	Chalcidoidea	Agaonidae	Platyneura	mayri	MK543434.1
Hymenoptera	Chalcidoidea	Agaonidae	Platyscapa	coronata	GQ367885.1
Hymenoptera	Chalcidoidea	Agaonidae	Platyscapa	sp.	GQ367896.1
Hymenoptera	Chalcidoidea	Agaonidae	Platyscapa	sp.	GQ367903.1
Hymenoptera	Chalcidoidea	Agaonidae	Platyscapa	sp.	GQ367910.1
Hymenoptera	Chalcidoidea	Agaonidae	Platyscapa	quadraticeps	GQ367908.1
Hymenoptera	Chalcidoidea	Agaonidae	Platyscapa	quadraticeps	MK543412.1
Hymenoptera	Chalcidoidea	Agaonidae	Platyscapa	quadraticeps	MK543413.1
Hymenoptera	Chalcidoidea	Agaonidae	Platyscapa	quadraticeps	MK559360.1
Hymenoptera	Chalcidoidea	Agaonidae	Platyscapa	sp.	MK543408.1
Hymenoptera	Chalcidoidea	Agaonidae	Platyscapa	sp.	MK543409.1
Hymenoptera	Chalcidoidea	Agaonidae	Platyscapa	sp.	MK543410.1
Hymenoptera	Chalcidoidea	Agaonidae	Platyscapa	sp.	MK543411.1
Hymenoptera	Chalcidoidea	Agaonidae	Platyscapa	sp.	MK543416.1
Hymenoptera	Chalcidoidea	Agaonidae	Platyscapa	sp.	MK543418.1
Hymenoptera	Chalcidoidea	Agaonidae	Pleistodontes	addicotti	GQ367887.1
Hymenoptera	Chalcidoidea	Agaonidae	Pleistodontes	froggatti	GQ367883.1
Hymenoptera	Chalcidoidea	Agaonidae	Pleistodontes	greenwoodi	GQ367889.1
Hymenoptera	Chalcidoidea	Agaonidae	Pleistodontes	nitens	GQ367886.1
Hymenoptera	Chalcidoidea	Agaonidae	Pleistodontes	regalis	JN103255.1
Hymenoptera	Chalcidoidea	Agaonidae	Sycophaga	cyclostigma	HM770628.1
Hymenoptera	Chalcidoidea	Agaonidae	Sycophaga	cyclostigma	JN001558.1
Hymenoptera	Chalcidoidea	Agaonidae	Sycophaga	sycomori	MG214359.1

Hymenoptera	Chalcidoidea	Agaonidae	Sycophaga	testacea	MK530780.1
Hymenoptera	Chalcidoidea	Agaonidae	Sycophaga	testacea	MK530781.1
Hymenoptera	Chalcidoidea	Agaonidae	Sycophaga	testacea	MK530782.1
Hymenoptera	Chalcidoidea	Agaonidae	Sycophaga	testacea	MK530783.1
Hymenoptera	Chalcidoidea	Agaonidae	Sycophaga	testacea	MK530784.1
Hymenoptera	Chalcidoidea	Agaonidae	Tetrapus	costaricanus	JN103266.1
Hymenoptera	Chalcidoidea	Agaonidae	Tetrapus	sp.	GQ367942.1
Hymenoptera	Chalcidoidea	Agaonidae	Valisia	compacta	JN103276.1
Hymenoptera	Chalcidoidea	Agaonidae	Valisia	malayana	JN103318.1
Hymenoptera	Chalcidoidea	Agaonidae	Waterstoniella	sp.	GQ367918.1
Hymenoptera	Chalcidoidea	Agaonidae	Waterstoniella	sp.	GQ367919.1
Hymenoptera	Chalcidoidea	Agaonidae	Waterstoniella	sp.	GQ367926.1
Hymenoptera	Chalcidoidea	Agaonidae	Waterstoniella	sp.	GQ367951.1
Hymenoptera	Chalcidoidea	Agaonidae	Waterstoniella	sp.	MK543428.1
Hymenoptera	Chalcidoidea	Agaonidae	Wiebesia	boldinghi	JN103288.1
Hymenoptera	Chalcidoidea	Agaonidae	Wiebesia	contubernalis	GQ367929.1
Hymenoptera	Chalcidoidea	Agaonidae	Wiebesia	flava	JN103249.1
Hymenoptera	Chalcidoidea	Agaonidae	Wiebesia	sp.	JN103316.1
Hymenoptera	Chalcidoidea	Aphelinidae	Aphelinidae	sp.	HM423379.1
Hymenoptera	Chalcidoidea	Aphelinidae	Aphelinidae	sp.	HQ930362.1
Hymenoptera	Chalcidoidea	Aphelinidae	Aphelinidae	sp.	HQ930366.1
Hymenoptera	Chalcidoidea	Aphelinidae	Aphelinidae	sp.	JF863291.1
Hymenoptera	Chalcidoidea	Aphelinidae	Aphelinidae	sp.	KJ085912.1
Hymenoptera	Chalcidoidea	Aphelinidae	Aphelinidae	sp.	KJ091348.1
Hymenoptera	Chalcidoidea	Aphelinidae	Aphelinidae	sp.	KJ164271.1
Hymenoptera	Chalcidoidea	Aphelinidae	Aphelinidae	sp.	KJ165959.1
Hymenoptera	Chalcidoidea	Aphelinidae	Aphelinidae	sp.	KJ167893.1
Hymenoptera	Chalcidoidea	Aphelinidae	Aphelinidae	sp.	KJ207750.1
Hymenoptera	Chalcidoidea	Aphelinidae	Aphelinidae	sp.	KJ637614.1
Hymenoptera	Chalcidoidea	Aphelinidae	Aphelinidae	sp.	KR365120.1
Hymenoptera	Chalcidoidea	Aphelinidae	Aphelinidae	sp.	KR365862.1
Hymenoptera	Chalcidoidea	Aphelinidae	Aphelinidae	sp.	KR366383.1
Hymenoptera	Chalcidoidea	Aphelinidae	Aphelinidae	sp.	KR368189.1
Hymenoptera	Chalcidoidea	Aphelinidae	Aphelinidae	sp.	KR369205.1
Hymenoptera	Chalcidoidea	Aphelinidae	Aphelinidae	sp.	KR369841.1
Hymenoptera	Chalcidoidea	Aphelinidae	Aphelinidae	sp.	KR372597.1
Hymenoptera	Chalcidoidea	Aphelinidae	Aphelinidae	sp.	KR401855.1
Hymenoptera	Chalcidoidea	Aphelinidae	Aphelinidae	sp.	KR404586.1
Hymenoptera	Chalcidoidea	Aphelinidae	Aphelinidae	sp.	KR406458.1
Hymenoptera	Chalcidoidea	Aphelinidae	Aphelinidae	sp.	KR406924.1
Hymenoptera	Chalcidoidea	Aphelinidae	Aphelinidae	sp.	KR407389.1
Hymenoptera	Chalcidoidea	Aphelinidae	Aphelinidae	sp.	KR415104.1
Hymenoptera	Chalcidoidea	Aphelinidae	Aphelinidae	sp.	KR416216.1
Hymenoptera	Chalcidoidea	Aphelinidae	Aphelinidae	sp.	KR416515.1
Hymenoptera	Chalcidoidea	Aphelinidae	Aphelinidae	sp.	KR419956.1
Hymenoptera	Chalcidoidea	Aphelinidae	Aphelinidae	sp.	KR419961.1
Hymenoptera	Chalcidoidea	Aphelinidae	Aphelinidae	sp.	KR420158.1
Hymenoptera	Chalcidoidea	Aphelinidae	Aphelinidae	sp.	KR783058.1
Hymenoptera	Chalcidoidea	Aphelinidae	Aphelinidae	sp.	KR783678.1
Hymenoptera	Chalcidoidea	Aphelinidae	Aphelinidae	sp.	KR784217.1
Hymenoptera	Chalcidoidea	Aphelinidae	Aphelinidae	sp.	KR784248.1
Hymenoptera	Chalcidoidea	Aphelinidae	Aphelinidae	sp.	KR784595.1
Hymenoptera	Chalcidoidea	Aphelinidae	Aphelinidae	sp.	KR785369.1
Hymenoptera	Chalcidoidea	Aphelinidae	Aphelinidae	sp.	KR787915.1

Hymenoptera	Chalcidoidea	Aphelinidae	Aphelinidae	sp.	KR788648.1
Hymenoptera	Chalcidoidea	Aphelinidae	Aphelinidae	sp.	KR789167.1
Hymenoptera	Chalcidoidea	Aphelinidae	Aphelinidae	sp.	KR789238.1
Hymenoptera	Chalcidoidea	Aphelinidae	Aphelinidae	sp.	KR790179.1
Hymenoptera	Chalcidoidea	Aphelinidae	Aphelinidae	sp.	KR791695.1
Hymenoptera	Chalcidoidea	Aphelinidae	Aphelinidae	sp.	KR793320.1
Hymenoptera	Chalcidoidea	Aphelinidae	Aphelinidae	sp.	KR795163.1
Hymenoptera	Chalcidoidea	Aphelinidae	Aphelinidae	sp.	KR795187.1
Hymenoptera	Chalcidoidea	Aphelinidae	Aphelinidae	sp.	KR795636.1
Hymenoptera	Chalcidoidea	Aphelinidae	Aphelinidae	sp.	KR797433.1
Hymenoptera	Chalcidoidea	Aphelinidae	Aphelinidae	sp.	KR797595.1
Hymenoptera	Chalcidoidea	Aphelinidae	Aphelinidae	sp.	KR799474.1
Hymenoptera	Chalcidoidea	Aphelinidae	Aphelinidae	sp.	KR800851.1
Hymenoptera	Chalcidoidea	Aphelinidae	Aphelinidae	sp.	KR801164.1
Hymenoptera	Chalcidoidea	Aphelinidae	Aphelinidae	sp.	KR801527.1
Hymenoptera	Chalcidoidea	Aphelinidae	Aphelinidae	sp.	KR801584.1
Hymenoptera	Chalcidoidea	Aphelinidae	Aphelinidae	sp.	KR801827.1
Hymenoptera	Chalcidoidea	Aphelinidae	Aphelinidae	sp.	KR802665.1
Hymenoptera	Chalcidoidea	Aphelinidae	Aphelinidae	sp.	KR803876.1
Hymenoptera	Chalcidoidea	Aphelinidae	Aphelinidae	sp.	KR804169.1
Hymenoptera	Chalcidoidea	Aphelinidae	Aphelinidae	sp.	KR805033.1
Hymenoptera	Chalcidoidea	Aphelinidae	Aphelinidae	sp.	KR805876.1
Hymenoptera	Chalcidoidea	Aphelinidae	Aphelinidae	sp.	KR806465.1
Hymenoptera	Chalcidoidea	Aphelinidae	Aphelinidae	sp.	KR807281.1
Hymenoptera	Chalcidoidea	Aphelinidae	Aphelinidae	sp.	KR807920.1
Hymenoptera	Chalcidoidea	Aphelinidae	Aphelinidae	sp.	KR873857.1
Hymenoptera	Chalcidoidea	Aphelinidae	Aphelinidae	sp.	KR874893.1
Hymenoptera	Chalcidoidea	Aphelinidae	Aphelinidae	sp.	KR875395.1
Hymenoptera	Chalcidoidea	Aphelinidae	Aphelinidae	sp.	KR877989.1
Hymenoptera	Chalcidoidea	Aphelinidae	Aphelinidae	sp.	KR878736.1
Hymenoptera	Chalcidoidea	Aphelinidae	Aphelinidae	sp.	KR880067.1
Hymenoptera	Chalcidoidea	Aphelinidae	Aphelinidae	sp.	KR880295.1
Hymenoptera	Chalcidoidea	Aphelinidae	Aphelinidae	sp.	KR881755.1
Hymenoptera	Chalcidoidea	Aphelinidae	Aphelinidae	sp.	KR882177.1
Hymenoptera	Chalcidoidea	Aphelinidae	Aphelinidae	sp.	KR883112.1
Hymenoptera	Chalcidoidea	Aphelinidae	Aphelinidae	sp.	KR883531.1
Hymenoptera	Chalcidoidea	Aphelinidae	Aphelinidae	sp.	KR883892.1
Hymenoptera	Chalcidoidea	Aphelinidae	Aphelinidae	sp.	KR885297.1
Hymenoptera	Chalcidoidea	Aphelinidae	Aphelinidae	sp.	KR887647.1
Hymenoptera	Chalcidoidea	Aphelinidae	Aphelinidae	sp.	KR887710.1
Hymenoptera	Chalcidoidea	Aphelinidae	Aphelinidae	sp.	KR888558.1
Hymenoptera	Chalcidoidea	Aphelinidae	Aphelinidae	sp.	KR888625.1
Hymenoptera	Chalcidoidea	Aphelinidae	Aphelinidae	sp.	KR889816.1
Hymenoptera	Chalcidoidea	Aphelinidae	Aphelinidae	sp.	KR890195.1
Hymenoptera	Chalcidoidea	Aphelinidae	Aphelinidae	sp.	KR891678.1
Hymenoptera	Chalcidoidea	Aphelinidae	Aphelinidae	sp.	KR894353.1
Hymenoptera	Chalcidoidea	Aphelinidae	Aphelinidae	sp.	KR897864.1
Hymenoptera	Chalcidoidea	Aphelinidae	Aphelinidae	sp.	KR899122.1
Hymenoptera	Chalcidoidea	Aphelinidae	Aphelinidae	sp.	KR899946.1
Hymenoptera	Chalcidoidea	Aphelinidae	Aphelinidae	sp.	KR899972.1
Hymenoptera	Chalcidoidea	Aphelinidae	Aphelinidae	sp.	KR900208.1
Hymenoptera	Chalcidoidea	Aphelinidae	Aphelinidae	sp.	KR900761.1
Hymenoptera	Chalcidoidea	Aphelinidae	Aphelinidae	sp.	KR925278.1
Hymenoptera	Chalcidoidea	Aphelinidae	Aphelinidae	sp.	KR926008.1

Hymenoptera	Chalcidoidea	Aphelinidae	Aphelinidae	sp.	KR926598.1
Hymenoptera	Chalcidoidea	Aphelinidae	Aphelinidae	sp.	KR926672.1
Hymenoptera	Chalcidoidea	Aphelinidae	Aphelinidae	sp.	KR926741.1
Hymenoptera	Chalcidoidea	Aphelinidae	Aphelinidae	sp.	KR934445.1
Hymenoptera	Chalcidoidea	Aphelinidae	Aphelinidae	sp.	KT605723.1
Hymenoptera	Chalcidoidea	Aphelinidae	Aphelinidae	sp.	KT605740.1
Hymenoptera	Chalcidoidea	Aphelinidae	Aphelinidae	sp.	KT607356.1
Hymenoptera	Chalcidoidea	Aphelinidae	Aphelinidae	sp.	KY829976.1
Hymenoptera	Chalcidoidea	Aphelinidae	Aphelinidae	sp.	KY831279.1
Hymenoptera	Chalcidoidea	Aphelinidae	Aphelinidae	sp.	KY832778.1
Hymenoptera	Chalcidoidea	Aphelinidae	Aphelinidae	sp.	KY833387.1
Hymenoptera	Chalcidoidea	Aphelinidae	Aphelinidae	sp.	KY834357.1
Hymenoptera	Chalcidoidea	Aphelinidae	Aphelinidae	sp.	KY834630.1
Hymenoptera	Chalcidoidea	Aphelinidae	Aphelinidae	sp.	KY835007.1
Hymenoptera	Chalcidoidea	Aphelinidae	Aphelinidae	sp.	KY835266.1
Hymenoptera	Chalcidoidea	Aphelinidae	Aphelinidae	sp.	KY836211.1
Hymenoptera	Chalcidoidea	Aphelinidae	Aphelinidae	sp.	KY836844.1
Hymenoptera	Chalcidoidea	Aphelinidae	Aphelinidae	sp.	KY837290.1
Hymenoptera	Chalcidoidea	Aphelinidae	Aphelinidae	sp.	KY837683.1
Hymenoptera	Chalcidoidea	Aphelinidae	Aphelinidae	sp.	KY837890.1
Hymenoptera	Chalcidoidea	Aphelinidae	Aphelinidae	sp.	KY838224.1
Hymenoptera	Chalcidoidea	Aphelinidae	Aphelinidae	sp.	KY838862.1
Hymenoptera	Chalcidoidea	Aphelinidae	Aphelinidae	sp.	KY839324.1
Hymenoptera	Chalcidoidea	Aphelinidae	Aphelinidae	sp.	KY840488.1
Hymenoptera	Chalcidoidea	Aphelinidae	Aphelinidae	sp.	KY841370.1
Hymenoptera	Chalcidoidea	Aphelinidae	Aphelinidae	sp.	KY841601.1
Hymenoptera	Chalcidoidea	Aphelinidae	Aphelinidae	sp.	KY841953.1
Hymenoptera	Chalcidoidea	Aphelinidae	Aphelinidae	sp.	KY844488.1
Hymenoptera	Chalcidoidea	Aphelinidae	Aphelinidae	sp.	KY845133.1
Hymenoptera	Chalcidoidea	Aphelinidae	Aphelinidae	sp.	MF929198.1
Hymenoptera	Chalcidoidea	Aphelinidae	Aphelinidae	sp.	MF929666.1
Hymenoptera	Chalcidoidea	Aphelinidae	Aphelinidae	sp.	MF930056.1
Hymenoptera	Chalcidoidea	Aphelinidae	Aphelinidae	sp.	MF931204.1
Hymenoptera	Chalcidoidea	Aphelinidae	Aphelinidae	sp.	MF931298.1
Hymenoptera	Chalcidoidea	Aphelinidae	Aphelinidae	sp.	MF931414.1
Hymenoptera	Chalcidoidea	Aphelinidae	Aphelinidae	sp.	MF931920.1
Hymenoptera	Chalcidoidea	Aphelinidae	Aphelinidae	sp.	MF932081.1
Hymenoptera	Chalcidoidea	Aphelinidae	Aphelinidae	sp.	MF933219.1
Hymenoptera	Chalcidoidea	Aphelinidae	Aphelinidae	sp.	MF933340.1
Hymenoptera	Chalcidoidea	Aphelinidae	Aphelinidae	sp.	MF933527.1
Hymenoptera	Chalcidoidea	Aphelinidae	Aphelinidae	sp.	MF934181.1
Hymenoptera	Chalcidoidea	Aphelinidae	Aphelinidae	sp.	MF934522.1
Hymenoptera	Chalcidoidea	Aphelinidae	Aphelinidae	sp.	MF935087.1
Hymenoptera	Chalcidoidea	Aphelinidae	Aphelinidae	sp.	MF935119.1
Hymenoptera	Chalcidoidea	Aphelinidae	Aphelinidae	sp.	MF936287.1
Hymenoptera	Chalcidoidea	Aphelinidae	Aphelinidae	sp.	MF936538.1
Hymenoptera	Chalcidoidea	Aphelinidae	Aphelinidae	sp.	MF936798.1
Hymenoptera	Chalcidoidea	Aphelinidae	Aphelinidae	sp.	MF936808.1
Hymenoptera	Chalcidoidea	Aphelinidae	Aphelinidae	sp.	MF937214.1
Hymenoptera	Chalcidoidea	Aphelinidae	Aphelinidae	sp.	MF937594.1
Hymenoptera	Chalcidoidea	Aphelinidae	Aphelinidae	sp.	MF937881.1
Hymenoptera	Chalcidoidea	Aphelinidae	Aphelinidae	sp.	MF938636.1
Hymenoptera	Chalcidoidea	Aphelinidae	Aphelinidae	sp.	MF938672.1
Hymenoptera	Chalcidoidea	Aphelinidae	Aphelinidae	sp.	MF938911.1

Hymenoptera	Chalcidoidea	Aphelinidae	Aphelinidae	sp.	MG439710.1
Hymenoptera	Chalcidoidea	Aphelinidae	Aphelinidae	sp.	MG441396.1
Hymenoptera	Chalcidoidea	Aphelinidae	Aphelinidae	sp.	MG441713.1
Hymenoptera	Chalcidoidea	Aphelinidae	Aphelinidae	sp.	MG442490.1
Hymenoptera	Chalcidoidea	Aphelinidae	Aphelinidae	sp.	MG442614.1
Hymenoptera	Chalcidoidea	Aphelinidae	Aphelinidae	sp.	MG443818.1
Hymenoptera	Chalcidoidea	Aphelinidae	Aphelinidae	sp.	MG444035.1
Hymenoptera	Chalcidoidea	Aphelinidae	Aphelinidae	sp.	MG444076.1
Hymenoptera	Chalcidoidea	Aphelinidae	Aphelinidae	sp.	MG444386.1
Hymenoptera	Chalcidoidea	Aphelinidae	Aphelinidae	sp.	MG444411.1
Hymenoptera	Chalcidoidea	Aphelinidae	Aphelinidae	sp.	MG444465.1
Hymenoptera	Chalcidoidea	Aphelinidae	Aphelinidae	sp.	MG444563.1
Hymenoptera	Chalcidoidea	Aphelinidae	Aphelinidae	sp.	MG444841.1
Hymenoptera	Chalcidoidea	Aphelinidae	Aphelinidae	sp.	MG444874.1
Hymenoptera	Chalcidoidea	Aphelinidae	Aphelinidae	sp.	MG445060.1
Hymenoptera	Chalcidoidea	Aphelinidae	Aphelinidae	sp.	MG445179.1
Hymenoptera	Chalcidoidea	Aphelinidae	Aphelinidae	sp.	MG445954.1
Hymenoptera	Chalcidoidea	Aphelinidae	Aphelinidae	sp.	MG446652.1
Hymenoptera	Chalcidoidea	Aphelinidae	Aphelinidae	sp.	MG446944.1
Hymenoptera	Chalcidoidea	Aphelinidae	Aphelinidae	sp.	MG448265.1
Hymenoptera	Chalcidoidea	Aphelinidae	Aphelinidae	sp.	MG481277.1
Hymenoptera	Chalcidoidea	Aphelinidae	Aphelinidae	sp.	MH456580.1
Hymenoptera	Chalcidoidea	Aphelinidae	Aphelinidae	sp.	MH926387.1
Hymenoptera	Chalcidoidea	Aphelinidae	Aphelinidae	sp.	MH926513.1
Hymenoptera	Chalcidoidea	Aphelinidae	Aphelinidae	sp.	MH926535.1
Hymenoptera	Chalcidoidea	Aphelinidae	Aphelinidae	sp.	MH926549.1
Hymenoptera	Chalcidoidea	Aphelinidae	Aphelinidae	sp.	MH926871.1
Hymenoptera	Chalcidoidea	Aphelinidae	Aphelinidae	sp.	MH926938.1
Hymenoptera	Chalcidoidea	Aphelinidae	Aphelinidae	sp.	MH927634.1
Hymenoptera	Chalcidoidea	Aphelinidae	Aphelinidae	sp.	MH927642.1
Hymenoptera	Chalcidoidea	Aphelinidae	Aphelinidae	sp.	MH927644.1
Hymenoptera	Chalcidoidea	Aphelinidae	Aphelinidae	sp.	MH928566.1
Hymenoptera	Chalcidoidea	Aphelinidae	Aphelinidae	sp.	MH928593.1
Hymenoptera	Chalcidoidea	Aphelinidae	Aphelinidae	sp.	MH928670.1
Hymenoptera	Chalcidoidea	Aphelinidae	Aphelinidae	sp.	MH928787.1
Hymenoptera	Chalcidoidea	Aphelinidae	Aphelinidae	sp.	MH928858.1
Hymenoptera	Chalcidoidea	Aphelinidae	Aphelinus	abdominalis	FM210123.1
Hymenoptera	Chalcidoidea	Aphelinidae	Aphelinus	abdominalis	KR882786.1
Hymenoptera	Chalcidoidea	Aphelinidae	Aphelinus	abdominalis	KR895742.1
Hymenoptera	Chalcidoidea	Aphelinidae	Aphelinus	abdominalis	KY912640.1
Hymenoptera	Chalcidoidea	Aphelinidae	Aphelinus	abdominalis	KY912641.1
Hymenoptera	Chalcidoidea	Aphelinidae	Aphelinus	gossypii	LC260600.1
Hymenoptera	Chalcidoidea	Aphelinidae	Aphelinus	gossypii	LC260602.1
Hymenoptera	Chalcidoidea	Aphelinidae	Aphelinus	gossypii	LC260603.1
Hymenoptera	Chalcidoidea	Aphelinidae	Aphelinus	mali	KY912643.1
Hymenoptera	Chalcidoidea	Aphelinidae	Aphelinus	paramali	KR800372.1
Hymenoptera	Chalcidoidea	Aphelinidae	Aphelinus	paramali	KR801845.1
Hymenoptera	Chalcidoidea	Aphelinidae	Aphelinus	paramali	MF807204.1
Hymenoptera	Chalcidoidea	Aphelinidae	Aphelinus	sp.	KR402234.1
Hymenoptera	Chalcidoidea	Aphelinidae	Aphelinus	sp.	KR404211.1
Hymenoptera	Chalcidoidea	Aphelinidae	Aphelinus	sp.	KR409028.1
Hymenoptera	Chalcidoidea	Aphelinidae	Aphelinus	sp.	KR414169.1
Hymenoptera	Chalcidoidea	Aphelinidae	Aphelinus	sp.	KR414690.1
Hymenoptera	Chalcidoidea	Aphelinidae	Aphelinus	sp.	KR417511.1

Hymenoptera	Chalcidoidea	Aphelinidae	Aphelinus	sp.	KR418158.1
Hymenoptera	Chalcidoidea	Aphelinidae	Aphelinus	sp.	KR784049.1
Hymenoptera	Chalcidoidea	Aphelinidae	Aphelinus	sp.	KR785943.1
Hymenoptera	Chalcidoidea	Aphelinidae	Aphelinus	sp.	KR785988.1
Hymenoptera	Chalcidoidea	Aphelinidae	Aphelinus	sp.	KR787217.1
Hymenoptera	Chalcidoidea	Aphelinidae	Aphelinus	sp.	KR787849.1
Hymenoptera	Chalcidoidea	Aphelinidae	Aphelinus	sp.	KR788690.1
Hymenoptera	Chalcidoidea	Aphelinidae	Aphelinus	sp.	KR789217.1
Hymenoptera	Chalcidoidea	Aphelinidae	Aphelinus	sp.	KR789358.1
Hymenoptera	Chalcidoidea	Aphelinidae	Aphelinus	sp.	KR789711.1
Hymenoptera	Chalcidoidea	Aphelinidae	Aphelinus	sp.	KR790504.1
Hymenoptera	Chalcidoidea	Aphelinidae	Aphelinus	sp.	KR790959.1
Hymenoptera	Chalcidoidea	Aphelinidae	Aphelinus	sp.	KR792488.1
Hymenoptera	Chalcidoidea	Aphelinidae	Aphelinus	sp.	KR794621.1
Hymenoptera	Chalcidoidea	Aphelinidae	Aphelinus	sp.	KR795625.1
Hymenoptera	Chalcidoidea	Aphelinidae	Aphelinus	sp.	KR797526.1
Hymenoptera	Chalcidoidea	Aphelinidae	Aphelinus	sp.	KR797748.1
Hymenoptera	Chalcidoidea	Aphelinidae	Aphelinus	sp.	KR798017.1
Hymenoptera	Chalcidoidea	Aphelinidae	Aphelinus	sp.	KR798702.1
Hymenoptera	Chalcidoidea	Aphelinidae	Aphelinus	sp.	KR800433.1
Hymenoptera	Chalcidoidea	Aphelinidae	Aphelinus	sp.	KR800817.1
Hymenoptera	Chalcidoidea	Aphelinidae	Aphelinus	sp.	KR800918.1
Hymenoptera	Chalcidoidea	Aphelinidae	Aphelinus	sp.	KR802013.1
Hymenoptera	Chalcidoidea	Aphelinidae	Aphelinus	sp.	KR805812.1
Hymenoptera	Chalcidoidea	Aphelinidae	Aphelinus	sp.	KR807214.1
Hymenoptera	Chalcidoidea	Aphelinidae	Aphelinus	sp.	KR808775.1
Hymenoptera	Chalcidoidea	Aphelinidae	Aphelinus	sp.	KR874418.1
Hymenoptera	Chalcidoidea	Aphelinidae	Aphelinus	sp.	KR874515.1
Hymenoptera	Chalcidoidea	Aphelinidae	Aphelinus	sp.	KR875162.1
Hymenoptera	Chalcidoidea	Aphelinidae	Aphelinus	sp.	KR876157.1
Hymenoptera	Chalcidoidea	Aphelinidae	Aphelinus	sp.	KR877306.1
Hymenoptera	Chalcidoidea	Aphelinidae	Aphelinus	sp.	KR877500.1
Hymenoptera	Chalcidoidea	Aphelinidae	Aphelinus	sp.	KR881209.1
Hymenoptera	Chalcidoidea	Aphelinidae	Aphelinus	sp.	KR881824.1
Hymenoptera	Chalcidoidea	Aphelinidae	Aphelinus	sp.	KR882909.1
Hymenoptera	Chalcidoidea	Aphelinidae	Aphelinus	sp.	KR883100.1
Hymenoptera	Chalcidoidea	Aphelinidae	Aphelinus	sp.	KR884136.1
Hymenoptera	Chalcidoidea	Aphelinidae	Aphelinus	sp.	KR884189.1
Hymenoptera	Chalcidoidea	Aphelinidae	Aphelinus	sp.	KR884993.1
Hymenoptera	Chalcidoidea	Aphelinidae	Aphelinus	sp.	KR885252.1
Hymenoptera	Chalcidoidea	Aphelinidae	Aphelinus	sp.	KR887552.1
Hymenoptera	Chalcidoidea	Aphelinidae	Aphelinus	sp.	KR892503.1
Hymenoptera	Chalcidoidea	Aphelinidae	Aphelinus	sp.	KR893386.1
Hymenoptera	Chalcidoidea	Aphelinidae	Aphelinus	sp.	KR893802.1
Hymenoptera	Chalcidoidea	Aphelinidae	Aphelinus	sp.	KR894865.1
Hymenoptera	Chalcidoidea	Aphelinidae	Aphelinus	sp.	KR897309.1
Hymenoptera	Chalcidoidea	Aphelinidae	Aphelinus	sp.	KR897484.1
Hymenoptera	Chalcidoidea	Aphelinidae	Aphelinus	sp.	KY833035.1
Hymenoptera	Chalcidoidea	Aphelinidae	Aphelinus	sp.	KY837599.1
Hymenoptera	Chalcidoidea	Aphelinidae	Aphelinus	sp.	KY838213.1
Hymenoptera	Chalcidoidea	Aphelinidae	Aphelinus	sp.	KY838692.1
Hymenoptera	Chalcidoidea	Aphelinidae	Aphelinus	sp.	KY839119.1
Hymenoptera	Chalcidoidea	Aphelinidae	Aphelinus	sp.	KY846984.1
Hymenoptera	Chalcidoidea	Aphelinidae	Aphelinus	sp.	MF932949.1

Hymenoptera	Chalcidoidea	Aphelinidae	Aphelinus	sp.	MF935563.1
Hymenoptera	Chalcidoidea	Aphelinidae	Aphelinus	sp.	MG438726.1
Hymenoptera	Chalcidoidea	Aphelinidae	Aphelinus	sp.	MG439361.1
Hymenoptera	Chalcidoidea	Aphelinidae	Aphelinus	sp.	MG439646.1
Hymenoptera	Chalcidoidea	Aphelinidae	Aphelinus	sp.	MG439786.1
Hymenoptera	Chalcidoidea	Aphelinidae	Aphelinus	sp.	MG439828.2
Hymenoptera	Chalcidoidea	Aphelinidae	Aphelinus	sp.	MG440306.2
Hymenoptera	Chalcidoidea	Aphelinidae	Aphelinus	sp.	MG441227.1
Hymenoptera	Chalcidoidea	Aphelinidae	Aphelinus	sp.	MG441268.1
Hymenoptera	Chalcidoidea	Aphelinidae	Aphelinus	sp.	MG441446.1
Hymenoptera	Chalcidoidea	Aphelinidae	Aphelinus	sp.	MG441698.1
Hymenoptera	Chalcidoidea	Aphelinidae	Aphelinus	sp.	MG442032.1
Hymenoptera	Chalcidoidea	Aphelinidae	Aphelinus	sp.	MG442849.1
Hymenoptera	Chalcidoidea	Aphelinidae	Aphelinus	sp.	MG442865.1
Hymenoptera	Chalcidoidea	Aphelinidae	Aphelinus	sp.	MG442898.1
Hymenoptera	Chalcidoidea	Aphelinidae	Aphelinus	sp.	MG443211.1
Hymenoptera	Chalcidoidea	Aphelinidae	Aphelinus	sp.	MG443221.1
Hymenoptera	Chalcidoidea	Aphelinidae	Aphelinus	sp.	MG443314.1
Hymenoptera	Chalcidoidea	Aphelinidae	Aphelinus	sp.	MG443557.1
Hymenoptera	Chalcidoidea	Aphelinidae	Aphelinus	sp.	MG443822.1
Hymenoptera	Chalcidoidea	Aphelinidae	Aphelinus	sp.	MG443830.1
Hymenoptera	Chalcidoidea	Aphelinidae	Aphelinus	sp.	MG444049.1
Hymenoptera	Chalcidoidea	Aphelinidae	Aphelinus	sp.	MG444098.1
Hymenoptera	Chalcidoidea	Aphelinidae	Aphelinus	sp.	MG444173.1
Hymenoptera	Chalcidoidea	Aphelinidae	Aphelinus	sp.	MG444174.1
Hymenoptera	Chalcidoidea	Aphelinidae	Aphelinus	sp.	MG444705.1
Hymenoptera	Chalcidoidea	Aphelinidae	Aphelinus	sp.	MG445235.1
Hymenoptera	Chalcidoidea	Aphelinidae	Aphelinus	sp.	MG445410.1
Hymenoptera	Chalcidoidea	Aphelinidae	Aphelinus	sp.	MG445527.1
Hymenoptera	Chalcidoidea	Aphelinidae	Aphelinus	sp.	MG445544.1
Hymenoptera	Chalcidoidea	Aphelinidae	Aphelinus	sp.	MG445753.1
Hymenoptera	Chalcidoidea	Aphelinidae	Aphelinus	sp.	MG445864.1
Hymenoptera	Chalcidoidea	Aphelinidae	Aphelinus	sp.	MG445994.1
Hymenoptera	Chalcidoidea	Aphelinidae	Aphelinus	sp.	MG446469.1
Hymenoptera	Chalcidoidea	Aphelinidae	Aphelinus	sp.	MG447244.1
Hymenoptera	Chalcidoidea	Aphelinidae	Aphelinus	sp.	MG447428.1
Hymenoptera	Chalcidoidea	Aphelinidae	Aphelinus	sp.	MG447592.1
Hymenoptera	Chalcidoidea	Aphelinidae	Aphelinus	sp.	MG447692.1
Hymenoptera	Chalcidoidea	Aphelinidae	Aphelinus	sp.	MG447809.1
Hymenoptera	Chalcidoidea	Aphelinidae	Aphelinus	sp.	MG448018.1
Hymenoptera	Chalcidoidea	Aphelinidae	Aphelinus	sp.	MG448049.1
Hymenoptera	Chalcidoidea	Aphelinidae	Aphelinus	sp.	MG448358.1
Hymenoptera	Chalcidoidea	Aphelinidae	Aphelinus	sp.	MG448461.1
Hymenoptera	Chalcidoidea	Aphelinidae	Aphelinus	sp.	MG448532.1
Hymenoptera	Chalcidoidea	Aphelinidae	Aphelinus	sp.	MK308513.1
Hymenoptera	Chalcidoidea	Aphelinidae	Aphelinus	varipes	HQ599571.1
Hymenoptera	Chalcidoidea	Aphelinidae	Aphelinus	varipes	JX507449.1
Hymenoptera	Chalcidoidea	Aphelinidae	Aphelinus	varipes	KJ085820.1
Hymenoptera	Chalcidoidea	Aphelinidae	Aphelinus	varipes	KJ086033.1
Hymenoptera	Chalcidoidea	Aphelinidae	Aphelinus	varipes	KR798288.1
Hymenoptera	Chalcidoidea	Aphelinidae	Aphelinus	varipes	KR798300.1
Hymenoptera	Chalcidoidea	Aphelinidae	Aphelinus	varipes	KR802899.1
Hymenoptera	Chalcidoidea	Aphelinidae	Aphelinus	varipes	KR803574.1
Hymenoptera	Chalcidoidea	Aphelinidae	Aphelinus	varipes	KR804034.1

Hymenoptera	Chalcidoidea	Aphelinidae	Aphelinus	varipes	KR809185.1
Hymenoptera	Chalcidoidea	Aphelinidae	Aphelinus	varipes	KY912639.1
Hymenoptera	Chalcidoidea	Aphelinidae	Aphelinus	varipes	LC260604.1
Hymenoptera	Chalcidoidea	Aphelinidae	Aphelinus	varipes	LC260605.1
Hymenoptera	Chalcidoidea	Aphelinidae	Aphelinus	varipes	MF807203.1
Hymenoptera	Chalcidoidea	Aphelinidae	Aphelinus	varipes	MF850274.1
Hymenoptera	Chalcidoidea	Aphelinidae	Aphytis	aonidiae	JQ268915.1
Hymenoptera	Chalcidoidea	Aphelinidae	Aphytis	chilensis	MH456432.1
Hymenoptera	Chalcidoidea	Aphelinidae	Aphytis	chrysomphali	JQ083695.1
Hymenoptera	Chalcidoidea	Aphelinidae	Aphytis	chrysomphali	JQ083696.1
Hymenoptera	Chalcidoidea	Aphelinidae	Aphytis	chrysomphali	JQ083697.1
Hymenoptera	Chalcidoidea	Aphelinidae	Aphytis	chrysomphali	JQ083698.1
Hymenoptera	Chalcidoidea	Aphelinidae	Aphytis	chrysomphali	JQ083699.1
Hymenoptera	Chalcidoidea	Aphelinidae	Aphytis	chrysomphali	JQ083700.1
Hymenoptera	Chalcidoidea	Aphelinidae	Aphytis	hispanicus	JQ268913.1
Hymenoptera	Chalcidoidea	Aphelinidae	Aphytis	lepidosaphes	MH456646.1
Hymenoptera	Chalcidoidea	Aphelinidae	Aphytis	lingnanensis	JQ083705.1
Hymenoptera	Chalcidoidea	Aphelinidae	Aphytis	lingnanensis	JQ083706.1
Hymenoptera	Chalcidoidea	Aphelinidae	Aphytis	lingnanensis	JQ083707.1
Hymenoptera	Chalcidoidea	Aphelinidae	Aphytis	lingnanensis	JQ083708.1
Hymenoptera	Chalcidoidea	Aphelinidae	Aphytis	lingnanensis	JQ083709.1
Hymenoptera	Chalcidoidea	Aphelinidae	Aphytis	melinus	JQ083669.1
Hymenoptera	Chalcidoidea	Aphelinidae	Aphytis	melinus	JQ083670.1
Hymenoptera	Chalcidoidea	Aphelinidae	Aphytis	melinus	JQ083671.1
Hymenoptera	Chalcidoidea	Aphelinidae	Aphytis	melinus	JQ083672.1
Hymenoptera	Chalcidoidea	Aphelinidae	Aphytis	melinus	JQ083673.1
Hymenoptera	Chalcidoidea	Aphelinidae	Aphytis	melinus	JQ268916.1
Hymenoptera	Chalcidoidea	Aphelinidae	Aphytis	melinus	JQ268917.1
Hymenoptera	Chalcidoidea	Aphelinidae	Aphytis	melinus	MH456559.1
Hymenoptera	Chalcidoidea	Aphelinidae	Aphytis	sp.	JQ083675.1
Hymenoptera	Chalcidoidea	Aphelinidae	Aphytis	sp.	JQ083676.1
Hymenoptera	Chalcidoidea	Aphelinidae	Aphytis	sp.	JQ083677.1
Hymenoptera	Chalcidoidea	Aphelinidae	Aphytis	sp.	JQ083679.1
Hymenoptera	Chalcidoidea	Aphelinidae	Aphytis	sp.	JQ083681.1
Hymenoptera	Chalcidoidea	Aphelinidae	Aphytis	sp.	JQ083682.1
Hymenoptera	Chalcidoidea	Aphelinidae	Aphytis	sp.	JQ083683.1
Hymenoptera	Chalcidoidea	Aphelinidae	Aphytis	sp.	JQ083686.1
Hymenoptera	Chalcidoidea	Aphelinidae	Aphytis	sp.	JQ083687.1
Hymenoptera	Chalcidoidea	Aphelinidae	Aphytis	sp.	JQ083688.1
Hymenoptera	Chalcidoidea	Aphelinidae	Aphytis	sp.	JQ083689.1
Hymenoptera	Chalcidoidea	Aphelinidae	Aphytis	sp.	JQ083690.1
Hymenoptera	Chalcidoidea	Aphelinidae	Aphytis	sp.	JQ083691.1
Hymenoptera	Chalcidoidea	Aphelinidae	Aphytis	sp.	JQ083693.1
Hymenoptera	Chalcidoidea	Aphelinidae	Aphytis	sp.	JQ083694.1
Hymenoptera	Chalcidoidea	Aphelinidae	Aphytis	sp.	JQ083701.1
Hymenoptera	Chalcidoidea	Aphelinidae	Aphytis	sp.	JQ083702.1
Hymenoptera	Chalcidoidea	Aphelinidae	Aphytis	sp.	KR406743.1
Hymenoptera	Chalcidoidea	Aphelinidae	Aphytis	sp.	KR409222.1
Hymenoptera	Chalcidoidea	Aphelinidae	Aphytis	sp.	KR414997.1
Hymenoptera	Chalcidoidea	Aphelinidae	Aphytis	sp.	KR782808.1
Hymenoptera	Chalcidoidea	Aphelinidae	Aphytis	sp.	KR786441.1
Hymenoptera	Chalcidoidea	Aphelinidae	Aphytis	sp.	KR790133.1
Hymenoptera	Chalcidoidea	Aphelinidae	Aphytis	sp.	KR791905.1
Hymenoptera	Chalcidoidea	Aphelinidae	Aphytis	sp.	KR792362.1

Hymenoptera	Chalcidoidea	Aphelinidae	Aphytis	sp.	KR792462.1
Hymenoptera	Chalcidoidea	Aphelinidae	Aphytis	sp.	KR797109.1
Hymenoptera	Chalcidoidea	Aphelinidae	Aphytis	sp.	KR798121.1
Hymenoptera	Chalcidoidea	Aphelinidae	Aphytis	sp.	KR801403.1
Hymenoptera	Chalcidoidea	Aphelinidae	Aphytis	sp.	KR801768.1
Hymenoptera	Chalcidoidea	Aphelinidae	Aphytis	sp.	KR803244.1
Hymenoptera	Chalcidoidea	Aphelinidae	Aphytis	sp.	KR805312.1
Hymenoptera	Chalcidoidea	Aphelinidae	Aphytis	sp.	KR806685.1
Hymenoptera	Chalcidoidea	Aphelinidae	Aphytis	sp.	KR808517.1
Hymenoptera	Chalcidoidea	Aphelinidae	Aphytis	sp.	KR875933.1
Hymenoptera	Chalcidoidea	Aphelinidae	Aphytis	sp.	KR876393.1
Hymenoptera	Chalcidoidea	Aphelinidae	Aphytis	sp.	KR878885.1
Hymenoptera	Chalcidoidea	Aphelinidae	Aphytis	sp.	KR882769.1
Hymenoptera	Chalcidoidea	Aphelinidae	Aphytis	sp.	KR887611.1
Hymenoptera	Chalcidoidea	Aphelinidae	Aphytis	sp.	KR897424.1
Hymenoptera	Chalcidoidea	Aphelinidae	Aphytis	sp.	KR897876.1
Hymenoptera	Chalcidoidea	Aphelinidae	Aphytis	sp.	MF933428.1
Hymenoptera	Chalcidoidea	Aphelinidae	Aphytis	sp.	MF934228.1
Hymenoptera	Chalcidoidea	Aphelinidae	Aphytis	sp.	MF934722.1
Hymenoptera	Chalcidoidea	Aphelinidae	Aphytis	sp.	MF935837.1
Hymenoptera	Chalcidoidea	Aphelinidae	Aphytis	sp.	MF937154.1
Hymenoptera	Chalcidoidea	Aphelinidae	Aphytis	sp.	MF937983.1
Hymenoptera	Chalcidoidea	Aphelinidae	Aphytis	sp.	MG444709.1
Hymenoptera	Chalcidoidea	Aphelinidae	Aphytis	sp.	MG445224.1
Hymenoptera	Chalcidoidea	Aphelinidae	Aphytis	sp.	MG445230.1
Hymenoptera	Chalcidoidea	Aphelinidae	Aphytis	sp.	MG445403.1
Hymenoptera	Chalcidoidea	Aphelinidae	Aphytis	sp.	MG445641.1
Hymenoptera	Chalcidoidea	Aphelinidae	Aphytis	sp.	MH456575.1
Hymenoptera	Chalcidoidea	Aphelinidae	Aphytis	sp.	MH456581.1
Hymenoptera	Chalcidoidea	Aphelinidae	Aphytis	sp.	MH456713.1
Hymenoptera	Chalcidoidea	Aphelinidae	Aphytis	sp.	MH456752.1
Hymenoptera	Chalcidoidea	Aphelinidae	Aphytis	sp.	MH456764.1
Hymenoptera	Chalcidoidea	Aphelinidae	Centrodora	sp.	KR783602.1
Hymenoptera	Chalcidoidea	Aphelinidae	Centrodora	sp.	KR783845.1
Hymenoptera	Chalcidoidea	Aphelinidae	Centrodora	sp.	KR784613.1
Hymenoptera	Chalcidoidea	Aphelinidae	Centrodora	sp.	KR784985.1
Hymenoptera	Chalcidoidea	Aphelinidae	Centrodora	sp.	KR785565.1
Hymenoptera	Chalcidoidea	Aphelinidae	Centrodora	sp.	KR787220.1
Hymenoptera	Chalcidoidea	Aphelinidae	Centrodora	sp.	KR787235.1
Hymenoptera	Chalcidoidea	Aphelinidae	Centrodora	sp.	KR788646.1
Hymenoptera	Chalcidoidea	Aphelinidae	Centrodora	sp.	KR788933.1
Hymenoptera	Chalcidoidea	Aphelinidae	Centrodora	sp.	KR789552.1
Hymenoptera	Chalcidoidea	Aphelinidae	Centrodora	sp.	KR790229.1
Hymenoptera	Chalcidoidea	Aphelinidae	Centrodora	sp.	KR790682.1
Hymenoptera	Chalcidoidea	Aphelinidae	Centrodora	sp.	KR790783.1
Hymenoptera	Chalcidoidea	Aphelinidae	Centrodora	sp.	KR792750.1
Hymenoptera	Chalcidoidea	Aphelinidae	Centrodora	sp.	KR796435.1
Hymenoptera	Chalcidoidea	Aphelinidae	Centrodora	sp.	KR797324.1
Hymenoptera	Chalcidoidea	Aphelinidae	Centrodora	sp.	KR798862.1
Hymenoptera	Chalcidoidea	Aphelinidae	Centrodora	sp.	KR799545.1
Hymenoptera	Chalcidoidea	Aphelinidae	Centrodora	sp.	KR799959.1
Hymenoptera	Chalcidoidea	Aphelinidae	Centrodora	sp.	KR800085.1
Hymenoptera	Chalcidoidea	Aphelinidae	Centrodora	sp.	KR800336.1
Hymenoptera	Chalcidoidea	Aphelinidae	Centrodora	sp.	KR800873.1

Hymenoptera	Chalcidoidea	Aphelinidae	Centrodora	sp.	KR804722.1
Hymenoptera	Chalcidoidea	Aphelinidae	Centrodora	sp.	KR804916.1
Hymenoptera	Chalcidoidea	Aphelinidae	Centrodora	sp.	KR805915.1
Hymenoptera	Chalcidoidea	Aphelinidae	Centrodora	sp.	KR806195.1
Hymenoptera	Chalcidoidea	Aphelinidae	Centrodora	sp.	KR806683.1
Hymenoptera	Chalcidoidea	Aphelinidae	Centrodora	sp.	KR808784.1
Hymenoptera	Chalcidoidea	Aphelinidae	Centrodora	sp.	KR809223.1
Hymenoptera	Chalcidoidea	Aphelinidae	Centrodora	sp.	KR880123.1
Hymenoptera	Chalcidoidea	Aphelinidae	Centrodora	sp.	KR890613.1
Hymenoptera	Chalcidoidea	Aphelinidae	Centrodora	sp.	KR890981.1
Hymenoptera	Chalcidoidea	Aphelinidae	Centrodora	sp.	KR891346.1
Hymenoptera	Chalcidoidea	Aphelinidae	Centrodora	sp.	KR897614.1
Hymenoptera	Chalcidoidea	Aphelinidae	Centrodora	sp.	MF931233.1
Hymenoptera	Chalcidoidea	Aphelinidae	Centrodora	sp.	MG439813.2
Hymenoptera	Chalcidoidea	Aphelinidae	Centrodora	sp.	MG443247.1
Hymenoptera	Chalcidoidea	Aphelinidae	Centrodora	sp.	MG444020.1
Hymenoptera	Chalcidoidea	Aphelinidae	Centrodora	sp.	MG444290.1
Hymenoptera	Chalcidoidea	Aphelinidae	Centrodora	sp.	MG445637.1
Hymenoptera	Chalcidoidea	Aphelinidae	Centrodora	sp.	MG446046.1
Hymenoptera	Chalcidoidea	Aphelinidae	Centrodora	sp.	MG446322.1
Hymenoptera	Chalcidoidea	Aphelinidae	Centrodora	sp.	MG447445.1
Hymenoptera	Chalcidoidea	Aphelinidae	Centrodora	sp.	MG448294.1
Hymenoptera	Chalcidoidea	Aphelinidae	Coccophaginae	sp.	MH456744.1
Hymenoptera	Chalcidoidea	Aphelinidae	Coccophaginae	sp.	MH456756.1
Hymenoptera	Chalcidoidea	Aphelinidae	Coccophagus	gurneyi	MH456688.1
Hymenoptera	Chalcidoidea	Aphelinidae	Coccophagus	lycimnia	FM210137.1
Hymenoptera	Chalcidoidea	Aphelinidae	Coccophagus	sp.	KF938924.1
Hymenoptera	Chalcidoidea	Aphelinidae	Coccophagus	yoshidae	MH456402.1
Hymenoptera	Chalcidoidea	Aphelinidae	Coccophagus	yoshidae	MH456480.1
Hymenoptera	Chalcidoidea	Aphelinidae	Coccophagus	yoshidae	MH456609.1
Hymenoptera	Chalcidoidea	Aphelinidae	Coccophagus	yoshidae	MH456610.1
Hymenoptera	Chalcidoidea	Aphelinidae	Encarsia	berlesei	KT884744.1
Hymenoptera	Chalcidoidea	Aphelinidae	Encarsia	citrina	MH928406.1
Hymenoptera	Chalcidoidea	Aphelinidae	Encarsia	citrina	MH928635.1
Hymenoptera	Chalcidoidea	Aphelinidae	Encarsia	citrina	MH928822.1
Hymenoptera	Chalcidoidea	Aphelinidae	Encarsia	gracilens	MH928979.1
Hymenoptera	Chalcidoidea	Aphelinidae	Encarsia	hispida	MH456571.1
Hymenoptera	Chalcidoidea	Aphelinidae	Encarsia	inaron	KF055389.1
Hymenoptera	Chalcidoidea	Aphelinidae	Encarsia	inaron	KF055390.1
Hymenoptera	Chalcidoidea	Aphelinidae	Encarsia	inaron	KF055391.1
Hymenoptera	Chalcidoidea	Aphelinidae	Encarsia	inaron	KF055394.1
Hymenoptera	Chalcidoidea	Aphelinidae	Encarsia	inaron	KY839153.1
Hymenoptera	Chalcidoidea	Aphelinidae	Encarsia	inquirenda	JQ268914.1
Hymenoptera	Chalcidoidea	Aphelinidae	Encarsia	iris	HQ660515.1
Hymenoptera	Chalcidoidea	Aphelinidae	Encarsia	iris	JF750716.1
Hymenoptera	Chalcidoidea	Aphelinidae	Encarsia	iris	JF750717.1
Hymenoptera	Chalcidoidea	Aphelinidae	Encarsia	iris	JF750718.1
Hymenoptera	Chalcidoidea	Aphelinidae	Encarsia	iris	JF750719.1
Hymenoptera	Chalcidoidea	Aphelinidae	Encarsia	lounsburyi	MH456759.1
Hymenoptera	Chalcidoidea	Aphelinidae	Encarsia	perniciosi	JQ083711.1
Hymenoptera	Chalcidoidea	Aphelinidae	Encarsia	perniciosi	JQ083712.1
Hymenoptera	Chalcidoidea	Aphelinidae	Encarsia	perniciosi	JQ083713.1
Hymenoptera	Chalcidoidea	Aphelinidae	Encarsia	perniciosi	JQ083714.1
Hymenoptera	Chalcidoidea	Aphelinidae	Encarsia	perniciosi	JQ083715.1

Hymenoptera	Chalcidoidea	Aphelinidae	Encarsia	perniciosi	JQ083716.1
Hymenoptera	Chalcidoidea	Aphelinidae	Encarsia	perniciosi	JQ083717.1
Hymenoptera	Chalcidoidea	Aphelinidae	Encarsia	sp.	KJ087645.1
Hymenoptera	Chalcidoidea	Aphelinidae	Encarsia	sp.	KJ444561.1
Hymenoptera	Chalcidoidea	Aphelinidae	Encarsia	sp.	KR784483.1
Hymenoptera	Chalcidoidea	Aphelinidae	Encarsia	sp.	KR789837.1
Hymenoptera	Chalcidoidea	Aphelinidae	Encarsia	sp.	KR790340.1
Hymenoptera	Chalcidoidea	Aphelinidae	Encarsia	sp.	KR791785.1
Hymenoptera	Chalcidoidea	Aphelinidae	Encarsia	sp.	KR792837.1
Hymenoptera	Chalcidoidea	Aphelinidae	Encarsia	sp.	KR795157.1
Hymenoptera	Chalcidoidea	Aphelinidae	Encarsia	sp.	KR795684.1
Hymenoptera	Chalcidoidea	Aphelinidae	Encarsia	sp.	KR799927.1
Hymenoptera	Chalcidoidea	Aphelinidae	Encarsia	sp.	KR801325.1
Hymenoptera	Chalcidoidea	Aphelinidae	Encarsia	sp.	KR802217.1
Hymenoptera	Chalcidoidea	Aphelinidae	Encarsia	sp.	KR893683.1
Hymenoptera	Chalcidoidea	Aphelinidae	Encarsia	sp.	KR896473.1
Hymenoptera	Chalcidoidea	Aphelinidae	Encarsia	sp.	KR926578.1
Hymenoptera	Chalcidoidea	Aphelinidae	Encarsia	sp.	KY846424.1
Hymenoptera	Chalcidoidea	Aphelinidae	Encarsia	sp.	MF807205.1
Hymenoptera	Chalcidoidea	Aphelinidae	Encarsia	sp.	MF932284.1
Hymenoptera	Chalcidoidea	Aphelinidae	Encarsia	sp.	MG444775.1
Hymenoptera	Chalcidoidea	Aphelinidae	Encarsia	sp.	MG444828.1
Hymenoptera	Chalcidoidea	Aphelinidae	Encarsia	sp.	MH456783.1
Hymenoptera	Chalcidoidea	Aphelinidae	Encarsia	sp.	MH927031.1
Hymenoptera	Chalcidoidea	Aphelinidae	Encarsia	sp.	MH929027.1
Hymenoptera	Chalcidoidea	Aphelinidae	Eretmocerus	orchamoplati	HQ660514.1
Hymenoptera	Chalcidoidea	Aphelinidae	Eretmocerus	orchamoplati	JF750711.1
Hymenoptera	Chalcidoidea	Aphelinidae	Eretmocerus	orchamoplati	JF750712.1
Hymenoptera	Chalcidoidea	Aphelinidae	Eretmocerus	orchamoplati	JF750713.1
Hymenoptera	Chalcidoidea	Aphelinidae	Eretmocerus	orchamoplati	JF750714.1
Hymenoptera	Chalcidoidea	Aphelinidae	Eretmocerus	orchamoplati	JF750715.1
Hymenoptera	Chalcidoidea	Aphelinidae	Marietta	caridei	MH456576.1
Hymenoptera	Chalcidoidea	Aphelinidae	UNVERIFIED	Aphelinidae	KJ207939.1
Hymenoptera	Chalcidoidea	Aphelinidae	UNVERIFIED	Aphelinidae	MG439866.1
Hymenoptera	Chalcidoidea	Aphelinidae	UNVERIFIED	Aphelinidae	MG440061.1
Hymenoptera	Chalcidoidea	Aphelinidae	UNVERIFIED	Aphelinidae	MG440371.1
Hymenoptera	Chalcidoidea	Aphelinidae	UNVERIFIED	Aphelinidae	MG440754.1
Hymenoptera	Chalcidoidea	Aphelinidae	UNVERIFIED	Aphelinidae	MG440971.1
Hymenoptera	Chalcidoidea	Aphelinidae	UNVERIFIED	Aphelinus	MG439820.1
Hymenoptera	Chalcidoidea	Aphelinidae	UNVERIFIED	Aphelinus	MG440333.1
Hymenoptera	Chalcidoidea	Aphelinidae	UNVERIFIED	Aphelinus	MG440418.1
Hymenoptera	Chalcidoidea	Aphelinidae	UNVERIFIED	Aphelinus	MG440435.1
Hymenoptera	Chalcidoidea	Aphelinidae	UNVERIFIED	Aphelinus	MG440714.1
Hymenoptera	Chalcidoidea	Aphelinidae	UNVERIFIED	Aphelinus	MG440953.1
Hymenoptera	Chalcidoidea	Aphelinidae	UNVERIFIED	Aphelinus	MG444544.1
Hymenoptera	Chalcidoidea	Aphelinidae	Wallaceaphytis	kikiae	KF718962.1
Hymenoptera	Chalcidoidea	Azotidae	Ablerus	perspeciosus	JQ268912.1
Hymenoptera	Chalcidoidea	Azotidae	Azotidae	sp.	KY830487.1
Hymenoptera	Chalcidoidea	Azotidae	Azotidae	sp.	KY831862.1
Hymenoptera	Chalcidoidea	Azotidae	Azotidae	sp.	KY834754.1
Hymenoptera	Chalcidoidea	Azotidae	Azotidae	sp.	KY835481.1
Hymenoptera	Chalcidoidea	Azotidae	Azotidae	sp.	KY836393.1
Hymenoptera	Chalcidoidea	Azotidae	Azotidae	sp.	KY838343.1
Hymenoptera	Chalcidoidea	Azotidae	Azotidae	sp.	KY839766.1

Hymenoptera	Chalcidoidea	Azotidae	Azotidae	sp.	KY841081.1
Hymenoptera	Chalcidoidea	Azotidae	Azotidae	sp.	KY842201.1
Hymenoptera	Chalcidoidea	Azotidae	Azotidae	sp.	KY843072.1
Hymenoptera	Chalcidoidea	Azotidae	Azotidae	sp.	KY843900.1
Hymenoptera	Chalcidoidea	Azotidae	Azotidae	sp.	KY845754.1
Hymenoptera	Chalcidoidea	Chalcididae	Brachymeria	femorata	KT175580.1
Hymenoptera	Chalcidoidea	Chalcididae	Brachymeria	sp.	MK202111.1
Hymenoptera	Chalcidoidea	Chalcididae	Chalcididae	sp.	HQ929948.1
Hymenoptera	Chalcidoidea	Chalcididae	Chalcididae	sp.	HQ930200.1
Hymenoptera	Chalcidoidea	Chalcididae	Chalcididae	sp.	HQ930233.1
Hymenoptera	Chalcidoidea	Chalcididae	Chalcididae	sp.	KJ087360.1
Hymenoptera	Chalcidoidea	Chalcididae	Chalcididae	sp.	KJ444660.1
Hymenoptera	Chalcidoidea	Chalcididae	Chalcididae	sp.	KJ637473.1
Hymenoptera	Chalcidoidea	Chalcididae	Chalcididae	sp.	KY830097.1
Hymenoptera	Chalcidoidea	Chalcididae	Chalcididae	sp.	KY834201.1
Hymenoptera	Chalcidoidea	Chalcididae	Chalcididae	sp.	KY834474.1
Hymenoptera	Chalcidoidea	Chalcididae	Chalcididae	sp.	KY845095.1
Hymenoptera	Chalcidoidea	Chalcididae	Chalcididae	sp.	MF899919.1
Hymenoptera	Chalcidoidea	Chalcididae	Chalcididae	sp.	MG499448.1
Hymenoptera	Chalcidoidea	Chalcididae	Chalcididae	sp.	OL694545.1
Hymenoptera	Chalcidoidea	Chalcididae	Chalcididae	sp.	OL694570.1
Hymenoptera	Chalcidoidea	Chalcididae	Conura	albifrons	HM883271.1
Hymenoptera	Chalcidoidea	Chalcididae	Conura	albifrons	HQ106978.1
Hymenoptera	Chalcidoidea	Chalcididae	Conura	albifrons	JN293250.1
Hymenoptera	Chalcidoidea	Chalcididae	Conura	albifrons	JN294284.1
Hymenoptera	Chalcidoidea	Chalcididae	Conura	albifrons	KF444813.1
Hymenoptera	Chalcidoidea	Chalcididae	Conura	albifrons	KJ208154.1
Hymenoptera	Chalcidoidea	Chalcididae	Conura	albifrons	KT708114.1
Hymenoptera	Chalcidoidea	Chalcididae	Conura	albifrons	KU874695.1
Hymenoptera	Chalcidoidea	Chalcididae	Conura	albifrons	MF908363.1
Hymenoptera	Chalcidoidea	Chalcididae	Conura	albifrons	MG441840.1
Hymenoptera	Chalcidoidea	Chalcididae	Conura	albifrons	MG442100.1
Hymenoptera	Chalcidoidea	Chalcididae	Conura	albifrons	MG444274.1
Hymenoptera	Chalcidoidea	Chalcididae	Conura	albifrons	MG444980.1
Hymenoptera	Chalcidoidea	Chalcididae	Conura	albifrons	MG445282.1
Hymenoptera	Chalcidoidea	Chalcididae	Conura	albifrons	MG445288.1
Hymenoptera	Chalcidoidea	Chalcididae	Conura	albifrons	MG445961.1
Hymenoptera	Chalcidoidea	Chalcididae	Conura	sp.	MK202025.1
Hymenoptera	Chalcidoidea	Chalcididae	Conura	sp.	MK202029.1
Hymenoptera	Chalcidoidea	Chalcididae	Conura	sp.	MK202035.1
Hymenoptera	Chalcidoidea	Chalcididae	Conura	sp.	MK202036.1
Hymenoptera	Chalcidoidea	Chalcididae	Conura	sp.	MK202044.1
Hymenoptera	Chalcidoidea	Chalcididae	Conura	sp.	MK202045.1
Hymenoptera	Chalcidoidea	Chalcididae	Conura	sp.	MK202054.1
Hymenoptera	Chalcidoidea	Chalcididae	Conura	sp.	MK202055.1
Hymenoptera	Chalcidoidea	Chalcididae	Conura	sp.	MK202060.1
Hymenoptera	Chalcidoidea	Chalcididae	Conura	sp.	MK202064.1
Hymenoptera	Chalcidoidea	Chalcididae	Conura	sp.	MK202068.1
Hymenoptera	Chalcidoidea	Chalcididae	Conura	sp.	MK202073.1
Hymenoptera	Chalcidoidea	Chalcididae	Conura	sp.	MK202084.1
Hymenoptera	Chalcidoidea	Chalcididae	Conura	sp.	MK202091.1
Hymenoptera	Chalcidoidea	Chalcididae	Conura	sp.	MK202098.1
Hymenoptera	Chalcidoidea	Chalcididae	Conura	sp.	MK202106.1
Hymenoptera	Chalcidoidea	Chalcididae	Conura	sp.	MK202112.1

Hymenoptera	Chalcidoidea	Chalcididae	Conura	sp.	MK202116.1
Hymenoptera	Chalcidoidea	Chalcididae	Conura	sp.	MK202117.1
Hymenoptera	Chalcidoidea	Chalcididae	Conura	sp.	MK202121.1
Hymenoptera	Chalcidoidea	Chalcididae	Conura	sp.	MK202123.1
Hymenoptera	Chalcidoidea	Chalcididae	Conura	sp.	MK202127.1
Hymenoptera	Chalcidoidea	Chalcididae	Conura	sp.	MK202137.1
Hymenoptera	Chalcidoidea	Chalcididae	Conura	sp.	MK202140.1
Hymenoptera	Chalcidoidea	Chalcididae	Dirhinus	sp.	MK202023.1
Hymenoptera	Chalcidoidea	Chalcididae	Dirhinus	sp.	MK202063.1
Hymenoptera	Chalcidoidea	Chalcididae	Dirhinus	sp.	MK202069.1
Hymenoptera	Chalcidoidea	Chalcididae	Dirhinus	sp.	MK202104.1
Hymenoptera	Chalcidoidea	Chalcididae	Epitranus	sp.	KY837094.1
Hymenoptera	Chalcidoidea	Chalcididae	Haltichella	rufipes	JQ756606.1
Hymenoptera	Chalcidoidea	Chalcididae	Melanosmicra	acutodentata	MK202144.1
Hymenoptera	Chalcidoidea	Chalcididae	Melanosmicra	areta	MK202126.1
Hymenoptera	Chalcidoidea	Chalcididae	Melanosmicra	immaculata	MK202022.1
Hymenoptera	Chalcidoidea	Chalcididae	Melanosmicra	sp.	MK202058.1
Hymenoptera	Chalcidoidea	Chalcididae	Melanosmicra	sp.	MK202139.1
Hymenoptera	Chalcidoidea	Chalcididae	Melanosmicra	sp.	MK202147.1
Hymenoptera	Chalcidoidea	Chalcididae	Notaspidium	acutum	MK202032.1
Hymenoptera	Chalcidoidea	Chalcididae	Notaspidium	boharti	MK202092.1
Hymenoptera	Chalcidoidea	Chalcididae	Phasgonophora	sulcata	JF708281.1
Hymenoptera	Chalcidoidea	Chalcididae	Phasgonophora	sulcata	JF708282.1
Hymenoptera	Chalcidoidea	Chalcididae	Phasgonophora	sulcata	JF708285.1
Hymenoptera	Chalcidoidea	Chalcididae	Phasgonophora	sulcata	JF708286.1
Hymenoptera	Chalcidoidea	Chalcididae	Phasgonophora	sulcata	JF708287.1
Hymenoptera	Chalcidoidea	Chalcididae	UNVERIFIED	Conura	MG440302.1
Hymenoptera	Chalcidoidea	Chalcididae	UNVERIFIED	Conura	MG440511.1
Hymenoptera	Chalcidoidea	Chalcidoidea	Chalcidoidea	sp.	FN662393.1
Hymenoptera	Chalcidoidea	Encyrtidae	Aenasius	advena	KJ850498.1
Hymenoptera	Chalcidoidea	Encyrtidae	Aenasius	bambawalei	KY832864.1
Hymenoptera	Chalcidoidea	Encyrtidae	Aenasius	bambawalei	KY837980.1
Hymenoptera	Chalcidoidea	Encyrtidae	Aenasius	bambawalei	KY838887.1
Hymenoptera	Chalcidoidea	Encyrtidae	Aenasius	bambawalei	KY841566.1
Hymenoptera	Chalcidoidea	Encyrtidae	Aenasius	bambawalei	KY846843.1
Hymenoptera	Chalcidoidea	Encyrtidae	Ageniaspis	citricola	KF850108.1
Hymenoptera	Chalcidoidea	Encyrtidae	Ageniaspis	sp.	KF850121.1
Hymenoptera	Chalcidoidea	Encyrtidae	Anagyrus	cachamai	MG731504.1
Hymenoptera	Chalcidoidea	Encyrtidae	Anagyrus	cachamai	MG731505.1
Hymenoptera	Chalcidoidea	Encyrtidae	Anagyrus	cachamai	MG731506.1
Hymenoptera	Chalcidoidea	Encyrtidae	Anagyrus	cachamai	MG731518.1
Hymenoptera	Chalcidoidea	Encyrtidae	Anagyrus	quilmes	MG731507.1
Hymenoptera	Chalcidoidea	Encyrtidae	Anagyrus	quilmes	MG731510.1
Hymenoptera	Chalcidoidea	Encyrtidae	Anagyrus	quilmes	MG731512.1
Hymenoptera	Chalcidoidea	Encyrtidae	Anagyrus	quilmes	MG731515.1
Hymenoptera	Chalcidoidea	Encyrtidae	Anagyrus	quilmes	MG731516.1
Hymenoptera	Chalcidoidea	Encyrtidae	Anagyrus	quilmes	MG731520.1
Hymenoptera	Chalcidoidea	Encyrtidae	Anagyrus	quilmes	MG731521.1
Hymenoptera	Chalcidoidea	Encyrtidae	Anagyrus	quilmes	MG731522.1
Hymenoptera	Chalcidoidea	Encyrtidae	Anagyrus	quilmes	MG731523.1
Hymenoptera	Chalcidoidea	Encyrtidae	Anagyrus	quilmes	MG731524.1
Hymenoptera	Chalcidoidea	Encyrtidae	Astymachus	japonicus	MN698730.1
Hymenoptera	Chalcidoidea	Encyrtidae	Avetianella	longoi	JQ688066.1
Hymenoptera	Chalcidoidea	Encyrtidae	Avetianella	longoi	JQ688067.1

Hymenoptera	Chalcidoidea	Encyrtidae	Avetianella	longoi	JQ688068.1
Hymenoptera	Chalcidoidea	Encyrtidae	Blepyrus	insularis	KJ850500.1
Hymenoptera	Chalcidoidea	Encyrtidae	Bothriothorax	sp.	KR894744.1
Hymenoptera	Chalcidoidea	Encyrtidae	Boucekiella	depressa	MN698733.1
Hymenoptera	Chalcidoidea	Encyrtidae	Comperiella	bifasciata	JQ268910.1
Hymenoptera	Chalcidoidea	Encyrtidae	Comperiella	bifasciata	JQ268911.1
Hymenoptera	Chalcidoidea	Encyrtidae	Copidosoma	agrotis	KF850094.1
Hymenoptera	Chalcidoidea	Encyrtidae	Copidosoma	aretas	KF850130.1
Hymenoptera	Chalcidoidea	Encyrtidae	Copidosoma	boucheanum	KF850099.1
Hymenoptera	Chalcidoidea	Encyrtidae	Copidosoma	cervius	KF850097.1
Hymenoptera	Chalcidoidea	Encyrtidae	Copidosoma	chalconotum	KF850101.1
Hymenoptera	Chalcidoidea	Encyrtidae	Copidosoma	coimbatorense	KF850107.1
Hymenoptera	Chalcidoidea	Encyrtidae	Copidosoma	coimbatorense	KF850131.1
Hymenoptera	Chalcidoidea	Encyrtidae	Copidosoma	floridanum	KF850096.1
Hymenoptera	Chalcidoidea	Encyrtidae	Copidosoma	floridanum	KF850136.1
Hymenoptera	Chalcidoidea	Encyrtidae	Copidosoma	floridanum	KF850141.1
Hymenoptera	Chalcidoidea	Encyrtidae	Copidosoma	floridanum	KR406982.1
Hymenoptera	Chalcidoidea	Encyrtidae	Copidosoma	floridanum	KR414572.1
Hymenoptera	Chalcidoidea	Encyrtidae	Copidosoma	floridanum	KR420962.1
Hymenoptera	Chalcidoidea	Encyrtidae	Copidosoma	floridanum	KR782588.1
Hymenoptera	Chalcidoidea	Encyrtidae	Copidosoma	floridanum	KR785022.1
Hymenoptera	Chalcidoidea	Encyrtidae	Copidosoma	floridanum	KR786198.1
Hymenoptera	Chalcidoidea	Encyrtidae	Copidosoma	floridanum	KR787723.1
Hymenoptera	Chalcidoidea	Encyrtidae	Copidosoma	floridanum	KR787833.1
Hymenoptera	Chalcidoidea	Encyrtidae	Copidosoma	floridanum	KR789152.1
Hymenoptera	Chalcidoidea	Encyrtidae	Copidosoma	floridanum	KR789450.1
Hymenoptera	Chalcidoidea	Encyrtidae	Copidosoma	floridanum	KR789596.1
Hymenoptera	Chalcidoidea	Encyrtidae	Copidosoma	floridanum	KR792534.1
Hymenoptera	Chalcidoidea	Encyrtidae	Copidosoma	floridanum	KR793914.1
Hymenoptera	Chalcidoidea	Encyrtidae	Copidosoma	floridanum	KR794305.1
Hymenoptera	Chalcidoidea	Encyrtidae	Copidosoma	floridanum	KR795395.1
Hymenoptera	Chalcidoidea	Encyrtidae	Copidosoma	floridanum	KR799312.1
Hymenoptera	Chalcidoidea	Encyrtidae	Copidosoma	floridanum	KR800638.1
Hymenoptera	Chalcidoidea	Encyrtidae	Copidosoma	floridanum	KR804110.1
Hymenoptera	Chalcidoidea	Encyrtidae	Copidosoma	floridanum	KR804725.1
Hymenoptera	Chalcidoidea	Encyrtidae	Copidosoma	floridanum	KR805138.1
Hymenoptera	Chalcidoidea	Encyrtidae	Copidosoma	floridanum	KR805502.1
Hymenoptera	Chalcidoidea	Encyrtidae	Copidosoma	floridanum	KR806827.1
Hymenoptera	Chalcidoidea	Encyrtidae	Copidosoma	floridanum	KR807027.1
Hymenoptera	Chalcidoidea	Encyrtidae	Copidosoma	floridanum	KR808577.1
Hymenoptera	Chalcidoidea	Encyrtidae	Copidosoma	floridanum	KR875131.1
Hymenoptera	Chalcidoidea	Encyrtidae	Copidosoma	floridanum	KR876352.1
Hymenoptera	Chalcidoidea	Encyrtidae	Copidosoma	floridanum	KR876834.1
Hymenoptera	Chalcidoidea	Encyrtidae	Copidosoma	floridanum	KR877346.1
Hymenoptera	Chalcidoidea	Encyrtidae	Copidosoma	floridanum	KR881873.1
Hymenoptera	Chalcidoidea	Encyrtidae	Copidosoma	floridanum	KR882690.1
Hymenoptera	Chalcidoidea	Encyrtidae	Copidosoma	floridanum	KR887258.1
Hymenoptera	Chalcidoidea	Encyrtidae	Copidosoma	floridanum	KR890279.1
Hymenoptera	Chalcidoidea	Encyrtidae	Copidosoma	floridanum	KR893952.1
Hymenoptera	Chalcidoidea	Encyrtidae	Copidosoma	floridanum	KR897932.1
Hymenoptera	Chalcidoidea	Encyrtidae	Copidosoma	floridanum	KR900845.1
Hymenoptera	Chalcidoidea	Encyrtidae	Copidosoma	floridanum	KR902174.1
Hymenoptera	Chalcidoidea	Encyrtidae	Copidosoma	floridanum	KT610656.1
Hymenoptera	Chalcidoidea	Encyrtidae	Copidosoma	floridanum	MF898745.1

Hymenoptera	Chalcidoidea	Encyrtidae	Copidosoma	floridanum	MG444029.1
Hymenoptera	Chalcidoidea	Encyrtidae	Copidosoma	floridanum	MG444896.1
Hymenoptera	Chalcidoidea	Encyrtidae	Copidosoma	floridanum	MG445072.1
Hymenoptera	Chalcidoidea	Encyrtidae	Copidosoma	floridanum	MG445123.1
Hymenoptera	Chalcidoidea	Encyrtidae	Copidosoma	floridanum	MG445159.1
Hymenoptera	Chalcidoidea	Encyrtidae	Copidosoma	fuscisquama	KF850110.1
Hymenoptera	Chalcidoidea	Encyrtidae	Copidosoma	koehleri	KX443096.1
Hymenoptera	Chalcidoidea	Encyrtidae	Copidosoma	lucidum	KF850129.1
Hymenoptera	Chalcidoidea	Encyrtidae	Copidosoma	noyesi	KF850105.1
Hymenoptera	Chalcidoidea	Encyrtidae	Copidosoma	sp.	KF850109.1
Hymenoptera	Chalcidoidea	Encyrtidae	Copidosoma	sp.	KF850112.1
Hymenoptera	Chalcidoidea	Encyrtidae	Copidosoma	sp.	KF850117.1
Hymenoptera	Chalcidoidea	Encyrtidae	Copidosoma	sp.	KF850124.1
Hymenoptera	Chalcidoidea	Encyrtidae	Copidosoma	sp.	KF850125.1
Hymenoptera	Chalcidoidea	Encyrtidae	Copidosoma	sp.	KF850126.1
Hymenoptera	Chalcidoidea	Encyrtidae	Copidosoma	sp.	KF850128.1
Hymenoptera	Chalcidoidea	Encyrtidae	Copidosoma	sp.	KF850133.1
Hymenoptera	Chalcidoidea	Encyrtidae	Copidosoma	sp.	KF850134.1
Hymenoptera	Chalcidoidea	Encyrtidae	Copidosoma	sp.	KF850135.1
Hymenoptera	Chalcidoidea	Encyrtidae	Copidosoma	sp.	KF850137.1
Hymenoptera	Chalcidoidea	Encyrtidae	Copidosoma	peticus	KF850098.1
Hymenoptera	Chalcidoidea	Encyrtidae	Copidosoma	phaloniae	KF850100.1
Hymenoptera	Chalcidoidea	Encyrtidae	Copidosoma	primulum	KF850102.1
Hymenoptera	Chalcidoidea	Encyrtidae	Copidosoma	primulum	KY831867.1
Hymenoptera	Chalcidoidea	Encyrtidae	Copidosoma	primulum	KY837727.1
Hymenoptera	Chalcidoidea	Encyrtidae	Copidosoma	primulum	KY840018.1
Hymenoptera	Chalcidoidea	Encyrtidae	Copidosoma	primulum	MF673616.1
Hymenoptera	Chalcidoidea	Encyrtidae	Copidosoma	primulum	MF673617.1
Hymenoptera	Chalcidoidea	Encyrtidae	Copidosoma	serricorne	KF850116.1
Hymenoptera	Chalcidoidea	Encyrtidae	Copidosoma	sosares	KF850113.1
Hymenoptera	Chalcidoidea	Encyrtidae	Copidosoma	sp.	HQ106980.1
Hymenoptera	Chalcidoidea	Encyrtidae	Copidosoma	sp.	HQ106985.1
Hymenoptera	Chalcidoidea	Encyrtidae	Copidosoma	sp.	HQ106986.1
Hymenoptera	Chalcidoidea	Encyrtidae	Copidosoma	sp.	HQ106987.1
Hymenoptera	Chalcidoidea	Encyrtidae	Copidosoma	sp.	HQ106988.1
Hymenoptera	Chalcidoidea	Encyrtidae	Copidosoma	sp.	HQ106990.1
Hymenoptera	Chalcidoidea	Encyrtidae	Copidosoma	sp.	HQ106992.1
Hymenoptera	Chalcidoidea	Encyrtidae	Copidosoma	sp.	HQ106996.1
Hymenoptera	Chalcidoidea	Encyrtidae	Copidosoma	sp.	HQ106998.1
Hymenoptera	Chalcidoidea	Encyrtidae	Copidosoma	sp.	HQ107000.1
Hymenoptera	Chalcidoidea	Encyrtidae	Copidosoma	sp.	HQ107002.1
Hymenoptera	Chalcidoidea	Encyrtidae	Copidosoma	sp.	HQ107003.1
Hymenoptera	Chalcidoidea	Encyrtidae	Copidosoma	sp.	HQ107004.1
Hymenoptera	Chalcidoidea	Encyrtidae	Copidosoma	sp.	HQ107006.1
Hymenoptera	Chalcidoidea	Encyrtidae	Copidosoma	sp.	HQ107007.1
Hymenoptera	Chalcidoidea	Encyrtidae	Copidosoma	sp.	HQ107009.1
Hymenoptera	Chalcidoidea	Encyrtidae	Copidosoma	sp.	KR783521.1
Hymenoptera	Chalcidoidea	Encyrtidae	Copidosoma	sp.	KR788209.1
Hymenoptera	Chalcidoidea	Encyrtidae	Copidosoma	sp.	KR793794.1
Hymenoptera	Chalcidoidea	Encyrtidae	Copidosoma	sp.	KR797140.1
Hymenoptera	Chalcidoidea	Encyrtidae	Copidosoma	sp.	KR803644.1
Hymenoptera	Chalcidoidea	Encyrtidae	Copidosoma	sp.	KR803783.1
Hymenoptera	Chalcidoidea	Encyrtidae	Copidosoma	sp.	KR804761.1
Hymenoptera	Chalcidoidea	Encyrtidae	Copidosoma	sp.	KR877046.1

Hymenoptera	Chalcidoidea	Encyrtidae	Copidosoma	sp.	KR886957.1
Hymenoptera	Chalcidoidea	Encyrtidae	Copidosoma	sp.	KR897641.1
Hymenoptera	Chalcidoidea	Encyrtidae	Copidosoma	sp.	KR899311.1
Hymenoptera	Chalcidoidea	Encyrtidae	Copidosoma	sp.	KR901314.1
Hymenoptera	Chalcidoidea	Encyrtidae	Copidosoma	sp.	KX535016.1
Hymenoptera	Chalcidoidea	Encyrtidae	Copidosoma	sp.	MF898523.1
Hymenoptera	Chalcidoidea	Encyrtidae	Copidosoma	sp.	MF902116.1
Hymenoptera	Chalcidoidea	Encyrtidae	Copidosoma	sp.	MF906651.1
Hymenoptera	Chalcidoidea	Encyrtidae	Copidosoma	sp.	MF906707.1
Hymenoptera	Chalcidoidea	Encyrtidae	Copidosoma	sp.	MF907230.1
Hymenoptera	Chalcidoidea	Encyrtidae	Copidosoma	sp.	MG444508.1
Hymenoptera	Chalcidoidea	Encyrtidae	Copidosoma	thebe	KF850114.1
Hymenoptera	Chalcidoidea	Encyrtidae	Copidosoma	transversum	KF850120.1
Hymenoptera	Chalcidoidea	Encyrtidae	Copidosoma	truncatellum	KF850093.1
Hymenoptera	Chalcidoidea	Encyrtidae	Copidosoma	varicorne	KF850095.1
Hymenoptera	Chalcidoidea	Encyrtidae	Copidosomopsis	meridionalis	KF850103.1
Hymenoptera	Chalcidoidea	Encyrtidae	Copidosomopsis	nacoleiae	KF850118.1
Hymenoptera	Chalcidoidea	Encyrtidae	Encyrtidae	sp.	JQ576165.1
Hymenoptera	Chalcidoidea	Encyrtidae	Encyrtidae	sp.	HM374661.1
Hymenoptera	Chalcidoidea	Encyrtidae	Encyrtidae	sp.	HM374747.1
Hymenoptera	Chalcidoidea	Encyrtidae	Encyrtidae	sp.	HM414498.1
Hymenoptera	Chalcidoidea	Encyrtidae	Encyrtidae	sp.	HQ538465.1
Hymenoptera	Chalcidoidea	Encyrtidae	Encyrtidae	sp.	HQ929592.1
Hymenoptera	Chalcidoidea	Encyrtidae	Encyrtidae	sp.	HQ929605.1
Hymenoptera	Chalcidoidea	Encyrtidae	Encyrtidae	sp.	HQ929777.1
Hymenoptera	Chalcidoidea	Encyrtidae	Encyrtidae	sp.	JN292643.1
Hymenoptera	Chalcidoidea	Encyrtidae	Encyrtidae	sp.	JN292644.1
Hymenoptera	Chalcidoidea	Encyrtidae	Encyrtidae	sp.	JN293218.1
Hymenoptera	Chalcidoidea	Encyrtidae	Encyrtidae	sp.	JN293568.1
Hymenoptera	Chalcidoidea	Encyrtidae	Encyrtidae	sp.	JN293569.1
Hymenoptera	Chalcidoidea	Encyrtidae	Encyrtidae	sp.	KJ165488.1
Hymenoptera	Chalcidoidea	Encyrtidae	Encyrtidae	sp.	KR366072.1
Hymenoptera	Chalcidoidea	Encyrtidae	Encyrtidae	sp.	KR368966.1
Hymenoptera	Chalcidoidea	Encyrtidae	Encyrtidae	sp.	KR370502.1
Hymenoptera	Chalcidoidea	Encyrtidae	Encyrtidae	sp.	KR373861.1
Hymenoptera	Chalcidoidea	Encyrtidae	Encyrtidae	sp.	KR403891.1
Hymenoptera	Chalcidoidea	Encyrtidae	Encyrtidae	sp.	KR408607.1
Hymenoptera	Chalcidoidea	Encyrtidae	Encyrtidae	sp.	KR412263.1
Hymenoptera	Chalcidoidea	Encyrtidae	Encyrtidae	sp.	KR416014.1
Hymenoptera	Chalcidoidea	Encyrtidae	Encyrtidae	sp.	KR418866.1
Hymenoptera	Chalcidoidea	Encyrtidae	Encyrtidae	sp.	KR422052.1
Hymenoptera	Chalcidoidea	Encyrtidae	Encyrtidae	sp.	KR782494.1
Hymenoptera	Chalcidoidea	Encyrtidae	Encyrtidae	sp.	KR782719.1
Hymenoptera	Chalcidoidea	Encyrtidae	Encyrtidae	sp.	KR782935.1
Hymenoptera	Chalcidoidea	Encyrtidae	Encyrtidae	sp.	KR783273.1
Hymenoptera	Chalcidoidea	Encyrtidae	Encyrtidae	sp.	KR783469.1
Hymenoptera	Chalcidoidea	Encyrtidae	Encyrtidae	sp.	KR784244.1
Hymenoptera	Chalcidoidea	Encyrtidae	Encyrtidae	sp.	KR784521.1
Hymenoptera	Chalcidoidea	Encyrtidae	Encyrtidae	sp.	KR784811.1
Hymenoptera	Chalcidoidea	Encyrtidae	Encyrtidae	sp.	KR785296.1
Hymenoptera	Chalcidoidea	Encyrtidae	Encyrtidae	sp.	KR785365.1
Hymenoptera	Chalcidoidea	Encyrtidae	Encyrtidae	sp.	KR785415.1
Hymenoptera	Chalcidoidea	Encyrtidae	Encyrtidae	sp.	KR786147.1
Hymenoptera	Chalcidoidea	Encyrtidae	Encyrtidae	sp.	KR786241.1

Hymenoptera	Chalcidoidea	Encyrtidae	Encyrtidae	sp.	KR786258.1
Hymenoptera	Chalcidoidea	Encyrtidae	Encyrtidae	sp.	KR786298.1
Hymenoptera	Chalcidoidea	Encyrtidae	Encyrtidae	sp.	KR788595.1
Hymenoptera	Chalcidoidea	Encyrtidae	Encyrtidae	sp.	KR788904.1
Hymenoptera	Chalcidoidea	Encyrtidae	Encyrtidae	sp.	KR788926.1
Hymenoptera	Chalcidoidea	Encyrtidae	Encyrtidae	sp.	KR789888.1
Hymenoptera	Chalcidoidea	Encyrtidae	Encyrtidae	sp.	KR790974.1
Hymenoptera	Chalcidoidea	Encyrtidae	Encyrtidae	sp.	KR791568.1
Hymenoptera	Chalcidoidea	Encyrtidae	Encyrtidae	sp.	KR791611.1
Hymenoptera	Chalcidoidea	Encyrtidae	Encyrtidae	sp.	KR791896.1
Hymenoptera	Chalcidoidea	Encyrtidae	Encyrtidae	sp.	KR791987.1
Hymenoptera	Chalcidoidea	Encyrtidae	Encyrtidae	sp.	KR792013.1
Hymenoptera	Chalcidoidea	Encyrtidae	Encyrtidae	sp.	KR792649.1
Hymenoptera	Chalcidoidea	Encyrtidae	Encyrtidae	sp.	KR792650.1
Hymenoptera	Chalcidoidea	Encyrtidae	Encyrtidae	sp.	KR792669.1
Hymenoptera	Chalcidoidea	Encyrtidae	Encyrtidae	sp.	KR792898.1
Hymenoptera	Chalcidoidea	Encyrtidae	Encyrtidae	sp.	KR793338.1
Hymenoptera	Chalcidoidea	Encyrtidae	Encyrtidae	sp.	KR793556.1
Hymenoptera	Chalcidoidea	Encyrtidae	Encyrtidae	sp.	KR793890.1
Hymenoptera	Chalcidoidea	Encyrtidae	Encyrtidae	sp.	KR793980.1
Hymenoptera	Chalcidoidea	Encyrtidae	Encyrtidae	sp.	KR794036.1
Hymenoptera	Chalcidoidea	Encyrtidae	Encyrtidae	sp.	KR794447.1
Hymenoptera	Chalcidoidea	Encyrtidae	Encyrtidae	sp.	KR795194.1
Hymenoptera	Chalcidoidea	Encyrtidae	Encyrtidae	sp.	KR795811.1
Hymenoptera	Chalcidoidea	Encyrtidae	Encyrtidae	sp.	KR795909.1
Hymenoptera	Chalcidoidea	Encyrtidae	Encyrtidae	sp.	KR796296.1
Hymenoptera	Chalcidoidea	Encyrtidae	Encyrtidae	sp.	KR796541.1
Hymenoptera	Chalcidoidea	Encyrtidae	Encyrtidae	sp.	KR796935.1
Hymenoptera	Chalcidoidea	Encyrtidae	Encyrtidae	sp.	KR798256.1
Hymenoptera	Chalcidoidea	Encyrtidae	Encyrtidae	sp.	KR798298.1
Hymenoptera	Chalcidoidea	Encyrtidae	Encyrtidae	sp.	KR798745.1
Hymenoptera	Chalcidoidea	Encyrtidae	Encyrtidae	sp.	KR798877.1
Hymenoptera	Chalcidoidea	Encyrtidae	Encyrtidae	sp.	KR799479.1
Hymenoptera	Chalcidoidea	Encyrtidae	Encyrtidae	sp.	KR799635.1
Hymenoptera	Chalcidoidea	Encyrtidae	Encyrtidae	sp.	KR800019.1
Hymenoptera	Chalcidoidea	Encyrtidae	Encyrtidae	sp.	KR801157.1
Hymenoptera	Chalcidoidea	Encyrtidae	Encyrtidae	sp.	KR801812.1
Hymenoptera	Chalcidoidea	Encyrtidae	Encyrtidae	sp.	KR802391.1
Hymenoptera	Chalcidoidea	Encyrtidae	Encyrtidae	sp.	KR803064.1
Hymenoptera	Chalcidoidea	Encyrtidae	Encyrtidae	sp.	KR803597.1
Hymenoptera	Chalcidoidea	Encyrtidae	Encyrtidae	sp.	KR804217.1
Hymenoptera	Chalcidoidea	Encyrtidae	Encyrtidae	sp.	KR804224.1
Hymenoptera	Chalcidoidea	Encyrtidae	Encyrtidae	sp.	KR804521.1
Hymenoptera	Chalcidoidea	Encyrtidae	Encyrtidae	sp.	KR805039.1
Hymenoptera	Chalcidoidea	Encyrtidae	Encyrtidae	sp.	KR805056.1
Hymenoptera	Chalcidoidea	Encyrtidae	Encyrtidae	sp.	KR805159.1
Hymenoptera	Chalcidoidea	Encyrtidae	Encyrtidae	sp.	KR805546.1
Hymenoptera	Chalcidoidea	Encyrtidae	Encyrtidae	sp.	KR805761.1
Hymenoptera	Chalcidoidea	Encyrtidae	Encyrtidae	sp.	KR805837.1
Hymenoptera	Chalcidoidea	Encyrtidae	Encyrtidae	sp.	KR806187.1
Hymenoptera	Chalcidoidea	Encyrtidae	Encyrtidae	sp.	KR806420.1
Hymenoptera	Chalcidoidea	Encyrtidae	Encyrtidae	sp.	KR806930.1
Hymenoptera	Chalcidoidea	Encyrtidae	Encyrtidae	sp.	KR807458.1
Hymenoptera	Chalcidoidea	Encyrtidae	Encyrtidae	sp.	KR807914.1

Hymenoptera	Chalcidoidea	Encyrtidae	Encyrtidae	sp.	KR808007.1
Hymenoptera	Chalcidoidea	Encyrtidae	Encyrtidae	sp.	KR808171.1
Hymenoptera	Chalcidoidea	Encyrtidae	Encyrtidae	sp.	KR808653.1
Hymenoptera	Chalcidoidea	Encyrtidae	Encyrtidae	sp.	KR808675.1
Hymenoptera	Chalcidoidea	Encyrtidae	Encyrtidae	sp.	KR808728.1
Hymenoptera	Chalcidoidea	Encyrtidae	Encyrtidae	sp.	KR808769.1
Hymenoptera	Chalcidoidea	Encyrtidae	Encyrtidae	sp.	KR809182.1
Hymenoptera	Chalcidoidea	Encyrtidae	Encyrtidae	sp.	KR873691.1
Hymenoptera	Chalcidoidea	Encyrtidae	Encyrtidae	sp.	KR874139.1
Hymenoptera	Chalcidoidea	Encyrtidae	Encyrtidae	sp.	KR874479.1
Hymenoptera	Chalcidoidea	Encyrtidae	Encyrtidae	sp.	KR876988.1
Hymenoptera	Chalcidoidea	Encyrtidae	Encyrtidae	sp.	KR877402.1
Hymenoptera	Chalcidoidea	Encyrtidae	Encyrtidae	sp.	KR877446.1
Hymenoptera	Chalcidoidea	Encyrtidae	Encyrtidae	sp.	KR878943.1
Hymenoptera	Chalcidoidea	Encyrtidae	Encyrtidae	sp.	KR879007.1
Hymenoptera	Chalcidoidea	Encyrtidae	Encyrtidae	sp.	KR879260.1
Hymenoptera	Chalcidoidea	Encyrtidae	Encyrtidae	sp.	KR881093.1
Hymenoptera	Chalcidoidea	Encyrtidae	Encyrtidae	sp.	KR881127.1
Hymenoptera	Chalcidoidea	Encyrtidae	Encyrtidae	sp.	KR881736.1
Hymenoptera	Chalcidoidea	Encyrtidae	Encyrtidae	sp.	KR883045.1
Hymenoptera	Chalcidoidea	Encyrtidae	Encyrtidae	sp.	KR885232.1
Hymenoptera	Chalcidoidea	Encyrtidae	Encyrtidae	sp.	KR885833.1
Hymenoptera	Chalcidoidea	Encyrtidae	Encyrtidae	sp.	KR886023.1
Hymenoptera	Chalcidoidea	Encyrtidae	Encyrtidae	sp.	KR887827.1
Hymenoptera	Chalcidoidea	Encyrtidae	Encyrtidae	sp.	KR887906.1
Hymenoptera	Chalcidoidea	Encyrtidae	Encyrtidae	sp.	KR888056.1
Hymenoptera	Chalcidoidea	Encyrtidae	Encyrtidae	sp.	KR888928.1
Hymenoptera	Chalcidoidea	Encyrtidae	Encyrtidae	sp.	KR889939.1
Hymenoptera	Chalcidoidea	Encyrtidae	Encyrtidae	sp.	KR890496.1
Hymenoptera	Chalcidoidea	Encyrtidae	Encyrtidae	sp.	KR892140.1
Hymenoptera	Chalcidoidea	Encyrtidae	Encyrtidae	sp.	KR892427.1
Hymenoptera	Chalcidoidea	Encyrtidae	Encyrtidae	sp.	KR893209.1
Hymenoptera	Chalcidoidea	Encyrtidae	Encyrtidae	sp.	KR894330.1
Hymenoptera	Chalcidoidea	Encyrtidae	Encyrtidae	sp.	KR896389.1
Hymenoptera	Chalcidoidea	Encyrtidae	Encyrtidae	sp.	KR897243.1
Hymenoptera	Chalcidoidea	Encyrtidae	Encyrtidae	sp.	KR897263.1
Hymenoptera	Chalcidoidea	Encyrtidae	Encyrtidae	sp.	KR897806.1
Hymenoptera	Chalcidoidea	Encyrtidae	Encyrtidae	sp.	KR899008.1
Hymenoptera	Chalcidoidea	Encyrtidae	Encyrtidae	sp.	KR899079.1
Hymenoptera	Chalcidoidea	Encyrtidae	Encyrtidae	sp.	KR899791.1
Hymenoptera	Chalcidoidea	Encyrtidae	Encyrtidae	sp.	KR900526.1
Hymenoptera	Chalcidoidea	Encyrtidae	Encyrtidae	sp.	KR901604.1
Hymenoptera	Chalcidoidea	Encyrtidae	Encyrtidae	sp.	KR902270.1
Hymenoptera	Chalcidoidea	Encyrtidae	Encyrtidae	sp.	KR928230.1
Hymenoptera	Chalcidoidea	Encyrtidae	Encyrtidae	sp.	KT619841.1
Hymenoptera	Chalcidoidea	Encyrtidae	Encyrtidae	sp.	KT620043.1
Hymenoptera	Chalcidoidea	Encyrtidae	Encyrtidae	sp.	KT622952.1
Hymenoptera	Chalcidoidea	Encyrtidae	Encyrtidae	sp.	KY831595.1
Hymenoptera	Chalcidoidea	Encyrtidae	Encyrtidae	sp.	KY833506.1
Hymenoptera	Chalcidoidea	Encyrtidae	Encyrtidae	sp.	KY833651.1
Hymenoptera	Chalcidoidea	Encyrtidae	Encyrtidae	sp.	KY837312.1
Hymenoptera	Chalcidoidea	Encyrtidae	Encyrtidae	sp.	KY837585.1
Hymenoptera	Chalcidoidea	Encyrtidae	Encyrtidae	sp.	KY839039.1
Hymenoptera	Chalcidoidea	Encyrtidae	Encyrtidae	sp.	KY841897.1

Hymenoptera	Chalcidoidea	Encyrtidae	Encyrtidae	sp.	KY844858.1
Hymenoptera	Chalcidoidea	Encyrtidae	Encyrtidae	sp.	KY845087.1
Hymenoptera	Chalcidoidea	Encyrtidae	Encyrtidae	sp.	KY846197.1
Hymenoptera	Chalcidoidea	Encyrtidae	Encyrtidae	sp.	KY846706.1
Hymenoptera	Chalcidoidea	Encyrtidae	Encyrtidae	sp.	MF898949.1
Hymenoptera	Chalcidoidea	Encyrtidae	Encyrtidae	sp.	MF898985.1
Hymenoptera	Chalcidoidea	Encyrtidae	Encyrtidae	sp.	MF899394.1
Hymenoptera	Chalcidoidea	Encyrtidae	Encyrtidae	sp.	MF899771.1
Hymenoptera	Chalcidoidea	Encyrtidae	Encyrtidae	sp.	MF899875.1
Hymenoptera	Chalcidoidea	Encyrtidae	Encyrtidae	sp.	MF900029.1
Hymenoptera	Chalcidoidea	Encyrtidae	Encyrtidae	sp.	MF900097.1
Hymenoptera	Chalcidoidea	Encyrtidae	Encyrtidae	sp.	MF900347.1
Hymenoptera	Chalcidoidea	Encyrtidae	Encyrtidae	sp.	MF900367.1
Hymenoptera	Chalcidoidea	Encyrtidae	Encyrtidae	sp.	MF902176.1
Hymenoptera	Chalcidoidea	Encyrtidae	Encyrtidae	sp.	MF903383.1
Hymenoptera	Chalcidoidea	Encyrtidae	Encyrtidae	sp.	MF903802.1
Hymenoptera	Chalcidoidea	Encyrtidae	Encyrtidae	sp.	MF903935.1
Hymenoptera	Chalcidoidea	Encyrtidae	Encyrtidae	sp.	MF905154.1
Hymenoptera	Chalcidoidea	Encyrtidae	Encyrtidae	sp.	MF907102.1
Hymenoptera	Chalcidoidea	Encyrtidae	Encyrtidae	sp.	MF907201.1
Hymenoptera	Chalcidoidea	Encyrtidae	Encyrtidae	sp.	MF907754.1
Hymenoptera	Chalcidoidea	Encyrtidae	Encyrtidae	sp.	MF908179.1
Hymenoptera	Chalcidoidea	Encyrtidae	Encyrtidae	sp.	MF908377.1
Hymenoptera	Chalcidoidea	Encyrtidae	Encyrtidae	sp.	MG438549.1
Hymenoptera	Chalcidoidea	Encyrtidae	Encyrtidae	sp.	MG438821.1
Hymenoptera	Chalcidoidea	Encyrtidae	Encyrtidae	sp.	MG438906.1
Hymenoptera	Chalcidoidea	Encyrtidae	Encyrtidae	sp.	MG438926.1
Hymenoptera	Chalcidoidea	Encyrtidae	Encyrtidae	sp.	MG439014.1
Hymenoptera	Chalcidoidea	Encyrtidae	Encyrtidae	sp.	MG439367.1
Hymenoptera	Chalcidoidea	Encyrtidae	Encyrtidae	sp.	MG439731.1
Hymenoptera	Chalcidoidea	Encyrtidae	Encyrtidae	sp.	MG441253.1
Hymenoptera	Chalcidoidea	Encyrtidae	Encyrtidae	sp.	MG441427.1
Hymenoptera	Chalcidoidea	Encyrtidae	Encyrtidae	sp.	MG441463.1
Hymenoptera	Chalcidoidea	Encyrtidae	Encyrtidae	sp.	MG441491.1
Hymenoptera	Chalcidoidea	Encyrtidae	Encyrtidae	sp.	MG441803.1
Hymenoptera	Chalcidoidea	Encyrtidae	Encyrtidae	sp.	MG441825.1
Hymenoptera	Chalcidoidea	Encyrtidae	Encyrtidae	sp.	MG441922.1
Hymenoptera	Chalcidoidea	Encyrtidae	Encyrtidae	sp.	MG442038.1
Hymenoptera	Chalcidoidea	Encyrtidae	Encyrtidae	sp.	MG442551.1
Hymenoptera	Chalcidoidea	Encyrtidae	Encyrtidae	sp.	MG442673.1
Hymenoptera	Chalcidoidea	Encyrtidae	Encyrtidae	sp.	MG442824.1
Hymenoptera	Chalcidoidea	Encyrtidae	Encyrtidae	sp.	MG442834.1
Hymenoptera	Chalcidoidea	Encyrtidae	Encyrtidae	sp.	MG442977.1
Hymenoptera	Chalcidoidea	Encyrtidae	Encyrtidae	sp.	MG443430.1
Hymenoptera	Chalcidoidea	Encyrtidae	Encyrtidae	sp.	MG443445.1
Hymenoptera	Chalcidoidea	Encyrtidae	Encyrtidae	sp.	MG443852.1
Hymenoptera	Chalcidoidea	Encyrtidae	Encyrtidae	sp.	MG443955.1
Hymenoptera	Chalcidoidea	Encyrtidae	Encyrtidae	sp.	MG444218.1
Hymenoptera	Chalcidoidea	Encyrtidae	Encyrtidae	sp.	MG444242.1
Hymenoptera	Chalcidoidea	Encyrtidae	Encyrtidae	sp.	MG444343.1
Hymenoptera	Chalcidoidea	Encyrtidae	Encyrtidae	sp.	MG444604.1
Hymenoptera	Chalcidoidea	Encyrtidae	Encyrtidae	sp.	MG444674.1
Hymenoptera	Chalcidoidea	Encyrtidae	Encyrtidae	sp.	MG444677.1
Hymenoptera	Chalcidoidea	Encyrtidae	Encyrtidae	sp.	MG444717.1

Hymenoptera	Chalcidoidea	Encyrtidae	Encyrtidae	sp.	MG444798.1
Hymenoptera	Chalcidoidea	Encyrtidae	Encyrtidae	sp.	MG444856.1
Hymenoptera	Chalcidoidea	Encyrtidae	Encyrtidae	sp.	MG445203.1
Hymenoptera	Chalcidoidea	Encyrtidae	Encyrtidae	sp.	MG445227.1
Hymenoptera	Chalcidoidea	Encyrtidae	Encyrtidae	sp.	MG445277.1
Hymenoptera	Chalcidoidea	Encyrtidae	Encyrtidae	sp.	MG445577.1
Hymenoptera	Chalcidoidea	Encyrtidae	Encyrtidae	sp.	MG445610.1
Hymenoptera	Chalcidoidea	Encyrtidae	Encyrtidae	sp.	MG445973.1
Hymenoptera	Chalcidoidea	Encyrtidae	Encyrtidae	sp.	MG446677.1
Hymenoptera	Chalcidoidea	Encyrtidae	Encyrtidae	sp.	MG446816.1
Hymenoptera	Chalcidoidea	Encyrtidae	Encyrtidae	sp.	MG447229.1
Hymenoptera	Chalcidoidea	Encyrtidae	Encyrtidae	sp.	MG447701.1
Hymenoptera	Chalcidoidea	Encyrtidae	Encyrtidae	sp.	MG447773.1
Hymenoptera	Chalcidoidea	Encyrtidae	Encyrtidae	sp.	MG448053.1
Hymenoptera	Chalcidoidea	Encyrtidae	Encyrtidae	sp.	MG448333.1
Hymenoptera	Chalcidoidea	Encyrtidae	Encyrtidae	sp.	MG480261.1
Hymenoptera	Chalcidoidea	Encyrtidae	Encyrtidae	sp.	MG498615.1
Hymenoptera	Chalcidoidea	Encyrtidae	Encyrtidae	sp.	MH456773.1
Hymenoptera	Chalcidoidea	Encyrtidae	Encyrtidae	sp.	MH926937.1
Hymenoptera	Chalcidoidea	Encyrtidae	Encyrtidae	sp.	MH927888.1
Hymenoptera	Chalcidoidea	Encyrtidae	Encyrtidae	sp.	MN669097.1
Hymenoptera	Chalcidoidea	Encyrtidae	Encyrtidae	sp.	MN676849.1
Hymenoptera	Chalcidoidea	Encyrtidae	Encyrtidae	sp.	MN682725.1
Hymenoptera	Chalcidoidea	Encyrtidae	Encyrtidae	sp.	MZ630086.1
Hymenoptera	Chalcidoidea	Encyrtidae	Encyrtidae	sp.	OL694542.1
Hymenoptera	Chalcidoidea	Encyrtidae	Encyrtidae	sp.	OL694553.1
Hymenoptera	Chalcidoidea	Encyrtidae	Encyrtidae	sp.	OL694559.1
Hymenoptera	Chalcidoidea	Encyrtidae	Homalotylus	mirabilis	MH979997.1
Hymenoptera	Chalcidoidea	Encyrtidae	Homalotylus	terminalis	MH979998.1
Hymenoptera	Chalcidoidea	Encyrtidae	Leptomastidea	hypogeococci	MG731496.1
Hymenoptera	Chalcidoidea	Encyrtidae	Leptomastidea	hypogeococci	MG731497.1
Hymenoptera	Chalcidoidea	Encyrtidae	Leptomastidea	hypogeococci	MG731498.1
Hymenoptera	Chalcidoidea	Encyrtidae	Leptomastidea	hypogeococci	MG731499.1
Hymenoptera	Chalcidoidea	Encyrtidae	Metaphycus	aeckei	MH456594.1
Hymenoptera	Chalcidoidea	Encyrtidae	Metaphycus	corniae	MH119409.1
Hymenoptera	Chalcidoidea	Encyrtidae	Metaphycus	corniae	MH119410.1
Hymenoptera	Chalcidoidea	Encyrtidae	Metaphycus	cylindricus	MH119345.1
Hymenoptera	Chalcidoidea	Encyrtidae	Metaphycus	cylindricus	MH119346.1
Hymenoptera	Chalcidoidea	Encyrtidae	Metaphycus	cylindricus	MH119347.1
Hymenoptera	Chalcidoidea	Encyrtidae	Metaphycus	cylindricus	MH119349.1
Hymenoptera	Chalcidoidea	Encyrtidae	Metaphycus	cylindricus	MH119353.1
Hymenoptera	Chalcidoidea	Encyrtidae	Metaphycus	cylindricus	MH119354.1
Hymenoptera	Chalcidoidea	Encyrtidae	Metaphycus	cylindricus	MH119355.1
Hymenoptera	Chalcidoidea	Encyrtidae	Metaphycus	cylindricus	MH119357.1
Hymenoptera	Chalcidoidea	Encyrtidae	Metaphycus	eriococci	MH119359.1
Hymenoptera	Chalcidoidea	Encyrtidae	Metaphycus	eriococci	MH119364.1
Hymenoptera	Chalcidoidea	Encyrtidae	Metaphycus	eriococci	MH119365.1
Hymenoptera	Chalcidoidea	Encyrtidae	Metaphycus	eriococci	MH119366.1
Hymenoptera	Chalcidoidea	Encyrtidae	Metaphycus	eriococci	MH119367.1
Hymenoptera	Chalcidoidea	Encyrtidae	Metaphycus	eriococci	MH119371.1
Hymenoptera	Chalcidoidea	Encyrtidae	Metaphycus	eriococci	MH119378.1
Hymenoptera	Chalcidoidea	Encyrtidae	Metaphycus	eriococci	MH119379.1
Hymenoptera	Chalcidoidea	Encyrtidae	Metaphycus	flavus	FM210164.1
Hymenoptera	Chalcidoidea	Encyrtidae	Metaphycus	flavus	MG946790.1

Hymenoptera	Chalcidoidea	Encyrtidae	Metaphycus	flavus	MG946791.1
Hymenoptera	Chalcidoidea	Encyrtidae	Metaphycus	flavus	MH456723.1
Hymenoptera	Chalcidoidea	Encyrtidae	Metaphycus	flavus	MH456778.1
Hymenoptera	Chalcidoidea	Encyrtidae	Metaphycus	flavus	MH456780.1
Hymenoptera	Chalcidoidea	Encyrtidae	Metaphycus	garmon	MH119414.1
Hymenoptera	Chalcidoidea	Encyrtidae	Metaphycus	garmon	MH119417.1
Hymenoptera	Chalcidoidea	Encyrtidae	Metaphycus	garmon	MH119419.1
Hymenoptera	Chalcidoidea	Encyrtidae	Metaphycus	garmon	MH119420.1
Hymenoptera	Chalcidoidea	Encyrtidae	Metaphycus	gerardi	MH119423.1
Hymenoptera	Chalcidoidea	Encyrtidae	Metaphycus	groenlandicus	KR783093.1
Hymenoptera	Chalcidoidea	Encyrtidae	Metaphycus	groenlandicus	KR784386.1
Hymenoptera	Chalcidoidea	Encyrtidae	Metaphycus	groenlandicus	KR874706.1
Hymenoptera	Chalcidoidea	Encyrtidae	Metaphycus	groenlandicus	KR890545.1
Hymenoptera	Chalcidoidea	Encyrtidae	Metaphycus	groenlandicus	MG444018.1
Hymenoptera	Chalcidoidea	Encyrtidae	Metaphycus	lounsburyi	MH456508.1
Hymenoptera	Chalcidoidea	Encyrtidae	Metaphycus	nitens	MH119429.1
Hymenoptera	Chalcidoidea	Encyrtidae	Metaphycus	sp.	MH456591.1
Hymenoptera	Chalcidoidea	Encyrtidae	Metaphycus	stanleyi	MH456614.1
Hymenoptera	Chalcidoidea	Encyrtidae	Microterys	sp.	MH119388.1
Hymenoptera	Chalcidoidea	Encyrtidae	Microterys	sp.	MH119389.1
Hymenoptera	Chalcidoidea	Encyrtidae	Microterys	sp.	MH119391.1
Hymenoptera	Chalcidoidea	Encyrtidae	Neastymachus	axillaris	KM095502.1
Hymenoptera	Chalcidoidea	Encyrtidae	Oobius	agrili	MF568016.1
Hymenoptera	Chalcidoidea	Encyrtidae	Oobius	buprestidis	MF568009.1
Hymenoptera	Chalcidoidea	Encyrtidae	Oobius	fleischeri	MF568010.1
Hymenoptera	Chalcidoidea	Encyrtidae	Oobius	primorskyensis	MF568018.1
Hymenoptera	Chalcidoidea	Encyrtidae	Ooencyrtus	ferdowsii	KR270994.1
Hymenoptera	Chalcidoidea	Encyrtidae	Ooencyrtus	nezarae	KY964494.1
Hymenoptera	Chalcidoidea	Encyrtidae	Ooencyrtus	sp.	KC149976.1
Hymenoptera	Chalcidoidea	Encyrtidae	Ooencyrtus	sp.	KF724500.1
Hymenoptera	Chalcidoidea	Encyrtidae	Ooencyrtus	sp.	KF724501.1
Hymenoptera	Chalcidoidea	Encyrtidae	Praleurocerus	viridis	KJ955497.1
Hymenoptera	Chalcidoidea	Encyrtidae	Pseudencyrtus	sp.	KU373908.1
Hymenoptera	Chalcidoidea	Encyrtidae	Pseudencyrtus	sp.	MN668512.1
Hymenoptera	Chalcidoidea	Encyrtidae	Syrphophagus	aphidivorus	JF906507.1
Hymenoptera	Chalcidoidea	Encyrtidae	Syrphophagus	aphidivorus	JX507455.1
Hymenoptera	Chalcidoidea	Encyrtidae	Syrphophagus	aphidivorus	MF979499.1
Hymenoptera	Chalcidoidea	Encyrtidae	Syrphophagus	aphidivorus	MF979500.1
Hymenoptera	Chalcidoidea	Encyrtidae	Syrphophagus	aphidivorus	MF979501.1
Hymenoptera	Chalcidoidea	Encyrtidae	Syrphophagus	aphidivorus	MF979502.1
Hymenoptera	Chalcidoidea	Encyrtidae	Syrphophagus	tachikawai	LC260617.1
Hymenoptera	Chalcidoidea	Encyrtidae	Syrphophagus	tachikawai	LC260618.1
Hymenoptera	Chalcidoidea	Encyrtidae	Syrphophagus	tachikawai	LC260619.1
Hymenoptera	Chalcidoidea	Encyrtidae	Syrphophagus	tachikawai	LC260620.1
Hymenoptera	Chalcidoidea	Encyrtidae	UNVERIFIED	Copidosoma	MG440639.1
Hymenoptera	Chalcidoidea	Encyrtidae	UNVERIFIED	Ooencyrtus	KF724504.1
Hymenoptera	Chalcidoidea	Encyrtidae	Zaomma	eriococci	MH119396.1
Hymenoptera	Chalcidoidea	Encyrtidae	Zaomma	eriococci	MH119397.1
Hymenoptera	Chalcidoidea	Encyrtidae	Zaomma	eriococci	MH119398.1
Hymenoptera	Chalcidoidea	Encyrtidae	Zaomma	eriococci	MH119399.1
Hymenoptera	Chalcidoidea	Encyrtidae	Zaomma	eriococci	MH119401.1
Hymenoptera	Chalcidoidea	Encyrtidae	Zaomma	eriococci	MH119402.1
Hymenoptera	Chalcidoidea	Encyrtidae	Zaomma	eriococci	MH119403.1
Hymenoptera	Chalcidoidea	Encyrtidae	Zaomma	eriococci	MH119404.1

Hymenoptera	Chalcidoidea	Encyrtidae	Zaomma	eriococci	MH119406.1
Hymenoptera	Chalcidoidea	Encyrtinae	Encyrtinae	sp.	MG444525.1
Hymenoptera	Chalcidoidea	Eriaporidae	Myiocnema	comperei	KJ955498.1
Hymenoptera	Chalcidoidea	Eriaporidae	Promuscidea	unfasciativentris	AB667989.1
Hymenoptera	Chalcidoidea	Eucharitidae	Eucharitidae	sp.	KR800413.1
Hymenoptera	Chalcidoidea	Eucharitidae	Eucharitidae	sp.	KR881925.1
Hymenoptera	Chalcidoidea	Eucharitidae	Eucharitidae	sp.	MF902912.1
Hymenoptera	Chalcidoidea	Eucharitidae	Eucharitidae	sp.	MF906044.1
Hymenoptera	Chalcidoidea	Eucharitidae	Eucharitidae	sp.	MH926917.1
Hymenoptera	Chalcidoidea	Eucharitidae	Eucharitidae	sp.	MH927181.1
Hymenoptera	Chalcidoidea	Eucharitidae	Eucharitidae	sp.	MH927201.1
Hymenoptera	Chalcidoidea	Eucharitidae	Eucharitidae	sp.	MH928833.1
Hymenoptera	Chalcidoidea	Eucharitidae	Orasema	aenea	KR733124.1
Hymenoptera	Chalcidoidea	Eucharitidae	Orasema	aenea	KR733126.1
Hymenoptera	Chalcidoidea	Eucharitidae	Orasema	aureoviridis	KR733114.1
Hymenoptera	Chalcidoidea	Eucharitidae	Orasema	aureoviridis	KR733115.1
Hymenoptera	Chalcidoidea	Eucharitidae	Orasema	bakeri	KR733117.1
Hymenoptera	Chalcidoidea	Eucharitidae	Orasema	bakeri	KR733127.1
Hymenoptera	Chalcidoidea	Eucharitidae	Orasema	bakeri	KR733142.1
Hymenoptera	Chalcidoidea	Eucharitidae	Orasema	cockerelli	KR733121.1
Hymenoptera	Chalcidoidea	Eucharitidae	Orasema	cockerelli	KR733129.1
Hymenoptera	Chalcidoidea	Eucharitidae	Orasema	coloradensis	KR733123.1
Hymenoptera	Chalcidoidea	Eucharitidae	Orasema	simulatrix	KR733130.1
Hymenoptera	Chalcidoidea	Eucharitidae	Orasema	simulatrix	KR733135.1
Hymenoptera	Chalcidoidea	Eucharitidae	Orasema	simulatrix	KR733138.1
Hymenoptera	Chalcidoidea	Eucharitidae	Orasema	simulatrix	KR733139.1
Hymenoptera	Chalcidoidea	Eucharitidae	Orasema	simulatrix	KR733141.1
Hymenoptera	Chalcidoidea	Eucharitidae	Orasema	sp.	KR733116.1
Hymenoptera	Chalcidoidea	Eucharitidae	Orasema	sp.	KR733118.1
Hymenoptera	Chalcidoidea	Eucharitidae	Orasema	sp.	KR733119.1
Hymenoptera	Chalcidoidea	Eucharitidae	Orasema	sp.	KR733120.1
Hymenoptera	Chalcidoidea	Eucharitidae	Orasema	sp.	KR733125.1
Hymenoptera	Chalcidoidea	Eucharitidae	Orasema	xanthopus	KR733122.1
Hymenoptera	Chalcidoidea	Eucharitidae	Orasema	xanthopus	KR733143.1
Hymenoptera	Chalcidoidea	Eucharitidae	Psilocharis	afra	KC213237.1
Hymenoptera	Chalcidoidea	Eucharitidae	UNVERIFIED	Eucharitidae	MG439914.1
Hymenoptera	Chalcidoidea	Eulophidae	Achrysocharoides	acerianus	MZ631371.1
Hymenoptera	Chalcidoidea	Eulophidae	Achrysocharoides	acerianus	MZ631829.1
Hymenoptera	Chalcidoidea	Eulophidae	Achrysocharoides	acerianus	MZ632585.1
Hymenoptera	Chalcidoidea	Eulophidae	Achrysocharoides	cilla	KR782528.1
Hymenoptera	Chalcidoidea	Eulophidae	Achrysocharoides	cilla	KR784657.1
Hymenoptera	Chalcidoidea	Eulophidae	Achrysocharoides	cilla	MG836432.1
Hymenoptera	Chalcidoidea	Eulophidae	Achrysocharoides	sp.	HM365040.1
Hymenoptera	Chalcidoidea	Eulophidae	Achrysocharoides	sp.	KR795680.1
Hymenoptera	Chalcidoidea	Eulophidae	Achrysocharoides	sp.	KR798650.1
Hymenoptera	Chalcidoidea	Eulophidae	Achrysocharoides	sp.	MZ632618.1
Hymenoptera	Chalcidoidea	Eulophidae	Anaprostocetus	acuminatus	KT599310.1
Hymenoptera	Chalcidoidea	Eulophidae	Aprostocetus	blastophagusi	MG836478.1
Hymenoptera	Chalcidoidea	Eulophidae	Aprostocetus	caudatus	MG836477.1
Hymenoptera	Chalcidoidea	Eulophidae	Aprostocetus	caudatus	MG836483.1
Hymenoptera	Chalcidoidea	Eulophidae	Aprostocetus	caudatus	MG836484.1
Hymenoptera	Chalcidoidea	Eulophidae	Aprostocetus	ceroplastae	MG836486.1
Hymenoptera	Chalcidoidea	Eulophidae	Aprostocetus	ciliatus	MG836501.1
Hymenoptera	Chalcidoidea	Eulophidae	Aprostocetus	citrinus	MG836490.1

Hymenoptera	Chalcidoidea	Eulophidae	Aprostocetus	clavicornis	MG836492.1
Hymenoptera	Chalcidoidea	Eulophidae	Aprostocetus	crypturgus	MG836489.1
Hymenoptera	Chalcidoidea	Eulophidae	Aprostocetus	csokakoensis	MG836485.1
Hymenoptera	Chalcidoidea	Eulophidae	Aprostocetus	dendroctoni	MG836488.1
Hymenoptera	Chalcidoidea	Eulophidae	Aprostocetus	dryocosmi	MG836479.1
Hymenoptera	Chalcidoidea	Eulophidae	Aprostocetus	epicharmus	MG836480.1
Hymenoptera	Chalcidoidea	Eulophidae	Aprostocetus	fukutai	MG836499.1
Hymenoptera	Chalcidoidea	Eulophidae	Aprostocetus	gala	KF817576.1
Hymenoptera	Chalcidoidea	Eulophidae	Aprostocetus	ilexi	MG836493.1
Hymenoptera	Chalcidoidea	Eulophidae	Aprostocetus	massonianae	MG836495.1
Hymenoptera	Chalcidoidea	Eulophidae	Aprostocetus	meltoftei	HQ566560.1
Hymenoptera	Chalcidoidea	Eulophidae	Aprostocetus	meltoftei	HQ566567.1
Hymenoptera	Chalcidoidea	Eulophidae	Aprostocetus	meltoftei	HQ566568.1
Hymenoptera	Chalcidoidea	Eulophidae	Aprostocetus	meltoftei	KR800425.1
Hymenoptera	Chalcidoidea	Eulophidae	Aprostocetus	meltoftei	KR803780.1
Hymenoptera	Chalcidoidea	Eulophidae	Aprostocetus	meltoftei	KR805635.1
Hymenoptera	Chalcidoidea	Eulophidae	Aprostocetus	meltoftei	KU374349.1
Hymenoptera	Chalcidoidea	Eulophidae	Aprostocetus	meltoftei	KU374533.1
Hymenoptera	Chalcidoidea	Eulophidae	Aprostocetus	meltoftei	MN668995.1
Hymenoptera	Chalcidoidea	Eulophidae	Aprostocetus	meltoftei	MN670585.1
Hymenoptera	Chalcidoidea	Eulophidae	Aprostocetus	meltoftei	MN674601.1
Hymenoptera	Chalcidoidea	Eulophidae	Aprostocetus	meltoftei	MN674910.1
Hymenoptera	Chalcidoidea	Eulophidae	Aprostocetus	meltoftei	MN675099.1
Hymenoptera	Chalcidoidea	Eulophidae	Aprostocetus	meltoftei	MN676073.1
Hymenoptera	Chalcidoidea	Eulophidae	Aprostocetus	meltoftei	MN676683.1
Hymenoptera	Chalcidoidea	Eulophidae	Aprostocetus	meltoftei	MN678249.1
Hymenoptera	Chalcidoidea	Eulophidae	Aprostocetus	meltoftei	MN679643.1
Hymenoptera	Chalcidoidea	Eulophidae	Aprostocetus	meltoftei	MN679966.1
Hymenoptera	Chalcidoidea	Eulophidae	Aprostocetus	meltoftei	MN681549.1
Hymenoptera	Chalcidoidea	Eulophidae	Aprostocetus	minimus	MG836476.1
Hymenoptera	Chalcidoidea	Eulophidae	Aprostocetus	monacoi	KP233971.1
Hymenoptera	Chalcidoidea	Eulophidae	Aprostocetus	prolixus	MG836473.1
Hymenoptera	Chalcidoidea	Eulophidae	Aprostocetus	purpureus	MG836497.1
Hymenoptera	Chalcidoidea	Eulophidae	Aprostocetus	sp.	HM374826.1
Hymenoptera	Chalcidoidea	Eulophidae	Aprostocetus	sp.	HM414501.1
Hymenoptera	Chalcidoidea	Eulophidae	Aprostocetus	sp.	HQ106659.1
Hymenoptera	Chalcidoidea	Eulophidae	Aprostocetus	sp.	HQ929648.1
Hymenoptera	Chalcidoidea	Eulophidae	Aprostocetus	sp.	JN293500.1
Hymenoptera	Chalcidoidea	Eulophidae	Aprostocetus	sp.	KF444810.1
Hymenoptera	Chalcidoidea	Eulophidae	Aprostocetus	sp.	KJ092579.1
Hymenoptera	Chalcidoidea	Eulophidae	Aprostocetus	sp.	KJ165188.1
Hymenoptera	Chalcidoidea	Eulophidae	Aprostocetus	sp.	KJ165473.1
Hymenoptera	Chalcidoidea	Eulophidae	Aprostocetus	sp.	KJ165560.1
Hymenoptera	Chalcidoidea	Eulophidae	Aprostocetus	sp.	KJ167669.1
Hymenoptera	Chalcidoidea	Eulophidae	Aprostocetus	sp.	KJ207798.1
Hymenoptera	Chalcidoidea	Eulophidae	Aprostocetus	sp.	KJ208585.1
Hymenoptera	Chalcidoidea	Eulophidae	Aprostocetus	sp.	KJ208718.1
Hymenoptera	Chalcidoidea	Eulophidae	Aprostocetus	sp.	KJ445362.1
Hymenoptera	Chalcidoidea	Eulophidae	Aprostocetus	sp.	KJ445450.1
Hymenoptera	Chalcidoidea	Eulophidae	Aprostocetus	sp.	KR401339.1
Hymenoptera	Chalcidoidea	Eulophidae	Aprostocetus	sp.	KR401711.1
Hymenoptera	Chalcidoidea	Eulophidae	Aprostocetus	sp.	KR403191.1
Hymenoptera	Chalcidoidea	Eulophidae	Aprostocetus	sp.	KR404100.1
Hymenoptera	Chalcidoidea	Eulophidae	Aprostocetus	sp.	KR406765.1

Hymenoptera	Chalcidoidea	Eulophidae	Aprostocetus	sp.	KR407390.1
Hymenoptera	Chalcidoidea	Eulophidae	Aprostocetus	sp.	KR410937.1
Hymenoptera	Chalcidoidea	Eulophidae	Aprostocetus	sp.	KR413747.1
Hymenoptera	Chalcidoidea	Eulophidae	Aprostocetus	sp.	KR415089.1
Hymenoptera	Chalcidoidea	Eulophidae	Aprostocetus	sp.	KR415646.1
Hymenoptera	Chalcidoidea	Eulophidae	Aprostocetus	sp.	KR415670.1
Hymenoptera	Chalcidoidea	Eulophidae	Aprostocetus	sp.	KR416614.1
Hymenoptera	Chalcidoidea	Eulophidae	Aprostocetus	sp.	KR420804.1
Hymenoptera	Chalcidoidea	Eulophidae	Aprostocetus	sp.	KR422288.1
Hymenoptera	Chalcidoidea	Eulophidae	Aprostocetus	sp.	KR782641.1
Hymenoptera	Chalcidoidea	Eulophidae	Aprostocetus	sp.	KR783020.1
Hymenoptera	Chalcidoidea	Eulophidae	Aprostocetus	sp.	KR783353.1
Hymenoptera	Chalcidoidea	Eulophidae	Aprostocetus	sp.	KR785936.1
Hymenoptera	Chalcidoidea	Eulophidae	Aprostocetus	sp.	KR786522.1
Hymenoptera	Chalcidoidea	Eulophidae	Aprostocetus	sp.	KR787236.1
Hymenoptera	Chalcidoidea	Eulophidae	Aprostocetus	sp.	KR788310.1
Hymenoptera	Chalcidoidea	Eulophidae	Aprostocetus	sp.	KR788369.1
Hymenoptera	Chalcidoidea	Eulophidae	Aprostocetus	sp.	KR789541.1
Hymenoptera	Chalcidoidea	Eulophidae	Aprostocetus	sp.	KR790111.1
Hymenoptera	Chalcidoidea	Eulophidae	Aprostocetus	sp.	KR790692.1
Hymenoptera	Chalcidoidea	Eulophidae	Aprostocetus	sp.	KR792480.1
Hymenoptera	Chalcidoidea	Eulophidae	Aprostocetus	sp.	KR792531.1
Hymenoptera	Chalcidoidea	Eulophidae	Aprostocetus	sp.	KR793870.1
Hymenoptera	Chalcidoidea	Eulophidae	Aprostocetus	sp.	KR794997.1
Hymenoptera	Chalcidoidea	Eulophidae	Aprostocetus	sp.	KR796009.1
Hymenoptera	Chalcidoidea	Eulophidae	Aprostocetus	sp.	KR796143.1
Hymenoptera	Chalcidoidea	Eulophidae	Aprostocetus	sp.	KR796205.1
Hymenoptera	Chalcidoidea	Eulophidae	Aprostocetus	sp.	KR796952.1
Hymenoptera	Chalcidoidea	Eulophidae	Aprostocetus	sp.	KR797184.1
Hymenoptera	Chalcidoidea	Eulophidae	Aprostocetus	sp.	KR799930.1
Hymenoptera	Chalcidoidea	Eulophidae	Aprostocetus	sp.	KR800253.1
Hymenoptera	Chalcidoidea	Eulophidae	Aprostocetus	sp.	KR801302.1
Hymenoptera	Chalcidoidea	Eulophidae	Aprostocetus	sp.	KR801338.1
Hymenoptera	Chalcidoidea	Eulophidae	Aprostocetus	sp.	KR802532.1
Hymenoptera	Chalcidoidea	Eulophidae	Aprostocetus	sp.	KR807093.1
Hymenoptera	Chalcidoidea	Eulophidae	Aprostocetus	sp.	KR807236.1
Hymenoptera	Chalcidoidea	Eulophidae	Aprostocetus	sp.	KR808375.1
Hymenoptera	Chalcidoidea	Eulophidae	Aprostocetus	sp.	KR875101.1
Hymenoptera	Chalcidoidea	Eulophidae	Aprostocetus	sp.	KR875186.1
Hymenoptera	Chalcidoidea	Eulophidae	Aprostocetus	sp.	KR875809.1
Hymenoptera	Chalcidoidea	Eulophidae	Aprostocetus	sp.	KR875987.1
Hymenoptera	Chalcidoidea	Eulophidae	Aprostocetus	sp.	KR877305.1
Hymenoptera	Chalcidoidea	Eulophidae	Aprostocetus	sp.	KR877406.1
Hymenoptera	Chalcidoidea	Eulophidae	Aprostocetus	sp.	KR878991.1
Hymenoptera	Chalcidoidea	Eulophidae	Aprostocetus	sp.	KR879923.1
Hymenoptera	Chalcidoidea	Eulophidae	Aprostocetus	sp.	KR880676.1
Hymenoptera	Chalcidoidea	Eulophidae	Aprostocetus	sp.	KR883840.1
Hymenoptera	Chalcidoidea	Eulophidae	Aprostocetus	sp.	KR884483.1
Hymenoptera	Chalcidoidea	Eulophidae	Aprostocetus	sp.	KR884611.1
Hymenoptera	Chalcidoidea	Eulophidae	Aprostocetus	sp.	KR885083.1
Hymenoptera	Chalcidoidea	Eulophidae	Aprostocetus	sp.	KR886861.1
Hymenoptera	Chalcidoidea	Eulophidae	Aprostocetus	sp.	KR886986.1
Hymenoptera	Chalcidoidea	Eulophidae	Aprostocetus	sp.	KR887956.1
Hymenoptera	Chalcidoidea	Eulophidae	Aprostocetus	sp.	KR888508.1

Hymenoptera	Chalcidoidea	Eulophidae	Aprostocetus	sp.	KR888906.1
Hymenoptera	Chalcidoidea	Eulophidae	Aprostocetus	sp.	KR889230.1
Hymenoptera	Chalcidoidea	Eulophidae	Aprostocetus	sp.	KR889432.1
Hymenoptera	Chalcidoidea	Eulophidae	Aprostocetus	sp.	KR889564.1
Hymenoptera	Chalcidoidea	Eulophidae	Aprostocetus	sp.	KR889687.1
Hymenoptera	Chalcidoidea	Eulophidae	Aprostocetus	sp.	KR890074.1
Hymenoptera	Chalcidoidea	Eulophidae	Aprostocetus	sp.	KR890361.1
Hymenoptera	Chalcidoidea	Eulophidae	Aprostocetus	sp.	KR890405.1
Hymenoptera	Chalcidoidea	Eulophidae	Aprostocetus	sp.	KR891231.1
Hymenoptera	Chalcidoidea	Eulophidae	Aprostocetus	sp.	KR891404.1
Hymenoptera	Chalcidoidea	Eulophidae	Aprostocetus	sp.	KR891491.1
Hymenoptera	Chalcidoidea	Eulophidae	Aprostocetus	sp.	KR891735.1
Hymenoptera	Chalcidoidea	Eulophidae	Aprostocetus	sp.	KR893668.1
Hymenoptera	Chalcidoidea	Eulophidae	Aprostocetus	sp.	KR895073.1
Hymenoptera	Chalcidoidea	Eulophidae	Aprostocetus	sp.	KR895481.1
Hymenoptera	Chalcidoidea	Eulophidae	Aprostocetus	sp.	KR896707.1
Hymenoptera	Chalcidoidea	Eulophidae	Aprostocetus	sp.	KR897127.1
Hymenoptera	Chalcidoidea	Eulophidae	Aprostocetus	sp.	KR897707.1
Hymenoptera	Chalcidoidea	Eulophidae	Aprostocetus	sp.	KR898756.1
Hymenoptera	Chalcidoidea	Eulophidae	Aprostocetus	sp.	KR899196.1
Hymenoptera	Chalcidoidea	Eulophidae	Aprostocetus	sp.	KR900723.1
Hymenoptera	Chalcidoidea	Eulophidae	Aprostocetus	sp.	KR928371.1
Hymenoptera	Chalcidoidea	Eulophidae	Aprostocetus	sp.	KR929263.1
Hymenoptera	Chalcidoidea	Eulophidae	Aprostocetus	sp.	KR929708.1
Hymenoptera	Chalcidoidea	Eulophidae	Aprostocetus	sp.	KR932821.1
Hymenoptera	Chalcidoidea	Eulophidae	Aprostocetus	sp.	KX051686.1
Hymenoptera	Chalcidoidea	Eulophidae	Aprostocetus	sp.	MF898668.1
Hymenoptera	Chalcidoidea	Eulophidae	Aprostocetus	sp.	MF898671.1
Hymenoptera	Chalcidoidea	Eulophidae	Aprostocetus	sp.	MF899060.1
Hymenoptera	Chalcidoidea	Eulophidae	Aprostocetus	sp.	MF899187.1
Hymenoptera	Chalcidoidea	Eulophidae	Aprostocetus	sp.	MF900914.1
Hymenoptera	Chalcidoidea	Eulophidae	Aprostocetus	sp.	MF903315.1
Hymenoptera	Chalcidoidea	Eulophidae	Aprostocetus	sp.	MF903559.1
Hymenoptera	Chalcidoidea	Eulophidae	Aprostocetus	sp.	MF905077.1
Hymenoptera	Chalcidoidea	Eulophidae	Aprostocetus	sp.	MF906187.1
Hymenoptera	Chalcidoidea	Eulophidae	Aprostocetus	sp.	MF906206.1
Hymenoptera	Chalcidoidea	Eulophidae	Aprostocetus	sp.	MF906727.1
Hymenoptera	Chalcidoidea	Eulophidae	Aprostocetus	sp.	MG334935.1
Hymenoptera	Chalcidoidea	Eulophidae	Aprostocetus	sp.	MG336503.1
Hymenoptera	Chalcidoidea	Eulophidae	Aprostocetus	sp.	MG339338.1
Hymenoptera	Chalcidoidea	Eulophidae	Aprostocetus	sp.	MG340083.1
Hymenoptera	Chalcidoidea	Eulophidae	Aprostocetus	sp.	MG340410.1
Hymenoptera	Chalcidoidea	Eulophidae	Aprostocetus	sp.	MG342134.1
Hymenoptera	Chalcidoidea	Eulophidae	Aprostocetus	sp.	MG342294.1
Hymenoptera	Chalcidoidea	Eulophidae	Aprostocetus	sp.	MG343177.1
Hymenoptera	Chalcidoidea	Eulophidae	Aprostocetus	sp.	MG343873.1
Hymenoptera	Chalcidoidea	Eulophidae	Aprostocetus	sp.	MG344105.1
Hymenoptera	Chalcidoidea	Eulophidae	Aprostocetus	sp.	MG480644.1
Hymenoptera	Chalcidoidea	Eulophidae	Aprostocetus	torquentis	MG836491.1
Hymenoptera	Chalcidoidea	Eulophidae	Aprostocetus	westwoodii	MG836496.1
Hymenoptera	Chalcidoidea	Eulophidae	Asecodes	erxias	MG836471.1
Hymenoptera	Chalcidoidea	Eulophidae	Asecodes	erxias	MG836472.1
Hymenoptera	Chalcidoidea	Eulophidae	Asecodes	galerucae	MG836474.1
Hymenoptera	Chalcidoidea	Eulophidae	Asecodes	lucens	MG836498.1

Hymenoptera	Chalcidoidea	Eulophidae	Asecodes	parviclava	KC808621.1
Hymenoptera	Chalcidoidea	Eulophidae	Asecodes	parviclava	MG836475.1
Hymenoptera	Chalcidoidea	Eulophidae	Asecodes	sp.	HM365029.1
Hymenoptera	Chalcidoidea	Eulophidae	Asecodes	sp.	KR874483.1
Hymenoptera	Chalcidoidea	Eulophidae	Asecodes	sp.	KR877790.1
Hymenoptera	Chalcidoidea	Eulophidae	Astichus	sp.	HM365048.1
Hymenoptera	Chalcidoidea	Eulophidae	Aulogymnus	arsames	MZ633275.1
Hymenoptera	Chalcidoidea	Eulophidae	Aulogymnus	sp.	HM365054.1
Hymenoptera	Chalcidoidea	Eulophidae	Baryscapus	coerulescens	HQ106675.1
Hymenoptera	Chalcidoidea	Eulophidae	Baryscapus	coerulescens	HQ106676.1
Hymenoptera	Chalcidoidea	Eulophidae	Baryscapus	coerulescens	HQ106677.1
Hymenoptera	Chalcidoidea	Eulophidae	Baryscapus	coerulescens	HQ106678.1
Hymenoptera	Chalcidoidea	Eulophidae	Baryscapus	coerulescens	HQ106679.1
Hymenoptera	Chalcidoidea	Eulophidae	Baryscapus	coerulescens	HQ106680.1
Hymenoptera	Chalcidoidea	Eulophidae	Baryscapus	coerulescens	HQ106681.1
Hymenoptera	Chalcidoidea	Eulophidae	Baryscapus	coerulescens	HQ106682.1
Hymenoptera	Chalcidoidea	Eulophidae	Baryscapus	coerulescens	HQ106683.1
Hymenoptera	Chalcidoidea	Eulophidae	Baryscapus	coerulescens	HQ106684.1
Hymenoptera	Chalcidoidea	Eulophidae	Baryscapus	coerulescens	HQ106685.1
Hymenoptera	Chalcidoidea	Eulophidae	Baryscapus	coerulescens	HQ106686.1
Hymenoptera	Chalcidoidea	Eulophidae	Baryscapus	coerulescens	HQ106687.1
Hymenoptera	Chalcidoidea	Eulophidae	Baryscapus	coerulescens	HQ106689.1
Hymenoptera	Chalcidoidea	Eulophidae	Baryscapus	coerulescens	HQ106692.1
Hymenoptera	Chalcidoidea	Eulophidae	Baryscapus	coerulescens	HQ106693.1
Hymenoptera	Chalcidoidea	Eulophidae	Baryscapus	coerulescens	HQ106694.1
Hymenoptera	Chalcidoidea	Eulophidae	Baryscapus	coerulescens	HQ106695.1
Hymenoptera	Chalcidoidea	Eulophidae	Baryscapus	coerulescens	HQ106696.1
Hymenoptera	Chalcidoidea	Eulophidae	Baryscapus	coerulescens	HQ106698.1
Hymenoptera	Chalcidoidea	Eulophidae	Baryscapus	coerulescens	HQ106699.1
Hymenoptera	Chalcidoidea	Eulophidae	Baryscapus	coerulescens	HQ106700.1
Hymenoptera	Chalcidoidea	Eulophidae	Baryscapus	coerulescens	HQ106702.1
Hymenoptera	Chalcidoidea	Eulophidae	Baryscapus	coerulescens	HQ106703.1
Hymenoptera	Chalcidoidea	Eulophidae	Baryscapus	coerulescens	HQ106704.1
Hymenoptera	Chalcidoidea	Eulophidae	Baryscapus	coerulescens	HQ106705.1
Hymenoptera	Chalcidoidea	Eulophidae	Baryscapus	coerulescens	HQ106707.1
Hymenoptera	Chalcidoidea	Eulophidae	Baryscapus	coerulescens	HQ106708.1
Hymenoptera	Chalcidoidea	Eulophidae	Baryscapus	coerulescens	HQ106710.1
Hymenoptera	Chalcidoidea	Eulophidae	Baryscapus	coerulescens	HQ106711.1
Hymenoptera	Chalcidoidea	Eulophidae	Baryscapus	coerulescens	HQ106713.1
Hymenoptera	Chalcidoidea	Eulophidae	Baryscapus	coerulescens	HQ106714.1
Hymenoptera	Chalcidoidea	Eulophidae	Baryscapus	coerulescens	HQ106715.1
Hymenoptera	Chalcidoidea	Eulophidae	Baryscapus	coerulescens	HQ106716.1
Hymenoptera	Chalcidoidea	Eulophidae	Baryscapus	coerulescens	HQ106717.1
Hymenoptera	Chalcidoidea	Eulophidae	Baryscapus	coerulescens	HQ106718.1
Hymenoptera	Chalcidoidea	Eulophidae	Baryscapus	coerulescens	HQ106719.1
Hymenoptera	Chalcidoidea	Eulophidae	Baryscapus	coerulescens	HQ106721.1
Hymenoptera	Chalcidoidea	Eulophidae	Baryscapus	coerulescens	HQ106722.1
Hymenoptera	Chalcidoidea	Eulophidae	Baryscapus	coerulescens	HQ106723.1
Hymenoptera	Chalcidoidea	Eulophidae	Baryscapus	coerulescens	HQ106727.1
Hymenoptera	Chalcidoidea	Eulophidae	Baryscapus	coerulescens	HQ106728.1
Hymenoptera	Chalcidoidea	Eulophidae	Baryscapus	coerulescens	HQ106730.1
Hymenoptera	Chalcidoidea	Eulophidae	Baryscapus	coerulescens	HQ106731.1
Hymenoptera	Chalcidoidea	Eulophidae	Baryscapus	coerulescens	HQ106732.1
Hymenoptera	Chalcidoidea	Eulophidae	Baryscapus	coerulescens	HQ106734.1

Hymenoptera	Chalcidoidea	Eulophidae	Baryscapus	coerulescens	HQ106737.1
Hymenoptera	Chalcidoidea	Eulophidae	Baryscapus	coerulescens	HQ106738.1
Hymenoptera	Chalcidoidea	Eulophidae	Baryscapus	coerulescens	HQ106739.1
Hymenoptera	Chalcidoidea	Eulophidae	Baryscapus	coerulescens	HQ106742.1
Hymenoptera	Chalcidoidea	Eulophidae	Baryscapus	coerulescens	HQ106743.1
Hymenoptera	Chalcidoidea	Eulophidae	Baryscapus	coerulescens	HQ106745.1
Hymenoptera	Chalcidoidea	Eulophidae	Baryscapus	coerulescens	HQ106746.1
Hymenoptera	Chalcidoidea	Eulophidae	Baryscapus	coerulescens	HQ106747.1
Hymenoptera	Chalcidoidea	Eulophidae	Baryscapus	coerulescens	HQ106748.1
Hymenoptera	Chalcidoidea	Eulophidae	Baryscapus	coerulescens	HQ106749.1
Hymenoptera	Chalcidoidea	Eulophidae	Baryscapus	coerulescens	HQ106750.1
Hymenoptera	Chalcidoidea	Eulophidae	Baryscapus	coerulescens	HQ106751.1
Hymenoptera	Chalcidoidea	Eulophidae	Baryscapus	coerulescens	HQ106752.1
Hymenoptera	Chalcidoidea	Eulophidae	Baryscapus	coerulescens	HQ106753.1
Hymenoptera	Chalcidoidea	Eulophidae	Baryscapus	coerulescens	HQ106754.1
Hymenoptera	Chalcidoidea	Eulophidae	Baryscapus	coerulescens	HQ106755.1
Hymenoptera	Chalcidoidea	Eulophidae	Baryscapus	coerulescens	HQ106756.1
Hymenoptera	Chalcidoidea	Eulophidae	Baryscapus	coerulescens	KR787811.1
Hymenoptera	Chalcidoidea	Eulophidae	Baryscapus	coerulescens	KR802711.1
Hymenoptera	Chalcidoidea	Eulophidae	Baryscapus	coerulescens	KR897115.1
Hymenoptera	Chalcidoidea	Eulophidae	Baryscapus	pallidae	MZ633427.1
Hymenoptera	Chalcidoidea	Eulophidae	Baryscapus	silvestrii	KP233995.1
Hymenoptera	Chalcidoidea	Eulophidae	Baryscapus	sp.	HM374765.1
Hymenoptera	Chalcidoidea	Eulophidae	Baryscapus	sp.	HQ106759.1
Hymenoptera	Chalcidoidea	Eulophidae	Baryscapus	sp.	HQ106760.1
Hymenoptera	Chalcidoidea	Eulophidae	Baryscapus	sp.	HQ106761.1
Hymenoptera	Chalcidoidea	Eulophidae	Baryscapus	sp.	HQ106762.1
Hymenoptera	Chalcidoidea	Eulophidae	Baryscapus	sp.	HQ106763.1
Hymenoptera	Chalcidoidea	Eulophidae	Baryscapus	sp.	HQ106764.1
Hymenoptera	Chalcidoidea	Eulophidae	Baryscapus	sp.	HQ106765.1
Hymenoptera	Chalcidoidea	Eulophidae	Baryscapus	sp.	HQ106766.1
Hymenoptera	Chalcidoidea	Eulophidae	Baryscapus	sp.	HQ106767.1
Hymenoptera	Chalcidoidea	Eulophidae	Baryscapus	sp.	HQ106768.1
Hymenoptera	Chalcidoidea	Eulophidae	Baryscapus	sp.	HQ106769.1
Hymenoptera	Chalcidoidea	Eulophidae	Baryscapus	sp.	HQ106770.1
Hymenoptera	Chalcidoidea	Eulophidae	Baryscapus	sp.	HQ106771.1
Hymenoptera	Chalcidoidea	Eulophidae	Baryscapus	sp.	HQ106772.1
Hymenoptera	Chalcidoidea	Eulophidae	Baryscapus	sp.	HQ106773.1
Hymenoptera	Chalcidoidea	Eulophidae	Baryscapus	sp.	HQ106774.1
Hymenoptera	Chalcidoidea	Eulophidae	Baryscapus	sp.	HQ106775.1
Hymenoptera	Chalcidoidea	Eulophidae	Baryscapus	sp.	HQ106776.1
Hymenoptera	Chalcidoidea	Eulophidae	Baryscapus	sp.	HQ106777.1
Hymenoptera	Chalcidoidea	Eulophidae	Baryscapus	sp.	HQ106778.1
Hymenoptera	Chalcidoidea	Eulophidae	Baryscapus	sp.	HQ552513.1
Hymenoptera	Chalcidoidea	Eulophidae	Baryscapus	sp.	HQ929587.1
Hymenoptera	Chalcidoidea	Eulophidae	Baryscapus	sp.	JN307278.1
Hymenoptera	Chalcidoidea	Eulophidae	Baryscapus	sp.	KF444811.1
Hymenoptera	Chalcidoidea	Eulophidae	Baryscapus	sp.	KR409851.1
Hymenoptera	Chalcidoidea	Eulophidae	Baryscapus	sp.	KR787905.1
Hymenoptera	Chalcidoidea	Eulophidae	Baryscapus	sp.	KR789417.1
Hymenoptera	Chalcidoidea	Eulophidae	Baryscapus	sp.	KR789671.1
Hymenoptera	Chalcidoidea	Eulophidae	Baryscapus	sp.	KR790294.1
Hymenoptera	Chalcidoidea	Eulophidae	Baryscapus	sp.	KR793482.1
Hymenoptera	Chalcidoidea	Eulophidae	Baryscapus	sp.	KR795688.1

Hymenoptera	Chalcidoidea	Eulophidae	Baryscapus	sp.	KR795816.1
Hymenoptera	Chalcidoidea	Eulophidae	Baryscapus	sp.	KR798895.1
Hymenoptera	Chalcidoidea	Eulophidae	Baryscapus	sp.	KR799273.1
Hymenoptera	Chalcidoidea	Eulophidae	Baryscapus	sp.	KR804069.1
Hymenoptera	Chalcidoidea	Eulophidae	Baryscapus	sp.	KR805531.1
Hymenoptera	Chalcidoidea	Eulophidae	Baryscapus	sp.	KR808485.1
Hymenoptera	Chalcidoidea	Eulophidae	Baryscapus	sp.	KR808849.1
Hymenoptera	Chalcidoidea	Eulophidae	Baryscapus	sp.	KR876389.1
Hymenoptera	Chalcidoidea	Eulophidae	Baryscapus	sp.	KR880001.1
Hymenoptera	Chalcidoidea	Eulophidae	Baryscapus	sp.	KR885638.1
Hymenoptera	Chalcidoidea	Eulophidae	Baryscapus	sp.	KR887127.1
Hymenoptera	Chalcidoidea	Eulophidae	Baryscapus	sp.	KR890403.1
Hymenoptera	Chalcidoidea	Eulophidae	Baryscapus	sp.	KR890622.1
Hymenoptera	Chalcidoidea	Eulophidae	Baryscapus	sp.	KR890879.1
Hymenoptera	Chalcidoidea	Eulophidae	Baryscapus	sp.	KR894016.1
Hymenoptera	Chalcidoidea	Eulophidae	Baryscapus	sp.	KR895309.1
Hymenoptera	Chalcidoidea	Eulophidae	Baryscapus	sp.	KR895826.1
Hymenoptera	Chalcidoidea	Eulophidae	Baryscapus	sp.	KR897505.1
Hymenoptera	Chalcidoidea	Eulophidae	Baryscapus	sp.	KR898418.1
Hymenoptera	Chalcidoidea	Eulophidae	Baryscapus	sp.	KR931737.1
Hymenoptera	Chalcidoidea	Eulophidae	Baryscapus	sp.	MF900016.1
Hymenoptera	Chalcidoidea	Eulophidae	Baryscapus	sp.	MF900286.1
Hymenoptera	Chalcidoidea	Eulophidae	Baryscapus	sp.	MF900355.1
Hymenoptera	Chalcidoidea	Eulophidae	Baryscapus	sp.	MF903262.1
Hymenoptera	Chalcidoidea	Eulophidae	Baryscapus	sp.	MF903721.1
Hymenoptera	Chalcidoidea	Eulophidae	Baryscapus	sp.	MF907517.1
Hymenoptera	Chalcidoidea	Eulophidae	Baryscapus	sp.	MG335219.1
Hymenoptera	Chalcidoidea	Eulophidae	Baryscapus	sp.	MG336911.1
Hymenoptera	Chalcidoidea	Eulophidae	Baryscapus	sp.	MG337854.1
Hymenoptera	Chalcidoidea	Eulophidae	Baryscapus	sp.	MG444531.1
Hymenoptera	Chalcidoidea	Eulophidae	Baryscapus	sp.	MG444999.1
Hymenoptera	Chalcidoidea	Eulophidae	Bellerus	sp.	HM365049.1
Hymenoptera	Chalcidoidea	Eulophidae	Ceranisus	menes	HM365037.1
Hymenoptera	Chalcidoidea	Eulophidae	Chouioia	cunea	MN011551.1
Hymenoptera	Chalcidoidea	Eulophidae	Chrysocharis	assis	MZ629216.1
Hymenoptera	Chalcidoidea	Eulophidae	Chrysocharis	clarkae	MG836431.1
Hymenoptera	Chalcidoidea	Eulophidae	Chrysocharis	clarkae	MG836449.1
Hymenoptera	Chalcidoidea	Eulophidae	Chrysocharis	clarkae	MG836452.1
Hymenoptera	Chalcidoidea	Eulophidae	Chrysocharis	clarkae	MG836453.1
Hymenoptera	Chalcidoidea	Eulophidae	Chrysocharis	eurynota	MZ656264.1
Hymenoptera	Chalcidoidea	Eulophidae	Chrysocharis	eurynota	MZ656406.1
Hymenoptera	Chalcidoidea	Eulophidae	Chrysocharis	laomedon	MZ630677.1
Hymenoptera	Chalcidoidea	Eulophidae	Chrysocharis	laomedon	MZ630787.1
Hymenoptera	Chalcidoidea	Eulophidae	Chrysocharis	laomedon	MZ630860.1
Hymenoptera	Chalcidoidea	Eulophidae	Chrysocharis	laomedon	MZ630935.1
Hymenoptera	Chalcidoidea	Eulophidae	Chrysocharis	laomedon	MZ633233.1
Hymenoptera	Chalcidoidea	Eulophidae	Chrysocharis	nautius	MZ629393.1
Hymenoptera	Chalcidoidea	Eulophidae	Chrysocharis	nautius	MZ630435.1
Hymenoptera	Chalcidoidea	Eulophidae	Chrysocharis	nautius	MZ630836.1
Hymenoptera	Chalcidoidea	Eulophidae	Chrysocharis	nautius	MZ631709.1
Hymenoptera	Chalcidoidea	Eulophidae	Chrysocharis	nautius	MZ633396.1
Hymenoptera	Chalcidoidea	Eulophidae	Chrysocharis	pallipes	KM073131.1
Hymenoptera	Chalcidoidea	Eulophidae	Chrysocharis	pallipes	KM073132.1
Hymenoptera	Chalcidoidea	Eulophidae	Chrysocharis	pallipes	KM073136.1

Hymenoptera	Chalcidoidea	Eulophidae	Chrysocharis	pallipes	KM073137.1
Hymenoptera	Chalcidoidea	Eulophidae	Chrysocharis	pallipes	KM073139.1
Hymenoptera	Chalcidoidea	Eulophidae	Chrysocharis	pallipes	KM073140.1
Hymenoptera	Chalcidoidea	Eulophidae	Chrysocharis	pallipes	KM073143.1
Hymenoptera	Chalcidoidea	Eulophidae	Chrysocharis	pallipes	KM073145.1
Hymenoptera	Chalcidoidea	Eulophidae	Chrysocharis	pallipes	KM073146.1
Hymenoptera	Chalcidoidea	Eulophidae	Chrysocharis	pallipes	KM073147.1
Hymenoptera	Chalcidoidea	Eulophidae	Chrysocharis	pallipes	KM073149.1
Hymenoptera	Chalcidoidea	Eulophidae	Chrysocharis	pallipes	KM073150.1
Hymenoptera	Chalcidoidea	Eulophidae	Chrysocharis	pallipes	KM073151.1
Hymenoptera	Chalcidoidea	Eulophidae	Chrysocharis	pentheus	LC542885.1
Hymenoptera	Chalcidoidea	Eulophidae	Chrysocharis	pentheus	MZ631998.1
Hymenoptera	Chalcidoidea	Eulophidae	Chrysocharis	prodice	MZ629714.1
Hymenoptera	Chalcidoidea	Eulophidae	Chrysocharis	prodice	MZ630227.1
Hymenoptera	Chalcidoidea	Eulophidae	Chrysocharis	prodice	MZ630896.1
Hymenoptera	Chalcidoidea	Eulophidae	Chrysocharis	prodice	MZ631265.1
Hymenoptera	Chalcidoidea	Eulophidae	Chrysocharis	prodice	MZ632595.1
Hymenoptera	Chalcidoidea	Eulophidae	Chrysocharis	prodice	MZ633792.1
Hymenoptera	Chalcidoidea	Eulophidae	Chrysocharis	prodice	MZ658693.1
Hymenoptera	Chalcidoidea	Eulophidae	Chrysocharis	pubicornis	KM073153.1
Hymenoptera	Chalcidoidea	Eulophidae	Chrysocharis	pubicornis	KM073154.1
Hymenoptera	Chalcidoidea	Eulophidae	Chrysocharis	pubicornis	LC542886.1
Hymenoptera	Chalcidoidea	Eulophidae	Chrysocharis	pubicornis	MG336398.1
Hymenoptera	Chalcidoidea	Eulophidae	Chrysocharis	pubicornis	MG342829.1
Hymenoptera	Chalcidoidea	Eulophidae	Chrysocharis	sp.	HM365030.1
Hymenoptera	Chalcidoidea	Eulophidae	Chrysocharis	sp.	KR790845.1
Hymenoptera	Chalcidoidea	Eulophidae	Chrysocharis	sp.	MG339625.1
Hymenoptera	Chalcidoidea	Eulophidae	Chrysocharis	sp.	MZ630280.1
Hymenoptera	Chalcidoidea	Eulophidae	Chrysocharis	sp.	MZ630318.1
Hymenoptera	Chalcidoidea	Eulophidae	Chrysocharis	sp.	MZ630674.1
Hymenoptera	Chalcidoidea	Eulophidae	Chrysocharis	sp.	MZ630992.1
Hymenoptera	Chalcidoidea	Eulophidae	Chrysocharis	sp.	MZ631242.1
Hymenoptera	Chalcidoidea	Eulophidae	Chrysocharis	sp.	MZ631863.1
Hymenoptera	Chalcidoidea	Eulophidae	Chrysocharis	sp.	MZ632970.1
Hymenoptera	Chalcidoidea	Eulophidae	Chrysocharis	sp.	MZ632997.1
Hymenoptera	Chalcidoidea	Eulophidae	Chrysocharis	viridis	MG836454.1
Hymenoptera	Chalcidoidea	Eulophidae	Chrysonotomyia	germanica	HM365036.1
Hymenoptera	Chalcidoidea	Eulophidae	Chrysonotomyia	sp.	HM365031.1
Hymenoptera	Chalcidoidea	Eulophidae	Cirrospilus	diallus	MZ629429.1
Hymenoptera	Chalcidoidea	Eulophidae	Cirrospilus	diallus	MZ629494.1
Hymenoptera	Chalcidoidea	Eulophidae	Cirrospilus	diallus	MZ629540.1
Hymenoptera	Chalcidoidea	Eulophidae	Cirrospilus	diallus	MZ629705.1
Hymenoptera	Chalcidoidea	Eulophidae	Cirrospilus	diallus	MZ629750.1
Hymenoptera	Chalcidoidea	Eulophidae	Cirrospilus	diallus	MZ629832.1
Hymenoptera	Chalcidoidea	Eulophidae	Cirrospilus	diallus	MZ630478.1
Hymenoptera	Chalcidoidea	Eulophidae	Cirrospilus	diallus	MZ630840.1
Hymenoptera	Chalcidoidea	Eulophidae	Cirrospilus	diallus	MZ631173.1
Hymenoptera	Chalcidoidea	Eulophidae	Cirrospilus	diallus	MZ631402.1
Hymenoptera	Chalcidoidea	Eulophidae	Cirrospilus	diallus	MZ631429.1
Hymenoptera	Chalcidoidea	Eulophidae	Cirrospilus	diallus	MZ631695.1
Hymenoptera	Chalcidoidea	Eulophidae	Cirrospilus	diallus	MZ632136.1
Hymenoptera	Chalcidoidea	Eulophidae	Cirrospilus	diallus	MZ632775.1
Hymenoptera	Chalcidoidea	Eulophidae	Cirrospilus	diallus	MZ632921.1
Hymenoptera	Chalcidoidea	Eulophidae	Cirrospilus	diallus	MZ632966.1

Hymenoptera	Chalcidoidea	Eulophidae	Cirrospilus	diallus	MZ633224.1
Hymenoptera	Chalcidoidea	Eulophidae	Cirrospilus	diallus	MZ633697.1
Hymenoptera	Chalcidoidea	Eulophidae	Cirrospilus	lyncus	MZ629960.1
Hymenoptera	Chalcidoidea	Eulophidae	Cirrospilus	lyncus	MZ631414.1
Hymenoptera	Chalcidoidea	Eulophidae	Cirrospilus	lyncus	MZ632191.1
Hymenoptera	Chalcidoidea	Eulophidae	Cirrospilus	lyncus	MZ633900.1
Hymenoptera	Chalcidoidea	Eulophidae	Cirrospilus	pictus	MG836462.1
Hymenoptera	Chalcidoidea	Eulophidae	Cirrospilus	pictus	MG836463.1
Hymenoptera	Chalcidoidea	Eulophidae	Cirrospilus	pictus	MG836464.1
Hymenoptera	Chalcidoidea	Eulophidae	Cirrospilus	pictus	MT609876.1
Hymenoptera	Chalcidoidea	Eulophidae	Cirrospilus	pictus	MZ629929.1
Hymenoptera	Chalcidoidea	Eulophidae	Cirrospilus	sp.	MZ630941.1
Hymenoptera	Chalcidoidea	Eulophidae	Cirrospilus	sp.	MZ632411.1
Hymenoptera	Chalcidoidea	Eulophidae	Cirrospilus	sp.	MZ633646.1
Hymenoptera	Chalcidoidea	Eulophidae	Cirrospilus	variegatus	MG836465.1
Hymenoptera	Chalcidoidea	Eulophidae	Cirrospilus	vittatus	MG836459.1
Hymenoptera	Chalcidoidea	Eulophidae	Cirrospilus	vittatus	MG836460.1
Hymenoptera	Chalcidoidea	Eulophidae	Cirrospilus	vittatus	MG836461.1
Hymenoptera	Chalcidoidea	Eulophidae	Closterocerus	chamaeleon	MG836428.1
Hymenoptera	Chalcidoidea	Eulophidae	Closterocerus	insignis	MG836427.1
Hymenoptera	Chalcidoidea	Eulophidae	Closterocerus	tau	HM365035.1
Hymenoptera	Chalcidoidea	Eulophidae	Closterocerus	trifasciatus	HM365034.1
Hymenoptera	Chalcidoidea	Eulophidae	Closterocerus	trifasciatus	KR782952.1
Hymenoptera	Chalcidoidea	Eulophidae	Closterocerus	trifasciatus	KR784859.1
Hymenoptera	Chalcidoidea	Eulophidae	Closterocerus	trifasciatus	KR786057.1
Hymenoptera	Chalcidoidea	Eulophidae	Closterocerus	trifasciatus	KR790709.1
Hymenoptera	Chalcidoidea	Eulophidae	Closterocerus	trifasciatus	KR800508.1
Hymenoptera	Chalcidoidea	Eulophidae	Closterocerus	trifasciatus	KR802266.1
Hymenoptera	Chalcidoidea	Eulophidae	Closterocerus	trifasciatus	LC542887.1
Hymenoptera	Chalcidoidea	Eulophidae	Closterocerus	trifasciatus	MZ633378.1
Hymenoptera	Chalcidoidea	Eulophidae	Dasyomphale	sp.	HM365044.1
Hymenoptera	Chalcidoidea	Eulophidae	Derostenus	sp.	MZ630446.1
Hymenoptera	Chalcidoidea	Eulophidae	Derostenus	sp.	MZ631923.1
Hymenoptera	Chalcidoidea	Eulophidae	Derostenus	sp.	MZ632281.1
Hymenoptera	Chalcidoidea	Eulophidae	Derostenus	sp.	MZ632293.1
Hymenoptera	Chalcidoidea	Eulophidae	Derostenus	sp.	MZ633940.1
Hymenoptera	Chalcidoidea	Eulophidae	Derostenus	sp.	MZ658705.1
Hymenoptera	Chalcidoidea	Eulophidae	Diaulinopsis	arenaria	MG836436.1
Hymenoptera	Chalcidoidea	Eulophidae	Diaulinopsis	arenaria	MG836437.1
Hymenoptera	Chalcidoidea	Eulophidae	Diglyphomorpha	sp.	HQ926651.1
Hymenoptera	Chalcidoidea	Eulophidae	Diglyphus	albiscapus	LC542888.1
Hymenoptera	Chalcidoidea	Eulophidae	Diglyphus	albiscapus	MG836455.1
Hymenoptera	Chalcidoidea	Eulophidae	Diglyphus	gibbus	MG836458.1
Hymenoptera	Chalcidoidea	Eulophidae	Diglyphus	isaea	FM210148.1
Hymenoptera	Chalcidoidea	Eulophidae	Diglyphus	isaea	FM210149.1
Hymenoptera	Chalcidoidea	Eulophidae	Diglyphus	isaea	FM210150.1
Hymenoptera	Chalcidoidea	Eulophidae	Diglyphus	isaea	FM210151.1
Hymenoptera	Chalcidoidea	Eulophidae	Diglyphus	isaea	FM210152.1
Hymenoptera	Chalcidoidea	Eulophidae	Diglyphus	isaea	FM210153.1
Hymenoptera	Chalcidoidea	Eulophidae	Diglyphus	isaea	FM210154.1
Hymenoptera	Chalcidoidea	Eulophidae	Diglyphus	isaea	FM210155.1
Hymenoptera	Chalcidoidea	Eulophidae	Diglyphus	isaea	FM210156.1
Hymenoptera	Chalcidoidea	Eulophidae	Diglyphus	isaea	FM210157.1
Hymenoptera	Chalcidoidea	Eulophidae	Diglyphus	isaea	KM016074.1

Hymenoptera	Chalcidoidea	Eulophidae	Diglyphus	isaea	KM073120.1
Hymenoptera	Chalcidoidea	Eulophidae	Diglyphus	isaea	KM073121.1
Hymenoptera	Chalcidoidea	Eulophidae	Diglyphus	isaea	KM073122.1
Hymenoptera	Chalcidoidea	Eulophidae	Diglyphus	isaea	KM073123.1
Hymenoptera	Chalcidoidea	Eulophidae	Diglyphus	isaea	KM073124.1
Hymenoptera	Chalcidoidea	Eulophidae	Diglyphus	isaea	KM073125.1
Hymenoptera	Chalcidoidea	Eulophidae	Diglyphus	isaea	KM073126.1
Hymenoptera	Chalcidoidea	Eulophidae	Diglyphus	isaea	KM073127.1
Hymenoptera	Chalcidoidea	Eulophidae	Diglyphus	isaea	KM073128.1
Hymenoptera	Chalcidoidea	Eulophidae	Diglyphus	isaea	KM073129.1
Hymenoptera	Chalcidoidea	Eulophidae	Diglyphus	isaea	LC542889.1
Hymenoptera	Chalcidoidea	Eulophidae	Diglyphus	isaea	MG836457.1
Hymenoptera	Chalcidoidea	Eulophidae	Diglyphus	isaea	MG836487.1
Hymenoptera	Chalcidoidea	Eulophidae	Diglyphus	pulchripes	KR794612.1
Hymenoptera	Chalcidoidea	Eulophidae	Diglyphus	pulchripes	KR877947.1
Hymenoptera	Chalcidoidea	Eulophidae	Diglyphus	pulchripes	KR885689.1
Hymenoptera	Chalcidoidea	Eulophidae	Elachertus	cacoeciae	MN861998.1
Hymenoptera	Chalcidoidea	Eulophidae	Elachertus	isadas	MG836433.1
Hymenoptera	Chalcidoidea	Eulophidae	Elachertus	petiolifuniculus	MG836426.1
Hymenoptera	Chalcidoidea	Eulophidae	Elachertus	petiolifuniculus	MG836434.1
Hymenoptera	Chalcidoidea	Eulophidae	Elachertus	petiolifuniculus	MG836435.1
Hymenoptera	Chalcidoidea	Eulophidae	Elachertus	sp.	HQ107127.1
Hymenoptera	Chalcidoidea	Eulophidae	Elachertus	sp.	HQ107133.1
Hymenoptera	Chalcidoidea	Eulophidae	Elachertus	sp.	KR893924.1
Hymenoptera	Chalcidoidea	Eulophidae	Elachertus	sp.	MZ629220.1
Hymenoptera	Chalcidoidea	Eulophidae	Elachertus	sp.	MZ630013.1
Hymenoptera	Chalcidoidea	Eulophidae	Elachertus	sp.	MZ630192.1
Hymenoptera	Chalcidoidea	Eulophidae	Elachertus	sp.	MZ630388.1
Hymenoptera	Chalcidoidea	Eulophidae	Elachertus	sp.	MZ630509.1
Hymenoptera	Chalcidoidea	Eulophidae	Elachertus	sp.	MZ630632.1
Hymenoptera	Chalcidoidea	Eulophidae	Elasminae	sp.	KR784834.1
Hymenoptera	Chalcidoidea	Eulophidae	Elasminae	sp.	KR796315.1
Hymenoptera	Chalcidoidea	Eulophidae	Elasmus	atratus	HQ107134.1
Hymenoptera	Chalcidoidea	Eulophidae	Elasmus	atratus	HQ107136.1
Hymenoptera	Chalcidoidea	Eulophidae	Elasmus	atratus	HQ107137.1
Hymenoptera	Chalcidoidea	Eulophidae	Elasmus	atratus	HQ107138.1
Hymenoptera	Chalcidoidea	Eulophidae	Elasmus	atratus	HQ107139.1
Hymenoptera	Chalcidoidea	Eulophidae	Elasmus	atratus	HQ107140.1
Hymenoptera	Chalcidoidea	Eulophidae	Elasmus	atratus	HQ107141.1
Hymenoptera	Chalcidoidea	Eulophidae	Elasmus	atratus	HQ107142.1
Hymenoptera	Chalcidoidea	Eulophidae	Elasmus	atratus	HQ107143.1
Hymenoptera	Chalcidoidea	Eulophidae	Elasmus	atratus	HQ107144.1
Hymenoptera	Chalcidoidea	Eulophidae	Elasmus	atratus	HQ107145.1
Hymenoptera	Chalcidoidea	Eulophidae	Elasmus	atratus	HQ107146.1
Hymenoptera	Chalcidoidea	Eulophidae	Elasmus	atratus	HQ107147.1
Hymenoptera	Chalcidoidea	Eulophidae	Elasmus	atratus	HQ107148.1
Hymenoptera	Chalcidoidea	Eulophidae	Elasmus	atratus	HQ107149.1
Hymenoptera	Chalcidoidea	Eulophidae	Elasmus	atratus	HQ107150.1
Hymenoptera	Chalcidoidea	Eulophidae	Elasmus	atratus	HQ107151.1
Hymenoptera	Chalcidoidea	Eulophidae	Elasmus	atratus	HQ107152.1
Hymenoptera	Chalcidoidea	Eulophidae	Elasmus	atratus	HQ107153.1
Hymenoptera	Chalcidoidea	Eulophidae	Elasmus	atratus	HQ107154.1
Hymenoptera	Chalcidoidea	Eulophidae	Elasmus	atratus	HQ107156.1
Hymenoptera	Chalcidoidea	Eulophidae	Elasmus	atratus	HQ107157.1

Hymenoptera	Chalcidoidea	Eulophidae	Elasmus	atratus	HQ107158.1
Hymenoptera	Chalcidoidea	Eulophidae	Elasmus	atratus	HQ107159.1
Hymenoptera	Chalcidoidea	Eulophidae	Elasmus	atratus	HQ107160.1
Hymenoptera	Chalcidoidea	Eulophidae	Elasmus	atratus	HQ107161.1
Hymenoptera	Chalcidoidea	Eulophidae	Elasmus	atratus	HQ107163.1
Hymenoptera	Chalcidoidea	Eulophidae	Elasmus	atratus	HQ107164.1
Hymenoptera	Chalcidoidea	Eulophidae	Elasmus	atratus	HQ107165.1
Hymenoptera	Chalcidoidea	Eulophidae	Elasmus	atratus	HQ107166.1
Hymenoptera	Chalcidoidea	Eulophidae	Elasmus	atratus	HQ107167.1
Hymenoptera	Chalcidoidea	Eulophidae	Elasmus	atratus	HQ107169.1
Hymenoptera	Chalcidoidea	Eulophidae	Elasmus	atratus	HQ107170.1
Hymenoptera	Chalcidoidea	Eulophidae	Elasmus	atratus	HQ107171.1
Hymenoptera	Chalcidoidea	Eulophidae	Elasmus	atratus	HQ107172.1
Hymenoptera	Chalcidoidea	Eulophidae	Elasmus	atratus	HQ107173.1
Hymenoptera	Chalcidoidea	Eulophidae	Elasmus	atratus	HQ107175.1
Hymenoptera	Chalcidoidea	Eulophidae	Elasmus	atratus	HQ107177.1
Hymenoptera	Chalcidoidea	Eulophidae	Elasmus	atratus	HQ107179.1
Hymenoptera	Chalcidoidea	Eulophidae	Elasmus	atratus	HQ107180.1
Hymenoptera	Chalcidoidea	Eulophidae	Elasmus	atratus	HQ107182.1
Hymenoptera	Chalcidoidea	Eulophidae	Elasmus	atratus	HQ107183.1
Hymenoptera	Chalcidoidea	Eulophidae	Elasmus	atratus	KR794054.1
Hymenoptera	Chalcidoidea	Eulophidae	Elasmus	atratus	KR884930.1
Hymenoptera	Chalcidoidea	Eulophidae	Elasmus	sp.	KR410018.1
Hymenoptera	Chalcidoidea	Eulophidae	Emersonella	lemae	KF444814.1
Hymenoptera	Chalcidoidea	Eulophidae	Entedon	intumescens	MG836451.1
Hymenoptera	Chalcidoidea	Eulophidae	Entedon	pallicrus	MG836448.1
Hymenoptera	Chalcidoidea	Eulophidae	Entedon	pumilae	MG836450.1
Hymenoptera	Chalcidoidea	Eulophidae	Entedon	sp.	HM365041.1
Hymenoptera	Chalcidoidea	Eulophidae	Entedon	sp.	JN292673.1
Hymenoptera	Chalcidoidea	Eulophidae	Entedon	sp.	KF936646.1
Hymenoptera	Chalcidoidea	Eulophidae	Entedon	sp.	KR795642.1
Hymenoptera	Chalcidoidea	Eulophidae	Entedon	sp.	KR799118.1
Hymenoptera	Chalcidoidea	Eulophidae	Entedon	sp.	KR801012.1
Hymenoptera	Chalcidoidea	Eulophidae	Entedon	sp.	KR801214.1
Hymenoptera	Chalcidoidea	Eulophidae	Entedon	sp.	KR877111.1
Hymenoptera	Chalcidoidea	Eulophidae	Entedon	sp.	MF904214.1
Hymenoptera	Chalcidoidea	Eulophidae	Entedon	sp.	MG339040.1
Hymenoptera	Chalcidoidea	Eulophidae	Entedon	sp.	MG340095.1
Hymenoptera	Chalcidoidea	Eulophidae	Entedon	sp.	MG341109.1
Hymenoptera	Chalcidoidea	Eulophidae	Entedon	sp.	MG483876.1
Hymenoptera	Chalcidoidea	Eulophidae	Entedoninae	sp.	HM883313.1
Hymenoptera	Chalcidoidea	Eulophidae	Entedoninae	sp.	KR406321.1
Hymenoptera	Chalcidoidea	Eulophidae	Entedoninae	sp.	KR407039.1
Hymenoptera	Chalcidoidea	Eulophidae	Entedoninae	sp.	KR410569.1
Hymenoptera	Chalcidoidea	Eulophidae	Entedoninae	sp.	KR410732.1
Hymenoptera	Chalcidoidea	Eulophidae	Entedoninae	sp.	KR414030.1
Hymenoptera	Chalcidoidea	Eulophidae	Entedoninae	sp.	KR417332.1
Hymenoptera	Chalcidoidea	Eulophidae	Entedoninae	sp.	KR417689.1
Hymenoptera	Chalcidoidea	Eulophidae	Entedoninae	sp.	KR422145.1
Hymenoptera	Chalcidoidea	Eulophidae	Entedoninae	sp.	KR787801.1
Hymenoptera	Chalcidoidea	Eulophidae	Entedoninae	sp.	KR790319.1
Hymenoptera	Chalcidoidea	Eulophidae	Entedoninae	sp.	KR794640.1
Hymenoptera	Chalcidoidea	Eulophidae	Entedoninae	sp.	KR796440.1
Hymenoptera	Chalcidoidea	Eulophidae	Entedoninae	sp.	KR799718.1

Hymenoptera	Chalcidoidea	Eulophidae	Entedoninae	sp.	KR800387.1
Hymenoptera	Chalcidoidea	Eulophidae	Entedoninae	sp.	KR803536.1
Hymenoptera	Chalcidoidea	Eulophidae	Entedoninae	sp.	KR805478.1
Hymenoptera	Chalcidoidea	Eulophidae	Entedoninae	sp.	KR806787.1
Hymenoptera	Chalcidoidea	Eulophidae	Entedoninae	sp.	KR807761.1
Hymenoptera	Chalcidoidea	Eulophidae	Entedoninae	sp.	KR807774.1
Hymenoptera	Chalcidoidea	Eulophidae	Entedoninae	sp.	KR891664.1
Hymenoptera	Chalcidoidea	Eulophidae	Entedoninae	sp.	KT706918.1
Hymenoptera	Chalcidoidea	Eulophidae	Entedoninae	sp.	MF903807.1
Hymenoptera	Chalcidoidea	Eulophidae	Entedoninae	sp.	MF905328.1
Hymenoptera	Chalcidoidea	Eulophidae	Entedoninae	sp.	MG336434.1
Hymenoptera	Chalcidoidea	Eulophidae	Entedoninae	sp.	MG338311.1
Hymenoptera	Chalcidoidea	Eulophidae	Entedoninae	sp.	MG339760.1
Hymenoptera	Chalcidoidea	Eulophidae	Entedoninae	sp.	MG340728.1
Hymenoptera	Chalcidoidea	Eulophidae	Entedoninae	sp.	MZ657756.1
Hymenoptera	Chalcidoidea	Eulophidae	Entedononecremnus	sp.	HM365045.1
Hymenoptera	Chalcidoidea	Eulophidae	Entiinae	sp.	GU675352.1
Hymenoptera	Chalcidoidea	Eulophidae	Entiinae	sp.	GU675359.1
Hymenoptera	Chalcidoidea	Eulophidae	Entiinae	sp.	KR803084.1
Hymenoptera	Chalcidoidea	Eulophidae	Euderus	albitarsis	MG836466.1
Hymenoptera	Chalcidoidea	Eulophidae	Euderus	albitarsis	MG836467.1
Hymenoptera	Chalcidoidea	Eulophidae	Euderus	albitarsis	MG836468.1
Hymenoptera	Chalcidoidea	Eulophidae	Euderus	albitarsis	MG836469.1
Hymenoptera	Chalcidoidea	Eulophidae	Euderus	cushmani	MG836470.1
Hymenoptera	Chalcidoidea	Eulophidae	Euderus	set	MK295000.1
Hymenoptera	Chalcidoidea	Eulophidae	Euderus	set	MK295009.1
Hymenoptera	Chalcidoidea	Eulophidae	Euderus	sp.	HM365047.1
Hymenoptera	Chalcidoidea	Eulophidae	Euderus	sp.	HM374819.1
Hymenoptera	Chalcidoidea	Eulophidae	Euderus	sp.	HM414503.1
Hymenoptera	Chalcidoidea	Eulophidae	Euderus	sp.	KR791285.1
Hymenoptera	Chalcidoidea	Eulophidae	Euderus	sp.	KR792605.1
Hymenoptera	Chalcidoidea	Eulophidae	Euderus	sp.	KR800623.1
Hymenoptera	Chalcidoidea	Eulophidae	Euderus	sp.	KR802660.1
Hymenoptera	Chalcidoidea	Eulophidae	Euderus	sp.	MG339457.1
Hymenoptera	Chalcidoidea	Eulophidae	Eulophidae	sp.	HM374668.1
Hymenoptera	Chalcidoidea	Eulophidae	Eulophidae	sp.	HM374804.1
Hymenoptera	Chalcidoidea	Eulophidae	Eulophidae	sp.	HM374815.1
Hymenoptera	Chalcidoidea	Eulophidae	Eulophidae	sp.	HM374829.1
Hymenoptera	Chalcidoidea	Eulophidae	Eulophidae	sp.	HM374834.1
Hymenoptera	Chalcidoidea	Eulophidae	Eulophidae	sp.	HM374835.1
Hymenoptera	Chalcidoidea	Eulophidae	Eulophidae	sp.	HM414508.1
Hymenoptera	Chalcidoidea	Eulophidae	Eulophidae	sp.	HM414518.1
Hymenoptera	Chalcidoidea	Eulophidae	Eulophidae	sp.	HM414608.1
Hymenoptera	Chalcidoidea	Eulophidae	Eulophidae	sp.	HM423358.1
Hymenoptera	Chalcidoidea	Eulophidae	Eulophidae	sp.	HQ552650.1
Hymenoptera	Chalcidoidea	Eulophidae	Eulophidae	sp.	HQ928898.1
Hymenoptera	Chalcidoidea	Eulophidae	Eulophidae	sp.	HQ929285.1
Hymenoptera	Chalcidoidea	Eulophidae	Eulophidae	sp.	HQ929579.1
Hymenoptera	Chalcidoidea	Eulophidae	Eulophidae	sp.	HQ929800.1
Hymenoptera	Chalcidoidea	Eulophidae	Eulophidae	sp.	HQ930309.1
Hymenoptera	Chalcidoidea	Eulophidae	Eulophidae	sp.	HQ930315.1
Hymenoptera	Chalcidoidea	Eulophidae	Eulophidae	sp.	HQ930318.1
Hymenoptera	Chalcidoidea	Eulophidae	Eulophidae	sp.	HQ930356.1
Hymenoptera	Chalcidoidea	Eulophidae	Eulophidae	sp.	HQ930358.1

Hymenoptera	Chalcidoidea	Eulophidae	Eulophidae	sp.	HQ930365.1
Hymenoptera	Chalcidoidea	Eulophidae	Eulophidae	sp.	JF271349.1
Hymenoptera	Chalcidoidea	Eulophidae	Eulophidae	sp.	JF271350.1
Hymenoptera	Chalcidoidea	Eulophidae	Eulophidae	sp.	JF271352.1
Hymenoptera	Chalcidoidea	Eulophidae	Eulophidae	sp.	JF863265.1
Hymenoptera	Chalcidoidea	Eulophidae	Eulophidae	sp.	JF863275.1
Hymenoptera	Chalcidoidea	Eulophidae	Eulophidae	sp.	JN292473.1
Hymenoptera	Chalcidoidea	Eulophidae	Eulophidae	sp.	JN292896.1
Hymenoptera	Chalcidoidea	Eulophidae	Eulophidae	sp.	JN293141.1
Hymenoptera	Chalcidoidea	Eulophidae	Eulophidae	sp.	JN293249.1
Hymenoptera	Chalcidoidea	Eulophidae	Eulophidae	sp.	JN293385.1
Hymenoptera	Chalcidoidea	Eulophidae	Eulophidae	sp.	JN293386.1
Hymenoptera	Chalcidoidea	Eulophidae	Eulophidae	sp.	JN293567.1
Hymenoptera	Chalcidoidea	Eulophidae	Eulophidae	sp.	JN293575.1
Hymenoptera	Chalcidoidea	Eulophidae	Eulophidae	sp.	JN293576.1
Hymenoptera	Chalcidoidea	Eulophidae	Eulophidae	sp.	KJ083326.1
Hymenoptera	Chalcidoidea	Eulophidae	Eulophidae	sp.	KJ083443.1
Hymenoptera	Chalcidoidea	Eulophidae	Eulophidae	sp.	KJ084356.1
Hymenoptera	Chalcidoidea	Eulophidae	Eulophidae	sp.	KJ085574.1
Hymenoptera	Chalcidoidea	Eulophidae	Eulophidae	sp.	KJ086628.1
Hymenoptera	Chalcidoidea	Eulophidae	Eulophidae	sp.	KJ086783.1
Hymenoptera	Chalcidoidea	Eulophidae	Eulophidae	sp.	KJ087480.1
Hymenoptera	Chalcidoidea	Eulophidae	Eulophidae	sp.	KJ087844.1
Hymenoptera	Chalcidoidea	Eulophidae	Eulophidae	sp.	KJ088780.1
Hymenoptera	Chalcidoidea	Eulophidae	Eulophidae	sp.	KJ088787.1
Hymenoptera	Chalcidoidea	Eulophidae	Eulophidae	sp.	KJ088898.1
Hymenoptera	Chalcidoidea	Eulophidae	Eulophidae	sp.	KJ089247.1
Hymenoptera	Chalcidoidea	Eulophidae	Eulophidae	sp.	KJ089561.1
Hymenoptera	Chalcidoidea	Eulophidae	Eulophidae	sp.	KJ091835.1
Hymenoptera	Chalcidoidea	Eulophidae	Eulophidae	sp.	KJ092087.1
Hymenoptera	Chalcidoidea	Eulophidae	Eulophidae	sp.	KJ092571.1
Hymenoptera	Chalcidoidea	Eulophidae	Eulophidae	sp.	KJ092625.1
Hymenoptera	Chalcidoidea	Eulophidae	Eulophidae	sp.	KJ092733.1
Hymenoptera	Chalcidoidea	Eulophidae	Eulophidae	sp.	KJ093039.1
Hymenoptera	Chalcidoidea	Eulophidae	Eulophidae	sp.	KJ164306.1
Hymenoptera	Chalcidoidea	Eulophidae	Eulophidae	sp.	KJ165710.1
Hymenoptera	Chalcidoidea	Eulophidae	Eulophidae	sp.	KJ208002.1
Hymenoptera	Chalcidoidea	Eulophidae	Eulophidae	sp.	KJ208370.1
Hymenoptera	Chalcidoidea	Eulophidae	Eulophidae	sp.	KJ208448.1
Hymenoptera	Chalcidoidea	Eulophidae	Eulophidae	sp.	KJ208521.1
Hymenoptera	Chalcidoidea	Eulophidae	Eulophidae	sp.	KJ208737.1
Hymenoptera	Chalcidoidea	Eulophidae	Eulophidae	sp.	KJ208837.1
Hymenoptera	Chalcidoidea	Eulophidae	Eulophidae	sp.	KJ208956.1
Hymenoptera	Chalcidoidea	Eulophidae	Eulophidae	sp.	KJ209137.1
Hymenoptera	Chalcidoidea	Eulophidae	Eulophidae	sp.	KJ443886.1
Hymenoptera	Chalcidoidea	Eulophidae	Eulophidae	sp.	KJ444100.1
Hymenoptera	Chalcidoidea	Eulophidae	Eulophidae	sp.	KJ445584.1
Hymenoptera	Chalcidoidea	Eulophidae	Eulophidae	sp.	KR365434.1
Hymenoptera	Chalcidoidea	Eulophidae	Eulophidae	sp.	KR367061.1
Hymenoptera	Chalcidoidea	Eulophidae	Eulophidae	sp.	KR367670.1
Hymenoptera	Chalcidoidea	Eulophidae	Eulophidae	sp.	KR368106.1
Hymenoptera	Chalcidoidea	Eulophidae	Eulophidae	sp.	KR368136.1
Hymenoptera	Chalcidoidea	Eulophidae	Eulophidae	sp.	KR368857.1
Hymenoptera	Chalcidoidea	Eulophidae	Eulophidae	sp.	KR373334.1

Hymenoptera	Chalcidoidea	Eulophidae	Eulophidae	sp.	KR373785.1
Hymenoptera	Chalcidoidea	Eulophidae	Eulophidae	sp.	KR374399.1
Hymenoptera	Chalcidoidea	Eulophidae	Eulophidae	sp.	KR374612.1
Hymenoptera	Chalcidoidea	Eulophidae	Eulophidae	sp.	KR374781.1
Hymenoptera	Chalcidoidea	Eulophidae	Eulophidae	sp.	KR401948.1
Hymenoptera	Chalcidoidea	Eulophidae	Eulophidae	sp.	KR402485.1
Hymenoptera	Chalcidoidea	Eulophidae	Eulophidae	sp.	KR402594.1
Hymenoptera	Chalcidoidea	Eulophidae	Eulophidae	sp.	KR402781.1
Hymenoptera	Chalcidoidea	Eulophidae	Eulophidae	sp.	KR402813.1
Hymenoptera	Chalcidoidea	Eulophidae	Eulophidae	sp.	KR403004.1
Hymenoptera	Chalcidoidea	Eulophidae	Eulophidae	sp.	KR403362.1
Hymenoptera	Chalcidoidea	Eulophidae	Eulophidae	sp.	KR403808.1
Hymenoptera	Chalcidoidea	Eulophidae	Eulophidae	sp.	KR404347.1
Hymenoptera	Chalcidoidea	Eulophidae	Eulophidae	sp.	KR405323.1
Hymenoptera	Chalcidoidea	Eulophidae	Eulophidae	sp.	KR405757.1
Hymenoptera	Chalcidoidea	Eulophidae	Eulophidae	sp.	KR406209.1
Hymenoptera	Chalcidoidea	Eulophidae	Eulophidae	sp.	KR406381.1
Hymenoptera	Chalcidoidea	Eulophidae	Eulophidae	sp.	KR407063.1
Hymenoptera	Chalcidoidea	Eulophidae	Eulophidae	sp.	KR407309.1
Hymenoptera	Chalcidoidea	Eulophidae	Eulophidae	sp.	KR407531.1
Hymenoptera	Chalcidoidea	Eulophidae	Eulophidae	sp.	KR407774.1
Hymenoptera	Chalcidoidea	Eulophidae	Eulophidae	sp.	KR407783.1
Hymenoptera	Chalcidoidea	Eulophidae	Eulophidae	sp.	KR408244.1
Hymenoptera	Chalcidoidea	Eulophidae	Eulophidae	sp.	KR408576.1
Hymenoptera	Chalcidoidea	Eulophidae	Eulophidae	sp.	KR409470.1
Hymenoptera	Chalcidoidea	Eulophidae	Eulophidae	sp.	KR409525.1
Hymenoptera	Chalcidoidea	Eulophidae	Eulophidae	sp.	KR409545.1
Hymenoptera	Chalcidoidea	Eulophidae	Eulophidae	sp.	KR409970.1
Hymenoptera	Chalcidoidea	Eulophidae	Eulophidae	sp.	KR410124.1
Hymenoptera	Chalcidoidea	Eulophidae	Eulophidae	sp.	KR410131.1
Hymenoptera	Chalcidoidea	Eulophidae	Eulophidae	sp.	KR410189.1
Hymenoptera	Chalcidoidea	Eulophidae	Eulophidae	sp.	KR410524.1
Hymenoptera	Chalcidoidea	Eulophidae	Eulophidae	sp.	KR411192.1
Hymenoptera	Chalcidoidea	Eulophidae	Eulophidae	sp.	KR411262.1
Hymenoptera	Chalcidoidea	Eulophidae	Eulophidae	sp.	KR412686.1
Hymenoptera	Chalcidoidea	Eulophidae	Eulophidae	sp.	KR413228.1
Hymenoptera	Chalcidoidea	Eulophidae	Eulophidae	sp.	KR413233.1
Hymenoptera	Chalcidoidea	Eulophidae	Eulophidae	sp.	KR413377.1
Hymenoptera	Chalcidoidea	Eulophidae	Eulophidae	sp.	KR413934.1
Hymenoptera	Chalcidoidea	Eulophidae	Eulophidae	sp.	KR414226.1
Hymenoptera	Chalcidoidea	Eulophidae	Eulophidae	sp.	KR414279.1
Hymenoptera	Chalcidoidea	Eulophidae	Eulophidae	sp.	KR414621.1
Hymenoptera	Chalcidoidea	Eulophidae	Eulophidae	sp.	KR414629.1
Hymenoptera	Chalcidoidea	Eulophidae	Eulophidae	sp.	KR415004.1
Hymenoptera	Chalcidoidea	Eulophidae	Eulophidae	sp.	KR415346.1
Hymenoptera	Chalcidoidea	Eulophidae	Eulophidae	sp.	KR415651.1
Hymenoptera	Chalcidoidea	Eulophidae	Eulophidae	sp.	KR416141.1
Hymenoptera	Chalcidoidea	Eulophidae	Eulophidae	sp.	KR416335.1
Hymenoptera	Chalcidoidea	Eulophidae	Eulophidae	sp.	KR416448.1
Hymenoptera	Chalcidoidea	Eulophidae	Eulophidae	sp.	KR417674.1
Hymenoptera	Chalcidoidea	Eulophidae	Eulophidae	sp.	KR417813.1
Hymenoptera	Chalcidoidea	Eulophidae	Eulophidae	sp.	KR417967.1
Hymenoptera	Chalcidoidea	Eulophidae	Eulophidae	sp.	KR418399.1
Hymenoptera	Chalcidoidea	Eulophidae	Eulophidae	sp.	KR418999.1

Hymenoptera	Chalcidoidea	Eulophidae	Eulophidae	sp.	KR419229.1
Hymenoptera	Chalcidoidea	Eulophidae	Eulophidae	sp.	KR419318.1
Hymenoptera	Chalcidoidea	Eulophidae	Eulophidae	sp.	KR419471.1
Hymenoptera	Chalcidoidea	Eulophidae	Eulophidae	sp.	KR419517.1
Hymenoptera	Chalcidoidea	Eulophidae	Eulophidae	sp.	KR419814.1
Hymenoptera	Chalcidoidea	Eulophidae	Eulophidae	sp.	KR419980.1
Hymenoptera	Chalcidoidea	Eulophidae	Eulophidae	sp.	KR420090.1
Hymenoptera	Chalcidoidea	Eulophidae	Eulophidae	sp.	KR421039.1
Hymenoptera	Chalcidoidea	Eulophidae	Eulophidae	sp.	KR421097.1
Hymenoptera	Chalcidoidea	Eulophidae	Eulophidae	sp.	KR421957.1
Hymenoptera	Chalcidoidea	Eulophidae	Eulophidae	sp.	KR421977.1
Hymenoptera	Chalcidoidea	Eulophidae	Eulophidae	sp.	KR782453.1
Hymenoptera	Chalcidoidea	Eulophidae	Eulophidae	sp.	KR782568.1
Hymenoptera	Chalcidoidea	Eulophidae	Eulophidae	sp.	KR782587.1
Hymenoptera	Chalcidoidea	Eulophidae	Eulophidae	sp.	KR782742.1
Hymenoptera	Chalcidoidea	Eulophidae	Eulophidae	sp.	KR782885.1
Hymenoptera	Chalcidoidea	Eulophidae	Eulophidae	sp.	KR782937.1
Hymenoptera	Chalcidoidea	Eulophidae	Eulophidae	sp.	KR783104.1
Hymenoptera	Chalcidoidea	Eulophidae	Eulophidae	sp.	KR783282.1
Hymenoptera	Chalcidoidea	Eulophidae	Eulophidae	sp.	KR783301.1
Hymenoptera	Chalcidoidea	Eulophidae	Eulophidae	sp.	KR783362.1
Hymenoptera	Chalcidoidea	Eulophidae	Eulophidae	sp.	KR783585.1
Hymenoptera	Chalcidoidea	Eulophidae	Eulophidae	sp.	KR783604.1
Hymenoptera	Chalcidoidea	Eulophidae	Eulophidae	sp.	KR783605.1
Hymenoptera	Chalcidoidea	Eulophidae	Eulophidae	sp.	KR783732.1
Hymenoptera	Chalcidoidea	Eulophidae	Eulophidae	sp.	KR783862.1
Hymenoptera	Chalcidoidea	Eulophidae	Eulophidae	sp.	KR783879.1
Hymenoptera	Chalcidoidea	Eulophidae	Eulophidae	sp.	KR783910.1
Hymenoptera	Chalcidoidea	Eulophidae	Eulophidae	sp.	KR783954.1
Hymenoptera	Chalcidoidea	Eulophidae	Eulophidae	sp.	KR784214.1
Hymenoptera	Chalcidoidea	Eulophidae	Eulophidae	sp.	KR784241.1
Hymenoptera	Chalcidoidea	Eulophidae	Eulophidae	sp.	KR784288.1
Hymenoptera	Chalcidoidea	Eulophidae	Eulophidae	sp.	KR784574.1
Hymenoptera	Chalcidoidea	Eulophidae	Eulophidae	sp.	KR784652.1
Hymenoptera	Chalcidoidea	Eulophidae	Eulophidae	sp.	KR784800.1
Hymenoptera	Chalcidoidea	Eulophidae	Eulophidae	sp.	KR784812.1
Hymenoptera	Chalcidoidea	Eulophidae	Eulophidae	sp.	KR784989.1
Hymenoptera	Chalcidoidea	Eulophidae	Eulophidae	sp.	KR785001.1
Hymenoptera	Chalcidoidea	Eulophidae	Eulophidae	sp.	KR785210.1
Hymenoptera	Chalcidoidea	Eulophidae	Eulophidae	sp.	KR785224.1
Hymenoptera	Chalcidoidea	Eulophidae	Eulophidae	sp.	KR785244.1
Hymenoptera	Chalcidoidea	Eulophidae	Eulophidae	sp.	KR785272.1
Hymenoptera	Chalcidoidea	Eulophidae	Eulophidae	sp.	KR785447.1
Hymenoptera	Chalcidoidea	Eulophidae	Eulophidae	sp.	KR785500.1
Hymenoptera	Chalcidoidea	Eulophidae	Eulophidae	sp.	KR785515.1
Hymenoptera	Chalcidoidea	Eulophidae	Eulophidae	sp.	KR785625.1
Hymenoptera	Chalcidoidea	Eulophidae	Eulophidae	sp.	KR785682.1
Hymenoptera	Chalcidoidea	Eulophidae	Eulophidae	sp.	KR785685.1
Hymenoptera	Chalcidoidea	Eulophidae	Eulophidae	sp.	KR785711.1
Hymenoptera	Chalcidoidea	Eulophidae	Eulophidae	sp.	KR785757.1
Hymenoptera	Chalcidoidea	Eulophidae	Eulophidae	sp.	KR785846.1
Hymenoptera	Chalcidoidea	Eulophidae	Eulophidae	sp.	KR786028.1
Hymenoptera	Chalcidoidea	Eulophidae	Eulophidae	sp.	KR786036.1
Hymenoptera	Chalcidoidea	Eulophidae	Eulophidae	sp.	KR786075.1

Hymenoptera	Chalcidoidea	Eulophidae	Eulophidae	sp.	KR786257.1
Hymenoptera	Chalcidoidea	Eulophidae	Eulophidae	sp.	KR786318.1
Hymenoptera	Chalcidoidea	Eulophidae	Eulophidae	sp.	KR786320.1
Hymenoptera	Chalcidoidea	Eulophidae	Eulophidae	sp.	KR786369.1
Hymenoptera	Chalcidoidea	Eulophidae	Eulophidae	sp.	KR786381.1
Hymenoptera	Chalcidoidea	Eulophidae	Eulophidae	sp.	KR786385.1
Hymenoptera	Chalcidoidea	Eulophidae	Eulophidae	sp.	KR786537.1
Hymenoptera	Chalcidoidea	Eulophidae	Eulophidae	sp.	KR786546.1
Hymenoptera	Chalcidoidea	Eulophidae	Eulophidae	sp.	KR786621.1
Hymenoptera	Chalcidoidea	Eulophidae	Eulophidae	sp.	KR786738.1
Hymenoptera	Chalcidoidea	Eulophidae	Eulophidae	sp.	KR786814.1
Hymenoptera	Chalcidoidea	Eulophidae	Eulophidae	sp.	KR786917.1
Hymenoptera	Chalcidoidea	Eulophidae	Eulophidae	sp.	KR786943.1
Hymenoptera	Chalcidoidea	Eulophidae	Eulophidae	sp.	KR787229.1
Hymenoptera	Chalcidoidea	Eulophidae	Eulophidae	sp.	KR787348.1
Hymenoptera	Chalcidoidea	Eulophidae	Eulophidae	sp.	KR787434.1
Hymenoptera	Chalcidoidea	Eulophidae	Eulophidae	sp.	KR787526.1
Hymenoptera	Chalcidoidea	Eulophidae	Eulophidae	sp.	KR787615.1
Hymenoptera	Chalcidoidea	Eulophidae	Eulophidae	sp.	KR787640.1
Hymenoptera	Chalcidoidea	Eulophidae	Eulophidae	sp.	KR787683.1
Hymenoptera	Chalcidoidea	Eulophidae	Eulophidae	sp.	KR787758.1
Hymenoptera	Chalcidoidea	Eulophidae	Eulophidae	sp.	KR787844.1
Hymenoptera	Chalcidoidea	Eulophidae	Eulophidae	sp.	KR787895.1
Hymenoptera	Chalcidoidea	Eulophidae	Eulophidae	sp.	KR788007.1
Hymenoptera	Chalcidoidea	Eulophidae	Eulophidae	sp.	KR788173.1
Hymenoptera	Chalcidoidea	Eulophidae	Eulophidae	sp.	KR788298.1
Hymenoptera	Chalcidoidea	Eulophidae	Eulophidae	sp.	KR788506.1
Hymenoptera	Chalcidoidea	Eulophidae	Eulophidae	sp.	KR788575.1
Hymenoptera	Chalcidoidea	Eulophidae	Eulophidae	sp.	KR788676.1
Hymenoptera	Chalcidoidea	Eulophidae	Eulophidae	sp.	KR788791.1
Hymenoptera	Chalcidoidea	Eulophidae	Eulophidae	sp.	KR788935.1
Hymenoptera	Chalcidoidea	Eulophidae	Eulophidae	sp.	KR788998.1
Hymenoptera	Chalcidoidea	Eulophidae	Eulophidae	sp.	KR789016.1
Hymenoptera	Chalcidoidea	Eulophidae	Eulophidae	sp.	KR789103.1
Hymenoptera	Chalcidoidea	Eulophidae	Eulophidae	sp.	KR789109.1
Hymenoptera	Chalcidoidea	Eulophidae	Eulophidae	sp.	KR789618.1
Hymenoptera	Chalcidoidea	Eulophidae	Eulophidae	sp.	KR789663.1
Hymenoptera	Chalcidoidea	Eulophidae	Eulophidae	sp.	KR789726.1
Hymenoptera	Chalcidoidea	Eulophidae	Eulophidae	sp.	KR789910.1
Hymenoptera	Chalcidoidea	Eulophidae	Eulophidae	sp.	KR790078.1
Hymenoptera	Chalcidoidea	Eulophidae	Eulophidae	sp.	KR790127.1
Hymenoptera	Chalcidoidea	Eulophidae	Eulophidae	sp.	KR790157.1
Hymenoptera	Chalcidoidea	Eulophidae	Eulophidae	sp.	KR790341.1
Hymenoptera	Chalcidoidea	Eulophidae	Eulophidae	sp.	KR790342.1
Hymenoptera	Chalcidoidea	Eulophidae	Eulophidae	sp.	KR790856.1
Hymenoptera	Chalcidoidea	Eulophidae	Eulophidae	sp.	KR790890.1
Hymenoptera	Chalcidoidea	Eulophidae	Eulophidae	sp.	KR791095.1
Hymenoptera	Chalcidoidea	Eulophidae	Eulophidae	sp.	KR791221.1
Hymenoptera	Chalcidoidea	Eulophidae	Eulophidae	sp.	KR791361.1
Hymenoptera	Chalcidoidea	Eulophidae	Eulophidae	sp.	KR791506.1
Hymenoptera	Chalcidoidea	Eulophidae	Eulophidae	sp.	KR791676.1
Hymenoptera	Chalcidoidea	Eulophidae	Eulophidae	sp.	KR791721.1
Hymenoptera	Chalcidoidea	Eulophidae	Eulophidae	sp.	KR791827.1
Hymenoptera	Chalcidoidea	Eulophidae	Eulophidae	sp.	KR791848.1

Hymenoptera	Chalcidoidea	Eulophidae	Eulophidae	sp.	KR792006.1
Hymenoptera	Chalcidoidea	Eulophidae	Eulophidae	sp.	KR792038.1
Hymenoptera	Chalcidoidea	Eulophidae	Eulophidae	sp.	KR792039.1
Hymenoptera	Chalcidoidea	Eulophidae	Eulophidae	sp.	KR792046.1
Hymenoptera	Chalcidoidea	Eulophidae	Eulophidae	sp.	KR792288.1
Hymenoptera	Chalcidoidea	Eulophidae	Eulophidae	sp.	KR792790.1
Hymenoptera	Chalcidoidea	Eulophidae	Eulophidae	sp.	KR792820.1
Hymenoptera	Chalcidoidea	Eulophidae	Eulophidae	sp.	KR792878.1
Hymenoptera	Chalcidoidea	Eulophidae	Eulophidae	sp.	KR793257.1
Hymenoptera	Chalcidoidea	Eulophidae	Eulophidae	sp.	KR793275.1
Hymenoptera	Chalcidoidea	Eulophidae	Eulophidae	sp.	KR793534.1
Hymenoptera	Chalcidoidea	Eulophidae	Eulophidae	sp.	KR793741.1
Hymenoptera	Chalcidoidea	Eulophidae	Eulophidae	sp.	KR793874.1
Hymenoptera	Chalcidoidea	Eulophidae	Eulophidae	sp.	KR793921.1
Hymenoptera	Chalcidoidea	Eulophidae	Eulophidae	sp.	KR793955.1
Hymenoptera	Chalcidoidea	Eulophidae	Eulophidae	sp.	KR794134.1
Hymenoptera	Chalcidoidea	Eulophidae	Eulophidae	sp.	KR794135.1
Hymenoptera	Chalcidoidea	Eulophidae	Eulophidae	sp.	KR794238.1
Hymenoptera	Chalcidoidea	Eulophidae	Eulophidae	sp.	KR794272.1
Hymenoptera	Chalcidoidea	Eulophidae	Eulophidae	sp.	KR794331.1
Hymenoptera	Chalcidoidea	Eulophidae	Eulophidae	sp.	KR794367.1
Hymenoptera	Chalcidoidea	Eulophidae	Eulophidae	sp.	KR794372.1
Hymenoptera	Chalcidoidea	Eulophidae	Eulophidae	sp.	KR794457.1
Hymenoptera	Chalcidoidea	Eulophidae	Eulophidae	sp.	KR794565.1
Hymenoptera	Chalcidoidea	Eulophidae	Eulophidae	sp.	KR794769.1
Hymenoptera	Chalcidoidea	Eulophidae	Eulophidae	sp.	KR795089.1
Hymenoptera	Chalcidoidea	Eulophidae	Eulophidae	sp.	KR795091.1
Hymenoptera	Chalcidoidea	Eulophidae	Eulophidae	sp.	KR795096.1
Hymenoptera	Chalcidoidea	Eulophidae	Eulophidae	sp.	KR795217.1
Hymenoptera	Chalcidoidea	Eulophidae	Eulophidae	sp.	KR795519.1
Hymenoptera	Chalcidoidea	Eulophidae	Eulophidae	sp.	KR795523.1
Hymenoptera	Chalcidoidea	Eulophidae	Eulophidae	sp.	KR795531.1
Hymenoptera	Chalcidoidea	Eulophidae	Eulophidae	sp.	KR795538.1
Hymenoptera	Chalcidoidea	Eulophidae	Eulophidae	sp.	KR795729.1
Hymenoptera	Chalcidoidea	Eulophidae	Eulophidae	sp.	KR795874.1
Hymenoptera	Chalcidoidea	Eulophidae	Eulophidae	sp.	KR795994.1
Hymenoptera	Chalcidoidea	Eulophidae	Eulophidae	sp.	KR796053.1
Hymenoptera	Chalcidoidea	Eulophidae	Eulophidae	sp.	KR796169.1
Hymenoptera	Chalcidoidea	Eulophidae	Eulophidae	sp.	KR796231.1
Hymenoptera	Chalcidoidea	Eulophidae	Eulophidae	sp.	KR796481.1
Hymenoptera	Chalcidoidea	Eulophidae	Eulophidae	sp.	KR796487.1
Hymenoptera	Chalcidoidea	Eulophidae	Eulophidae	sp.	KR796564.1
Hymenoptera	Chalcidoidea	Eulophidae	Eulophidae	sp.	KR796608.1
Hymenoptera	Chalcidoidea	Eulophidae	Eulophidae	sp.	KR796637.1
Hymenoptera	Chalcidoidea	Eulophidae	Eulophidae	sp.	KR796730.1
Hymenoptera	Chalcidoidea	Eulophidae	Eulophidae	sp.	KR796992.1
Hymenoptera	Chalcidoidea	Eulophidae	Eulophidae	sp.	KR797066.1
Hymenoptera	Chalcidoidea	Eulophidae	Eulophidae	sp.	KR797085.1
Hymenoptera	Chalcidoidea	Eulophidae	Eulophidae	sp.	KR797119.1
Hymenoptera	Chalcidoidea	Eulophidae	Eulophidae	sp.	KR797145.1
Hymenoptera	Chalcidoidea	Eulophidae	Eulophidae	sp.	KR797166.1
Hymenoptera	Chalcidoidea	Eulophidae	Eulophidae	sp.	KR797170.1
Hymenoptera	Chalcidoidea	Eulophidae	Eulophidae	sp.	KR797359.1
Hymenoptera	Chalcidoidea	Eulophidae	Eulophidae	sp.	KR797485.1

Hymenoptera	Chalcidoidea	Eulophidae	Eulophidae	sp.	KR797559.1
Hymenoptera	Chalcidoidea	Eulophidae	Eulophidae	sp.	KR797600.1
Hymenoptera	Chalcidoidea	Eulophidae	Eulophidae	sp.	KR797629.1
Hymenoptera	Chalcidoidea	Eulophidae	Eulophidae	sp.	KR798045.1
Hymenoptera	Chalcidoidea	Eulophidae	Eulophidae	sp.	KR798047.1
Hymenoptera	Chalcidoidea	Eulophidae	Eulophidae	sp.	KR798200.1
Hymenoptera	Chalcidoidea	Eulophidae	Eulophidae	sp.	KR798209.1
Hymenoptera	Chalcidoidea	Eulophidae	Eulophidae	sp.	KR798282.1
Hymenoptera	Chalcidoidea	Eulophidae	Eulophidae	sp.	KR798397.1
Hymenoptera	Chalcidoidea	Eulophidae	Eulophidae	sp.	KR798606.1
Hymenoptera	Chalcidoidea	Eulophidae	Eulophidae	sp.	KR798612.1
Hymenoptera	Chalcidoidea	Eulophidae	Eulophidae	sp.	KR798810.1
Hymenoptera	Chalcidoidea	Eulophidae	Eulophidae	sp.	KR798814.1
Hymenoptera	Chalcidoidea	Eulophidae	Eulophidae	sp.	KR798856.1
Hymenoptera	Chalcidoidea	Eulophidae	Eulophidae	sp.	KR799050.1
Hymenoptera	Chalcidoidea	Eulophidae	Eulophidae	sp.	KR799059.1
Hymenoptera	Chalcidoidea	Eulophidae	Eulophidae	sp.	KR799090.1
Hymenoptera	Chalcidoidea	Eulophidae	Eulophidae	sp.	KR799436.1
Hymenoptera	Chalcidoidea	Eulophidae	Eulophidae	sp.	KR799442.1
Hymenoptera	Chalcidoidea	Eulophidae	Eulophidae	sp.	KR799616.1
Hymenoptera	Chalcidoidea	Eulophidae	Eulophidae	sp.	KR799763.1
Hymenoptera	Chalcidoidea	Eulophidae	Eulophidae	sp.	KR799773.1
Hymenoptera	Chalcidoidea	Eulophidae	Eulophidae	sp.	KR799916.1
Hymenoptera	Chalcidoidea	Eulophidae	Eulophidae	sp.	KR799962.1
Hymenoptera	Chalcidoidea	Eulophidae	Eulophidae	sp.	KR800037.1
Hymenoptera	Chalcidoidea	Eulophidae	Eulophidae	sp.	KR800083.1
Hymenoptera	Chalcidoidea	Eulophidae	Eulophidae	sp.	KR800284.1
Hymenoptera	Chalcidoidea	Eulophidae	Eulophidae	sp.	KR800439.1
Hymenoptera	Chalcidoidea	Eulophidae	Eulophidae	sp.	KR800584.1
Hymenoptera	Chalcidoidea	Eulophidae	Eulophidae	sp.	KR800612.1
Hymenoptera	Chalcidoidea	Eulophidae	Eulophidae	sp.	KR800646.1
Hymenoptera	Chalcidoidea	Eulophidae	Eulophidae	sp.	KR801067.1
Hymenoptera	Chalcidoidea	Eulophidae	Eulophidae	sp.	KR801165.1
Hymenoptera	Chalcidoidea	Eulophidae	Eulophidae	sp.	KR801217.1
Hymenoptera	Chalcidoidea	Eulophidae	Eulophidae	sp.	KR801249.1
Hymenoptera	Chalcidoidea	Eulophidae	Eulophidae	sp.	KR801262.1
Hymenoptera	Chalcidoidea	Eulophidae	Eulophidae	sp.	KR801433.1
Hymenoptera	Chalcidoidea	Eulophidae	Eulophidae	sp.	KR801492.1
Hymenoptera	Chalcidoidea	Eulophidae	Eulophidae	sp.	KR801502.1
Hymenoptera	Chalcidoidea	Eulophidae	Eulophidae	sp.	KR801539.1
Hymenoptera	Chalcidoidea	Eulophidae	Eulophidae	sp.	KR801612.1
Hymenoptera	Chalcidoidea	Eulophidae	Eulophidae	sp.	KR801634.1
Hymenoptera	Chalcidoidea	Eulophidae	Eulophidae	sp.	KR801690.1
Hymenoptera	Chalcidoidea	Eulophidae	Eulophidae	sp.	KR801731.1
Hymenoptera	Chalcidoidea	Eulophidae	Eulophidae	sp.	KR802130.1
Hymenoptera	Chalcidoidea	Eulophidae	Eulophidae	sp.	KR802152.1
Hymenoptera	Chalcidoidea	Eulophidae	Eulophidae	sp.	KR802315.1
Hymenoptera	Chalcidoidea	Eulophidae	Eulophidae	sp.	KR802410.1
Hymenoptera	Chalcidoidea	Eulophidae	Eulophidae	sp.	KR802542.1
Hymenoptera	Chalcidoidea	Eulophidae	Eulophidae	sp.	KR802973.1
Hymenoptera	Chalcidoidea	Eulophidae	Eulophidae	sp.	KR802981.1
Hymenoptera	Chalcidoidea	Eulophidae	Eulophidae	sp.	KR803210.1
Hymenoptera	Chalcidoidea	Eulophidae	Eulophidae	sp.	KR803318.1
Hymenoptera	Chalcidoidea	Eulophidae	Eulophidae	sp.	KR803361.1

Hymenoptera	Chalcidoidea	Eulophidae	Eulophidae	sp.	KR803438.1
Hymenoptera	Chalcidoidea	Eulophidae	Eulophidae	sp.	KR803650.1
Hymenoptera	Chalcidoidea	Eulophidae	Eulophidae	sp.	KR803770.1
Hymenoptera	Chalcidoidea	Eulophidae	Eulophidae	sp.	KR803800.1
Hymenoptera	Chalcidoidea	Eulophidae	Eulophidae	sp.	KR803823.1
Hymenoptera	Chalcidoidea	Eulophidae	Eulophidae	sp.	KR803835.1
Hymenoptera	Chalcidoidea	Eulophidae	Eulophidae	sp.	KR803836.1
Hymenoptera	Chalcidoidea	Eulophidae	Eulophidae	sp.	KR803844.1
Hymenoptera	Chalcidoidea	Eulophidae	Eulophidae	sp.	KR803949.1
Hymenoptera	Chalcidoidea	Eulophidae	Eulophidae	sp.	KR804071.1
Hymenoptera	Chalcidoidea	Eulophidae	Eulophidae	sp.	KR804078.1
Hymenoptera	Chalcidoidea	Eulophidae	Eulophidae	sp.	KR804200.1
Hymenoptera	Chalcidoidea	Eulophidae	Eulophidae	sp.	KR804546.1
Hymenoptera	Chalcidoidea	Eulophidae	Eulophidae	sp.	KR804588.1
Hymenoptera	Chalcidoidea	Eulophidae	Eulophidae	sp.	KR804607.1
Hymenoptera	Chalcidoidea	Eulophidae	Eulophidae	sp.	KR804959.1
Hymenoptera	Chalcidoidea	Eulophidae	Eulophidae	sp.	KR805105.1
Hymenoptera	Chalcidoidea	Eulophidae	Eulophidae	sp.	KR805117.1
Hymenoptera	Chalcidoidea	Eulophidae	Eulophidae	sp.	KR805235.1
Hymenoptera	Chalcidoidea	Eulophidae	Eulophidae	sp.	KR805293.1
Hymenoptera	Chalcidoidea	Eulophidae	Eulophidae	sp.	KR805522.1
Hymenoptera	Chalcidoidea	Eulophidae	Eulophidae	sp.	KR805525.1
Hymenoptera	Chalcidoidea	Eulophidae	Eulophidae	sp.	KR805648.1
Hymenoptera	Chalcidoidea	Eulophidae	Eulophidae	sp.	KR805651.1
Hymenoptera	Chalcidoidea	Eulophidae	Eulophidae	sp.	KR805770.1
Hymenoptera	Chalcidoidea	Eulophidae	Eulophidae	sp.	KR806049.1
Hymenoptera	Chalcidoidea	Eulophidae	Eulophidae	sp.	KR806133.1
Hymenoptera	Chalcidoidea	Eulophidae	Eulophidae	sp.	KR806149.1
Hymenoptera	Chalcidoidea	Eulophidae	Eulophidae	sp.	KR806172.1
Hymenoptera	Chalcidoidea	Eulophidae	Eulophidae	sp.	KR806176.1
Hymenoptera	Chalcidoidea	Eulophidae	Eulophidae	sp.	KR806293.1
Hymenoptera	Chalcidoidea	Eulophidae	Eulophidae	sp.	KR806540.1
Hymenoptera	Chalcidoidea	Eulophidae	Eulophidae	sp.	KR806574.1
Hymenoptera	Chalcidoidea	Eulophidae	Eulophidae	sp.	KR806581.1
Hymenoptera	Chalcidoidea	Eulophidae	Eulophidae	sp.	KR806757.1
Hymenoptera	Chalcidoidea	Eulophidae	Eulophidae	sp.	KR806815.1
Hymenoptera	Chalcidoidea	Eulophidae	Eulophidae	sp.	KR806917.1
Hymenoptera	Chalcidoidea	Eulophidae	Eulophidae	sp.	KR807056.1
Hymenoptera	Chalcidoidea	Eulophidae	Eulophidae	sp.	KR807095.1
Hymenoptera	Chalcidoidea	Eulophidae	Eulophidae	sp.	KR807133.1
Hymenoptera	Chalcidoidea	Eulophidae	Eulophidae	sp.	KR807153.1
Hymenoptera	Chalcidoidea	Eulophidae	Eulophidae	sp.	KR807353.1
Hymenoptera	Chalcidoidea	Eulophidae	Eulophidae	sp.	KR807359.1
Hymenoptera	Chalcidoidea	Eulophidae	Eulophidae	sp.	KR807378.1
Hymenoptera	Chalcidoidea	Eulophidae	Eulophidae	sp.	KR807407.1
Hymenoptera	Chalcidoidea	Eulophidae	Eulophidae	sp.	KR807516.1
Hymenoptera	Chalcidoidea	Eulophidae	Eulophidae	sp.	KR807535.1
Hymenoptera	Chalcidoidea	Eulophidae	Eulophidae	sp.	KR807624.1
Hymenoptera	Chalcidoidea	Eulophidae	Eulophidae	sp.	KR807778.1
Hymenoptera	Chalcidoidea	Eulophidae	Eulophidae	sp.	KR807876.1
Hymenoptera	Chalcidoidea	Eulophidae	Eulophidae	sp.	KR807889.1
Hymenoptera	Chalcidoidea	Eulophidae	Eulophidae	sp.	KR808153.1
Hymenoptera	Chalcidoidea	Eulophidae	Eulophidae	sp.	KR808284.1
Hymenoptera	Chalcidoidea	Eulophidae	Eulophidae	sp.	KR808304.1

Hymenoptera	Chalcidoidea	Eulophidae	Eulophidae	sp.	KR808349.1
Hymenoptera	Chalcidoidea	Eulophidae	Eulophidae	sp.	KR808438.1
Hymenoptera	Chalcidoidea	Eulophidae	Eulophidae	sp.	KR808476.1
Hymenoptera	Chalcidoidea	Eulophidae	Eulophidae	sp.	KR808734.1
Hymenoptera	Chalcidoidea	Eulophidae	Eulophidae	sp.	KR808820.1
Hymenoptera	Chalcidoidea	Eulophidae	Eulophidae	sp.	KR808851.1
Hymenoptera	Chalcidoidea	Eulophidae	Eulophidae	sp.	KR808882.1
Hymenoptera	Chalcidoidea	Eulophidae	Eulophidae	sp.	KR808947.1
Hymenoptera	Chalcidoidea	Eulophidae	Eulophidae	sp.	KR808983.1
Hymenoptera	Chalcidoidea	Eulophidae	Eulophidae	sp.	KR809148.1
Hymenoptera	Chalcidoidea	Eulophidae	Eulophidae	sp.	KR809250.1
Hymenoptera	Chalcidoidea	Eulophidae	Eulophidae	sp.	KR809325.1
Hymenoptera	Chalcidoidea	Eulophidae	Eulophidae	sp.	KR873604.1
Hymenoptera	Chalcidoidea	Eulophidae	Eulophidae	sp.	KR874027.1
Hymenoptera	Chalcidoidea	Eulophidae	Eulophidae	sp.	KR874578.1
Hymenoptera	Chalcidoidea	Eulophidae	Eulophidae	sp.	KR874608.1
Hymenoptera	Chalcidoidea	Eulophidae	Eulophidae	sp.	KR874728.1
Hymenoptera	Chalcidoidea	Eulophidae	Eulophidae	sp.	KR874980.1
Hymenoptera	Chalcidoidea	Eulophidae	Eulophidae	sp.	KR875108.1
Hymenoptera	Chalcidoidea	Eulophidae	Eulophidae	sp.	KR875354.1
Hymenoptera	Chalcidoidea	Eulophidae	Eulophidae	sp.	KR875356.1
Hymenoptera	Chalcidoidea	Eulophidae	Eulophidae	sp.	KR875409.1
Hymenoptera	Chalcidoidea	Eulophidae	Eulophidae	sp.	KR875542.1
Hymenoptera	Chalcidoidea	Eulophidae	Eulophidae	sp.	KR875698.1
Hymenoptera	Chalcidoidea	Eulophidae	Eulophidae	sp.	KR875916.1
Hymenoptera	Chalcidoidea	Eulophidae	Eulophidae	sp.	KR875976.1
Hymenoptera	Chalcidoidea	Eulophidae	Eulophidae	sp.	KR875990.1
Hymenoptera	Chalcidoidea	Eulophidae	Eulophidae	sp.	KR876040.1
Hymenoptera	Chalcidoidea	Eulophidae	Eulophidae	sp.	KR876108.1
Hymenoptera	Chalcidoidea	Eulophidae	Eulophidae	sp.	KR876397.1
Hymenoptera	Chalcidoidea	Eulophidae	Eulophidae	sp.	KR876407.1
Hymenoptera	Chalcidoidea	Eulophidae	Eulophidae	sp.	KR876512.1
Hymenoptera	Chalcidoidea	Eulophidae	Eulophidae	sp.	KR876520.1
Hymenoptera	Chalcidoidea	Eulophidae	Eulophidae	sp.	KR876702.1
Hymenoptera	Chalcidoidea	Eulophidae	Eulophidae	sp.	KR876719.1
Hymenoptera	Chalcidoidea	Eulophidae	Eulophidae	sp.	KR876758.1
Hymenoptera	Chalcidoidea	Eulophidae	Eulophidae	sp.	KR876943.1
Hymenoptera	Chalcidoidea	Eulophidae	Eulophidae	sp.	KR876999.1
Hymenoptera	Chalcidoidea	Eulophidae	Eulophidae	sp.	KR877210.1
Hymenoptera	Chalcidoidea	Eulophidae	Eulophidae	sp.	KR877627.1
Hymenoptera	Chalcidoidea	Eulophidae	Eulophidae	sp.	KR877783.1
Hymenoptera	Chalcidoidea	Eulophidae	Eulophidae	sp.	KR878113.1
Hymenoptera	Chalcidoidea	Eulophidae	Eulophidae	sp.	KR878339.1
Hymenoptera	Chalcidoidea	Eulophidae	Eulophidae	sp.	KR878358.1
Hymenoptera	Chalcidoidea	Eulophidae	Eulophidae	sp.	KR878817.1
Hymenoptera	Chalcidoidea	Eulophidae	Eulophidae	sp.	KR878883.1
Hymenoptera	Chalcidoidea	Eulophidae	Eulophidae	sp.	KR879099.1
Hymenoptera	Chalcidoidea	Eulophidae	Eulophidae	sp.	KR879145.1
Hymenoptera	Chalcidoidea	Eulophidae	Eulophidae	sp.	KR879150.1
Hymenoptera	Chalcidoidea	Eulophidae	Eulophidae	sp.	KR879259.1
Hymenoptera	Chalcidoidea	Eulophidae	Eulophidae	sp.	KR879281.1
Hymenoptera	Chalcidoidea	Eulophidae	Eulophidae	sp.	KR879356.1
Hymenoptera	Chalcidoidea	Eulophidae	Eulophidae	sp.	KR879796.1
Hymenoptera	Chalcidoidea	Eulophidae	Eulophidae	sp.	KR879938.1

Hymenoptera	Chalcidoidea	Eulophidae	Eulophidae	sp.	KR880329.1
Hymenoptera	Chalcidoidea	Eulophidae	Eulophidae	sp.	KR880388.1
Hymenoptera	Chalcidoidea	Eulophidae	Eulophidae	sp.	KR880596.1
Hymenoptera	Chalcidoidea	Eulophidae	Eulophidae	sp.	KR880751.1
Hymenoptera	Chalcidoidea	Eulophidae	Eulophidae	sp.	KR881362.1
Hymenoptera	Chalcidoidea	Eulophidae	Eulophidae	sp.	KR881490.1
Hymenoptera	Chalcidoidea	Eulophidae	Eulophidae	sp.	KR881791.1
Hymenoptera	Chalcidoidea	Eulophidae	Eulophidae	sp.	KR881797.1
Hymenoptera	Chalcidoidea	Eulophidae	Eulophidae	sp.	KR882476.1
Hymenoptera	Chalcidoidea	Eulophidae	Eulophidae	sp.	KR882621.1
Hymenoptera	Chalcidoidea	Eulophidae	Eulophidae	sp.	KR882733.1
Hymenoptera	Chalcidoidea	Eulophidae	Eulophidae	sp.	KR882954.1
Hymenoptera	Chalcidoidea	Eulophidae	Eulophidae	sp.	KR883061.1
Hymenoptera	Chalcidoidea	Eulophidae	Eulophidae	sp.	KR883185.1
Hymenoptera	Chalcidoidea	Eulophidae	Eulophidae	sp.	KR883202.1
Hymenoptera	Chalcidoidea	Eulophidae	Eulophidae	sp.	KR883554.1
Hymenoptera	Chalcidoidea	Eulophidae	Eulophidae	sp.	KR883655.1
Hymenoptera	Chalcidoidea	Eulophidae	Eulophidae	sp.	KR883729.1
Hymenoptera	Chalcidoidea	Eulophidae	Eulophidae	sp.	KR883751.1
Hymenoptera	Chalcidoidea	Eulophidae	Eulophidae	sp.	KR883875.1
Hymenoptera	Chalcidoidea	Eulophidae	Eulophidae	sp.	KR884243.1
Hymenoptera	Chalcidoidea	Eulophidae	Eulophidae	sp.	KR884268.1
Hymenoptera	Chalcidoidea	Eulophidae	Eulophidae	sp.	KR884521.1
Hymenoptera	Chalcidoidea	Eulophidae	Eulophidae	sp.	KR884583.1
Hymenoptera	Chalcidoidea	Eulophidae	Eulophidae	sp.	KR885020.1
Hymenoptera	Chalcidoidea	Eulophidae	Eulophidae	sp.	KR885542.1
Hymenoptera	Chalcidoidea	Eulophidae	Eulophidae	sp.	KR885659.1
Hymenoptera	Chalcidoidea	Eulophidae	Eulophidae	sp.	KR885668.1
Hymenoptera	Chalcidoidea	Eulophidae	Eulophidae	sp.	KR885765.1
Hymenoptera	Chalcidoidea	Eulophidae	Eulophidae	sp.	KR885810.1
Hymenoptera	Chalcidoidea	Eulophidae	Eulophidae	sp.	KR885886.1
Hymenoptera	Chalcidoidea	Eulophidae	Eulophidae	sp.	KR886142.1
Hymenoptera	Chalcidoidea	Eulophidae	Eulophidae	sp.	KR886169.1
Hymenoptera	Chalcidoidea	Eulophidae	Eulophidae	sp.	KR886313.1
Hymenoptera	Chalcidoidea	Eulophidae	Eulophidae	sp.	KR886336.1
Hymenoptera	Chalcidoidea	Eulophidae	Eulophidae	sp.	KR886435.1
Hymenoptera	Chalcidoidea	Eulophidae	Eulophidae	sp.	KR886443.1
Hymenoptera	Chalcidoidea	Eulophidae	Eulophidae	sp.	KR886521.1
Hymenoptera	Chalcidoidea	Eulophidae	Eulophidae	sp.	KR886632.1
Hymenoptera	Chalcidoidea	Eulophidae	Eulophidae	sp.	KR886732.1
Hymenoptera	Chalcidoidea	Eulophidae	Eulophidae	sp.	KR886742.1
Hymenoptera	Chalcidoidea	Eulophidae	Eulophidae	sp.	KR886762.1
Hymenoptera	Chalcidoidea	Eulophidae	Eulophidae	sp.	KR886808.1
Hymenoptera	Chalcidoidea	Eulophidae	Eulophidae	sp.	KR887439.1
Hymenoptera	Chalcidoidea	Eulophidae	Eulophidae	sp.	KR887490.1
Hymenoptera	Chalcidoidea	Eulophidae	Eulophidae	sp.	KR887809.1
Hymenoptera	Chalcidoidea	Eulophidae	Eulophidae	sp.	KR887843.1
Hymenoptera	Chalcidoidea	Eulophidae	Eulophidae	sp.	KR888025.1
Hymenoptera	Chalcidoidea	Eulophidae	Eulophidae	sp.	KR888109.1
Hymenoptera	Chalcidoidea	Eulophidae	Eulophidae	sp.	KR888659.1
Hymenoptera	Chalcidoidea	Eulophidae	Eulophidae	sp.	KR888674.1
Hymenoptera	Chalcidoidea	Eulophidae	Eulophidae	sp.	KR888701.1
Hymenoptera	Chalcidoidea	Eulophidae	Eulophidae	sp.	KR888768.1
Hymenoptera	Chalcidoidea	Eulophidae	Eulophidae	sp.	KR888786.1

Hymenoptera	Chalcidoidea	Eulophidae	Eulophidae	sp.	KR888996.1
Hymenoptera	Chalcidoidea	Eulophidae	Eulophidae	sp.	KR889010.1
Hymenoptera	Chalcidoidea	Eulophidae	Eulophidae	sp.	KR889222.1
Hymenoptera	Chalcidoidea	Eulophidae	Eulophidae	sp.	KR889289.1
Hymenoptera	Chalcidoidea	Eulophidae	Eulophidae	sp.	KR889367.1
Hymenoptera	Chalcidoidea	Eulophidae	Eulophidae	sp.	KR889370.1
Hymenoptera	Chalcidoidea	Eulophidae	Eulophidae	sp.	KR889616.1
Hymenoptera	Chalcidoidea	Eulophidae	Eulophidae	sp.	KR889661.1
Hymenoptera	Chalcidoidea	Eulophidae	Eulophidae	sp.	KR889785.1
Hymenoptera	Chalcidoidea	Eulophidae	Eulophidae	sp.	KR889820.1
Hymenoptera	Chalcidoidea	Eulophidae	Eulophidae	sp.	KR890114.1
Hymenoptera	Chalcidoidea	Eulophidae	Eulophidae	sp.	KR890347.1
Hymenoptera	Chalcidoidea	Eulophidae	Eulophidae	sp.	KR890536.1
Hymenoptera	Chalcidoidea	Eulophidae	Eulophidae	sp.	KR890643.1
Hymenoptera	Chalcidoidea	Eulophidae	Eulophidae	sp.	KR890666.1
Hymenoptera	Chalcidoidea	Eulophidae	Eulophidae	sp.	KR890809.1
Hymenoptera	Chalcidoidea	Eulophidae	Eulophidae	sp.	KR890850.1
Hymenoptera	Chalcidoidea	Eulophidae	Eulophidae	sp.	KR891353.1
Hymenoptera	Chalcidoidea	Eulophidae	Eulophidae	sp.	KR891446.1
Hymenoptera	Chalcidoidea	Eulophidae	Eulophidae	sp.	KR891466.1
Hymenoptera	Chalcidoidea	Eulophidae	Eulophidae	sp.	KR891518.1
Hymenoptera	Chalcidoidea	Eulophidae	Eulophidae	sp.	KR891579.1
Hymenoptera	Chalcidoidea	Eulophidae	Eulophidae	sp.	KR891786.1
Hymenoptera	Chalcidoidea	Eulophidae	Eulophidae	sp.	KR891962.1
Hymenoptera	Chalcidoidea	Eulophidae	Eulophidae	sp.	KR892042.1
Hymenoptera	Chalcidoidea	Eulophidae	Eulophidae	sp.	KR892083.1
Hymenoptera	Chalcidoidea	Eulophidae	Eulophidae	sp.	KR892384.1
Hymenoptera	Chalcidoidea	Eulophidae	Eulophidae	sp.	KR893147.1
Hymenoptera	Chalcidoidea	Eulophidae	Eulophidae	sp.	KR893243.1
Hymenoptera	Chalcidoidea	Eulophidae	Eulophidae	sp.	KR893695.1
Hymenoptera	Chalcidoidea	Eulophidae	Eulophidae	sp.	KR893931.1
Hymenoptera	Chalcidoidea	Eulophidae	Eulophidae	sp.	KR894415.1
Hymenoptera	Chalcidoidea	Eulophidae	Eulophidae	sp.	KR894501.1
Hymenoptera	Chalcidoidea	Eulophidae	Eulophidae	sp.	KR894893.1
Hymenoptera	Chalcidoidea	Eulophidae	Eulophidae	sp.	KR895671.1
Hymenoptera	Chalcidoidea	Eulophidae	Eulophidae	sp.	KR895754.1
Hymenoptera	Chalcidoidea	Eulophidae	Eulophidae	sp.	KR895831.1
Hymenoptera	Chalcidoidea	Eulophidae	Eulophidae	sp.	KR895849.1
Hymenoptera	Chalcidoidea	Eulophidae	Eulophidae	sp.	KR895891.1
Hymenoptera	Chalcidoidea	Eulophidae	Eulophidae	sp.	KR896315.1
Hymenoptera	Chalcidoidea	Eulophidae	Eulophidae	sp.	KR896414.1
Hymenoptera	Chalcidoidea	Eulophidae	Eulophidae	sp.	KR896447.1
Hymenoptera	Chalcidoidea	Eulophidae	Eulophidae	sp.	KR896513.1
Hymenoptera	Chalcidoidea	Eulophidae	Eulophidae	sp.	KR896673.1
Hymenoptera	Chalcidoidea	Eulophidae	Eulophidae	sp.	KR896848.1
Hymenoptera	Chalcidoidea	Eulophidae	Eulophidae	sp.	KR897132.1
Hymenoptera	Chalcidoidea	Eulophidae	Eulophidae	sp.	KR897610.1
Hymenoptera	Chalcidoidea	Eulophidae	Eulophidae	sp.	KR897763.1
Hymenoptera	Chalcidoidea	Eulophidae	Eulophidae	sp.	KR897796.1
Hymenoptera	Chalcidoidea	Eulophidae	Eulophidae	sp.	KR898112.1
Hymenoptera	Chalcidoidea	Eulophidae	Eulophidae	sp.	KR898165.1
Hymenoptera	Chalcidoidea	Eulophidae	Eulophidae	sp.	KR898425.1
Hymenoptera	Chalcidoidea	Eulophidae	Eulophidae	sp.	KR898463.1
Hymenoptera	Chalcidoidea	Eulophidae	Eulophidae	sp.	KR898553.1

Hymenoptera	Chalcidoidea	Eulophidae	Eulophidae	sp.	KR898865.1
Hymenoptera	Chalcidoidea	Eulophidae	Eulophidae	sp.	KR898969.1
Hymenoptera	Chalcidoidea	Eulophidae	Eulophidae	sp.	KR899071.1
Hymenoptera	Chalcidoidea	Eulophidae	Eulophidae	sp.	KR899108.1
Hymenoptera	Chalcidoidea	Eulophidae	Eulophidae	sp.	KR899211.1
Hymenoptera	Chalcidoidea	Eulophidae	Eulophidae	sp.	KR899355.1
Hymenoptera	Chalcidoidea	Eulophidae	Eulophidae	sp.	KR899389.1
Hymenoptera	Chalcidoidea	Eulophidae	Eulophidae	sp.	KR899419.1
Hymenoptera	Chalcidoidea	Eulophidae	Eulophidae	sp.	KR899432.1
Hymenoptera	Chalcidoidea	Eulophidae	Eulophidae	sp.	KR899676.1
Hymenoptera	Chalcidoidea	Eulophidae	Eulophidae	sp.	KR899721.1
Hymenoptera	Chalcidoidea	Eulophidae	Eulophidae	sp.	KR899920.1
Hymenoptera	Chalcidoidea	Eulophidae	Eulophidae	sp.	KR899975.1
Hymenoptera	Chalcidoidea	Eulophidae	Eulophidae	sp.	KR900159.1
Hymenoptera	Chalcidoidea	Eulophidae	Eulophidae	sp.	KR900294.1
Hymenoptera	Chalcidoidea	Eulophidae	Eulophidae	sp.	KR900534.1
Hymenoptera	Chalcidoidea	Eulophidae	Eulophidae	sp.	KR900547.1
Hymenoptera	Chalcidoidea	Eulophidae	Eulophidae	sp.	KR900641.1
Hymenoptera	Chalcidoidea	Eulophidae	Eulophidae	sp.	KR901086.1
Hymenoptera	Chalcidoidea	Eulophidae	Eulophidae	sp.	KR901196.1
Hymenoptera	Chalcidoidea	Eulophidae	Eulophidae	sp.	KR901524.1
Hymenoptera	Chalcidoidea	Eulophidae	Eulophidae	sp.	KR901696.1
Hymenoptera	Chalcidoidea	Eulophidae	Eulophidae	sp.	KR901744.1
Hymenoptera	Chalcidoidea	Eulophidae	Eulophidae	sp.	KR901898.1
Hymenoptera	Chalcidoidea	Eulophidae	Eulophidae	sp.	KR902021.1
Hymenoptera	Chalcidoidea	Eulophidae	Eulophidae	sp.	KR902265.1
Hymenoptera	Chalcidoidea	Eulophidae	Eulophidae	sp.	KR902269.1
Hymenoptera	Chalcidoidea	Eulophidae	Eulophidae	sp.	KR924000.1
Hymenoptera	Chalcidoidea	Eulophidae	Eulophidae	sp.	KR924547.1
Hymenoptera	Chalcidoidea	Eulophidae	Eulophidae	sp.	KR924908.1
Hymenoptera	Chalcidoidea	Eulophidae	Eulophidae	sp.	KR925336.1
Hymenoptera	Chalcidoidea	Eulophidae	Eulophidae	sp.	KR925931.1
Hymenoptera	Chalcidoidea	Eulophidae	Eulophidae	sp.	KR925987.1
Hymenoptera	Chalcidoidea	Eulophidae	Eulophidae	sp.	KR926978.1
Hymenoptera	Chalcidoidea	Eulophidae	Eulophidae	sp.	KR927385.1
Hymenoptera	Chalcidoidea	Eulophidae	Eulophidae	sp.	KR927425.1
Hymenoptera	Chalcidoidea	Eulophidae	Eulophidae	sp.	KR927551.1
Hymenoptera	Chalcidoidea	Eulophidae	Eulophidae	sp.	KR927955.1
Hymenoptera	Chalcidoidea	Eulophidae	Eulophidae	sp.	KR928761.1
Hymenoptera	Chalcidoidea	Eulophidae	Eulophidae	sp.	KR929844.1
Hymenoptera	Chalcidoidea	Eulophidae	Eulophidae	sp.	KR929980.1
Hymenoptera	Chalcidoidea	Eulophidae	Eulophidae	sp.	KR930779.1
Hymenoptera	Chalcidoidea	Eulophidae	Eulophidae	sp.	KR931027.1
Hymenoptera	Chalcidoidea	Eulophidae	Eulophidae	sp.	KR932377.1
Hymenoptera	Chalcidoidea	Eulophidae	Eulophidae	sp.	KR934156.1
Hymenoptera	Chalcidoidea	Eulophidae	Eulophidae	sp.	KR934832.1
Hymenoptera	Chalcidoidea	Eulophidae	Eulophidae	sp.	KT623046.1
Hymenoptera	Chalcidoidea	Eulophidae	Eulophidae	sp.	KT705403.1
Hymenoptera	Chalcidoidea	Eulophidae	Eulophidae	sp.	KT705768.1
Hymenoptera	Chalcidoidea	Eulophidae	Eulophidae	sp.	KT705794.1
Hymenoptera	Chalcidoidea	Eulophidae	Eulophidae	sp.	KT706406.1
Hymenoptera	Chalcidoidea	Eulophidae	Eulophidae	sp.	KT706426.1
Hymenoptera	Chalcidoidea	Eulophidae	Eulophidae	sp.	KT707190.1
Hymenoptera	Chalcidoidea	Eulophidae	Eulophidae	sp.	KT707614.1

Hymenoptera	Chalcidoidea	Eulophidae	Eulophidae	sp.	KT707676.1
Hymenoptera	Chalcidoidea	Eulophidae	Eulophidae	sp.	KT708537.1
Hymenoptera	Chalcidoidea	Eulophidae	Eulophidae	sp.	KU873377.1
Hymenoptera	Chalcidoidea	Eulophidae	Eulophidae	sp.	KU873379.1
Hymenoptera	Chalcidoidea	Eulophidae	Eulophidae	sp.	KU873380.1
Hymenoptera	Chalcidoidea	Eulophidae	Eulophidae	sp.	KX053200.1
Hymenoptera	Chalcidoidea	Eulophidae	Eulophidae	sp.	KX053202.1
Hymenoptera	Chalcidoidea	Eulophidae	Eulophidae	sp.	KX535032.1
Hymenoptera	Chalcidoidea	Eulophidae	Eulophidae	sp.	KX535144.1
Hymenoptera	Chalcidoidea	Eulophidae	Eulophidae	sp.	KY829644.1
Hymenoptera	Chalcidoidea	Eulophidae	Eulophidae	sp.	KY829694.1
Hymenoptera	Chalcidoidea	Eulophidae	Eulophidae	sp.	KY829741.1
Hymenoptera	Chalcidoidea	Eulophidae	Eulophidae	sp.	KY830321.1
Hymenoptera	Chalcidoidea	Eulophidae	Eulophidae	sp.	KY830835.1
Hymenoptera	Chalcidoidea	Eulophidae	Eulophidae	sp.	KY830896.1
Hymenoptera	Chalcidoidea	Eulophidae	Eulophidae	sp.	KY831124.1
Hymenoptera	Chalcidoidea	Eulophidae	Eulophidae	sp.	KY831547.1
Hymenoptera	Chalcidoidea	Eulophidae	Eulophidae	sp.	KY831748.1
Hymenoptera	Chalcidoidea	Eulophidae	Eulophidae	sp.	KY831913.1
Hymenoptera	Chalcidoidea	Eulophidae	Eulophidae	sp.	KY832534.1
Hymenoptera	Chalcidoidea	Eulophidae	Eulophidae	sp.	KY833632.1
Hymenoptera	Chalcidoidea	Eulophidae	Eulophidae	sp.	KY833797.1
Hymenoptera	Chalcidoidea	Eulophidae	Eulophidae	sp.	KY834088.1
Hymenoptera	Chalcidoidea	Eulophidae	Eulophidae	sp.	KY834105.1
Hymenoptera	Chalcidoidea	Eulophidae	Eulophidae	sp.	KY834209.1
Hymenoptera	Chalcidoidea	Eulophidae	Eulophidae	sp.	KY834258.1
Hymenoptera	Chalcidoidea	Eulophidae	Eulophidae	sp.	KY834298.1
Hymenoptera	Chalcidoidea	Eulophidae	Eulophidae	sp.	KY834531.1
Hymenoptera	Chalcidoidea	Eulophidae	Eulophidae	sp.	KY834917.1
Hymenoptera	Chalcidoidea	Eulophidae	Eulophidae	sp.	KY835057.1
Hymenoptera	Chalcidoidea	Eulophidae	Eulophidae	sp.	KY835123.1
Hymenoptera	Chalcidoidea	Eulophidae	Eulophidae	sp.	KY835168.1
Hymenoptera	Chalcidoidea	Eulophidae	Eulophidae	sp.	KY835244.1
Hymenoptera	Chalcidoidea	Eulophidae	Eulophidae	sp.	KY835709.1
Hymenoptera	Chalcidoidea	Eulophidae	Eulophidae	sp.	KY836464.1
Hymenoptera	Chalcidoidea	Eulophidae	Eulophidae	sp.	KY836486.1
Hymenoptera	Chalcidoidea	Eulophidae	Eulophidae	sp.	KY836833.1
Hymenoptera	Chalcidoidea	Eulophidae	Eulophidae	sp.	KY836934.1
Hymenoptera	Chalcidoidea	Eulophidae	Eulophidae	sp.	KY837217.1
Hymenoptera	Chalcidoidea	Eulophidae	Eulophidae	sp.	KY837338.1
Hymenoptera	Chalcidoidea	Eulophidae	Eulophidae	sp.	KY837644.1
Hymenoptera	Chalcidoidea	Eulophidae	Eulophidae	sp.	KY837728.1
Hymenoptera	Chalcidoidea	Eulophidae	Eulophidae	sp.	KY838019.1
Hymenoptera	Chalcidoidea	Eulophidae	Eulophidae	sp.	KY838027.1
Hymenoptera	Chalcidoidea	Eulophidae	Eulophidae	sp.	KY838100.1
Hymenoptera	Chalcidoidea	Eulophidae	Eulophidae	sp.	KY838191.1
Hymenoptera	Chalcidoidea	Eulophidae	Eulophidae	sp.	KY838302.1
Hymenoptera	Chalcidoidea	Eulophidae	Eulophidae	sp.	KY838663.1
Hymenoptera	Chalcidoidea	Eulophidae	Eulophidae	sp.	KY838966.1
Hymenoptera	Chalcidoidea	Eulophidae	Eulophidae	sp.	KY838977.1
Hymenoptera	Chalcidoidea	Eulophidae	Eulophidae	sp.	KY839346.1
Hymenoptera	Chalcidoidea	Eulophidae	Eulophidae	sp.	KY839405.1
Hymenoptera	Chalcidoidea	Eulophidae	Eulophidae	sp.	KY839449.1
Hymenoptera	Chalcidoidea	Eulophidae	Eulophidae	sp.	KY839660.1

Hymenoptera	Chalcidoidea	Eulophidae	Eulophidae	sp.	KY840506.1
Hymenoptera	Chalcidoidea	Eulophidae	Eulophidae	sp.	KY840553.1
Hymenoptera	Chalcidoidea	Eulophidae	Eulophidae	sp.	KY841426.1
Hymenoptera	Chalcidoidea	Eulophidae	Eulophidae	sp.	KY841447.1
Hymenoptera	Chalcidoidea	Eulophidae	Eulophidae	sp.	KY841516.1
Hymenoptera	Chalcidoidea	Eulophidae	Eulophidae	sp.	KY841732.1
Hymenoptera	Chalcidoidea	Eulophidae	Eulophidae	sp.	KY841751.1
Hymenoptera	Chalcidoidea	Eulophidae	Eulophidae	sp.	KY841799.1
Hymenoptera	Chalcidoidea	Eulophidae	Eulophidae	sp.	KY842904.1
Hymenoptera	Chalcidoidea	Eulophidae	Eulophidae	sp.	KY843276.1
Hymenoptera	Chalcidoidea	Eulophidae	Eulophidae	sp.	KY843283.1
Hymenoptera	Chalcidoidea	Eulophidae	Eulophidae	sp.	KY843369.1
Hymenoptera	Chalcidoidea	Eulophidae	Eulophidae	sp.	KY843478.1
Hymenoptera	Chalcidoidea	Eulophidae	Eulophidae	sp.	KY843757.1
Hymenoptera	Chalcidoidea	Eulophidae	Eulophidae	sp.	KY843778.1
Hymenoptera	Chalcidoidea	Eulophidae	Eulophidae	sp.	KY843834.1
Hymenoptera	Chalcidoidea	Eulophidae	Eulophidae	sp.	KY843952.1
Hymenoptera	Chalcidoidea	Eulophidae	Eulophidae	sp.	KY844150.1
Hymenoptera	Chalcidoidea	Eulophidae	Eulophidae	sp.	KY844517.1
Hymenoptera	Chalcidoidea	Eulophidae	Eulophidae	sp.	KY844651.1
Hymenoptera	Chalcidoidea	Eulophidae	Eulophidae	sp.	KY844759.1
Hymenoptera	Chalcidoidea	Eulophidae	Eulophidae	sp.	KY844910.1
Hymenoptera	Chalcidoidea	Eulophidae	Eulophidae	sp.	KY844982.1
Hymenoptera	Chalcidoidea	Eulophidae	Eulophidae	sp.	KY845043.1
Hymenoptera	Chalcidoidea	Eulophidae	Eulophidae	sp.	KY845466.1
Hymenoptera	Chalcidoidea	Eulophidae	Eulophidae	sp.	KY845489.1
Hymenoptera	Chalcidoidea	Eulophidae	Eulophidae	sp.	KY846002.1
Hymenoptera	Chalcidoidea	Eulophidae	Eulophidae	sp.	KY846090.1
Hymenoptera	Chalcidoidea	Eulophidae	Eulophidae	sp.	KY846380.1
Hymenoptera	Chalcidoidea	Eulophidae	Eulophidae	sp.	KY846480.1
Hymenoptera	Chalcidoidea	Eulophidae	Eulophidae	sp.	KY846554.1
Hymenoptera	Chalcidoidea	Eulophidae	Eulophidae	sp.	KY846689.1
Hymenoptera	Chalcidoidea	Eulophidae	Eulophidae	sp.	KY847122.1
Hymenoptera	Chalcidoidea	Eulophidae	Eulophidae	sp.	KY847187.1
Hymenoptera	Chalcidoidea	Eulophidae	Eulophidae	sp.	KY847419.1
Hymenoptera	Chalcidoidea	Eulophidae	Eulophidae	sp.	LC505634.1
Hymenoptera	Chalcidoidea	Eulophidae	Eulophidae	sp.	MF898500.1
Hymenoptera	Chalcidoidea	Eulophidae	Eulophidae	sp.	MF898549.1
Hymenoptera	Chalcidoidea	Eulophidae	Eulophidae	sp.	MF898906.1
Hymenoptera	Chalcidoidea	Eulophidae	Eulophidae	sp.	MF899024.1
Hymenoptera	Chalcidoidea	Eulophidae	Eulophidae	sp.	MF899032.1
Hymenoptera	Chalcidoidea	Eulophidae	Eulophidae	sp.	MF899114.1
Hymenoptera	Chalcidoidea	Eulophidae	Eulophidae	sp.	MF899167.1
Hymenoptera	Chalcidoidea	Eulophidae	Eulophidae	sp.	MF899219.1
Hymenoptera	Chalcidoidea	Eulophidae	Eulophidae	sp.	MF899274.1
Hymenoptera	Chalcidoidea	Eulophidae	Eulophidae	sp.	MF899315.1
Hymenoptera	Chalcidoidea	Eulophidae	Eulophidae	sp.	MF899412.1
Hymenoptera	Chalcidoidea	Eulophidae	Eulophidae	sp.	MF899616.1
Hymenoptera	Chalcidoidea	Eulophidae	Eulophidae	sp.	MF899638.1
Hymenoptera	Chalcidoidea	Eulophidae	Eulophidae	sp.	MF899673.1
Hymenoptera	Chalcidoidea	Eulophidae	Eulophidae	sp.	MF899926.1
Hymenoptera	Chalcidoidea	Eulophidae	Eulophidae	sp.	MF900023.1
Hymenoptera	Chalcidoidea	Eulophidae	Eulophidae	sp.	MF900102.1
Hymenoptera	Chalcidoidea	Eulophidae	Eulophidae	sp.	MF900229.1

Hymenoptera	Chalcidoidea	Eulophidae	Eulophidae	sp.	MF900273.1
Hymenoptera	Chalcidoidea	Eulophidae	Eulophidae	sp.	MF900395.1
Hymenoptera	Chalcidoidea	Eulophidae	Eulophidae	sp.	MF900512.1
Hymenoptera	Chalcidoidea	Eulophidae	Eulophidae	sp.	MF900554.1
Hymenoptera	Chalcidoidea	Eulophidae	Eulophidae	sp.	MF900712.1
Hymenoptera	Chalcidoidea	Eulophidae	Eulophidae	sp.	MF900716.1
Hymenoptera	Chalcidoidea	Eulophidae	Eulophidae	sp.	MF900740.1
Hymenoptera	Chalcidoidea	Eulophidae	Eulophidae	sp.	MF900788.1
Hymenoptera	Chalcidoidea	Eulophidae	Eulophidae	sp.	MF900842.1
Hymenoptera	Chalcidoidea	Eulophidae	Eulophidae	sp.	MF900869.1
Hymenoptera	Chalcidoidea	Eulophidae	Eulophidae	sp.	MF900958.1
Hymenoptera	Chalcidoidea	Eulophidae	Eulophidae	sp.	MF901178.1
Hymenoptera	Chalcidoidea	Eulophidae	Eulophidae	sp.	MF901188.1
Hymenoptera	Chalcidoidea	Eulophidae	Eulophidae	sp.	MF901251.1
Hymenoptera	Chalcidoidea	Eulophidae	Eulophidae	sp.	MF901277.1
Hymenoptera	Chalcidoidea	Eulophidae	Eulophidae	sp.	MF901365.1
Hymenoptera	Chalcidoidea	Eulophidae	Eulophidae	sp.	MF901367.1
Hymenoptera	Chalcidoidea	Eulophidae	Eulophidae	sp.	MF901414.1
Hymenoptera	Chalcidoidea	Eulophidae	Eulophidae	sp.	MF901435.1
Hymenoptera	Chalcidoidea	Eulophidae	Eulophidae	sp.	MF901550.1
Hymenoptera	Chalcidoidea	Eulophidae	Eulophidae	sp.	MF901564.1
Hymenoptera	Chalcidoidea	Eulophidae	Eulophidae	sp.	MF901603.1
Hymenoptera	Chalcidoidea	Eulophidae	Eulophidae	sp.	MF901646.1
Hymenoptera	Chalcidoidea	Eulophidae	Eulophidae	sp.	MF901689.1
Hymenoptera	Chalcidoidea	Eulophidae	Eulophidae	sp.	MF901792.1
Hymenoptera	Chalcidoidea	Eulophidae	Eulophidae	sp.	MF901883.1
Hymenoptera	Chalcidoidea	Eulophidae	Eulophidae	sp.	MF901891.1
Hymenoptera	Chalcidoidea	Eulophidae	Eulophidae	sp.	MF902021.1
Hymenoptera	Chalcidoidea	Eulophidae	Eulophidae	sp.	MF902161.1
Hymenoptera	Chalcidoidea	Eulophidae	Eulophidae	sp.	MF902179.1
Hymenoptera	Chalcidoidea	Eulophidae	Eulophidae	sp.	MF902223.1
Hymenoptera	Chalcidoidea	Eulophidae	Eulophidae	sp.	MF902271.1
Hymenoptera	Chalcidoidea	Eulophidae	Eulophidae	sp.	MF902589.1
Hymenoptera	Chalcidoidea	Eulophidae	Eulophidae	sp.	MF902657.1
Hymenoptera	Chalcidoidea	Eulophidae	Eulophidae	sp.	MF902796.1
Hymenoptera	Chalcidoidea	Eulophidae	Eulophidae	sp.	MF902947.1
Hymenoptera	Chalcidoidea	Eulophidae	Eulophidae	sp.	MF903156.1
Hymenoptera	Chalcidoidea	Eulophidae	Eulophidae	sp.	MF903174.1
Hymenoptera	Chalcidoidea	Eulophidae	Eulophidae	sp.	MF903253.1
Hymenoptera	Chalcidoidea	Eulophidae	Eulophidae	sp.	MF903317.1
Hymenoptera	Chalcidoidea	Eulophidae	Eulophidae	sp.	MF903373.1
Hymenoptera	Chalcidoidea	Eulophidae	Eulophidae	sp.	MF903459.1
Hymenoptera	Chalcidoidea	Eulophidae	Eulophidae	sp.	MF903539.1
Hymenoptera	Chalcidoidea	Eulophidae	Eulophidae	sp.	MF903558.1
Hymenoptera	Chalcidoidea	Eulophidae	Eulophidae	sp.	MF903568.1
Hymenoptera	Chalcidoidea	Eulophidae	Eulophidae	sp.	MF903609.1
Hymenoptera	Chalcidoidea	Eulophidae	Eulophidae	sp.	MF903672.1
Hymenoptera	Chalcidoidea	Eulophidae	Eulophidae	sp.	MF903827.1
Hymenoptera	Chalcidoidea	Eulophidae	Eulophidae	sp.	MF903857.1
Hymenoptera	Chalcidoidea	Eulophidae	Eulophidae	sp.	MF903907.1
Hymenoptera	Chalcidoidea	Eulophidae	Eulophidae	sp.	MF903934.1
Hymenoptera	Chalcidoidea	Eulophidae	Eulophidae	sp.	MF903966.1
Hymenoptera	Chalcidoidea	Eulophidae	Eulophidae	sp.	MF903974.1
Hymenoptera	Chalcidoidea	Eulophidae	Eulophidae	sp.	MF904068.1

Hymenoptera	Chalcidoidea	Eulophidae	Eulophidae	sp.	MF904096.1
Hymenoptera	Chalcidoidea	Eulophidae	Eulophidae	sp.	MF904233.1
Hymenoptera	Chalcidoidea	Eulophidae	Eulophidae	sp.	MF904290.1
Hymenoptera	Chalcidoidea	Eulophidae	Eulophidae	sp.	MF904427.1
Hymenoptera	Chalcidoidea	Eulophidae	Eulophidae	sp.	MF904511.1
Hymenoptera	Chalcidoidea	Eulophidae	Eulophidae	sp.	MF904520.1
Hymenoptera	Chalcidoidea	Eulophidae	Eulophidae	sp.	MF904701.1
Hymenoptera	Chalcidoidea	Eulophidae	Eulophidae	sp.	MF904737.1
Hymenoptera	Chalcidoidea	Eulophidae	Eulophidae	sp.	MF904740.1
Hymenoptera	Chalcidoidea	Eulophidae	Eulophidae	sp.	MF904952.1
Hymenoptera	Chalcidoidea	Eulophidae	Eulophidae	sp.	MF904990.1
Hymenoptera	Chalcidoidea	Eulophidae	Eulophidae	sp.	MF905043.1
Hymenoptera	Chalcidoidea	Eulophidae	Eulophidae	sp.	MF905370.1
Hymenoptera	Chalcidoidea	Eulophidae	Eulophidae	sp.	MF905502.1
Hymenoptera	Chalcidoidea	Eulophidae	Eulophidae	sp.	MF905511.1
Hymenoptera	Chalcidoidea	Eulophidae	Eulophidae	sp.	MF905796.1
Hymenoptera	Chalcidoidea	Eulophidae	Eulophidae	sp.	MF905828.1
Hymenoptera	Chalcidoidea	Eulophidae	Eulophidae	sp.	MF905882.1
Hymenoptera	Chalcidoidea	Eulophidae	Eulophidae	sp.	MF905938.1
Hymenoptera	Chalcidoidea	Eulophidae	Eulophidae	sp.	MF905954.1
Hymenoptera	Chalcidoidea	Eulophidae	Eulophidae	sp.	MF905959.1
Hymenoptera	Chalcidoidea	Eulophidae	Eulophidae	sp.	MF906072.1
Hymenoptera	Chalcidoidea	Eulophidae	Eulophidae	sp.	MF906123.1
Hymenoptera	Chalcidoidea	Eulophidae	Eulophidae	sp.	MF906371.1
Hymenoptera	Chalcidoidea	Eulophidae	Eulophidae	sp.	MF906388.1
Hymenoptera	Chalcidoidea	Eulophidae	Eulophidae	sp.	MF906462.1
Hymenoptera	Chalcidoidea	Eulophidae	Eulophidae	sp.	MF906483.1
Hymenoptera	Chalcidoidea	Eulophidae	Eulophidae	sp.	MF906518.1
Hymenoptera	Chalcidoidea	Eulophidae	Eulophidae	sp.	MF906529.1
Hymenoptera	Chalcidoidea	Eulophidae	Eulophidae	sp.	MF906580.1
Hymenoptera	Chalcidoidea	Eulophidae	Eulophidae	sp.	MF906704.1
Hymenoptera	Chalcidoidea	Eulophidae	Eulophidae	sp.	MF906743.1
Hymenoptera	Chalcidoidea	Eulophidae	Eulophidae	sp.	MF906760.1
Hymenoptera	Chalcidoidea	Eulophidae	Eulophidae	sp.	MF906831.1
Hymenoptera	Chalcidoidea	Eulophidae	Eulophidae	sp.	MF906892.1
Hymenoptera	Chalcidoidea	Eulophidae	Eulophidae	sp.	MF907152.1
Hymenoptera	Chalcidoidea	Eulophidae	Eulophidae	sp.	MF907293.1
Hymenoptera	Chalcidoidea	Eulophidae	Eulophidae	sp.	MF907358.1
Hymenoptera	Chalcidoidea	Eulophidae	Eulophidae	sp.	MF907430.1
Hymenoptera	Chalcidoidea	Eulophidae	Eulophidae	sp.	MF907652.1
Hymenoptera	Chalcidoidea	Eulophidae	Eulophidae	sp.	MF907667.1
Hymenoptera	Chalcidoidea	Eulophidae	Eulophidae	sp.	MF907772.1
Hymenoptera	Chalcidoidea	Eulophidae	Eulophidae	sp.	MF907860.1
Hymenoptera	Chalcidoidea	Eulophidae	Eulophidae	sp.	MF907953.1
Hymenoptera	Chalcidoidea	Eulophidae	Eulophidae	sp.	MF907978.1
Hymenoptera	Chalcidoidea	Eulophidae	Eulophidae	sp.	MF908074.1
Hymenoptera	Chalcidoidea	Eulophidae	Eulophidae	sp.	MF908124.1
Hymenoptera	Chalcidoidea	Eulophidae	Eulophidae	sp.	MF908172.1
Hymenoptera	Chalcidoidea	Eulophidae	Eulophidae	sp.	MG334696.1
Hymenoptera	Chalcidoidea	Eulophidae	Eulophidae	sp.	MG334748.1
Hymenoptera	Chalcidoidea	Eulophidae	Eulophidae	sp.	MG334777.1
Hymenoptera	Chalcidoidea	Eulophidae	Eulophidae	sp.	MG334884.1
Hymenoptera	Chalcidoidea	Eulophidae	Eulophidae	sp.	MG334953.1
Hymenoptera	Chalcidoidea	Eulophidae	Eulophidae	sp.	MG335018.1

Hymenoptera	Chalcidoidea	Eulophidae	Eulophidae	sp.	MG335096.1
Hymenoptera	Chalcidoidea	Eulophidae	Eulophidae	sp.	MG335170.1
Hymenoptera	Chalcidoidea	Eulophidae	Eulophidae	sp.	MG335291.1
Hymenoptera	Chalcidoidea	Eulophidae	Eulophidae	sp.	MG335507.1
Hymenoptera	Chalcidoidea	Eulophidae	Eulophidae	sp.	MG335552.1
Hymenoptera	Chalcidoidea	Eulophidae	Eulophidae	sp.	MG335579.1
Hymenoptera	Chalcidoidea	Eulophidae	Eulophidae	sp.	MG335607.1
Hymenoptera	Chalcidoidea	Eulophidae	Eulophidae	sp.	MG335641.1
Hymenoptera	Chalcidoidea	Eulophidae	Eulophidae	sp.	MG335732.1
Hymenoptera	Chalcidoidea	Eulophidae	Eulophidae	sp.	MG335905.1
Hymenoptera	Chalcidoidea	Eulophidae	Eulophidae	sp.	MG335933.1
Hymenoptera	Chalcidoidea	Eulophidae	Eulophidae	sp.	MG335942.1
Hymenoptera	Chalcidoidea	Eulophidae	Eulophidae	sp.	MG336031.1
Hymenoptera	Chalcidoidea	Eulophidae	Eulophidae	sp.	MG336314.1
Hymenoptera	Chalcidoidea	Eulophidae	Eulophidae	sp.	MG336445.1
Hymenoptera	Chalcidoidea	Eulophidae	Eulophidae	sp.	MG336933.1
Hymenoptera	Chalcidoidea	Eulophidae	Eulophidae	sp.	MG337047.1
Hymenoptera	Chalcidoidea	Eulophidae	Eulophidae	sp.	MG337170.1
Hymenoptera	Chalcidoidea	Eulophidae	Eulophidae	sp.	MG337190.1
Hymenoptera	Chalcidoidea	Eulophidae	Eulophidae	sp.	MG337198.1
Hymenoptera	Chalcidoidea	Eulophidae	Eulophidae	sp.	MG337225.1
Hymenoptera	Chalcidoidea	Eulophidae	Eulophidae	sp.	MG337297.1
Hymenoptera	Chalcidoidea	Eulophidae	Eulophidae	sp.	MG337305.1
Hymenoptera	Chalcidoidea	Eulophidae	Eulophidae	sp.	MG337310.1
Hymenoptera	Chalcidoidea	Eulophidae	Eulophidae	sp.	MG337337.1
Hymenoptera	Chalcidoidea	Eulophidae	Eulophidae	sp.	MG337459.1
Hymenoptera	Chalcidoidea	Eulophidae	Eulophidae	sp.	MG337501.1
Hymenoptera	Chalcidoidea	Eulophidae	Eulophidae	sp.	MG337513.1
Hymenoptera	Chalcidoidea	Eulophidae	Eulophidae	sp.	MG337547.1
Hymenoptera	Chalcidoidea	Eulophidae	Eulophidae	sp.	MG337602.1
Hymenoptera	Chalcidoidea	Eulophidae	Eulophidae	sp.	MG337772.1
Hymenoptera	Chalcidoidea	Eulophidae	Eulophidae	sp.	MG337905.1
Hymenoptera	Chalcidoidea	Eulophidae	Eulophidae	sp.	MG337929.1
Hymenoptera	Chalcidoidea	Eulophidae	Eulophidae	sp.	MG338068.1
Hymenoptera	Chalcidoidea	Eulophidae	Eulophidae	sp.	MG338147.1
Hymenoptera	Chalcidoidea	Eulophidae	Eulophidae	sp.	MG338392.1
Hymenoptera	Chalcidoidea	Eulophidae	Eulophidae	sp.	MG338441.1
Hymenoptera	Chalcidoidea	Eulophidae	Eulophidae	sp.	MG338507.1
Hymenoptera	Chalcidoidea	Eulophidae	Eulophidae	sp.	MG338741.1
Hymenoptera	Chalcidoidea	Eulophidae	Eulophidae	sp.	MG338923.1
Hymenoptera	Chalcidoidea	Eulophidae	Eulophidae	sp.	MG338937.1
Hymenoptera	Chalcidoidea	Eulophidae	Eulophidae	sp.	MG338969.1
Hymenoptera	Chalcidoidea	Eulophidae	Eulophidae	sp.	MG339056.1
Hymenoptera	Chalcidoidea	Eulophidae	Eulophidae	sp.	MG339063.1
Hymenoptera	Chalcidoidea	Eulophidae	Eulophidae	sp.	MG339077.1
Hymenoptera	Chalcidoidea	Eulophidae	Eulophidae	sp.	MG339096.1
Hymenoptera	Chalcidoidea	Eulophidae	Eulophidae	sp.	MG339260.1
Hymenoptera	Chalcidoidea	Eulophidae	Eulophidae	sp.	MG339321.1
Hymenoptera	Chalcidoidea	Eulophidae	Eulophidae	sp.	MG339469.1
Hymenoptera	Chalcidoidea	Eulophidae	Eulophidae	sp.	MG339534.1
Hymenoptera	Chalcidoidea	Eulophidae	Eulophidae	sp.	MG339663.1
Hymenoptera	Chalcidoidea	Eulophidae	Eulophidae	sp.	MG339706.1
Hymenoptera	Chalcidoidea	Eulophidae	Eulophidae	sp.	MG339740.1
Hymenoptera	Chalcidoidea	Eulophidae	Eulophidae	sp.	MG339806.1

Hymenoptera	Chalcidoidea	Eulophidae	Eulophidae	sp.	MG339895.1
Hymenoptera	Chalcidoidea	Eulophidae	Eulophidae	sp.	MG339898.1
Hymenoptera	Chalcidoidea	Eulophidae	Eulophidae	sp.	MG339934.1
Hymenoptera	Chalcidoidea	Eulophidae	Eulophidae	sp.	MG340007.1
Hymenoptera	Chalcidoidea	Eulophidae	Eulophidae	sp.	MG340063.1
Hymenoptera	Chalcidoidea	Eulophidae	Eulophidae	sp.	MG340242.1
Hymenoptera	Chalcidoidea	Eulophidae	Eulophidae	sp.	MG340259.1
Hymenoptera	Chalcidoidea	Eulophidae	Eulophidae	sp.	MG340481.1
Hymenoptera	Chalcidoidea	Eulophidae	Eulophidae	sp.	MG340492.1
Hymenoptera	Chalcidoidea	Eulophidae	Eulophidae	sp.	MG340535.1
Hymenoptera	Chalcidoidea	Eulophidae	Eulophidae	sp.	MG340794.1
Hymenoptera	Chalcidoidea	Eulophidae	Eulophidae	sp.	MG340916.1
Hymenoptera	Chalcidoidea	Eulophidae	Eulophidae	sp.	MG340920.1
Hymenoptera	Chalcidoidea	Eulophidae	Eulophidae	sp.	MG340947.1
Hymenoptera	Chalcidoidea	Eulophidae	Eulophidae	sp.	MG341000.1
Hymenoptera	Chalcidoidea	Eulophidae	Eulophidae	sp.	MG341065.1
Hymenoptera	Chalcidoidea	Eulophidae	Eulophidae	sp.	MG341163.1
Hymenoptera	Chalcidoidea	Eulophidae	Eulophidae	sp.	MG341262.1
Hymenoptera	Chalcidoidea	Eulophidae	Eulophidae	sp.	MG341312.1
Hymenoptera	Chalcidoidea	Eulophidae	Eulophidae	sp.	MG341604.1
Hymenoptera	Chalcidoidea	Eulophidae	Eulophidae	sp.	MG341807.1
Hymenoptera	Chalcidoidea	Eulophidae	Eulophidae	sp.	MG341836.1
Hymenoptera	Chalcidoidea	Eulophidae	Eulophidae	sp.	MG341843.1
Hymenoptera	Chalcidoidea	Eulophidae	Eulophidae	sp.	MG342018.1
Hymenoptera	Chalcidoidea	Eulophidae	Eulophidae	sp.	MG342088.1
Hymenoptera	Chalcidoidea	Eulophidae	Eulophidae	sp.	MG342144.1
Hymenoptera	Chalcidoidea	Eulophidae	Eulophidae	sp.	MG342170.1
Hymenoptera	Chalcidoidea	Eulophidae	Eulophidae	sp.	MG342190.1
Hymenoptera	Chalcidoidea	Eulophidae	Eulophidae	sp.	MG342442.1
Hymenoptera	Chalcidoidea	Eulophidae	Eulophidae	sp.	MG342465.1
Hymenoptera	Chalcidoidea	Eulophidae	Eulophidae	sp.	MG342484.1
Hymenoptera	Chalcidoidea	Eulophidae	Eulophidae	sp.	MG342512.1
Hymenoptera	Chalcidoidea	Eulophidae	Eulophidae	sp.	MG342526.1
Hymenoptera	Chalcidoidea	Eulophidae	Eulophidae	sp.	MG342538.1
Hymenoptera	Chalcidoidea	Eulophidae	Eulophidae	sp.	MG342539.1
Hymenoptera	Chalcidoidea	Eulophidae	Eulophidae	sp.	MG342580.1
Hymenoptera	Chalcidoidea	Eulophidae	Eulophidae	sp.	MG342809.1
Hymenoptera	Chalcidoidea	Eulophidae	Eulophidae	sp.	MG343092.1
Hymenoptera	Chalcidoidea	Eulophidae	Eulophidae	sp.	MG343093.1
Hymenoptera	Chalcidoidea	Eulophidae	Eulophidae	sp.	MG343155.1
Hymenoptera	Chalcidoidea	Eulophidae	Eulophidae	sp.	MG343165.1
Hymenoptera	Chalcidoidea	Eulophidae	Eulophidae	sp.	MG343400.1
Hymenoptera	Chalcidoidea	Eulophidae	Eulophidae	sp.	MG343420.1
Hymenoptera	Chalcidoidea	Eulophidae	Eulophidae	sp.	MG343474.1
Hymenoptera	Chalcidoidea	Eulophidae	Eulophidae	sp.	MG343496.1
Hymenoptera	Chalcidoidea	Eulophidae	Eulophidae	sp.	MG343689.1
Hymenoptera	Chalcidoidea	Eulophidae	Eulophidae	sp.	MG343996.1
Hymenoptera	Chalcidoidea	Eulophidae	Eulophidae	sp.	MG344085.1
Hymenoptera	Chalcidoidea	Eulophidae	Eulophidae	sp.	MG344161.1
Hymenoptera	Chalcidoidea	Eulophidae	Eulophidae	sp.	MG344210.1
Hymenoptera	Chalcidoidea	Eulophidae	Eulophidae	sp.	MG344232.1
Hymenoptera	Chalcidoidea	Eulophidae	Eulophidae	sp.	MG344296.1
Hymenoptera	Chalcidoidea	Eulophidae	Eulophidae	sp.	MG344342.1
Hymenoptera	Chalcidoidea	Eulophidae	Eulophidae	sp.	MG344405.1

Hymenoptera	Chalcidoidea	Eulophidae	Eulophidae	sp.	MG344485.1
Hymenoptera	Chalcidoidea	Eulophidae	Eulophidae	sp.	MG344505.1
Hymenoptera	Chalcidoidea	Eulophidae	Eulophidae	sp.	MG344584.1
Hymenoptera	Chalcidoidea	Eulophidae	Eulophidae	sp.	MG438672.1
Hymenoptera	Chalcidoidea	Eulophidae	Eulophidae	sp.	MG439272.1
Hymenoptera	Chalcidoidea	Eulophidae	Eulophidae	sp.	MG441104.1
Hymenoptera	Chalcidoidea	Eulophidae	Eulophidae	sp.	MG441679.1
Hymenoptera	Chalcidoidea	Eulophidae	Eulophidae	sp.	MG441703.1
Hymenoptera	Chalcidoidea	Eulophidae	Eulophidae	sp.	MG442192.1
Hymenoptera	Chalcidoidea	Eulophidae	Eulophidae	sp.	MG442499.1
Hymenoptera	Chalcidoidea	Eulophidae	Eulophidae	sp.	MG442583.1
Hymenoptera	Chalcidoidea	Eulophidae	Eulophidae	sp.	MG442903.1
Hymenoptera	Chalcidoidea	Eulophidae	Eulophidae	sp.	MG443066.1
Hymenoptera	Chalcidoidea	Eulophidae	Eulophidae	sp.	MG443646.1
Hymenoptera	Chalcidoidea	Eulophidae	Eulophidae	sp.	MG443970.1
Hymenoptera	Chalcidoidea	Eulophidae	Eulophidae	sp.	MG444170.1
Hymenoptera	Chalcidoidea	Eulophidae	Eulophidae	sp.	MG444261.1
Hymenoptera	Chalcidoidea	Eulophidae	Eulophidae	sp.	MG444795.1
Hymenoptera	Chalcidoidea	Eulophidae	Eulophidae	sp.	MG444891.1
Hymenoptera	Chalcidoidea	Eulophidae	Eulophidae	sp.	MG445115.1
Hymenoptera	Chalcidoidea	Eulophidae	Eulophidae	sp.	MG445517.1
Hymenoptera	Chalcidoidea	Eulophidae	Eulophidae	sp.	MG446331.1
Hymenoptera	Chalcidoidea	Eulophidae	Eulophidae	sp.	MG446540.1
Hymenoptera	Chalcidoidea	Eulophidae	Eulophidae	sp.	MG446819.1
Hymenoptera	Chalcidoidea	Eulophidae	Eulophidae	sp.	MG447148.1
Hymenoptera	Chalcidoidea	Eulophidae	Eulophidae	sp.	MG447688.1
Hymenoptera	Chalcidoidea	Eulophidae	Eulophidae	sp.	MG448048.1
Hymenoptera	Chalcidoidea	Eulophidae	Eulophidae	sp.	MG480456.1
Hymenoptera	Chalcidoidea	Eulophidae	Eulophidae	sp.	MG482444.1
Hymenoptera	Chalcidoidea	Eulophidae	Eulophidae	sp.	MG482507.1
Hymenoptera	Chalcidoidea	Eulophidae	Eulophidae	sp.	MG483084.1
Hymenoptera	Chalcidoidea	Eulophidae	Eulophidae	sp.	MG483155.1
Hymenoptera	Chalcidoidea	Eulophidae	Eulophidae	sp.	MG484705.1
Hymenoptera	Chalcidoidea	Eulophidae	Eulophidae	sp.	MG485032.1
Hymenoptera	Chalcidoidea	Eulophidae	Eulophidae	sp.	MG485659.1
Hymenoptera	Chalcidoidea	Eulophidae	Eulophidae	sp.	MG487269.1
Hymenoptera	Chalcidoidea	Eulophidae	Eulophidae	sp.	MH095020.1
Hymenoptera	Chalcidoidea	Eulophidae	Eulophidae	sp.	MH095494.1
Hymenoptera	Chalcidoidea	Eulophidae	Eulophidae	sp.	MH095513.1
Hymenoptera	Chalcidoidea	Eulophidae	Eulophidae	sp.	MH926392.1
Hymenoptera	Chalcidoidea	Eulophidae	Eulophidae	sp.	MH926403.1
Hymenoptera	Chalcidoidea	Eulophidae	Eulophidae	sp.	MH926423.1
Hymenoptera	Chalcidoidea	Eulophidae	Eulophidae	sp.	MH926522.1
Hymenoptera	Chalcidoidea	Eulophidae	Eulophidae	sp.	MH926539.1
Hymenoptera	Chalcidoidea	Eulophidae	Eulophidae	sp.	MH926556.1
Hymenoptera	Chalcidoidea	Eulophidae	Eulophidae	sp.	MH926612.1
Hymenoptera	Chalcidoidea	Eulophidae	Eulophidae	sp.	MH926637.1
Hymenoptera	Chalcidoidea	Eulophidae	Eulophidae	sp.	MH926646.1
Hymenoptera	Chalcidoidea	Eulophidae	Eulophidae	sp.	MH926715.1
Hymenoptera	Chalcidoidea	Eulophidae	Eulophidae	sp.	MH926731.1
Hymenoptera	Chalcidoidea	Eulophidae	Eulophidae	sp.	MH926748.1
Hymenoptera	Chalcidoidea	Eulophidae	Eulophidae	sp.	MH926781.1
Hymenoptera	Chalcidoidea	Eulophidae	Eulophidae	sp.	MH926797.1
Hymenoptera	Chalcidoidea	Eulophidae	Eulophidae	sp.	MH926908.1

Hymenoptera	Chalcidoidea	Eulophidae	Eulophidae	sp.	MH926916.1
Hymenoptera	Chalcidoidea	Eulophidae	Eulophidae	sp.	MH926953.1
Hymenoptera	Chalcidoidea	Eulophidae	Eulophidae	sp.	MH927042.1
Hymenoptera	Chalcidoidea	Eulophidae	Eulophidae	sp.	MH927057.1
Hymenoptera	Chalcidoidea	Eulophidae	Eulophidae	sp.	MH927086.1
Hymenoptera	Chalcidoidea	Eulophidae	Eulophidae	sp.	MH927089.1
Hymenoptera	Chalcidoidea	Eulophidae	Eulophidae	sp.	MH927100.1
Hymenoptera	Chalcidoidea	Eulophidae	Eulophidae	sp.	MH927175.1
Hymenoptera	Chalcidoidea	Eulophidae	Eulophidae	sp.	MH927192.1
Hymenoptera	Chalcidoidea	Eulophidae	Eulophidae	sp.	MH927270.1
Hymenoptera	Chalcidoidea	Eulophidae	Eulophidae	sp.	MH927290.1
Hymenoptera	Chalcidoidea	Eulophidae	Eulophidae	sp.	MH927326.1
Hymenoptera	Chalcidoidea	Eulophidae	Eulophidae	sp.	MH927403.1
Hymenoptera	Chalcidoidea	Eulophidae	Eulophidae	sp.	MH927413.1
Hymenoptera	Chalcidoidea	Eulophidae	Eulophidae	sp.	MH927420.1
Hymenoptera	Chalcidoidea	Eulophidae	Eulophidae	sp.	MH927450.1
Hymenoptera	Chalcidoidea	Eulophidae	Eulophidae	sp.	MH927483.1
Hymenoptera	Chalcidoidea	Eulophidae	Eulophidae	sp.	MH927551.1
Hymenoptera	Chalcidoidea	Eulophidae	Eulophidae	sp.	MH927556.1
Hymenoptera	Chalcidoidea	Eulophidae	Eulophidae	sp.	MH927602.1
Hymenoptera	Chalcidoidea	Eulophidae	Eulophidae	sp.	MH927606.1
Hymenoptera	Chalcidoidea	Eulophidae	Eulophidae	sp.	MH927706.1
Hymenoptera	Chalcidoidea	Eulophidae	Eulophidae	sp.	MH927754.1
Hymenoptera	Chalcidoidea	Eulophidae	Eulophidae	sp.	MH927786.1
Hymenoptera	Chalcidoidea	Eulophidae	Eulophidae	sp.	MH927823.1
Hymenoptera	Chalcidoidea	Eulophidae	Eulophidae	sp.	MH927868.1
Hymenoptera	Chalcidoidea	Eulophidae	Eulophidae	sp.	MH927872.1
Hymenoptera	Chalcidoidea	Eulophidae	Eulophidae	sp.	MH928000.1
Hymenoptera	Chalcidoidea	Eulophidae	Eulophidae	sp.	MH928013.1
Hymenoptera	Chalcidoidea	Eulophidae	Eulophidae	sp.	MH928106.1
Hymenoptera	Chalcidoidea	Eulophidae	Eulophidae	sp.	MH928128.1
Hymenoptera	Chalcidoidea	Eulophidae	Eulophidae	sp.	MH928189.1
Hymenoptera	Chalcidoidea	Eulophidae	Eulophidae	sp.	MH928219.1
Hymenoptera	Chalcidoidea	Eulophidae	Eulophidae	sp.	MH928405.1
Hymenoptera	Chalcidoidea	Eulophidae	Eulophidae	sp.	MH928519.1
Hymenoptera	Chalcidoidea	Eulophidae	Eulophidae	sp.	MH928560.1
Hymenoptera	Chalcidoidea	Eulophidae	Eulophidae	sp.	MH928656.1
Hymenoptera	Chalcidoidea	Eulophidae	Eulophidae	sp.	MH928691.1
Hymenoptera	Chalcidoidea	Eulophidae	Eulophidae	sp.	MH928746.1
Hymenoptera	Chalcidoidea	Eulophidae	Eulophidae	sp.	MH928747.1
Hymenoptera	Chalcidoidea	Eulophidae	Eulophidae	sp.	MH928775.1
Hymenoptera	Chalcidoidea	Eulophidae	Eulophidae	sp.	MH928848.1
Hymenoptera	Chalcidoidea	Eulophidae	Eulophidae	sp.	MH928878.1
Hymenoptera	Chalcidoidea	Eulophidae	Eulophidae	sp.	MH928892.1
Hymenoptera	Chalcidoidea	Eulophidae	Eulophidae	sp.	MH929029.1
Hymenoptera	Chalcidoidea	Eulophidae	Eulophidae	sp.	MH929060.1
Hymenoptera	Chalcidoidea	Eulophidae	Eulophidae	sp.	MN665473.1
Hymenoptera	Chalcidoidea	Eulophidae	Eulophidae	sp.	MN665494.1
Hymenoptera	Chalcidoidea	Eulophidae	Eulophidae	sp.	MN665774.1
Hymenoptera	Chalcidoidea	Eulophidae	Eulophidae	sp.	MN665807.1
Hymenoptera	Chalcidoidea	Eulophidae	Eulophidae	sp.	MN665909.1
Hymenoptera	Chalcidoidea	Eulophidae	Eulophidae	sp.	MN665943.1
Hymenoptera	Chalcidoidea	Eulophidae	Eulophidae	sp.	MN666216.1
Hymenoptera	Chalcidoidea	Eulophidae	Eulophidae	sp.	MN666276.1

Hymenoptera	Chalcidoidea	Eulophidae	Eulophidae	sp.	MN666427.1
Hymenoptera	Chalcidoidea	Eulophidae	Eulophidae	sp.	MN666522.1
Hymenoptera	Chalcidoidea	Eulophidae	Eulophidae	sp.	MN666758.1
Hymenoptera	Chalcidoidea	Eulophidae	Eulophidae	sp.	MN666805.1
Hymenoptera	Chalcidoidea	Eulophidae	Eulophidae	sp.	MN666813.1
Hymenoptera	Chalcidoidea	Eulophidae	Eulophidae	sp.	MN667131.1
Hymenoptera	Chalcidoidea	Eulophidae	Eulophidae	sp.	MN667191.1
Hymenoptera	Chalcidoidea	Eulophidae	Eulophidae	sp.	MN667497.1
Hymenoptera	Chalcidoidea	Eulophidae	Eulophidae	sp.	MN667541.1
Hymenoptera	Chalcidoidea	Eulophidae	Eulophidae	sp.	MN667620.1
Hymenoptera	Chalcidoidea	Eulophidae	Eulophidae	sp.	MN667631.1
Hymenoptera	Chalcidoidea	Eulophidae	Eulophidae	sp.	MN667664.1
Hymenoptera	Chalcidoidea	Eulophidae	Eulophidae	sp.	MN667772.1
Hymenoptera	Chalcidoidea	Eulophidae	Eulophidae	sp.	MN667773.1
Hymenoptera	Chalcidoidea	Eulophidae	Eulophidae	sp.	MN667850.1
Hymenoptera	Chalcidoidea	Eulophidae	Eulophidae	sp.	MN668046.1
Hymenoptera	Chalcidoidea	Eulophidae	Eulophidae	sp.	MN668274.1
Hymenoptera	Chalcidoidea	Eulophidae	Eulophidae	sp.	MN668319.1
Hymenoptera	Chalcidoidea	Eulophidae	Eulophidae	sp.	MN668338.1
Hymenoptera	Chalcidoidea	Eulophidae	Eulophidae	sp.	MN668373.1
Hymenoptera	Chalcidoidea	Eulophidae	Eulophidae	sp.	MN668430.1
Hymenoptera	Chalcidoidea	Eulophidae	Eulophidae	sp.	MN668576.1
Hymenoptera	Chalcidoidea	Eulophidae	Eulophidae	sp.	MN668583.1
Hymenoptera	Chalcidoidea	Eulophidae	Eulophidae	sp.	MN668714.1
Hymenoptera	Chalcidoidea	Eulophidae	Eulophidae	sp.	MN668773.1
Hymenoptera	Chalcidoidea	Eulophidae	Eulophidae	sp.	MN668964.1
Hymenoptera	Chalcidoidea	Eulophidae	Eulophidae	sp.	MN669005.1
Hymenoptera	Chalcidoidea	Eulophidae	Eulophidae	sp.	MN669035.1
Hymenoptera	Chalcidoidea	Eulophidae	Eulophidae	sp.	MN669143.1
Hymenoptera	Chalcidoidea	Eulophidae	Eulophidae	sp.	MN669375.1
Hymenoptera	Chalcidoidea	Eulophidae	Eulophidae	sp.	MN669444.1
Hymenoptera	Chalcidoidea	Eulophidae	Eulophidae	sp.	MN669481.1
Hymenoptera	Chalcidoidea	Eulophidae	Eulophidae	sp.	MN669496.1
Hymenoptera	Chalcidoidea	Eulophidae	Eulophidae	sp.	MN669511.1
Hymenoptera	Chalcidoidea	Eulophidae	Eulophidae	sp.	MN669674.1
Hymenoptera	Chalcidoidea	Eulophidae	Eulophidae	sp.	MN669686.1
Hymenoptera	Chalcidoidea	Eulophidae	Eulophidae	sp.	MN669874.1
Hymenoptera	Chalcidoidea	Eulophidae	Eulophidae	sp.	MN669925.1
Hymenoptera	Chalcidoidea	Eulophidae	Eulophidae	sp.	MN669977.1
Hymenoptera	Chalcidoidea	Eulophidae	Eulophidae	sp.	MN669998.1
Hymenoptera	Chalcidoidea	Eulophidae	Eulophidae	sp.	MN670036.1
Hymenoptera	Chalcidoidea	Eulophidae	Eulophidae	sp.	MN670165.1
Hymenoptera	Chalcidoidea	Eulophidae	Eulophidae	sp.	MN670357.1
Hymenoptera	Chalcidoidea	Eulophidae	Eulophidae	sp.	MN670391.1
Hymenoptera	Chalcidoidea	Eulophidae	Eulophidae	sp.	MN670410.1
Hymenoptera	Chalcidoidea	Eulophidae	Eulophidae	sp.	MN670582.1
Hymenoptera	Chalcidoidea	Eulophidae	Eulophidae	sp.	MN670606.1
Hymenoptera	Chalcidoidea	Eulophidae	Eulophidae	sp.	MN670709.1
Hymenoptera	Chalcidoidea	Eulophidae	Eulophidae	sp.	MN670712.1
Hymenoptera	Chalcidoidea	Eulophidae	Eulophidae	sp.	MN670817.1
Hymenoptera	Chalcidoidea	Eulophidae	Eulophidae	sp.	MN671045.1
Hymenoptera	Chalcidoidea	Eulophidae	Eulophidae	sp.	MN671081.1
Hymenoptera	Chalcidoidea	Eulophidae	Eulophidae	sp.	MN671093.1
Hymenoptera	Chalcidoidea	Eulophidae	Eulophidae	sp.	MN671138.1

Hymenoptera	Chalcidoidea	Eulophidae	Eulophidae	sp.	MN671180.1
Hymenoptera	Chalcidoidea	Eulophidae	Eulophidae	sp.	MN671281.1
Hymenoptera	Chalcidoidea	Eulophidae	Eulophidae	sp.	MN671386.1
Hymenoptera	Chalcidoidea	Eulophidae	Eulophidae	sp.	MN671430.1
Hymenoptera	Chalcidoidea	Eulophidae	Eulophidae	sp.	MN671573.1
Hymenoptera	Chalcidoidea	Eulophidae	Eulophidae	sp.	MN671630.1
Hymenoptera	Chalcidoidea	Eulophidae	Eulophidae	sp.	MN671744.1
Hymenoptera	Chalcidoidea	Eulophidae	Eulophidae	sp.	MN671757.1
Hymenoptera	Chalcidoidea	Eulophidae	Eulophidae	sp.	MN671950.1
Hymenoptera	Chalcidoidea	Eulophidae	Eulophidae	sp.	MN671952.1
Hymenoptera	Chalcidoidea	Eulophidae	Eulophidae	sp.	MN671993.1
Hymenoptera	Chalcidoidea	Eulophidae	Eulophidae	sp.	MN672013.1
Hymenoptera	Chalcidoidea	Eulophidae	Eulophidae	sp.	MN672253.1
Hymenoptera	Chalcidoidea	Eulophidae	Eulophidae	sp.	MN672255.1
Hymenoptera	Chalcidoidea	Eulophidae	Eulophidae	sp.	MN672258.1
Hymenoptera	Chalcidoidea	Eulophidae	Eulophidae	sp.	MN672587.1
Hymenoptera	Chalcidoidea	Eulophidae	Eulophidae	sp.	MN673188.1
Hymenoptera	Chalcidoidea	Eulophidae	Eulophidae	sp.	MN673207.1
Hymenoptera	Chalcidoidea	Eulophidae	Eulophidae	sp.	MN673269.1
Hymenoptera	Chalcidoidea	Eulophidae	Eulophidae	sp.	MN673337.1
Hymenoptera	Chalcidoidea	Eulophidae	Eulophidae	sp.	MN673448.1
Hymenoptera	Chalcidoidea	Eulophidae	Eulophidae	sp.	MN674212.1
Hymenoptera	Chalcidoidea	Eulophidae	Eulophidae	sp.	MN674437.1
Hymenoptera	Chalcidoidea	Eulophidae	Eulophidae	sp.	MN674486.1
Hymenoptera	Chalcidoidea	Eulophidae	Eulophidae	sp.	MN674585.1
Hymenoptera	Chalcidoidea	Eulophidae	Eulophidae	sp.	MN674614.1
Hymenoptera	Chalcidoidea	Eulophidae	Eulophidae	sp.	MN674947.1
Hymenoptera	Chalcidoidea	Eulophidae	Eulophidae	sp.	MN674952.1
Hymenoptera	Chalcidoidea	Eulophidae	Eulophidae	sp.	MN674990.1
Hymenoptera	Chalcidoidea	Eulophidae	Eulophidae	sp.	MN675010.1
Hymenoptera	Chalcidoidea	Eulophidae	Eulophidae	sp.	MN675037.1
Hymenoptera	Chalcidoidea	Eulophidae	Eulophidae	sp.	MN675038.1
Hymenoptera	Chalcidoidea	Eulophidae	Eulophidae	sp.	MN675087.1
Hymenoptera	Chalcidoidea	Eulophidae	Eulophidae	sp.	MN675169.1
Hymenoptera	Chalcidoidea	Eulophidae	Eulophidae	sp.	MN675201.1
Hymenoptera	Chalcidoidea	Eulophidae	Eulophidae	sp.	MN675655.1
Hymenoptera	Chalcidoidea	Eulophidae	Eulophidae	sp.	MN675694.1
Hymenoptera	Chalcidoidea	Eulophidae	Eulophidae	sp.	MN675784.1
Hymenoptera	Chalcidoidea	Eulophidae	Eulophidae	sp.	MN675921.1
Hymenoptera	Chalcidoidea	Eulophidae	Eulophidae	sp.	MN676165.1
Hymenoptera	Chalcidoidea	Eulophidae	Eulophidae	sp.	MN676213.1
Hymenoptera	Chalcidoidea	Eulophidae	Eulophidae	sp.	MN676291.1
Hymenoptera	Chalcidoidea	Eulophidae	Eulophidae	sp.	MN676355.1
Hymenoptera	Chalcidoidea	Eulophidae	Eulophidae	sp.	MN676435.1
Hymenoptera	Chalcidoidea	Eulophidae	Eulophidae	sp.	MN676605.1
Hymenoptera	Chalcidoidea	Eulophidae	Eulophidae	sp.	MN676643.1
Hymenoptera	Chalcidoidea	Eulophidae	Eulophidae	sp.	MN676669.1
Hymenoptera	Chalcidoidea	Eulophidae	Eulophidae	sp.	MN676702.1
Hymenoptera	Chalcidoidea	Eulophidae	Eulophidae	sp.	MN676889.1
Hymenoptera	Chalcidoidea	Eulophidae	Eulophidae	sp.	MN676939.1
Hymenoptera	Chalcidoidea	Eulophidae	Eulophidae	sp.	MN677064.1
Hymenoptera	Chalcidoidea	Eulophidae	Eulophidae	sp.	MN677097.1
Hymenoptera	Chalcidoidea	Eulophidae	Eulophidae	sp.	MN677106.1
Hymenoptera	Chalcidoidea	Eulophidae	Eulophidae	sp.	MN677170.1

Hymenoptera	Chalcidoidea	Eulophidae	Eulophidae	sp.	MN677175.1
Hymenoptera	Chalcidoidea	Eulophidae	Eulophidae	sp.	MN677285.1
Hymenoptera	Chalcidoidea	Eulophidae	Eulophidae	sp.	MN677286.1
Hymenoptera	Chalcidoidea	Eulophidae	Eulophidae	sp.	MN677558.1
Hymenoptera	Chalcidoidea	Eulophidae	Eulophidae	sp.	MN677584.1
Hymenoptera	Chalcidoidea	Eulophidae	Eulophidae	sp.	MN677646.1
Hymenoptera	Chalcidoidea	Eulophidae	Eulophidae	sp.	MN677659.1
Hymenoptera	Chalcidoidea	Eulophidae	Eulophidae	sp.	MN677740.1
Hymenoptera	Chalcidoidea	Eulophidae	Eulophidae	sp.	MN677771.1
Hymenoptera	Chalcidoidea	Eulophidae	Eulophidae	sp.	MN677776.1
Hymenoptera	Chalcidoidea	Eulophidae	Eulophidae	sp.	MN677815.1
Hymenoptera	Chalcidoidea	Eulophidae	Eulophidae	sp.	MN678061.1
Hymenoptera	Chalcidoidea	Eulophidae	Eulophidae	sp.	MN678069.1
Hymenoptera	Chalcidoidea	Eulophidae	Eulophidae	sp.	MN678274.1
Hymenoptera	Chalcidoidea	Eulophidae	Eulophidae	sp.	MN678284.1
Hymenoptera	Chalcidoidea	Eulophidae	Eulophidae	sp.	MN678302.1
Hymenoptera	Chalcidoidea	Eulophidae	Eulophidae	sp.	MN678319.1
Hymenoptera	Chalcidoidea	Eulophidae	Eulophidae	sp.	MN678408.1
Hymenoptera	Chalcidoidea	Eulophidae	Eulophidae	sp.	MN678543.1
Hymenoptera	Chalcidoidea	Eulophidae	Eulophidae	sp.	MN678583.1
Hymenoptera	Chalcidoidea	Eulophidae	Eulophidae	sp.	MN678605.1
Hymenoptera	Chalcidoidea	Eulophidae	Eulophidae	sp.	MN678614.1
Hymenoptera	Chalcidoidea	Eulophidae	Eulophidae	sp.	MN678698.1
Hymenoptera	Chalcidoidea	Eulophidae	Eulophidae	sp.	MN678800.1
Hymenoptera	Chalcidoidea	Eulophidae	Eulophidae	sp.	MN678885.1
Hymenoptera	Chalcidoidea	Eulophidae	Eulophidae	sp.	MN678925.1
Hymenoptera	Chalcidoidea	Eulophidae	Eulophidae	sp.	MN679098.1
Hymenoptera	Chalcidoidea	Eulophidae	Eulophidae	sp.	MN679141.1
Hymenoptera	Chalcidoidea	Eulophidae	Eulophidae	sp.	MN679147.1
Hymenoptera	Chalcidoidea	Eulophidae	Eulophidae	sp.	MN679390.1
Hymenoptera	Chalcidoidea	Eulophidae	Eulophidae	sp.	MN679536.1
Hymenoptera	Chalcidoidea	Eulophidae	Eulophidae	sp.	MN679646.1
Hymenoptera	Chalcidoidea	Eulophidae	Eulophidae	sp.	MN679688.1
Hymenoptera	Chalcidoidea	Eulophidae	Eulophidae	sp.	MN679745.1
Hymenoptera	Chalcidoidea	Eulophidae	Eulophidae	sp.	MN679851.1
Hymenoptera	Chalcidoidea	Eulophidae	Eulophidae	sp.	MN679916.1
Hymenoptera	Chalcidoidea	Eulophidae	Eulophidae	sp.	MN679947.1
Hymenoptera	Chalcidoidea	Eulophidae	Eulophidae	sp.	MN679992.1
Hymenoptera	Chalcidoidea	Eulophidae	Eulophidae	sp.	MN680046.1
Hymenoptera	Chalcidoidea	Eulophidae	Eulophidae	sp.	MN680062.1
Hymenoptera	Chalcidoidea	Eulophidae	Eulophidae	sp.	MN680160.1
Hymenoptera	Chalcidoidea	Eulophidae	Eulophidae	sp.	MN680394.1
Hymenoptera	Chalcidoidea	Eulophidae	Eulophidae	sp.	MN680430.1
Hymenoptera	Chalcidoidea	Eulophidae	Eulophidae	sp.	MN680515.1
Hymenoptera	Chalcidoidea	Eulophidae	Eulophidae	sp.	MN681027.1
Hymenoptera	Chalcidoidea	Eulophidae	Eulophidae	sp.	MN681194.1
Hymenoptera	Chalcidoidea	Eulophidae	Eulophidae	sp.	MN681376.1
Hymenoptera	Chalcidoidea	Eulophidae	Eulophidae	sp.	MN681508.1
Hymenoptera	Chalcidoidea	Eulophidae	Eulophidae	sp.	MN681650.1
Hymenoptera	Chalcidoidea	Eulophidae	Eulophidae	sp.	MN681892.1
Hymenoptera	Chalcidoidea	Eulophidae	Eulophidae	sp.	MN681904.1
Hymenoptera	Chalcidoidea	Eulophidae	Eulophidae	sp.	MN682166.1
Hymenoptera	Chalcidoidea	Eulophidae	Eulophidae	sp.	MN682533.1
Hymenoptera	Chalcidoidea	Eulophidae	Eulophidae	sp.	MN682665.1

Hymenoptera	Chalcidoidea	Eulophidae	Eulophidae	sp.	MN682722.1
Hymenoptera	Chalcidoidea	Eulophidae	Eulophidae	sp.	MN682977.1
Hymenoptera	Chalcidoidea	Eulophidae	Eulophidae	sp.	MN683039.1
Hymenoptera	Chalcidoidea	Eulophidae	Eulophidae	sp.	MN683089.1
Hymenoptera	Chalcidoidea	Eulophidae	Eulophidae	sp.	MN683145.1
Hymenoptera	Chalcidoidea	Eulophidae	Eulophidae	sp.	MN683346.1
Hymenoptera	Chalcidoidea	Eulophidae	Eulophidae	sp.	MN683373.1
Hymenoptera	Chalcidoidea	Eulophidae	Eulophidae	sp.	MW784318.1
Hymenoptera	Chalcidoidea	Eulophidae	Eulophidae	sp.	MZ629303.1
Hymenoptera	Chalcidoidea	Eulophidae	Eulophidae	sp.	MZ629397.1
Hymenoptera	Chalcidoidea	Eulophidae	Eulophidae	sp.	MZ629534.1
Hymenoptera	Chalcidoidea	Eulophidae	Eulophidae	sp.	MZ629569.1
Hymenoptera	Chalcidoidea	Eulophidae	Eulophidae	sp.	MZ629637.1
Hymenoptera	Chalcidoidea	Eulophidae	Eulophidae	sp.	MZ630024.1
Hymenoptera	Chalcidoidea	Eulophidae	Eulophidae	sp.	MZ630269.1
Hymenoptera	Chalcidoidea	Eulophidae	Eulophidae	sp.	MZ630308.1
Hymenoptera	Chalcidoidea	Eulophidae	Eulophidae	sp.	MZ630779.1
Hymenoptera	Chalcidoidea	Eulophidae	Eulophidae	sp.	MZ631100.1
Hymenoptera	Chalcidoidea	Eulophidae	Eulophidae	sp.	MZ631299.1
Hymenoptera	Chalcidoidea	Eulophidae	Eulophidae	sp.	MZ631471.1
Hymenoptera	Chalcidoidea	Eulophidae	Eulophidae	sp.	MZ631568.1
Hymenoptera	Chalcidoidea	Eulophidae	Eulophidae	sp.	MZ631810.1
Hymenoptera	Chalcidoidea	Eulophidae	Eulophidae	sp.	MZ631941.1
Hymenoptera	Chalcidoidea	Eulophidae	Eulophidae	sp.	MZ632069.1
Hymenoptera	Chalcidoidea	Eulophidae	Eulophidae	sp.	MZ632404.1
Hymenoptera	Chalcidoidea	Eulophidae	Eulophidae	sp.	MZ632500.1
Hymenoptera	Chalcidoidea	Eulophidae	Eulophidae	sp.	MZ632891.1
Hymenoptera	Chalcidoidea	Eulophidae	Eulophidae	sp.	MZ632981.1
Hymenoptera	Chalcidoidea	Eulophidae	Eulophidae	sp.	MZ633139.1
Hymenoptera	Chalcidoidea	Eulophidae	Eulophidae	sp.	MZ633175.1
Hymenoptera	Chalcidoidea	Eulophidae	Eulophidae	sp.	MZ633263.1
Hymenoptera	Chalcidoidea	Eulophidae	Eulophidae	sp.	MZ656810.1
Hymenoptera	Chalcidoidea	Eulophidae	Eulophidae	sp.	MZ657185.1
Hymenoptera	Chalcidoidea	Eulophidae	Eulophidae	sp.	MZ657267.1
Hymenoptera	Chalcidoidea	Eulophidae	Eulophidae	sp.	MZ658182.1
Hymenoptera	Chalcidoidea	Eulophidae	Eulophidae	sp.	MZ658188.1
Hymenoptera	Chalcidoidea	Eulophidae	Eulophidae	sp.	MZ658427.1
Hymenoptera	Chalcidoidea	Eulophidae	Eulophidae	sp.	OL694540.1
Hymenoptera	Chalcidoidea	Eulophidae	Eulophus	sp.	HM365051.1
Hymenoptera	Chalcidoidea	Eulophidae	Euplectrus	alvarowillei	KP150381.1
Hymenoptera	Chalcidoidea	Eulophidae	Euplectrus	alvarowillei	KP150394.1
Hymenoptera	Chalcidoidea	Eulophidae	Euplectrus	bicolor	KY421532.1
Hymenoptera	Chalcidoidea	Eulophidae	Euplectrus	bicolor	KY421539.1
Hymenoptera	Chalcidoidea	Eulophidae	Euplectrus	bicolor	MH587800.1
Hymenoptera	Chalcidoidea	Eulophidae	Euplectrus	bicolor	MH587806.1
Hymenoptera	Chalcidoidea	Eulophidae	Euplectrus	bicolor	MH587817.1
Hymenoptera	Chalcidoidea	Eulophidae	Euplectrus	bicolor	MH587818.1
Hymenoptera	Chalcidoidea	Eulophidae	Euplectrus	bicolor	MH587821.1
Hymenoptera	Chalcidoidea	Eulophidae	Euplectrus	bicolor	MH587828.1
Hymenoptera	Chalcidoidea	Eulophidae	Euplectrus	bicolor	MH587829.1
Hymenoptera	Chalcidoidea	Eulophidae	Euplectrus	bicolor	MH587831.1
Hymenoptera	Chalcidoidea	Eulophidae	Euplectrus	bicolor	MH587834.1
Hymenoptera	Chalcidoidea	Eulophidae	Euplectrus	bicolor	MH587835.1
Hymenoptera	Chalcidoidea	Eulophidae	Euplectrus	bicolor	MH587847.1

Hymenoptera	Chalcidoidea	Eulophidae	Euplectrus	bicolor	MH587850.1
Hymenoptera	Chalcidoidea	Eulophidae	Euplectrus	bicolor	MH587851.1
Hymenoptera	Chalcidoidea	Eulophidae	Euplectrus	bicolor	MH587863.1
Hymenoptera	Chalcidoidea	Eulophidae	Euplectrus	bicolor	MH587873.1
Hymenoptera	Chalcidoidea	Eulophidae	Euplectrus	bicolor	MH587882.1
Hymenoptera	Chalcidoidea	Eulophidae	Euplectrus	bicolor	MH587898.1
Hymenoptera	Chalcidoidea	Eulophidae	Euplectrus	bicolor	MH587904.1
Hymenoptera	Chalcidoidea	Eulophidae	Euplectrus	bicolor	MH587909.1
Hymenoptera	Chalcidoidea	Eulophidae	Euplectrus	bicolor	MH587911.1
Hymenoptera	Chalcidoidea	Eulophidae	Euplectrus	bicolor	MH587912.1
Hymenoptera	Chalcidoidea	Eulophidae	Euplectrus	bicolor	MH587914.1
Hymenoptera	Chalcidoidea	Eulophidae	Euplectrus	bicolor	MH587919.1
Hymenoptera	Chalcidoidea	Eulophidae	Euplectrus	bicolor	MH587930.1
Hymenoptera	Chalcidoidea	Eulophidae	Euplectrus	bicolor	MH587931.1
Hymenoptera	Chalcidoidea	Eulophidae	Euplectrus	bicolor	MH587942.1
Hymenoptera	Chalcidoidea	Eulophidae	Euplectrus	bicolor	MH587944.1
Hymenoptera	Chalcidoidea	Eulophidae	Euplectrus	bicolor	MH587950.1
Hymenoptera	Chalcidoidea	Eulophidae	Euplectrus	bicolor	MH587951.1
Hymenoptera	Chalcidoidea	Eulophidae	Euplectrus	bicolor	MH587959.1
Hymenoptera	Chalcidoidea	Eulophidae	Euplectrus	bicolor	MH587965.1
Hymenoptera	Chalcidoidea	Eulophidae	Euplectrus	bicolor	MH587970.1
Hymenoptera	Chalcidoidea	Eulophidae	Euplectrus	bicolor	MH587978.1
Hymenoptera	Chalcidoidea	Eulophidae	Euplectrus	bicolor	MH587981.1
Hymenoptera	Chalcidoidea	Eulophidae	Euplectrus	bicolor	MH587984.1
Hymenoptera	Chalcidoidea	Eulophidae	Euplectrus	bicolor	MH587985.1
Hymenoptera	Chalcidoidea	Eulophidae	Euplectrus	bicolor	MH587988.1
Hymenoptera	Chalcidoidea	Eulophidae	Euplectrus	bicolor	MH587989.1
Hymenoptera	Chalcidoidea	Eulophidae	Euplectrus	bicolor	MH587991.1
Hymenoptera	Chalcidoidea	Eulophidae	Euplectrus	bicolor	MH587999.1
Hymenoptera	Chalcidoidea	Eulophidae	Euplectrus	bicolor	MH588003.1
Hymenoptera	Chalcidoidea	Eulophidae	Euplectrus	bicolor	MH588006.1
Hymenoptera	Chalcidoidea	Eulophidae	Euplectrus	bicolor	MH588008.1
Hymenoptera	Chalcidoidea	Eulophidae	Euplectrus	bicolor	MH588011.1
Hymenoptera	Chalcidoidea	Eulophidae	Euplectrus	bicolor	MH588032.1
Hymenoptera	Chalcidoidea	Eulophidae	Euplectrus	bicolor	MH588055.1
Hymenoptera	Chalcidoidea	Eulophidae	Euplectrus	carlrettenmeyeri	KP150411.1
Hymenoptera	Chalcidoidea	Eulophidae	Euplectrus	charlesmicheneri	HQ548193.1
Hymenoptera	Chalcidoidea	Eulophidae	Euplectrus	charlesmicheneri	HQ548194.1
Hymenoptera	Chalcidoidea	Eulophidae	Euplectrus	corriemoreauae	KP150369.1
Hymenoptera	Chalcidoidea	Eulophidae	Euplectrus	daveroubiki	HQ548197.1
Hymenoptera	Chalcidoidea	Eulophidae	Euplectrus	daveroubiki	KP150336.1
Hymenoptera	Chalcidoidea	Eulophidae	Euplectrus	dianariasae	KP150389.1
Hymenoptera	Chalcidoidea	Eulophidae	Euplectrus	eowilsoni	KP150309.1
Hymenoptera	Chalcidoidea	Eulophidae	Euplectrus	eowilsoni	KP150311.1
Hymenoptera	Chalcidoidea	Eulophidae	Euplectrus	eowilsoni	KP150318.1
Hymenoptera	Chalcidoidea	Eulophidae	Euplectrus	eowilsoni	KP150359.1
Hymenoptera	Chalcidoidea	Eulophidae	Euplectrus	eowilsoni	KP150366.1
Hymenoptera	Chalcidoidea	Eulophidae	Euplectrus	eowilsoni	KP150399.1
Hymenoptera	Chalcidoidea	Eulophidae	Euplectrus	eowilsoni	KP150405.1
Hymenoptera	Chalcidoidea	Eulophidae	Euplectrus	eowilsoni	KP150406.1
Hymenoptera	Chalcidoidea	Eulophidae	Euplectrus	eowilsoni	KP150412.1
Hymenoptera	Chalcidoidea	Eulophidae	Euplectrus	eowilsoni	KP150420.1
Hymenoptera	Chalcidoidea	Eulophidae	Euplectrus	euplexiae	MK000733.1
Hymenoptera	Chalcidoidea	Eulophidae	Euplectrus	flavipes	MH587808.1

Hymenoptera	Chalcidoidea	Eulophidae	Euplectrus	flavipes	MH587809.1
Hymenoptera	Chalcidoidea	Eulophidae	Euplectrus	flavipes	MH587814.1
Hymenoptera	Chalcidoidea	Eulophidae	Euplectrus	flavipes	MH587816.1
Hymenoptera	Chalcidoidea	Eulophidae	Euplectrus	flavipes	MH587838.1
Hymenoptera	Chalcidoidea	Eulophidae	Euplectrus	flavipes	MH587841.1
Hymenoptera	Chalcidoidea	Eulophidae	Euplectrus	flavipes	MH587843.1
Hymenoptera	Chalcidoidea	Eulophidae	Euplectrus	flavipes	MH587859.1
Hymenoptera	Chalcidoidea	Eulophidae	Euplectrus	flavipes	MH587879.1
Hymenoptera	Chalcidoidea	Eulophidae	Euplectrus	flavipes	MH587881.1
Hymenoptera	Chalcidoidea	Eulophidae	Euplectrus	flavipes	MH587921.1
Hymenoptera	Chalcidoidea	Eulophidae	Euplectrus	flavipes	MH587933.1
Hymenoptera	Chalcidoidea	Eulophidae	Euplectrus	flavipes	MH587936.1
Hymenoptera	Chalcidoidea	Eulophidae	Euplectrus	flavipes	MH587946.1
Hymenoptera	Chalcidoidea	Eulophidae	Euplectrus	flavipes	MH588015.1
Hymenoptera	Chalcidoidea	Eulophidae	Euplectrus	flavipes	MH588017.1
Hymenoptera	Chalcidoidea	Eulophidae	Euplectrus	flavipes	MH588019.1
Hymenoptera	Chalcidoidea	Eulophidae	Euplectrus	flavipes	MH588020.1
Hymenoptera	Chalcidoidea	Eulophidae	Euplectrus	flavipes	MH588021.1
Hymenoptera	Chalcidoidea	Eulophidae	Euplectrus	flavipes	MH588028.1
Hymenoptera	Chalcidoidea	Eulophidae	Euplectrus	flavipes	MH588031.1
Hymenoptera	Chalcidoidea	Eulophidae	Euplectrus	flavipes	MH588034.1
Hymenoptera	Chalcidoidea	Eulophidae	Euplectrus	floryae	KP150326.1
Hymenoptera	Chalcidoidea	Eulophidae	Euplectrus	floryae	KP150332.1
Hymenoptera	Chalcidoidea	Eulophidae	Euplectrus	floryae	KP150360.1
Hymenoptera	Chalcidoidea	Eulophidae	Euplectrus	gavinbroadi	KP150357.1
Hymenoptera	Chalcidoidea	Eulophidae	Euplectrus	hansoni	KP150367.1
Hymenoptera	Chalcidoidea	Eulophidae	Euplectrus	intactus	MH587801.1
Hymenoptera	Chalcidoidea	Eulophidae	Euplectrus	intactus	MH587802.1
Hymenoptera	Chalcidoidea	Eulophidae	Euplectrus	intactus	MH587803.1
Hymenoptera	Chalcidoidea	Eulophidae	Euplectrus	intactus	MH587811.1
Hymenoptera	Chalcidoidea	Eulophidae	Euplectrus	intactus	MH587812.1
Hymenoptera	Chalcidoidea	Eulophidae	Euplectrus	intactus	MH587837.1
Hymenoptera	Chalcidoidea	Eulophidae	Euplectrus	intactus	MH587848.1
Hymenoptera	Chalcidoidea	Eulophidae	Euplectrus	intactus	MH587855.1
Hymenoptera	Chalcidoidea	Eulophidae	Euplectrus	intactus	MH587877.1
Hymenoptera	Chalcidoidea	Eulophidae	Euplectrus	intactus	MH587917.1
Hymenoptera	Chalcidoidea	Eulophidae	Euplectrus	intactus	MH587934.1
Hymenoptera	Chalcidoidea	Eulophidae	Euplectrus	intactus	MH587957.1
Hymenoptera	Chalcidoidea	Eulophidae	Euplectrus	intactus	MH587964.1
Hymenoptera	Chalcidoidea	Eulophidae	Euplectrus	intactus	MH588047.1
Hymenoptera	Chalcidoidea	Eulophidae	Euplectrus	jacklonginoi	KP150314.1
Hymenoptera	Chalcidoidea	Eulophidae	Euplectrus	jacklonginoi	KP150382.1
Hymenoptera	Chalcidoidea	Eulophidae	Euplectrus	johoyesi	KP150334.1
Hymenoptera	Chalcidoidea	Eulophidae	Euplectrus	lubomirmasneri	KP150417.1
Hymenoptera	Chalcidoidea	Eulophidae	Euplectrus	maculiventris	MH587815.1
Hymenoptera	Chalcidoidea	Eulophidae	Euplectrus	magdae	KP150408.1
Hymenoptera	Chalcidoidea	Eulophidae	Euplectrus	mikegatesi	HQ926654.1
Hymenoptera	Chalcidoidea	Eulophidae	Euplectrus	mikeschauffi	KP150337.1
Hymenoptera	Chalcidoidea	Eulophidae	Euplectrus	mikeschauffi	KP150387.1
Hymenoptera	Chalcidoidea	Eulophidae	Euplectrus	pammitchellae	KP150364.1
Hymenoptera	Chalcidoidea	Eulophidae	Euplectrus	paulheberti	KP150372.1
Hymenoptera	Chalcidoidea	Eulophidae	Euplectrus	phthorimaeae	MW583595.1
Hymenoptera	Chalcidoidea	Eulophidae	Euplectrus	ronaldzunigai	KP150401.1
Hymenoptera	Chalcidoidea	Eulophidae	Euplectrus	ronaldzunigai	KP150410.1

Hymenoptera	Chalcidoidea	Eulophidae	Euplectrus	scottshawi	HQ548196.1
Hymenoptera	Chalcidoidea	Eulophidae	Euplectrus	scottshawi	KP150348.1
Hymenoptera	Chalcidoidea	Eulophidae	Euplectrus	scottshawi	KP150403.1
Hymenoptera	Chalcidoidea	Eulophidae	Euplectrus	scottshawi	KP150409.1
Hymenoptera	Chalcidoidea	Eulophidae	Euplectrus	sondrawardae	KP150349.1
Hymenoptera	Chalcidoidea	Eulophidae	Euplectrus	sp.	HM365050.1
Hymenoptera	Chalcidoidea	Eulophidae	Euplectrus	sp.	HQ552306.1
Hymenoptera	Chalcidoidea	Eulophidae	Euplectrus	sp.	HQ552308.1
Hymenoptera	Chalcidoidea	Eulophidae	Euplectrus	sp.	JN292154.1
Hymenoptera	Chalcidoidea	Eulophidae	Euplectrus	sp.	JQ574788.1
Hymenoptera	Chalcidoidea	Eulophidae	Euplectrus	sp.	JQ575782.1
Hymenoptera	Chalcidoidea	Eulophidae	Euplectrus	sp.	JQ575783.1
Hymenoptera	Chalcidoidea	Eulophidae	Euplectrus	sp.	JQ575784.1
Hymenoptera	Chalcidoidea	Eulophidae	Euplectrus	sp.	JQ575786.1
Hymenoptera	Chalcidoidea	Eulophidae	Euplectrus	sp.	JQ575788.1
Hymenoptera	Chalcidoidea	Eulophidae	Euplectrus	sp.	JQ575804.1
Hymenoptera	Chalcidoidea	Eulophidae	Euplectrus	sp.	JQ575807.1
Hymenoptera	Chalcidoidea	Eulophidae	Euplectrus	sp.	JQ575808.1
Hymenoptera	Chalcidoidea	Eulophidae	Euplectrus	sp.	JQ575809.1
Hymenoptera	Chalcidoidea	Eulophidae	Euplectrus	sp.	JQ575811.1
Hymenoptera	Chalcidoidea	Eulophidae	Euplectrus	sp.	JQ575813.1
Hymenoptera	Chalcidoidea	Eulophidae	Euplectrus	sp.	JQ575814.1
Hymenoptera	Chalcidoidea	Eulophidae	Euplectrus	sp.	JQ575815.1
Hymenoptera	Chalcidoidea	Eulophidae	Euplectrus	sp.	JQ575816.1
Hymenoptera	Chalcidoidea	Eulophidae	Euplectrus	sp.	KP150358.1
Hymenoptera	Chalcidoidea	Eulophidae	Euplectrus	sp.	KP150361.1
Hymenoptera	Chalcidoidea	Eulophidae	Euplectrus	sp.	KP150378.1
Hymenoptera	Chalcidoidea	Eulophidae	Euplectrus	sp.	KR793510.1
Hymenoptera	Chalcidoidea	Eulophidae	Euplectrus	sp.	KR794675.1
Hymenoptera	Chalcidoidea	Eulophidae	Euplectrus	sp.	KR802409.1
Hymenoptera	Chalcidoidea	Eulophidae	Euplectrus	sp.	KR884732.1
Hymenoptera	Chalcidoidea	Eulophidae	Euplectrus	sp.	KR899126.1
Hymenoptera	Chalcidoidea	Eulophidae	Euplectrus	sp.	KY315732.1
Hymenoptera	Chalcidoidea	Eulophidae	Euplectrus	sp.	KY421523.1
Hymenoptera	Chalcidoidea	Eulophidae	Euplectrus	sp.	KY421525.1
Hymenoptera	Chalcidoidea	Eulophidae	Euplectrus	sp.	KY421530.1
Hymenoptera	Chalcidoidea	Eulophidae	Euplectrus	sp.	MF903162.1
Hymenoptera	Chalcidoidea	Eulophidae	Euplectrus	sp.	MF906096.1
Hymenoptera	Chalcidoidea	Eulophidae	Euplectrus	sp.	MG340810.1
Hymenoptera	Chalcidoidea	Eulophidae	Euplectrus	sp.	MG341954.1
Hymenoptera	Chalcidoidea	Eulophidae	Euplectrus	sp.	MH587797.1
Hymenoptera	Chalcidoidea	Eulophidae	Euplectrus	sp.	MH587798.1
Hymenoptera	Chalcidoidea	Eulophidae	Euplectrus	sp.	MH587799.1
Hymenoptera	Chalcidoidea	Eulophidae	Euplectrus	sp.	MH587804.1
Hymenoptera	Chalcidoidea	Eulophidae	Euplectrus	sp.	MH587805.1
Hymenoptera	Chalcidoidea	Eulophidae	Euplectrus	sp.	MH587807.1
Hymenoptera	Chalcidoidea	Eulophidae	Euplectrus	sp.	MH587813.1
Hymenoptera	Chalcidoidea	Eulophidae	Euplectrus	sp.	MH587826.1
Hymenoptera	Chalcidoidea	Eulophidae	Euplectrus	sp.	MH587827.1
Hymenoptera	Chalcidoidea	Eulophidae	Euplectrus	sp.	MH587830.1
Hymenoptera	Chalcidoidea	Eulophidae	Euplectrus	sp.	MH587832.1
Hymenoptera	Chalcidoidea	Eulophidae	Euplectrus	sp.	MH587836.1
Hymenoptera	Chalcidoidea	Eulophidae	Euplectrus	sp.	MH587840.1
Hymenoptera	Chalcidoidea	Eulophidae	Euplectrus	sp.	MH587846.1

Hymenoptera	Chalcidoidea	Eulophidae	Euplectrus	sp.	MH587852.1
Hymenoptera	Chalcidoidea	Eulophidae	Euplectrus	sp.	MH587854.1
Hymenoptera	Chalcidoidea	Eulophidae	Euplectrus	sp.	MH587856.1
Hymenoptera	Chalcidoidea	Eulophidae	Euplectrus	sp.	MH587857.1
Hymenoptera	Chalcidoidea	Eulophidae	Euplectrus	sp.	MH587858.1
Hymenoptera	Chalcidoidea	Eulophidae	Euplectrus	sp.	MH587862.1
Hymenoptera	Chalcidoidea	Eulophidae	Euplectrus	sp.	MH587865.1
Hymenoptera	Chalcidoidea	Eulophidae	Euplectrus	sp.	MH587884.1
Hymenoptera	Chalcidoidea	Eulophidae	Euplectrus	sp.	MH587889.1
Hymenoptera	Chalcidoidea	Eulophidae	Euplectrus	sp.	MH587896.1
Hymenoptera	Chalcidoidea	Eulophidae	Euplectrus	sp.	MH587905.1
Hymenoptera	Chalcidoidea	Eulophidae	Euplectrus	sp.	MH587906.1
Hymenoptera	Chalcidoidea	Eulophidae	Euplectrus	sp.	MH587943.1
Hymenoptera	Chalcidoidea	Eulophidae	Euplectrus	sp.	MH587953.1
Hymenoptera	Chalcidoidea	Eulophidae	Euplectrus	sp.	MH587955.1
Hymenoptera	Chalcidoidea	Eulophidae	Euplectrus	sp.	MH587995.1
Hymenoptera	Chalcidoidea	Eulophidae	Euplectrus	sp.	MH588004.1
Hymenoptera	Chalcidoidea	Eulophidae	Euplectrus	sp.	MH588029.1
Hymenoptera	Chalcidoidea	Eulophidae	Euplectrus	sp.	MH588041.1
Hymenoptera	Chalcidoidea	Eulophidae	Euplectrus	victoriapookae	KP150343.1
Hymenoptera	Chalcidoidea	Eulophidae	Euplectrus	victoriapookae	KP150419.1
Hymenoptera	Chalcidoidea	Eulophidae	Euplectrus	wonyoungchoi	KP150329.1
Hymenoptera	Chalcidoidea	Eulophidae	Euplectrus	xiomarae	KP150310.1
Hymenoptera	Chalcidoidea	Eulophidae	Euplectrus	xiomarae	KP150316.1
Hymenoptera	Chalcidoidea	Eulophidae	Euplectrus	xiomarae	KP150321.1
Hymenoptera	Chalcidoidea	Eulophidae	Euplectrus	xiomarae	KP150323.1
Hymenoptera	Chalcidoidea	Eulophidae	Euplectrus	xiomarae	KP150339.1
Hymenoptera	Chalcidoidea	Eulophidae	Euplectrus	xiomarae	KP150341.1
Hymenoptera	Chalcidoidea	Eulophidae	Euplectrus	xiomarae	KP150350.1
Hymenoptera	Chalcidoidea	Eulophidae	Euplectrus	xiomarae	KP150352.1
Hymenoptera	Chalcidoidea	Eulophidae	Euplectrus	xiomarae	KP150377.1
Hymenoptera	Chalcidoidea	Eulophidae	Euplectrus	xiomarae	KP150388.1
Hymenoptera	Chalcidoidea	Eulophidae	Euplectrus	xiomarae	KP150397.1
Hymenoptera	Chalcidoidea	Eulophidae	Euplectrus	xiomarae	KP150425.1
Hymenoptera	Chalcidoidea	Eulophidae	Hadrotrichodes	waukheon	HM365053.1
Hymenoptera	Chalcidoidea	Eulophidae	Hemiptarsenus	varicornis	KY834054.1
Hymenoptera	Chalcidoidea	Eulophidae	Hemiptarsenus	varicornis	KY838269.1
Hymenoptera	Chalcidoidea	Eulophidae	Holcotetrastichus	sp.	KF444818.1
Hymenoptera	Chalcidoidea	Eulophidae	Horismenus	petiolatus	HM365039.1
Hymenoptera	Chalcidoidea	Eulophidae	Hyssopus	novus	KR797611.1
Hymenoptera	Chalcidoidea	Eulophidae	Hyssopus	pallidus	KP072613.1
Hymenoptera	Chalcidoidea	Eulophidae	Leptocybe	invasa	JQ289999.1
Hymenoptera	Chalcidoidea	Eulophidae	Leptocybe	invasa	JQ290004.1
Hymenoptera	Chalcidoidea	Eulophidae	Leptocybe	invasa	KP233953.1
Hymenoptera	Chalcidoidea	Eulophidae	Leptocybe	invasa	KP233954.1
Hymenoptera	Chalcidoidea	Eulophidae	Leptocybe	invasa	KP233972.1
Hymenoptera	Chalcidoidea	Eulophidae	Leptocybe	invasa	KP233985.1
Hymenoptera	Chalcidoidea	Eulophidae	Leptocybe	invasa	KP233989.1
Hymenoptera	Chalcidoidea	Eulophidae	Leptocybe	invasa	KP233990.1
Hymenoptera	Chalcidoidea	Eulophidae	Leptocybe	invasa	MH093001.1
Hymenoptera	Chalcidoidea	Eulophidae	Leptocybe	invasa	MH093002.1
Hymenoptera	Chalcidoidea	Eulophidae	Leptocybe	invasa	MH093006.1
Hymenoptera	Chalcidoidea	Eulophidae	Leptocybe	invasa	MH093010.1
Hymenoptera	Chalcidoidea	Eulophidae	Leptocybe	invasa	MH093043.1

Hymenoptera	Chalcidoidea	Eulophidae	Leptocybe	invasa	MH093047.1
Hymenoptera	Chalcidoidea	Eulophidae	Leptocybe	invasa	MH093048.1
Hymenoptera	Chalcidoidea	Eulophidae	Leptocybe	invasa	MH093052.1
Hymenoptera	Chalcidoidea	Eulophidae	Leptocybe	invasa	MH093053.1
Hymenoptera	Chalcidoidea	Eulophidae	Leptocybe	invasa	MH093054.1
Hymenoptera	Chalcidoidea	Eulophidae	Leptocybe	invasa	MH093057.1
Hymenoptera	Chalcidoidea	Eulophidae	Leptocybe	invasa	MH093063.1
Hymenoptera	Chalcidoidea	Eulophidae	Leptocybe	invasa	MH093064.1
Hymenoptera	Chalcidoidea	Eulophidae	Leptocybe	invasa	MH093065.1
Hymenoptera	Chalcidoidea	Eulophidae	Leptocybe	invasa	MH093071.1
Hymenoptera	Chalcidoidea	Eulophidae	Leptocybe	invasa	MH093079.1
Hymenoptera	Chalcidoidea	Eulophidae	Leptocybe	invasa	MH093080.1
Hymenoptera	Chalcidoidea	Eulophidae	Leptocybe	invasa	MH093082.1
Hymenoptera	Chalcidoidea	Eulophidae	Leptocybe	invasa	MH093087.1
Hymenoptera	Chalcidoidea	Eulophidae	Leptocybe	invasa	MH093089.1
Hymenoptera	Chalcidoidea	Eulophidae	Leptocybe	invasa	MH093092.1
Hymenoptera	Chalcidoidea	Eulophidae	Leptocybe	invasa	MH093111.1
Hymenoptera	Chalcidoidea	Eulophidae	Leptocybe	invasa	MH093182.1
Hymenoptera	Chalcidoidea	Eulophidae	Leptocybe	invasa	MH093185.1
Hymenoptera	Chalcidoidea	Eulophidae	Leptocybe	invasa	MH093186.1
Hymenoptera	Chalcidoidea	Eulophidae	Leptocybe	invasa	MN524231.1
Hymenoptera	Chalcidoidea	Eulophidae	Melittobia	sp.	MG263515.1
Hymenoptera	Chalcidoidea	Eulophidae	Minotetrastichus	sp.	KJ086069.1
Hymenoptera	Chalcidoidea	Eulophidae	Minotetrastichus	sp.	KJ087409.1
Hymenoptera	Chalcidoidea	Eulophidae	Minotetrastichus	sp.	KJ092505.1
Hymenoptera	Chalcidoidea	Eulophidae	Minotetrastichus	sp.	MG445591.1
Hymenoptera	Chalcidoidea	Eulophidae	Necremnus	tidius	KR886785.1
Hymenoptera	Chalcidoidea	Eulophidae	Neochrysocharis	beasleyi	MG836430.1
Hymenoptera	Chalcidoidea	Eulophidae	Neochrysocharis	chlorogaster	MZ631606.1
Hymenoptera	Chalcidoidea	Eulophidae	Neochrysocharis	clinias	HM365038.1
Hymenoptera	Chalcidoidea	Eulophidae	Neochrysocharis	formosa	HM365028.1
Hymenoptera	Chalcidoidea	Eulophidae	Neochrysocharis	formosa	LC542890.1
Hymenoptera	Chalcidoidea	Eulophidae	Neochrysocharis	formosa	MG836429.1
Hymenoptera	Chalcidoidea	Eulophidae	Neochrysocharis	formosa	MH841902.1
Hymenoptera	Chalcidoidea	Eulophidae	Neochrysocharis	formosa	MH841903.1
Hymenoptera	Chalcidoidea	Eulophidae	Neochrysocharis	sp.	JN293504.1
Hymenoptera	Chalcidoidea	Eulophidae	Neochrysocharis	sp.	KJ165214.1
Hymenoptera	Chalcidoidea	Eulophidae	Neochrysocharis	sp.	KR801248.1
Hymenoptera	Chalcidoidea	Eulophidae	Neochrysocharis	sp.	KR884381.1
Hymenoptera	Chalcidoidea	Eulophidae	Neochrysocharis	sp.	KR886017.1
Hymenoptera	Chalcidoidea	Eulophidae	Neochrysocharis	sp.	KR900797.1
Hymenoptera	Chalcidoidea	Eulophidae	Neochrysocharis	sp.	KR930921.1
Hymenoptera	Chalcidoidea	Eulophidae	Neochrysocharis	sp.	KR934038.1
Hymenoptera	Chalcidoidea	Eulophidae	Neochrysocharis	sp.	MF904182.1
Hymenoptera	Chalcidoidea	Eulophidae	Neochrysocharis	sp.	MG336853.1
Hymenoptera	Chalcidoidea	Eulophidae	Neochrysocharis	sp.	MG337243.1
Hymenoptera	Chalcidoidea	Eulophidae	Neochrysocharis	sp.	MG340024.1
Hymenoptera	Chalcidoidea	Eulophidae	Neochrysocharis	sp.	MG342272.1
Hymenoptera	Chalcidoidea	Eulophidae	Neochrysocharis	sp.	MG344241.1
Hymenoptera	Chalcidoidea	Eulophidae	Neochrysocharis	sp.	MG344374.1
Hymenoptera	Chalcidoidea	Eulophidae	Ogmoelachertus	sp.	HQ548195.1
Hymenoptera	Chalcidoidea	Eulophidae	Omphale	radialis	HM365033.1
Hymenoptera	Chalcidoidea	Eulophidae	Ophelimus	maskelli	HM365046.1
Hymenoptera	Chalcidoidea	Eulophidae	Ophelimus	sp.	MK155058.1

Hymenoptera	Chalcidoidea	Eulophidae	Pediobius	albipes	MT609868.1
Hymenoptera	Chalcidoidea	Eulophidae	Pediobius	cassidae	MG836447.1
Hymenoptera	Chalcidoidea	Eulophidae	Pediobius	eubius	MG836445.1
Hymenoptera	Chalcidoidea	Eulophidae	Pediobius	facialis	MG836444.1
Hymenoptera	Chalcidoidea	Eulophidae	Pediobius	foliorum	MG836446.1
Hymenoptera	Chalcidoidea	Eulophidae	Pediobius	metallicus	LC542891.1
Hymenoptera	Chalcidoidea	Eulophidae	Pediobius	metallicus	MG836438.1
Hymenoptera	Chalcidoidea	Eulophidae	Pediobius	metallicus	MG836439.1
Hymenoptera	Chalcidoidea	Eulophidae	Pediobius	metallicus	MG836440.1
Hymenoptera	Chalcidoidea	Eulophidae	Pediobius	ocellatus	MT609870.1
Hymenoptera	Chalcidoidea	Eulophidae	Pediobius	ocellatus	MT609880.1
Hymenoptera	Chalcidoidea	Eulophidae	Pediobius	pyrgo	MG836441.1
Hymenoptera	Chalcidoidea	Eulophidae	Pediobius	pyrgo	MG836442.1
Hymenoptera	Chalcidoidea	Eulophidae	Pediobius	pyrgo	MG836443.1
Hymenoptera	Chalcidoidea	Eulophidae	Pediobius	saulius	GU087051.1
Hymenoptera	Chalcidoidea	Eulophidae	Pediobius	saulius	GU087054.1
Hymenoptera	Chalcidoidea	Eulophidae	Pediobius	saulius	GU087055.1
Hymenoptera	Chalcidoidea	Eulophidae	Pediobius	saulius	GU087056.1
Hymenoptera	Chalcidoidea	Eulophidae	Pediobius	saulius	GU087060.1
Hymenoptera	Chalcidoidea	Eulophidae	Pediobius	saulius	GU087062.1
Hymenoptera	Chalcidoidea	Eulophidae	Pediobius	saulius	GU087065.1
Hymenoptera	Chalcidoidea	Eulophidae	Pediobius	saulius	GU087085.1
Hymenoptera	Chalcidoidea	Eulophidae	Pediobius	saulius	GU087086.1
Hymenoptera	Chalcidoidea	Eulophidae	Pediobius	saulius	GU087090.1
Hymenoptera	Chalcidoidea	Eulophidae	Pediobius	saulius	GU087097.1
Hymenoptera	Chalcidoidea	Eulophidae	Pediobius	saulius	GU087103.1
Hymenoptera	Chalcidoidea	Eulophidae	Pediobius	saulius	GU087114.1
Hymenoptera	Chalcidoidea	Eulophidae	Pediobius	saulius	GU087120.1
Hymenoptera	Chalcidoidea	Eulophidae	Pediobius	saulius	GU087121.1
Hymenoptera	Chalcidoidea	Eulophidae	Pediobius	saulius	GU087123.1
Hymenoptera	Chalcidoidea	Eulophidae	Pediobius	saulius	GU087124.1
Hymenoptera	Chalcidoidea	Eulophidae	Pediobius	saulius	GU087128.1
Hymenoptera	Chalcidoidea	Eulophidae	Pediobius	saulius	MZ632543.1
Hymenoptera	Chalcidoidea	Eulophidae	Pediobius	sp.	HM365043.1
Hymenoptera	Chalcidoidea	Eulophidae	Pediobius	sp.	HQ107668.1
Hymenoptera	Chalcidoidea	Eulophidae	Pediobius	sp.	HQ107669.1
Hymenoptera	Chalcidoidea	Eulophidae	Pediobius	sp.	HQ107670.1
Hymenoptera	Chalcidoidea	Eulophidae	Pediobius	sp.	HQ107671.1
Hymenoptera	Chalcidoidea	Eulophidae	Pediobius	sp.	HQ107673.1
Hymenoptera	Chalcidoidea	Eulophidae	Pediobius	sp.	KC808624.1
Hymenoptera	Chalcidoidea	Eulophidae	Pediobius	sp.	KC808630.1
Hymenoptera	Chalcidoidea	Eulophidae	Pediobius	sp.	KR796772.1
Hymenoptera	Chalcidoidea	Eulophidae	Pediobius	sp.	KR804258.1
Hymenoptera	Chalcidoidea	Eulophidae	Pediobius	sp.	MG445897.1
Hymenoptera	Chalcidoidea	Eulophidae	Pediobius	sp.	MZ630610.1
Hymenoptera	Chalcidoidea	Eulophidae	Pediobomyia	canaliculata	HM365042.1
Hymenoptera	Chalcidoidea	Eulophidae	Pnigalio	agraules	MZ629184.1
Hymenoptera	Chalcidoidea	Eulophidae	Pnigalio	agraules	MZ629544.1
Hymenoptera	Chalcidoidea	Eulophidae	Pnigalio	agraules	MZ630210.1
Hymenoptera	Chalcidoidea	Eulophidae	Pnigalio	agraules	MZ630621.1
Hymenoptera	Chalcidoidea	Eulophidae	Pnigalio	agraules	MZ630698.1
Hymenoptera	Chalcidoidea	Eulophidae	Pnigalio	agraules	MZ631959.1
Hymenoptera	Chalcidoidea	Eulophidae	Pnigalio	agraules	MZ633156.1
Hymenoptera	Chalcidoidea	Eulophidae	Pnigalio	agraules	MZ633867.1

Hymenoptera	Chalcidoidea	Eulophidae	Pnigalio	audax	MG836494.1
Hymenoptera	Chalcidoidea	Eulophidae	Pnigalio	katonis	LC542892.1
Hymenoptera	Chalcidoidea	Eulophidae	Pnigalio	longulus	MZ630591.1
Hymenoptera	Chalcidoidea	Eulophidae	Pnigalio	longulus	MZ632695.1
Hymenoptera	Chalcidoidea	Eulophidae	Pnigalio	maculipes	MF900117.1
Hymenoptera	Chalcidoidea	Eulophidae	Pnigalio	maculipes	MF907131.1
Hymenoptera	Chalcidoidea	Eulophidae	Pnigalio	maculipes	MG342670.1
Hymenoptera	Chalcidoidea	Eulophidae	Pnigalio	maculipes	MG343733.1
Hymenoptera	Chalcidoidea	Eulophidae	Pnigalio	minio	KF444816.1
Hymenoptera	Chalcidoidea	Eulophidae	Pnigalio	minio	KR795786.1
Hymenoptera	Chalcidoidea	Eulophidae	Pnigalio	minio	MG342811.1
Hymenoptera	Chalcidoidea	Eulophidae	Pnigalio	pallipes	HM423377.1
Hymenoptera	Chalcidoidea	Eulophidae	Pnigalio	soemius	MZ660075.1
Hymenoptera	Chalcidoidea	Eulophidae	Pnigalio	sp.	HM365052.1
Hymenoptera	Chalcidoidea	Eulophidae	Pnigalio	sp.	HQ552501.1
Hymenoptera	Chalcidoidea	Eulophidae	Pnigalio	sp.	KR403134.1
Hymenoptera	Chalcidoidea	Eulophidae	Pnigalio	sp.	KR403798.1
Hymenoptera	Chalcidoidea	Eulophidae	Pnigalio	sp.	KR406335.1
Hymenoptera	Chalcidoidea	Eulophidae	Pnigalio	sp.	KR407260.1
Hymenoptera	Chalcidoidea	Eulophidae	Pnigalio	sp.	KR410460.1
Hymenoptera	Chalcidoidea	Eulophidae	Pnigalio	sp.	KR411828.1
Hymenoptera	Chalcidoidea	Eulophidae	Pnigalio	sp.	KR412123.1
Hymenoptera	Chalcidoidea	Eulophidae	Pnigalio	sp.	KR414076.1
Hymenoptera	Chalcidoidea	Eulophidae	Pnigalio	sp.	KR417247.1
Hymenoptera	Chalcidoidea	Eulophidae	Pnigalio	sp.	KR421099.1
Hymenoptera	Chalcidoidea	Eulophidae	Pnigalio	sp.	KR421533.1
Hymenoptera	Chalcidoidea	Eulophidae	Pnigalio	sp.	KR784873.1
Hymenoptera	Chalcidoidea	Eulophidae	Pnigalio	sp.	KR786765.1
Hymenoptera	Chalcidoidea	Eulophidae	Pnigalio	sp.	KR786785.1
Hymenoptera	Chalcidoidea	Eulophidae	Pnigalio	sp.	KR789222.1
Hymenoptera	Chalcidoidea	Eulophidae	Pnigalio	sp.	KR789464.1
Hymenoptera	Chalcidoidea	Eulophidae	Pnigalio	sp.	KR791965.1
Hymenoptera	Chalcidoidea	Eulophidae	Pnigalio	sp.	KR801876.1
Hymenoptera	Chalcidoidea	Eulophidae	Pnigalio	sp.	KR802080.1
Hymenoptera	Chalcidoidea	Eulophidae	Pnigalio	sp.	KR808475.1
Hymenoptera	Chalcidoidea	Eulophidae	Pnigalio	sp.	KR875427.1
Hymenoptera	Chalcidoidea	Eulophidae	Pnigalio	sp.	KR876244.1
Hymenoptera	Chalcidoidea	Eulophidae	Pnigalio	sp.	KR876592.1
Hymenoptera	Chalcidoidea	Eulophidae	Pnigalio	sp.	KR876703.1
Hymenoptera	Chalcidoidea	Eulophidae	Pnigalio	sp.	KR881254.1
Hymenoptera	Chalcidoidea	Eulophidae	Pnigalio	sp.	KR882003.1
Hymenoptera	Chalcidoidea	Eulophidae	Pnigalio	sp.	KR884903.1
Hymenoptera	Chalcidoidea	Eulophidae	Pnigalio	sp.	KR885851.1
Hymenoptera	Chalcidoidea	Eulophidae	Pnigalio	sp.	KR891352.1
Hymenoptera	Chalcidoidea	Eulophidae	Pnigalio	sp.	KR891492.1
Hymenoptera	Chalcidoidea	Eulophidae	Pnigalio	sp.	KR893872.1
Hymenoptera	Chalcidoidea	Eulophidae	Pnigalio	sp.	KR896682.1
Hymenoptera	Chalcidoidea	Eulophidae	Pnigalio	sp.	KR902181.1
Hymenoptera	Chalcidoidea	Eulophidae	Pnigalio	sp.	MF902957.1
Hymenoptera	Chalcidoidea	Eulophidae	Pnigalio	sp.	MG336810.1
Hymenoptera	Chalcidoidea	Eulophidae	Pnigalio	sp.	MG337603.1
Hymenoptera	Chalcidoidea	Eulophidae	Pnigalio	sp.	MG340287.1
Hymenoptera	Chalcidoidea	Eulophidae	Pnigalio	sp.	MW784445.1
Hymenoptera	Chalcidoidea	Eulophidae	Pnigalio	sp.	MZ629726.1

Hymenoptera	Chalcidoidea	Eulophidae	Pnigalio	sp.	MZ631032.1
Hymenoptera	Chalcidoidea	Eulophidae	Pnigalio	sp.	MZ631286.1
Hymenoptera	Chalcidoidea	Eulophidae	Pnigalio	sp.	MZ632179.1
Hymenoptera	Chalcidoidea	Eulophidae	Pnigalio	sp.	MZ632326.1
Hymenoptera	Chalcidoidea	Eulophidae	Pnigalio	sp.	MZ632567.1
Hymenoptera	Chalcidoidea	Eulophidae	Pnigalio	sp.	MZ632690.1
Hymenoptera	Chalcidoidea	Eulophidae	Pnigalio	sp.	MZ633229.1
Hymenoptera	Chalcidoidea	Eulophidae	Pnigalio	sp.	MZ633473.1
Hymenoptera	Chalcidoidea	Eulophidae	Pnigalio	sp.	MZ633610.1
Hymenoptera	Chalcidoidea	Eulophidae	Pnigalio	sp.	MZ657585.1
Hymenoptera	Chalcidoidea	Eulophidae	Pronotalia	sp.	KR782828.1
Hymenoptera	Chalcidoidea	Eulophidae	Pronotalia	sp.	KR785817.1
Hymenoptera	Chalcidoidea	Eulophidae	Pronotalia	sp.	KR786749.1
Hymenoptera	Chalcidoidea	Eulophidae	Pronotalia	sp.	KR794773.1
Hymenoptera	Chalcidoidea	Eulophidae	Pronotalia	sp.	KR796088.1
Hymenoptera	Chalcidoidea	Eulophidae	Pronotalia	sp.	KR803646.1
Hymenoptera	Chalcidoidea	Eulophidae	Pronotalia	sp.	KR809088.1
Hymenoptera	Chalcidoidea	Eulophidae	Pronotalia	sp.	KR889695.1
Hymenoptera	Chalcidoidea	Eulophidae	Pronotalia	sp.	KR926015.1
Hymenoptera	Chalcidoidea	Eulophidae	Quadrastichus	vacuna	KR797201.1
Hymenoptera	Chalcidoidea	Eulophidae	Rhynchentedon	maximus	HM365032.1
Hymenoptera	Chalcidoidea	Eulophidae	Sympiesis	ancylae	KJ207927.1
Hymenoptera	Chalcidoidea	Eulophidae	Sympiesis	ancylae	KR879071.1
Hymenoptera	Chalcidoidea	Eulophidae	Sympiesis	dolichogaster	MN525188.1
Hymenoptera	Chalcidoidea	Eulophidae	Sympiesis	gordius	MZ657049.1
Hymenoptera	Chalcidoidea	Eulophidae	Sympiesis	sericeicornis	KR792875.1
Hymenoptera	Chalcidoidea	Eulophidae	Sympiesis	sericeicornis	MZ629235.1
Hymenoptera	Chalcidoidea	Eulophidae	Sympiesis	sericeicornis	MZ631106.1
Hymenoptera	Chalcidoidea	Eulophidae	Sympiesis	sericeicornis	MZ633120.1
Hymenoptera	Chalcidoidea	Eulophidae	Sympiesis	sericeicornis	MZ633602.1
Hymenoptera	Chalcidoidea	Eulophidae	Sympiesis	sp.	KR784108.1
Hymenoptera	Chalcidoidea	Eulophidae	Sympiesis	sp.	KR794214.1
Hymenoptera	Chalcidoidea	Eulophidae	Sympiesis	sp.	KR804939.1
Hymenoptera	Chalcidoidea	Eulophidae	Sympiesis	sp.	KR888794.1
Hymenoptera	Chalcidoidea	Eulophidae	Sympiesis	sp.	MG339828.1
Hymenoptera	Chalcidoidea	Eulophidae	Sympiesis	stigmata	KR878039.1
Hymenoptera	Chalcidoidea	Eulophidae	Tamarixia	dryi	MK293946.1
Hymenoptera	Chalcidoidea	Eulophidae	Tamarixia	dryi	MK293948.1
Hymenoptera	Chalcidoidea	Eulophidae	Tamarixia	dryi	MK293949.1
Hymenoptera	Chalcidoidea	Eulophidae	Tamarixia	dryi	MK293950.1
Hymenoptera	Chalcidoidea	Eulophidae	Tamarixia	radiata	KT253009.1
Hymenoptera	Chalcidoidea	Eulophidae	Tamarixia	sp.	MK302489.1
Hymenoptera	Chalcidoidea	Eulophidae	Tetrastichinae	sp.	GU675356.1
Hymenoptera	Chalcidoidea	Eulophidae	Tetrastichinae	sp.	GU675360.1
Hymenoptera	Chalcidoidea	Eulophidae	Tetrastichinae	sp.	HM374824.1
Hymenoptera	Chalcidoidea	Eulophidae	Tetrastichinae	sp.	HM374825.1
Hymenoptera	Chalcidoidea	Eulophidae	Tetrastichinae	sp.	HM374827.1
Hymenoptera	Chalcidoidea	Eulophidae	Tetrastichinae	sp.	HM414500.1
Hymenoptera	Chalcidoidea	Eulophidae	Tetrastichinae	sp.	HM414519.1
Hymenoptera	Chalcidoidea	Eulophidae	Tetrastichinae	sp.	HM414605.1
Hymenoptera	Chalcidoidea	Eulophidae	Tetrastichinae	sp.	HM414635.1
Hymenoptera	Chalcidoidea	Eulophidae	Tetrastichinae	sp.	HM414651.1
Hymenoptera	Chalcidoidea	Eulophidae	Tetrastichinae	sp.	HM432909.1
Hymenoptera	Chalcidoidea	Eulophidae	Tetrastichinae	sp.	HM883317.1

Hymenoptera	Chalcidoidea	Eulophidae	Tetrastichinae	sp.	HQ566525.1
Hymenoptera	Chalcidoidea	Eulophidae	Tetrastichinae	sp.	JN292486.1
Hymenoptera	Chalcidoidea	Eulophidae	Tetrastichinae	sp.	KJ091511.1
Hymenoptera	Chalcidoidea	Eulophidae	Tetrastichinae	sp.	KJ209467.1
Hymenoptera	Chalcidoidea	Eulophidae	Tetrastichinae	sp.	KR401506.1
Hymenoptera	Chalcidoidea	Eulophidae	Tetrastichinae	sp.	KR402000.1
Hymenoptera	Chalcidoidea	Eulophidae	Tetrastichinae	sp.	KR402101.1
Hymenoptera	Chalcidoidea	Eulophidae	Tetrastichinae	sp.	KR402565.1
Hymenoptera	Chalcidoidea	Eulophidae	Tetrastichinae	sp.	KR402868.1
Hymenoptera	Chalcidoidea	Eulophidae	Tetrastichinae	sp.	KR402953.1
Hymenoptera	Chalcidoidea	Eulophidae	Tetrastichinae	sp.	KR402982.1
Hymenoptera	Chalcidoidea	Eulophidae	Tetrastichinae	sp.	KR404524.1
Hymenoptera	Chalcidoidea	Eulophidae	Tetrastichinae	sp.	KR404599.1
Hymenoptera	Chalcidoidea	Eulophidae	Tetrastichinae	sp.	KR404831.1
Hymenoptera	Chalcidoidea	Eulophidae	Tetrastichinae	sp.	KR405516.1
Hymenoptera	Chalcidoidea	Eulophidae	Tetrastichinae	sp.	KR405796.1
Hymenoptera	Chalcidoidea	Eulophidae	Tetrastichinae	sp.	KR408172.1
Hymenoptera	Chalcidoidea	Eulophidae	Tetrastichinae	sp.	KR408332.1
Hymenoptera	Chalcidoidea	Eulophidae	Tetrastichinae	sp.	KR409007.1
Hymenoptera	Chalcidoidea	Eulophidae	Tetrastichinae	sp.	KR409082.1
Hymenoptera	Chalcidoidea	Eulophidae	Tetrastichinae	sp.	KR411332.1
Hymenoptera	Chalcidoidea	Eulophidae	Tetrastichinae	sp.	KR411347.1
Hymenoptera	Chalcidoidea	Eulophidae	Tetrastichinae	sp.	KR412455.1
Hymenoptera	Chalcidoidea	Eulophidae	Tetrastichinae	sp.	KR412765.1
Hymenoptera	Chalcidoidea	Eulophidae	Tetrastichinae	sp.	KR412884.1
Hymenoptera	Chalcidoidea	Eulophidae	Tetrastichinae	sp.	KR413209.1
Hymenoptera	Chalcidoidea	Eulophidae	Tetrastichinae	sp.	KR413470.1
Hymenoptera	Chalcidoidea	Eulophidae	Tetrastichinae	sp.	KR413597.1
Hymenoptera	Chalcidoidea	Eulophidae	Tetrastichinae	sp.	KR414037.1
Hymenoptera	Chalcidoidea	Eulophidae	Tetrastichinae	sp.	KR414870.1
Hymenoptera	Chalcidoidea	Eulophidae	Tetrastichinae	sp.	KR415477.1
Hymenoptera	Chalcidoidea	Eulophidae	Tetrastichinae	sp.	KR415556.1
Hymenoptera	Chalcidoidea	Eulophidae	Tetrastichinae	sp.	KR415877.1
Hymenoptera	Chalcidoidea	Eulophidae	Tetrastichinae	sp.	KR416043.1
Hymenoptera	Chalcidoidea	Eulophidae	Tetrastichinae	sp.	KR416123.1
Hymenoptera	Chalcidoidea	Eulophidae	Tetrastichinae	sp.	KR416767.1
Hymenoptera	Chalcidoidea	Eulophidae	Tetrastichinae	sp.	KR416826.1
Hymenoptera	Chalcidoidea	Eulophidae	Tetrastichinae	sp.	KR416941.1
Hymenoptera	Chalcidoidea	Eulophidae	Tetrastichinae	sp.	KR417767.1
Hymenoptera	Chalcidoidea	Eulophidae	Tetrastichinae	sp.	KR418064.1
Hymenoptera	Chalcidoidea	Eulophidae	Tetrastichinae	sp.	KR418197.1
Hymenoptera	Chalcidoidea	Eulophidae	Tetrastichinae	sp.	KR419273.1
Hymenoptera	Chalcidoidea	Eulophidae	Tetrastichinae	sp.	KR419663.1
Hymenoptera	Chalcidoidea	Eulophidae	Tetrastichinae	sp.	KR420727.1
Hymenoptera	Chalcidoidea	Eulophidae	Tetrastichinae	sp.	KR420991.1
Hymenoptera	Chalcidoidea	Eulophidae	Tetrastichinae	sp.	KR421422.1
Hymenoptera	Chalcidoidea	Eulophidae	Tetrastichinae	sp.	KR421710.1
Hymenoptera	Chalcidoidea	Eulophidae	Tetrastichinae	sp.	KR421797.1
Hymenoptera	Chalcidoidea	Eulophidae	Tetrastichinae	sp.	KR422156.1
Hymenoptera	Chalcidoidea	Eulophidae	Tetrastichinae	sp.	KR422174.1
Hymenoptera	Chalcidoidea	Eulophidae	Tetrastichinae	sp.	KR783742.1
Hymenoptera	Chalcidoidea	Eulophidae	Tetrastichinae	sp.	KR784044.1
Hymenoptera	Chalcidoidea	Eulophidae	Tetrastichinae	sp.	KR784294.1
Hymenoptera	Chalcidoidea	Eulophidae	Tetrastichinae	sp.	KR784999.1

Hymenoptera	Chalcidoidea	Eulophidae	Tetrastichinae	sp.	KR785920.1
Hymenoptera	Chalcidoidea	Eulophidae	Tetrastichinae	sp.	KR786188.1
Hymenoptera	Chalcidoidea	Eulophidae	Tetrastichinae	sp.	KR786487.1
Hymenoptera	Chalcidoidea	Eulophidae	Tetrastichinae	sp.	KR787291.1
Hymenoptera	Chalcidoidea	Eulophidae	Tetrastichinae	sp.	KR793135.1
Hymenoptera	Chalcidoidea	Eulophidae	Tetrastichinae	sp.	KR793213.1
Hymenoptera	Chalcidoidea	Eulophidae	Tetrastichinae	sp.	KR793324.1
Hymenoptera	Chalcidoidea	Eulophidae	Tetrastichinae	sp.	KR793530.1
Hymenoptera	Chalcidoidea	Eulophidae	Tetrastichinae	sp.	KR793791.1
Hymenoptera	Chalcidoidea	Eulophidae	Tetrastichinae	sp.	KR794391.1
Hymenoptera	Chalcidoidea	Eulophidae	Tetrastichinae	sp.	KR794718.1
Hymenoptera	Chalcidoidea	Eulophidae	Tetrastichinae	sp.	KR795160.1
Hymenoptera	Chalcidoidea	Eulophidae	Tetrastichinae	sp.	KR795253.1
Hymenoptera	Chalcidoidea	Eulophidae	Tetrastichinae	sp.	KR795510.1
Hymenoptera	Chalcidoidea	Eulophidae	Tetrastichinae	sp.	KR796128.1
Hymenoptera	Chalcidoidea	Eulophidae	Tetrastichinae	sp.	KR796286.1
Hymenoptera	Chalcidoidea	Eulophidae	Tetrastichinae	sp.	KR796855.1
Hymenoptera	Chalcidoidea	Eulophidae	Tetrastichinae	sp.	KR797284.1
Hymenoptera	Chalcidoidea	Eulophidae	Tetrastichinae	sp.	KR799172.1
Hymenoptera	Chalcidoidea	Eulophidae	Tetrastichinae	sp.	KR800349.1
Hymenoptera	Chalcidoidea	Eulophidae	Tetrastichinae	sp.	KR800373.1
Hymenoptera	Chalcidoidea	Eulophidae	Tetrastichinae	sp.	KR800734.1
Hymenoptera	Chalcidoidea	Eulophidae	Tetrastichinae	sp.	KR802079.1
Hymenoptera	Chalcidoidea	Eulophidae	Tetrastichinae	sp.	KR802314.1
Hymenoptera	Chalcidoidea	Eulophidae	Tetrastichinae	sp.	KR802679.1
Hymenoptera	Chalcidoidea	Eulophidae	Tetrastichinae	sp.	KR802853.1
Hymenoptera	Chalcidoidea	Eulophidae	Tetrastichinae	sp.	KR803192.1
Hymenoptera	Chalcidoidea	Eulophidae	Tetrastichinae	sp.	KR803741.1
Hymenoptera	Chalcidoidea	Eulophidae	Tetrastichinae	sp.	KR804737.1
Hymenoptera	Chalcidoidea	Eulophidae	Tetrastichinae	sp.	KR804910.1
Hymenoptera	Chalcidoidea	Eulophidae	Tetrastichinae	sp.	KR805826.1
Hymenoptera	Chalcidoidea	Eulophidae	Tetrastichinae	sp.	KR807283.1
Hymenoptera	Chalcidoidea	Eulophidae	Tetrastichinae	sp.	KR808393.1
Hymenoptera	Chalcidoidea	Eulophidae	Tetrastichinae	sp.	KR808744.1
Hymenoptera	Chalcidoidea	Eulophidae	Tetrastichinae	sp.	KR809009.1
Hymenoptera	Chalcidoidea	Eulophidae	Tetrastichinae	sp.	KR877273.1
Hymenoptera	Chalcidoidea	Eulophidae	Tetrastichinae	sp.	KR879228.1
Hymenoptera	Chalcidoidea	Eulophidae	Tetrastichinae	sp.	KR880146.1
Hymenoptera	Chalcidoidea	Eulophidae	Tetrastichinae	sp.	KR880491.1
Hymenoptera	Chalcidoidea	Eulophidae	Tetrastichinae	sp.	KR882054.1
Hymenoptera	Chalcidoidea	Eulophidae	Tetrastichinae	sp.	KR883532.1
Hymenoptera	Chalcidoidea	Eulophidae	Tetrastichinae	sp.	KR883816.1
Hymenoptera	Chalcidoidea	Eulophidae	Tetrastichinae	sp.	KR885525.1
Hymenoptera	Chalcidoidea	Eulophidae	Tetrastichinae	sp.	KR885528.1
Hymenoptera	Chalcidoidea	Eulophidae	Tetrastichinae	sp.	KR887465.1
Hymenoptera	Chalcidoidea	Eulophidae	Tetrastichinae	sp.	KR890408.1
Hymenoptera	Chalcidoidea	Eulophidae	Tetrastichinae	sp.	KR891654.1
Hymenoptera	Chalcidoidea	Eulophidae	Tetrastichinae	sp.	KR893736.1
Hymenoptera	Chalcidoidea	Eulophidae	Tetrastichinae	sp.	KR894488.1
Hymenoptera	Chalcidoidea	Eulophidae	Tetrastichinae	sp.	KR895094.1
Hymenoptera	Chalcidoidea	Eulophidae	Tetrastichinae	sp.	KR895593.1
Hymenoptera	Chalcidoidea	Eulophidae	Tetrastichinae	sp.	KR895752.1
Hymenoptera	Chalcidoidea	Eulophidae	Tetrastichinae	sp.	KR925865.1
Hymenoptera	Chalcidoidea	Eulophidae	Tetrastichinae	sp.	KR929455.1

Hymenoptera	Chalcidoidea	Eulophidae	Tetrastichinae	sp.	KT615121.1
Hymenoptera	Chalcidoidea	Eulophidae	Tetrastichinae	sp.	KT615908.1
Hymenoptera	Chalcidoidea	Eulophidae	Tetrastichinae	sp.	KT707231.1
Hymenoptera	Chalcidoidea	Eulophidae	Tetrastichinae	sp.	KX535205.1
Hymenoptera	Chalcidoidea	Eulophidae	Tetrastichinae	sp.	KY829623.1
Hymenoptera	Chalcidoidea	Eulophidae	Tetrastichinae	sp.	KY829855.1
Hymenoptera	Chalcidoidea	Eulophidae	Tetrastichinae	sp.	KY830149.1
Hymenoptera	Chalcidoidea	Eulophidae	Tetrastichinae	sp.	KY830164.1
Hymenoptera	Chalcidoidea	Eulophidae	Tetrastichinae	sp.	KY830928.1
Hymenoptera	Chalcidoidea	Eulophidae	Tetrastichinae	sp.	KY831207.1
Hymenoptera	Chalcidoidea	Eulophidae	Tetrastichinae	sp.	KY831808.1
Hymenoptera	Chalcidoidea	Eulophidae	Tetrastichinae	sp.	KY832137.1
Hymenoptera	Chalcidoidea	Eulophidae	Tetrastichinae	sp.	KY832314.1
Hymenoptera	Chalcidoidea	Eulophidae	Tetrastichinae	sp.	KY832363.1
Hymenoptera	Chalcidoidea	Eulophidae	Tetrastichinae	sp.	KY833673.1
Hymenoptera	Chalcidoidea	Eulophidae	Tetrastichinae	sp.	KY834044.1
Hymenoptera	Chalcidoidea	Eulophidae	Tetrastichinae	sp.	KY836136.1
Hymenoptera	Chalcidoidea	Eulophidae	Tetrastichinae	sp.	KY836459.1
Hymenoptera	Chalcidoidea	Eulophidae	Tetrastichinae	sp.	KY836480.1
Hymenoptera	Chalcidoidea	Eulophidae	Tetrastichinae	sp.	KY836812.1
Hymenoptera	Chalcidoidea	Eulophidae	Tetrastichinae	sp.	KY837135.1
Hymenoptera	Chalcidoidea	Eulophidae	Tetrastichinae	sp.	KY837143.1
Hymenoptera	Chalcidoidea	Eulophidae	Tetrastichinae	sp.	KY837672.1
Hymenoptera	Chalcidoidea	Eulophidae	Tetrastichinae	sp.	KY837910.1
Hymenoptera	Chalcidoidea	Eulophidae	Tetrastichinae	sp.	KY837960.1
Hymenoptera	Chalcidoidea	Eulophidae	Tetrastichinae	sp.	KY838200.1
Hymenoptera	Chalcidoidea	Eulophidae	Tetrastichinae	sp.	KY838730.1
Hymenoptera	Chalcidoidea	Eulophidae	Tetrastichinae	sp.	KY838878.1
Hymenoptera	Chalcidoidea	Eulophidae	Tetrastichinae	sp.	KY839112.1
Hymenoptera	Chalcidoidea	Eulophidae	Tetrastichinae	sp.	KY839560.1
Hymenoptera	Chalcidoidea	Eulophidae	Tetrastichinae	sp.	KY839793.1
Hymenoptera	Chalcidoidea	Eulophidae	Tetrastichinae	sp.	KY840659.1
Hymenoptera	Chalcidoidea	Eulophidae	Tetrastichinae	sp.	KY841214.1
Hymenoptera	Chalcidoidea	Eulophidae	Tetrastichinae	sp.	KY841764.1
Hymenoptera	Chalcidoidea	Eulophidae	Tetrastichinae	sp.	KY843486.1
Hymenoptera	Chalcidoidea	Eulophidae	Tetrastichinae	sp.	MF898514.1
Hymenoptera	Chalcidoidea	Eulophidae	Tetrastichinae	sp.	MF898755.1
Hymenoptera	Chalcidoidea	Eulophidae	Tetrastichinae	sp.	MF901204.1
Hymenoptera	Chalcidoidea	Eulophidae	Tetrastichinae	sp.	MF901233.1
Hymenoptera	Chalcidoidea	Eulophidae	Tetrastichinae	sp.	MF902020.1
Hymenoptera	Chalcidoidea	Eulophidae	Tetrastichinae	sp.	MF902157.1
Hymenoptera	Chalcidoidea	Eulophidae	Tetrastichinae	sp.	MF903352.1
Hymenoptera	Chalcidoidea	Eulophidae	Tetrastichinae	sp.	MF903768.1
Hymenoptera	Chalcidoidea	Eulophidae	Tetrastichinae	sp.	MF904221.1
Hymenoptera	Chalcidoidea	Eulophidae	Tetrastichinae	sp.	MF904731.1
Hymenoptera	Chalcidoidea	Eulophidae	Tetrastichinae	sp.	MF905071.1
Hymenoptera	Chalcidoidea	Eulophidae	Tetrastichinae	sp.	MF906773.1
Hymenoptera	Chalcidoidea	Eulophidae	Tetrastichinae	sp.	MF907580.1
Hymenoptera	Chalcidoidea	Eulophidae	Tetrastichinae	sp.	MG335357.1
Hymenoptera	Chalcidoidea	Eulophidae	Tetrastichinae	sp.	MG336440.1
Hymenoptera	Chalcidoidea	Eulophidae	Tetrastichinae	sp.	MG338354.1
Hymenoptera	Chalcidoidea	Eulophidae	Tetrastichinae	sp.	MG338938.1
Hymenoptera	Chalcidoidea	Eulophidae	Tetrastichinae	sp.	MG338942.1
Hymenoptera	Chalcidoidea	Eulophidae	Tetrastichinae	sp.	MG339590.1

Hymenoptera	Chalcidoidea	Eulophidae	Tetrastichinae	sp.	MG340871.1
Hymenoptera	Chalcidoidea	Eulophidae	Tetrastichinae	sp.	MG341164.1
Hymenoptera	Chalcidoidea	Eulophidae	Tetrastichinae	sp.	MG341277.1
Hymenoptera	Chalcidoidea	Eulophidae	Tetrastichinae	sp.	MG341386.1
Hymenoptera	Chalcidoidea	Eulophidae	Tetrastichinae	sp.	MG342834.1
Hymenoptera	Chalcidoidea	Eulophidae	Tetrastichinae	sp.	MG342904.1
Hymenoptera	Chalcidoidea	Eulophidae	Tetrastichinae	sp.	MG342939.1
Hymenoptera	Chalcidoidea	Eulophidae	Tetrastichinae	sp.	MG343086.1
Hymenoptera	Chalcidoidea	Eulophidae	Tetrastichinae	sp.	MG343132.1
Hymenoptera	Chalcidoidea	Eulophidae	Tetrastichinae	sp.	MG443756.1
Hymenoptera	Chalcidoidea	Eulophidae	Tetrastichinae	sp.	MG443859.1
Hymenoptera	Chalcidoidea	Eulophidae	Tetrastichinae	sp.	MG444646.1
Hymenoptera	Chalcidoidea	Eulophidae	Tetrastichinae	sp.	MG447266.1
Hymenoptera	Chalcidoidea	Eulophidae	Tetrastichinae	sp.	MH094892.1
Hymenoptera	Chalcidoidea	Eulophidae	Tetrastichinae	sp.	MH095100.1
Hymenoptera	Chalcidoidea	Eulophidae	Tetrastichinae	sp.	MH095143.1
Hymenoptera	Chalcidoidea	Eulophidae	Tetrastichinae	sp.	MH095477.1
Hymenoptera	Chalcidoidea	Eulophidae	Tetrastichinae	sp.	MH456697.1
Hymenoptera	Chalcidoidea	Eulophidae	Tetrastichinae	sp.	MN678167.1
Hymenoptera	Chalcidoidea	Eulophidae	Tetrastichinae	sp.	MN678679.1
Hymenoptera	Chalcidoidea	Eulophidae	Tetrastichinae	sp.	MW784476.1
Hymenoptera	Chalcidoidea	Eulophidae	Tetrastichinae	sp.	MZ632172.1
Hymenoptera	Chalcidoidea	Eulophidae	Tetrastichus	sp.	KR785019.1
Hymenoptera	Chalcidoidea	Eulophidae	Tetrastichus	sp.	KR926400.1
Hymenoptera	Chalcidoidea	Eulophidae	Tetrastichus	sp.	MG342565.1
Hymenoptera	Chalcidoidea	Eulophidae	Trisecodes	agromyzae	HM365055.1
Hymenoptera	Chalcidoidea	Eulophidae	UNVERIFIED	Asecodes	MG440650.1
Hymenoptera	Chalcidoidea	Eulophidae	UNVERIFIED	Encarsia	KC870908.1
Hymenoptera	Chalcidoidea	Eulophidae	UNVERIFIED	Encarsia	KC870909.1
Hymenoptera	Chalcidoidea	Eulophidae	UNVERIFIED	Encarsia	KC870910.1
Hymenoptera	Chalcidoidea	Eulophidae	UNVERIFIED	Encarsia	KC870913.1
Hymenoptera	Chalcidoidea	Eulophidae	UNVERIFIED	Eulophidae	MG441047.1
Hymenoptera	Chalcidoidea	Eulophidae	UNVERIFIED	Pediobius	KC991185.1
Hymenoptera	Chalcidoidea	Eulophinae	Eulophinae	sp.	JQ575781.1
Hymenoptera	Chalcidoidea	Eulophinae	Eulophinae	sp.	GU675350.1
Hymenoptera	Chalcidoidea	Eulophinae	Eulophinae	sp.	GU675351.1
Hymenoptera	Chalcidoidea	Eulophinae	Eulophinae	sp.	GU675357.1
Hymenoptera	Chalcidoidea	Eulophinae	Eulophinae	sp.	GU675364.1
Hymenoptera	Chalcidoidea	Eulophinae	Eulophinae	sp.	GU675365.1
Hymenoptera	Chalcidoidea	Eulophinae	Eulophinae	sp.	HM432911.1
Hymenoptera	Chalcidoidea	Eulophinae	Eulophinae	sp.	HQ972393.1
Hymenoptera	Chalcidoidea	Eulophinae	Eulophinae	sp.	JX830822.1
Hymenoptera	Chalcidoidea	Eulophinae	Eulophinae	sp.	JX831517.1
Hymenoptera	Chalcidoidea	Eulophinae	Eulophinae	sp.	KJ086598.1
Hymenoptera	Chalcidoidea	Eulophinae	Eulophinae	sp.	KJ166054.1
Hymenoptera	Chalcidoidea	Eulophinae	Eulophinae	sp.	KR795804.1
Hymenoptera	Chalcidoidea	Eulophinae	Eulophinae	sp.	KR796212.1
Hymenoptera	Chalcidoidea	Eulophinae	Eulophinae	sp.	KR801125.1
Hymenoptera	Chalcidoidea	Eulophinae	Eulophinae	sp.	KR885988.1
Hymenoptera	Chalcidoidea	Eulophinae	Eulophinae	sp.	KR886779.1
Hymenoptera	Chalcidoidea	Eulophinae	Eulophinae	sp.	KR890673.1
Hymenoptera	Chalcidoidea	Eulophinae	Eulophinae	sp.	KT706259.1
Hymenoptera	Chalcidoidea	Eulophinae	Eulophinae	sp.	MZ629213.1
Hymenoptera	Chalcidoidea	Eulophinae	Eulophinae	sp.	MZ629543.1

Hymenoptera	Chalcidoidea	Eulophinae	Eulophinae	sp.	MZ630430.1
Hymenoptera	Chalcidoidea	Eulophinae	Eulophinae	sp.	MZ630619.1
Hymenoptera	Chalcidoidea	Eulophinae	Eulophinae	sp.	MZ631330.1
Hymenoptera	Chalcidoidea	Eulophinae	Eulophinae	sp.	MZ633329.1
Hymenoptera	Chalcidoidea	Eupelmidae	Anastatus	acherontiae	KU052673.1
Hymenoptera	Chalcidoidea	Eupelmidae	Anastatus	sp.	KR348752.1
Hymenoptera	Chalcidoidea	Eupelmidae	Anastatus	bangalorensis	KU052674.1
Hymenoptera	Chalcidoidea	Eupelmidae	Anastatus	fulloi	MK604241.1
Hymenoptera	Chalcidoidea	Eupelmidae	Anastatus	gansuensis	MK373759.1
Hymenoptera	Chalcidoidea	Eupelmidae	Anastatus	japonicus	MK604240.1
Hymenoptera	Chalcidoidea	Eupelmidae	Anastatus	meilingensis	MK604242.1
Hymenoptera	Chalcidoidea	Eupelmidae	Anastatus	orientalis	MN746719.1
Hymenoptera	Chalcidoidea	Eupelmidae	Anastatus	orientalis	MN746721.1
Hymenoptera	Chalcidoidea	Eupelmidae	Anastatus	orientalis	MN746724.1
Hymenoptera	Chalcidoidea	Eupelmidae	Anastatus	orientalis	MN746725.1
Hymenoptera	Chalcidoidea	Eupelmidae	Anastatus	orientalis	MN746727.1
Hymenoptera	Chalcidoidea	Eupelmidae	Anastatus	orientalis	MN746728.1
Hymenoptera	Chalcidoidea	Eupelmidae	Anastatus	orientalis	MN746729.1
Hymenoptera	Chalcidoidea	Eupelmidae	Anastatus	orientalis	MN746730.1
Hymenoptera	Chalcidoidea	Eupelmidae	Anastatus	orientalis	MN746732.1
Hymenoptera	Chalcidoidea	Eupelmidae	Anastatus	orientalis	MN746733.1
Hymenoptera	Chalcidoidea	Eupelmidae	Anastatus	orientalis	MN746735.1
Hymenoptera	Chalcidoidea	Eupelmidae	Anastatus	orientalis	MN746736.1
Hymenoptera	Chalcidoidea	Eupelmidae	Anastatus	orientalis	MN746737.1
Hymenoptera	Chalcidoidea	Eupelmidae	Anastatus	orientalis	MN746738.1
Hymenoptera	Chalcidoidea	Eupelmidae	Anastatus	orientalis	MN746739.1
Hymenoptera	Chalcidoidea	Eupelmidae	Anastatus	orientalis	MN746740.1
Hymenoptera	Chalcidoidea	Eupelmidae	Anastatus	orientalis	MN746742.1
Hymenoptera	Chalcidoidea	Eupelmidae	Anastatus	orientalis	MN746748.1
Hymenoptera	Chalcidoidea	Eupelmidae	Anastatus	orientalis	MN746749.1
Hymenoptera	Chalcidoidea	Eupelmidae	Anastatus	orientalis	MN746750.1
Hymenoptera	Chalcidoidea	Eupelmidae	Anastatus	orientalis	MN746752.1
Hymenoptera	Chalcidoidea	Eupelmidae	Anastatus	orientalis	MN746756.1
Hymenoptera	Chalcidoidea	Eupelmidae	Anastatus	orientalis	MN746757.1
Hymenoptera	Chalcidoidea	Eupelmidae	Anastatus	orientalis	MN746758.1
Hymenoptera	Chalcidoidea	Eupelmidae	Anastatus	orientalis	MN746761.1
Hymenoptera	Chalcidoidea	Eupelmidae	Anastatus	orientalis	MN746762.1
Hymenoptera	Chalcidoidea	Eupelmidae	Anastatus	sidereus	KR348751.1
Hymenoptera	Chalcidoidea	Eupelmidae	Anastatus	sp.	KR885523.1
Hymenoptera	Chalcidoidea	Eupelmidae	Anastatus	sp.	MG344499.1
Hymenoptera	Chalcidoidea	Eupelmidae	Anastatus	sp.	MN135771.1
Hymenoptera	Chalcidoidea	Eupelmidae	Arachnophaga	eucnemia	KR802702.1
Hymenoptera	Chalcidoidea	Eupelmidae	Brasema	macrocarpae	HQ930307.1
Hymenoptera	Chalcidoidea	Eupelmidae	Brasema	rhadinosa	HM423347.1
Hymenoptera	Chalcidoidea	Eupelmidae	Brasema	sp.	KR108721.1
Hymenoptera	Chalcidoidea	Eupelmidae	Brasema	sp.	KR108722.1
Hymenoptera	Chalcidoidea	Eupelmidae	Brasema	sp.	KR108723.1
Hymenoptera	Chalcidoidea	Eupelmidae	Brasema	sp.	KR108734.1
Hymenoptera	Chalcidoidea	Eupelmidae	Brasema	sp.	KR996139.1
Hymenoptera	Chalcidoidea	Eupelmidae	Eopelma	gibsoni	KY609221.1
Hymenoptera	Chalcidoidea	Eupelmidae	Eupelmidae	sp.	KJ208871.1
Hymenoptera	Chalcidoidea	Eupelmidae	Eupelmidae	sp.	KR791535.1
Hymenoptera	Chalcidoidea	Eupelmidae	Eupelmidae	sp.	KR806485.1
Hymenoptera	Chalcidoidea	Eupelmidae	Eupelmidae	sp.	KR807455.1

Hymenoptera	Chalcidoidea	Eupelmidae	Eupelmidae	sp.	KR876138.1
Hymenoptera	Chalcidoidea	Eupelmidae	Eupelmidae	sp.	KR881304.1
Hymenoptera	Chalcidoidea	Eupelmidae	Eupelmidae	sp.	KR898027.1
Hymenoptera	Chalcidoidea	Eupelmidae	Eupelmidae	sp.	KY831305.1
Hymenoptera	Chalcidoidea	Eupelmidae	Eupelmidae	sp.	KY835626.1
Hymenoptera	Chalcidoidea	Eupelmidae	Eupelmidae	sp.	KY835966.1
Hymenoptera	Chalcidoidea	Eupelmidae	Eupelmidae	sp.	KY840450.1
Hymenoptera	Chalcidoidea	Eupelmidae	Eupelmus	acinellus	KJ018383.1
Hymenoptera	Chalcidoidea	Eupelmidae	Eupelmus	acinellus	KJ018384.1
Hymenoptera	Chalcidoidea	Eupelmidae	Eupelmus	atropurpureus	KR348771.1
Hymenoptera	Chalcidoidea	Eupelmidae	Eupelmus	aulatus	KJ018333.1
Hymenoptera	Chalcidoidea	Eupelmidae	Eupelmus	aulatus	KJ018363.1
Hymenoptera	Chalcidoidea	Eupelmidae	Eupelmus	aulatus	KJ018379.1
Hymenoptera	Chalcidoidea	Eupelmidae	Eupelmus	aulatus	KJ018395.1
Hymenoptera	Chalcidoidea	Eupelmidae	Eupelmus	aulatus	KJ018401.1
Hymenoptera	Chalcidoidea	Eupelmidae	Eupelmus	aulatus	KJ018403.1
Hymenoptera	Chalcidoidea	Eupelmidae	Eupelmus	aulatus	KJ018438.1
Hymenoptera	Chalcidoidea	Eupelmidae	Eupelmus	aulatus	KJ018439.1
Hymenoptera	Chalcidoidea	Eupelmidae	Eupelmus	aulatus	KJ018471.1
Hymenoptera	Chalcidoidea	Eupelmidae	Eupelmus	aulatus	MG343741.1
Hymenoptera	Chalcidoidea	Eupelmidae	Eupelmus	azureus	KJ018331.1
Hymenoptera	Chalcidoidea	Eupelmidae	Eupelmus	azureus	KJ018372.1
Hymenoptera	Chalcidoidea	Eupelmidae	Eupelmus	azureus	KJ018378.1
Hymenoptera	Chalcidoidea	Eupelmidae	Eupelmus	azureus	KJ018399.1
Hymenoptera	Chalcidoidea	Eupelmidae	Eupelmus	azureus	KJ018404.1
Hymenoptera	Chalcidoidea	Eupelmidae	Eupelmus	azureus	KJ018448.1
Hymenoptera	Chalcidoidea	Eupelmidae	Eupelmus	azureus	KJ018449.1
Hymenoptera	Chalcidoidea	Eupelmidae	Eupelmus	azureus	KR348755.1
Hymenoptera	Chalcidoidea	Eupelmidae	Eupelmus	azureus	KR348757.1
Hymenoptera	Chalcidoidea	Eupelmidae	Eupelmus	azureus	KR348769.1
Hymenoptera	Chalcidoidea	Eupelmidae	Eupelmus	cerris	KJ018335.1
Hymenoptera	Chalcidoidea	Eupelmidae	Eupelmus	confusus	KJ018343.1
Hymenoptera	Chalcidoidea	Eupelmidae	Eupelmus	confusus	KJ018345.1
Hymenoptera	Chalcidoidea	Eupelmidae	Eupelmus	confusus	KJ018347.1
Hymenoptera	Chalcidoidea	Eupelmidae	Eupelmus	confusus	KJ018348.1
Hymenoptera	Chalcidoidea	Eupelmidae	Eupelmus	confusus	KJ018351.1
Hymenoptera	Chalcidoidea	Eupelmidae	Eupelmus	confusus	KJ018352.1
Hymenoptera	Chalcidoidea	Eupelmidae	Eupelmus	confusus	KJ018356.1
Hymenoptera	Chalcidoidea	Eupelmidae	Eupelmus	confusus	KJ018360.1
Hymenoptera	Chalcidoidea	Eupelmidae	Eupelmus	confusus	KJ018361.1
Hymenoptera	Chalcidoidea	Eupelmidae	Eupelmus	confusus	KJ018367.1
Hymenoptera	Chalcidoidea	Eupelmidae	Eupelmus	confusus	KJ018368.1
Hymenoptera	Chalcidoidea	Eupelmidae	Eupelmus	confusus	KJ018369.1
Hymenoptera	Chalcidoidea	Eupelmidae	Eupelmus	confusus	KJ018370.1
Hymenoptera	Chalcidoidea	Eupelmidae	Eupelmus	confusus	KJ018376.1
Hymenoptera	Chalcidoidea	Eupelmidae	Eupelmus	confusus	KJ018377.1
Hymenoptera	Chalcidoidea	Eupelmidae	Eupelmus	confusus	KJ018391.1
Hymenoptera	Chalcidoidea	Eupelmidae	Eupelmus	confusus	KJ018392.1
Hymenoptera	Chalcidoidea	Eupelmidae	Eupelmus	confusus	KJ018408.1
Hymenoptera	Chalcidoidea	Eupelmidae	Eupelmus	confusus	KJ018409.1
Hymenoptera	Chalcidoidea	Eupelmidae	Eupelmus	confusus	KJ018412.1
Hymenoptera	Chalcidoidea	Eupelmidae	Eupelmus	confusus	KJ018413.1
Hymenoptera	Chalcidoidea	Eupelmidae	Eupelmus	confusus	KJ018415.1
Hymenoptera	Chalcidoidea	Eupelmidae	Eupelmus	confusus	KJ018416.1

Hymenoptera	Chalcidoidea	Eupelmidae	Eupelmus	confusus	KJ018417.1
Hymenoptera	Chalcidoidea	Eupelmidae	Eupelmus	confusus	KJ018419.1
Hymenoptera	Chalcidoidea	Eupelmidae	Eupelmus	confusus	KJ018424.1
Hymenoptera	Chalcidoidea	Eupelmidae	Eupelmus	confusus	KJ018426.1
Hymenoptera	Chalcidoidea	Eupelmidae	Eupelmus	confusus	KJ018428.1
Hymenoptera	Chalcidoidea	Eupelmidae	Eupelmus	confusus	KJ018429.1
Hymenoptera	Chalcidoidea	Eupelmidae	Eupelmus	confusus	KJ018430.1
Hymenoptera	Chalcidoidea	Eupelmidae	Eupelmus	confusus	KJ018432.1
Hymenoptera	Chalcidoidea	Eupelmidae	Eupelmus	confusus	KJ018452.1
Hymenoptera	Chalcidoidea	Eupelmidae	Eupelmus	confusus	KJ018469.1
Hymenoptera	Chalcidoidea	Eupelmidae	Eupelmus	confusus	KJ018473.1
Hymenoptera	Chalcidoidea	Eupelmidae	Eupelmus	confusus	KJ018474.1
Hymenoptera	Chalcidoidea	Eupelmidae	Eupelmus	confusus	KR348758.1
Hymenoptera	Chalcidoidea	Eupelmidae	Eupelmus	cushmani	KF444812.1
Hymenoptera	Chalcidoidea	Eupelmidae	Eupelmus	falcatus	KR348749.1
Hymenoptera	Chalcidoidea	Eupelmidae	Eupelmus	fulvipes	KJ018364.1
Hymenoptera	Chalcidoidea	Eupelmidae	Eupelmus	fulvipes	KJ018464.1
Hymenoptera	Chalcidoidea	Eupelmidae	Eupelmus	fulvipes	KJ018466.1
Hymenoptera	Chalcidoidea	Eupelmidae	Eupelmus	gemellus	KJ018338.1
Hymenoptera	Chalcidoidea	Eupelmidae	Eupelmus	gemellus	KJ018344.1
Hymenoptera	Chalcidoidea	Eupelmidae	Eupelmus	gemellus	KJ018349.1
Hymenoptera	Chalcidoidea	Eupelmidae	Eupelmus	gemellus	KJ018381.1
Hymenoptera	Chalcidoidea	Eupelmidae	Eupelmus	gemellus	KJ018382.1
Hymenoptera	Chalcidoidea	Eupelmidae	Eupelmus	gemellus	KJ018405.1
Hymenoptera	Chalcidoidea	Eupelmidae	Eupelmus	gemellus	KJ018407.1
Hymenoptera	Chalcidoidea	Eupelmidae	Eupelmus	gemellus	KJ018410.1
Hymenoptera	Chalcidoidea	Eupelmidae	Eupelmus	gemellus	KJ018421.1
Hymenoptera	Chalcidoidea	Eupelmidae	Eupelmus	gemellus	KJ018422.1
Hymenoptera	Chalcidoidea	Eupelmidae	Eupelmus	gemellus	KJ018425.1
Hymenoptera	Chalcidoidea	Eupelmidae	Eupelmus	gemellus	KJ018427.1
Hymenoptera	Chalcidoidea	Eupelmidae	Eupelmus	gemellus	KJ018441.1
Hymenoptera	Chalcidoidea	Eupelmidae	Eupelmus	gemellus	KJ018442.1
Hymenoptera	Chalcidoidea	Eupelmidae	Eupelmus	gemellus	KJ018470.1
Hymenoptera	Chalcidoidea	Eupelmidae	Eupelmus	gemellus	KR348765.1
Hymenoptera	Chalcidoidea	Eupelmidae	Eupelmus	janstai	KJ018330.1
Hymenoptera	Chalcidoidea	Eupelmidae	Eupelmus	juniperinus	KR348746.1
Hymenoptera	Chalcidoidea	Eupelmidae	Eupelmus	kiefferi	KJ018328.1
Hymenoptera	Chalcidoidea	Eupelmidae	Eupelmus	kiefferi	KJ018329.1
Hymenoptera	Chalcidoidea	Eupelmidae	Eupelmus	kiefferi	KJ018337.1
Hymenoptera	Chalcidoidea	Eupelmidae	Eupelmus	kiefferi	KJ018340.1
Hymenoptera	Chalcidoidea	Eupelmidae	Eupelmus	kiefferi	KJ018342.1
Hymenoptera	Chalcidoidea	Eupelmidae	Eupelmus	kiefferi	KJ018350.1
Hymenoptera	Chalcidoidea	Eupelmidae	Eupelmus	kiefferi	KJ018354.1
Hymenoptera	Chalcidoidea	Eupelmidae	Eupelmus	kiefferi	KJ018355.1
Hymenoptera	Chalcidoidea	Eupelmidae	Eupelmus	kiefferi	KJ018362.1
Hymenoptera	Chalcidoidea	Eupelmidae	Eupelmus	kiefferi	KJ018365.1
Hymenoptera	Chalcidoidea	Eupelmidae	Eupelmus	kiefferi	KJ018366.1
Hymenoptera	Chalcidoidea	Eupelmidae	Eupelmus	kiefferi	KJ018393.1
Hymenoptera	Chalcidoidea	Eupelmidae	Eupelmus	kiefferi	KJ018397.1
Hymenoptera	Chalcidoidea	Eupelmidae	Eupelmus	kiefferi	KJ018400.1
Hymenoptera	Chalcidoidea	Eupelmidae	Eupelmus	kiefferi	KJ018406.1
Hymenoptera	Chalcidoidea	Eupelmidae	Eupelmus	kiefferi	KJ018414.1
Hymenoptera	Chalcidoidea	Eupelmidae	Eupelmus	kiefferi	KJ018436.1
Hymenoptera	Chalcidoidea	Eupelmidae	Eupelmus	kiefferi	KJ018437.1

Hymenoptera	Chalcidoidea	Eupelmidae	Eupelmus	kiefferi	KJ018450.1
Hymenoptera	Chalcidoidea	Eupelmidae	Eupelmus	kiefferi	KJ018451.1
Hymenoptera	Chalcidoidea	Eupelmidae	Eupelmus	kiefferi	KJ018467.1
Hymenoptera	Chalcidoidea	Eupelmidae	Eupelmus	kiefferi	KJ018476.1
Hymenoptera	Chalcidoidea	Eupelmidae	Eupelmus	kiefferi	KR348753.1
Hymenoptera	Chalcidoidea	Eupelmidae	Eupelmus	kiefferi	KR348770.1
Hymenoptera	Chalcidoidea	Eupelmidae	Eupelmus	linearis	KJ018334.1
Hymenoptera	Chalcidoidea	Eupelmidae	Eupelmus	linearis	KR348747.1
Hymenoptera	Chalcidoidea	Eupelmidae	Eupelmus	longicalvus	KJ018327.1
Hymenoptera	Chalcidoidea	Eupelmidae	Eupelmus	longicalvus	KJ018418.1
Hymenoptera	Chalcidoidea	Eupelmidae	Eupelmus	longicalvus	KJ018455.1
Hymenoptera	Chalcidoidea	Eupelmidae	Eupelmus	longicalvus	KJ018456.1
Hymenoptera	Chalcidoidea	Eupelmidae	Eupelmus	longicalvus	KJ018457.1
Hymenoptera	Chalcidoidea	Eupelmidae	Eupelmus	longicalvus	KJ018458.1
Hymenoptera	Chalcidoidea	Eupelmidae	Eupelmus	longicalvus	KJ018459.1
Hymenoptera	Chalcidoidea	Eupelmidae	Eupelmus	martellii	KJ018468.1
Hymenoptera	Chalcidoidea	Eupelmidae	Eupelmus	matranus	KR348759.1
Hymenoptera	Chalcidoidea	Eupelmidae	Eupelmus	memnonius	KT352073.1
Hymenoptera	Chalcidoidea	Eupelmidae	Eupelmus	microzonus	KR348754.1
Hymenoptera	Chalcidoidea	Eupelmidae	Eupelmus	minozonus	KJ018323.1
Hymenoptera	Chalcidoidea	Eupelmidae	Eupelmus	minozonus	KJ018324.1
Hymenoptera	Chalcidoidea	Eupelmidae	Eupelmus	minozonus	KJ018325.1
Hymenoptera	Chalcidoidea	Eupelmidae	Eupelmus	minozonus	KJ018336.1
Hymenoptera	Chalcidoidea	Eupelmidae	Eupelmus	minozonus	KJ018472.1
Hymenoptera	Chalcidoidea	Eupelmidae	Eupelmus	opacus	KJ018434.1
Hymenoptera	Chalcidoidea	Eupelmidae	Eupelmus	opacus	KJ018435.1
Hymenoptera	Chalcidoidea	Eupelmidae	Eupelmus	pini	KR348745.1
Hymenoptera	Chalcidoidea	Eupelmidae	Eupelmus	pini	KT352074.1
Hymenoptera	Chalcidoidea	Eupelmidae	Eupelmus	pistaciae	KJ018321.1
Hymenoptera	Chalcidoidea	Eupelmidae	Eupelmus	pistaciae	KJ018322.1
Hymenoptera	Chalcidoidea	Eupelmidae	Eupelmus	pistaciae	KJ018444.1
Hymenoptera	Chalcidoidea	Eupelmidae	Eupelmus	priotoni	KJ018332.1
Hymenoptera	Chalcidoidea	Eupelmidae	Eupelmus	purpuricollis	KJ018460.1
Hymenoptera	Chalcidoidea	Eupelmidae	Eupelmus	purpuricollis	KJ018461.1
Hymenoptera	Chalcidoidea	Eupelmidae	Eupelmus	seculatus	KR348750.1
Hymenoptera	Chalcidoidea	Eupelmidae	Eupelmus	simizonus	KJ018388.1
Hymenoptera	Chalcidoidea	Eupelmidae	Eupelmus	sp.	KR410181.1
Hymenoptera	Chalcidoidea	Eupelmidae	Eupelmus	sp.	MZ632083.1
Hymenoptera	Chalcidoidea	Eupelmidae	Eupelmus	spermophilus	MH841921.1
Hymenoptera	Chalcidoidea	Eupelmidae	Eupelmus	spermophilus	MH841922.1
Hymenoptera	Chalcidoidea	Eupelmidae	Eupelmus	spermophilus	MH841926.1
Hymenoptera	Chalcidoidea	Eupelmidae	Eupelmus	spermophilus	MH841927.1
Hymenoptera	Chalcidoidea	Eupelmidae	Eupelmus	spermophilus	MH841928.1
Hymenoptera	Chalcidoidea	Eupelmidae	Eupelmus	spermophilus	MH841929.1
Hymenoptera	Chalcidoidea	Eupelmidae	Eupelmus	testaceiventris	KR348748.1
Hymenoptera	Chalcidoidea	Eupelmidae	Eupelmus	tibicinis	KJ018389.1
Hymenoptera	Chalcidoidea	Eupelmidae	Eupelmus	tibicinis	KJ018390.1
Hymenoptera	Chalcidoidea	Eupelmidae	Eupelmus	tibicinis	KJ018453.1
Hymenoptera	Chalcidoidea	Eupelmidae	Eupelmus	tibicinis	KJ018454.1
Hymenoptera	Chalcidoidea	Eupelmidae	Eupelmus	tremulae	KJ018446.1
Hymenoptera	Chalcidoidea	Eupelmidae	Eupelmus	urozonus	KJ018320.1
Hymenoptera	Chalcidoidea	Eupelmidae	Eupelmus	urozonus	KJ018326.1
Hymenoptera	Chalcidoidea	Eupelmidae	Eupelmus	urozonus	KJ018339.1
Hymenoptera	Chalcidoidea	Eupelmidae	Eupelmus	urozonus	KJ018346.1

Hymenoptera	Chalcidoidea	Eupelmidae	Eupelmus	urozonus	KJ018353.1
Hymenoptera	Chalcidoidea	Eupelmidae	Eupelmus	urozonus	KJ018357.1
Hymenoptera	Chalcidoidea	Eupelmidae	Eupelmus	urozonus	KJ018373.1
Hymenoptera	Chalcidoidea	Eupelmidae	Eupelmus	urozonus	KJ018385.1
Hymenoptera	Chalcidoidea	Eupelmidae	Eupelmus	urozonus	KJ018387.1
Hymenoptera	Chalcidoidea	Eupelmidae	Eupelmus	urozonus	KJ018394.1
Hymenoptera	Chalcidoidea	Eupelmidae	Eupelmus	urozonus	KJ018396.1
Hymenoptera	Chalcidoidea	Eupelmidae	Eupelmus	urozonus	KJ018398.1
Hymenoptera	Chalcidoidea	Eupelmidae	Eupelmus	urozonus	KJ018402.1
Hymenoptera	Chalcidoidea	Eupelmidae	Eupelmus	urozonus	KJ018420.1
Hymenoptera	Chalcidoidea	Eupelmidae	Eupelmus	urozonus	KJ018423.1
Hymenoptera	Chalcidoidea	Eupelmidae	Eupelmus	urozonus	KJ018431.1
Hymenoptera	Chalcidoidea	Eupelmidae	Eupelmus	urozonus	KJ018433.1
Hymenoptera	Chalcidoidea	Eupelmidae	Eupelmus	urozonus	KJ018443.1
Hymenoptera	Chalcidoidea	Eupelmidae	Eupelmus	urozonus	KJ018445.1
Hymenoptera	Chalcidoidea	Eupelmidae	Eupelmus	urozonus	KJ018447.1
Hymenoptera	Chalcidoidea	Eupelmidae	Eupelmus	urozonus	KJ018463.1
Hymenoptera	Chalcidoidea	Eupelmidae	Eupelmus	urozonus	KJ018475.1
Hymenoptera	Chalcidoidea	Eupelmidae	Eupelmus	urozonus	KR348756.1
Hymenoptera	Chalcidoidea	Eupelmidae	Eupelmus	urozonus	KR348760.1
Hymenoptera	Chalcidoidea	Eupelmidae	Eupelmus	urozonus	KR348763.1
Hymenoptera	Chalcidoidea	Eupelmidae	Eupelmus	urozonus	KR348766.1
Hymenoptera	Chalcidoidea	Eupelmidae	Eupelmus	urozonus	MN531304.1
Hymenoptera	Chalcidoidea	Eupelmidae	Eupelmus	urozonus	MZ631451.1
Hymenoptera	Chalcidoidea	Eupelmidae	Eupelmus	utahensis	MF902739.1
Hymenoptera	Chalcidoidea	Eupelmidae	Eupelmus	vesicularis	KR788857.1
Hymenoptera	Chalcidoidea	Eupelmidae	Eupelmus	vesicularis	KR791575.1
Hymenoptera	Chalcidoidea	Eupelmidae	Eupelmus	vesicularis	KR924137.1
Hymenoptera	Chalcidoidea	Eupelmidae	Eupelmus	vesicularis	KR925456.1
Hymenoptera	Chalcidoidea	Eupelmidae	Eupelmus	vesicularis	MF901588.1
Hymenoptera	Chalcidoidea	Eupelmidae	Eupelmus	vesicularis	MG342865.1
Hymenoptera	Chalcidoidea	Eupelmidae	Eupelmus	vindex	KR348744.1
Hymenoptera	Chalcidoidea	Eupelmidae	Eupelmus	vindex	KR348767.1
Hymenoptera	Chalcidoidea	Eupelmidae	Eupelmus	vindex	KR348768.1
Hymenoptera	Chalcidoidea	Eupelmidae	Merostenus	sp.	HQ930271.1
Hymenoptera	Chalcidoidea	Eupelmidae	Reikosiella	sp.	KR348761.1
Hymenoptera	Chalcidoidea	Eupelmidae	Reikosiella	sp.	KR348762.1
Hymenoptera	Chalcidoidea	Eupelmidae	Tineobius	tamaricis	KT962861.1
Hymenoptera	Chalcidoidea	Eurytomidae	Aximopsis	collina	MH878923.1
Hymenoptera	Chalcidoidea	Eurytomidae	Aximopsis	sp.	MH878911.1
Hymenoptera	Chalcidoidea	Eurytomidae	Aximopsis	sp.	MH878912.1
Hymenoptera	Chalcidoidea	Eurytomidae	Aximopsis	sp.	MH878913.1
Hymenoptera	Chalcidoidea	Eurytomidae	Aximopsis	sp.	MH878914.1
Hymenoptera	Chalcidoidea	Eurytomidae	Aximopsis	sp.	MH878919.1
Hymenoptera	Chalcidoidea	Eurytomidae	Aximopsis	sp.	MH878925.1
Hymenoptera	Chalcidoidea	Eurytomidae	Aximopsis	sp.	MK041065.1
Hymenoptera	Chalcidoidea	Eurytomidae	Bruchophagus	caucasicus	JQ756607.1
Hymenoptera	Chalcidoidea	Eurytomidae	Bruchophagus	gibbus	MH878927.1
Hymenoptera	Chalcidoidea	Eurytomidae	Bruchophagus	sp.	MH878926.1
Hymenoptera	Chalcidoidea	Eurytomidae	Bruchophagus	sp.	MH878928.1
Hymenoptera	Chalcidoidea	Eurytomidae	Bruchophagus	sp.	MH878929.1
Hymenoptera	Chalcidoidea	Eurytomidae	Bruchophagus	sp.	MH878930.1
Hymenoptera	Chalcidoidea	Eurytomidae	Bruchophagus	sp.	MH878931.1
Hymenoptera	Chalcidoidea	Eurytomidae	Bruchophagus	sp.	MH878932.1

Hymenoptera	Chalcidoidea	Eurytomidae	Bruchophagus	sp.	MH878933.1
Hymenoptera	Chalcidoidea	Eurytomidae	Bruchophagus	sp.	MH878934.1
Hymenoptera	Chalcidoidea	Eurytomidae	Bruchophagus	sp.	MH878935.1
Hymenoptera	Chalcidoidea	Eurytomidae	Eurytoma	aciculata	KT599266.1
Hymenoptera	Chalcidoidea	Eurytomidae	Eurytoma	sp.	KC685217.1
Hymenoptera	Chalcidoidea	Eurytomidae	Eurytoma	sp.	KC685223.1
Hymenoptera	Chalcidoidea	Eurytomidae	Eurytoma	asphodeli	MH878839.1
Hymenoptera	Chalcidoidea	Eurytomidae	Eurytoma	asphodeli	MH878840.1
Hymenoptera	Chalcidoidea	Eurytomidae	Eurytoma	asphodeli	MH878841.1
Hymenoptera	Chalcidoidea	Eurytomidae	Eurytoma	asphodeli	MH878842.1
Hymenoptera	Chalcidoidea	Eurytomidae	Eurytoma	asphodeli	MH878843.1
Hymenoptera	Chalcidoidea	Eurytomidae	Eurytoma	asphodeli	MH878844.1
Hymenoptera	Chalcidoidea	Eurytomidae	Eurytoma	asphodeli	MH878845.1
Hymenoptera	Chalcidoidea	Eurytomidae	Eurytoma	asphodeli	MH878846.1
Hymenoptera	Chalcidoidea	Eurytomidae	Eurytoma	asphodeli	MH878847.1
Hymenoptera	Chalcidoidea	Eurytomidae	Eurytoma	asphodeli	MH878848.1
Hymenoptera	Chalcidoidea	Eurytomidae	Eurytoma	asphodeli	MH878849.1
Hymenoptera	Chalcidoidea	Eurytomidae	Eurytoma	asphodeli	MH878850.1
Hymenoptera	Chalcidoidea	Eurytomidae	Eurytoma	asphodeli	MH878854.1
Hymenoptera	Chalcidoidea	Eurytomidae	Eurytoma	asphodeli	MH878857.1
Hymenoptera	Chalcidoidea	Eurytomidae	Eurytoma	asphodeli	MH878858.1
Hymenoptera	Chalcidoidea	Eurytomidae	Eurytoma	asphodeli	MH878864.1
Hymenoptera	Chalcidoidea	Eurytomidae	Eurytoma	asphodeli	MH878865.1
Hymenoptera	Chalcidoidea	Eurytomidae	Eurytoma	asphodeli	MH878868.1
Hymenoptera	Chalcidoidea	Eurytomidae	Eurytoma	asphodeli	MH878869.1
Hymenoptera	Chalcidoidea	Eurytomidae	Eurytoma	asphodeli	MH878870.1
Hymenoptera	Chalcidoidea	Eurytomidae	Eurytoma	asphodeli	MH878871.1
Hymenoptera	Chalcidoidea	Eurytomidae	Eurytoma	asphodeli	MH878873.1
Hymenoptera	Chalcidoidea	Eurytomidae	Eurytoma	asphodeli	MH878874.1
Hymenoptera	Chalcidoidea	Eurytomidae	Eurytoma	asphodeli	MH878875.1
Hymenoptera	Chalcidoidea	Eurytomidae	Eurytoma	asphodeli	MH878877.1
Hymenoptera	Chalcidoidea	Eurytomidae	Eurytoma	asphodeli	MH878878.1
Hymenoptera	Chalcidoidea	Eurytomidae	Eurytoma	asphodeli	MH878879.1
Hymenoptera	Chalcidoidea	Eurytomidae	Eurytoma	asphodeli	MH878880.1
Hymenoptera	Chalcidoidea	Eurytomidae	Eurytoma	asphodeli	MH878882.1
Hymenoptera	Chalcidoidea	Eurytomidae	Eurytoma	asphodeli	MH878884.1
Hymenoptera	Chalcidoidea	Eurytomidae	Eurytoma	asphodeli	MH878885.1
Hymenoptera	Chalcidoidea	Eurytomidae	Eurytoma	asphodeli	MH878886.1
Hymenoptera	Chalcidoidea	Eurytomidae	Eurytoma	asphodeli	MH878888.1
Hymenoptera	Chalcidoidea	Eurytomidae	Eurytoma	asphodeli	MH878891.1
Hymenoptera	Chalcidoidea	Eurytomidae	Eurytoma	asphodeli	MH878892.1
Hymenoptera	Chalcidoidea	Eurytomidae	Eurytoma	asphodeli	MH878893.1
Hymenoptera	Chalcidoidea	Eurytomidae	Eurytoma	asphodeli	MH878896.1
Hymenoptera	Chalcidoidea	Eurytomidae	Eurytoma	asphodeli	MH878897.1
Hymenoptera	Chalcidoidea	Eurytomidae	Eurytoma	asphodeli	MH878898.1
Hymenoptera	Chalcidoidea	Eurytomidae	Eurytoma	asphodeli	MH878899.1
Hymenoptera	Chalcidoidea	Eurytomidae	Eurytoma	asphodeli	MH878907.1
Hymenoptera	Chalcidoidea	Eurytomidae	Eurytoma	asphodeli	MH878908.1
Hymenoptera	Chalcidoidea	Eurytomidae	Eurytoma	asphodeli	MH878910.1
Hymenoptera	Chalcidoidea	Eurytomidae	Eurytoma	bruiventris	MH878936.1
Hymenoptera	Chalcidoidea	Eurytomidae	Eurytoma	discordans	KC685118.1
Hymenoptera	Chalcidoidea	Eurytomidae	Eurytoma	juniperina	KX885559.1
Hymenoptera	Chalcidoidea	Eurytomidae	Eurytoma	longavena	KC685185.1
Hymenoptera	Chalcidoidea	Eurytomidae	Eurytoma	longavena	KR786474.1

Hymenoptera	Chalcidoidea	Eurytomidae	Eurytoma	longavena	KR879583.1
Hymenoptera	Chalcidoidea	Eurytomidae	Eurytoma	longavena	KR887064.1
Hymenoptera	Chalcidoidea	Eurytomidae	Eurytoma	oleae	MH841910.1
Hymenoptera	Chalcidoidea	Eurytomidae	Eurytoma	oleae	MH841918.1
Hymenoptera	Chalcidoidea	Eurytomidae	Eurytoma	robusta	MH878937.1
Hymenoptera	Chalcidoidea	Eurytomidae	Eurytoma	sp.	JN292443.1
Hymenoptera	Chalcidoidea	Eurytomidae	Eurytoma	sp.	JN292460.1
Hymenoptera	Chalcidoidea	Eurytomidae	Eurytoma	sp.	JN292461.1
Hymenoptera	Chalcidoidea	Eurytomidae	Eurytoma	sp.	JN293254.1
Hymenoptera	Chalcidoidea	Eurytomidae	Eurytoma	sp.	KF444815.1
Hymenoptera	Chalcidoidea	Eurytomidae	Eurytoma	sp.	KR407554.1
Hymenoptera	Chalcidoidea	Eurytomidae	Eurytoma	sp.	KR409624.1
Hymenoptera	Chalcidoidea	Eurytomidae	Eurytoma	sp.	KR415439.1
Hymenoptera	Chalcidoidea	Eurytomidae	Eurytoma	sp.	KR782647.1
Hymenoptera	Chalcidoidea	Eurytomidae	Eurytoma	sp.	KR782923.1
Hymenoptera	Chalcidoidea	Eurytomidae	Eurytoma	sp.	KR783864.1
Hymenoptera	Chalcidoidea	Eurytomidae	Eurytoma	sp.	KR783924.1
Hymenoptera	Chalcidoidea	Eurytomidae	Eurytoma	sp.	KR784183.1
Hymenoptera	Chalcidoidea	Eurytomidae	Eurytoma	sp.	KR784609.1
Hymenoptera	Chalcidoidea	Eurytomidae	Eurytoma	sp.	KR787497.1
Hymenoptera	Chalcidoidea	Eurytomidae	Eurytoma	sp.	KR787512.1
Hymenoptera	Chalcidoidea	Eurytomidae	Eurytoma	sp.	KR788693.1
Hymenoptera	Chalcidoidea	Eurytomidae	Eurytoma	sp.	KR788779.1
Hymenoptera	Chalcidoidea	Eurytomidae	Eurytoma	sp.	KR789872.1
Hymenoptera	Chalcidoidea	Eurytomidae	Eurytoma	sp.	KR792322.1
Hymenoptera	Chalcidoidea	Eurytomidae	Eurytoma	sp.	KR794034.1
Hymenoptera	Chalcidoidea	Eurytomidae	Eurytoma	sp.	KR794508.1
Hymenoptera	Chalcidoidea	Eurytomidae	Eurytoma	sp.	KR794666.1
Hymenoptera	Chalcidoidea	Eurytomidae	Eurytoma	sp.	KR795623.1
Hymenoptera	Chalcidoidea	Eurytomidae	Eurytoma	sp.	KR795711.1
Hymenoptera	Chalcidoidea	Eurytomidae	Eurytoma	sp.	KR795789.1
Hymenoptera	Chalcidoidea	Eurytomidae	Eurytoma	sp.	KR796686.1
Hymenoptera	Chalcidoidea	Eurytomidae	Eurytoma	sp.	KR797521.1
Hymenoptera	Chalcidoidea	Eurytomidae	Eurytoma	sp.	KR800105.1
Hymenoptera	Chalcidoidea	Eurytomidae	Eurytoma	sp.	KR800367.1
Hymenoptera	Chalcidoidea	Eurytomidae	Eurytoma	sp.	KR801425.1
Hymenoptera	Chalcidoidea	Eurytomidae	Eurytoma	sp.	KR802204.1
Hymenoptera	Chalcidoidea	Eurytomidae	Eurytoma	sp.	KR802389.1
Hymenoptera	Chalcidoidea	Eurytomidae	Eurytoma	sp.	KR802764.1
Hymenoptera	Chalcidoidea	Eurytomidae	Eurytoma	sp.	KR806262.1
Hymenoptera	Chalcidoidea	Eurytomidae	Eurytoma	sp.	KR807218.1
Hymenoptera	Chalcidoidea	Eurytomidae	Eurytoma	sp.	KR808463.1
Hymenoptera	Chalcidoidea	Eurytomidae	Eurytoma	sp.	KR808709.1
Hymenoptera	Chalcidoidea	Eurytomidae	Eurytoma	sp.	KR875618.1
Hymenoptera	Chalcidoidea	Eurytomidae	Eurytoma	sp.	KR882638.1
Hymenoptera	Chalcidoidea	Eurytomidae	Eurytoma	sp.	KR897184.1
Hymenoptera	Chalcidoidea	Eurytomidae	Eurytoma	sp.	KU496741.1
Hymenoptera	Chalcidoidea	Eurytomidae	Eurytoma	sp.	MF899305.1
Hymenoptera	Chalcidoidea	Eurytomidae	Eurytoma	sp.	MF900154.1
Hymenoptera	Chalcidoidea	Eurytomidae	Eurytoma	sp.	MG334774.1
Hymenoptera	Chalcidoidea	Eurytomidae	Eurytoma	sp.	MG337813.1
Hymenoptera	Chalcidoidea	Eurytomidae	Eurytoma	sp.	MG338813.1
Hymenoptera	Chalcidoidea	Eurytomidae	Eurytoma	sp.	MG340008.1
Hymenoptera	Chalcidoidea	Eurytomidae	Eurytoma	sp.	MG341154.1

Hymenoptera	Chalcidoidea	Eurytomidae	Eurytoma	sp.	MG343643.1
Hymenoptera	Chalcidoidea	Eurytomidae	Eurytoma	sp.	MG344512.1
Hymenoptera	Chalcidoidea	Eurytomidae	Eurytoma	sp.	MG344612.1
Hymenoptera	Chalcidoidea	Eurytomidae	Eurytoma	varicolor	MH841904.1
Hymenoptera	Chalcidoidea	Eurytomidae	Eurytoma	varicolor	MH841905.1
Hymenoptera	Chalcidoidea	Eurytomidae	Eurytoma	varicolor	MH841906.1
Hymenoptera	Chalcidoidea	Eurytomidae	Eurytomidae	sp.	HM414676.1
Hymenoptera	Chalcidoidea	Eurytomidae	Eurytomidae	sp.	HQ929602.1
Hymenoptera	Chalcidoidea	Eurytomidae	Eurytomidae	sp.	HQ930349.1
Hymenoptera	Chalcidoidea	Eurytomidae	Eurytomidae	sp.	KR373034.1
Hymenoptera	Chalcidoidea	Eurytomidae	Eurytomidae	sp.	KR401968.1
Hymenoptera	Chalcidoidea	Eurytomidae	Eurytomidae	sp.	KR402792.1
Hymenoptera	Chalcidoidea	Eurytomidae	Eurytomidae	sp.	KR403791.1
Hymenoptera	Chalcidoidea	Eurytomidae	Eurytomidae	sp.	KR404869.1
Hymenoptera	Chalcidoidea	Eurytomidae	Eurytomidae	sp.	KR405738.1
Hymenoptera	Chalcidoidea	Eurytomidae	Eurytomidae	sp.	KR407994.1
Hymenoptera	Chalcidoidea	Eurytomidae	Eurytomidae	sp.	KR408139.1
Hymenoptera	Chalcidoidea	Eurytomidae	Eurytomidae	sp.	KR411819.1
Hymenoptera	Chalcidoidea	Eurytomidae	Eurytomidae	sp.	KR416357.1
Hymenoptera	Chalcidoidea	Eurytomidae	Eurytomidae	sp.	KR421668.1
Hymenoptera	Chalcidoidea	Eurytomidae	Eurytomidae	sp.	KR783352.1
Hymenoptera	Chalcidoidea	Eurytomidae	Eurytomidae	sp.	KR784912.1
Hymenoptera	Chalcidoidea	Eurytomidae	Eurytomidae	sp.	KR784995.1
Hymenoptera	Chalcidoidea	Eurytomidae	Eurytomidae	sp.	KR786918.1
Hymenoptera	Chalcidoidea	Eurytomidae	Eurytomidae	sp.	KR789861.1
Hymenoptera	Chalcidoidea	Eurytomidae	Eurytomidae	sp.	KR790997.1
Hymenoptera	Chalcidoidea	Eurytomidae	Eurytomidae	sp.	KR792877.1
Hymenoptera	Chalcidoidea	Eurytomidae	Eurytomidae	sp.	KR793222.1
Hymenoptera	Chalcidoidea	Eurytomidae	Eurytomidae	sp.	KR793757.1
Hymenoptera	Chalcidoidea	Eurytomidae	Eurytomidae	sp.	KR795727.1
Hymenoptera	Chalcidoidea	Eurytomidae	Eurytomidae	sp.	KR799061.1
Hymenoptera	Chalcidoidea	Eurytomidae	Eurytomidae	sp.	KR801081.1
Hymenoptera	Chalcidoidea	Eurytomidae	Eurytomidae	sp.	KR801877.1
Hymenoptera	Chalcidoidea	Eurytomidae	Eurytomidae	sp.	KR805129.1
Hymenoptera	Chalcidoidea	Eurytomidae	Eurytomidae	sp.	KR805913.1
Hymenoptera	Chalcidoidea	Eurytomidae	Eurytomidae	sp.	KR874581.1
Hymenoptera	Chalcidoidea	Eurytomidae	Eurytomidae	sp.	KR874968.1
Hymenoptera	Chalcidoidea	Eurytomidae	Eurytomidae	sp.	KR875010.1
Hymenoptera	Chalcidoidea	Eurytomidae	Eurytomidae	sp.	KR879704.1
Hymenoptera	Chalcidoidea	Eurytomidae	Eurytomidae	sp.	KR880653.1
Hymenoptera	Chalcidoidea	Eurytomidae	Eurytomidae	sp.	KR881076.1
Hymenoptera	Chalcidoidea	Eurytomidae	Eurytomidae	sp.	KR881395.1
Hymenoptera	Chalcidoidea	Eurytomidae	Eurytomidae	sp.	KR881860.1
Hymenoptera	Chalcidoidea	Eurytomidae	Eurytomidae	sp.	KR884232.1
Hymenoptera	Chalcidoidea	Eurytomidae	Eurytomidae	sp.	KR885311.1
Hymenoptera	Chalcidoidea	Eurytomidae	Eurytomidae	sp.	KR888886.1
Hymenoptera	Chalcidoidea	Eurytomidae	Eurytomidae	sp.	KR889388.1
Hymenoptera	Chalcidoidea	Eurytomidae	Eurytomidae	sp.	KR893034.1
Hymenoptera	Chalcidoidea	Eurytomidae	Eurytomidae	sp.	KR898550.1
Hymenoptera	Chalcidoidea	Eurytomidae	Eurytomidae	sp.	KR899080.1
Hymenoptera	Chalcidoidea	Eurytomidae	Eurytomidae	sp.	KR899158.1
Hymenoptera	Chalcidoidea	Eurytomidae	Eurytomidae	sp.	KR901088.1
Hymenoptera	Chalcidoidea	Eurytomidae	Eurytomidae	sp.	KR927623.1
Hymenoptera	Chalcidoidea	Eurytomidae	Eurytomidae	sp.	KT619490.1

Hymenoptera	Chalcidoidea	Eurytomidae	Eurytomidae	sp.	KT621376.1
Hymenoptera	Chalcidoidea	Eurytomidae	Eurytomidae	sp.	KT621503.1
Hymenoptera	Chalcidoidea	Eurytomidae	Eurytomidae	sp.	KT622331.1
Hymenoptera	Chalcidoidea	Eurytomidae	Eurytomidae	sp.	KT623023.1
Hymenoptera	Chalcidoidea	Eurytomidae	Eurytomidae	sp.	KY832193.1
Hymenoptera	Chalcidoidea	Eurytomidae	Eurytomidae	sp.	KY832237.1
Hymenoptera	Chalcidoidea	Eurytomidae	Eurytomidae	sp.	KY835099.1
Hymenoptera	Chalcidoidea	Eurytomidae	Eurytomidae	sp.	KY837399.1
Hymenoptera	Chalcidoidea	Eurytomidae	Eurytomidae	sp.	KY838230.1
Hymenoptera	Chalcidoidea	Eurytomidae	Eurytomidae	sp.	KY839692.1
Hymenoptera	Chalcidoidea	Eurytomidae	Eurytomidae	sp.	KY841054.1
Hymenoptera	Chalcidoidea	Eurytomidae	Eurytomidae	sp.	KY845423.1
Hymenoptera	Chalcidoidea	Eurytomidae	Eurytomidae	sp.	MF900144.1
Hymenoptera	Chalcidoidea	Eurytomidae	Eurytomidae	sp.	MF900157.1
Hymenoptera	Chalcidoidea	Eurytomidae	Eurytomidae	sp.	MF901661.1
Hymenoptera	Chalcidoidea	Eurytomidae	Eurytomidae	sp.	MF903080.1
Hymenoptera	Chalcidoidea	Eurytomidae	Eurytomidae	sp.	MF905034.1
Hymenoptera	Chalcidoidea	Eurytomidae	Eurytomidae	sp.	MF905142.1
Hymenoptera	Chalcidoidea	Eurytomidae	Eurytomidae	sp.	MF905552.1
Hymenoptera	Chalcidoidea	Eurytomidae	Eurytomidae	sp.	MF905679.1
Hymenoptera	Chalcidoidea	Eurytomidae	Eurytomidae	sp.	MF906062.1
Hymenoptera	Chalcidoidea	Eurytomidae	Eurytomidae	sp.	MF907751.1
Hymenoptera	Chalcidoidea	Eurytomidae	Eurytomidae	sp.	MG335635.1
Hymenoptera	Chalcidoidea	Eurytomidae	Eurytomidae	sp.	MG336038.1
Hymenoptera	Chalcidoidea	Eurytomidae	Eurytomidae	sp.	MG336119.1
Hymenoptera	Chalcidoidea	Eurytomidae	Eurytomidae	sp.	MG336759.1
Hymenoptera	Chalcidoidea	Eurytomidae	Eurytomidae	sp.	MG337859.1
Hymenoptera	Chalcidoidea	Eurytomidae	Eurytomidae	sp.	MG338872.1
Hymenoptera	Chalcidoidea	Eurytomidae	Eurytomidae	sp.	MG338989.1
Hymenoptera	Chalcidoidea	Eurytomidae	Eurytomidae	sp.	MG339270.1
Hymenoptera	Chalcidoidea	Eurytomidae	Eurytomidae	sp.	MG340495.1
Hymenoptera	Chalcidoidea	Eurytomidae	Eurytomidae	sp.	MG341450.1
Hymenoptera	Chalcidoidea	Eurytomidae	Eurytomidae	sp.	MG341874.1
Hymenoptera	Chalcidoidea	Eurytomidae	Eurytomidae	sp.	MG342287.1
Hymenoptera	Chalcidoidea	Eurytomidae	Eurytomidae	sp.	MG343841.1
Hymenoptera	Chalcidoidea	Eurytomidae	Eurytomidae	sp.	MG344604.1
Hymenoptera	Chalcidoidea	Eurytomidae	Eurytomidae	sp.	MG485454.1
Hymenoptera	Chalcidoidea	Eurytomidae	Eurytominae	sp.	KR787058.1
Hymenoptera	Chalcidoidea	Eurytomidae	Eurytominae	sp.	KR787489.1
Hymenoptera	Chalcidoidea	Eurytomidae	Eurytominae	sp.	KR792109.1
Hymenoptera	Chalcidoidea	Eurytomidae	Eurytominae	sp.	MF904548.1
Hymenoptera	Chalcidoidea	Eurytomidae	Eurytominae	sp.	MF904805.1
Hymenoptera	Chalcidoidea	Eurytomidae	Eurytominae	sp.	MG337522.1
Hymenoptera	Chalcidoidea	Eurytomidae	Ficomila	sp.	GQ367946.1
Hymenoptera	Chalcidoidea	Eurytomidae	Sycophila	aethiopica	MH841894.1
Hymenoptera	Chalcidoidea	Eurytomidae	Sycophila	sp.	FJ499717.1
Hymenoptera	Chalcidoidea	Eurytomidae	Sycophila	sp.	FJ499720.1
Hymenoptera	Chalcidoidea	Eurytomidae	Sycophila	sp.	FJ499738.1
Hymenoptera	Chalcidoidea	Eurytomidae	Sycophila	sp.	FJ499741.1
Hymenoptera	Chalcidoidea	Eurytomidae	Sycophila	sp.	FJ499742.1
Hymenoptera	Chalcidoidea	Eurytomidae	Sycophila	sp.	FJ499748.1
Hymenoptera	Chalcidoidea	Eurytomidae	Sycophila	sp.	FJ499749.1
Hymenoptera	Chalcidoidea	Eurytomidae	Sycophila	sp.	FJ499752.1
Hymenoptera	Chalcidoidea	Eurytomidae	Sycophila	sp.	FJ499753.1

Hymenoptera	Chalcidoidea	Eurytomidae	Sycophila	sp.	FJ499773.1
Hymenoptera	Chalcidoidea	Eurytomidae	Sycophila	sp.	FJ499774.1
Hymenoptera	Chalcidoidea	Eurytomidae	Sycophila	sp.	FJ499775.1
Hymenoptera	Chalcidoidea	Eurytomidae	Sycophila	sp.	FJ499776.1
Hymenoptera	Chalcidoidea	Eurytomidae	Sycophila	sp.	FJ499797.1
Hymenoptera	Chalcidoidea	Eurytomidae	Sycophila	sp.	FJ499798.1
Hymenoptera	Chalcidoidea	Eurytomidae	Sycophila	sp.	FJ499799.1
Hymenoptera	Chalcidoidea	Eurytomidae	Sycophila	sp.	FJ499800.1
Hymenoptera	Chalcidoidea	Eurytomidae	Sycophila	sp.	FJ499801.1
Hymenoptera	Chalcidoidea	Eurytomidae	Sycophila	sp.	FJ499802.1
Hymenoptera	Chalcidoidea	Eurytomidae	Systole	sp.	MW984158.1
Hymenoptera	Chalcidoidea	Eurytomidae	Tetramesa	sp.	KR799792.1
Hymenoptera	Chalcidoidea	Eurytomidae	Tetramesa	sp.	KR800436.1
Hymenoptera	Chalcidoidea	Eurytomidae	Tetramesa	sp.	KU496847.1
Hymenoptera	Chalcidoidea	Eurytomidae	Tetramesa	sp.	MG339505.1
Hymenoptera	Chalcidoidea	Eurytomidae	Tetramesa	sp.	MG340113.1
Hymenoptera	Chalcidoidea	Hymenoptera	Hymenoptera	sp.	HQ990315.1
Hymenoptera	Chalcidoidea	Hymenoptera	Hymenoptera	sp.	HQ990316.1
Hymenoptera	Chalcidoidea	Hymenoptera	Hymenoptera	sp.	JN288508.1
Hymenoptera	Chalcidoidea	Hymenoptera	Hymenoptera	sp.	JN288509.1
Hymenoptera	Chalcidoidea	Hymenoptera	Hymenoptera	sp.	JN288520.1
Hymenoptera	Chalcidoidea	Hymenoptera	Hymenoptera	sp.	JN288521.1
Hymenoptera	Chalcidoidea	Hymenoptera	Hymenoptera	sp.	JN288672.1
Hymenoptera	Chalcidoidea	Hymenoptera	Hymenoptera	sp.	JN288721.1
Hymenoptera	Chalcidoidea	Hymenoptera	Hymenoptera	sp.	JN288919.1
Hymenoptera	Chalcidoidea	Hymenoptera	Hymenoptera	sp.	JN288920.1
Hymenoptera	Chalcidoidea	Hymenoptera	Hymenoptera	sp.	JN288994.1
Hymenoptera	Chalcidoidea	Hymenoptera	Hymenoptera	sp.	JN288995.1
Hymenoptera	Chalcidoidea	Hymenoptera	Hymenoptera	sp.	JN289000.1
Hymenoptera	Chalcidoidea	Hymenoptera	Hymenoptera	sp.	JN289005.1
Hymenoptera	Chalcidoidea	Hymenoptera	Hymenoptera	sp.	JN289006.1
Hymenoptera	Chalcidoidea	Hymenoptera	Hymenoptera	sp.	JN289007.1
Hymenoptera	Chalcidoidea	Hymenoptera	Hymenoptera	sp.	JN289082.1
Hymenoptera	Chalcidoidea	Hymenoptera	Hymenoptera	sp.	JN289103.1
Hymenoptera	Chalcidoidea	Hymenoptera	Hymenoptera	sp.	JN292167.1
Hymenoptera	Chalcidoidea	Hymenoptera	Hymenoptera	sp.	JN305995.1
Hymenoptera	Chalcidoidea	Leucospidae	Leucospis	affinis	KJ167162.1
Hymenoptera	Chalcidoidea	Megastigmidae	Bootanomyia	sp.	MT375391.1
Hymenoptera	Chalcidoidea	Megastigmidae	Bootanomyia	sp.	MT375392.1
Hymenoptera	Chalcidoidea	Megastigmidae	Megastigmus	aculeatus	JQ756596.1
Hymenoptera	Chalcidoidea	Megastigmidae	Megastigmus	atedius	KU496776.1
Hymenoptera	Chalcidoidea	Megastigmidae	Megastigmus	lawsoni	MT375388.1
Hymenoptera	Chalcidoidea	Megastigmidae	Megastigmus	manonae	MN165923.1
Hymenoptera	Chalcidoidea	Megastigmidae	Megastigmus	manonae	MN165927.1
Hymenoptera	Chalcidoidea	Megastigmidae	Megastigmus	pretorianensis	MN165919.1
Hymenoptera	Chalcidoidea	Megastigmidae	Megastigmus	sp.	GQ367876.1
Hymenoptera	Chalcidoidea	Megastigmidae	Megastigmus	sp.	KF938926.1
Hymenoptera	Chalcidoidea	Megastigmidae	Megastigmus	sp.	KR365347.1
Hymenoptera	Chalcidoidea	Megastigmidae	Megastigmus	sp.	KR366045.1
Hymenoptera	Chalcidoidea	Megastigmidae	Megastigmus	sp.	KR370742.1
Hymenoptera	Chalcidoidea	Megastigmidae	Megastigmus	sp.	KR372971.1
Hymenoptera	Chalcidoidea	Megastigmidae	Megastigmus	sp.	KR374431.1
Hymenoptera	Chalcidoidea	Megastigmidae	Megastigmus	sp.	MT375387.1
Hymenoptera	Chalcidoidea	Megastigmidae	Megastigmus	sp.	MT375393.1

Hymenoptera	Chalcidoidea	Megastigmidae	Megastigmus	sp.	MT375394.1
Hymenoptera	Chalcidoidea	Megastigmidae	Megastigmus	sp.	MT375395.1
Hymenoptera	Chalcidoidea	Megastigmidae	Megastigmus	zebrinus	MN165915.1
Hymenoptera	Chalcidoidea	Megastigmidae	Megastigmus	zvimendeli	MN165914.1
Hymenoptera	Chalcidoidea	Megastigmidae	Megastigmus	zvimendeli	MN165921.1
Hymenoptera	Chalcidoidea	Mymaridae	Acmopolynema	sp.	MG353854.1
Hymenoptera	Chalcidoidea	Mymaridae	Alaptus	sp.	KR784586.1
Hymenoptera	Chalcidoidea	Mymaridae	Alaptus	sp.	KR789509.1
Hymenoptera	Chalcidoidea	Mymaridae	Alaptus	sp.	KR791864.1
Hymenoptera	Chalcidoidea	Mymaridae	Alaptus	sp.	KR797525.1
Hymenoptera	Chalcidoidea	Mymaridae	Alaptus	sp.	KR806688.1
Hymenoptera	Chalcidoidea	Mymaridae	Alaptus	sp.	KR929419.1
Hymenoptera	Chalcidoidea	Mymaridae	Alaptus	sp.	KR934506.1
Hymenoptera	Chalcidoidea	Mymaridae	Alaptus	sp.	KR934615.1
Hymenoptera	Chalcidoidea	Mymaridae	Alaptus	sp.	MG346271.1
Hymenoptera	Chalcidoidea	Mymaridae	Alaptus	sp.	MG346317.1
Hymenoptera	Chalcidoidea	Mymaridae	Alaptus	sp.	MG346575.1
Hymenoptera	Chalcidoidea	Mymaridae	Alaptus	sp.	MG347862.1
Hymenoptera	Chalcidoidea	Mymaridae	Alaptus	sp.	MG347907.1
Hymenoptera	Chalcidoidea	Mymaridae	Alaptus	sp.	MG348621.1
Hymenoptera	Chalcidoidea	Mymaridae	Alaptus	sp.	MG348804.1
Hymenoptera	Chalcidoidea	Mymaridae	Alaptus	sp.	MG348816.1
Hymenoptera	Chalcidoidea	Mymaridae	Alaptus	sp.	MG351342.1
Hymenoptera	Chalcidoidea	Mymaridae	Alaptus	sp.	MG351693.1
Hymenoptera	Chalcidoidea	Mymaridae	Alaptus	sp.	MG352063.1
Hymenoptera	Chalcidoidea	Mymaridae	Alaptus	sp.	MG352644.1
Hymenoptera	Chalcidoidea	Mymaridae	Alaptus	sp.	MG352874.1
Hymenoptera	Chalcidoidea	Mymaridae	Alaptus	sp.	MG353009.1
Hymenoptera	Chalcidoidea	Mymaridae	Alaptus	sp.	MG353332.1
Hymenoptera	Chalcidoidea	Mymaridae	Alaptus	sp.	MG353890.1
Hymenoptera	Chalcidoidea	Mymaridae	Alaptus	sp.	MG354618.1
Hymenoptera	Chalcidoidea	Mymaridae	Alaptus	sp.	MG354818.1
Hymenoptera	Chalcidoidea	Mymaridae	Alaptus	sp.	MG497565.1
Hymenoptera	Chalcidoidea	Mymaridae	Alaptus	sp.	MG499276.1
Hymenoptera	Chalcidoidea	Mymaridae	Alaptus	sp.	MG500322.1
Hymenoptera	Chalcidoidea	Mymaridae	Alaptus	sp.	MG501373.1
Hymenoptera	Chalcidoidea	Mymaridae	Alaptus	sp.	MG503751.1
Hymenoptera	Chalcidoidea	Mymaridae	Alaptus	sp.	MG505710.1
Hymenoptera	Chalcidoidea	Mymaridae	Anagrus	nilaparvatae	MK024863.1
Hymenoptera	Chalcidoidea	Mymaridae	Anagrus	rugmanjonesi	MK544853.1
Hymenoptera	Chalcidoidea	Mymaridae	Anagrus	rugmanjonesi	MK544854.1
Hymenoptera	Chalcidoidea	Mymaridae	Anagrus	rugmanjonesi	MK544855.1
Hymenoptera	Chalcidoidea	Mymaridae	Anagrus	sp.	HQ929801.1
Hymenoptera	Chalcidoidea	Mymaridae	Anagrus	sp.	HQ930350.1
Hymenoptera	Chalcidoidea	Mymaridae	Anagrus	sp.	KJ083564.1
Hymenoptera	Chalcidoidea	Mymaridae	Anagrus	sp.	KJ083618.1
Hymenoptera	Chalcidoidea	Mymaridae	Anagrus	sp.	KJ084106.1
Hymenoptera	Chalcidoidea	Mymaridae	Anagrus	sp.	KJ084310.1
Hymenoptera	Chalcidoidea	Mymaridae	Anagrus	sp.	KJ084625.1
Hymenoptera	Chalcidoidea	Mymaridae	Anagrus	sp.	KJ085167.1
Hymenoptera	Chalcidoidea	Mymaridae	Anagrus	sp.	KJ085325.1
Hymenoptera	Chalcidoidea	Mymaridae	Anagrus	sp.	KJ085503.1
Hymenoptera	Chalcidoidea	Mymaridae	Anagrus	sp.	KJ085645.1
Hymenoptera	Chalcidoidea	Mymaridae	Anagrus	sp.	KJ086483.1

Hymenoptera	Chalcidoidea	Mymaridae	Anagrus	sp.	KJ087005.1
Hymenoptera	Chalcidoidea	Mymaridae	Anagrus	sp.	KJ087805.1
Hymenoptera	Chalcidoidea	Mymaridae	Anagrus	sp.	KJ088088.1
Hymenoptera	Chalcidoidea	Mymaridae	Anagrus	sp.	KJ089066.1
Hymenoptera	Chalcidoidea	Mymaridae	Anagrus	sp.	KJ089489.1
Hymenoptera	Chalcidoidea	Mymaridae	Anagrus	sp.	KJ089592.1
Hymenoptera	Chalcidoidea	Mymaridae	Anagrus	sp.	KJ089915.1
Hymenoptera	Chalcidoidea	Mymaridae	Anagrus	sp.	KJ090128.1
Hymenoptera	Chalcidoidea	Mymaridae	Anagrus	sp.	KJ090680.1
Hymenoptera	Chalcidoidea	Mymaridae	Anagrus	sp.	KJ090795.1
Hymenoptera	Chalcidoidea	Mymaridae	Anagrus	sp.	KJ090928.1
Hymenoptera	Chalcidoidea	Mymaridae	Anagrus	sp.	KJ091079.1
Hymenoptera	Chalcidoidea	Mymaridae	Anagrus	sp.	KJ091169.1
Hymenoptera	Chalcidoidea	Mymaridae	Anagrus	sp.	KJ091893.1
Hymenoptera	Chalcidoidea	Mymaridae	Anagrus	sp.	KJ091981.1
Hymenoptera	Chalcidoidea	Mymaridae	Anagrus	sp.	KJ093067.1
Hymenoptera	Chalcidoidea	Mymaridae	Anagrus	sp.	KJ093217.1
Hymenoptera	Chalcidoidea	Mymaridae	Anagrus	sp.	KJ164571.1
Hymenoptera	Chalcidoidea	Mymaridae	Anagrus	sp.	KJ164733.1
Hymenoptera	Chalcidoidea	Mymaridae	Anagrus	sp.	KJ165279.1
Hymenoptera	Chalcidoidea	Mymaridae	Anagrus	sp.	KJ165987.1
Hymenoptera	Chalcidoidea	Mymaridae	Anagrus	sp.	KJ167812.1
Hymenoptera	Chalcidoidea	Mymaridae	Anagrus	sp.	KJ167833.1
Hymenoptera	Chalcidoidea	Mymaridae	Anagrus	sp.	KJ207486.1
Hymenoptera	Chalcidoidea	Mymaridae	Anagrus	sp.	KJ207497.1
Hymenoptera	Chalcidoidea	Mymaridae	Anagrus	sp.	KJ207580.1
Hymenoptera	Chalcidoidea	Mymaridae	Anagrus	sp.	KJ207582.1
Hymenoptera	Chalcidoidea	Mymaridae	Anagrus	sp.	KJ207729.1
Hymenoptera	Chalcidoidea	Mymaridae	Anagrus	sp.	KJ207767.1
Hymenoptera	Chalcidoidea	Mymaridae	Anagrus	sp.	KJ207845.1
Hymenoptera	Chalcidoidea	Mymaridae	Anagrus	sp.	KJ208076.1
Hymenoptera	Chalcidoidea	Mymaridae	Anagrus	sp.	KJ208104.1
Hymenoptera	Chalcidoidea	Mymaridae	Anagrus	sp.	KJ208266.1
Hymenoptera	Chalcidoidea	Mymaridae	Anagrus	sp.	KJ208319.1
Hymenoptera	Chalcidoidea	Mymaridae	Anagrus	sp.	KJ208350.1
Hymenoptera	Chalcidoidea	Mymaridae	Anagrus	sp.	KJ208553.1
Hymenoptera	Chalcidoidea	Mymaridae	Anagrus	sp.	KJ208635.1
Hymenoptera	Chalcidoidea	Mymaridae	Anagrus	sp.	KJ208641.1
Hymenoptera	Chalcidoidea	Mymaridae	Anagrus	sp.	KJ209174.1
Hymenoptera	Chalcidoidea	Mymaridae	Anagrus	sp.	KJ209235.1
Hymenoptera	Chalcidoidea	Mymaridae	Anagrus	sp.	KJ209353.1
Hymenoptera	Chalcidoidea	Mymaridae	Anagrus	sp.	KJ209427.1
Hymenoptera	Chalcidoidea	Mymaridae	Anagrus	sp.	KJ444569.1
Hymenoptera	Chalcidoidea	Mymaridae	Anagrus	sp.	KJ444673.1
Hymenoptera	Chalcidoidea	Mymaridae	Anagrus	sp.	KJ444849.1
Hymenoptera	Chalcidoidea	Mymaridae	Anagrus	sp.	KJ445309.1
Hymenoptera	Chalcidoidea	Mymaridae	Anagrus	sp.	KJ445322.1
Hymenoptera	Chalcidoidea	Mymaridae	Anagrus	sp.	KJ445623.1
Hymenoptera	Chalcidoidea	Mymaridae	Anagrus	sp.	KM555415.1
Hymenoptera	Chalcidoidea	Mymaridae	Anagrus	sp.	KM555524.1
Hymenoptera	Chalcidoidea	Mymaridae	Anagrus	sp.	KM555559.1
Hymenoptera	Chalcidoidea	Mymaridae	Anagrus	sp.	KM555833.1
Hymenoptera	Chalcidoidea	Mymaridae	Anagrus	sp.	KM556064.1
Hymenoptera	Chalcidoidea	Mymaridae	Anagrus	sp.	KM556106.1

Hymenoptera	Chalcidoidea	Mymaridae	Anagrus	sp.	KM556301.1
Hymenoptera	Chalcidoidea	Mymaridae	Anagrus	sp.	KM556350.1
Hymenoptera	Chalcidoidea	Mymaridae	Anagrus	sp.	KM556531.1
Hymenoptera	Chalcidoidea	Mymaridae	Anagrus	sp.	KM556678.1
Hymenoptera	Chalcidoidea	Mymaridae	Anagrus	sp.	KM556834.1
Hymenoptera	Chalcidoidea	Mymaridae	Anagrus	sp.	KM557043.1
Hymenoptera	Chalcidoidea	Mymaridae	Anagrus	sp.	KM557403.1
Hymenoptera	Chalcidoidea	Mymaridae	Anagrus	sp.	KM557411.1
Hymenoptera	Chalcidoidea	Mymaridae	Anagrus	sp.	KM557471.1
Hymenoptera	Chalcidoidea	Mymaridae	Anagrus	sp.	KM557508.1
Hymenoptera	Chalcidoidea	Mymaridae	Anagrus	sp.	KM557925.1
Hymenoptera	Chalcidoidea	Mymaridae	Anagrus	sp.	KM558109.1
Hymenoptera	Chalcidoidea	Mymaridae	Anagrus	sp.	KM558144.1
Hymenoptera	Chalcidoidea	Mymaridae	Anagrus	sp.	KM558270.1
Hymenoptera	Chalcidoidea	Mymaridae	Anagrus	sp.	KM558353.1
Hymenoptera	Chalcidoidea	Mymaridae	Anagrus	sp.	KM558397.1
Hymenoptera	Chalcidoidea	Mymaridae	Anagrus	sp.	KM558623.1
Hymenoptera	Chalcidoidea	Mymaridae	Anagrus	sp.	KM558683.1
Hymenoptera	Chalcidoidea	Mymaridae	Anagrus	sp.	KM559280.1
Hymenoptera	Chalcidoidea	Mymaridae	Anagrus	sp.	KM559379.1
Hymenoptera	Chalcidoidea	Mymaridae	Anagrus	sp.	KM559863.1
Hymenoptera	Chalcidoidea	Mymaridae	Anagrus	sp.	KM559897.1
Hymenoptera	Chalcidoidea	Mymaridae	Anagrus	sp.	KM560006.1
Hymenoptera	Chalcidoidea	Mymaridae	Anagrus	sp.	KM560207.1
Hymenoptera	Chalcidoidea	Mymaridae	Anagrus	sp.	KM560228.1
Hymenoptera	Chalcidoidea	Mymaridae	Anagrus	sp.	KM560333.1
Hymenoptera	Chalcidoidea	Mymaridae	Anagrus	sp.	KM560418.1
Hymenoptera	Chalcidoidea	Mymaridae	Anagrus	sp.	KM560429.1
Hymenoptera	Chalcidoidea	Mymaridae	Anagrus	sp.	KM560519.1
Hymenoptera	Chalcidoidea	Mymaridae	Anagrus	sp.	KM560722.1
Hymenoptera	Chalcidoidea	Mymaridae	Anagrus	sp.	KM560892.1
Hymenoptera	Chalcidoidea	Mymaridae	Anagrus	sp.	KM560895.1
Hymenoptera	Chalcidoidea	Mymaridae	Anagrus	sp.	KM561302.1
Hymenoptera	Chalcidoidea	Mymaridae	Anagrus	sp.	KM561371.1
Hymenoptera	Chalcidoidea	Mymaridae	Anagrus	sp.	KM561484.1
Hymenoptera	Chalcidoidea	Mymaridae	Anagrus	sp.	KM561620.1
Hymenoptera	Chalcidoidea	Mymaridae	Anagrus	sp.	KM561624.1
Hymenoptera	Chalcidoidea	Mymaridae	Anagrus	sp.	KM561950.1
Hymenoptera	Chalcidoidea	Mymaridae	Anagrus	sp.	KM562015.1
Hymenoptera	Chalcidoidea	Mymaridae	Anagrus	sp.	KM562064.1
Hymenoptera	Chalcidoidea	Mymaridae	Anagrus	sp.	KM562363.1
Hymenoptera	Chalcidoidea	Mymaridae	Anagrus	sp.	KM562540.1
Hymenoptera	Chalcidoidea	Mymaridae	Anagrus	sp.	KM562564.1
Hymenoptera	Chalcidoidea	Mymaridae	Anagrus	sp.	KM562831.1
Hymenoptera	Chalcidoidea	Mymaridae	Anagrus	sp.	KM563515.1
Hymenoptera	Chalcidoidea	Mymaridae	Anagrus	sp.	KM563832.1
Hymenoptera	Chalcidoidea	Mymaridae	Anagrus	sp.	KM563908.1
Hymenoptera	Chalcidoidea	Mymaridae	Anagrus	sp.	KM563912.1
Hymenoptera	Chalcidoidea	Mymaridae	Anagrus	sp.	KM564068.1
Hymenoptera	Chalcidoidea	Mymaridae	Anagrus	sp.	KM564294.1
Hymenoptera	Chalcidoidea	Mymaridae	Anagrus	sp.	KM564743.1
Hymenoptera	Chalcidoidea	Mymaridae	Anagrus	sp.	KM564872.1
Hymenoptera	Chalcidoidea	Mymaridae	Anagrus	sp.	KM565076.1
Hymenoptera	Chalcidoidea	Mymaridae	Anagrus	sp.	KM565345.1

Hymenoptera	Chalcidoidea	Mymaridae	Anagrus	sp.	KM566039.1
Hymenoptera	Chalcidoidea	Mymaridae	Anagrus	sp.	KM566180.1
Hymenoptera	Chalcidoidea	Mymaridae	Anagrus	sp.	KM566201.1
Hymenoptera	Chalcidoidea	Mymaridae	Anagrus	sp.	KM566340.1
Hymenoptera	Chalcidoidea	Mymaridae	Anagrus	sp.	KM566377.1
Hymenoptera	Chalcidoidea	Mymaridae	Anagrus	sp.	KM566716.1
Hymenoptera	Chalcidoidea	Mymaridae	Anagrus	sp.	KM567162.1
Hymenoptera	Chalcidoidea	Mymaridae	Anagrus	sp.	KM567784.1
Hymenoptera	Chalcidoidea	Mymaridae	Anagrus	sp.	KM567857.1
Hymenoptera	Chalcidoidea	Mymaridae	Anagrus	sp.	KM567985.1
Hymenoptera	Chalcidoidea	Mymaridae	Anagrus	sp.	KM568810.1
Hymenoptera	Chalcidoidea	Mymaridae	Anagrus	sp.	KM569289.1
Hymenoptera	Chalcidoidea	Mymaridae	Anagrus	sp.	KR401408.1
Hymenoptera	Chalcidoidea	Mymaridae	Anagrus	sp.	KR401432.1
Hymenoptera	Chalcidoidea	Mymaridae	Anagrus	sp.	KR402077.1
Hymenoptera	Chalcidoidea	Mymaridae	Anagrus	sp.	KR402242.1
Hymenoptera	Chalcidoidea	Mymaridae	Anagrus	sp.	KR402579.1
Hymenoptera	Chalcidoidea	Mymaridae	Anagrus	sp.	KR402795.1
Hymenoptera	Chalcidoidea	Mymaridae	Anagrus	sp.	KR403390.1
Hymenoptera	Chalcidoidea	Mymaridae	Anagrus	sp.	KR403520.1
Hymenoptera	Chalcidoidea	Mymaridae	Anagrus	sp.	KR404184.1
Hymenoptera	Chalcidoidea	Mymaridae	Anagrus	sp.	KR404244.1
Hymenoptera	Chalcidoidea	Mymaridae	Anagrus	sp.	KR404510.1
Hymenoptera	Chalcidoidea	Mymaridae	Anagrus	sp.	KR404657.1
Hymenoptera	Chalcidoidea	Mymaridae	Anagrus	sp.	KR405774.1
Hymenoptera	Chalcidoidea	Mymaridae	Anagrus	sp.	KR406316.1
Hymenoptera	Chalcidoidea	Mymaridae	Anagrus	sp.	KR406901.1
Hymenoptera	Chalcidoidea	Mymaridae	Anagrus	sp.	KR406969.1
Hymenoptera	Chalcidoidea	Mymaridae	Anagrus	sp.	KR407239.1
Hymenoptera	Chalcidoidea	Mymaridae	Anagrus	sp.	KR407548.1
Hymenoptera	Chalcidoidea	Mymaridae	Anagrus	sp.	KR407815.1
Hymenoptera	Chalcidoidea	Mymaridae	Anagrus	sp.	KR407832.1
Hymenoptera	Chalcidoidea	Mymaridae	Anagrus	sp.	KR408530.1
Hymenoptera	Chalcidoidea	Mymaridae	Anagrus	sp.	KR409265.1
Hymenoptera	Chalcidoidea	Mymaridae	Anagrus	sp.	KR409425.1
Hymenoptera	Chalcidoidea	Mymaridae	Anagrus	sp.	KR409480.1
Hymenoptera	Chalcidoidea	Mymaridae	Anagrus	sp.	KR409992.1
Hymenoptera	Chalcidoidea	Mymaridae	Anagrus	sp.	KR410317.1
Hymenoptera	Chalcidoidea	Mymaridae	Anagrus	sp.	KR410348.1
Hymenoptera	Chalcidoidea	Mymaridae	Anagrus	sp.	KR410666.1
Hymenoptera	Chalcidoidea	Mymaridae	Anagrus	sp.	KR410851.1
Hymenoptera	Chalcidoidea	Mymaridae	Anagrus	sp.	KR411213.1
Hymenoptera	Chalcidoidea	Mymaridae	Anagrus	sp.	KR411226.1
Hymenoptera	Chalcidoidea	Mymaridae	Anagrus	sp.	KR411754.1
Hymenoptera	Chalcidoidea	Mymaridae	Anagrus	sp.	KR413057.1
Hymenoptera	Chalcidoidea	Mymaridae	Anagrus	sp.	KR413268.1
Hymenoptera	Chalcidoidea	Mymaridae	Anagrus	sp.	KR413759.1
Hymenoptera	Chalcidoidea	Mymaridae	Anagrus	sp.	KR413947.1
Hymenoptera	Chalcidoidea	Mymaridae	Anagrus	sp.	KR414084.1
Hymenoptera	Chalcidoidea	Mymaridae	Anagrus	sp.	KR414268.1
Hymenoptera	Chalcidoidea	Mymaridae	Anagrus	sp.	KR414626.1
Hymenoptera	Chalcidoidea	Mymaridae	Anagrus	sp.	KR414691.1
Hymenoptera	Chalcidoidea	Mymaridae	Anagrus	sp.	KR415208.1
Hymenoptera	Chalcidoidea	Mymaridae	Anagrus	sp.	KR415554.1

Hymenoptera	Chalcidoidea	Mymaridae	Anagrus	sp.	KR415943.1
Hymenoptera	Chalcidoidea	Mymaridae	Anagrus	sp.	KR415955.1
Hymenoptera	Chalcidoidea	Mymaridae	Anagrus	sp.	KR416388.1
Hymenoptera	Chalcidoidea	Mymaridae	Anagrus	sp.	KR416639.1
Hymenoptera	Chalcidoidea	Mymaridae	Anagrus	sp.	KR416725.1
Hymenoptera	Chalcidoidea	Mymaridae	Anagrus	sp.	KR416752.1
Hymenoptera	Chalcidoidea	Mymaridae	Anagrus	sp.	KR416877.1
Hymenoptera	Chalcidoidea	Mymaridae	Anagrus	sp.	KR417316.1
Hymenoptera	Chalcidoidea	Mymaridae	Anagrus	sp.	KR417624.1
Hymenoptera	Chalcidoidea	Mymaridae	Anagrus	sp.	KR417664.1
Hymenoptera	Chalcidoidea	Mymaridae	Anagrus	sp.	KR417865.1
Hymenoptera	Chalcidoidea	Mymaridae	Anagrus	sp.	KR418355.1
Hymenoptera	Chalcidoidea	Mymaridae	Anagrus	sp.	KR419337.1
Hymenoptera	Chalcidoidea	Mymaridae	Anagrus	sp.	KR419479.1
Hymenoptera	Chalcidoidea	Mymaridae	Anagrus	sp.	KR419619.1
Hymenoptera	Chalcidoidea	Mymaridae	Anagrus	sp.	KR420719.1
Hymenoptera	Chalcidoidea	Mymaridae	Anagrus	sp.	KR420740.1
Hymenoptera	Chalcidoidea	Mymaridae	Anagrus	sp.	KR421600.1
Hymenoptera	Chalcidoidea	Mymaridae	Anagrus	sp.	KR421781.1
Hymenoptera	Chalcidoidea	Mymaridae	Anagrus	sp.	KR422006.1
Hymenoptera	Chalcidoidea	Mymaridae	Anagrus	sp.	KR782524.1
Hymenoptera	Chalcidoidea	Mymaridae	Anagrus	sp.	KR782638.1
Hymenoptera	Chalcidoidea	Mymaridae	Anagrus	sp.	KR783411.1
Hymenoptera	Chalcidoidea	Mymaridae	Anagrus	sp.	KR783556.1
Hymenoptera	Chalcidoidea	Mymaridae	Anagrus	sp.	KR783740.1
Hymenoptera	Chalcidoidea	Mymaridae	Anagrus	sp.	KR783886.1
Hymenoptera	Chalcidoidea	Mymaridae	Anagrus	sp.	KR783923.1
Hymenoptera	Chalcidoidea	Mymaridae	Anagrus	sp.	KR784022.1
Hymenoptera	Chalcidoidea	Mymaridae	Anagrus	sp.	KR784131.1
Hymenoptera	Chalcidoidea	Mymaridae	Anagrus	sp.	KR784869.1
Hymenoptera	Chalcidoidea	Mymaridae	Anagrus	sp.	KR785049.1
Hymenoptera	Chalcidoidea	Mymaridae	Anagrus	sp.	KR785546.1
Hymenoptera	Chalcidoidea	Mymaridae	Anagrus	sp.	KR785591.1
Hymenoptera	Chalcidoidea	Mymaridae	Anagrus	sp.	KR785825.1
Hymenoptera	Chalcidoidea	Mymaridae	Anagrus	sp.	KR785865.1
Hymenoptera	Chalcidoidea	Mymaridae	Anagrus	sp.	KR786897.1
Hymenoptera	Chalcidoidea	Mymaridae	Anagrus	sp.	KR787031.1
Hymenoptera	Chalcidoidea	Mymaridae	Anagrus	sp.	KR787277.1
Hymenoptera	Chalcidoidea	Mymaridae	Anagrus	sp.	KR787827.1
Hymenoptera	Chalcidoidea	Mymaridae	Anagrus	sp.	KR788652.1
Hymenoptera	Chalcidoidea	Mymaridae	Anagrus	sp.	KR789000.1
Hymenoptera	Chalcidoidea	Mymaridae	Anagrus	sp.	KR789536.1
Hymenoptera	Chalcidoidea	Mymaridae	Anagrus	sp.	KR790151.1
Hymenoptera	Chalcidoidea	Mymaridae	Anagrus	sp.	KR790787.1
Hymenoptera	Chalcidoidea	Mymaridae	Anagrus	sp.	KR790947.1
Hymenoptera	Chalcidoidea	Mymaridae	Anagrus	sp.	KR791601.1
Hymenoptera	Chalcidoidea	Mymaridae	Anagrus	sp.	KR792417.1
Hymenoptera	Chalcidoidea	Mymaridae	Anagrus	sp.	KR792812.1
Hymenoptera	Chalcidoidea	Mymaridae	Anagrus	sp.	KR793168.1
Hymenoptera	Chalcidoidea	Mymaridae	Anagrus	sp.	KR793777.1
Hymenoptera	Chalcidoidea	Mymaridae	Anagrus	sp.	KR793887.1
Hymenoptera	Chalcidoidea	Mymaridae	Anagrus	sp.	KR794653.1
Hymenoptera	Chalcidoidea	Mymaridae	Anagrus	sp.	KR794929.1
Hymenoptera	Chalcidoidea	Mymaridae	Anagrus	sp.	KR796001.1

Hymenoptera	Chalcidoidea	Mymaridae	Anagrus	sp.	KR798375.1
Hymenoptera	Chalcidoidea	Mymaridae	Anagrus	sp.	KR798667.1
Hymenoptera	Chalcidoidea	Mymaridae	Anagrus	sp.	KR799411.1
Hymenoptera	Chalcidoidea	Mymaridae	Anagrus	sp.	KR799453.1
Hymenoptera	Chalcidoidea	Mymaridae	Anagrus	sp.	KR799961.1
Hymenoptera	Chalcidoidea	Mymaridae	Anagrus	sp.	KR800325.1
Hymenoptera	Chalcidoidea	Mymaridae	Anagrus	sp.	KR800524.1
Hymenoptera	Chalcidoidea	Mymaridae	Anagrus	sp.	KR801100.1
Hymenoptera	Chalcidoidea	Mymaridae	Anagrus	sp.	KR801916.1
Hymenoptera	Chalcidoidea	Mymaridae	Anagrus	sp.	KR802003.1
Hymenoptera	Chalcidoidea	Mymaridae	Anagrus	sp.	KR802447.1
Hymenoptera	Chalcidoidea	Mymaridae	Anagrus	sp.	KR803120.1
Hymenoptera	Chalcidoidea	Mymaridae	Anagrus	sp.	KR803203.1
Hymenoptera	Chalcidoidea	Mymaridae	Anagrus	sp.	KR803241.1
Hymenoptera	Chalcidoidea	Mymaridae	Anagrus	sp.	KR803999.1
Hymenoptera	Chalcidoidea	Mymaridae	Anagrus	sp.	KR804618.1
Hymenoptera	Chalcidoidea	Mymaridae	Anagrus	sp.	KR804852.1
Hymenoptera	Chalcidoidea	Mymaridae	Anagrus	sp.	KR804907.1
Hymenoptera	Chalcidoidea	Mymaridae	Anagrus	sp.	KR804913.1
Hymenoptera	Chalcidoidea	Mymaridae	Anagrus	sp.	KR805249.1
Hymenoptera	Chalcidoidea	Mymaridae	Anagrus	sp.	KR806865.1
Hymenoptera	Chalcidoidea	Mymaridae	Anagrus	sp.	KR806924.1
Hymenoptera	Chalcidoidea	Mymaridae	Anagrus	sp.	KR808691.1
Hymenoptera	Chalcidoidea	Mymaridae	Anagrus	sp.	KR808765.1
Hymenoptera	Chalcidoidea	Mymaridae	Anagrus	sp.	KR873459.1
Hymenoptera	Chalcidoidea	Mymaridae	Anagrus	sp.	KR874085.1
Hymenoptera	Chalcidoidea	Mymaridae	Anagrus	sp.	KR874173.1
Hymenoptera	Chalcidoidea	Mymaridae	Anagrus	sp.	KR874513.1
Hymenoptera	Chalcidoidea	Mymaridae	Anagrus	sp.	KR874875.1
Hymenoptera	Chalcidoidea	Mymaridae	Anagrus	sp.	KR875510.1
Hymenoptera	Chalcidoidea	Mymaridae	Anagrus	sp.	KR876522.1
Hymenoptera	Chalcidoidea	Mymaridae	Anagrus	sp.	KR877260.1
Hymenoptera	Chalcidoidea	Mymaridae	Anagrus	sp.	KR878154.1
Hymenoptera	Chalcidoidea	Mymaridae	Anagrus	sp.	KR878417.1
Hymenoptera	Chalcidoidea	Mymaridae	Anagrus	sp.	KR879339.1
Hymenoptera	Chalcidoidea	Mymaridae	Anagrus	sp.	KR880707.1
Hymenoptera	Chalcidoidea	Mymaridae	Anagrus	sp.	KR880896.1
Hymenoptera	Chalcidoidea	Mymaridae	Anagrus	sp.	KR881406.1
Hymenoptera	Chalcidoidea	Mymaridae	Anagrus	sp.	KR881870.1
Hymenoptera	Chalcidoidea	Mymaridae	Anagrus	sp.	KR884506.1
Hymenoptera	Chalcidoidea	Mymaridae	Anagrus	sp.	KR885653.1
Hymenoptera	Chalcidoidea	Mymaridae	Anagrus	sp.	KR885916.1
Hymenoptera	Chalcidoidea	Mymaridae	Anagrus	sp.	KR885935.1
Hymenoptera	Chalcidoidea	Mymaridae	Anagrus	sp.	KR886535.1
Hymenoptera	Chalcidoidea	Mymaridae	Anagrus	sp.	KR886857.1
Hymenoptera	Chalcidoidea	Mymaridae	Anagrus	sp.	KR887792.1
Hymenoptera	Chalcidoidea	Mymaridae	Anagrus	sp.	KR888101.1
Hymenoptera	Chalcidoidea	Mymaridae	Anagrus	sp.	KR888312.1
Hymenoptera	Chalcidoidea	Mymaridae	Anagrus	sp.	KR890038.1
Hymenoptera	Chalcidoidea	Mymaridae	Anagrus	sp.	KR890204.1
Hymenoptera	Chalcidoidea	Mymaridae	Anagrus	sp.	KR892211.1
Hymenoptera	Chalcidoidea	Mymaridae	Anagrus	sp.	KR892595.1
Hymenoptera	Chalcidoidea	Mymaridae	Anagrus	sp.	KR893691.1
Hymenoptera	Chalcidoidea	Mymaridae	Anagrus	sp.	KR893724.1

Hymenoptera	Chalcidoidea	Mymaridae	Anagrus	sp.	KR893754.1
Hymenoptera	Chalcidoidea	Mymaridae	Anagrus	sp.	KR894088.1
Hymenoptera	Chalcidoidea	Mymaridae	Anagrus	sp.	KR894173.1
Hymenoptera	Chalcidoidea	Mymaridae	Anagrus	sp.	KR894395.1
Hymenoptera	Chalcidoidea	Mymaridae	Anagrus	sp.	KR894845.1
Hymenoptera	Chalcidoidea	Mymaridae	Anagrus	sp.	KR895122.1
Hymenoptera	Chalcidoidea	Mymaridae	Anagrus	sp.	KR895776.1
Hymenoptera	Chalcidoidea	Mymaridae	Anagrus	sp.	KR896304.1
Hymenoptera	Chalcidoidea	Mymaridae	Anagrus	sp.	KR896564.1
Hymenoptera	Chalcidoidea	Mymaridae	Anagrus	sp.	KR896634.1
Hymenoptera	Chalcidoidea	Mymaridae	Anagrus	sp.	KR896715.1
Hymenoptera	Chalcidoidea	Mymaridae	Anagrus	sp.	KR896903.1
Hymenoptera	Chalcidoidea	Mymaridae	Anagrus	sp.	KR896924.1
Hymenoptera	Chalcidoidea	Mymaridae	Anagrus	sp.	KR897215.1
Hymenoptera	Chalcidoidea	Mymaridae	Anagrus	sp.	KR897498.1
Hymenoptera	Chalcidoidea	Mymaridae	Anagrus	sp.	KR897931.1
Hymenoptera	Chalcidoidea	Mymaridae	Anagrus	sp.	KR898473.1
Hymenoptera	Chalcidoidea	Mymaridae	Anagrus	sp.	KR899497.1
Hymenoptera	Chalcidoidea	Mymaridae	Anagrus	sp.	KR901203.1
Hymenoptera	Chalcidoidea	Mymaridae	Anagrus	sp.	KT603787.1
Hymenoptera	Chalcidoidea	Mymaridae	Anagrus	sp.	KT604127.1
Hymenoptera	Chalcidoidea	Mymaridae	Anagrus	sp.	KT604288.1
Hymenoptera	Chalcidoidea	Mymaridae	Anagrus	sp.	KT604446.1
Hymenoptera	Chalcidoidea	Mymaridae	Anagrus	sp.	KT604914.1
Hymenoptera	Chalcidoidea	Mymaridae	Anagrus	sp.	KT605337.1
Hymenoptera	Chalcidoidea	Mymaridae	Anagrus	sp.	KT605955.1
Hymenoptera	Chalcidoidea	Mymaridae	Anagrus	sp.	KT607234.1
Hymenoptera	Chalcidoidea	Mymaridae	Anagrus	sp.	KT607262.1
Hymenoptera	Chalcidoidea	Mymaridae	Anagrus	sp.	KT607355.1
Hymenoptera	Chalcidoidea	Mymaridae	Anagrus	sp.	KT607573.1
Hymenoptera	Chalcidoidea	Mymaridae	Anagrus	sp.	KU702641.1
Hymenoptera	Chalcidoidea	Mymaridae	Anagrus	sp.	KX535018.1
Hymenoptera	Chalcidoidea	Mymaridae	Anagrus	sp.	KY847127.1
Hymenoptera	Chalcidoidea	Mymaridae	Anagrus	sp.	MG346905.1
Hymenoptera	Chalcidoidea	Mymaridae	Anagrus	sp.	MG347250.1
Hymenoptera	Chalcidoidea	Mymaridae	Anagrus	sp.	MG347628.1
Hymenoptera	Chalcidoidea	Mymaridae	Anagrus	sp.	MG347934.1
Hymenoptera	Chalcidoidea	Mymaridae	Anagrus	sp.	MG348006.1
Hymenoptera	Chalcidoidea	Mymaridae	Anagrus	sp.	MG348609.1
Hymenoptera	Chalcidoidea	Mymaridae	Anagrus	sp.	MG348743.1
Hymenoptera	Chalcidoidea	Mymaridae	Anagrus	sp.	MG348793.1
Hymenoptera	Chalcidoidea	Mymaridae	Anagrus	sp.	MG349142.1
Hymenoptera	Chalcidoidea	Mymaridae	Anagrus	sp.	MG349870.1
Hymenoptera	Chalcidoidea	Mymaridae	Anagrus	sp.	MG350544.1
Hymenoptera	Chalcidoidea	Mymaridae	Anagrus	sp.	MG350872.1
Hymenoptera	Chalcidoidea	Mymaridae	Anagrus	sp.	MG350937.1
Hymenoptera	Chalcidoidea	Mymaridae	Anagrus	sp.	MG351054.1
Hymenoptera	Chalcidoidea	Mymaridae	Anagrus	sp.	MG351232.1
Hymenoptera	Chalcidoidea	Mymaridae	Anagrus	sp.	MG351515.1
Hymenoptera	Chalcidoidea	Mymaridae	Anagrus	sp.	MG351634.1
Hymenoptera	Chalcidoidea	Mymaridae	Anagrus	sp.	MG352007.1
Hymenoptera	Chalcidoidea	Mymaridae	Anagrus	sp.	MG352479.1
Hymenoptera	Chalcidoidea	Mymaridae	Anagrus	sp.	MG352529.1
Hymenoptera	Chalcidoidea	Mymaridae	Anagrus	sp.	MG352788.1

Hymenoptera	Chalcidoidea	Mymaridae	Anagrus	sp.	MG352930.1
Hymenoptera	Chalcidoidea	Mymaridae	Anagrus	sp.	MG353554.1
Hymenoptera	Chalcidoidea	Mymaridae	Anagrus	sp.	MG354086.1
Hymenoptera	Chalcidoidea	Mymaridae	Anagrus	sp.	MG354578.1
Hymenoptera	Chalcidoidea	Mymaridae	Anagrus	sp.	MG354694.1
Hymenoptera	Chalcidoidea	Mymaridae	Anagrus	sp.	MG355474.1
Hymenoptera	Chalcidoidea	Mymaridae	Anagrus	sp.	MG481499.1
Hymenoptera	Chalcidoidea	Mymaridae	Anagrus	sp.	MG501474.1
Hymenoptera	Chalcidoidea	Mymaridae	Anagrus	sp.	MG502696.1
Hymenoptera	Chalcidoidea	Mymaridae	Anagrus	sp.	MG503029.1
Hymenoptera	Chalcidoidea	Mymaridae	Anagrus	sp.	MG503574.1
Hymenoptera	Chalcidoidea	Mymaridae	Anagrus	sp.	MG505921.1
Hymenoptera	Chalcidoidea	Mymaridae	Anagrus	sp.	MG509375.1
Hymenoptera	Chalcidoidea	Mymaridae	Anagrus	sp.	MG510977.1
Hymenoptera	Chalcidoidea	Mymaridae	Anagrus	sp.	MG511743.1
Hymenoptera	Chalcidoidea	Mymaridae	Anagrus	sp.	MG911990.1
Hymenoptera	Chalcidoidea	Mymaridae	Anagrus	sp.	MG911991.1
Hymenoptera	Chalcidoidea	Mymaridae	Anagrus	sp.	MG911992.1
Hymenoptera	Chalcidoidea	Mymaridae	Anagrus	sp.	MG911994.1
Hymenoptera	Chalcidoidea	Mymaridae	Anagrus	sp.	MG911997.1
Hymenoptera	Chalcidoidea	Mymaridae	Anagrus	sp.	MG912000.1
Hymenoptera	Chalcidoidea	Mymaridae	Anagrus	sp.	MG912001.1
Hymenoptera	Chalcidoidea	Mymaridae	Anagrus	sp.	MG912004.1
Hymenoptera	Chalcidoidea	Mymaridae	Anagrus	sp.	MG912005.1
Hymenoptera	Chalcidoidea	Mymaridae	Anagrus	sp.	MG912006.1
Hymenoptera	Chalcidoidea	Mymaridae	Anagrus	sp.	MG912008.1
Hymenoptera	Chalcidoidea	Mymaridae	Anagrus	sp.	MG912009.1
Hymenoptera	Chalcidoidea	Mymaridae	Anagrus	sp.	MN665469.1
Hymenoptera	Chalcidoidea	Mymaridae	Anagrus	sp.	MN665725.1
Hymenoptera	Chalcidoidea	Mymaridae	Anagrus	sp.	MN666262.1
Hymenoptera	Chalcidoidea	Mymaridae	Anagrus	sp.	MN667174.1
Hymenoptera	Chalcidoidea	Mymaridae	Anagrus	sp.	MN667302.1
Hymenoptera	Chalcidoidea	Mymaridae	Anagrus	sp.	MN667454.1
Hymenoptera	Chalcidoidea	Mymaridae	Anagrus	sp.	MN667629.1
Hymenoptera	Chalcidoidea	Mymaridae	Anagrus	sp.	MN668211.1
Hymenoptera	Chalcidoidea	Mymaridae	Anagrus	sp.	MN668229.1
Hymenoptera	Chalcidoidea	Mymaridae	Anagrus	sp.	MN668487.1
Hymenoptera	Chalcidoidea	Mymaridae	Anagrus	sp.	MN668575.1
Hymenoptera	Chalcidoidea	Mymaridae	Anagrus	sp.	MN669391.1
Hymenoptera	Chalcidoidea	Mymaridae	Anagrus	sp.	MN669495.1
Hymenoptera	Chalcidoidea	Mymaridae	Anagrus	sp.	MN669582.1
Hymenoptera	Chalcidoidea	Mymaridae	Anagrus	sp.	MN670505.1
Hymenoptera	Chalcidoidea	Mymaridae	Anagrus	sp.	MN670961.1
Hymenoptera	Chalcidoidea	Mymaridae	Anagrus	sp.	MN673544.1
Hymenoptera	Chalcidoidea	Mymaridae	Anagrus	sp.	MN673849.1
Hymenoptera	Chalcidoidea	Mymaridae	Anagrus	sp.	MN674038.1
Hymenoptera	Chalcidoidea	Mymaridae	Anagrus	sp.	MN674493.1
Hymenoptera	Chalcidoidea	Mymaridae	Anagrus	sp.	MN675452.1
Hymenoptera	Chalcidoidea	Mymaridae	Anagrus	sp.	MN675590.1
Hymenoptera	Chalcidoidea	Mymaridae	Anagrus	sp.	MN678972.1
Hymenoptera	Chalcidoidea	Mymaridae	Anagrus	sp.	MN681897.1
Hymenoptera	Chalcidoidea	Mymaridae	Anagrus	sp.	MN682653.1
Hymenoptera	Chalcidoidea	Mymaridae	Anagrus	sp.	MN682794.1
Hymenoptera	Chalcidoidea	Mymaridae	Anagrus	sp.	MN683023.1

Hymenoptera	Chalcidoidea	Mymaridae	Anagrus	ustulatus	MG349999.1
Hymenoptera	Chalcidoidea	Mymaridae	Anaphes	listronoti	KM568040.1
Hymenoptera	Chalcidoidea	Mymaridae	Anaphes	listronoti	KR784571.1
Hymenoptera	Chalcidoidea	Mymaridae	Anaphes	listronoti	KR785030.1
Hymenoptera	Chalcidoidea	Mymaridae	Anaphes	listronoti	KR786122.1
Hymenoptera	Chalcidoidea	Mymaridae	Anaphes	listronoti	KR788838.1
Hymenoptera	Chalcidoidea	Mymaridae	Anaphes	listronoti	KR789185.1
Hymenoptera	Chalcidoidea	Mymaridae	Anaphes	listronoti	KR791311.1
Hymenoptera	Chalcidoidea	Mymaridae	Anaphes	listronoti	KR791410.1
Hymenoptera	Chalcidoidea	Mymaridae	Anaphes	listronoti	KR795866.1
Hymenoptera	Chalcidoidea	Mymaridae	Anaphes	listronoti	KR797898.1
Hymenoptera	Chalcidoidea	Mymaridae	Anaphes	listronoti	KR799525.1
Hymenoptera	Chalcidoidea	Mymaridae	Anaphes	listronoti	KR801176.1
Hymenoptera	Chalcidoidea	Mymaridae	Anaphes	listronoti	KR803093.1
Hymenoptera	Chalcidoidea	Mymaridae	Anaphes	listronoti	KR803332.1
Hymenoptera	Chalcidoidea	Mymaridae	Anaphes	listronoti	KR804514.1
Hymenoptera	Chalcidoidea	Mymaridae	Anaphes	listronoti	KR805002.1
Hymenoptera	Chalcidoidea	Mymaridae	Anaphes	listronoti	KR806361.1
Hymenoptera	Chalcidoidea	Mymaridae	Anaphes	listronoti	KR807721.1
Hymenoptera	Chalcidoidea	Mymaridae	Anaphes	listronoti	KR876851.1
Hymenoptera	Chalcidoidea	Mymaridae	Anaphes	listronoti	KR882468.1
Hymenoptera	Chalcidoidea	Mymaridae	Anaphes	listronoti	KR882585.1
Hymenoptera	Chalcidoidea	Mymaridae	Anaphes	listronoti	KR886306.1
Hymenoptera	Chalcidoidea	Mymaridae	Anaphes	listronoti	KR888580.1
Hymenoptera	Chalcidoidea	Mymaridae	Anaphes	listronoti	KR888999.1
Hymenoptera	Chalcidoidea	Mymaridae	Anaphes	sp.	KM558614.1
Hymenoptera	Chalcidoidea	Mymaridae	Anaphes	sp.	KM559503.1
Hymenoptera	Chalcidoidea	Mymaridae	Anaphes	sp.	KM561321.1
Hymenoptera	Chalcidoidea	Mymaridae	Anaphes	sp.	KM562898.1
Hymenoptera	Chalcidoidea	Mymaridae	Anaphes	sp.	KM562999.1
Hymenoptera	Chalcidoidea	Mymaridae	Anaphes	sp.	KM564161.1
Hymenoptera	Chalcidoidea	Mymaridae	Anaphes	sp.	KM565725.1
Hymenoptera	Chalcidoidea	Mymaridae	Anaphes	sp.	KM566684.1
Hymenoptera	Chalcidoidea	Mymaridae	Anaphes	sp.	KM569240.1
Hymenoptera	Chalcidoidea	Mymaridae	Anaphes	sp.	KR401707.1
Hymenoptera	Chalcidoidea	Mymaridae	Anaphes	sp.	KR404364.1
Hymenoptera	Chalcidoidea	Mymaridae	Anaphes	sp.	KR405444.1
Hymenoptera	Chalcidoidea	Mymaridae	Anaphes	sp.	KR405786.1
Hymenoptera	Chalcidoidea	Mymaridae	Anaphes	sp.	KR406070.1
Hymenoptera	Chalcidoidea	Mymaridae	Anaphes	sp.	KR407173.1
Hymenoptera	Chalcidoidea	Mymaridae	Anaphes	sp.	KR407549.1
Hymenoptera	Chalcidoidea	Mymaridae	Anaphes	sp.	KR408461.1
Hymenoptera	Chalcidoidea	Mymaridae	Anaphes	sp.	KR410775.1
Hymenoptera	Chalcidoidea	Mymaridae	Anaphes	sp.	KR411878.1
Hymenoptera	Chalcidoidea	Mymaridae	Anaphes	sp.	KR412866.1
Hymenoptera	Chalcidoidea	Mymaridae	Anaphes	sp.	KR415660.1
Hymenoptera	Chalcidoidea	Mymaridae	Anaphes	sp.	KR415828.1
Hymenoptera	Chalcidoidea	Mymaridae	Anaphes	sp.	KR417195.1
Hymenoptera	Chalcidoidea	Mymaridae	Anaphes	sp.	KR417531.1
Hymenoptera	Chalcidoidea	Mymaridae	Anaphes	sp.	KR418318.1
Hymenoptera	Chalcidoidea	Mymaridae	Anaphes	sp.	KR418801.1
Hymenoptera	Chalcidoidea	Mymaridae	Anaphes	sp.	KR419838.1
Hymenoptera	Chalcidoidea	Mymaridae	Anaphes	sp.	KR420120.1
Hymenoptera	Chalcidoidea	Mymaridae	Anaphes	sp.	KR421660.1

Hymenoptera	Chalcidoidea	Mymaridae	Anaphes	sp.	KR422115.1
Hymenoptera	Chalcidoidea	Mymaridae	Anaphes	sp.	KR782452.1
Hymenoptera	Chalcidoidea	Mymaridae	Anaphes	sp.	KR782621.1
Hymenoptera	Chalcidoidea	Mymaridae	Anaphes	sp.	KR786146.1
Hymenoptera	Chalcidoidea	Mymaridae	Anaphes	sp.	KR786734.1
Hymenoptera	Chalcidoidea	Mymaridae	Anaphes	sp.	KR788564.1
Hymenoptera	Chalcidoidea	Mymaridae	Anaphes	sp.	KR789157.1
Hymenoptera	Chalcidoidea	Mymaridae	Anaphes	sp.	KR790742.1
Hymenoptera	Chalcidoidea	Mymaridae	Anaphes	sp.	KR792867.1
Hymenoptera	Chalcidoidea	Mymaridae	Anaphes	sp.	KR792914.1
Hymenoptera	Chalcidoidea	Mymaridae	Anaphes	sp.	KR793133.1
Hymenoptera	Chalcidoidea	Mymaridae	Anaphes	sp.	KR794480.1
Hymenoptera	Chalcidoidea	Mymaridae	Anaphes	sp.	KR795834.1
Hymenoptera	Chalcidoidea	Mymaridae	Anaphes	sp.	KR796061.1
Hymenoptera	Chalcidoidea	Mymaridae	Anaphes	sp.	KR797844.1
Hymenoptera	Chalcidoidea	Mymaridae	Anaphes	sp.	KR800520.1
Hymenoptera	Chalcidoidea	Mymaridae	Anaphes	sp.	KR800634.1
Hymenoptera	Chalcidoidea	Mymaridae	Anaphes	sp.	KR800913.1
Hymenoptera	Chalcidoidea	Mymaridae	Anaphes	sp.	KR801917.1
Hymenoptera	Chalcidoidea	Mymaridae	Anaphes	sp.	KR802548.1
Hymenoptera	Chalcidoidea	Mymaridae	Anaphes	sp.	KR803430.1
Hymenoptera	Chalcidoidea	Mymaridae	Anaphes	sp.	KR804271.1
Hymenoptera	Chalcidoidea	Mymaridae	Anaphes	sp.	KR804364.1
Hymenoptera	Chalcidoidea	Mymaridae	Anaphes	sp.	KR878204.1
Hymenoptera	Chalcidoidea	Mymaridae	Anaphes	sp.	KR878641.1
Hymenoptera	Chalcidoidea	Mymaridae	Anaphes	sp.	KR884457.1
Hymenoptera	Chalcidoidea	Mymaridae	Anaphes	sp.	KR886077.1
Hymenoptera	Chalcidoidea	Mymaridae	Anaphes	sp.	KR888108.1
Hymenoptera	Chalcidoidea	Mymaridae	Anaphes	sp.	KR889659.1
Hymenoptera	Chalcidoidea	Mymaridae	Anaphes	sp.	KR890130.1
Hymenoptera	Chalcidoidea	Mymaridae	Anaphes	sp.	KR929160.1
Hymenoptera	Chalcidoidea	Mymaridae	Anaphes	sp.	MG499196.1
Hymenoptera	Chalcidoidea	Mymaridae	Anaphes	sp.	MG507533.1
Hymenoptera	Chalcidoidea	Mymaridae	Anaphes	sp.	MN668526.1
Hymenoptera	Chalcidoidea	Mymaridae	Anaphes	sp.	MN670057.1
Hymenoptera	Chalcidoidea	Mymaridae	Anaphes	sp.	MN671452.1
Hymenoptera	Chalcidoidea	Mymaridae	Anaphes	sp.	MN673486.1
Hymenoptera	Chalcidoidea	Mymaridae	Anaphes	sp.	MN673998.1
Hymenoptera	Chalcidoidea	Mymaridae	Anaphes	sp.	MN675726.1
Hymenoptera	Chalcidoidea	Mymaridae	Anaphes	sp.	MN675729.1
Hymenoptera	Chalcidoidea	Mymaridae	Anaphes	sp.	MN675917.1
Hymenoptera	Chalcidoidea	Mymaridae	Anaphes	sp.	MN679496.1
Hymenoptera	Chalcidoidea	Mymaridae	Anaphes	sp.	MN679498.1
Hymenoptera	Chalcidoidea	Mymaridae	Anaphes	sp.	MN679667.1
Hymenoptera	Chalcidoidea	Mymaridae	Anaphes	sp.	MN679841.1
Hymenoptera	Chalcidoidea	Mymaridae	Anaphes	sp.	MN681115.1
Hymenoptera	Chalcidoidea	Mymaridae	Anaphes	sp.	MN681703.1
Hymenoptera	Chalcidoidea	Mymaridae	Anaphes	sp.	MN683096.1
Hymenoptera	Chalcidoidea	Mymaridae	Anaphes	sp.	MZ630690.1
Hymenoptera	Chalcidoidea	Mymaridae	Camptoptera	sp.	KR885897.1
Hymenoptera	Chalcidoidea	Mymaridae	Camptoptera	sp.	KY839317.1
Hymenoptera	Chalcidoidea	Mymaridae	Camptoptera	sp.	MG352517.1
Hymenoptera	Chalcidoidea	Mymaridae	Cleruchus	sp.	MG502688.1
Hymenoptera	Chalcidoidea	Mymaridae	Cosmocomoidea	morrilli	KR782685.1

Hymenoptera	Chalcidoidea	Mymaridae	Cosmocomoidea	morrilli	KR786293.1
Hymenoptera	Chalcidoidea	Mymaridae	Cosmocomoidea	morrilli	KR786524.1
Hymenoptera	Chalcidoidea	Mymaridae	Cosmocomoidea	morrilli	KR788581.1
Hymenoptera	Chalcidoidea	Mymaridae	Cosmocomoidea	morrilli	KR791386.1
Hymenoptera	Chalcidoidea	Mymaridae	Cosmocomoidea	morrilli	KR791866.1
Hymenoptera	Chalcidoidea	Mymaridae	Cosmocomoidea	morrilli	KR793152.1
Hymenoptera	Chalcidoidea	Mymaridae	Cosmocomoidea	morrilli	KR794265.1
Hymenoptera	Chalcidoidea	Mymaridae	Cosmocomoidea	morrilli	KR795431.1
Hymenoptera	Chalcidoidea	Mymaridae	Cosmocomoidea	morrilli	KR795437.1
Hymenoptera	Chalcidoidea	Mymaridae	Cosmocomoidea	morrilli	KR797220.1
Hymenoptera	Chalcidoidea	Mymaridae	Cosmocomoidea	morrilli	KR797437.1
Hymenoptera	Chalcidoidea	Mymaridae	Cosmocomoidea	morrilli	KR798904.1
Hymenoptera	Chalcidoidea	Mymaridae	Cosmocomoidea	morrilli	KR799590.1
Hymenoptera	Chalcidoidea	Mymaridae	Cosmocomoidea	morrilli	KR800809.1
Hymenoptera	Chalcidoidea	Mymaridae	Cosmocomoidea	morrilli	KR804014.1
Hymenoptera	Chalcidoidea	Mymaridae	Cosmocomoidea	morrilli	KR805561.1
Hymenoptera	Chalcidoidea	Mymaridae	Cosmocomoidea	morrilli	KR807989.1
Hymenoptera	Chalcidoidea	Mymaridae	Cosmocomoidea	morrilli	KR874339.1
Hymenoptera	Chalcidoidea	Mymaridae	Cosmocomoidea	morrilli	KR882992.1
Hymenoptera	Chalcidoidea	Mymaridae	Cosmocomoidea	morrilli	KR902214.1
Hymenoptera	Chalcidoidea	Mymaridae	Cosmocomoidea	morrilli	MG498686.1
Hymenoptera	Chalcidoidea	Mymaridae	Cosmocomoidea	sp.	MG348835.1
Hymenoptera	Chalcidoidea	Mymaridae	Cosmocomoidea	sp.	MG356153.1
Hymenoptera	Chalcidoidea	Mymaridae	Cosmocomoidea	sp.	MW784302.1
Hymenoptera	Chalcidoidea	Mymaridae	Dicopomorpha	sp.	MG348961.1
Hymenoptera	Chalcidoidea	Mymaridae	Dicopomorpha	sp.	MG351615.1
Hymenoptera	Chalcidoidea	Mymaridae	Erythmelus	sp.	HQ930391.1
Hymenoptera	Chalcidoidea	Mymaridae	Erythmelus	sp.	JF863270.1
Hymenoptera	Chalcidoidea	Mymaridae	Erythmelus	sp.	MG346868.1
Hymenoptera	Chalcidoidea	Mymaridae	Erythmelus	sp.	MG346876.1
Hymenoptera	Chalcidoidea	Mymaridae	Erythmelus	sp.	MG347114.1
Hymenoptera	Chalcidoidea	Mymaridae	Erythmelus	sp.	MG348370.1
Hymenoptera	Chalcidoidea	Mymaridae	Erythmelus	sp.	MG351260.1
Hymenoptera	Chalcidoidea	Mymaridae	Erythmelus	sp.	MG352040.1
Hymenoptera	Chalcidoidea	Mymaridae	Erythmelus	sp.	MG352546.1
Hymenoptera	Chalcidoidea	Mymaridae	Erythmelus	sp.	MG353302.1
Hymenoptera	Chalcidoidea	Mymaridae	Erythmelus	sp.	MG355621.1
Hymenoptera	Chalcidoidea	Mymaridae	Erythmelus	sp.	MG497083.1
Hymenoptera	Chalcidoidea	Mymaridae	Erythmelus	sp.	MG497171.1
Hymenoptera	Chalcidoidea	Mymaridae	Erythmelus	sp.	MG498037.1
Hymenoptera	Chalcidoidea	Mymaridae	Erythmelus	sp.	MG499917.1
Hymenoptera	Chalcidoidea	Mymaridae	Erythmelus	sp.	MG500107.1
Hymenoptera	Chalcidoidea	Mymaridae	Erythmelus	sp.	MG504946.1
Hymenoptera	Chalcidoidea	Mymaridae	Erythmelus	sp.	MG505705.1
Hymenoptera	Chalcidoidea	Mymaridae	Gonatocerus	fuscicornis	MG501736.1
Hymenoptera	Chalcidoidea	Mymaridae	Gonatocerus	morrilli	KM561019.1
Hymenoptera	Chalcidoidea	Mymaridae	Gonatocerus	morrilli	KM567516.1
Hymenoptera	Chalcidoidea	Mymaridae	Gonatocerus	sp.	KJ443898.1
Hymenoptera	Chalcidoidea	Mymaridae	Gonatocerus	sp.	KM555534.1
Hymenoptera	Chalcidoidea	Mymaridae	Gonatocerus	sp.	KM555597.1
Hymenoptera	Chalcidoidea	Mymaridae	Gonatocerus	sp.	KM555621.1
Hymenoptera	Chalcidoidea	Mymaridae	Gonatocerus	sp.	KM555726.1
Hymenoptera	Chalcidoidea	Mymaridae	Gonatocerus	sp.	KM555727.1
Hymenoptera	Chalcidoidea	Mymaridae	Gonatocerus	sp.	KM556046.1

Hymenoptera	Chalcidoidea	Mymaridae	Gonatocerus	sp.	KM556088.1
Hymenoptera	Chalcidoidea	Mymaridae	Gonatocerus	sp.	KM556105.1
Hymenoptera	Chalcidoidea	Mymaridae	Gonatocerus	sp.	KM556228.1
Hymenoptera	Chalcidoidea	Mymaridae	Gonatocerus	sp.	KM556245.1
Hymenoptera	Chalcidoidea	Mymaridae	Gonatocerus	sp.	KM556258.1
Hymenoptera	Chalcidoidea	Mymaridae	Gonatocerus	sp.	KM556325.1
Hymenoptera	Chalcidoidea	Mymaridae	Gonatocerus	sp.	KM556363.1
Hymenoptera	Chalcidoidea	Mymaridae	Gonatocerus	sp.	KM556406.1
Hymenoptera	Chalcidoidea	Mymaridae	Gonatocerus	sp.	KM556462.1
Hymenoptera	Chalcidoidea	Mymaridae	Gonatocerus	sp.	KM556706.1
Hymenoptera	Chalcidoidea	Mymaridae	Gonatocerus	sp.	KM557074.1
Hymenoptera	Chalcidoidea	Mymaridae	Gonatocerus	sp.	KM557083.1
Hymenoptera	Chalcidoidea	Mymaridae	Gonatocerus	sp.	KM557087.1
Hymenoptera	Chalcidoidea	Mymaridae	Gonatocerus	sp.	KM557208.1
Hymenoptera	Chalcidoidea	Mymaridae	Gonatocerus	sp.	KM557238.1
Hymenoptera	Chalcidoidea	Mymaridae	Gonatocerus	sp.	KM557385.1
Hymenoptera	Chalcidoidea	Mymaridae	Gonatocerus	sp.	KM557566.1
Hymenoptera	Chalcidoidea	Mymaridae	Gonatocerus	sp.	KM557676.1
Hymenoptera	Chalcidoidea	Mymaridae	Gonatocerus	sp.	KM557828.1
Hymenoptera	Chalcidoidea	Mymaridae	Gonatocerus	sp.	KM557847.1
Hymenoptera	Chalcidoidea	Mymaridae	Gonatocerus	sp.	KM557879.1
Hymenoptera	Chalcidoidea	Mymaridae	Gonatocerus	sp.	KM558090.1
Hymenoptera	Chalcidoidea	Mymaridae	Gonatocerus	sp.	KM558145.1
Hymenoptera	Chalcidoidea	Mymaridae	Gonatocerus	sp.	KM558202.1
Hymenoptera	Chalcidoidea	Mymaridae	Gonatocerus	sp.	KM558326.1
Hymenoptera	Chalcidoidea	Mymaridae	Gonatocerus	sp.	KM558404.1
Hymenoptera	Chalcidoidea	Mymaridae	Gonatocerus	sp.	KM558605.1
Hymenoptera	Chalcidoidea	Mymaridae	Gonatocerus	sp.	KM558756.1
Hymenoptera	Chalcidoidea	Mymaridae	Gonatocerus	sp.	KM558792.1
Hymenoptera	Chalcidoidea	Mymaridae	Gonatocerus	sp.	KM558914.1
Hymenoptera	Chalcidoidea	Mymaridae	Gonatocerus	sp.	KM558983.1
Hymenoptera	Chalcidoidea	Mymaridae	Gonatocerus	sp.	KM559100.1
Hymenoptera	Chalcidoidea	Mymaridae	Gonatocerus	sp.	KM559463.1
Hymenoptera	Chalcidoidea	Mymaridae	Gonatocerus	sp.	KM559584.1
Hymenoptera	Chalcidoidea	Mymaridae	Gonatocerus	sp.	KM559615.1
Hymenoptera	Chalcidoidea	Mymaridae	Gonatocerus	sp.	KM559720.1
Hymenoptera	Chalcidoidea	Mymaridae	Gonatocerus	sp.	KM559848.1
Hymenoptera	Chalcidoidea	Mymaridae	Gonatocerus	sp.	KM560053.1
Hymenoptera	Chalcidoidea	Mymaridae	Gonatocerus	sp.	KM560261.1
Hymenoptera	Chalcidoidea	Mymaridae	Gonatocerus	sp.	KM560307.1
Hymenoptera	Chalcidoidea	Mymaridae	Gonatocerus	sp.	KM560379.1
Hymenoptera	Chalcidoidea	Mymaridae	Gonatocerus	sp.	KM560481.1
Hymenoptera	Chalcidoidea	Mymaridae	Gonatocerus	sp.	KM561275.1
Hymenoptera	Chalcidoidea	Mymaridae	Gonatocerus	sp.	KM561465.1
Hymenoptera	Chalcidoidea	Mymaridae	Gonatocerus	sp.	KM561478.1
Hymenoptera	Chalcidoidea	Mymaridae	Gonatocerus	sp.	KM561689.1
Hymenoptera	Chalcidoidea	Mymaridae	Gonatocerus	sp.	KM561752.1
Hymenoptera	Chalcidoidea	Mymaridae	Gonatocerus	sp.	KM561796.1
Hymenoptera	Chalcidoidea	Mymaridae	Gonatocerus	sp.	KM562001.1
Hymenoptera	Chalcidoidea	Mymaridae	Gonatocerus	sp.	KM562023.1
Hymenoptera	Chalcidoidea	Mymaridae	Gonatocerus	sp.	KM562043.1
Hymenoptera	Chalcidoidea	Mymaridae	Gonatocerus	sp.	KM562077.1
Hymenoptera	Chalcidoidea	Mymaridae	Gonatocerus	sp.	KM562219.1
Hymenoptera	Chalcidoidea	Mymaridae	Gonatocerus	sp.	KM562317.1

Hymenoptera	Chalcidoidea	Mymaridae	Gonatocerus	sp.	KM562318.1
Hymenoptera	Chalcidoidea	Mymaridae	Gonatocerus	sp.	KM562623.1
Hymenoptera	Chalcidoidea	Mymaridae	Gonatocerus	sp.	KM562905.1
Hymenoptera	Chalcidoidea	Mymaridae	Gonatocerus	sp.	KM563160.1
Hymenoptera	Chalcidoidea	Mymaridae	Gonatocerus	sp.	KM563366.1
Hymenoptera	Chalcidoidea	Mymaridae	Gonatocerus	sp.	KM563421.1
Hymenoptera	Chalcidoidea	Mymaridae	Gonatocerus	sp.	KM563529.1
Hymenoptera	Chalcidoidea	Mymaridae	Gonatocerus	sp.	KM563800.1
Hymenoptera	Chalcidoidea	Mymaridae	Gonatocerus	sp.	KM563897.1
Hymenoptera	Chalcidoidea	Mymaridae	Gonatocerus	sp.	KM563910.1
Hymenoptera	Chalcidoidea	Mymaridae	Gonatocerus	sp.	KM564143.1
Hymenoptera	Chalcidoidea	Mymaridae	Gonatocerus	sp.	KM564486.1
Hymenoptera	Chalcidoidea	Mymaridae	Gonatocerus	sp.	KM564612.1
Hymenoptera	Chalcidoidea	Mymaridae	Gonatocerus	sp.	KM564613.1
Hymenoptera	Chalcidoidea	Mymaridae	Gonatocerus	sp.	KM564672.1
Hymenoptera	Chalcidoidea	Mymaridae	Gonatocerus	sp.	KM564703.1
Hymenoptera	Chalcidoidea	Mymaridae	Gonatocerus	sp.	KM564827.1
Hymenoptera	Chalcidoidea	Mymaridae	Gonatocerus	sp.	KM565251.1
Hymenoptera	Chalcidoidea	Mymaridae	Gonatocerus	sp.	KM565407.1
Hymenoptera	Chalcidoidea	Mymaridae	Gonatocerus	sp.	KM565573.1
Hymenoptera	Chalcidoidea	Mymaridae	Gonatocerus	sp.	KM565704.1
Hymenoptera	Chalcidoidea	Mymaridae	Gonatocerus	sp.	KM565706.1
Hymenoptera	Chalcidoidea	Mymaridae	Gonatocerus	sp.	KM565790.1
Hymenoptera	Chalcidoidea	Mymaridae	Gonatocerus	sp.	KM565813.1
Hymenoptera	Chalcidoidea	Mymaridae	Gonatocerus	sp.	KM565900.1
Hymenoptera	Chalcidoidea	Mymaridae	Gonatocerus	sp.	KM565919.1
Hymenoptera	Chalcidoidea	Mymaridae	Gonatocerus	sp.	KM566027.1
Hymenoptera	Chalcidoidea	Mymaridae	Gonatocerus	sp.	KM566116.1
Hymenoptera	Chalcidoidea	Mymaridae	Gonatocerus	sp.	KM566186.1
Hymenoptera	Chalcidoidea	Mymaridae	Gonatocerus	sp.	KM566303.1
Hymenoptera	Chalcidoidea	Mymaridae	Gonatocerus	sp.	KM566365.1
Hymenoptera	Chalcidoidea	Mymaridae	Gonatocerus	sp.	KM566393.1
Hymenoptera	Chalcidoidea	Mymaridae	Gonatocerus	sp.	KM566404.1
Hymenoptera	Chalcidoidea	Mymaridae	Gonatocerus	sp.	KM566464.1
Hymenoptera	Chalcidoidea	Mymaridae	Gonatocerus	sp.	KM566505.1
Hymenoptera	Chalcidoidea	Mymaridae	Gonatocerus	sp.	KM566581.1
Hymenoptera	Chalcidoidea	Mymaridae	Gonatocerus	sp.	KM566694.1
Hymenoptera	Chalcidoidea	Mymaridae	Gonatocerus	sp.	KM567049.1
Hymenoptera	Chalcidoidea	Mymaridae	Gonatocerus	sp.	KM567428.1
Hymenoptera	Chalcidoidea	Mymaridae	Gonatocerus	sp.	KM567511.1
Hymenoptera	Chalcidoidea	Mymaridae	Gonatocerus	sp.	KM567618.1
Hymenoptera	Chalcidoidea	Mymaridae	Gonatocerus	sp.	KM567839.1
Hymenoptera	Chalcidoidea	Mymaridae	Gonatocerus	sp.	KM568028.1
Hymenoptera	Chalcidoidea	Mymaridae	Gonatocerus	sp.	KM568060.1
Hymenoptera	Chalcidoidea	Mymaridae	Gonatocerus	sp.	KM568172.1
Hymenoptera	Chalcidoidea	Mymaridae	Gonatocerus	sp.	KM568342.1
Hymenoptera	Chalcidoidea	Mymaridae	Gonatocerus	sp.	KM568821.1
Hymenoptera	Chalcidoidea	Mymaridae	Gonatocerus	sp.	KM568888.1
Hymenoptera	Chalcidoidea	Mymaridae	Gonatocerus	sp.	KM568996.1
Hymenoptera	Chalcidoidea	Mymaridae	Gonatocerus	sp.	KM569257.1
Hymenoptera	Chalcidoidea	Mymaridae	Gonatocerus	sp.	KM569291.1
Hymenoptera	Chalcidoidea	Mymaridae	Gonatocerus	sp.	KM997074.1
Hymenoptera	Chalcidoidea	Mymaridae	Gonatocerus	sp.	KR403264.1
Hymenoptera	Chalcidoidea	Mymaridae	Gonatocerus	sp.	KR405238.1

Hymenoptera	Chalcidoidea	Mymaridae	Gonatocerus	sp.	KR405403.1
Hymenoptera	Chalcidoidea	Mymaridae	Gonatocerus	sp.	KR405724.1
Hymenoptera	Chalcidoidea	Mymaridae	Gonatocerus	sp.	KR406068.1
Hymenoptera	Chalcidoidea	Mymaridae	Gonatocerus	sp.	KR406168.1
Hymenoptera	Chalcidoidea	Mymaridae	Gonatocerus	sp.	KR406282.1
Hymenoptera	Chalcidoidea	Mymaridae	Gonatocerus	sp.	KR406778.1
Hymenoptera	Chalcidoidea	Mymaridae	Gonatocerus	sp.	KR411571.1
Hymenoptera	Chalcidoidea	Mymaridae	Gonatocerus	sp.	KR412679.1
Hymenoptera	Chalcidoidea	Mymaridae	Gonatocerus	sp.	KR414288.1
Hymenoptera	Chalcidoidea	Mymaridae	Gonatocerus	sp.	KR416085.1
Hymenoptera	Chalcidoidea	Mymaridae	Gonatocerus	sp.	KR416552.1
Hymenoptera	Chalcidoidea	Mymaridae	Gonatocerus	sp.	KR417334.1
Hymenoptera	Chalcidoidea	Mymaridae	Gonatocerus	sp.	KR417550.1
Hymenoptera	Chalcidoidea	Mymaridae	Gonatocerus	sp.	KR419711.1
Hymenoptera	Chalcidoidea	Mymaridae	Gonatocerus	sp.	KR419964.1
Hymenoptera	Chalcidoidea	Mymaridae	Gonatocerus	sp.	KR419993.1
Hymenoptera	Chalcidoidea	Mymaridae	Gonatocerus	sp.	KR421120.1
Hymenoptera	Chalcidoidea	Mymaridae	Gonatocerus	sp.	KR421732.1
Hymenoptera	Chalcidoidea	Mymaridae	Gonatocerus	sp.	KR783397.1
Hymenoptera	Chalcidoidea	Mymaridae	Gonatocerus	sp.	KR784276.1
Hymenoptera	Chalcidoidea	Mymaridae	Gonatocerus	sp.	KR784642.1
Hymenoptera	Chalcidoidea	Mymaridae	Gonatocerus	sp.	KR784845.1
Hymenoptera	Chalcidoidea	Mymaridae	Gonatocerus	sp.	KR785061.1
Hymenoptera	Chalcidoidea	Mymaridae	Gonatocerus	sp.	KR785162.1
Hymenoptera	Chalcidoidea	Mymaridae	Gonatocerus	sp.	KR786112.1
Hymenoptera	Chalcidoidea	Mymaridae	Gonatocerus	sp.	KR786790.1
Hymenoptera	Chalcidoidea	Mymaridae	Gonatocerus	sp.	KR787073.1
Hymenoptera	Chalcidoidea	Mymaridae	Gonatocerus	sp.	KR787440.1
Hymenoptera	Chalcidoidea	Mymaridae	Gonatocerus	sp.	KR788765.1
Hymenoptera	Chalcidoidea	Mymaridae	Gonatocerus	sp.	KR790596.1
Hymenoptera	Chalcidoidea	Mymaridae	Gonatocerus	sp.	KR793801.1
Hymenoptera	Chalcidoidea	Mymaridae	Gonatocerus	sp.	KR793972.1
Hymenoptera	Chalcidoidea	Mymaridae	Gonatocerus	sp.	KR794321.1
Hymenoptera	Chalcidoidea	Mymaridae	Gonatocerus	sp.	KR796702.1
Hymenoptera	Chalcidoidea	Mymaridae	Gonatocerus	sp.	KR797725.1
Hymenoptera	Chalcidoidea	Mymaridae	Gonatocerus	sp.	KR798258.1
Hymenoptera	Chalcidoidea	Mymaridae	Gonatocerus	sp.	KR798418.1
Hymenoptera	Chalcidoidea	Mymaridae	Gonatocerus	sp.	KR798520.1
Hymenoptera	Chalcidoidea	Mymaridae	Gonatocerus	sp.	KR798946.1
Hymenoptera	Chalcidoidea	Mymaridae	Gonatocerus	sp.	KR799821.1
Hymenoptera	Chalcidoidea	Mymaridae	Gonatocerus	sp.	KR801818.1
Hymenoptera	Chalcidoidea	Mymaridae	Gonatocerus	sp.	KR801882.1
Hymenoptera	Chalcidoidea	Mymaridae	Gonatocerus	sp.	KR801935.1
Hymenoptera	Chalcidoidea	Mymaridae	Gonatocerus	sp.	KR803730.1
Hymenoptera	Chalcidoidea	Mymaridae	Gonatocerus	sp.	KR804000.1
Hymenoptera	Chalcidoidea	Mymaridae	Gonatocerus	sp.	KR804172.1
Hymenoptera	Chalcidoidea	Mymaridae	Gonatocerus	sp.	KR804296.1
Hymenoptera	Chalcidoidea	Mymaridae	Gonatocerus	sp.	KR805530.1
Hymenoptera	Chalcidoidea	Mymaridae	Gonatocerus	sp.	KR805534.1
Hymenoptera	Chalcidoidea	Mymaridae	Gonatocerus	sp.	KR806165.1
Hymenoptera	Chalcidoidea	Mymaridae	Gonatocerus	sp.	KR808905.1
Hymenoptera	Chalcidoidea	Mymaridae	Gonatocerus	sp.	KR809138.1
Hymenoptera	Chalcidoidea	Mymaridae	Gonatocerus	sp.	KR873748.1
Hymenoptera	Chalcidoidea	Mymaridae	Gonatocerus	sp.	KR874007.1

Hymenoptera	Chalcidoidea	Mymaridae	Gonatocerus	sp.	KR874487.1
Hymenoptera	Chalcidoidea	Mymaridae	Gonatocerus	sp.	KR874697.1
Hymenoptera	Chalcidoidea	Mymaridae	Gonatocerus	sp.	KR875355.1
Hymenoptera	Chalcidoidea	Mymaridae	Gonatocerus	sp.	KR875880.1
Hymenoptera	Chalcidoidea	Mymaridae	Gonatocerus	sp.	KR876114.1
Hymenoptera	Chalcidoidea	Mymaridae	Gonatocerus	sp.	KR876448.1
Hymenoptera	Chalcidoidea	Mymaridae	Gonatocerus	sp.	KR876629.1
Hymenoptera	Chalcidoidea	Mymaridae	Gonatocerus	sp.	KR878372.1
Hymenoptera	Chalcidoidea	Mymaridae	Gonatocerus	sp.	KR878891.1
Hymenoptera	Chalcidoidea	Mymaridae	Gonatocerus	sp.	KR880287.1
Hymenoptera	Chalcidoidea	Mymaridae	Gonatocerus	sp.	KR881380.1
Hymenoptera	Chalcidoidea	Mymaridae	Gonatocerus	sp.	KR881684.1
Hymenoptera	Chalcidoidea	Mymaridae	Gonatocerus	sp.	KR883541.1
Hymenoptera	Chalcidoidea	Mymaridae	Gonatocerus	sp.	KR883955.1
Hymenoptera	Chalcidoidea	Mymaridae	Gonatocerus	sp.	KR884033.1
Hymenoptera	Chalcidoidea	Mymaridae	Gonatocerus	sp.	KR884599.1
Hymenoptera	Chalcidoidea	Mymaridae	Gonatocerus	sp.	KR885127.1
Hymenoptera	Chalcidoidea	Mymaridae	Gonatocerus	sp.	KR885314.1
Hymenoptera	Chalcidoidea	Mymaridae	Gonatocerus	sp.	KR885430.1
Hymenoptera	Chalcidoidea	Mymaridae	Gonatocerus	sp.	KR887426.1
Hymenoptera	Chalcidoidea	Mymaridae	Gonatocerus	sp.	KR888139.1
Hymenoptera	Chalcidoidea	Mymaridae	Gonatocerus	sp.	KR888157.1
Hymenoptera	Chalcidoidea	Mymaridae	Gonatocerus	sp.	KR890028.1
Hymenoptera	Chalcidoidea	Mymaridae	Gonatocerus	sp.	KR890113.1
Hymenoptera	Chalcidoidea	Mymaridae	Gonatocerus	sp.	KR891477.1
Hymenoptera	Chalcidoidea	Mymaridae	Gonatocerus	sp.	KR891941.1
Hymenoptera	Chalcidoidea	Mymaridae	Gonatocerus	sp.	KR893028.1
Hymenoptera	Chalcidoidea	Mymaridae	Gonatocerus	sp.	KR893759.1
Hymenoptera	Chalcidoidea	Mymaridae	Gonatocerus	sp.	KR894300.1
Hymenoptera	Chalcidoidea	Mymaridae	Gonatocerus	sp.	KR894672.1
Hymenoptera	Chalcidoidea	Mymaridae	Gonatocerus	sp.	KR895026.1
Hymenoptera	Chalcidoidea	Mymaridae	Gonatocerus	sp.	KR896227.1
Hymenoptera	Chalcidoidea	Mymaridae	Gonatocerus	sp.	KR896746.1
Hymenoptera	Chalcidoidea	Mymaridae	Gonatocerus	sp.	KR897328.1
Hymenoptera	Chalcidoidea	Mymaridae	Gonatocerus	sp.	KR897417.1
Hymenoptera	Chalcidoidea	Mymaridae	Gonatocerus	sp.	KR897683.1
Hymenoptera	Chalcidoidea	Mymaridae	Gonatocerus	sp.	KR898160.1
Hymenoptera	Chalcidoidea	Mymaridae	Gonatocerus	sp.	KR898906.1
Hymenoptera	Chalcidoidea	Mymaridae	Gonatocerus	sp.	KR898950.1
Hymenoptera	Chalcidoidea	Mymaridae	Gonatocerus	sp.	KR899063.1
Hymenoptera	Chalcidoidea	Mymaridae	Gonatocerus	sp.	KR899210.1
Hymenoptera	Chalcidoidea	Mymaridae	Gonatocerus	sp.	KR900407.1
Hymenoptera	Chalcidoidea	Mymaridae	Gonatocerus	sp.	KR901115.1
Hymenoptera	Chalcidoidea	Mymaridae	Gonatocerus	sp.	KR901138.1
Hymenoptera	Chalcidoidea	Mymaridae	Gonatocerus	sp.	KR930001.1
Hymenoptera	Chalcidoidea	Mymaridae	Gonatocerus	sp.	KR932778.1
Hymenoptera	Chalcidoidea	Mymaridae	Gonatocerus	sp.	KR934840.1
Hymenoptera	Chalcidoidea	Mymaridae	Gonatocerus	sp.	KX535036.1
Hymenoptera	Chalcidoidea	Mymaridae	Gonatocerus	sp.	KY837929.1
Hymenoptera	Chalcidoidea	Mymaridae	Gonatocerus	sp.	KY842987.1
Hymenoptera	Chalcidoidea	Mymaridae	Gonatocerus	sp.	KY847304.1
Hymenoptera	Chalcidoidea	Mymaridae	Gonatocerus	sp.	MG350125.1
Hymenoptera	Chalcidoidea	Mymaridae	Gonatocerus	sp.	MG350326.1
Hymenoptera	Chalcidoidea	Mymaridae	Gonatocerus	sp.	MG352815.1

Hymenoptera	Chalcidoidea	Mymaridae	Gonatocerus	sp.	MG353234.1
Hymenoptera	Chalcidoidea	Mymaridae	Gonatocerus	sp.	MG497383.1
Hymenoptera	Chalcidoidea	Mymaridae	Gonatocerus	sp.	MG499294.1
Hymenoptera	Chalcidoidea	Mymaridae	Gonatocerus	sp.	MG500078.1
Hymenoptera	Chalcidoidea	Mymaridae	Gonatocerus	sp.	MG502262.1
Hymenoptera	Chalcidoidea	Mymaridae	Gonatocerus	sp.	MG904875.1
Hymenoptera	Chalcidoidea	Mymaridae	Gonatocerus	sp.	MG904884.1
Hymenoptera	Chalcidoidea	Mymaridae	Gonatocerus	sp.	MG904885.1
Hymenoptera	Chalcidoidea	Mymaridae	Gonatocerus	sp.	MW784332.1
Hymenoptera	Chalcidoidea	Mymaridae	Gonatocerus	sp.	MW784335.1
Hymenoptera	Chalcidoidea	Mymaridae	Gonatocerus	sp.	MW784337.1
Hymenoptera	Chalcidoidea	Mymaridae	Lymaenon	sp.	HM374782.1
Hymenoptera	Chalcidoidea	Mymaridae	Lymaenon	sp.	HQ929617.1
Hymenoptera	Chalcidoidea	Mymaridae	Lymaenon	sp.	HQ929618.1
Hymenoptera	Chalcidoidea	Mymaridae	Lymaenon	sp.	HQ929621.1
Hymenoptera	Chalcidoidea	Mymaridae	Lymaenon	sp.	HQ929637.1
Hymenoptera	Chalcidoidea	Mymaridae	Lymaenon	sp.	HQ930357.1
Hymenoptera	Chalcidoidea	Mymaridae	Lymaenon	sp.	JN292334.1
Hymenoptera	Chalcidoidea	Mymaridae	Lymaenon	sp.	JN292897.1
Hymenoptera	Chalcidoidea	Mymaridae	Lymaenon	sp.	JN293244.1
Hymenoptera	Chalcidoidea	Mymaridae	Lymaenon	sp.	KY829818.1
Hymenoptera	Chalcidoidea	Mymaridae	Lymaenon	sp.	KY830021.1
Hymenoptera	Chalcidoidea	Mymaridae	Lymaenon	sp.	KY830831.1
Hymenoptera	Chalcidoidea	Mymaridae	Lymaenon	sp.	KY831448.1
Hymenoptera	Chalcidoidea	Mymaridae	Lymaenon	sp.	KY833166.1
Hymenoptera	Chalcidoidea	Mymaridae	Lymaenon	sp.	KY834675.1
Hymenoptera	Chalcidoidea	Mymaridae	Lymaenon	sp.	KY834809.1
Hymenoptera	Chalcidoidea	Mymaridae	Lymaenon	sp.	KY834837.1
Hymenoptera	Chalcidoidea	Mymaridae	Lymaenon	sp.	KY835610.1
Hymenoptera	Chalcidoidea	Mymaridae	Lymaenon	sp.	KY836314.1
Hymenoptera	Chalcidoidea	Mymaridae	Lymaenon	sp.	KY837007.1
Hymenoptera	Chalcidoidea	Mymaridae	Lymaenon	sp.	KY838901.1
Hymenoptera	Chalcidoidea	Mymaridae	Lymaenon	sp.	KY839668.1
Hymenoptera	Chalcidoidea	Mymaridae	Lymaenon	sp.	KY839884.1
Hymenoptera	Chalcidoidea	Mymaridae	Lymaenon	sp.	KY839917.1
Hymenoptera	Chalcidoidea	Mymaridae	Lymaenon	sp.	KY839921.1
Hymenoptera	Chalcidoidea	Mymaridae	Lymaenon	sp.	KY841729.1
Hymenoptera	Chalcidoidea	Mymaridae	Lymaenon	sp.	KY841830.1
Hymenoptera	Chalcidoidea	Mymaridae	Lymaenon	sp.	KY842217.1
Hymenoptera	Chalcidoidea	Mymaridae	Lymaenon	sp.	KY842445.1
Hymenoptera	Chalcidoidea	Mymaridae	Lymaenon	sp.	KY843386.1
Hymenoptera	Chalcidoidea	Mymaridae	Lymaenon	sp.	KY843515.1
Hymenoptera	Chalcidoidea	Mymaridae	Lymaenon	sp.	KY844727.1
Hymenoptera	Chalcidoidea	Mymaridae	Lymaenon	sp.	KY845304.1
Hymenoptera	Chalcidoidea	Mymaridae	Lymaenon	sp.	KY845724.1
Hymenoptera	Chalcidoidea	Mymaridae	Lymaenon	sp.	KY846153.1
Hymenoptera	Chalcidoidea	Mymaridae	Lymaenon	sp.	KY846194.1
Hymenoptera	Chalcidoidea	Mymaridae	Lymaenon	sp.	KY846271.1
Hymenoptera	Chalcidoidea	Mymaridae	Lymaenon	sp.	MG346480.1
Hymenoptera	Chalcidoidea	Mymaridae	Lymaenon	sp.	MG347396.1
Hymenoptera	Chalcidoidea	Mymaridae	Lymaenon	sp.	MG347780.1
Hymenoptera	Chalcidoidea	Mymaridae	Lymaenon	sp.	MG348648.1
Hymenoptera	Chalcidoidea	Mymaridae	Lymaenon	sp.	MG348836.1
Hymenoptera	Chalcidoidea	Mymaridae	Lymaenon	sp.	MG349393.1

Hymenoptera	Chalcidoidea	Mymaridae	Lymaenon	sp.	MG349448.1
Hymenoptera	Chalcidoidea	Mymaridae	Lymaenon	sp.	MG349540.1
Hymenoptera	Chalcidoidea	Mymaridae	Lymaenon	sp.	MG349785.1
Hymenoptera	Chalcidoidea	Mymaridae	Lymaenon	sp.	MG350582.1
Hymenoptera	Chalcidoidea	Mymaridae	Lymaenon	sp.	MG351060.1
Hymenoptera	Chalcidoidea	Mymaridae	Lymaenon	sp.	MG352317.1
Hymenoptera	Chalcidoidea	Mymaridae	Lymaenon	sp.	MG352535.1
Hymenoptera	Chalcidoidea	Mymaridae	Lymaenon	sp.	MG353049.1
Hymenoptera	Chalcidoidea	Mymaridae	Lymaenon	sp.	MG353313.1
Hymenoptera	Chalcidoidea	Mymaridae	Lymaenon	sp.	MG355454.1
Hymenoptera	Chalcidoidea	Mymaridae	Lymaenon	sp.	MG497571.1
Hymenoptera	Chalcidoidea	Mymaridae	Lymaenon	sp.	MG499893.1
Hymenoptera	Chalcidoidea	Mymaridae	Lymaenon	sp.	MG500542.1
Hymenoptera	Chalcidoidea	Mymaridae	Lymaenon	sp.	MG501648.1
Hymenoptera	Chalcidoidea	Mymaridae	Lymaenon	sp.	MG503596.1
Hymenoptera	Chalcidoidea	Mymaridae	Lymaenon	sp.	MG506316.1
Hymenoptera	Chalcidoidea	Mymaridae	Lymaenon	sp.	MG506769.1
Hymenoptera	Chalcidoidea	Mymaridae	Lymaenon	sp.	MW784384.1
Hymenoptera	Chalcidoidea	Mymaridae	Mymar	sp.	KY830275.1
Hymenoptera	Chalcidoidea	Mymaridae	Mymar	sp.	KY832033.1
Hymenoptera	Chalcidoidea	Mymaridae	Mymar	sp.	KY832087.1
Hymenoptera	Chalcidoidea	Mymaridae	Mymar	sp.	KY832844.1
Hymenoptera	Chalcidoidea	Mymaridae	Mymar	sp.	KY833116.1
Hymenoptera	Chalcidoidea	Mymaridae	Mymar	sp.	KY833143.1
Hymenoptera	Chalcidoidea	Mymaridae	Mymar	sp.	KY833895.1
Hymenoptera	Chalcidoidea	Mymaridae	Mymar	sp.	KY833898.1
Hymenoptera	Chalcidoidea	Mymaridae	Mymar	sp.	KY834109.1
Hymenoptera	Chalcidoidea	Mymaridae	Mymar	sp.	KY835406.1
Hymenoptera	Chalcidoidea	Mymaridae	Mymar	sp.	KY835933.1
Hymenoptera	Chalcidoidea	Mymaridae	Mymar	sp.	KY836421.1
Hymenoptera	Chalcidoidea	Mymaridae	Mymar	sp.	KY837455.1
Hymenoptera	Chalcidoidea	Mymaridae	Mymar	sp.	KY837707.1
Hymenoptera	Chalcidoidea	Mymaridae	Mymar	sp.	KY838147.1
Hymenoptera	Chalcidoidea	Mymaridae	Mymar	sp.	KY841075.1
Hymenoptera	Chalcidoidea	Mymaridae	Mymar	sp.	KY841195.1
Hymenoptera	Chalcidoidea	Mymaridae	Mymar	sp.	KY841408.1
Hymenoptera	Chalcidoidea	Mymaridae	Mymar	sp.	KY841841.1
Hymenoptera	Chalcidoidea	Mymaridae	Mymar	sp.	KY842263.1
Hymenoptera	Chalcidoidea	Mymaridae	Mymar	sp.	KY843913.1
Hymenoptera	Chalcidoidea	Mymaridae	Mymar	sp.	KY845818.1
Hymenoptera	Chalcidoidea	Mymaridae	Mymar	sp.	KY846470.1
Hymenoptera	Chalcidoidea	Mymaridae	Mymar	sp.	MG350338.1
Hymenoptera	Chalcidoidea	Mymaridae	Mymaridae	sp.	JN289508.1
Hymenoptera	Chalcidoidea	Mymaridae	Mymaridae	sp.	KJ083610.1
Hymenoptera	Chalcidoidea	Mymaridae	Mymaridae	sp.	KJ083980.1
Hymenoptera	Chalcidoidea	Mymaridae	Mymaridae	sp.	KJ084220.1
Hymenoptera	Chalcidoidea	Mymaridae	Mymaridae	sp.	KJ084603.1
Hymenoptera	Chalcidoidea	Mymaridae	Mymaridae	sp.	KJ084730.1
Hymenoptera	Chalcidoidea	Mymaridae	Mymaridae	sp.	KJ089469.1
Hymenoptera	Chalcidoidea	Mymaridae	Mymaridae	sp.	KJ090039.1
Hymenoptera	Chalcidoidea	Mymaridae	Mymaridae	sp.	KJ090478.1
Hymenoptera	Chalcidoidea	Mymaridae	Mymaridae	sp.	KJ092436.1
Hymenoptera	Chalcidoidea	Mymaridae	Mymaridae	sp.	KJ092498.1
Hymenoptera	Chalcidoidea	Mymaridae	Mymaridae	sp.	KJ163536.1

Hymenoptera	Chalcidoidea	Mymaridae	Mymaridae	sp.	KJ165141.1
Hymenoptera	Chalcidoidea	Mymaridae	Mymaridae	sp.	KJ165150.1
Hymenoptera	Chalcidoidea	Mymaridae	Mymaridae	sp.	KJ165264.1
Hymenoptera	Chalcidoidea	Mymaridae	Mymaridae	sp.	KJ165877.1
Hymenoptera	Chalcidoidea	Mymaridae	Mymaridae	sp.	KJ167137.1
Hymenoptera	Chalcidoidea	Mymaridae	Mymaridae	sp.	KJ167781.1
Hymenoptera	Chalcidoidea	Mymaridae	Mymaridae	sp.	KJ207492.1
Hymenoptera	Chalcidoidea	Mymaridae	Mymaridae	sp.	KJ208023.1
Hymenoptera	Chalcidoidea	Mymaridae	Mymaridae	sp.	KJ208822.1
Hymenoptera	Chalcidoidea	Mymaridae	Mymaridae	sp.	KJ444564.1
Hymenoptera	Chalcidoidea	Mymaridae	Mymaridae	sp.	KJ444809.1
Hymenoptera	Chalcidoidea	Mymaridae	Mymaridae	sp.	KJ445536.1
Hymenoptera	Chalcidoidea	Mymaridae	Mymaridae	sp.	KM555373.1
Hymenoptera	Chalcidoidea	Mymaridae	Mymaridae	sp.	KM555434.1
Hymenoptera	Chalcidoidea	Mymaridae	Mymaridae	sp.	KM555485.1
Hymenoptera	Chalcidoidea	Mymaridae	Mymaridae	sp.	KM555522.1
Hymenoptera	Chalcidoidea	Mymaridae	Mymaridae	sp.	KM555576.1
Hymenoptera	Chalcidoidea	Mymaridae	Mymaridae	sp.	KM555589.1
Hymenoptera	Chalcidoidea	Mymaridae	Mymaridae	sp.	KM555613.1
Hymenoptera	Chalcidoidea	Mymaridae	Mymaridae	sp.	KM555691.1
Hymenoptera	Chalcidoidea	Mymaridae	Mymaridae	sp.	KM555732.1
Hymenoptera	Chalcidoidea	Mymaridae	Mymaridae	sp.	KM555760.1
Hymenoptera	Chalcidoidea	Mymaridae	Mymaridae	sp.	KM555761.1
Hymenoptera	Chalcidoidea	Mymaridae	Mymaridae	sp.	KM555767.1
Hymenoptera	Chalcidoidea	Mymaridae	Mymaridae	sp.	KM555777.1
Hymenoptera	Chalcidoidea	Mymaridae	Mymaridae	sp.	KM555791.1
Hymenoptera	Chalcidoidea	Mymaridae	Mymaridae	sp.	KM555816.1
Hymenoptera	Chalcidoidea	Mymaridae	Mymaridae	sp.	KM555961.1
Hymenoptera	Chalcidoidea	Mymaridae	Mymaridae	sp.	KM556098.1
Hymenoptera	Chalcidoidea	Mymaridae	Mymaridae	sp.	KM556147.1
Hymenoptera	Chalcidoidea	Mymaridae	Mymaridae	sp.	KM556152.1
Hymenoptera	Chalcidoidea	Mymaridae	Mymaridae	sp.	KM556273.1
Hymenoptera	Chalcidoidea	Mymaridae	Mymaridae	sp.	KM556345.1
Hymenoptera	Chalcidoidea	Mymaridae	Mymaridae	sp.	KM556366.1
Hymenoptera	Chalcidoidea	Mymaridae	Mymaridae	sp.	KM556378.1
Hymenoptera	Chalcidoidea	Mymaridae	Mymaridae	sp.	KM556605.1
Hymenoptera	Chalcidoidea	Mymaridae	Mymaridae	sp.	KM556633.1
Hymenoptera	Chalcidoidea	Mymaridae	Mymaridae	sp.	KM556672.1
Hymenoptera	Chalcidoidea	Mymaridae	Mymaridae	sp.	KM556692.1
Hymenoptera	Chalcidoidea	Mymaridae	Mymaridae	sp.	KM556804.1
Hymenoptera	Chalcidoidea	Mymaridae	Mymaridae	sp.	KM556821.1
Hymenoptera	Chalcidoidea	Mymaridae	Mymaridae	sp.	KM556838.1
Hymenoptera	Chalcidoidea	Mymaridae	Mymaridae	sp.	KM556865.1
Hymenoptera	Chalcidoidea	Mymaridae	Mymaridae	sp.	KM556921.1
Hymenoptera	Chalcidoidea	Mymaridae	Mymaridae	sp.	KM556927.1
Hymenoptera	Chalcidoidea	Mymaridae	Mymaridae	sp.	KM556932.1
Hymenoptera	Chalcidoidea	Mymaridae	Mymaridae	sp.	KM557023.1
Hymenoptera	Chalcidoidea	Mymaridae	Mymaridae	sp.	KM557044.1
Hymenoptera	Chalcidoidea	Mymaridae	Mymaridae	sp.	KM557066.1
Hymenoptera	Chalcidoidea	Mymaridae	Mymaridae	sp.	KM557163.1
Hymenoptera	Chalcidoidea	Mymaridae	Mymaridae	sp.	KM557177.1
Hymenoptera	Chalcidoidea	Mymaridae	Mymaridae	sp.	KM557220.1
Hymenoptera	Chalcidoidea	Mymaridae	Mymaridae	sp.	KM557282.1
Hymenoptera	Chalcidoidea	Mymaridae	Mymaridae	sp.	KM557361.1

Hymenoptera	Chalcidoidea	Mymaridae	Mymaridae	sp.	KM557366.1
Hymenoptera	Chalcidoidea	Mymaridae	Mymaridae	sp.	KM557386.1
Hymenoptera	Chalcidoidea	Mymaridae	Mymaridae	sp.	KM557394.1
Hymenoptera	Chalcidoidea	Mymaridae	Mymaridae	sp.	KM557404.1
Hymenoptera	Chalcidoidea	Mymaridae	Mymaridae	sp.	KM557463.1
Hymenoptera	Chalcidoidea	Mymaridae	Mymaridae	sp.	KM557503.1
Hymenoptera	Chalcidoidea	Mymaridae	Mymaridae	sp.	KM557538.1
Hymenoptera	Chalcidoidea	Mymaridae	Mymaridae	sp.	KM557671.1
Hymenoptera	Chalcidoidea	Mymaridae	Mymaridae	sp.	KM557685.1
Hymenoptera	Chalcidoidea	Mymaridae	Mymaridae	sp.	KM557713.1
Hymenoptera	Chalcidoidea	Mymaridae	Mymaridae	sp.	KM557775.1
Hymenoptera	Chalcidoidea	Mymaridae	Mymaridae	sp.	KM557814.1
Hymenoptera	Chalcidoidea	Mymaridae	Mymaridae	sp.	KM557876.1
Hymenoptera	Chalcidoidea	Mymaridae	Mymaridae	sp.	KM557887.1
Hymenoptera	Chalcidoidea	Mymaridae	Mymaridae	sp.	KM557927.1
Hymenoptera	Chalcidoidea	Mymaridae	Mymaridae	sp.	KM558008.1
Hymenoptera	Chalcidoidea	Mymaridae	Mymaridae	sp.	KM558149.1
Hymenoptera	Chalcidoidea	Mymaridae	Mymaridae	sp.	KM558166.1
Hymenoptera	Chalcidoidea	Mymaridae	Mymaridae	sp.	KM558226.1
Hymenoptera	Chalcidoidea	Mymaridae	Mymaridae	sp.	KM558240.1
Hymenoptera	Chalcidoidea	Mymaridae	Mymaridae	sp.	KM558315.1
Hymenoptera	Chalcidoidea	Mymaridae	Mymaridae	sp.	KM558767.1
Hymenoptera	Chalcidoidea	Mymaridae	Mymaridae	sp.	KM558772.1
Hymenoptera	Chalcidoidea	Mymaridae	Mymaridae	sp.	KM558778.1
Hymenoptera	Chalcidoidea	Mymaridae	Mymaridae	sp.	KM558975.1
Hymenoptera	Chalcidoidea	Mymaridae	Mymaridae	sp.	KM559001.1
Hymenoptera	Chalcidoidea	Mymaridae	Mymaridae	sp.	KM559009.1
Hymenoptera	Chalcidoidea	Mymaridae	Mymaridae	sp.	KM559134.1
Hymenoptera	Chalcidoidea	Mymaridae	Mymaridae	sp.	KM559147.1
Hymenoptera	Chalcidoidea	Mymaridae	Mymaridae	sp.	KM559193.1
Hymenoptera	Chalcidoidea	Mymaridae	Mymaridae	sp.	KM559229.1
Hymenoptera	Chalcidoidea	Mymaridae	Mymaridae	sp.	KM559239.1
Hymenoptera	Chalcidoidea	Mymaridae	Mymaridae	sp.	KM559243.1
Hymenoptera	Chalcidoidea	Mymaridae	Mymaridae	sp.	KM559286.1
Hymenoptera	Chalcidoidea	Mymaridae	Mymaridae	sp.	KM559335.1
Hymenoptera	Chalcidoidea	Mymaridae	Mymaridae	sp.	KM559347.1
Hymenoptera	Chalcidoidea	Mymaridae	Mymaridae	sp.	KM559417.1
Hymenoptera	Chalcidoidea	Mymaridae	Mymaridae	sp.	KM559559.1
Hymenoptera	Chalcidoidea	Mymaridae	Mymaridae	sp.	KM559595.1
Hymenoptera	Chalcidoidea	Mymaridae	Mymaridae	sp.	KM559614.1
Hymenoptera	Chalcidoidea	Mymaridae	Mymaridae	sp.	KM559643.1
Hymenoptera	Chalcidoidea	Mymaridae	Mymaridae	sp.	KM559651.1
Hymenoptera	Chalcidoidea	Mymaridae	Mymaridae	sp.	KM559680.1
Hymenoptera	Chalcidoidea	Mymaridae	Mymaridae	sp.	KM559760.1
Hymenoptera	Chalcidoidea	Mymaridae	Mymaridae	sp.	KM559818.1
Hymenoptera	Chalcidoidea	Mymaridae	Mymaridae	sp.	KM559884.1
Hymenoptera	Chalcidoidea	Mymaridae	Mymaridae	sp.	KM559993.1
Hymenoptera	Chalcidoidea	Mymaridae	Mymaridae	sp.	KM560004.1
Hymenoptera	Chalcidoidea	Mymaridae	Mymaridae	sp.	KM560040.1
Hymenoptera	Chalcidoidea	Mymaridae	Mymaridae	sp.	KM560118.1
Hymenoptera	Chalcidoidea	Mymaridae	Mymaridae	sp.	KM560130.1
Hymenoptera	Chalcidoidea	Mymaridae	Mymaridae	sp.	KM560179.1
Hymenoptera	Chalcidoidea	Mymaridae	Mymaridae	sp.	KM560188.1
Hymenoptera	Chalcidoidea	Mymaridae	Mymaridae	sp.	KM560227.1

Hymenoptera	Chalcidoidea	Mymaridae	Mymaridae	sp.	KM560234.1
Hymenoptera	Chalcidoidea	Mymaridae	Mymaridae	sp.	KM560306.1
Hymenoptera	Chalcidoidea	Mymaridae	Mymaridae	sp.	KM560319.1
Hymenoptera	Chalcidoidea	Mymaridae	Mymaridae	sp.	KM560326.1
Hymenoptera	Chalcidoidea	Mymaridae	Mymaridae	sp.	KM560384.1
Hymenoptera	Chalcidoidea	Mymaridae	Mymaridae	sp.	KM560391.1
Hymenoptera	Chalcidoidea	Mymaridae	Mymaridae	sp.	KM560433.1
Hymenoptera	Chalcidoidea	Mymaridae	Mymaridae	sp.	KM560435.1
Hymenoptera	Chalcidoidea	Mymaridae	Mymaridae	sp.	KM560516.1
Hymenoptera	Chalcidoidea	Mymaridae	Mymaridae	sp.	KM560578.1
Hymenoptera	Chalcidoidea	Mymaridae	Mymaridae	sp.	KM560581.1
Hymenoptera	Chalcidoidea	Mymaridae	Mymaridae	sp.	KM560586.1
Hymenoptera	Chalcidoidea	Mymaridae	Mymaridae	sp.	KM560604.1
Hymenoptera	Chalcidoidea	Mymaridae	Mymaridae	sp.	KM560713.1
Hymenoptera	Chalcidoidea	Mymaridae	Mymaridae	sp.	KM560776.1
Hymenoptera	Chalcidoidea	Mymaridae	Mymaridae	sp.	KM560788.1
Hymenoptera	Chalcidoidea	Mymaridae	Mymaridae	sp.	KM560900.1
Hymenoptera	Chalcidoidea	Mymaridae	Mymaridae	sp.	KM561010.1
Hymenoptera	Chalcidoidea	Mymaridae	Mymaridae	sp.	KM561077.1
Hymenoptera	Chalcidoidea	Mymaridae	Mymaridae	sp.	KM561131.1
Hymenoptera	Chalcidoidea	Mymaridae	Mymaridae	sp.	KM561232.1
Hymenoptera	Chalcidoidea	Mymaridae	Mymaridae	sp.	KM561508.1
Hymenoptera	Chalcidoidea	Mymaridae	Mymaridae	sp.	KM561522.1
Hymenoptera	Chalcidoidea	Mymaridae	Mymaridae	sp.	KM561544.1
Hymenoptera	Chalcidoidea	Mymaridae	Mymaridae	sp.	KM561608.1
Hymenoptera	Chalcidoidea	Mymaridae	Mymaridae	sp.	KM561639.1
Hymenoptera	Chalcidoidea	Mymaridae	Mymaridae	sp.	KM561644.1
Hymenoptera	Chalcidoidea	Mymaridae	Mymaridae	sp.	KM561703.1
Hymenoptera	Chalcidoidea	Mymaridae	Mymaridae	sp.	KM561759.1
Hymenoptera	Chalcidoidea	Mymaridae	Mymaridae	sp.	KM561874.1
Hymenoptera	Chalcidoidea	Mymaridae	Mymaridae	sp.	KM561911.1
Hymenoptera	Chalcidoidea	Mymaridae	Mymaridae	sp.	KM561915.1
Hymenoptera	Chalcidoidea	Mymaridae	Mymaridae	sp.	KM561947.1
Hymenoptera	Chalcidoidea	Mymaridae	Mymaridae	sp.	KM561979.1
Hymenoptera	Chalcidoidea	Mymaridae	Mymaridae	sp.	KM562102.1
Hymenoptera	Chalcidoidea	Mymaridae	Mymaridae	sp.	KM562107.1
Hymenoptera	Chalcidoidea	Mymaridae	Mymaridae	sp.	KM562114.1
Hymenoptera	Chalcidoidea	Mymaridae	Mymaridae	sp.	KM562144.1
Hymenoptera	Chalcidoidea	Mymaridae	Mymaridae	sp.	KM562185.1
Hymenoptera	Chalcidoidea	Mymaridae	Mymaridae	sp.	KM562191.1
Hymenoptera	Chalcidoidea	Mymaridae	Mymaridae	sp.	KM562216.1
Hymenoptera	Chalcidoidea	Mymaridae	Mymaridae	sp.	KM562252.1
Hymenoptera	Chalcidoidea	Mymaridae	Mymaridae	sp.	KM562286.1
Hymenoptera	Chalcidoidea	Mymaridae	Mymaridae	sp.	KM562339.1
Hymenoptera	Chalcidoidea	Mymaridae	Mymaridae	sp.	KM562425.1
Hymenoptera	Chalcidoidea	Mymaridae	Mymaridae	sp.	KM562426.1
Hymenoptera	Chalcidoidea	Mymaridae	Mymaridae	sp.	KM562491.1
Hymenoptera	Chalcidoidea	Mymaridae	Mymaridae	sp.	KM562496.1
Hymenoptera	Chalcidoidea	Mymaridae	Mymaridae	sp.	KM562504.1
Hymenoptera	Chalcidoidea	Mymaridae	Mymaridae	sp.	KM562640.1
Hymenoptera	Chalcidoidea	Mymaridae	Mymaridae	sp.	KM562750.1
Hymenoptera	Chalcidoidea	Mymaridae	Mymaridae	sp.	KM562776.1
Hymenoptera	Chalcidoidea	Mymaridae	Mymaridae	sp.	KM562867.1
Hymenoptera	Chalcidoidea	Mymaridae	Mymaridae	sp.	KM562918.1

Hymenoptera	Chalcidoidea	Mymaridae	Mymaridae	sp.	KM563004.1
Hymenoptera	Chalcidoidea	Mymaridae	Mymaridae	sp.	KM563026.1
Hymenoptera	Chalcidoidea	Mymaridae	Mymaridae	sp.	KM563042.1
Hymenoptera	Chalcidoidea	Mymaridae	Mymaridae	sp.	KM563055.1
Hymenoptera	Chalcidoidea	Mymaridae	Mymaridae	sp.	KM563122.1
Hymenoptera	Chalcidoidea	Mymaridae	Mymaridae	sp.	KM563209.1
Hymenoptera	Chalcidoidea	Mymaridae	Mymaridae	sp.	KM563236.1
Hymenoptera	Chalcidoidea	Mymaridae	Mymaridae	sp.	KM563253.1
Hymenoptera	Chalcidoidea	Mymaridae	Mymaridae	sp.	KM563266.1
Hymenoptera	Chalcidoidea	Mymaridae	Mymaridae	sp.	KM563277.1
Hymenoptera	Chalcidoidea	Mymaridae	Mymaridae	sp.	KM563381.1
Hymenoptera	Chalcidoidea	Mymaridae	Mymaridae	sp.	KM563483.1
Hymenoptera	Chalcidoidea	Mymaridae	Mymaridae	sp.	KM563585.1
Hymenoptera	Chalcidoidea	Mymaridae	Mymaridae	sp.	KM563607.1
Hymenoptera	Chalcidoidea	Mymaridae	Mymaridae	sp.	KM563668.1
Hymenoptera	Chalcidoidea	Mymaridae	Mymaridae	sp.	KM563870.1
Hymenoptera	Chalcidoidea	Mymaridae	Mymaridae	sp.	KM564000.1
Hymenoptera	Chalcidoidea	Mymaridae	Mymaridae	sp.	KM564030.1
Hymenoptera	Chalcidoidea	Mymaridae	Mymaridae	sp.	KM564087.1
Hymenoptera	Chalcidoidea	Mymaridae	Mymaridae	sp.	KM564168.1
Hymenoptera	Chalcidoidea	Mymaridae	Mymaridae	sp.	KM564194.1
Hymenoptera	Chalcidoidea	Mymaridae	Mymaridae	sp.	KM564207.1
Hymenoptera	Chalcidoidea	Mymaridae	Mymaridae	sp.	KM564358.1
Hymenoptera	Chalcidoidea	Mymaridae	Mymaridae	sp.	KM564467.1
Hymenoptera	Chalcidoidea	Mymaridae	Mymaridae	sp.	KM564480.1
Hymenoptera	Chalcidoidea	Mymaridae	Mymaridae	sp.	KM564526.1
Hymenoptera	Chalcidoidea	Mymaridae	Mymaridae	sp.	KM564621.1
Hymenoptera	Chalcidoidea	Mymaridae	Mymaridae	sp.	KM564698.1
Hymenoptera	Chalcidoidea	Mymaridae	Mymaridae	sp.	KM564837.1
Hymenoptera	Chalcidoidea	Mymaridae	Mymaridae	sp.	KM564868.1
Hymenoptera	Chalcidoidea	Mymaridae	Mymaridae	sp.	KM564887.1
Hymenoptera	Chalcidoidea	Mymaridae	Mymaridae	sp.	KM564892.1
Hymenoptera	Chalcidoidea	Mymaridae	Mymaridae	sp.	KM565071.1
Hymenoptera	Chalcidoidea	Mymaridae	Mymaridae	sp.	KM565107.1
Hymenoptera	Chalcidoidea	Mymaridae	Mymaridae	sp.	KM565156.1
Hymenoptera	Chalcidoidea	Mymaridae	Mymaridae	sp.	KM565164.1
Hymenoptera	Chalcidoidea	Mymaridae	Mymaridae	sp.	KM565220.1
Hymenoptera	Chalcidoidea	Mymaridae	Mymaridae	sp.	KM565353.1
Hymenoptera	Chalcidoidea	Mymaridae	Mymaridae	sp.	KM565362.1
Hymenoptera	Chalcidoidea	Mymaridae	Mymaridae	sp.	KM565464.1
Hymenoptera	Chalcidoidea	Mymaridae	Mymaridae	sp.	KM565518.1
Hymenoptera	Chalcidoidea	Mymaridae	Mymaridae	sp.	KM565559.1
Hymenoptera	Chalcidoidea	Mymaridae	Mymaridae	sp.	KM565585.1
Hymenoptera	Chalcidoidea	Mymaridae	Mymaridae	sp.	KM565608.1
Hymenoptera	Chalcidoidea	Mymaridae	Mymaridae	sp.	KM565649.1
Hymenoptera	Chalcidoidea	Mymaridae	Mymaridae	sp.	KM565653.1
Hymenoptera	Chalcidoidea	Mymaridae	Mymaridae	sp.	KM565712.1
Hymenoptera	Chalcidoidea	Mymaridae	Mymaridae	sp.	KM565714.1
Hymenoptera	Chalcidoidea	Mymaridae	Mymaridae	sp.	KM565811.1
Hymenoptera	Chalcidoidea	Mymaridae	Mymaridae	sp.	KM565845.1
Hymenoptera	Chalcidoidea	Mymaridae	Mymaridae	sp.	KM565913.1
Hymenoptera	Chalcidoidea	Mymaridae	Mymaridae	sp.	KM565945.1
Hymenoptera	Chalcidoidea	Mymaridae	Mymaridae	sp.	KM565982.1
Hymenoptera	Chalcidoidea	Mymaridae	Mymaridae	sp.	KM566003.1

Hymenoptera	Chalcidoidea	Mymaridae	Mymaridae	sp.	KM566020.1
Hymenoptera	Chalcidoidea	Mymaridae	Mymaridae	sp.	KM566038.1
Hymenoptera	Chalcidoidea	Mymaridae	Mymaridae	sp.	KM566087.1
Hymenoptera	Chalcidoidea	Mymaridae	Mymaridae	sp.	KM566223.1
Hymenoptera	Chalcidoidea	Mymaridae	Mymaridae	sp.	KM566286.1
Hymenoptera	Chalcidoidea	Mymaridae	Mymaridae	sp.	KM566330.1
Hymenoptera	Chalcidoidea	Mymaridae	Mymaridae	sp.	KM566370.1
Hymenoptera	Chalcidoidea	Mymaridae	Mymaridae	sp.	KM566382.1
Hymenoptera	Chalcidoidea	Mymaridae	Mymaridae	sp.	KM566388.1
Hymenoptera	Chalcidoidea	Mymaridae	Mymaridae	sp.	KM566410.1
Hymenoptera	Chalcidoidea	Mymaridae	Mymaridae	sp.	KM566668.1
Hymenoptera	Chalcidoidea	Mymaridae	Mymaridae	sp.	KM566711.1
Hymenoptera	Chalcidoidea	Mymaridae	Mymaridae	sp.	KM566750.1
Hymenoptera	Chalcidoidea	Mymaridae	Mymaridae	sp.	KM567022.1
Hymenoptera	Chalcidoidea	Mymaridae	Mymaridae	sp.	KM567048.1
Hymenoptera	Chalcidoidea	Mymaridae	Mymaridae	sp.	KM567076.1
Hymenoptera	Chalcidoidea	Mymaridae	Mymaridae	sp.	KM567086.1
Hymenoptera	Chalcidoidea	Mymaridae	Mymaridae	sp.	KM567127.1
Hymenoptera	Chalcidoidea	Mymaridae	Mymaridae	sp.	KM567208.1
Hymenoptera	Chalcidoidea	Mymaridae	Mymaridae	sp.	KM567247.1
Hymenoptera	Chalcidoidea	Mymaridae	Mymaridae	sp.	KM567337.1
Hymenoptera	Chalcidoidea	Mymaridae	Mymaridae	sp.	KM567343.1
Hymenoptera	Chalcidoidea	Mymaridae	Mymaridae	sp.	KM567406.1
Hymenoptera	Chalcidoidea	Mymaridae	Mymaridae	sp.	KM567477.1
Hymenoptera	Chalcidoidea	Mymaridae	Mymaridae	sp.	KM567483.1
Hymenoptera	Chalcidoidea	Mymaridae	Mymaridae	sp.	KM567492.1
Hymenoptera	Chalcidoidea	Mymaridae	Mymaridae	sp.	KM567531.1
Hymenoptera	Chalcidoidea	Mymaridae	Mymaridae	sp.	KM567562.1
Hymenoptera	Chalcidoidea	Mymaridae	Mymaridae	sp.	KM567592.1
Hymenoptera	Chalcidoidea	Mymaridae	Mymaridae	sp.	KM567838.1
Hymenoptera	Chalcidoidea	Mymaridae	Mymaridae	sp.	KM567908.1
Hymenoptera	Chalcidoidea	Mymaridae	Mymaridae	sp.	KM568079.1
Hymenoptera	Chalcidoidea	Mymaridae	Mymaridae	sp.	KM568142.1
Hymenoptera	Chalcidoidea	Mymaridae	Mymaridae	sp.	KM568147.1
Hymenoptera	Chalcidoidea	Mymaridae	Mymaridae	sp.	KM568153.1
Hymenoptera	Chalcidoidea	Mymaridae	Mymaridae	sp.	KM568227.1
Hymenoptera	Chalcidoidea	Mymaridae	Mymaridae	sp.	KM568303.1
Hymenoptera	Chalcidoidea	Mymaridae	Mymaridae	sp.	KM568322.1
Hymenoptera	Chalcidoidea	Mymaridae	Mymaridae	sp.	KM568392.1
Hymenoptera	Chalcidoidea	Mymaridae	Mymaridae	sp.	KM568696.1
Hymenoptera	Chalcidoidea	Mymaridae	Mymaridae	sp.	KM568705.1
Hymenoptera	Chalcidoidea	Mymaridae	Mymaridae	sp.	KM568721.1
Hymenoptera	Chalcidoidea	Mymaridae	Mymaridae	sp.	KM568753.1
Hymenoptera	Chalcidoidea	Mymaridae	Mymaridae	sp.	KM568805.1
Hymenoptera	Chalcidoidea	Mymaridae	Mymaridae	sp.	KM568854.1
Hymenoptera	Chalcidoidea	Mymaridae	Mymaridae	sp.	KM568921.1
Hymenoptera	Chalcidoidea	Mymaridae	Mymaridae	sp.	KM568975.1
Hymenoptera	Chalcidoidea	Mymaridae	Mymaridae	sp.	KM569054.1
Hymenoptera	Chalcidoidea	Mymaridae	Mymaridae	sp.	KM569078.1
Hymenoptera	Chalcidoidea	Mymaridae	Mymaridae	sp.	KM569108.1
Hymenoptera	Chalcidoidea	Mymaridae	Mymaridae	sp.	KM569160.1
Hymenoptera	Chalcidoidea	Mymaridae	Mymaridae	sp.	KM569171.1
Hymenoptera	Chalcidoidea	Mymaridae	Mymaridae	sp.	KM569181.1
Hymenoptera	Chalcidoidea	Mymaridae	Mymaridae	sp.	KM569311.1

Hymenoptera	Chalcidoidea	Mymaridae	Mymaridae	sp.	KM569355.1
Hymenoptera	Chalcidoidea	Mymaridae	Mymaridae	sp.	KM569385.1
Hymenoptera	Chalcidoidea	Mymaridae	Mymaridae	sp.	KM569398.1
Hymenoptera	Chalcidoidea	Mymaridae	Mymaridae	sp.	KM569474.1
Hymenoptera	Chalcidoidea	Mymaridae	Mymaridae	sp.	KM569514.1
Hymenoptera	Chalcidoidea	Mymaridae	Mymaridae	sp.	KM569543.1
Hymenoptera	Chalcidoidea	Mymaridae	Mymaridae	sp.	KM569572.1
Hymenoptera	Chalcidoidea	Mymaridae	Mymaridae	sp.	KM569620.1
Hymenoptera	Chalcidoidea	Mymaridae	Mymaridae	sp.	KR401551.1
Hymenoptera	Chalcidoidea	Mymaridae	Mymaridae	sp.	KR401702.1
Hymenoptera	Chalcidoidea	Mymaridae	Mymaridae	sp.	KR402142.1
Hymenoptera	Chalcidoidea	Mymaridae	Mymaridae	sp.	KR402372.1
Hymenoptera	Chalcidoidea	Mymaridae	Mymaridae	sp.	KR402439.1
Hymenoptera	Chalcidoidea	Mymaridae	Mymaridae	sp.	KR402597.1
Hymenoptera	Chalcidoidea	Mymaridae	Mymaridae	sp.	KR402638.1
Hymenoptera	Chalcidoidea	Mymaridae	Mymaridae	sp.	KR403094.1
Hymenoptera	Chalcidoidea	Mymaridae	Mymaridae	sp.	KR403318.1
Hymenoptera	Chalcidoidea	Mymaridae	Mymaridae	sp.	KR403409.1
Hymenoptera	Chalcidoidea	Mymaridae	Mymaridae	sp.	KR403479.1
Hymenoptera	Chalcidoidea	Mymaridae	Mymaridae	sp.	KR403536.1
Hymenoptera	Chalcidoidea	Mymaridae	Mymaridae	sp.	KR403626.1
Hymenoptera	Chalcidoidea	Mymaridae	Mymaridae	sp.	KR404811.1
Hymenoptera	Chalcidoidea	Mymaridae	Mymaridae	sp.	KR405163.1
Hymenoptera	Chalcidoidea	Mymaridae	Mymaridae	sp.	KR405279.1
Hymenoptera	Chalcidoidea	Mymaridae	Mymaridae	sp.	KR405380.1
Hymenoptera	Chalcidoidea	Mymaridae	Mymaridae	sp.	KR405960.1
Hymenoptera	Chalcidoidea	Mymaridae	Mymaridae	sp.	KR406815.1
Hymenoptera	Chalcidoidea	Mymaridae	Mymaridae	sp.	KR407514.1
Hymenoptera	Chalcidoidea	Mymaridae	Mymaridae	sp.	KR407539.1
Hymenoptera	Chalcidoidea	Mymaridae	Mymaridae	sp.	KR407701.1
Hymenoptera	Chalcidoidea	Mymaridae	Mymaridae	sp.	KR408417.1
Hymenoptera	Chalcidoidea	Mymaridae	Mymaridae	sp.	KR408420.1
Hymenoptera	Chalcidoidea	Mymaridae	Mymaridae	sp.	KR409087.1
Hymenoptera	Chalcidoidea	Mymaridae	Mymaridae	sp.	KR409137.1
Hymenoptera	Chalcidoidea	Mymaridae	Mymaridae	sp.	KR409820.1
Hymenoptera	Chalcidoidea	Mymaridae	Mymaridae	sp.	KR410205.1
Hymenoptera	Chalcidoidea	Mymaridae	Mymaridae	sp.	KR410232.1
Hymenoptera	Chalcidoidea	Mymaridae	Mymaridae	sp.	KR411481.1
Hymenoptera	Chalcidoidea	Mymaridae	Mymaridae	sp.	KR411817.1
Hymenoptera	Chalcidoidea	Mymaridae	Mymaridae	sp.	KR412712.1
Hymenoptera	Chalcidoidea	Mymaridae	Mymaridae	sp.	KR412750.1
Hymenoptera	Chalcidoidea	Mymaridae	Mymaridae	sp.	KR412824.1
Hymenoptera	Chalcidoidea	Mymaridae	Mymaridae	sp.	KR413403.1
Hymenoptera	Chalcidoidea	Mymaridae	Mymaridae	sp.	KR413652.1
Hymenoptera	Chalcidoidea	Mymaridae	Mymaridae	sp.	KR413674.1
Hymenoptera	Chalcidoidea	Mymaridae	Mymaridae	sp.	KR414011.1
Hymenoptera	Chalcidoidea	Mymaridae	Mymaridae	sp.	KR414186.1
Hymenoptera	Chalcidoidea	Mymaridae	Mymaridae	sp.	KR415062.1
Hymenoptera	Chalcidoidea	Mymaridae	Mymaridae	sp.	KR415788.1
Hymenoptera	Chalcidoidea	Mymaridae	Mymaridae	sp.	KR415826.1
Hymenoptera	Chalcidoidea	Mymaridae	Mymaridae	sp.	KR416143.1
Hymenoptera	Chalcidoidea	Mymaridae	Mymaridae	sp.	KR416144.1
Hymenoptera	Chalcidoidea	Mymaridae	Mymaridae	sp.	KR416184.1
Hymenoptera	Chalcidoidea	Mymaridae	Mymaridae	sp.	KR417014.1

Hymenoptera	Chalcidoidea	Mymaridae	Mymaridae	sp.	KR417328.1
Hymenoptera	Chalcidoidea	Mymaridae	Mymaridae	sp.	KR417432.1
Hymenoptera	Chalcidoidea	Mymaridae	Mymaridae	sp.	KR417604.1
Hymenoptera	Chalcidoidea	Mymaridae	Mymaridae	sp.	KR418090.1
Hymenoptera	Chalcidoidea	Mymaridae	Mymaridae	sp.	KR418653.1
Hymenoptera	Chalcidoidea	Mymaridae	Mymaridae	sp.	KR418682.1
Hymenoptera	Chalcidoidea	Mymaridae	Mymaridae	sp.	KR418972.1
Hymenoptera	Chalcidoidea	Mymaridae	Mymaridae	sp.	KR419459.1
Hymenoptera	Chalcidoidea	Mymaridae	Mymaridae	sp.	KR420403.1
Hymenoptera	Chalcidoidea	Mymaridae	Mymaridae	sp.	KR420601.1
Hymenoptera	Chalcidoidea	Mymaridae	Mymaridae	sp.	KR420616.1
Hymenoptera	Chalcidoidea	Mymaridae	Mymaridae	sp.	KR420830.1
Hymenoptera	Chalcidoidea	Mymaridae	Mymaridae	sp.	KR421238.1
Hymenoptera	Chalcidoidea	Mymaridae	Mymaridae	sp.	KR421643.1
Hymenoptera	Chalcidoidea	Mymaridae	Mymaridae	sp.	KR782803.1
Hymenoptera	Chalcidoidea	Mymaridae	Mymaridae	sp.	KR782905.1
Hymenoptera	Chalcidoidea	Mymaridae	Mymaridae	sp.	KR782997.1
Hymenoptera	Chalcidoidea	Mymaridae	Mymaridae	sp.	KR783214.1
Hymenoptera	Chalcidoidea	Mymaridae	Mymaridae	sp.	KR783314.1
Hymenoptera	Chalcidoidea	Mymaridae	Mymaridae	sp.	KR784041.1
Hymenoptera	Chalcidoidea	Mymaridae	Mymaridae	sp.	KR785277.1
Hymenoptera	Chalcidoidea	Mymaridae	Mymaridae	sp.	KR786910.1
Hymenoptera	Chalcidoidea	Mymaridae	Mymaridae	sp.	KR787119.1
Hymenoptera	Chalcidoidea	Mymaridae	Mymaridae	sp.	KR787458.1
Hymenoptera	Chalcidoidea	Mymaridae	Mymaridae	sp.	KR787809.1
Hymenoptera	Chalcidoidea	Mymaridae	Mymaridae	sp.	KR788128.1
Hymenoptera	Chalcidoidea	Mymaridae	Mymaridae	sp.	KR788188.1
Hymenoptera	Chalcidoidea	Mymaridae	Mymaridae	sp.	KR788210.1
Hymenoptera	Chalcidoidea	Mymaridae	Mymaridae	sp.	KR788653.1
Hymenoptera	Chalcidoidea	Mymaridae	Mymaridae	sp.	KR788754.1
Hymenoptera	Chalcidoidea	Mymaridae	Mymaridae	sp.	KR789631.1
Hymenoptera	Chalcidoidea	Mymaridae	Mymaridae	sp.	KR790498.1
Hymenoptera	Chalcidoidea	Mymaridae	Mymaridae	sp.	KR790785.1
Hymenoptera	Chalcidoidea	Mymaridae	Mymaridae	sp.	KR791409.1
Hymenoptera	Chalcidoidea	Mymaridae	Mymaridae	sp.	KR792099.1
Hymenoptera	Chalcidoidea	Mymaridae	Mymaridae	sp.	KR794217.1
Hymenoptera	Chalcidoidea	Mymaridae	Mymaridae	sp.	KR794606.1
Hymenoptera	Chalcidoidea	Mymaridae	Mymaridae	sp.	KR794713.1
Hymenoptera	Chalcidoidea	Mymaridae	Mymaridae	sp.	KR795156.1
Hymenoptera	Chalcidoidea	Mymaridae	Mymaridae	sp.	KR795404.1
Hymenoptera	Chalcidoidea	Mymaridae	Mymaridae	sp.	KR797080.1
Hymenoptera	Chalcidoidea	Mymaridae	Mymaridae	sp.	KR798179.1
Hymenoptera	Chalcidoidea	Mymaridae	Mymaridae	sp.	KR798636.1
Hymenoptera	Chalcidoidea	Mymaridae	Mymaridae	sp.	KR799788.1
Hymenoptera	Chalcidoidea	Mymaridae	Mymaridae	sp.	KR799791.1
Hymenoptera	Chalcidoidea	Mymaridae	Mymaridae	sp.	KR800108.1
Hymenoptera	Chalcidoidea	Mymaridae	Mymaridae	sp.	KR801283.1
Hymenoptera	Chalcidoidea	Mymaridae	Mymaridae	sp.	KR801708.1
Hymenoptera	Chalcidoidea	Mymaridae	Mymaridae	sp.	KR802156.1
Hymenoptera	Chalcidoidea	Mymaridae	Mymaridae	sp.	KR803401.1
Hymenoptera	Chalcidoidea	Mymaridae	Mymaridae	sp.	KR803819.1
Hymenoptera	Chalcidoidea	Mymaridae	Mymaridae	sp.	KR804342.1
Hymenoptera	Chalcidoidea	Mymaridae	Mymaridae	sp.	KR804902.1
Hymenoptera	Chalcidoidea	Mymaridae	Mymaridae	sp.	KR805877.1

Hymenoptera	Chalcidoidea	Mymaridae	Mymaridae	sp.	KR805909.1
Hymenoptera	Chalcidoidea	Mymaridae	Mymaridae	sp.	KR806034.1
Hymenoptera	Chalcidoidea	Mymaridae	Mymaridae	sp.	KR807403.1
Hymenoptera	Chalcidoidea	Mymaridae	Mymaridae	sp.	KR807801.1
Hymenoptera	Chalcidoidea	Mymaridae	Mymaridae	sp.	KR808733.1
Hymenoptera	Chalcidoidea	Mymaridae	Mymaridae	sp.	KR809347.1
Hymenoptera	Chalcidoidea	Mymaridae	Mymaridae	sp.	KR873514.1
Hymenoptera	Chalcidoidea	Mymaridae	Mymaridae	sp.	KR873540.1
Hymenoptera	Chalcidoidea	Mymaridae	Mymaridae	sp.	KR874375.1
Hymenoptera	Chalcidoidea	Mymaridae	Mymaridae	sp.	KR874486.1
Hymenoptera	Chalcidoidea	Mymaridae	Mymaridae	sp.	KR874911.1
Hymenoptera	Chalcidoidea	Mymaridae	Mymaridae	sp.	KR875506.1
Hymenoptera	Chalcidoidea	Mymaridae	Mymaridae	sp.	KR875624.1
Hymenoptera	Chalcidoidea	Mymaridae	Mymaridae	sp.	KR876894.1
Hymenoptera	Chalcidoidea	Mymaridae	Mymaridae	sp.	KR877025.1
Hymenoptera	Chalcidoidea	Mymaridae	Mymaridae	sp.	KR877812.1
Hymenoptera	Chalcidoidea	Mymaridae	Mymaridae	sp.	KR879018.1
Hymenoptera	Chalcidoidea	Mymaridae	Mymaridae	sp.	KR879324.1
Hymenoptera	Chalcidoidea	Mymaridae	Mymaridae	sp.	KR880045.1
Hymenoptera	Chalcidoidea	Mymaridae	Mymaridae	sp.	KR882076.1
Hymenoptera	Chalcidoidea	Mymaridae	Mymaridae	sp.	KR882273.1
Hymenoptera	Chalcidoidea	Mymaridae	Mymaridae	sp.	KR882707.1
Hymenoptera	Chalcidoidea	Mymaridae	Mymaridae	sp.	KR882858.1
Hymenoptera	Chalcidoidea	Mymaridae	Mymaridae	sp.	KR882874.1
Hymenoptera	Chalcidoidea	Mymaridae	Mymaridae	sp.	KR883495.1
Hymenoptera	Chalcidoidea	Mymaridae	Mymaridae	sp.	KR883825.1
Hymenoptera	Chalcidoidea	Mymaridae	Mymaridae	sp.	KR883827.1
Hymenoptera	Chalcidoidea	Mymaridae	Mymaridae	sp.	KR884852.1
Hymenoptera	Chalcidoidea	Mymaridae	Mymaridae	sp.	KR885365.1
Hymenoptera	Chalcidoidea	Mymaridae	Mymaridae	sp.	KR885383.1
Hymenoptera	Chalcidoidea	Mymaridae	Mymaridae	sp.	KR885931.1
Hymenoptera	Chalcidoidea	Mymaridae	Mymaridae	sp.	KR886155.1
Hymenoptera	Chalcidoidea	Mymaridae	Mymaridae	sp.	KR886816.1
Hymenoptera	Chalcidoidea	Mymaridae	Mymaridae	sp.	KR886920.1
Hymenoptera	Chalcidoidea	Mymaridae	Mymaridae	sp.	KR887185.1
Hymenoptera	Chalcidoidea	Mymaridae	Mymaridae	sp.	KR887968.1
Hymenoptera	Chalcidoidea	Mymaridae	Mymaridae	sp.	KR888090.1
Hymenoptera	Chalcidoidea	Mymaridae	Mymaridae	sp.	KR888762.1
Hymenoptera	Chalcidoidea	Mymaridae	Mymaridae	sp.	KR889198.1
Hymenoptera	Chalcidoidea	Mymaridae	Mymaridae	sp.	KR889552.1
Hymenoptera	Chalcidoidea	Mymaridae	Mymaridae	sp.	KR890371.1
Hymenoptera	Chalcidoidea	Mymaridae	Mymaridae	sp.	KR891108.1
Hymenoptera	Chalcidoidea	Mymaridae	Mymaridae	sp.	KR891367.1
Hymenoptera	Chalcidoidea	Mymaridae	Mymaridae	sp.	KR891923.1
Hymenoptera	Chalcidoidea	Mymaridae	Mymaridae	sp.	KR892208.1
Hymenoptera	Chalcidoidea	Mymaridae	Mymaridae	sp.	KR892522.1
Hymenoptera	Chalcidoidea	Mymaridae	Mymaridae	sp.	KR892572.1
Hymenoptera	Chalcidoidea	Mymaridae	Mymaridae	sp.	KR892629.1
Hymenoptera	Chalcidoidea	Mymaridae	Mymaridae	sp.	KR893116.1
Hymenoptera	Chalcidoidea	Mymaridae	Mymaridae	sp.	KR893282.1
Hymenoptera	Chalcidoidea	Mymaridae	Mymaridae	sp.	KR893605.1
Hymenoptera	Chalcidoidea	Mymaridae	Mymaridae	sp.	KR893788.1
Hymenoptera	Chalcidoidea	Mymaridae	Mymaridae	sp.	KR894022.1
Hymenoptera	Chalcidoidea	Mymaridae	Mymaridae	sp.	KR894103.1

Hymenoptera	Chalcidoidea	Mymaridae	Mymaridae	sp.	KR894140.1
Hymenoptera	Chalcidoidea	Mymaridae	Mymaridae	sp.	KR894645.1
Hymenoptera	Chalcidoidea	Mymaridae	Mymaridae	sp.	KR894755.1
Hymenoptera	Chalcidoidea	Mymaridae	Mymaridae	sp.	KR895551.1
Hymenoptera	Chalcidoidea	Mymaridae	Mymaridae	sp.	KR902311.1
Hymenoptera	Chalcidoidea	Mymaridae	Mymaridae	sp.	KR925633.1
Hymenoptera	Chalcidoidea	Mymaridae	Mymaridae	sp.	KR928880.1
Hymenoptera	Chalcidoidea	Mymaridae	Mymaridae	sp.	KR929126.1
Hymenoptera	Chalcidoidea	Mymaridae	Mymaridae	sp.	KR933733.1
Hymenoptera	Chalcidoidea	Mymaridae	Mymaridae	sp.	KT622265.1
Hymenoptera	Chalcidoidea	Mymaridae	Mymaridae	sp.	KT623747.1
Hymenoptera	Chalcidoidea	Mymaridae	Mymaridae	sp.	KX054001.1
Hymenoptera	Chalcidoidea	Mymaridae	Mymaridae	sp.	MG355655.1
Hymenoptera	Chalcidoidea	Mymaridae	Mymaridae	sp.	MG486645.1
Hymenoptera	Chalcidoidea	Mymaridae	Mymaridae	sp.	MG512515.1
Hymenoptera	Chalcidoidea	Mymaridae	Mymaridae	sp.	MH927315.1
Hymenoptera	Chalcidoidea	Mymaridae	Mymaridae	sp.	MH927677.1
Hymenoptera	Chalcidoidea	Mymaridae	Mymaridae	sp.	MH927854.1
Hymenoptera	Chalcidoidea	Mymaridae	Mymaridae	sp.	MH928131.1
Hymenoptera	Chalcidoidea	Mymaridae	Mymaridae	sp.	MH928606.1
Hymenoptera	Chalcidoidea	Mymaridae	Mymaridae	sp.	MH928663.1
Hymenoptera	Chalcidoidea	Mymaridae	Mymaridae	sp.	MH928786.1
Hymenoptera	Chalcidoidea	Mymaridae	Mymaridae	sp.	MH928949.1
Hymenoptera	Chalcidoidea	Mymaridae	Mymaridae	sp.	MN665396.1
Hymenoptera	Chalcidoidea	Mymaridae	Mymaridae	sp.	MN666039.1
Hymenoptera	Chalcidoidea	Mymaridae	Mymaridae	sp.	MN666218.1
Hymenoptera	Chalcidoidea	Mymaridae	Mymaridae	sp.	MN666536.1
Hymenoptera	Chalcidoidea	Mymaridae	Mymaridae	sp.	MN667247.1
Hymenoptera	Chalcidoidea	Mymaridae	Mymaridae	sp.	MN667349.1
Hymenoptera	Chalcidoidea	Mymaridae	Mymaridae	sp.	MN667598.1
Hymenoptera	Chalcidoidea	Mymaridae	Mymaridae	sp.	MN668321.1
Hymenoptera	Chalcidoidea	Mymaridae	Mymaridae	sp.	MN669146.1
Hymenoptera	Chalcidoidea	Mymaridae	Mymaridae	sp.	MN669258.1
Hymenoptera	Chalcidoidea	Mymaridae	Mymaridae	sp.	MN669879.1
Hymenoptera	Chalcidoidea	Mymaridae	Mymaridae	sp.	MN670366.1
Hymenoptera	Chalcidoidea	Mymaridae	Mymaridae	sp.	MN670473.1
Hymenoptera	Chalcidoidea	Mymaridae	Mymaridae	sp.	MN670497.1
Hymenoptera	Chalcidoidea	Mymaridae	Mymaridae	sp.	MN670729.1
Hymenoptera	Chalcidoidea	Mymaridae	Mymaridae	sp.	MN670786.1
Hymenoptera	Chalcidoidea	Mymaridae	Mymaridae	sp.	MN670932.1
Hymenoptera	Chalcidoidea	Mymaridae	Mymaridae	sp.	MN671412.1
Hymenoptera	Chalcidoidea	Mymaridae	Mymaridae	sp.	MN671721.1
Hymenoptera	Chalcidoidea	Mymaridae	Mymaridae	sp.	MN672143.1
Hymenoptera	Chalcidoidea	Mymaridae	Mymaridae	sp.	MN672568.1
Hymenoptera	Chalcidoidea	Mymaridae	Mymaridae	sp.	MN673394.1
Hymenoptera	Chalcidoidea	Mymaridae	Mymaridae	sp.	MN673848.1
Hymenoptera	Chalcidoidea	Mymaridae	Mymaridae	sp.	MN674331.1
Hymenoptera	Chalcidoidea	Mymaridae	Mymaridae	sp.	MN674713.1
Hymenoptera	Chalcidoidea	Mymaridae	Mymaridae	sp.	MN674768.1
Hymenoptera	Chalcidoidea	Mymaridae	Mymaridae	sp.	MN675923.1
Hymenoptera	Chalcidoidea	Mymaridae	Mymaridae	sp.	MN676041.1
Hymenoptera	Chalcidoidea	Mymaridae	Mymaridae	sp.	MN676042.1
Hymenoptera	Chalcidoidea	Mymaridae	Mymaridae	sp.	MN676140.1
Hymenoptera	Chalcidoidea	Mymaridae	Mymaridae	sp.	MN676632.1

Hymenoptera	Chalcidoidea	Mymaridae	Mymaridae	sp.	MN676803.1
Hymenoptera	Chalcidoidea	Mymaridae	Mymaridae	sp.	MN676984.1
Hymenoptera	Chalcidoidea	Mymaridae	Mymaridae	sp.	MN677010.1
Hymenoptera	Chalcidoidea	Mymaridae	Mymaridae	sp.	MN677159.1
Hymenoptera	Chalcidoidea	Mymaridae	Mymaridae	sp.	MN677488.1
Hymenoptera	Chalcidoidea	Mymaridae	Mymaridae	sp.	MN677525.1
Hymenoptera	Chalcidoidea	Mymaridae	Mymaridae	sp.	MN677651.1
Hymenoptera	Chalcidoidea	Mymaridae	Mymaridae	sp.	MN677852.1
Hymenoptera	Chalcidoidea	Mymaridae	Mymaridae	sp.	MN677956.1
Hymenoptera	Chalcidoidea	Mymaridae	Mymaridae	sp.	MN678111.1
Hymenoptera	Chalcidoidea	Mymaridae	Mymaridae	sp.	MN679186.1
Hymenoptera	Chalcidoidea	Mymaridae	Mymaridae	sp.	MN679421.1
Hymenoptera	Chalcidoidea	Mymaridae	Mymaridae	sp.	MN679572.1
Hymenoptera	Chalcidoidea	Mymaridae	Mymaridae	sp.	MN679616.1
Hymenoptera	Chalcidoidea	Mymaridae	Mymaridae	sp.	MN679872.1
Hymenoptera	Chalcidoidea	Mymaridae	Mymaridae	sp.	MN680128.1
Hymenoptera	Chalcidoidea	Mymaridae	Mymaridae	sp.	MN680135.1
Hymenoptera	Chalcidoidea	Mymaridae	Mymaridae	sp.	MN680307.1
Hymenoptera	Chalcidoidea	Mymaridae	Mymaridae	sp.	MN682270.1
Hymenoptera	Chalcidoidea	Mymaridae	Mymaridae	sp.	MN682743.1
Hymenoptera	Chalcidoidea	Mymaridae	Neomymar	sp.	KR411486.1
Hymenoptera	Chalcidoidea	Mymaridae	Neomymar	sp.	KY831792.1
Hymenoptera	Chalcidoidea	Mymaridae	Neomymar	sp.	KY835998.1
Hymenoptera	Chalcidoidea	Mymaridae	Neomymar	sp.	KY839362.1
Hymenoptera	Chalcidoidea	Mymaridae	Omyomymar	sp.	MG349291.1
Hymenoptera	Chalcidoidea	Mymaridae	Omyomymar	sp.	MG501388.1
Hymenoptera	Chalcidoidea	Mymaridae	Ooctonus	notatus	KC157681.1
Hymenoptera	Chalcidoidea	Mymaridae	Ooctonus	notatus	KR882231.1
Hymenoptera	Chalcidoidea	Mymaridae	Ooctonus	notatus	KR900317.1
Hymenoptera	Chalcidoidea	Mymaridae	Ooctonus	notatus	KR923772.1
Hymenoptera	Chalcidoidea	Mymaridae	Ooctonus	notatus	KR923965.1
Hymenoptera	Chalcidoidea	Mymaridae	Ooctonus	notatus	KR925203.1
Hymenoptera	Chalcidoidea	Mymaridae	Ooctonus	notatus	KR925553.1
Hymenoptera	Chalcidoidea	Mymaridae	Ooctonus	notatus	KR925639.1
Hymenoptera	Chalcidoidea	Mymaridae	Ooctonus	notatus	KR926525.1
Hymenoptera	Chalcidoidea	Mymaridae	Ooctonus	notatus	KR926768.1
Hymenoptera	Chalcidoidea	Mymaridae	Ooctonus	notatus	KR927403.1
Hymenoptera	Chalcidoidea	Mymaridae	Ooctonus	notatus	KR927472.1
Hymenoptera	Chalcidoidea	Mymaridae	Ooctonus	notatus	KR928380.1
Hymenoptera	Chalcidoidea	Mymaridae	Ooctonus	notatus	KR929001.1
Hymenoptera	Chalcidoidea	Mymaridae	Ooctonus	notatus	KR930409.1
Hymenoptera	Chalcidoidea	Mymaridae	Ooctonus	notatus	KR931002.1
Hymenoptera	Chalcidoidea	Mymaridae	Ooctonus	notatus	KR931323.1
Hymenoptera	Chalcidoidea	Mymaridae	Ooctonus	notatus	KR931497.1
Hymenoptera	Chalcidoidea	Mymaridae	Ooctonus	notatus	KR931535.1
Hymenoptera	Chalcidoidea	Mymaridae	Ooctonus	notatus	KR931920.1
Hymenoptera	Chalcidoidea	Mymaridae	Ooctonus	notatus	KR932153.1
Hymenoptera	Chalcidoidea	Mymaridae	Ooctonus	notatus	KR932208.1
Hymenoptera	Chalcidoidea	Mymaridae	Ooctonus	notatus	KR932373.1
Hymenoptera	Chalcidoidea	Mymaridae	Ooctonus	notatus	KR934495.1
Hymenoptera	Chalcidoidea	Mymaridae	Ooctonus	notatus	KR934556.1
Hymenoptera	Chalcidoidea	Mymaridae	Ooctonus	notatus	KR935199.1
Hymenoptera	Chalcidoidea	Mymaridae	Ooctonus	notatus	MG350694.1
Hymenoptera	Chalcidoidea	Mymaridae	Ooctonus	notatus	MG351921.1

Hymenoptera	Chalcidoidea	Mymaridae	Ooctonus	notatus	MG353087.1
Hymenoptera	Chalcidoidea	Mymaridae	Ooctonus	notatus	MG353399.1
Hymenoptera	Chalcidoidea	Mymaridae	Ooctonus	silvensis	KC157689.1
Hymenoptera	Chalcidoidea	Mymaridae	Ooctonus	silvensis	KC157690.1
Hymenoptera	Chalcidoidea	Mymaridae	Ooctonus	silvensis	KM559690.1
Hymenoptera	Chalcidoidea	Mymaridae	Ooctonus	silvensis	KM563181.1
Hymenoptera	Chalcidoidea	Mymaridae	Ooctonus	silvensis	KM567869.1
Hymenoptera	Chalcidoidea	Mymaridae	Ooctonus	silvensis	KM568001.1
Hymenoptera	Chalcidoidea	Mymaridae	Ooctonus	silvensis	KR792758.1
Hymenoptera	Chalcidoidea	Mymaridae	Ooctonus	silvensis	KR803810.1
Hymenoptera	Chalcidoidea	Mymaridae	Ooctonus	silvensis	KT701016.1
Hymenoptera	Chalcidoidea	Mymaridae	Ooctonus	silvensis	MG355226.1
Hymenoptera	Chalcidoidea	Mymaridae	Ooctonus	silvensis	MG504042.1
Hymenoptera	Chalcidoidea	Mymaridae	Ooctonus	sp.	JN292358.1
Hymenoptera	Chalcidoidea	Mymaridae	Ooctonus	sp.	KC157693.1
Hymenoptera	Chalcidoidea	Mymaridae	Ooctonus	sp.	KC157694.1
Hymenoptera	Chalcidoidea	Mymaridae	Ooctonus	sp.	KC157697.1
Hymenoptera	Chalcidoidea	Mymaridae	Ooctonus	sp.	KR782518.1
Hymenoptera	Chalcidoidea	Mymaridae	Ooctonus	sp.	KR786870.1
Hymenoptera	Chalcidoidea	Mymaridae	Ooctonus	sp.	KR798723.1
Hymenoptera	Chalcidoidea	Mymaridae	Ooctonus	sp.	KR801423.1
Hymenoptera	Chalcidoidea	Mymaridae	Ooctonus	sp.	KR808865.1
Hymenoptera	Chalcidoidea	Mymaridae	Ooctonus	sp.	KR896271.1
Hymenoptera	Chalcidoidea	Mymaridae	Ooctonus	sp.	KT704035.1
Hymenoptera	Chalcidoidea	Mymaridae	Ooctonus	sp.	MG348205.1
Hymenoptera	Chalcidoidea	Mymaridae	Ooctonus	sp.	MG350902.1
Hymenoptera	Chalcidoidea	Mymaridae	Ooctonus	sp.	MG355755.1
Hymenoptera	Chalcidoidea	Mymaridae	Ooctonus	sp.	MG502977.1
Hymenoptera	Chalcidoidea	Mymaridae	Polynema	sp.	HM414674.1
Hymenoptera	Chalcidoidea	Mymaridae	Polynema	sp.	HQ929643.1
Hymenoptera	Chalcidoidea	Mymaridae	Polynema	sp.	HQ929776.1
Hymenoptera	Chalcidoidea	Mymaridae	Polynema	sp.	KM556132.1
Hymenoptera	Chalcidoidea	Mymaridae	Polynema	sp.	KM558074.1
Hymenoptera	Chalcidoidea	Mymaridae	Polynema	sp.	KM559646.1
Hymenoptera	Chalcidoidea	Mymaridae	Polynema	sp.	KM563316.1
Hymenoptera	Chalcidoidea	Mymaridae	Polynema	sp.	KM565009.1
Hymenoptera	Chalcidoidea	Mymaridae	Polynema	sp.	KM567061.1
Hymenoptera	Chalcidoidea	Mymaridae	Polynema	sp.	KR402569.1
Hymenoptera	Chalcidoidea	Mymaridae	Polynema	sp.	KR402677.1
Hymenoptera	Chalcidoidea	Mymaridae	Polynema	sp.	KR404377.1
Hymenoptera	Chalcidoidea	Mymaridae	Polynema	sp.	KR404661.1
Hymenoptera	Chalcidoidea	Mymaridae	Polynema	sp.	KR408485.1
Hymenoptera	Chalcidoidea	Mymaridae	Polynema	sp.	KR409149.1
Hymenoptera	Chalcidoidea	Mymaridae	Polynema	sp.	KR409880.1
Hymenoptera	Chalcidoidea	Mymaridae	Polynema	sp.	KR413780.1
Hymenoptera	Chalcidoidea	Mymaridae	Polynema	sp.	KR413964.1
Hymenoptera	Chalcidoidea	Mymaridae	Polynema	sp.	KR420561.1
Hymenoptera	Chalcidoidea	Mymaridae	Polynema	sp.	KR421931.1
Hymenoptera	Chalcidoidea	Mymaridae	Polynema	sp.	KR422265.1
Hymenoptera	Chalcidoidea	Mymaridae	Polynema	sp.	KR790148.1
Hymenoptera	Chalcidoidea	Mymaridae	Polynema	sp.	KR801153.1
Hymenoptera	Chalcidoidea	Mymaridae	Polynema	sp.	KR802316.1
Hymenoptera	Chalcidoidea	Mymaridae	Polynema	sp.	KR803621.1
Hymenoptera	Chalcidoidea	Mymaridae	Polynema	sp.	KR805328.1

Hymenoptera	Chalcidoidea	Mymaridae	Polynema	sp.	KR807944.1
Hymenoptera	Chalcidoidea	Mymaridae	Polynema	sp.	KR874824.1
Hymenoptera	Chalcidoidea	Mymaridae	Polynema	sp.	KR875794.1
Hymenoptera	Chalcidoidea	Mymaridae	Polynema	sp.	KR877821.1
Hymenoptera	Chalcidoidea	Mymaridae	Polynema	sp.	KR884094.1
Hymenoptera	Chalcidoidea	Mymaridae	Polynema	sp.	KR895127.1
Hymenoptera	Chalcidoidea	Mymaridae	Polynema	sp.	KR895373.1
Hymenoptera	Chalcidoidea	Mymaridae	Polynema	sp.	KR899347.1
Hymenoptera	Chalcidoidea	Mymaridae	Polynema	sp.	KR901104.1
Hymenoptera	Chalcidoidea	Mymaridae	Polynema	sp.	KY838693.1
Hymenoptera	Chalcidoidea	Mymaridae	Polynema	sp.	KY841811.1
Hymenoptera	Chalcidoidea	Mymaridae	Polynema	sp.	KY843961.1
Hymenoptera	Chalcidoidea	Mymaridae	Polynema	sp.	MG346316.1
Hymenoptera	Chalcidoidea	Mymaridae	Polynema	sp.	MG348144.1
Hymenoptera	Chalcidoidea	Mymaridae	Polynema	sp.	MG349225.1
Hymenoptera	Chalcidoidea	Mymaridae	Polynema	sp.	MG350290.1
Hymenoptera	Chalcidoidea	Mymaridae	Polynema	sp.	MG353028.1
Hymenoptera	Chalcidoidea	Mymaridae	Polynema	sp.	MG354165.1
Hymenoptera	Chalcidoidea	Mymaridae	Polynema	sp.	MG355133.1
Hymenoptera	Chalcidoidea	Mymaridae	Polynema	sp.	MZ629208.1
Hymenoptera	Chalcidoidea	Mymaridae	Stephanodes	sp.	MG355255.1
Hymenoptera	Chalcidoidea	Mymaridae	Stephanodes	sp.	MG499778.1
Hymenoptera	Chalcidoidea	Ormyridae	Ormyridae	sp.	HQ930215.1
Hymenoptera	Chalcidoidea	Ormyridae	Ormyridae	sp.	HQ930368.1
Hymenoptera	Chalcidoidea	Ormyridae	Ormyridae	sp.	HQ930369.1
Hymenoptera	Chalcidoidea	Ormyridae	Ormyridae	sp.	HQ930370.1
Hymenoptera	Chalcidoidea	Ormyridae	Ormyridae	sp.	HQ930382.1
Hymenoptera	Chalcidoidea	Ormyridae	Ormyridae	sp.	MG348544.1
Hymenoptera	Chalcidoidea	Ormyridae	Ormyrus	labotus	KR108716.1
Hymenoptera	Chalcidoidea	Ormyridae	Ormyrus	sp.	HQ930344.1
Hymenoptera	Chalcidoidea	Perilampidae	Monacon	simplex	MW984029.1
Hymenoptera	Chalcidoidea	Perilampidae	Monacon	tricorne	MW983752.1
Hymenoptera	Chalcidoidea	Perilampidae	Perilampidae	sp.	HQ552520.1
Hymenoptera	Chalcidoidea	Perilampidae	Perilampidae	sp.	HQ927051.1
Hymenoptera	Chalcidoidea	Perilampidae	Perilampidae	sp.	KM555659.1
Hymenoptera	Chalcidoidea	Perilampidae	Perilampidae	sp.	KM557720.1
Hymenoptera	Chalcidoidea	Perilampidae	Perilampidae	sp.	KM558289.1
Hymenoptera	Chalcidoidea	Perilampidae	Perilampidae	sp.	KM558344.1
Hymenoptera	Chalcidoidea	Perilampidae	Perilampidae	sp.	KM558440.1
Hymenoptera	Chalcidoidea	Perilampidae	Perilampidae	sp.	KM559020.1
Hymenoptera	Chalcidoidea	Perilampidae	Perilampidae	sp.	KM559172.1
Hymenoptera	Chalcidoidea	Perilampidae	Perilampidae	sp.	KM559212.1
Hymenoptera	Chalcidoidea	Perilampidae	Perilampidae	sp.	KM559266.1
Hymenoptera	Chalcidoidea	Perilampidae	Perilampidae	sp.	KM559622.1
Hymenoptera	Chalcidoidea	Perilampidae	Perilampidae	sp.	KM560927.1
Hymenoptera	Chalcidoidea	Perilampidae	Perilampidae	sp.	KM561861.1
Hymenoptera	Chalcidoidea	Perilampidae	Perilampidae	sp.	KM563559.1
Hymenoptera	Chalcidoidea	Perilampidae	Perilampidae	sp.	KM564359.1
Hymenoptera	Chalcidoidea	Perilampidae	Perilampidae	sp.	KM565054.1
Hymenoptera	Chalcidoidea	Perilampidae	Perilampidae	sp.	KM565056.1
Hymenoptera	Chalcidoidea	Perilampidae	Perilampidae	sp.	KM565057.1
Hymenoptera	Chalcidoidea	Perilampidae	Perilampidae	sp.	KM567460.1
Hymenoptera	Chalcidoidea	Perilampidae	Perilampidae	sp.	KM569212.1
Hymenoptera	Chalcidoidea	Perilampidae	Perilampidae	sp.	KR787397.1

Hymenoptera	Chalcidoidea	Perilampidae	Perilampidae	sp.	MG346524.1
Hymenoptera	Chalcidoidea	Perilampidae	Perilampidae	sp.	MG497770.1
Hymenoptera	Chalcidoidea	Perilampidae	Perilampidae	sp.	MG501612.1
Hymenoptera	Chalcidoidea	Perilampidae	Perilampidae	sp.	MH926749.1
Hymenoptera	Chalcidoidea	Perilampidae	Perilampidae	sp.	MH926928.1
Hymenoptera	Chalcidoidea	Perilampidae	Perilampinae	sp.	HQ930136.1
Hymenoptera	Chalcidoidea	Perilampidae	Perilampus	anomocerus	KR783178.1
Hymenoptera	Chalcidoidea	Perilampidae	Perilampus	chrysopae	KM561992.1
Hymenoptera	Chalcidoidea	Perilampidae	Perilampus	chrysopae	KM562486.1
Hymenoptera	Chalcidoidea	Perilampidae	Perilampus	chrysopae	KM566788.1
Hymenoptera	Chalcidoidea	Perilampidae	Perilampus	sp.	HQ107674.1
Hymenoptera	Chalcidoidea	Perilampidae	Perilampus	sp.	HQ107675.1
Hymenoptera	Chalcidoidea	Perilampidae	Perilampus	sp.	HQ107676.1
Hymenoptera	Chalcidoidea	Perilampidae	Perilampus	sp.	HQ107677.1
Hymenoptera	Chalcidoidea	Perilampidae	Perilampus	sp.	HQ107678.1
Hymenoptera	Chalcidoidea	Perilampidae	Perilampus	sp.	HQ107680.1
Hymenoptera	Chalcidoidea	Perilampidae	Perilampus	sp.	HQ107681.1
Hymenoptera	Chalcidoidea	Perilampidae	Perilampus	sp.	HQ107685.1
Hymenoptera	Chalcidoidea	Perilampidae	Perilampus	sp.	HQ107686.1
Hymenoptera	Chalcidoidea	Perilampidae	Perilampus	sp.	HQ107687.1
Hymenoptera	Chalcidoidea	Perilampidae	Perilampus	sp.	HQ107688.1
Hymenoptera	Chalcidoidea	Perilampidae	Perilampus	sp.	HQ107690.1
Hymenoptera	Chalcidoidea	Perilampidae	Perilampus	sp.	HQ107692.1
Hymenoptera	Chalcidoidea	Perilampidae	Perilampus	sp.	HQ107694.1
Hymenoptera	Chalcidoidea	Perilampidae	Perilampus	sp.	HQ107695.1
Hymenoptera	Chalcidoidea	Perilampidae	Perilampus	sp.	JN293499.1
Hymenoptera	Chalcidoidea	Perilampidae	Perilampus	sp.	JQ574565.1
Hymenoptera	Chalcidoidea	Perilampidae	Perilampus	sp.	JQ574566.1
Hymenoptera	Chalcidoidea	Perilampidae	Perilampus	sp.	JQ574567.1
Hymenoptera	Chalcidoidea	Perilampidae	Perilampus	sp.	KM557041.1
Hymenoptera	Chalcidoidea	Perilampidae	Perilampus	sp.	KR784372.1
Hymenoptera	Chalcidoidea	Perilampidae	Perilampus	sp.	KR786032.1
Hymenoptera	Chalcidoidea	Perilampidae	Perilampus	sp.	KR806731.1
Hymenoptera	Chalcidoidea	Perilampidae	Perilampus	sp.	KR875555.1
Hymenoptera	Chalcidoidea	Perilampidae	Perilampus	sp.	KR881140.1
Hymenoptera	Chalcidoidea	Perilampidae	Perilampus	sp.	KR885320.1
Hymenoptera	Chalcidoidea	Perilampidae	Perilampus	sp.	KR887932.1
Hymenoptera	Chalcidoidea	Perilampidae	Perilampus	sp.	KR888500.1
Hymenoptera	Chalcidoidea	Perilampidae	Perilampus	sp.	KR895133.1
Hymenoptera	Chalcidoidea	Perilampidae	Perilampus	sp.	KR898959.1
Hymenoptera	Chalcidoidea	Perilampidae	Perilampus	sp.	MF737457.1
Hymenoptera	Chalcidoidea	Perilampidae	Perilampus	sp.	MG353954.1
Hymenoptera	Chalcidoidea	Perilampidae	Perilampus	sp.	MG353982.1
Hymenoptera	Chalcidoidea	Perilampidae	Perilampus	sp.	MG355743.1
Hymenoptera	Chalcidoidea	Perilampidae	Perilampus	sp.	MG506920.1
Hymenoptera	Chalcidoidea	Perilampidae	Perilampus	tristis	KP072598.1
Hymenoptera	Chalcidoidea	Perilampidae	Perilampus	tristis	KP072600.1
Hymenoptera	Chalcidoidea	Perilampidae	Perilampus	tristis	KP072603.1
Hymenoptera	Chalcidoidea	Perilampidae	Perilampus	tristis	KP072604.1
Hymenoptera	Chalcidoidea	Perilampidae	Perilampus	tristis	KP072605.1
Hymenoptera	Chalcidoidea	Pteromalidae	Acophila	sp.	MK530713.1
Hymenoptera	Chalcidoidea	Pteromalidae	Acophila	sp.	MK530714.1
Hymenoptera	Chalcidoidea	Pteromalidae	Acophila	sp.	MK530715.1
Hymenoptera	Chalcidoidea	Pteromalidae	Acophila	sp.	MK530716.1

Hymenoptera	Chalcidoidea	Pteromalidae	Acophila	sp.	MK530718.1
Hymenoptera	Chalcidoidea	Pteromalidae	Acophila	sp.	MK530719.1
Hymenoptera	Chalcidoidea	Pteromalidae	Acophila	sp.	MK530787.1
Hymenoptera	Chalcidoidea	Pteromalidae	Acroclisoides	sinicus	MN395435.1
Hymenoptera	Chalcidoidea	Pteromalidae	Acroclisoides	sinicus	MN395436.1
Hymenoptera	Chalcidoidea	Pteromalidae	Acroclisoides	sinicus	MN395442.1
Hymenoptera	Chalcidoidea	Pteromalidae	Acroclisoides	sinicus	MN395449.1
Hymenoptera	Chalcidoidea	Pteromalidae	Acroclisoides	sinicus	MN395451.1
Hymenoptera	Chalcidoidea	Pteromalidae	Acroclisoides	sinicus	MN395459.1
Hymenoptera	Chalcidoidea	Pteromalidae	Acroclisoides	sinicus	MN395462.1
Hymenoptera	Chalcidoidea	Pteromalidae	Acroclisoides	sinicus	MN395465.1
Hymenoptera	Chalcidoidea	Pteromalidae	Acroclisoides	sinicus	MN413501.1
Hymenoptera	Chalcidoidea	Pteromalidae	Acroclisoides	sinicus	MN413502.1
Hymenoptera	Chalcidoidea	Pteromalidae	Acroclisoides	sinicus	MN413503.1
Hymenoptera	Chalcidoidea	Pteromalidae	Acroclisoides	solus	MH521285.1
Hymenoptera	Chalcidoidea	Pteromalidae	Acroclisoides	solus	MK188331.1
Hymenoptera	Chalcidoidea	Pteromalidae	Acrocormus	semifasciatus	OL538120.1
Hymenoptera	Chalcidoidea	Pteromalidae	Agiommatus	sp.	OL664064.1
Hymenoptera	Chalcidoidea	Pteromalidae	Anisopteromalus	calandrae	AB690353.1
Hymenoptera	Chalcidoidea	Pteromalidae	Anisopteromalus	calandrae	AB690354.1
Hymenoptera	Chalcidoidea	Pteromalidae	Anisopteromalus	sp.	AB690357.1
Hymenoptera	Chalcidoidea	Pteromalidae	Apelioma	pteromalinum	OL538074.1
Hymenoptera	Chalcidoidea	Pteromalidae	Apelioma	pteromalinum	OL538094.1
Hymenoptera	Chalcidoidea	Pteromalidae	Apelioma	pteromalinum	OL538141.1
Hymenoptera	Chalcidoidea	Pteromalidae	Apocrypta	bakeri	JF816303.1
Hymenoptera	Chalcidoidea	Pteromalidae	Apocrypta	bakeri	KF778385.1
Hymenoptera	Chalcidoidea	Pteromalidae	Arthrolytus	slovacus	OL538078.1
Hymenoptera	Chalcidoidea	Pteromalidae	Arthrolytus	slovacus	OL538110.1
Hymenoptera	Chalcidoidea	Pteromalidae	Arthrolytus	sp.	MW982696.1
Hymenoptera	Chalcidoidea	Pteromalidae	Asaphes	sp.	JF863268.1
Hymenoptera	Chalcidoidea	Pteromalidae	Asaphes	sp.	KU496688.1
Hymenoptera	Chalcidoidea	Pteromalidae	Asaphes	sp.	MF850289.1
Hymenoptera	Chalcidoidea	Pteromalidae	Asaphes	sp.	MF850291.1
Hymenoptera	Chalcidoidea	Pteromalidae	Asaphes	suspensus	JX507454.1
Hymenoptera	Chalcidoidea	Pteromalidae	Asaphes	suspensus	KY912676.1
Hymenoptera	Chalcidoidea	Pteromalidae	Asaphes	vulgaris	KF802812.1
Hymenoptera	Chalcidoidea	Pteromalidae	Asaphes	vulgaris	KF802813.1
Hymenoptera	Chalcidoidea	Pteromalidae	Asaphes	vulgaris	KM556888.1
Hymenoptera	Chalcidoidea	Pteromalidae	Asaphes	vulgaris	KM557440.1
Hymenoptera	Chalcidoidea	Pteromalidae	Asaphes	vulgaris	KM565300.1
Hymenoptera	Chalcidoidea	Pteromalidae	Asaphes	vulgaris	KR784097.1
Hymenoptera	Chalcidoidea	Pteromalidae	Asaphes	vulgaris	KR798214.1
Hymenoptera	Chalcidoidea	Pteromalidae	Asaphes	vulgaris	KR798261.1
Hymenoptera	Chalcidoidea	Pteromalidae	Asaphes	vulgaris	KR808719.1
Hymenoptera	Chalcidoidea	Pteromalidae	Asaphes	vulgaris	KR888424.1
Hymenoptera	Chalcidoidea	Pteromalidae	Asaphes	vulgaris	KR890581.1
Hymenoptera	Chalcidoidea	Pteromalidae	Asaphes	vulgaris	KY912681.1
Hymenoptera	Chalcidoidea	Pteromalidae	Asaphes	vulgaris	MG380711.1
Hymenoptera	Chalcidoidea	Pteromalidae	Atrichomalus	trianellatus	OL538102.1
Hymenoptera	Chalcidoidea	Pteromalidae	Camarothorax	sp.	MK543435.1
Hymenoptera	Chalcidoidea	Pteromalidae	Cecidostiba	sp.	JN293378.1
Hymenoptera	Chalcidoidea	Pteromalidae	Cecidostiba	sp.	KM558329.1
Hymenoptera	Chalcidoidea	Pteromalidae	Cecidostiba	sp.	KM562729.1
Hymenoptera	Chalcidoidea	Pteromalidae	Cecidostiba	sp.	KR365805.1

Hymenoptera	Chalcidoidea	Pteromalidae	Cecidostiba	sp.	KR366403.1
Hymenoptera	Chalcidoidea	Pteromalidae	Cecidostiba	sp.	KR367785.1
Hymenoptera	Chalcidoidea	Pteromalidae	Cecidostiba	sp.	KR368188.1
Hymenoptera	Chalcidoidea	Pteromalidae	Cecidostiba	sp.	KR369017.1
Hymenoptera	Chalcidoidea	Pteromalidae	Cecidostiba	sp.	KR369091.1
Hymenoptera	Chalcidoidea	Pteromalidae	Cecidostiba	sp.	KR369781.1
Hymenoptera	Chalcidoidea	Pteromalidae	Cecidostiba	sp.	KR371078.1
Hymenoptera	Chalcidoidea	Pteromalidae	Cecidostiba	sp.	KR371304.1
Hymenoptera	Chalcidoidea	Pteromalidae	Cecidostiba	sp.	KR371587.1
Hymenoptera	Chalcidoidea	Pteromalidae	Cecidostiba	sp.	KR372133.1
Hymenoptera	Chalcidoidea	Pteromalidae	Cecidostiba	sp.	KR373124.1
Hymenoptera	Chalcidoidea	Pteromalidae	Cecidostiba	sp.	KR373379.1
Hymenoptera	Chalcidoidea	Pteromalidae	Cecidostiba	sp.	KR373513.1
Hymenoptera	Chalcidoidea	Pteromalidae	Cecidostiba	sp.	KR374320.1
Hymenoptera	Chalcidoidea	Pteromalidae	Cecidostiba	sp.	KR374948.1
Hymenoptera	Chalcidoidea	Pteromalidae	Cecidostiba	sp.	KR783553.1
Hymenoptera	Chalcidoidea	Pteromalidae	Cecidostiba	sp.	KR784169.1
Hymenoptera	Chalcidoidea	Pteromalidae	Cecidostiba	sp.	KR796579.1
Hymenoptera	Chalcidoidea	Pteromalidae	Cecidostiba	sp.	KR797185.1
Hymenoptera	Chalcidoidea	Pteromalidae	Cecidostiba	sp.	KR797648.1
Hymenoptera	Chalcidoidea	Pteromalidae	Cecidostiba	sp.	KR798639.1
Hymenoptera	Chalcidoidea	Pteromalidae	Cecidostiba	sp.	KR800018.1
Hymenoptera	Chalcidoidea	Pteromalidae	Cecidostiba	sp.	KR805317.1
Hymenoptera	Chalcidoidea	Pteromalidae	Cecidostiba	sp.	KR806533.1
Hymenoptera	Chalcidoidea	Pteromalidae	Cecidostiba	sp.	KR877471.1
Hymenoptera	Chalcidoidea	Pteromalidae	Cecidostiba	sp.	KR893276.1
Hymenoptera	Chalcidoidea	Pteromalidae	Cecidostiba	sp.	KR924744.1
Hymenoptera	Chalcidoidea	Pteromalidae	Cecidostiba	sp.	KR932183.1
Hymenoptera	Chalcidoidea	Pteromalidae	Cecidostiba	sp.	KR933704.1
Hymenoptera	Chalcidoidea	Pteromalidae	Cecidostiba	sp.	KT266712.1
Hymenoptera	Chalcidoidea	Pteromalidae	Cecidostiba	sp.	MG374357.1
Hymenoptera	Chalcidoidea	Pteromalidae	Cecidostiba	sp.	MG375208.1
Hymenoptera	Chalcidoidea	Pteromalidae	Cecidostiba	sp.	MG375564.1
Hymenoptera	Chalcidoidea	Pteromalidae	Cecidostiba	sp.	MG378495.1
Hymenoptera	Chalcidoidea	Pteromalidae	Cecidostiba	sp.	MG379412.1
Hymenoptera	Chalcidoidea	Pteromalidae	Cecidostiba	sp.	MG380992.1
Hymenoptera	Chalcidoidea	Pteromalidae	Cecidostiba	sp.	MG382272.1
Hymenoptera	Chalcidoidea	Pteromalidae	Cecidostiba	sp.	MG498394.1
Hymenoptera	Chalcidoidea	Pteromalidae	Cecidostiba	sp.	MG498515.1
Hymenoptera	Chalcidoidea	Pteromalidae	Cecidostiba	sp.	MG498732.1
Hymenoptera	Chalcidoidea	Pteromalidae	Cecidostiba	sp.	MG498831.1
Hymenoptera	Chalcidoidea	Pteromalidae	Cecidostiba	sp.	MG501422.1
Hymenoptera	Chalcidoidea	Pteromalidae	Cecidostiba	sp.	MG502886.1
Hymenoptera	Chalcidoidea	Pteromalidae	Cecidostiba	sp.	MG505322.1
Hymenoptera	Chalcidoidea	Pteromalidae	Cecidostiba	sp.	MG506643.1
Hymenoptera	Chalcidoidea	Pteromalidae	Cecidostiba	sp.	MN665946.1
Hymenoptera	Chalcidoidea	Pteromalidae	Cecidostiba	sp.	MN667218.1
Hymenoptera	Chalcidoidea	Pteromalidae	Cecidostiba	sp.	MN670044.1
Hymenoptera	Chalcidoidea	Pteromalidae	Cecidostiba	sp.	MN671237.1
Hymenoptera	Chalcidoidea	Pteromalidae	Cecidostiba	sp.	MN675294.1
Hymenoptera	Chalcidoidea	Pteromalidae	Cecidostiba	sp.	MN676491.1
Hymenoptera	Chalcidoidea	Pteromalidae	Cecidostiba	sp.	MN676618.1
Hymenoptera	Chalcidoidea	Pteromalidae	Cecidostiba	sp.	MN679383.1
Hymenoptera	Chalcidoidea	Pteromalidae	Cecidostiba	sp.	MN679960.1

Hymenoptera	Chalcidoidea	Pteromalidae	Cecidostiba	sp.	MN680345.1
Hymenoptera	Chalcidoidea	Pteromalidae	Cecidostiba	sp.	MN682342.1
Hymenoptera	Chalcidoidea	Pteromalidae	Cerocephala	sp.	MG100830.1
Hymenoptera	Chalcidoidea	Pteromalidae	Cerocephala	sp.	MG100831.1
Hymenoptera	Chalcidoidea	Pteromalidae	Chlorocytus	sp.	KR365324.1
Hymenoptera	Chalcidoidea	Pteromalidae	Chlorocytus	sp.	KR366372.1
Hymenoptera	Chalcidoidea	Pteromalidae	Chlorocytus	sp.	KR367697.1
Hymenoptera	Chalcidoidea	Pteromalidae	Chlorocytus	sp.	KR367820.1
Hymenoptera	Chalcidoidea	Pteromalidae	Chlorocytus	sp.	KR368683.1
Hymenoptera	Chalcidoidea	Pteromalidae	Chlorocytus	sp.	KR370268.1
Hymenoptera	Chalcidoidea	Pteromalidae	Chlorocytus	sp.	KR370715.1
Hymenoptera	Chalcidoidea	Pteromalidae	Chlorocytus	sp.	KR374131.1
Hymenoptera	Chalcidoidea	Pteromalidae	Chlorocytus	sp.	KR797217.1
Hymenoptera	Chalcidoidea	Pteromalidae	Chlorocytus	sp.	KR879093.1
Hymenoptera	Chalcidoidea	Pteromalidae	Chlorocytus	sp.	MG378474.1
Hymenoptera	Chalcidoidea	Pteromalidae	Chlorocytus	sp.	MG381320.1
Hymenoptera	Chalcidoidea	Pteromalidae	Chlorocytus	sp.	MG500297.1
Hymenoptera	Chalcidoidea	Pteromalidae	Chlorocytus	sp.	MG503506.1
Hymenoptera	Chalcidoidea	Pteromalidae	Chlorocytus	sp.	MG504496.1
Hymenoptera	Chalcidoidea	Pteromalidae	Colotrechnus	ignotus	HM365026.1
Hymenoptera	Chalcidoidea	Pteromalidae	Colotrechnus	ignotus	MW982420.1
Hymenoptera	Chalcidoidea	Pteromalidae	Crossogaster	inusitata	JQ756567.1
Hymenoptera	Chalcidoidea	Pteromalidae	Crossogaster	odorans	JQ756562.1
Hymenoptera	Chalcidoidea	Pteromalidae	Crossogaster	odorans	JQ756588.1
Hymenoptera	Chalcidoidea	Pteromalidae	Crossogaster	odorans	JQ756591.1
Hymenoptera	Chalcidoidea	Pteromalidae	Crossogaster	quadrata	JQ756580.1
Hymenoptera	Chalcidoidea	Pteromalidae	Crossogaster	sp.	JQ756572.1
Hymenoptera	Chalcidoidea	Pteromalidae	Crossogaster	sp.	JQ756573.1
Hymenoptera	Chalcidoidea	Pteromalidae	Crossogaster	sp.	JQ756594.1
Hymenoptera	Chalcidoidea	Pteromalidae	Crossogaster	stigma	JQ756581.1
Hymenoptera	Chalcidoidea	Pteromalidae	Cryptoprymna	paludicola	OL538067.1
Hymenoptera	Chalcidoidea	Pteromalidae	Cryptoprymna	paludicola	OL538142.1
Hymenoptera	Chalcidoidea	Pteromalidae	Cyclogastrella	clypealis	OL538060.1
Hymenoptera	Chalcidoidea	Pteromalidae	Cyclogastrella	clypealis	OL538061.1
Hymenoptera	Chalcidoidea	Pteromalidae	Cyclogastrella	clypealis	OL538066.1
Hymenoptera	Chalcidoidea	Pteromalidae	Cyclogastrella	clypealis	OL538069.1
Hymenoptera	Chalcidoidea	Pteromalidae	Cyclogastrella	clypealis	OL538072.1
Hymenoptera	Chalcidoidea	Pteromalidae	Cyclogastrella	clypealis	OL538083.1
Hymenoptera	Chalcidoidea	Pteromalidae	Cyclogastrella	clypealis	OL538096.1
Hymenoptera	Chalcidoidea	Pteromalidae	Cyclogastrella	clypealis	OL538100.1
Hymenoptera	Chalcidoidea	Pteromalidae	Cyclogastrella	clypealis	OL538108.1
Hymenoptera	Chalcidoidea	Pteromalidae	Cyclogastrella	clypealis	OL538119.1
Hymenoptera	Chalcidoidea	Pteromalidae	Cyclogastrella	clypealis	OL538143.1
Hymenoptera	Chalcidoidea	Pteromalidae	Cyclogastrella	clypealis	OL538144.1
Hymenoptera	Chalcidoidea	Pteromalidae	Cyclogastrella	plana	MW983179.1
Hymenoptera	Chalcidoidea	Pteromalidae	Diaziella	sp.	JQ756541.1
Hymenoptera	Chalcidoidea	Pteromalidae	Diaziella	sp.	JQ756542.1
Hymenoptera	Chalcidoidea	Pteromalidae	Diaziella	sp.	JQ756543.1
Hymenoptera	Chalcidoidea	Pteromalidae	Diaziella	sp.	JQ756544.1
Hymenoptera	Chalcidoidea	Pteromalidae	Diaziella	sp.	JQ756545.1
Hymenoptera	Chalcidoidea	Pteromalidae	Diaziella	sp.	JQ756546.1
Hymenoptera	Chalcidoidea	Pteromalidae	Diaziella	sp.	JQ756547.1
Hymenoptera	Chalcidoidea	Pteromalidae	Diaziella	sp.	JQ756548.1
Hymenoptera	Chalcidoidea	Pteromalidae	Diaziella	sp.	JQ756549.1

Hymenoptera	Chalcidoidea	Pteromalidae	Diaziella	yangi	MK530785.1
Hymenoptera	Chalcidoidea	Pteromalidae	Dibrachys	cavus	KP072614.1
Hymenoptera	Chalcidoidea	Pteromalidae	Dibrachys	sp.	KR783770.1
Hymenoptera	Chalcidoidea	Pteromalidae	Dibrachys	sp.	KR784981.1
Hymenoptera	Chalcidoidea	Pteromalidae	Dibrachys	sp.	KR791408.1
Hymenoptera	Chalcidoidea	Pteromalidae	Dibrachys	sp.	KR804931.1
Hymenoptera	Chalcidoidea	Pteromalidae	Dibrachys	sp.	KR807726.1
Hymenoptera	Chalcidoidea	Pteromalidae	Dibrachys	sp.	KR873548.1
Hymenoptera	Chalcidoidea	Pteromalidae	Dibrachys	sp.	KR877247.1
Hymenoptera	Chalcidoidea	Pteromalidae	Dibrachys	sp.	KR890037.1
Hymenoptera	Chalcidoidea	Pteromalidae	Dibrachys	sp.	KR891347.1
Hymenoptera	Chalcidoidea	Pteromalidae	Dibrachys	sp.	KR893595.1
Hymenoptera	Chalcidoidea	Pteromalidae	Dibrachys	sp.	KR895666.1
Hymenoptera	Chalcidoidea	Pteromalidae	Dinotiscus	eupterus	KR369653.1
Hymenoptera	Chalcidoidea	Pteromalidae	Dinotiscus	eupterus	KU496721.1
Hymenoptera	Chalcidoidea	Pteromalidae	Dinotiscus	eupterus	MG503090.1
Hymenoptera	Chalcidoidea	Pteromalidae	Dinotiscus	sp.	MG376215.1
Hymenoptera	Chalcidoidea	Pteromalidae	Dinotiscus	sp.	MG381114.1
Hymenoptera	Chalcidoidea	Pteromalidae	Dinotiscus	sp.	MG381176.1
Hymenoptera	Chalcidoidea	Pteromalidae	Dinotiscus	sp.	MG382927.1
Hymenoptera	Chalcidoidea	Pteromalidae	Dinotoides	tenebricus	OL538057.1
Hymenoptera	Chalcidoidea	Pteromalidae	Dinotoides	tenebricus	OL538059.1
Hymenoptera	Chalcidoidea	Pteromalidae	Dinotoides	tenebricus	OL538063.1
Hymenoptera	Chalcidoidea	Pteromalidae	Dinotoides	tenebricus	OL538126.1
Hymenoptera	Chalcidoidea	Pteromalidae	Dinotoides	tenebricus	OL538132.1
Hymenoptera	Chalcidoidea	Pteromalidae	Dinotoides	tenebricus	OL538146.1
Hymenoptera	Chalcidoidea	Pteromalidae	Dinotoides	tenebricus	OL538147.1
Hymenoptera	Chalcidoidea	Pteromalidae	Ecrizotes	longicornis	OL538151.1
Hymenoptera	Chalcidoidea	Pteromalidae	Ecrizotes	monticola	OL538125.1
Hymenoptera	Chalcidoidea	Pteromalidae	Erythromalus	rufiventris	OL538109.1
Hymenoptera	Chalcidoidea	Pteromalidae	Erythromalus	rufiventris	OL538111.1
Hymenoptera	Chalcidoidea	Pteromalidae	Gastrancistrus	acutus	OL538054.1
Hymenoptera	Chalcidoidea	Pteromalidae	Gastrancistrus	affinis	OL538098.1
Hymenoptera	Chalcidoidea	Pteromalidae	Gastrancistrus	compressus	OL538064.1
Hymenoptera	Chalcidoidea	Pteromalidae	Gastrancistrus	compressus	OL538086.1
Hymenoptera	Chalcidoidea	Pteromalidae	Gastrancistrus	compressus	OL538093.1
Hymenoptera	Chalcidoidea	Pteromalidae	Gastrancistrus	compressus	OL538107.1
Hymenoptera	Chalcidoidea	Pteromalidae	Gastrancistrus	fumipeis	OL538071.1
Hymenoptera	Chalcidoidea	Pteromalidae	Gbelcia	crassiceps	OL538103.1
Hymenoptera	Chalcidoidea	Pteromalidae	Halticoptera	circulus	LC542883.1
Hymenoptera	Chalcidoidea	Pteromalidae	Halticoptera	longipetiolus	OL538075.1
Hymenoptera	Chalcidoidea	Pteromalidae	Halticoptera	longipetiolus	OL538149.1
Hymenoptera	Chalcidoidea	Pteromalidae	Halticopteroides	exemae	KF444822.1
Hymenoptera	Chalcidoidea	Pteromalidae	Heteroprymna	longicornis	OL538156.1
Hymenoptera	Chalcidoidea	Pteromalidae	Heteroprymna	longicornis	OL538157.1
Hymenoptera	Chalcidoidea	Pteromalidae	Heteroschema	sp.	MW982440.1
Hymenoptera	Chalcidoidea	Pteromalidae	Homoporus	pyrsius	KJ087770.1
Hymenoptera	Chalcidoidea	Pteromalidae	Homoporus	pyrsius	KR797819.1
Hymenoptera	Chalcidoidea	Pteromalidae	Hypopteromalus	percussor	KM559791.1
Hymenoptera	Chalcidoidea	Pteromalidae	Jaliscoa	grandis	MW982458.1
Hymenoptera	Chalcidoidea	Pteromalidae	Janssoniella	caudata	MW981743.1
Hymenoptera	Chalcidoidea	Pteromalidae	Janssoniella	caudata	MW983905.1
Hymenoptera	Chalcidoidea	Pteromalidae	Kaleva	corynocera	OL538058.1
Hymenoptera	Chalcidoidea	Pteromalidae	Kaleva	corynocera	OL538079.1

Hymenoptera	Chalcidoidea	Pteromalidae	Kaleva	corynocera	OL538085.1
Hymenoptera	Chalcidoidea	Pteromalidae	Kaleva	corynocera	OL538138.1
Hymenoptera	Chalcidoidea	Pteromalidae	Ksenoplata	quadrata	OL538087.1
Hymenoptera	Chalcidoidea	Pteromalidae	Ksenoplata	quadrata	OL538130.1
Hymenoptera	Chalcidoidea	Pteromalidae	Lariophagus	texanus	MW981983.1
Hymenoptera	Chalcidoidea	Pteromalidae	Lipothymus	sp.	MK530786.1
Hymenoptera	Chalcidoidea	Pteromalidae	Lyrcus	nigroaeneus	MG375445.1
Hymenoptera	Chalcidoidea	Pteromalidae	Lyrcus	perdubius	KR785755.1
Hymenoptera	Chalcidoidea	Pteromalidae	Lyrcus	perdubius	KR789666.1
Hymenoptera	Chalcidoidea	Pteromalidae	Lyrcus	sp.	MG377599.1
Hymenoptera	Chalcidoidea	Pteromalidae	Lyrcus	sp.	MW784386.1
Hymenoptera	Chalcidoidea	Pteromalidae	Macroglenes	eximius	OL538080.1
Hymenoptera	Chalcidoidea	Pteromalidae	Macroglenes	paludum	OL538091.1
Hymenoptera	Chalcidoidea	Pteromalidae	Merismus	megapterus	MW983657.1
Hymenoptera	Chalcidoidea	Pteromalidae	Mesopolobus	bruchophagi	KR881600.1
Hymenoptera	Chalcidoidea	Pteromalidae	Mesopolobus	bruchophagi	KR891474.1
Hymenoptera	Chalcidoidea	Pteromalidae	Mesopolobus	bruchophagi	KR892877.1
Hymenoptera	Chalcidoidea	Pteromalidae	Mesopolobus	fasciiventris	MZ630194.1
Hymenoptera	Chalcidoidea	Pteromalidae	Mesopolobus	fasciiventris	MZ656851.1
Hymenoptera	Chalcidoidea	Pteromalidae	Mesopolobus	incultus	MZ626366.1
Hymenoptera	Chalcidoidea	Pteromalidae	Mesopolobus	sp.	JN292344.1
Hymenoptera	Chalcidoidea	Pteromalidae	Mesopolobus	sp.	JN293206.1
Hymenoptera	Chalcidoidea	Pteromalidae	Mesopolobus	sp.	KM555996.1
Hymenoptera	Chalcidoidea	Pteromalidae	Mesopolobus	sp.	KM557309.1
Hymenoptera	Chalcidoidea	Pteromalidae	Mesopolobus	sp.	KM557597.1
Hymenoptera	Chalcidoidea	Pteromalidae	Mesopolobus	sp.	KM560542.1
Hymenoptera	Chalcidoidea	Pteromalidae	Mesopolobus	sp.	KM563672.1
Hymenoptera	Chalcidoidea	Pteromalidae	Mesopolobus	sp.	KM565014.1
Hymenoptera	Chalcidoidea	Pteromalidae	Mesopolobus	sp.	KM568108.1
Hymenoptera	Chalcidoidea	Pteromalidae	Mesopolobus	sp.	KM568955.1
Hymenoptera	Chalcidoidea	Pteromalidae	Mesopolobus	sp.	KR786412.1
Hymenoptera	Chalcidoidea	Pteromalidae	Mesopolobus	sp.	MF098549.1
Hymenoptera	Chalcidoidea	Pteromalidae	Mesopolobus	tortricidis	HQ107442.1
Hymenoptera	Chalcidoidea	Pteromalidae	Mesopolobus	tortricis	HQ107446.1
Hymenoptera	Chalcidoidea	Pteromalidae	Mesopolobus	tortricis	HQ107450.1
Hymenoptera	Chalcidoidea	Pteromalidae	Mesopolobus	tortricis	HQ107451.1
Hymenoptera	Chalcidoidea	Pteromalidae	Mesopolobus	tortricis	HQ107452.1
Hymenoptera	Chalcidoidea	Pteromalidae	Mesopolobus	tortricis	HQ107453.1
Hymenoptera	Chalcidoidea	Pteromalidae	Mesopolobus	tortricis	HQ107454.1
Hymenoptera	Chalcidoidea	Pteromalidae	Mesopolobus	tortricis	HQ107455.1
Hymenoptera	Chalcidoidea	Pteromalidae	Mesopolobus	tortricis	HQ107462.1
Hymenoptera	Chalcidoidea	Pteromalidae	Mesopolobus	tortricis	HQ107464.1
Hymenoptera	Chalcidoidea	Pteromalidae	Mesopolobus	tortricis	HQ107465.1
Hymenoptera	Chalcidoidea	Pteromalidae	Mesopolobus	tortricis	HQ107468.1
Hymenoptera	Chalcidoidea	Pteromalidae	Mesopolobus	tortricis	HQ107471.1
Hymenoptera	Chalcidoidea	Pteromalidae	Mesopolobus	tortricis	KM563161.1
Hymenoptera	Chalcidoidea	Pteromalidae	Mesopolobus	tortricis	KM566235.1
Hymenoptera	Chalcidoidea	Pteromalidae	Mesopolobus	tortricis	MG374023.1
Hymenoptera	Chalcidoidea	Pteromalidae	Mesopolobus	verditer	HQ107472.1
Hymenoptera	Chalcidoidea	Pteromalidae	Mesopolobus	verditer	HQ107473.1
Hymenoptera	Chalcidoidea	Pteromalidae	Mesopolobus	verditer	HQ107474.1
Hymenoptera	Chalcidoidea	Pteromalidae	Mesopolobus	verditer	HQ107475.1
Hymenoptera	Chalcidoidea	Pteromalidae	Mesopolobus	verditer	HQ107476.1
Hymenoptera	Chalcidoidea	Pteromalidae	Mesopolobus	verditer	HQ107477.1

Hymenoptera	Chalcidoidea	Pteromalidae	Mesopolobus	verditer	HQ107479.1
Hymenoptera	Chalcidoidea	Pteromalidae	Mesopolobus	verditer	HQ107481.1
Hymenoptera	Chalcidoidea	Pteromalidae	Mesopolobus	verditer	HQ107482.1
Hymenoptera	Chalcidoidea	Pteromalidae	Mesopolobus	verditer	HQ107483.1
Hymenoptera	Chalcidoidea	Pteromalidae	Mesopolobus	verditer	HQ107484.1
Hymenoptera	Chalcidoidea	Pteromalidae	Mesopolobus	verditer	HQ107485.1
Hymenoptera	Chalcidoidea	Pteromalidae	Mesopolobus	verditer	HQ107492.1
Hymenoptera	Chalcidoidea	Pteromalidae	Mesopolobus	verditer	HQ107494.1
Hymenoptera	Chalcidoidea	Pteromalidae	Mesopolobus	verditer	HQ107495.1
Hymenoptera	Chalcidoidea	Pteromalidae	Mesopolobus	verditer	HQ107498.1
Hymenoptera	Chalcidoidea	Pteromalidae	Mesopolobus	verditer	HQ107500.1
Hymenoptera	Chalcidoidea	Pteromalidae	Mesopolobus	verditer	HQ107504.1
Hymenoptera	Chalcidoidea	Pteromalidae	Mesopolobus	verditer	HQ107505.1
Hymenoptera	Chalcidoidea	Pteromalidae	Mesopolobus	verditer	HQ107506.1
Hymenoptera	Chalcidoidea	Pteromalidae	Mesopolobus	verditer	HQ107507.1
Hymenoptera	Chalcidoidea	Pteromalidae	Mesopolobus	verditer	HQ107514.1
Hymenoptera	Chalcidoidea	Pteromalidae	Mesopolobus	verditer	HQ107516.1
Hymenoptera	Chalcidoidea	Pteromalidae	Mesopolobus	verditer	HQ107517.1
Hymenoptera	Chalcidoidea	Pteromalidae	Mesopolobus	verditer	HQ107520.1
Hymenoptera	Chalcidoidea	Pteromalidae	Mesopolobus	verditer	HQ107523.1
Hymenoptera	Chalcidoidea	Pteromalidae	Mesopolobus	verditer	HQ107524.1
Hymenoptera	Chalcidoidea	Pteromalidae	Mesopolobus	verditer	HQ107529.1
Hymenoptera	Chalcidoidea	Pteromalidae	Mesopolobus	verditer	HQ107532.1
Hymenoptera	Chalcidoidea	Pteromalidae	Mesopolobus	verditer	HQ107533.1
Hymenoptera	Chalcidoidea	Pteromalidae	Mesopolobus	verditer	HQ107534.1
Hymenoptera	Chalcidoidea	Pteromalidae	Mesopolobus	verditer	HQ107535.1
Hymenoptera	Chalcidoidea	Pteromalidae	Mesopolobus	verditer	HQ107536.1
Hymenoptera	Chalcidoidea	Pteromalidae	Mesopolobus	verditer	HQ107541.1
Hymenoptera	Chalcidoidea	Pteromalidae	Mesopolobus	verditer	HQ107543.1
Hymenoptera	Chalcidoidea	Pteromalidae	Mesopolobus	verditer	HQ107545.1
Hymenoptera	Chalcidoidea	Pteromalidae	Mesopolobus	verditer	HQ107546.1
Hymenoptera	Chalcidoidea	Pteromalidae	Mesopolobus	verditer	HQ107547.1
Hymenoptera	Chalcidoidea	Pteromalidae	Mesopolobus	verditer	HQ107550.1
Hymenoptera	Chalcidoidea	Pteromalidae	Mesopolobus	verditer	HQ107551.1
Hymenoptera	Chalcidoidea	Pteromalidae	Mesopolobus	verditer	HQ107555.1
Hymenoptera	Chalcidoidea	Pteromalidae	Mesopolobus	verditer	HQ107561.1
Hymenoptera	Chalcidoidea	Pteromalidae	Mesopolobus	verditer	HQ107562.1
Hymenoptera	Chalcidoidea	Pteromalidae	Mesopolobus	verditer	HQ107563.1
Hymenoptera	Chalcidoidea	Pteromalidae	Mesopolobus	verditer	HQ107568.1
Hymenoptera	Chalcidoidea	Pteromalidae	Mesopolobus	verditer	HQ107570.1
Hymenoptera	Chalcidoidea	Pteromalidae	Mesopolobus	verditer	HQ107574.1
Hymenoptera	Chalcidoidea	Pteromalidae	Mesopolobus	verditer	KR370664.1
Hymenoptera	Chalcidoidea	Pteromalidae	Mesopolobus	verditer	KU496780.1
Hymenoptera	Chalcidoidea	Pteromalidae	Micradelus	acutus	OL538097.1
Hymenoptera	Chalcidoidea	Pteromalidae	Micranisa	degastris	JQ756554.1
Hymenoptera	Chalcidoidea	Pteromalidae	Micranisa	ralianga	MK530759.1
Hymenoptera	Chalcidoidea	Pteromalidae	Micranisa	sp.	MK530739.1
Hymenoptera	Chalcidoidea	Pteromalidae	Micranisa	sp.	MK530740.1
Hymenoptera	Chalcidoidea	Pteromalidae	Micranisa	sp.	MK530744.1
Hymenoptera	Chalcidoidea	Pteromalidae	Micranisa	sp.	MK530746.1
Hymenoptera	Chalcidoidea	Pteromalidae	Micranisa	sp.	MK530749.1
Hymenoptera	Chalcidoidea	Pteromalidae	Micranisa	sp.	MK530752.1
Hymenoptera	Chalcidoidea	Pteromalidae	Micranisa	sp.	MK530753.1
Hymenoptera	Chalcidoidea	Pteromalidae	Micranisa	sp.	MK530754.1

Hymenoptera	Chalcidoidea	Pteromalidae	Micranisa	sp.	MK530755.1
Hymenoptera	Chalcidoidea	Pteromalidae	Micranisa	sp.	MK530756.1
Hymenoptera	Chalcidoidea	Pteromalidae	Micranisa	sp.	MK530758.1
Hymenoptera	Chalcidoidea	Pteromalidae	Micranisa	sp.	MK530760.1
Hymenoptera	Chalcidoidea	Pteromalidae	Nasonia	vitripeis	KR887518.1
Hymenoptera	Chalcidoidea	Pteromalidae	Neocalosoter	pityophthori	KP119775.1
Hymenoptera	Chalcidoidea	Pteromalidae	Neosycophila	omeomorpha	MK530728.1
Hymenoptera	Chalcidoidea	Pteromalidae	Neosycophila	omeomorpha	MK530729.1
Hymenoptera	Chalcidoidea	Pteromalidae	Neosycophila	omeomorpha	MK530730.1
Hymenoptera	Chalcidoidea	Pteromalidae	Neosycophila	omeomorpha	MK530733.1
Hymenoptera	Chalcidoidea	Pteromalidae	Odontofroggatia	corneri	MK530734.1
Hymenoptera	Chalcidoidea	Pteromalidae	Odontofroggatia	corneri	MK530735.1
Hymenoptera	Chalcidoidea	Pteromalidae	Odontofroggatia	corneri	MK530736.1
Hymenoptera	Chalcidoidea	Pteromalidae	Odontofroggatia	galili	MK530738.1
Hymenoptera	Chalcidoidea	Pteromalidae	Odontofroggatia	sp.	HM770633.1
Hymenoptera	Chalcidoidea	Pteromalidae	Oxysychus	sp.	MW983948.1
Hymenoptera	Chalcidoidea	Pteromalidae	Pachycrepoideus	sp.	JQ808449.1
Hymenoptera	Chalcidoidea	Pteromalidae	Pachycrepoideus	vindemmiae	MG831937.1
Hymenoptera	Chalcidoidea	Pteromalidae	Pachycrepoideus	vindemmiae	MG831938.1
Hymenoptera	Chalcidoidea	Pteromalidae	Pachycrepoideus	vindemmiae	MK813907.1
Hymenoptera	Chalcidoidea	Pteromalidae	Pachyneuron	aphidis	JF906503.1
Hymenoptera	Chalcidoidea	Pteromalidae	Pachyneuron	aphidis	JX507457.1
Hymenoptera	Chalcidoidea	Pteromalidae	Pachyneuron	aphidis	KY832053.1
Hymenoptera	Chalcidoidea	Pteromalidae	Pachyneuron	aphidis	KY833161.1
Hymenoptera	Chalcidoidea	Pteromalidae	Pachyneuron	aphidis	KY834259.1
Hymenoptera	Chalcidoidea	Pteromalidae	Pachyneuron	aphidis	KY835322.1
Hymenoptera	Chalcidoidea	Pteromalidae	Pachyneuron	aphidis	KY841405.1
Hymenoptera	Chalcidoidea	Pteromalidae	Pachyneuron	aphidis	KY842618.1
Hymenoptera	Chalcidoidea	Pteromalidae	Pachyneuron	aphidis	KY844368.1
Hymenoptera	Chalcidoidea	Pteromalidae	Pachyneuron	aphidis	KY845889.1
Hymenoptera	Chalcidoidea	Pteromalidae	Pachyneuron	aphidis	KY847314.1
Hymenoptera	Chalcidoidea	Pteromalidae	Pachyneuron	aphidis	KY912686.1
Hymenoptera	Chalcidoidea	Pteromalidae	Pachyneuron	aphidis	KY912687.1
Hymenoptera	Chalcidoidea	Pteromalidae	Pachyneuron	aphidis	KY912688.1
Hymenoptera	Chalcidoidea	Pteromalidae	Pachyneuron	aphidis	LC260606.1
Hymenoptera	Chalcidoidea	Pteromalidae	Pachyneuron	aphidis	MF979489.1
Hymenoptera	Chalcidoidea	Pteromalidae	Pachyneuron	aphidis	MF979490.1
Hymenoptera	Chalcidoidea	Pteromalidae	Pachyneuron	aphidis	MF979491.1
Hymenoptera	Chalcidoidea	Pteromalidae	Pachyneuron	aphidis	MF979493.1
Hymenoptera	Chalcidoidea	Pteromalidae	Pachyneuron	aphidis	MF979496.1
Hymenoptera	Chalcidoidea	Pteromalidae	Pachyneuron	aphidis	MF979497.1
Hymenoptera	Chalcidoidea	Pteromalidae	Pachyneuron	aphidis	MK796245.1
Hymenoptera	Chalcidoidea	Pteromalidae	Pachyneuron	groenlandicum	KM558412.1
Hymenoptera	Chalcidoidea	Pteromalidae	Pachyneuron	groenlandicum	KU373782.1
Hymenoptera	Chalcidoidea	Pteromalidae	Pachyneuron	groenlandicum	KU373856.1
Hymenoptera	Chalcidoidea	Pteromalidae	Pachyneuron	groenlandicum	MG379195.1
Hymenoptera	Chalcidoidea	Pteromalidae	Pachyneuron	groenlandicum	MG379686.1
Hymenoptera	Chalcidoidea	Pteromalidae	Pachyneuron	groenlandicum	MN667214.1
Hymenoptera	Chalcidoidea	Pteromalidae	Pachyneuron	groenlandicum	MN667490.1
Hymenoptera	Chalcidoidea	Pteromalidae	Pachyneuron	groenlandicum	MN670668.1
Hymenoptera	Chalcidoidea	Pteromalidae	Pachyneuron	groenlandicum	MN673697.1
Hymenoptera	Chalcidoidea	Pteromalidae	Pachyneuron	groenlandicum	MN675192.1
Hymenoptera	Chalcidoidea	Pteromalidae	Pachyneuron	groenlandicum	MN679507.1
Hymenoptera	Chalcidoidea	Pteromalidae	Pachyneuron	groenlandicum	MN681150.1

Hymenoptera	Chalcidoidea	Pteromalidae	Pachyneuron	groenlandicum	MN682251.1
Hymenoptera	Chalcidoidea	Pteromalidae	Pachyneuron	groenlandicum	MN682850.1
Hymenoptera	Chalcidoidea	Pteromalidae	Pachyneuron	muscarum	KY912693.1
Hymenoptera	Chalcidoidea	Pteromalidae	Pachyneuron	solitarium	KY912696.1
Hymenoptera	Chalcidoidea	Pteromalidae	Pachyneuron	sp.	HQ599572.1
Hymenoptera	Chalcidoidea	Pteromalidae	Pachyneuron	sp.	HQ930387.1
Hymenoptera	Chalcidoidea	Pteromalidae	Pachyneuron	sp.	HQ930392.1
Hymenoptera	Chalcidoidea	Pteromalidae	Pachyneuron	sp.	KF894412.1
Hymenoptera	Chalcidoidea	Pteromalidae	Pachyneuron	sp.	KM557931.1
Hymenoptera	Chalcidoidea	Pteromalidae	Pachyneuron	sp.	KM564390.1
Hymenoptera	Chalcidoidea	Pteromalidae	Pachyneuron	sp.	KR366368.1
Hymenoptera	Chalcidoidea	Pteromalidae	Pachyneuron	sp.	KR367718.1
Hymenoptera	Chalcidoidea	Pteromalidae	Pachyneuron	sp.	KR369519.1
Hymenoptera	Chalcidoidea	Pteromalidae	Pachyneuron	sp.	KR372426.1
Hymenoptera	Chalcidoidea	Pteromalidae	Pachyneuron	sp.	KR372612.1
Hymenoptera	Chalcidoidea	Pteromalidae	Pachyneuron	sp.	KR879872.1
Hymenoptera	Chalcidoidea	Pteromalidae	Pachyneuron	sp.	KR927575.1
Hymenoptera	Chalcidoidea	Pteromalidae	Pachyneuron	sp.	KY754680.1
Hymenoptera	Chalcidoidea	Pteromalidae	Pachyneuron	sp.	MF807206.1
Hymenoptera	Chalcidoidea	Pteromalidae	Pachyneuron	sp.	MG376856.1
Hymenoptera	Chalcidoidea	Pteromalidae	Pachyneuron	sp.	MG378360.1
Hymenoptera	Chalcidoidea	Pteromalidae	Pachyneuron	sp.	MG382118.1
Hymenoptera	Chalcidoidea	Pteromalidae	Pachyneuron	sp.	MG498754.1
Hymenoptera	Chalcidoidea	Pteromalidae	Pachyneuron	sp.	MG501797.1
Hymenoptera	Chalcidoidea	Pteromalidae	Pachyneuron	sp.	MN668024.1
Hymenoptera	Chalcidoidea	Pteromalidae	Pachyneuron	sp.	MN668494.1
Hymenoptera	Chalcidoidea	Pteromalidae	Pachyneuron	sp.	MN668613.1
Hymenoptera	Chalcidoidea	Pteromalidae	Pachyneuron	sp.	MN669638.1
Hymenoptera	Chalcidoidea	Pteromalidae	Pachyneuron	sp.	MN670252.1
Hymenoptera	Chalcidoidea	Pteromalidae	Pachyneuron	sp.	MN672683.1
Hymenoptera	Chalcidoidea	Pteromalidae	Pachyneuron	sp.	MN673316.1
Hymenoptera	Chalcidoidea	Pteromalidae	Pachyneuron	sp.	MN674557.1
Hymenoptera	Chalcidoidea	Pteromalidae	Pachyneuron	sp.	MN676201.1
Hymenoptera	Chalcidoidea	Pteromalidae	Pachyneuron	sp.	MN676963.1
Hymenoptera	Chalcidoidea	Pteromalidae	Pachyneuron	sp.	MN677019.1
Hymenoptera	Chalcidoidea	Pteromalidae	Pachyneuron	sp.	MN679869.1
Hymenoptera	Chalcidoidea	Pteromalidae	Pachyneuron	sp.	MN680562.1
Hymenoptera	Chalcidoidea	Pteromalidae	Pachyneuron	sp.	MN683239.1
Hymenoptera	Chalcidoidea	Pteromalidae	Pachyneuron	sp.	MZ633294.1
Hymenoptera	Chalcidoidea	Pteromalidae	Panstenon	poaphilum	MW982571.1
Hymenoptera	Chalcidoidea	Pteromalidae	Pegopus	sp.	MW983034.1
Hymenoptera	Chalcidoidea	Pteromalidae	Pegopus	sp.	MW984100.1
Hymenoptera	Chalcidoidea	Pteromalidae	Philocaenus	sp.	JQ756584.1
Hymenoptera	Chalcidoidea	Pteromalidae	Philotrypesis	pilosa	KF778383.1
Hymenoptera	Chalcidoidea	Pteromalidae	Philotrypesis	pilosa	KF778384.1
Hymenoptera	Chalcidoidea	Pteromalidae	Philotrypesis	sp.	KF778392.1
Hymenoptera	Chalcidoidea	Pteromalidae	Philotrypesis	sp.	KF778393.1
Hymenoptera	Chalcidoidea	Pteromalidae	Philotrypesis	sp.	KF778394.1
Hymenoptera	Chalcidoidea	Pteromalidae	Philotrypesis	sp.	KM557211.1
Hymenoptera	Chalcidoidea	Pteromalidae	Philotrypesis	sp.	KM565788.1
Hymenoptera	Chalcidoidea	Pteromalidae	Philotrypesis	sp.	KM565922.1
Hymenoptera	Chalcidoidea	Pteromalidae	Philotrypesis	sp.	KR785606.1
Hymenoptera	Chalcidoidea	Pteromalidae	Platygerrhus	unicolor	OL538104.1
Hymenoptera	Chalcidoidea	Pteromalidae	Psilocera	confusa	OL538117.1

Hymenoptera	Chalcidoidea	Pteromalidae	Psilocera	crassispina	OL538056.1
Hymenoptera	Chalcidoidea	Pteromalidae	Psilocera	crassispina	OL538070.1
Hymenoptera	Chalcidoidea	Pteromalidae	Psilocera	crassispina	OL538113.1
Hymenoptera	Chalcidoidea	Pteromalidae	Psilocera	crassispina	OL538114.1
Hymenoptera	Chalcidoidea	Pteromalidae	Psilocera	crassispina	OL538135.1
Hymenoptera	Chalcidoidea	Pteromalidae	Psilocera	crassispina	OL538137.1
Hymenoptera	Chalcidoidea	Pteromalidae	Psilocera	crassispina	OL538139.1
Hymenoptera	Chalcidoidea	Pteromalidae	Psilocera	crassispina	OL538153.1
Hymenoptera	Chalcidoidea	Pteromalidae	Psilocera	crassispina	OL538155.1
Hymenoptera	Chalcidoidea	Pteromalidae	Psychophagoides	crassicornis	OL538123.1
Hymenoptera	Chalcidoidea	Pteromalidae	Psychophagus	omnivorus	MN477289.1
Hymenoptera	Chalcidoidea	Pteromalidae	Psychophagus	omnivorus	MN477290.1
Hymenoptera	Chalcidoidea	Pteromalidae	Pteromalidae	sp.	GU675355.1
Hymenoptera	Chalcidoidea	Pteromalidae	Pteromalidae	sp.	GU675358.1
Hymenoptera	Chalcidoidea	Pteromalidae	Pteromalidae	sp.	GU675361.1
Hymenoptera	Chalcidoidea	Pteromalidae	Pteromalidae	sp.	GU675362.1
Hymenoptera	Chalcidoidea	Pteromalidae	Pteromalidae	sp.	GU675363.1
Hymenoptera	Chalcidoidea	Pteromalidae	Pteromalidae	sp.	HM374692.1
Hymenoptera	Chalcidoidea	Pteromalidae	Pteromalidae	sp.	HM374693.1
Hymenoptera	Chalcidoidea	Pteromalidae	Pteromalidae	sp.	HM374740.1
Hymenoptera	Chalcidoidea	Pteromalidae	Pteromalidae	sp.	HM374785.1
Hymenoptera	Chalcidoidea	Pteromalidae	Pteromalidae	sp.	HM374787.1
Hymenoptera	Chalcidoidea	Pteromalidae	Pteromalidae	sp.	HM394866.1
Hymenoptera	Chalcidoidea	Pteromalidae	Pteromalidae	sp.	HM414445.1
Hymenoptera	Chalcidoidea	Pteromalidae	Pteromalidae	sp.	HM414504.1
Hymenoptera	Chalcidoidea	Pteromalidae	Pteromalidae	sp.	HM414675.1
Hymenoptera	Chalcidoidea	Pteromalidae	Pteromalidae	sp.	HM423351.1
Hymenoptera	Chalcidoidea	Pteromalidae	Pteromalidae	sp.	HM423352.1
Hymenoptera	Chalcidoidea	Pteromalidae	Pteromalidae	sp.	HM423399.1
Hymenoptera	Chalcidoidea	Pteromalidae	Pteromalidae	sp.	HM432863.1
Hymenoptera	Chalcidoidea	Pteromalidae	Pteromalidae	sp.	HM432908.1
Hymenoptera	Chalcidoidea	Pteromalidae	Pteromalidae	sp.	HM432910.1
Hymenoptera	Chalcidoidea	Pteromalidae	Pteromalidae	sp.	HM432912.1
Hymenoptera	Chalcidoidea	Pteromalidae	Pteromalidae	sp.	HM432913.1
Hymenoptera	Chalcidoidea	Pteromalidae	Pteromalidae	sp.	HM432914.1
Hymenoptera	Chalcidoidea	Pteromalidae	Pteromalidae	sp.	HM883319.1
Hymenoptera	Chalcidoidea	Pteromalidae	Pteromalidae	sp.	HQ927782.1
Hymenoptera	Chalcidoidea	Pteromalidae	Pteromalidae	sp.	HQ927784.1
Hymenoptera	Chalcidoidea	Pteromalidae	Pteromalidae	sp.	HQ927786.1
Hymenoptera	Chalcidoidea	Pteromalidae	Pteromalidae	sp.	HQ927811.1
Hymenoptera	Chalcidoidea	Pteromalidae	Pteromalidae	sp.	HQ929591.1
Hymenoptera	Chalcidoidea	Pteromalidae	Pteromalidae	sp.	HQ929598.1
Hymenoptera	Chalcidoidea	Pteromalidae	Pteromalidae	sp.	HQ929599.1
Hymenoptera	Chalcidoidea	Pteromalidae	Pteromalidae	sp.	HQ929604.1
Hymenoptera	Chalcidoidea	Pteromalidae	Pteromalidae	sp.	HQ929606.1
Hymenoptera	Chalcidoidea	Pteromalidae	Pteromalidae	sp.	HQ929608.1
Hymenoptera	Chalcidoidea	Pteromalidae	Pteromalidae	sp.	HQ929614.1
Hymenoptera	Chalcidoidea	Pteromalidae	Pteromalidae	sp.	HQ929634.1
Hymenoptera	Chalcidoidea	Pteromalidae	Pteromalidae	sp.	HQ929645.1
Hymenoptera	Chalcidoidea	Pteromalidae	Pteromalidae	sp.	HQ929646.1
Hymenoptera	Chalcidoidea	Pteromalidae	Pteromalidae	sp.	HQ929771.1
Hymenoptera	Chalcidoidea	Pteromalidae	Pteromalidae	sp.	HQ929805.1
Hymenoptera	Chalcidoidea	Pteromalidae	Pteromalidae	sp.	HQ929810.1
Hymenoptera	Chalcidoidea	Pteromalidae	Pteromalidae	sp.	HQ929812.1

Hymenoptera	Chalcidoidea	Pteromalidae	Pteromalidae	sp.	HQ930262.1
Hymenoptera	Chalcidoidea	Pteromalidae	Pteromalidae	sp.	HQ930291.1
Hymenoptera	Chalcidoidea	Pteromalidae	Pteromalidae	sp.	HQ930301.1
Hymenoptera	Chalcidoidea	Pteromalidae	Pteromalidae	sp.	HQ961291.1
Hymenoptera	Chalcidoidea	Pteromalidae	Pteromalidae	sp.	HQ961293.1
Hymenoptera	Chalcidoidea	Pteromalidae	Pteromalidae	sp.	HQ972322.1
Hymenoptera	Chalcidoidea	Pteromalidae	Pteromalidae	sp.	HQ972350.1
Hymenoptera	Chalcidoidea	Pteromalidae	Pteromalidae	sp.	HQ989983.1
Hymenoptera	Chalcidoidea	Pteromalidae	Pteromalidae	sp.	HQ989991.1
Hymenoptera	Chalcidoidea	Pteromalidae	Pteromalidae	sp.	JN292242.1
Hymenoptera	Chalcidoidea	Pteromalidae	Pteromalidae	sp.	JN292466.1
Hymenoptera	Chalcidoidea	Pteromalidae	Pteromalidae	sp.	JN292467.1
Hymenoptera	Chalcidoidea	Pteromalidae	Pteromalidae	sp.	JN293065.1
Hymenoptera	Chalcidoidea	Pteromalidae	Pteromalidae	sp.	JN293138.1
Hymenoptera	Chalcidoidea	Pteromalidae	Pteromalidae	sp.	JN293139.1
Hymenoptera	Chalcidoidea	Pteromalidae	Pteromalidae	sp.	JN293243.1
Hymenoptera	Chalcidoidea	Pteromalidae	Pteromalidae	sp.	JN293247.1
Hymenoptera	Chalcidoidea	Pteromalidae	Pteromalidae	sp.	JN293274.1
Hymenoptera	Chalcidoidea	Pteromalidae	Pteromalidae	sp.	JN293498.1
Hymenoptera	Chalcidoidea	Pteromalidae	Pteromalidae	sp.	JX829818.1
Hymenoptera	Chalcidoidea	Pteromalidae	Pteromalidae	sp.	JX832119.1
Hymenoptera	Chalcidoidea	Pteromalidae	Pteromalidae	sp.	KJ083281.1
Hymenoptera	Chalcidoidea	Pteromalidae	Pteromalidae	sp.	KJ083955.1
Hymenoptera	Chalcidoidea	Pteromalidae	Pteromalidae	sp.	KJ085373.1
Hymenoptera	Chalcidoidea	Pteromalidae	Pteromalidae	sp.	KJ087727.1
Hymenoptera	Chalcidoidea	Pteromalidae	Pteromalidae	sp.	KJ088204.1
Hymenoptera	Chalcidoidea	Pteromalidae	Pteromalidae	sp.	KJ089078.1
Hymenoptera	Chalcidoidea	Pteromalidae	Pteromalidae	sp.	KJ090305.1
Hymenoptera	Chalcidoidea	Pteromalidae	Pteromalidae	sp.	KJ166182.1
Hymenoptera	Chalcidoidea	Pteromalidae	Pteromalidae	sp.	KJ166673.1
Hymenoptera	Chalcidoidea	Pteromalidae	Pteromalidae	sp.	KJ209229.1
Hymenoptera	Chalcidoidea	Pteromalidae	Pteromalidae	sp.	KM555429.1
Hymenoptera	Chalcidoidea	Pteromalidae	Pteromalidae	sp.	KM555489.1
Hymenoptera	Chalcidoidea	Pteromalidae	Pteromalidae	sp.	KM555652.1
Hymenoptera	Chalcidoidea	Pteromalidae	Pteromalidae	sp.	KM555694.1
Hymenoptera	Chalcidoidea	Pteromalidae	Pteromalidae	sp.	KM555784.1
Hymenoptera	Chalcidoidea	Pteromalidae	Pteromalidae	sp.	KM555823.1
Hymenoptera	Chalcidoidea	Pteromalidae	Pteromalidae	sp.	KM556014.1
Hymenoptera	Chalcidoidea	Pteromalidae	Pteromalidae	sp.	KM556099.1
Hymenoptera	Chalcidoidea	Pteromalidae	Pteromalidae	sp.	KM556110.1
Hymenoptera	Chalcidoidea	Pteromalidae	Pteromalidae	sp.	KM556114.1
Hymenoptera	Chalcidoidea	Pteromalidae	Pteromalidae	sp.	KM556128.1
Hymenoptera	Chalcidoidea	Pteromalidae	Pteromalidae	sp.	KM556206.1
Hymenoptera	Chalcidoidea	Pteromalidae	Pteromalidae	sp.	KM556399.1
Hymenoptera	Chalcidoidea	Pteromalidae	Pteromalidae	sp.	KM556525.1
Hymenoptera	Chalcidoidea	Pteromalidae	Pteromalidae	sp.	KM556563.1
Hymenoptera	Chalcidoidea	Pteromalidae	Pteromalidae	sp.	KM556579.1
Hymenoptera	Chalcidoidea	Pteromalidae	Pteromalidae	sp.	KM556632.1
Hymenoptera	Chalcidoidea	Pteromalidae	Pteromalidae	sp.	KM556652.1
Hymenoptera	Chalcidoidea	Pteromalidae	Pteromalidae	sp.	KM556658.1
Hymenoptera	Chalcidoidea	Pteromalidae	Pteromalidae	sp.	KM556708.1
Hymenoptera	Chalcidoidea	Pteromalidae	Pteromalidae	sp.	KM556849.1
Hymenoptera	Chalcidoidea	Pteromalidae	Pteromalidae	sp.	KM556852.1
Hymenoptera	Chalcidoidea	Pteromalidae	Pteromalidae	sp.	KM556956.1

Hymenoptera	Chalcidoidea	Pteromalidae	Pteromalidae	sp.	KM556983.1
Hymenoptera	Chalcidoidea	Pteromalidae	Pteromalidae	sp.	KM557039.1
Hymenoptera	Chalcidoidea	Pteromalidae	Pteromalidae	sp.	KM557100.1
Hymenoptera	Chalcidoidea	Pteromalidae	Pteromalidae	sp.	KM557293.1
Hymenoptera	Chalcidoidea	Pteromalidae	Pteromalidae	sp.	KM557305.1
Hymenoptera	Chalcidoidea	Pteromalidae	Pteromalidae	sp.	KM557387.1
Hymenoptera	Chalcidoidea	Pteromalidae	Pteromalidae	sp.	KM557477.1
Hymenoptera	Chalcidoidea	Pteromalidae	Pteromalidae	sp.	KM557511.1
Hymenoptera	Chalcidoidea	Pteromalidae	Pteromalidae	sp.	KM557513.1
Hymenoptera	Chalcidoidea	Pteromalidae	Pteromalidae	sp.	KM557573.1
Hymenoptera	Chalcidoidea	Pteromalidae	Pteromalidae	sp.	KM557644.1
Hymenoptera	Chalcidoidea	Pteromalidae	Pteromalidae	sp.	KM557722.1
Hymenoptera	Chalcidoidea	Pteromalidae	Pteromalidae	sp.	KM557786.1
Hymenoptera	Chalcidoidea	Pteromalidae	Pteromalidae	sp.	KM557867.1
Hymenoptera	Chalcidoidea	Pteromalidae	Pteromalidae	sp.	KM558103.1
Hymenoptera	Chalcidoidea	Pteromalidae	Pteromalidae	sp.	KM558188.1
Hymenoptera	Chalcidoidea	Pteromalidae	Pteromalidae	sp.	KM558356.1
Hymenoptera	Chalcidoidea	Pteromalidae	Pteromalidae	sp.	KM558469.1
Hymenoptera	Chalcidoidea	Pteromalidae	Pteromalidae	sp.	KM558570.1
Hymenoptera	Chalcidoidea	Pteromalidae	Pteromalidae	sp.	KM558580.1
Hymenoptera	Chalcidoidea	Pteromalidae	Pteromalidae	sp.	KM558602.1
Hymenoptera	Chalcidoidea	Pteromalidae	Pteromalidae	sp.	KM558606.1
Hymenoptera	Chalcidoidea	Pteromalidae	Pteromalidae	sp.	KM558628.1
Hymenoptera	Chalcidoidea	Pteromalidae	Pteromalidae	sp.	KM558670.1
Hymenoptera	Chalcidoidea	Pteromalidae	Pteromalidae	sp.	KM558688.1
Hymenoptera	Chalcidoidea	Pteromalidae	Pteromalidae	sp.	KM558946.1
Hymenoptera	Chalcidoidea	Pteromalidae	Pteromalidae	sp.	KM559203.1
Hymenoptera	Chalcidoidea	Pteromalidae	Pteromalidae	sp.	KM559405.1
Hymenoptera	Chalcidoidea	Pteromalidae	Pteromalidae	sp.	KM559501.1
Hymenoptera	Chalcidoidea	Pteromalidae	Pteromalidae	sp.	KM559525.1
Hymenoptera	Chalcidoidea	Pteromalidae	Pteromalidae	sp.	KM559580.1
Hymenoptera	Chalcidoidea	Pteromalidae	Pteromalidae	sp.	KM559971.1
Hymenoptera	Chalcidoidea	Pteromalidae	Pteromalidae	sp.	KM559977.1
Hymenoptera	Chalcidoidea	Pteromalidae	Pteromalidae	sp.	KM559987.1
Hymenoptera	Chalcidoidea	Pteromalidae	Pteromalidae	sp.	KM560007.1
Hymenoptera	Chalcidoidea	Pteromalidae	Pteromalidae	sp.	KM560041.1
Hymenoptera	Chalcidoidea	Pteromalidae	Pteromalidae	sp.	KM560112.1
Hymenoptera	Chalcidoidea	Pteromalidae	Pteromalidae	sp.	KM560258.1
Hymenoptera	Chalcidoidea	Pteromalidae	Pteromalidae	sp.	KM560294.1
Hymenoptera	Chalcidoidea	Pteromalidae	Pteromalidae	sp.	KM560327.1
Hymenoptera	Chalcidoidea	Pteromalidae	Pteromalidae	sp.	KM560355.1
Hymenoptera	Chalcidoidea	Pteromalidae	Pteromalidae	sp.	KM560420.1
Hymenoptera	Chalcidoidea	Pteromalidae	Pteromalidae	sp.	KM560466.1
Hymenoptera	Chalcidoidea	Pteromalidae	Pteromalidae	sp.	KM560610.1
Hymenoptera	Chalcidoidea	Pteromalidae	Pteromalidae	sp.	KM560619.1
Hymenoptera	Chalcidoidea	Pteromalidae	Pteromalidae	sp.	KM560656.1
Hymenoptera	Chalcidoidea	Pteromalidae	Pteromalidae	sp.	KM560701.1
Hymenoptera	Chalcidoidea	Pteromalidae	Pteromalidae	sp.	KM560716.1
Hymenoptera	Chalcidoidea	Pteromalidae	Pteromalidae	sp.	KM560804.1
Hymenoptera	Chalcidoidea	Pteromalidae	Pteromalidae	sp.	KM560823.1
Hymenoptera	Chalcidoidea	Pteromalidae	Pteromalidae	sp.	KM561016.1
Hymenoptera	Chalcidoidea	Pteromalidae	Pteromalidae	sp.	KM561090.1
Hymenoptera	Chalcidoidea	Pteromalidae	Pteromalidae	sp.	KM561106.1
Hymenoptera	Chalcidoidea	Pteromalidae	Pteromalidae	sp.	KM561170.1

Hymenoptera	Chalcidoidea	Pteromalidae	Pteromalidae	sp.	KM561185.1
Hymenoptera	Chalcidoidea	Pteromalidae	Pteromalidae	sp.	KM561264.1
Hymenoptera	Chalcidoidea	Pteromalidae	Pteromalidae	sp.	KM561270.1
Hymenoptera	Chalcidoidea	Pteromalidae	Pteromalidae	sp.	KM561389.1
Hymenoptera	Chalcidoidea	Pteromalidae	Pteromalidae	sp.	KM561393.1
Hymenoptera	Chalcidoidea	Pteromalidae	Pteromalidae	sp.	KM561434.1
Hymenoptera	Chalcidoidea	Pteromalidae	Pteromalidae	sp.	KM561580.1
Hymenoptera	Chalcidoidea	Pteromalidae	Pteromalidae	sp.	KM561599.1
Hymenoptera	Chalcidoidea	Pteromalidae	Pteromalidae	sp.	KM561655.1
Hymenoptera	Chalcidoidea	Pteromalidae	Pteromalidae	sp.	KM561743.1
Hymenoptera	Chalcidoidea	Pteromalidae	Pteromalidae	sp.	KM561797.1
Hymenoptera	Chalcidoidea	Pteromalidae	Pteromalidae	sp.	KM562201.1
Hymenoptera	Chalcidoidea	Pteromalidae	Pteromalidae	sp.	KM562350.1
Hymenoptera	Chalcidoidea	Pteromalidae	Pteromalidae	sp.	KM562391.1
Hymenoptera	Chalcidoidea	Pteromalidae	Pteromalidae	sp.	KM562392.1
Hymenoptera	Chalcidoidea	Pteromalidae	Pteromalidae	sp.	KM562397.1
Hymenoptera	Chalcidoidea	Pteromalidae	Pteromalidae	sp.	KM562513.1
Hymenoptera	Chalcidoidea	Pteromalidae	Pteromalidae	sp.	KM562772.1
Hymenoptera	Chalcidoidea	Pteromalidae	Pteromalidae	sp.	KM562812.1
Hymenoptera	Chalcidoidea	Pteromalidae	Pteromalidae	sp.	KM562863.1
Hymenoptera	Chalcidoidea	Pteromalidae	Pteromalidae	sp.	KM562987.1
Hymenoptera	Chalcidoidea	Pteromalidae	Pteromalidae	sp.	KM563032.1
Hymenoptera	Chalcidoidea	Pteromalidae	Pteromalidae	sp.	KM563206.1
Hymenoptera	Chalcidoidea	Pteromalidae	Pteromalidae	sp.	KM563295.1
Hymenoptera	Chalcidoidea	Pteromalidae	Pteromalidae	sp.	KM563533.1
Hymenoptera	Chalcidoidea	Pteromalidae	Pteromalidae	sp.	KM563594.1
Hymenoptera	Chalcidoidea	Pteromalidae	Pteromalidae	sp.	KM563646.1
Hymenoptera	Chalcidoidea	Pteromalidae	Pteromalidae	sp.	KM563685.1
Hymenoptera	Chalcidoidea	Pteromalidae	Pteromalidae	sp.	KM563802.1
Hymenoptera	Chalcidoidea	Pteromalidae	Pteromalidae	sp.	KM563898.1
Hymenoptera	Chalcidoidea	Pteromalidae	Pteromalidae	sp.	KM563970.1
Hymenoptera	Chalcidoidea	Pteromalidae	Pteromalidae	sp.	KM564162.1
Hymenoptera	Chalcidoidea	Pteromalidae	Pteromalidae	sp.	KM564179.1
Hymenoptera	Chalcidoidea	Pteromalidae	Pteromalidae	sp.	KM564209.1
Hymenoptera	Chalcidoidea	Pteromalidae	Pteromalidae	sp.	KM564211.1
Hymenoptera	Chalcidoidea	Pteromalidae	Pteromalidae	sp.	KM564275.1
Hymenoptera	Chalcidoidea	Pteromalidae	Pteromalidae	sp.	KM564329.1
Hymenoptera	Chalcidoidea	Pteromalidae	Pteromalidae	sp.	KM564395.1
Hymenoptera	Chalcidoidea	Pteromalidae	Pteromalidae	sp.	KM564558.1
Hymenoptera	Chalcidoidea	Pteromalidae	Pteromalidae	sp.	KM564567.1
Hymenoptera	Chalcidoidea	Pteromalidae	Pteromalidae	sp.	KM564617.1
Hymenoptera	Chalcidoidea	Pteromalidae	Pteromalidae	sp.	KM564684.1
Hymenoptera	Chalcidoidea	Pteromalidae	Pteromalidae	sp.	KM564704.1
Hymenoptera	Chalcidoidea	Pteromalidae	Pteromalidae	sp.	KM564774.1
Hymenoptera	Chalcidoidea	Pteromalidae	Pteromalidae	sp.	KM564894.1
Hymenoptera	Chalcidoidea	Pteromalidae	Pteromalidae	sp.	KM564952.1
Hymenoptera	Chalcidoidea	Pteromalidae	Pteromalidae	sp.	KM565061.1
Hymenoptera	Chalcidoidea	Pteromalidae	Pteromalidae	sp.	KM565106.1
Hymenoptera	Chalcidoidea	Pteromalidae	Pteromalidae	sp.	KM565203.1
Hymenoptera	Chalcidoidea	Pteromalidae	Pteromalidae	sp.	KM565411.1
Hymenoptera	Chalcidoidea	Pteromalidae	Pteromalidae	sp.	KM565427.1
Hymenoptera	Chalcidoidea	Pteromalidae	Pteromalidae	sp.	KM565500.1
Hymenoptera	Chalcidoidea	Pteromalidae	Pteromalidae	sp.	KM565599.1
Hymenoptera	Chalcidoidea	Pteromalidae	Pteromalidae	sp.	KM565620.1

Hymenoptera	Chalcidoidea	Pteromalidae	Pteromalidae	sp.	KM565675.1
Hymenoptera	Chalcidoidea	Pteromalidae	Pteromalidae	sp.	KM565676.1
Hymenoptera	Chalcidoidea	Pteromalidae	Pteromalidae	sp.	KM565767.1
Hymenoptera	Chalcidoidea	Pteromalidae	Pteromalidae	sp.	KM565968.1
Hymenoptera	Chalcidoidea	Pteromalidae	Pteromalidae	sp.	KM565990.1
Hymenoptera	Chalcidoidea	Pteromalidae	Pteromalidae	sp.	KM566000.1
Hymenoptera	Chalcidoidea	Pteromalidae	Pteromalidae	sp.	KM566009.1
Hymenoptera	Chalcidoidea	Pteromalidae	Pteromalidae	sp.	KM566015.1
Hymenoptera	Chalcidoidea	Pteromalidae	Pteromalidae	sp.	KM566057.1
Hymenoptera	Chalcidoidea	Pteromalidae	Pteromalidae	sp.	KM566082.1
Hymenoptera	Chalcidoidea	Pteromalidae	Pteromalidae	sp.	KM566294.1
Hymenoptera	Chalcidoidea	Pteromalidae	Pteromalidae	sp.	KM566305.1
Hymenoptera	Chalcidoidea	Pteromalidae	Pteromalidae	sp.	KM566407.1
Hymenoptera	Chalcidoidea	Pteromalidae	Pteromalidae	sp.	KM566551.1
Hymenoptera	Chalcidoidea	Pteromalidae	Pteromalidae	sp.	KM566635.1
Hymenoptera	Chalcidoidea	Pteromalidae	Pteromalidae	sp.	KM566780.1
Hymenoptera	Chalcidoidea	Pteromalidae	Pteromalidae	sp.	KM566982.1
Hymenoptera	Chalcidoidea	Pteromalidae	Pteromalidae	sp.	KM567051.1
Hymenoptera	Chalcidoidea	Pteromalidae	Pteromalidae	sp.	KM567256.1
Hymenoptera	Chalcidoidea	Pteromalidae	Pteromalidae	sp.	KM567362.1
Hymenoptera	Chalcidoidea	Pteromalidae	Pteromalidae	sp.	KM567438.1
Hymenoptera	Chalcidoidea	Pteromalidae	Pteromalidae	sp.	KM567536.1
Hymenoptera	Chalcidoidea	Pteromalidae	Pteromalidae	sp.	KM567893.1
Hymenoptera	Chalcidoidea	Pteromalidae	Pteromalidae	sp.	KM567972.1
Hymenoptera	Chalcidoidea	Pteromalidae	Pteromalidae	sp.	KM567998.1
Hymenoptera	Chalcidoidea	Pteromalidae	Pteromalidae	sp.	KM568035.1
Hymenoptera	Chalcidoidea	Pteromalidae	Pteromalidae	sp.	KM568070.1
Hymenoptera	Chalcidoidea	Pteromalidae	Pteromalidae	sp.	KM568106.1
Hymenoptera	Chalcidoidea	Pteromalidae	Pteromalidae	sp.	KM568122.1
Hymenoptera	Chalcidoidea	Pteromalidae	Pteromalidae	sp.	KM568210.1
Hymenoptera	Chalcidoidea	Pteromalidae	Pteromalidae	sp.	KM568288.1
Hymenoptera	Chalcidoidea	Pteromalidae	Pteromalidae	sp.	KM568315.1
Hymenoptera	Chalcidoidea	Pteromalidae	Pteromalidae	sp.	KM568712.1
Hymenoptera	Chalcidoidea	Pteromalidae	Pteromalidae	sp.	KM568730.1
Hymenoptera	Chalcidoidea	Pteromalidae	Pteromalidae	sp.	KM568969.1
Hymenoptera	Chalcidoidea	Pteromalidae	Pteromalidae	sp.	KM568978.1
Hymenoptera	Chalcidoidea	Pteromalidae	Pteromalidae	sp.	KM569076.1
Hymenoptera	Chalcidoidea	Pteromalidae	Pteromalidae	sp.	KM569154.1
Hymenoptera	Chalcidoidea	Pteromalidae	Pteromalidae	sp.	KM569280.1
Hymenoptera	Chalcidoidea	Pteromalidae	Pteromalidae	sp.	KM569306.1
Hymenoptera	Chalcidoidea	Pteromalidae	Pteromalidae	sp.	KM569603.1
Hymenoptera	Chalcidoidea	Pteromalidae	Pteromalidae	sp.	KM569607.1
Hymenoptera	Chalcidoidea	Pteromalidae	Pteromalidae	sp.	KR364978.1
Hymenoptera	Chalcidoidea	Pteromalidae	Pteromalidae	sp.	KR365251.1
Hymenoptera	Chalcidoidea	Pteromalidae	Pteromalidae	sp.	KR365438.1
Hymenoptera	Chalcidoidea	Pteromalidae	Pteromalidae	sp.	KR365647.1
Hymenoptera	Chalcidoidea	Pteromalidae	Pteromalidae	sp.	KR365678.1
Hymenoptera	Chalcidoidea	Pteromalidae	Pteromalidae	sp.	KR366298.1
Hymenoptera	Chalcidoidea	Pteromalidae	Pteromalidae	sp.	KR366360.1
Hymenoptera	Chalcidoidea	Pteromalidae	Pteromalidae	sp.	KR366501.1
Hymenoptera	Chalcidoidea	Pteromalidae	Pteromalidae	sp.	KR366651.1
Hymenoptera	Chalcidoidea	Pteromalidae	Pteromalidae	sp.	KR366729.1
Hymenoptera	Chalcidoidea	Pteromalidae	Pteromalidae	sp.	KR366732.1
Hymenoptera	Chalcidoidea	Pteromalidae	Pteromalidae	sp.	KR366899.1

Hymenoptera	Chalcidoidea	Pteromalidae	Pteromalidae	sp.	KR366921.1
Hymenoptera	Chalcidoidea	Pteromalidae	Pteromalidae	sp.	KR367710.1
Hymenoptera	Chalcidoidea	Pteromalidae	Pteromalidae	sp.	KR368323.1
Hymenoptera	Chalcidoidea	Pteromalidae	Pteromalidae	sp.	KR368359.1
Hymenoptera	Chalcidoidea	Pteromalidae	Pteromalidae	sp.	KR368671.1
Hymenoptera	Chalcidoidea	Pteromalidae	Pteromalidae	sp.	KR368736.1
Hymenoptera	Chalcidoidea	Pteromalidae	Pteromalidae	sp.	KR368828.1
Hymenoptera	Chalcidoidea	Pteromalidae	Pteromalidae	sp.	KR369044.1
Hymenoptera	Chalcidoidea	Pteromalidae	Pteromalidae	sp.	KR369055.1
Hymenoptera	Chalcidoidea	Pteromalidae	Pteromalidae	sp.	KR369196.1
Hymenoptera	Chalcidoidea	Pteromalidae	Pteromalidae	sp.	KR369383.1
Hymenoptera	Chalcidoidea	Pteromalidae	Pteromalidae	sp.	KR369517.1
Hymenoptera	Chalcidoidea	Pteromalidae	Pteromalidae	sp.	KR369539.1
Hymenoptera	Chalcidoidea	Pteromalidae	Pteromalidae	sp.	KR369731.1
Hymenoptera	Chalcidoidea	Pteromalidae	Pteromalidae	sp.	KR369761.1
Hymenoptera	Chalcidoidea	Pteromalidae	Pteromalidae	sp.	KR370095.1
Hymenoptera	Chalcidoidea	Pteromalidae	Pteromalidae	sp.	KR370101.1
Hymenoptera	Chalcidoidea	Pteromalidae	Pteromalidae	sp.	KR370326.1
Hymenoptera	Chalcidoidea	Pteromalidae	Pteromalidae	sp.	KR370339.1
Hymenoptera	Chalcidoidea	Pteromalidae	Pteromalidae	sp.	KR370624.1
Hymenoptera	Chalcidoidea	Pteromalidae	Pteromalidae	sp.	KR370719.1
Hymenoptera	Chalcidoidea	Pteromalidae	Pteromalidae	sp.	KR370975.1
Hymenoptera	Chalcidoidea	Pteromalidae	Pteromalidae	sp.	KR371121.1
Hymenoptera	Chalcidoidea	Pteromalidae	Pteromalidae	sp.	KR371289.1
Hymenoptera	Chalcidoidea	Pteromalidae	Pteromalidae	sp.	KR371419.1
Hymenoptera	Chalcidoidea	Pteromalidae	Pteromalidae	sp.	KR371940.1
Hymenoptera	Chalcidoidea	Pteromalidae	Pteromalidae	sp.	KR372020.1
Hymenoptera	Chalcidoidea	Pteromalidae	Pteromalidae	sp.	KR372369.1
Hymenoptera	Chalcidoidea	Pteromalidae	Pteromalidae	sp.	KR372432.1
Hymenoptera	Chalcidoidea	Pteromalidae	Pteromalidae	sp.	KR372485.1
Hymenoptera	Chalcidoidea	Pteromalidae	Pteromalidae	sp.	KR373101.1
Hymenoptera	Chalcidoidea	Pteromalidae	Pteromalidae	sp.	KR373437.1
Hymenoptera	Chalcidoidea	Pteromalidae	Pteromalidae	sp.	KR374128.1
Hymenoptera	Chalcidoidea	Pteromalidae	Pteromalidae	sp.	KR375343.1
Hymenoptera	Chalcidoidea	Pteromalidae	Pteromalidae	sp.	KR375473.1
Hymenoptera	Chalcidoidea	Pteromalidae	Pteromalidae	sp.	KR782833.1
Hymenoptera	Chalcidoidea	Pteromalidae	Pteromalidae	sp.	KR783820.1
Hymenoptera	Chalcidoidea	Pteromalidae	Pteromalidae	sp.	KR783913.1
Hymenoptera	Chalcidoidea	Pteromalidae	Pteromalidae	sp.	KR784729.1
Hymenoptera	Chalcidoidea	Pteromalidae	Pteromalidae	sp.	KR785201.1
Hymenoptera	Chalcidoidea	Pteromalidae	Pteromalidae	sp.	KR785470.1
Hymenoptera	Chalcidoidea	Pteromalidae	Pteromalidae	sp.	KR785615.1
Hymenoptera	Chalcidoidea	Pteromalidae	Pteromalidae	sp.	KR785763.1
Hymenoptera	Chalcidoidea	Pteromalidae	Pteromalidae	sp.	KR786079.1
Hymenoptera	Chalcidoidea	Pteromalidae	Pteromalidae	sp.	KR786308.1
Hymenoptera	Chalcidoidea	Pteromalidae	Pteromalidae	sp.	KR786462.1
Hymenoptera	Chalcidoidea	Pteromalidae	Pteromalidae	sp.	KR786723.1
Hymenoptera	Chalcidoidea	Pteromalidae	Pteromalidae	sp.	KR786867.1
Hymenoptera	Chalcidoidea	Pteromalidae	Pteromalidae	sp.	KR787872.1
Hymenoptera	Chalcidoidea	Pteromalidae	Pteromalidae	sp.	KR788284.1
Hymenoptera	Chalcidoidea	Pteromalidae	Pteromalidae	sp.	KR788314.1
Hymenoptera	Chalcidoidea	Pteromalidae	Pteromalidae	sp.	KR788665.1
Hymenoptera	Chalcidoidea	Pteromalidae	Pteromalidae	sp.	KR789225.1
Hymenoptera	Chalcidoidea	Pteromalidae	Pteromalidae	sp.	KR790494.1

Hymenoptera	Chalcidoidea	Pteromalidae	Pteromalidae	sp.	KR790744.1
Hymenoptera	Chalcidoidea	Pteromalidae	Pteromalidae	sp.	KR791333.1
Hymenoptera	Chalcidoidea	Pteromalidae	Pteromalidae	sp.	KR791753.1
Hymenoptera	Chalcidoidea	Pteromalidae	Pteromalidae	sp.	KR792286.1
Hymenoptera	Chalcidoidea	Pteromalidae	Pteromalidae	sp.	KR792866.1
Hymenoptera	Chalcidoidea	Pteromalidae	Pteromalidae	sp.	KR793339.1
Hymenoptera	Chalcidoidea	Pteromalidae	Pteromalidae	sp.	KR793778.1
Hymenoptera	Chalcidoidea	Pteromalidae	Pteromalidae	sp.	KR793913.1
Hymenoptera	Chalcidoidea	Pteromalidae	Pteromalidae	sp.	KR793939.1
Hymenoptera	Chalcidoidea	Pteromalidae	Pteromalidae	sp.	KR793988.1
Hymenoptera	Chalcidoidea	Pteromalidae	Pteromalidae	sp.	KR794031.1
Hymenoptera	Chalcidoidea	Pteromalidae	Pteromalidae	sp.	KR794062.1
Hymenoptera	Chalcidoidea	Pteromalidae	Pteromalidae	sp.	KR795047.1
Hymenoptera	Chalcidoidea	Pteromalidae	Pteromalidae	sp.	KR795173.1
Hymenoptera	Chalcidoidea	Pteromalidae	Pteromalidae	sp.	KR795322.1
Hymenoptera	Chalcidoidea	Pteromalidae	Pteromalidae	sp.	KR795693.1
Hymenoptera	Chalcidoidea	Pteromalidae	Pteromalidae	sp.	KR795921.1
Hymenoptera	Chalcidoidea	Pteromalidae	Pteromalidae	sp.	KR796244.1
Hymenoptera	Chalcidoidea	Pteromalidae	Pteromalidae	sp.	KR796621.1
Hymenoptera	Chalcidoidea	Pteromalidae	Pteromalidae	sp.	KR796793.1
Hymenoptera	Chalcidoidea	Pteromalidae	Pteromalidae	sp.	KR797724.1
Hymenoptera	Chalcidoidea	Pteromalidae	Pteromalidae	sp.	KR798357.1
Hymenoptera	Chalcidoidea	Pteromalidae	Pteromalidae	sp.	KR798384.1
Hymenoptera	Chalcidoidea	Pteromalidae	Pteromalidae	sp.	KR798905.1
Hymenoptera	Chalcidoidea	Pteromalidae	Pteromalidae	sp.	KR799122.1
Hymenoptera	Chalcidoidea	Pteromalidae	Pteromalidae	sp.	KR799435.1
Hymenoptera	Chalcidoidea	Pteromalidae	Pteromalidae	sp.	KR799450.1
Hymenoptera	Chalcidoidea	Pteromalidae	Pteromalidae	sp.	KR800054.1
Hymenoptera	Chalcidoidea	Pteromalidae	Pteromalidae	sp.	KR800484.1
Hymenoptera	Chalcidoidea	Pteromalidae	Pteromalidae	sp.	KR801232.1
Hymenoptera	Chalcidoidea	Pteromalidae	Pteromalidae	sp.	KR801896.1
Hymenoptera	Chalcidoidea	Pteromalidae	Pteromalidae	sp.	KR801934.1
Hymenoptera	Chalcidoidea	Pteromalidae	Pteromalidae	sp.	KR802350.1
Hymenoptera	Chalcidoidea	Pteromalidae	Pteromalidae	sp.	KR802671.1
Hymenoptera	Chalcidoidea	Pteromalidae	Pteromalidae	sp.	KR802713.1
Hymenoptera	Chalcidoidea	Pteromalidae	Pteromalidae	sp.	KR802964.1
Hymenoptera	Chalcidoidea	Pteromalidae	Pteromalidae	sp.	KR804041.1
Hymenoptera	Chalcidoidea	Pteromalidae	Pteromalidae	sp.	KR804096.1
Hymenoptera	Chalcidoidea	Pteromalidae	Pteromalidae	sp.	KR804624.1
Hymenoptera	Chalcidoidea	Pteromalidae	Pteromalidae	sp.	KR804715.1
Hymenoptera	Chalcidoidea	Pteromalidae	Pteromalidae	sp.	KR804869.1
Hymenoptera	Chalcidoidea	Pteromalidae	Pteromalidae	sp.	KR804969.1
Hymenoptera	Chalcidoidea	Pteromalidae	Pteromalidae	sp.	KR805218.1
Hymenoptera	Chalcidoidea	Pteromalidae	Pteromalidae	sp.	KR805367.1
Hymenoptera	Chalcidoidea	Pteromalidae	Pteromalidae	sp.	KR805519.1
Hymenoptera	Chalcidoidea	Pteromalidae	Pteromalidae	sp.	KR806238.1
Hymenoptera	Chalcidoidea	Pteromalidae	Pteromalidae	sp.	KR807343.1
Hymenoptera	Chalcidoidea	Pteromalidae	Pteromalidae	sp.	KR807374.1
Hymenoptera	Chalcidoidea	Pteromalidae	Pteromalidae	sp.	KR808128.1
Hymenoptera	Chalcidoidea	Pteromalidae	Pteromalidae	sp.	KR808560.1
Hymenoptera	Chalcidoidea	Pteromalidae	Pteromalidae	sp.	KR808637.1
Hymenoptera	Chalcidoidea	Pteromalidae	Pteromalidae	sp.	KR875388.1
Hymenoptera	Chalcidoidea	Pteromalidae	Pteromalidae	sp.	KR875493.1
Hymenoptera	Chalcidoidea	Pteromalidae	Pteromalidae	sp.	KR876577.1

Hymenoptera	Chalcidoidea	Pteromalidae	Pteromalidae	sp.	KR877239.1
Hymenoptera	Chalcidoidea	Pteromalidae	Pteromalidae	sp.	KR877372.1
Hymenoptera	Chalcidoidea	Pteromalidae	Pteromalidae	sp.	KR877502.1
Hymenoptera	Chalcidoidea	Pteromalidae	Pteromalidae	sp.	KR877657.1
Hymenoptera	Chalcidoidea	Pteromalidae	Pteromalidae	sp.	KR878665.1
Hymenoptera	Chalcidoidea	Pteromalidae	Pteromalidae	sp.	KR879218.1
Hymenoptera	Chalcidoidea	Pteromalidae	Pteromalidae	sp.	KR879507.1
Hymenoptera	Chalcidoidea	Pteromalidae	Pteromalidae	sp.	KR879662.1
Hymenoptera	Chalcidoidea	Pteromalidae	Pteromalidae	sp.	KR879822.1
Hymenoptera	Chalcidoidea	Pteromalidae	Pteromalidae	sp.	KR880130.1
Hymenoptera	Chalcidoidea	Pteromalidae	Pteromalidae	sp.	KR881810.1
Hymenoptera	Chalcidoidea	Pteromalidae	Pteromalidae	sp.	KR883101.1
Hymenoptera	Chalcidoidea	Pteromalidae	Pteromalidae	sp.	KR883264.1
Hymenoptera	Chalcidoidea	Pteromalidae	Pteromalidae	sp.	KR883850.1
Hymenoptera	Chalcidoidea	Pteromalidae	Pteromalidae	sp.	KR883929.1
Hymenoptera	Chalcidoidea	Pteromalidae	Pteromalidae	sp.	KR883982.1
Hymenoptera	Chalcidoidea	Pteromalidae	Pteromalidae	sp.	KR884939.1
Hymenoptera	Chalcidoidea	Pteromalidae	Pteromalidae	sp.	KR885251.1
Hymenoptera	Chalcidoidea	Pteromalidae	Pteromalidae	sp.	KR885814.1
Hymenoptera	Chalcidoidea	Pteromalidae	Pteromalidae	sp.	KR886275.1
Hymenoptera	Chalcidoidea	Pteromalidae	Pteromalidae	sp.	KR888305.1
Hymenoptera	Chalcidoidea	Pteromalidae	Pteromalidae	sp.	KR888824.1
Hymenoptera	Chalcidoidea	Pteromalidae	Pteromalidae	sp.	KR889438.1
Hymenoptera	Chalcidoidea	Pteromalidae	Pteromalidae	sp.	KR889463.1
Hymenoptera	Chalcidoidea	Pteromalidae	Pteromalidae	sp.	KR889499.1
Hymenoptera	Chalcidoidea	Pteromalidae	Pteromalidae	sp.	KR891350.1
Hymenoptera	Chalcidoidea	Pteromalidae	Pteromalidae	sp.	KR892105.1
Hymenoptera	Chalcidoidea	Pteromalidae	Pteromalidae	sp.	KR892893.1
Hymenoptera	Chalcidoidea	Pteromalidae	Pteromalidae	sp.	KR893082.1
Hymenoptera	Chalcidoidea	Pteromalidae	Pteromalidae	sp.	KR895583.1
Hymenoptera	Chalcidoidea	Pteromalidae	Pteromalidae	sp.	KR895718.1
Hymenoptera	Chalcidoidea	Pteromalidae	Pteromalidae	sp.	KR895991.1
Hymenoptera	Chalcidoidea	Pteromalidae	Pteromalidae	sp.	KR897069.1
Hymenoptera	Chalcidoidea	Pteromalidae	Pteromalidae	sp.	KR898415.1
Hymenoptera	Chalcidoidea	Pteromalidae	Pteromalidae	sp.	KR899319.1
Hymenoptera	Chalcidoidea	Pteromalidae	Pteromalidae	sp.	KR899413.1
Hymenoptera	Chalcidoidea	Pteromalidae	Pteromalidae	sp.	KR899607.1
Hymenoptera	Chalcidoidea	Pteromalidae	Pteromalidae	sp.	KR900146.1
Hymenoptera	Chalcidoidea	Pteromalidae	Pteromalidae	sp.	KR901071.1
Hymenoptera	Chalcidoidea	Pteromalidae	Pteromalidae	sp.	KR924533.1
Hymenoptera	Chalcidoidea	Pteromalidae	Pteromalidae	sp.	KR926317.1
Hymenoptera	Chalcidoidea	Pteromalidae	Pteromalidae	sp.	KR927766.1
Hymenoptera	Chalcidoidea	Pteromalidae	Pteromalidae	sp.	KR930348.1
Hymenoptera	Chalcidoidea	Pteromalidae	Pteromalidae	sp.	KR932539.1
Hymenoptera	Chalcidoidea	Pteromalidae	Pteromalidae	sp.	KR933666.1
Hymenoptera	Chalcidoidea	Pteromalidae	Pteromalidae	sp.	KT706154.1
Hymenoptera	Chalcidoidea	Pteromalidae	Pteromalidae	sp.	KU567201.1
Hymenoptera	Chalcidoidea	Pteromalidae	Pteromalidae	sp.	KX535120.1
Hymenoptera	Chalcidoidea	Pteromalidae	Pteromalidae	sp.	KX535173.1
Hymenoptera	Chalcidoidea	Pteromalidae	Pteromalidae	sp.	KY831116.1
Hymenoptera	Chalcidoidea	Pteromalidae	Pteromalidae	sp.	KY831850.1
Hymenoptera	Chalcidoidea	Pteromalidae	Pteromalidae	sp.	KY834170.1
Hymenoptera	Chalcidoidea	Pteromalidae	Pteromalidae	sp.	KY834465.1
Hymenoptera	Chalcidoidea	Pteromalidae	Pteromalidae	sp.	KY835062.1

Hymenoptera	Chalcidoidea	Pteromalidae	Pteromalidae	sp.	KY836675.1
Hymenoptera	Chalcidoidea	Pteromalidae	Pteromalidae	sp.	KY837858.1
Hymenoptera	Chalcidoidea	Pteromalidae	Pteromalidae	sp.	KY838040.1
Hymenoptera	Chalcidoidea	Pteromalidae	Pteromalidae	sp.	KY838526.1
Hymenoptera	Chalcidoidea	Pteromalidae	Pteromalidae	sp.	KY838796.1
Hymenoptera	Chalcidoidea	Pteromalidae	Pteromalidae	sp.	KY840963.1
Hymenoptera	Chalcidoidea	Pteromalidae	Pteromalidae	sp.	KY841388.1
Hymenoptera	Chalcidoidea	Pteromalidae	Pteromalidae	sp.	KY841450.1
Hymenoptera	Chalcidoidea	Pteromalidae	Pteromalidae	sp.	KY842565.1
Hymenoptera	Chalcidoidea	Pteromalidae	Pteromalidae	sp.	KY842624.1
Hymenoptera	Chalcidoidea	Pteromalidae	Pteromalidae	sp.	KY843284.1
Hymenoptera	Chalcidoidea	Pteromalidae	Pteromalidae	sp.	KY844112.1
Hymenoptera	Chalcidoidea	Pteromalidae	Pteromalidae	sp.	KY844182.1
Hymenoptera	Chalcidoidea	Pteromalidae	Pteromalidae	sp.	KY844415.1
Hymenoptera	Chalcidoidea	Pteromalidae	Pteromalidae	sp.	KY845874.1
Hymenoptera	Chalcidoidea	Pteromalidae	Pteromalidae	sp.	KY847256.1
Hymenoptera	Chalcidoidea	Pteromalidae	Pteromalidae	sp.	LC604139.1
Hymenoptera	Chalcidoidea	Pteromalidae	Pteromalidae	sp.	MG373883.1
Hymenoptera	Chalcidoidea	Pteromalidae	Pteromalidae	sp.	MG373897.1
Hymenoptera	Chalcidoidea	Pteromalidae	Pteromalidae	sp.	MG373928.1
Hymenoptera	Chalcidoidea	Pteromalidae	Pteromalidae	sp.	MG373959.1
Hymenoptera	Chalcidoidea	Pteromalidae	Pteromalidae	sp.	MG374040.1
Hymenoptera	Chalcidoidea	Pteromalidae	Pteromalidae	sp.	MG374096.1
Hymenoptera	Chalcidoidea	Pteromalidae	Pteromalidae	sp.	MG374224.1
Hymenoptera	Chalcidoidea	Pteromalidae	Pteromalidae	sp.	MG374232.1
Hymenoptera	Chalcidoidea	Pteromalidae	Pteromalidae	sp.	MG374280.1
Hymenoptera	Chalcidoidea	Pteromalidae	Pteromalidae	sp.	MG374338.1
Hymenoptera	Chalcidoidea	Pteromalidae	Pteromalidae	sp.	MG374344.1
Hymenoptera	Chalcidoidea	Pteromalidae	Pteromalidae	sp.	MG374451.1
Hymenoptera	Chalcidoidea	Pteromalidae	Pteromalidae	sp.	MG374461.1
Hymenoptera	Chalcidoidea	Pteromalidae	Pteromalidae	sp.	MG374644.1
Hymenoptera	Chalcidoidea	Pteromalidae	Pteromalidae	sp.	MG374666.1
Hymenoptera	Chalcidoidea	Pteromalidae	Pteromalidae	sp.	MG374754.1
Hymenoptera	Chalcidoidea	Pteromalidae	Pteromalidae	sp.	MG374766.1
Hymenoptera	Chalcidoidea	Pteromalidae	Pteromalidae	sp.	MG374787.1
Hymenoptera	Chalcidoidea	Pteromalidae	Pteromalidae	sp.	MG374840.1
Hymenoptera	Chalcidoidea	Pteromalidae	Pteromalidae	sp.	MG374874.1
Hymenoptera	Chalcidoidea	Pteromalidae	Pteromalidae	sp.	MG374894.1
Hymenoptera	Chalcidoidea	Pteromalidae	Pteromalidae	sp.	MG374904.1
Hymenoptera	Chalcidoidea	Pteromalidae	Pteromalidae	sp.	MG374925.1
Hymenoptera	Chalcidoidea	Pteromalidae	Pteromalidae	sp.	MG374962.1
Hymenoptera	Chalcidoidea	Pteromalidae	Pteromalidae	sp.	MG375019.1
Hymenoptera	Chalcidoidea	Pteromalidae	Pteromalidae	sp.	MG375030.1
Hymenoptera	Chalcidoidea	Pteromalidae	Pteromalidae	sp.	MG375105.1
Hymenoptera	Chalcidoidea	Pteromalidae	Pteromalidae	sp.	MG375122.1
Hymenoptera	Chalcidoidea	Pteromalidae	Pteromalidae	sp.	MG375219.1
Hymenoptera	Chalcidoidea	Pteromalidae	Pteromalidae	sp.	MG375284.1
Hymenoptera	Chalcidoidea	Pteromalidae	Pteromalidae	sp.	MG375285.1
Hymenoptera	Chalcidoidea	Pteromalidae	Pteromalidae	sp.	MG375308.1
Hymenoptera	Chalcidoidea	Pteromalidae	Pteromalidae	sp.	MG375408.1
Hymenoptera	Chalcidoidea	Pteromalidae	Pteromalidae	sp.	MG375552.1
Hymenoptera	Chalcidoidea	Pteromalidae	Pteromalidae	sp.	MG375766.1
Hymenoptera	Chalcidoidea	Pteromalidae	Pteromalidae	sp.	MG375865.1
Hymenoptera	Chalcidoidea	Pteromalidae	Pteromalidae	sp.	MG375919.1

Hymenoptera	Chalcidoidea	Pteromalidae	Pteromalidae	sp.	MG376005.1
Hymenoptera	Chalcidoidea	Pteromalidae	Pteromalidae	sp.	MG376068.1
Hymenoptera	Chalcidoidea	Pteromalidae	Pteromalidae	sp.	MG376167.1
Hymenoptera	Chalcidoidea	Pteromalidae	Pteromalidae	sp.	MG376195.1
Hymenoptera	Chalcidoidea	Pteromalidae	Pteromalidae	sp.	MG376201.1
Hymenoptera	Chalcidoidea	Pteromalidae	Pteromalidae	sp.	MG376209.1
Hymenoptera	Chalcidoidea	Pteromalidae	Pteromalidae	sp.	MG376221.1
Hymenoptera	Chalcidoidea	Pteromalidae	Pteromalidae	sp.	MG376290.1
Hymenoptera	Chalcidoidea	Pteromalidae	Pteromalidae	sp.	MG376356.1
Hymenoptera	Chalcidoidea	Pteromalidae	Pteromalidae	sp.	MG376485.1
Hymenoptera	Chalcidoidea	Pteromalidae	Pteromalidae	sp.	MG376534.1
Hymenoptera	Chalcidoidea	Pteromalidae	Pteromalidae	sp.	MG376573.1
Hymenoptera	Chalcidoidea	Pteromalidae	Pteromalidae	sp.	MG376740.1
Hymenoptera	Chalcidoidea	Pteromalidae	Pteromalidae	sp.	MG376770.1
Hymenoptera	Chalcidoidea	Pteromalidae	Pteromalidae	sp.	MG376826.1
Hymenoptera	Chalcidoidea	Pteromalidae	Pteromalidae	sp.	MG376851.1
Hymenoptera	Chalcidoidea	Pteromalidae	Pteromalidae	sp.	MG376883.1
Hymenoptera	Chalcidoidea	Pteromalidae	Pteromalidae	sp.	MG376895.1
Hymenoptera	Chalcidoidea	Pteromalidae	Pteromalidae	sp.	MG376968.1
Hymenoptera	Chalcidoidea	Pteromalidae	Pteromalidae	sp.	MG377130.1
Hymenoptera	Chalcidoidea	Pteromalidae	Pteromalidae	sp.	MG377301.1
Hymenoptera	Chalcidoidea	Pteromalidae	Pteromalidae	sp.	MG377470.1
Hymenoptera	Chalcidoidea	Pteromalidae	Pteromalidae	sp.	MG377876.1
Hymenoptera	Chalcidoidea	Pteromalidae	Pteromalidae	sp.	MG377939.1
Hymenoptera	Chalcidoidea	Pteromalidae	Pteromalidae	sp.	MG378001.1
Hymenoptera	Chalcidoidea	Pteromalidae	Pteromalidae	sp.	MG378022.1
Hymenoptera	Chalcidoidea	Pteromalidae	Pteromalidae	sp.	MG378059.1
Hymenoptera	Chalcidoidea	Pteromalidae	Pteromalidae	sp.	MG378151.1
Hymenoptera	Chalcidoidea	Pteromalidae	Pteromalidae	sp.	MG378215.1
Hymenoptera	Chalcidoidea	Pteromalidae	Pteromalidae	sp.	MG378225.1
Hymenoptera	Chalcidoidea	Pteromalidae	Pteromalidae	sp.	MG378238.1
Hymenoptera	Chalcidoidea	Pteromalidae	Pteromalidae	sp.	MG378322.1
Hymenoptera	Chalcidoidea	Pteromalidae	Pteromalidae	sp.	MG378336.1
Hymenoptera	Chalcidoidea	Pteromalidae	Pteromalidae	sp.	MG378389.1
Hymenoptera	Chalcidoidea	Pteromalidae	Pteromalidae	sp.	MG378409.1
Hymenoptera	Chalcidoidea	Pteromalidae	Pteromalidae	sp.	MG378412.1
Hymenoptera	Chalcidoidea	Pteromalidae	Pteromalidae	sp.	MG378707.1
Hymenoptera	Chalcidoidea	Pteromalidae	Pteromalidae	sp.	MG378861.1
Hymenoptera	Chalcidoidea	Pteromalidae	Pteromalidae	sp.	MG378976.1
Hymenoptera	Chalcidoidea	Pteromalidae	Pteromalidae	sp.	MG378985.1
Hymenoptera	Chalcidoidea	Pteromalidae	Pteromalidae	sp.	MG379119.1
Hymenoptera	Chalcidoidea	Pteromalidae	Pteromalidae	sp.	MG379190.1
Hymenoptera	Chalcidoidea	Pteromalidae	Pteromalidae	sp.	MG379202.1
Hymenoptera	Chalcidoidea	Pteromalidae	Pteromalidae	sp.	MG379214.1
Hymenoptera	Chalcidoidea	Pteromalidae	Pteromalidae	sp.	MG379376.1
Hymenoptera	Chalcidoidea	Pteromalidae	Pteromalidae	sp.	MG379409.1
Hymenoptera	Chalcidoidea	Pteromalidae	Pteromalidae	sp.	MG379443.1
Hymenoptera	Chalcidoidea	Pteromalidae	Pteromalidae	sp.	MG379457.1
Hymenoptera	Chalcidoidea	Pteromalidae	Pteromalidae	sp.	MG379459.1
Hymenoptera	Chalcidoidea	Pteromalidae	Pteromalidae	sp.	MG379522.1
Hymenoptera	Chalcidoidea	Pteromalidae	Pteromalidae	sp.	MG379782.1
Hymenoptera	Chalcidoidea	Pteromalidae	Pteromalidae	sp.	MG379943.1
Hymenoptera	Chalcidoidea	Pteromalidae	Pteromalidae	sp.	MG380134.1
Hymenoptera	Chalcidoidea	Pteromalidae	Pteromalidae	sp.	MG380169.1

Hymenoptera	Chalcidoidea	Pteromalidae	Pteromalidae	sp.	MG380374.1
Hymenoptera	Chalcidoidea	Pteromalidae	Pteromalidae	sp.	MG380431.1
Hymenoptera	Chalcidoidea	Pteromalidae	Pteromalidae	sp.	MG380440.1
Hymenoptera	Chalcidoidea	Pteromalidae	Pteromalidae	sp.	MG380467.1
Hymenoptera	Chalcidoidea	Pteromalidae	Pteromalidae	sp.	MG380634.1
Hymenoptera	Chalcidoidea	Pteromalidae	Pteromalidae	sp.	MG380662.1
Hymenoptera	Chalcidoidea	Pteromalidae	Pteromalidae	sp.	MG380698.1
Hymenoptera	Chalcidoidea	Pteromalidae	Pteromalidae	sp.	MG380703.1
Hymenoptera	Chalcidoidea	Pteromalidae	Pteromalidae	sp.	MG380810.1
Hymenoptera	Chalcidoidea	Pteromalidae	Pteromalidae	sp.	MG380860.1
Hymenoptera	Chalcidoidea	Pteromalidae	Pteromalidae	sp.	MG380997.1
Hymenoptera	Chalcidoidea	Pteromalidae	Pteromalidae	sp.	MG381288.1
Hymenoptera	Chalcidoidea	Pteromalidae	Pteromalidae	sp.	MG381484.1
Hymenoptera	Chalcidoidea	Pteromalidae	Pteromalidae	sp.	MG381492.1
Hymenoptera	Chalcidoidea	Pteromalidae	Pteromalidae	sp.	MG381504.1
Hymenoptera	Chalcidoidea	Pteromalidae	Pteromalidae	sp.	MG381519.1
Hymenoptera	Chalcidoidea	Pteromalidae	Pteromalidae	sp.	MG381600.1
Hymenoptera	Chalcidoidea	Pteromalidae	Pteromalidae	sp.	MG381677.1
Hymenoptera	Chalcidoidea	Pteromalidae	Pteromalidae	sp.	MG381725.1
Hymenoptera	Chalcidoidea	Pteromalidae	Pteromalidae	sp.	MG381783.1
Hymenoptera	Chalcidoidea	Pteromalidae	Pteromalidae	sp.	MG381921.1
Hymenoptera	Chalcidoidea	Pteromalidae	Pteromalidae	sp.	MG381998.1
Hymenoptera	Chalcidoidea	Pteromalidae	Pteromalidae	sp.	MG382058.1
Hymenoptera	Chalcidoidea	Pteromalidae	Pteromalidae	sp.	MG382120.1
Hymenoptera	Chalcidoidea	Pteromalidae	Pteromalidae	sp.	MG382195.1
Hymenoptera	Chalcidoidea	Pteromalidae	Pteromalidae	sp.	MG382308.1
Hymenoptera	Chalcidoidea	Pteromalidae	Pteromalidae	sp.	MG382336.1
Hymenoptera	Chalcidoidea	Pteromalidae	Pteromalidae	sp.	MG382463.1
Hymenoptera	Chalcidoidea	Pteromalidae	Pteromalidae	sp.	MG382495.1
Hymenoptera	Chalcidoidea	Pteromalidae	Pteromalidae	sp.	MG382884.1
Hymenoptera	Chalcidoidea	Pteromalidae	Pteromalidae	sp.	MG382993.1
Hymenoptera	Chalcidoidea	Pteromalidae	Pteromalidae	sp.	MG383140.1
Hymenoptera	Chalcidoidea	Pteromalidae	Pteromalidae	sp.	MG383151.1
Hymenoptera	Chalcidoidea	Pteromalidae	Pteromalidae	sp.	MG383334.1
Hymenoptera	Chalcidoidea	Pteromalidae	Pteromalidae	sp.	MG497004.1
Hymenoptera	Chalcidoidea	Pteromalidae	Pteromalidae	sp.	MG497146.1
Hymenoptera	Chalcidoidea	Pteromalidae	Pteromalidae	sp.	MG497149.1
Hymenoptera	Chalcidoidea	Pteromalidae	Pteromalidae	sp.	MG497224.1
Hymenoptera	Chalcidoidea	Pteromalidae	Pteromalidae	sp.	MG497346.1
Hymenoptera	Chalcidoidea	Pteromalidae	Pteromalidae	sp.	MG497421.1
Hymenoptera	Chalcidoidea	Pteromalidae	Pteromalidae	sp.	MG497519.1
Hymenoptera	Chalcidoidea	Pteromalidae	Pteromalidae	sp.	MG497540.1
Hymenoptera	Chalcidoidea	Pteromalidae	Pteromalidae	sp.	MG497651.1
Hymenoptera	Chalcidoidea	Pteromalidae	Pteromalidae	sp.	MG497680.1
Hymenoptera	Chalcidoidea	Pteromalidae	Pteromalidae	sp.	MG497732.1
Hymenoptera	Chalcidoidea	Pteromalidae	Pteromalidae	sp.	MG497763.1
Hymenoptera	Chalcidoidea	Pteromalidae	Pteromalidae	sp.	MG497828.1
Hymenoptera	Chalcidoidea	Pteromalidae	Pteromalidae	sp.	MG497961.1
Hymenoptera	Chalcidoidea	Pteromalidae	Pteromalidae	sp.	MG498122.1
Hymenoptera	Chalcidoidea	Pteromalidae	Pteromalidae	sp.	MG498215.1
Hymenoptera	Chalcidoidea	Pteromalidae	Pteromalidae	sp.	MG498235.1
Hymenoptera	Chalcidoidea	Pteromalidae	Pteromalidae	sp.	MG498422.1
Hymenoptera	Chalcidoidea	Pteromalidae	Pteromalidae	sp.	MG498777.1
Hymenoptera	Chalcidoidea	Pteromalidae	Pteromalidae	sp.	MG498814.1

Hymenoptera	Chalcidoidea	Pteromalidae	Pteromalidae	sp.	MG499018.1
Hymenoptera	Chalcidoidea	Pteromalidae	Pteromalidae	sp.	MG499153.1
Hymenoptera	Chalcidoidea	Pteromalidae	Pteromalidae	sp.	MG499165.1
Hymenoptera	Chalcidoidea	Pteromalidae	Pteromalidae	sp.	MG499451.1
Hymenoptera	Chalcidoidea	Pteromalidae	Pteromalidae	sp.	MG499525.1
Hymenoptera	Chalcidoidea	Pteromalidae	Pteromalidae	sp.	MG499819.1
Hymenoptera	Chalcidoidea	Pteromalidae	Pteromalidae	sp.	MG499899.1
Hymenoptera	Chalcidoidea	Pteromalidae	Pteromalidae	sp.	MG499900.1
Hymenoptera	Chalcidoidea	Pteromalidae	Pteromalidae	sp.	MG499909.1
Hymenoptera	Chalcidoidea	Pteromalidae	Pteromalidae	sp.	MG500222.1
Hymenoptera	Chalcidoidea	Pteromalidae	Pteromalidae	sp.	MG500276.1
Hymenoptera	Chalcidoidea	Pteromalidae	Pteromalidae	sp.	MG500337.1
Hymenoptera	Chalcidoidea	Pteromalidae	Pteromalidae	sp.	MG500872.1
Hymenoptera	Chalcidoidea	Pteromalidae	Pteromalidae	sp.	MG501235.1
Hymenoptera	Chalcidoidea	Pteromalidae	Pteromalidae	sp.	MG501278.1
Hymenoptera	Chalcidoidea	Pteromalidae	Pteromalidae	sp.	MG501733.1
Hymenoptera	Chalcidoidea	Pteromalidae	Pteromalidae	sp.	MG501818.1
Hymenoptera	Chalcidoidea	Pteromalidae	Pteromalidae	sp.	MG501893.1
Hymenoptera	Chalcidoidea	Pteromalidae	Pteromalidae	sp.	MG501935.1
Hymenoptera	Chalcidoidea	Pteromalidae	Pteromalidae	sp.	MG502205.1
Hymenoptera	Chalcidoidea	Pteromalidae	Pteromalidae	sp.	MG502475.1
Hymenoptera	Chalcidoidea	Pteromalidae	Pteromalidae	sp.	MG502477.1
Hymenoptera	Chalcidoidea	Pteromalidae	Pteromalidae	sp.	MG502662.1
Hymenoptera	Chalcidoidea	Pteromalidae	Pteromalidae	sp.	MG503027.1
Hymenoptera	Chalcidoidea	Pteromalidae	Pteromalidae	sp.	MG503069.1
Hymenoptera	Chalcidoidea	Pteromalidae	Pteromalidae	sp.	MG503216.1
Hymenoptera	Chalcidoidea	Pteromalidae	Pteromalidae	sp.	MG503271.1
Hymenoptera	Chalcidoidea	Pteromalidae	Pteromalidae	sp.	MG503372.1
Hymenoptera	Chalcidoidea	Pteromalidae	Pteromalidae	sp.	MG503445.1
Hymenoptera	Chalcidoidea	Pteromalidae	Pteromalidae	sp.	MG503617.1
Hymenoptera	Chalcidoidea	Pteromalidae	Pteromalidae	sp.	MG503774.1
Hymenoptera	Chalcidoidea	Pteromalidae	Pteromalidae	sp.	MG503868.1
Hymenoptera	Chalcidoidea	Pteromalidae	Pteromalidae	sp.	MG504018.1
Hymenoptera	Chalcidoidea	Pteromalidae	Pteromalidae	sp.	MG504067.1
Hymenoptera	Chalcidoidea	Pteromalidae	Pteromalidae	sp.	MG504297.1
Hymenoptera	Chalcidoidea	Pteromalidae	Pteromalidae	sp.	MG504317.1
Hymenoptera	Chalcidoidea	Pteromalidae	Pteromalidae	sp.	MG504666.1
Hymenoptera	Chalcidoidea	Pteromalidae	Pteromalidae	sp.	MG504721.1
Hymenoptera	Chalcidoidea	Pteromalidae	Pteromalidae	sp.	MG505039.1
Hymenoptera	Chalcidoidea	Pteromalidae	Pteromalidae	sp.	MG505112.1
Hymenoptera	Chalcidoidea	Pteromalidae	Pteromalidae	sp.	MG505242.1
Hymenoptera	Chalcidoidea	Pteromalidae	Pteromalidae	sp.	MG505271.1
Hymenoptera	Chalcidoidea	Pteromalidae	Pteromalidae	sp.	MG505519.1
Hymenoptera	Chalcidoidea	Pteromalidae	Pteromalidae	sp.	MG505531.1
Hymenoptera	Chalcidoidea	Pteromalidae	Pteromalidae	sp.	MG505680.1
Hymenoptera	Chalcidoidea	Pteromalidae	Pteromalidae	sp.	MG505793.1
Hymenoptera	Chalcidoidea	Pteromalidae	Pteromalidae	sp.	MG505839.1
Hymenoptera	Chalcidoidea	Pteromalidae	Pteromalidae	sp.	MG505937.1
Hymenoptera	Chalcidoidea	Pteromalidae	Pteromalidae	sp.	MG506082.1
Hymenoptera	Chalcidoidea	Pteromalidae	Pteromalidae	sp.	MG506197.1
Hymenoptera	Chalcidoidea	Pteromalidae	Pteromalidae	sp.	MG506253.1
Hymenoptera	Chalcidoidea	Pteromalidae	Pteromalidae	sp.	MG506271.1
Hymenoptera	Chalcidoidea	Pteromalidae	Pteromalidae	sp.	MG506275.1
Hymenoptera	Chalcidoidea	Pteromalidae	Pteromalidae	sp.	MG506412.1

Hymenoptera	Chalcidoidea	Pteromalidae	Pteromalidae	sp.	MG506434.1
Hymenoptera	Chalcidoidea	Pteromalidae	Pteromalidae	sp.	MG506475.1
Hymenoptera	Chalcidoidea	Pteromalidae	Pteromalidae	sp.	MG506836.1
Hymenoptera	Chalcidoidea	Pteromalidae	Pteromalidae	sp.	MG508306.1
Hymenoptera	Chalcidoidea	Pteromalidae	Pteromalidae	sp.	MG509094.1
Hymenoptera	Chalcidoidea	Pteromalidae	Pteromalidae	sp.	MG509606.1
Hymenoptera	Chalcidoidea	Pteromalidae	Pteromalidae	sp.	MG513392.1
Hymenoptera	Chalcidoidea	Pteromalidae	Pteromalidae	sp.	MG513416.1
Hymenoptera	Chalcidoidea	Pteromalidae	Pteromalidae	sp.	MH094913.1
Hymenoptera	Chalcidoidea	Pteromalidae	Pteromalidae	sp.	MH456612.1
Hymenoptera	Chalcidoidea	Pteromalidae	Pteromalidae	sp.	MH926364.1
Hymenoptera	Chalcidoidea	Pteromalidae	Pteromalidae	sp.	MH926699.1
Hymenoptera	Chalcidoidea	Pteromalidae	Pteromalidae	sp.	MH926764.1
Hymenoptera	Chalcidoidea	Pteromalidae	Pteromalidae	sp.	MH926909.1
Hymenoptera	Chalcidoidea	Pteromalidae	Pteromalidae	sp.	MH926982.1
Hymenoptera	Chalcidoidea	Pteromalidae	Pteromalidae	sp.	MH927047.1
Hymenoptera	Chalcidoidea	Pteromalidae	Pteromalidae	sp.	MH927113.1
Hymenoptera	Chalcidoidea	Pteromalidae	Pteromalidae	sp.	MH927452.1
Hymenoptera	Chalcidoidea	Pteromalidae	Pteromalidae	sp.	MH927956.1
Hymenoptera	Chalcidoidea	Pteromalidae	Pteromalidae	sp.	MH927957.1
Hymenoptera	Chalcidoidea	Pteromalidae	Pteromalidae	sp.	MH928496.1
Hymenoptera	Chalcidoidea	Pteromalidae	Pteromalidae	sp.	MH928874.1
Hymenoptera	Chalcidoidea	Pteromalidae	Pteromalidae	sp.	MH929016.1
Hymenoptera	Chalcidoidea	Pteromalidae	Pteromalidae	sp.	MN531302.1
Hymenoptera	Chalcidoidea	Pteromalidae	Pteromalidae	sp.	MN531303.1
Hymenoptera	Chalcidoidea	Pteromalidae	Pteromalidae	sp.	MN665401.1
Hymenoptera	Chalcidoidea	Pteromalidae	Pteromalidae	sp.	MN665933.1
Hymenoptera	Chalcidoidea	Pteromalidae	Pteromalidae	sp.	MN666044.1
Hymenoptera	Chalcidoidea	Pteromalidae	Pteromalidae	sp.	MN666065.1
Hymenoptera	Chalcidoidea	Pteromalidae	Pteromalidae	sp.	MN666508.1
Hymenoptera	Chalcidoidea	Pteromalidae	Pteromalidae	sp.	MN666521.1
Hymenoptera	Chalcidoidea	Pteromalidae	Pteromalidae	sp.	MN666799.1
Hymenoptera	Chalcidoidea	Pteromalidae	Pteromalidae	sp.	MN666849.1
Hymenoptera	Chalcidoidea	Pteromalidae	Pteromalidae	sp.	MN666985.1
Hymenoptera	Chalcidoidea	Pteromalidae	Pteromalidae	sp.	MN667450.1
Hymenoptera	Chalcidoidea	Pteromalidae	Pteromalidae	sp.	MN667647.1
Hymenoptera	Chalcidoidea	Pteromalidae	Pteromalidae	sp.	MN667690.1
Hymenoptera	Chalcidoidea	Pteromalidae	Pteromalidae	sp.	MN667744.1
Hymenoptera	Chalcidoidea	Pteromalidae	Pteromalidae	sp.	MN667946.1
Hymenoptera	Chalcidoidea	Pteromalidae	Pteromalidae	sp.	MN668653.1
Hymenoptera	Chalcidoidea	Pteromalidae	Pteromalidae	sp.	MN668945.1
Hymenoptera	Chalcidoidea	Pteromalidae	Pteromalidae	sp.	MN669025.1
Hymenoptera	Chalcidoidea	Pteromalidae	Pteromalidae	sp.	MN669034.1
Hymenoptera	Chalcidoidea	Pteromalidae	Pteromalidae	sp.	MN669156.1
Hymenoptera	Chalcidoidea	Pteromalidae	Pteromalidae	sp.	MN669960.1
Hymenoptera	Chalcidoidea	Pteromalidae	Pteromalidae	sp.	MN670101.1
Hymenoptera	Chalcidoidea	Pteromalidae	Pteromalidae	sp.	MN670373.1
Hymenoptera	Chalcidoidea	Pteromalidae	Pteromalidae	sp.	MN670637.1
Hymenoptera	Chalcidoidea	Pteromalidae	Pteromalidae	sp.	MN671051.1
Hymenoptera	Chalcidoidea	Pteromalidae	Pteromalidae	sp.	MN671188.1
Hymenoptera	Chalcidoidea	Pteromalidae	Pteromalidae	sp.	MN671271.1
Hymenoptera	Chalcidoidea	Pteromalidae	Pteromalidae	sp.	MN672436.1
Hymenoptera	Chalcidoidea	Pteromalidae	Pteromalidae	sp.	MN672858.1
Hymenoptera	Chalcidoidea	Pteromalidae	Pteromalidae	sp.	MN672904.1

Hymenoptera	Chalcidoidea	Pteromalidae	Pteromalidae	sp.	MN673068.1
Hymenoptera	Chalcidoidea	Pteromalidae	Pteromalidae	sp.	MN673326.1
Hymenoptera	Chalcidoidea	Pteromalidae	Pteromalidae	sp.	MN673434.1
Hymenoptera	Chalcidoidea	Pteromalidae	Pteromalidae	sp.	MN673451.1
Hymenoptera	Chalcidoidea	Pteromalidae	Pteromalidae	sp.	MN673597.1
Hymenoptera	Chalcidoidea	Pteromalidae	Pteromalidae	sp.	MN673808.1
Hymenoptera	Chalcidoidea	Pteromalidae	Pteromalidae	sp.	MN674197.1
Hymenoptera	Chalcidoidea	Pteromalidae	Pteromalidae	sp.	MN674732.1
Hymenoptera	Chalcidoidea	Pteromalidae	Pteromalidae	sp.	MN674764.1
Hymenoptera	Chalcidoidea	Pteromalidae	Pteromalidae	sp.	MN674996.1
Hymenoptera	Chalcidoidea	Pteromalidae	Pteromalidae	sp.	MN675001.1
Hymenoptera	Chalcidoidea	Pteromalidae	Pteromalidae	sp.	MN675463.1
Hymenoptera	Chalcidoidea	Pteromalidae	Pteromalidae	sp.	MN675511.1
Hymenoptera	Chalcidoidea	Pteromalidae	Pteromalidae	sp.	MN675557.1
Hymenoptera	Chalcidoidea	Pteromalidae	Pteromalidae	sp.	MN675871.1
Hymenoptera	Chalcidoidea	Pteromalidae	Pteromalidae	sp.	MN675946.1
Hymenoptera	Chalcidoidea	Pteromalidae	Pteromalidae	sp.	MN676123.1
Hymenoptera	Chalcidoidea	Pteromalidae	Pteromalidae	sp.	MN676414.1
Hymenoptera	Chalcidoidea	Pteromalidae	Pteromalidae	sp.	MN676589.1
Hymenoptera	Chalcidoidea	Pteromalidae	Pteromalidae	sp.	MN676755.1
Hymenoptera	Chalcidoidea	Pteromalidae	Pteromalidae	sp.	MN676941.1
Hymenoptera	Chalcidoidea	Pteromalidae	Pteromalidae	sp.	MN677161.1
Hymenoptera	Chalcidoidea	Pteromalidae	Pteromalidae	sp.	MN677420.1
Hymenoptera	Chalcidoidea	Pteromalidae	Pteromalidae	sp.	MN677700.1
Hymenoptera	Chalcidoidea	Pteromalidae	Pteromalidae	sp.	MN677833.1
Hymenoptera	Chalcidoidea	Pteromalidae	Pteromalidae	sp.	MN678115.1
Hymenoptera	Chalcidoidea	Pteromalidae	Pteromalidae	sp.	MN678472.1
Hymenoptera	Chalcidoidea	Pteromalidae	Pteromalidae	sp.	MN678548.1
Hymenoptera	Chalcidoidea	Pteromalidae	Pteromalidae	sp.	MN678760.1
Hymenoptera	Chalcidoidea	Pteromalidae	Pteromalidae	sp.	MN679208.1
Hymenoptera	Chalcidoidea	Pteromalidae	Pteromalidae	sp.	MN679259.1
Hymenoptera	Chalcidoidea	Pteromalidae	Pteromalidae	sp.	MN679893.1
Hymenoptera	Chalcidoidea	Pteromalidae	Pteromalidae	sp.	MN679971.1
Hymenoptera	Chalcidoidea	Pteromalidae	Pteromalidae	sp.	MN680002.1
Hymenoptera	Chalcidoidea	Pteromalidae	Pteromalidae	sp.	MN680205.1
Hymenoptera	Chalcidoidea	Pteromalidae	Pteromalidae	sp.	MN680415.1
Hymenoptera	Chalcidoidea	Pteromalidae	Pteromalidae	sp.	MN680510.1
Hymenoptera	Chalcidoidea	Pteromalidae	Pteromalidae	sp.	MN681066.1
Hymenoptera	Chalcidoidea	Pteromalidae	Pteromalidae	sp.	MN681274.1
Hymenoptera	Chalcidoidea	Pteromalidae	Pteromalidae	sp.	MN681438.1
Hymenoptera	Chalcidoidea	Pteromalidae	Pteromalidae	sp.	MN682314.1
Hymenoptera	Chalcidoidea	Pteromalidae	Pteromalidae	sp.	MN682768.1
Hymenoptera	Chalcidoidea	Pteromalidae	Pteromalidae	sp.	MN682947.1
Hymenoptera	Chalcidoidea	Pteromalidae	Pteromalidae	sp.	MN683070.1
Hymenoptera	Chalcidoidea	Pteromalidae	Pteromalidae	sp.	MN683355.1
Hymenoptera	Chalcidoidea	Pteromalidae	Pteromalidae	sp.	MN683394.1
Hymenoptera	Chalcidoidea	Pteromalidae	Pteromalidae	sp.	MN683425.1
Hymenoptera	Chalcidoidea	Pteromalidae	Pteromalidae	sp.	MW784456.1
Hymenoptera	Chalcidoidea	Pteromalidae	Pteromalidae	sp.	MZ607084.1
Hymenoptera	Chalcidoidea	Pteromalidae	Pteromalidae	sp.	MZ607170.1
Hymenoptera	Chalcidoidea	Pteromalidae	Pteromalidae	sp.	MZ607251.1
Hymenoptera	Chalcidoidea	Pteromalidae	Pteromalidae	sp.	MZ607419.1
Hymenoptera	Chalcidoidea	Pteromalidae	Pteromalidae	sp.	MZ607854.1
Hymenoptera	Chalcidoidea	Pteromalidae	Pteromalidae	sp.	MZ607988.1

Hymenoptera	Chalcidoidea	Pteromalidae	Pteromalidae	sp.	MZ608107.1
Hymenoptera	Chalcidoidea	Pteromalidae	Pteromalidae	sp.	MZ608127.1
Hymenoptera	Chalcidoidea	Pteromalidae	Pteromalidae	sp.	MZ608213.1
Hymenoptera	Chalcidoidea	Pteromalidae	Pteromalidae	sp.	MZ608297.1
Hymenoptera	Chalcidoidea	Pteromalidae	Pteromalidae	sp.	MZ608504.1
Hymenoptera	Chalcidoidea	Pteromalidae	Pteromalidae	sp.	MZ608553.1
Hymenoptera	Chalcidoidea	Pteromalidae	Pteromalidae	sp.	MZ608754.1
Hymenoptera	Chalcidoidea	Pteromalidae	Pteromalidae	sp.	MZ609443.1
Hymenoptera	Chalcidoidea	Pteromalidae	Pteromalidae	sp.	MZ609677.1
Hymenoptera	Chalcidoidea	Pteromalidae	Pteromalidae	sp.	MZ609715.1
Hymenoptera	Chalcidoidea	Pteromalidae	Pteromalidae	sp.	MZ610349.1
Hymenoptera	Chalcidoidea	Pteromalidae	Pteromalidae	sp.	MZ610641.1
Hymenoptera	Chalcidoidea	Pteromalidae	Pteromalidae	sp.	MZ610862.1
Hymenoptera	Chalcidoidea	Pteromalidae	Pteromalidae	sp.	MZ630508.1
Hymenoptera	Chalcidoidea	Pteromalidae	Pteromalidae	sp.	MZ630681.1
Hymenoptera	Chalcidoidea	Pteromalidae	Pteromalidae	sp.	OL694562.1
Hymenoptera	Chalcidoidea	Pteromalidae	Pteromalinae	sp.	JN292661.1
Hymenoptera	Chalcidoidea	Pteromalidae	Pteromalinae	sp.	KM555574.1
Hymenoptera	Chalcidoidea	Pteromalidae	Pteromalinae	sp.	KM557178.1
Hymenoptera	Chalcidoidea	Pteromalidae	Pteromalinae	sp.	KM569359.1
Hymenoptera	Chalcidoidea	Pteromalidae	Pteromalinae	sp.	KR365607.1
Hymenoptera	Chalcidoidea	Pteromalidae	Pteromalinae	sp.	KR365668.1
Hymenoptera	Chalcidoidea	Pteromalidae	Pteromalinae	sp.	KR365866.1
Hymenoptera	Chalcidoidea	Pteromalidae	Pteromalinae	sp.	KR366097.1
Hymenoptera	Chalcidoidea	Pteromalidae	Pteromalinae	sp.	KR366468.1
Hymenoptera	Chalcidoidea	Pteromalidae	Pteromalinae	sp.	KR366766.1
Hymenoptera	Chalcidoidea	Pteromalidae	Pteromalinae	sp.	KR367837.1
Hymenoptera	Chalcidoidea	Pteromalidae	Pteromalinae	sp.	KR368466.1
Hymenoptera	Chalcidoidea	Pteromalidae	Pteromalinae	sp.	KR368824.1
Hymenoptera	Chalcidoidea	Pteromalidae	Pteromalinae	sp.	KR369191.1
Hymenoptera	Chalcidoidea	Pteromalidae	Pteromalinae	sp.	KR369413.1
Hymenoptera	Chalcidoidea	Pteromalidae	Pteromalinae	sp.	KR369414.1
Hymenoptera	Chalcidoidea	Pteromalidae	Pteromalinae	sp.	KR369902.1
Hymenoptera	Chalcidoidea	Pteromalidae	Pteromalinae	sp.	KR372232.1
Hymenoptera	Chalcidoidea	Pteromalidae	Pteromalinae	sp.	KR373083.1
Hymenoptera	Chalcidoidea	Pteromalidae	Pteromalinae	sp.	KR373298.1
Hymenoptera	Chalcidoidea	Pteromalidae	Pteromalinae	sp.	KR373453.1
Hymenoptera	Chalcidoidea	Pteromalidae	Pteromalinae	sp.	KR791471.1
Hymenoptera	Chalcidoidea	Pteromalidae	Pteromalinae	sp.	KR797374.1
Hymenoptera	Chalcidoidea	Pteromalidae	Pteromalinae	sp.	KR804532.1
Hymenoptera	Chalcidoidea	Pteromalidae	Pteromalinae	sp.	KR874704.1
Hymenoptera	Chalcidoidea	Pteromalidae	Pteromalinae	sp.	KR891271.1
Hymenoptera	Chalcidoidea	Pteromalidae	Pteromalinae	sp.	KR892750.1
Hymenoptera	Chalcidoidea	Pteromalidae	Pteromalinae	sp.	KR893445.1
Hymenoptera	Chalcidoidea	Pteromalidae	Pteromalinae	sp.	KR901342.1
Hymenoptera	Chalcidoidea	Pteromalidae	Pteromalinae	sp.	MG374751.1
Hymenoptera	Chalcidoidea	Pteromalidae	Pteromalinae	sp.	MG377336.1
Hymenoptera	Chalcidoidea	Pteromalidae	Pteromalinae	sp.	MG378000.1
Hymenoptera	Chalcidoidea	Pteromalidae	Pteromalinae	sp.	MG379323.1
Hymenoptera	Chalcidoidea	Pteromalidae	Pteromalinae	sp.	MG382683.1
Hymenoptera	Chalcidoidea	Pteromalidae	Pteromalinae	sp.	MT955777.1
Hymenoptera	Chalcidoidea	Pteromalidae	Pteromalus	sp.	MG784007.1
Hymenoptera	Chalcidoidea	Pteromalidae	Pteromalus	sp.	MG784035.1
Hymenoptera	Chalcidoidea	Pteromalidae	Pteromalus	altus	OL538065.1

Hymenoptera	Chalcidoidea	Pteromalidae	Pteromalus	bedeguaris	MH004457.1
Hymenoptera	Chalcidoidea	Pteromalidae	Pteromalus	cingulipes	MG784046.1
Hymenoptera	Chalcidoidea	Pteromalidae	Pteromalus	dolichurus	KT599307.1
Hymenoptera	Chalcidoidea	Pteromalidae	Pteromalus	dolichurus	KT599332.1
Hymenoptera	Chalcidoidea	Pteromalidae	Pteromalus	dolichurus	MN674987.1
Hymenoptera	Chalcidoidea	Pteromalidae	Pteromalus	elatus	KR803946.1
Hymenoptera	Chalcidoidea	Pteromalidae	Pteromalus	elatus	MG784039.1
Hymenoptera	Chalcidoidea	Pteromalidae	Pteromalus	elatus	MG784045.1
Hymenoptera	Chalcidoidea	Pteromalidae	Pteromalus	elatus	MN670873.1
Hymenoptera	Chalcidoidea	Pteromalidae	Pteromalus	elatus	MN672427.1
Hymenoptera	Chalcidoidea	Pteromalidae	Pteromalus	elatus	MN672902.1
Hymenoptera	Chalcidoidea	Pteromalidae	Pteromalus	elatus	MN675474.1
Hymenoptera	Chalcidoidea	Pteromalidae	Pteromalus	elatus	MN675488.1
Hymenoptera	Chalcidoidea	Pteromalidae	Pteromalus	elatus	MN677800.1
Hymenoptera	Chalcidoidea	Pteromalidae	Pteromalus	elatus	MN678749.1
Hymenoptera	Chalcidoidea	Pteromalidae	Pteromalus	elatus	MN679332.1
Hymenoptera	Chalcidoidea	Pteromalidae	Pteromalus	elatus	MN681307.1
Hymenoptera	Chalcidoidea	Pteromalidae	Pteromalus	elevatus	KR793889.1
Hymenoptera	Chalcidoidea	Pteromalidae	Pteromalus	hieracii	KR878107.1
Hymenoptera	Chalcidoidea	Pteromalidae	Pteromalus	intermedius	MG784023.1
Hymenoptera	Chalcidoidea	Pteromalidae	Pteromalus	phycidis	HQ107719.1
Hymenoptera	Chalcidoidea	Pteromalidae	Pteromalus	phycidis	HQ107720.1
Hymenoptera	Chalcidoidea	Pteromalidae	Pteromalus	phycidis	HQ107721.1
Hymenoptera	Chalcidoidea	Pteromalidae	Pteromalus	phycidis	HQ107722.1
Hymenoptera	Chalcidoidea	Pteromalidae	Pteromalus	phycidis	HQ107724.1
Hymenoptera	Chalcidoidea	Pteromalidae	Pteromalus	phycidis	HQ107725.1
Hymenoptera	Chalcidoidea	Pteromalidae	Pteromalus	phycidis	HQ107726.1
Hymenoptera	Chalcidoidea	Pteromalidae	Pteromalus	phycidis	HQ107727.1
Hymenoptera	Chalcidoidea	Pteromalidae	Pteromalus	phycidis	HQ107728.1
Hymenoptera	Chalcidoidea	Pteromalidae	Pteromalus	phycidis	HQ107731.1
Hymenoptera	Chalcidoidea	Pteromalidae	Pteromalus	phycidis	HQ107732.1
Hymenoptera	Chalcidoidea	Pteromalidae	Pteromalus	phycidis	HQ107733.1
Hymenoptera	Chalcidoidea	Pteromalidae	Pteromalus	phycidis	HQ107734.1
Hymenoptera	Chalcidoidea	Pteromalidae	Pteromalus	phycidis	HQ107735.1
Hymenoptera	Chalcidoidea	Pteromalidae	Pteromalus	phycidis	HQ107737.1
Hymenoptera	Chalcidoidea	Pteromalidae	Pteromalus	phycidis	HQ107742.1
Hymenoptera	Chalcidoidea	Pteromalidae	Pteromalus	phycidis	HQ107743.1
Hymenoptera	Chalcidoidea	Pteromalidae	Pteromalus	phycidis	HQ107744.1
Hymenoptera	Chalcidoidea	Pteromalidae	Pteromalus	phycidis	HQ107745.1
Hymenoptera	Chalcidoidea	Pteromalidae	Pteromalus	phycidis	HQ107748.1
Hymenoptera	Chalcidoidea	Pteromalidae	Pteromalus	phycidis	HQ107752.1
Hymenoptera	Chalcidoidea	Pteromalidae	Pteromalus	phycidis	HQ107753.1
Hymenoptera	Chalcidoidea	Pteromalidae	Pteromalus	phycidis	HQ107755.1
Hymenoptera	Chalcidoidea	Pteromalidae	Pteromalus	phycidis	HQ929583.1
Hymenoptera	Chalcidoidea	Pteromalidae	Pteromalus	phycidis	JN292345.1
Hymenoptera	Chalcidoidea	Pteromalidae	Pteromalus	phycidis	JN293137.1
Hymenoptera	Chalcidoidea	Pteromalidae	Pteromalus	phycidis	KJ092800.1
Hymenoptera	Chalcidoidea	Pteromalidae	Pteromalus	phycidis	KM556068.1
Hymenoptera	Chalcidoidea	Pteromalidae	Pteromalus	phycidis	KM559483.1
Hymenoptera	Chalcidoidea	Pteromalidae	Pteromalus	phycidis	KM559726.1
Hymenoptera	Chalcidoidea	Pteromalidae	Pteromalus	phycidis	KM560792.1
Hymenoptera	Chalcidoidea	Pteromalidae	Pteromalus	phycidis	KR787286.1
Hymenoptera	Chalcidoidea	Pteromalidae	Pteromalus	phycidis	KR793408.1
Hymenoptera	Chalcidoidea	Pteromalidae	Pteromalus	phycidis	KR882699.1

Hymenoptera	Chalcidoidea	Pteromalidae	Pteromalus	phycidis	KR883412.1
Hymenoptera	Chalcidoidea	Pteromalidae	Pteromalus	phycidis	KR895033.1
Hymenoptera	Chalcidoidea	Pteromalidae	Pteromalus	phycidis	MG376965.1
Hymenoptera	Chalcidoidea	Pteromalidae	Pteromalus	phycidis	MG500316.1
Hymenoptera	Chalcidoidea	Pteromalidae	Pteromalus	semotus	KR370441.1
Hymenoptera	Chalcidoidea	Pteromalidae	Pteromalus	sp.	HM574109.1
Hymenoptera	Chalcidoidea	Pteromalidae	Pteromalus	sp.	HM574113.1
Hymenoptera	Chalcidoidea	Pteromalidae	Pteromalus	sp.	HQ989982.1
Hymenoptera	Chalcidoidea	Pteromalidae	Pteromalus	sp.	JN292321.1
Hymenoptera	Chalcidoidea	Pteromalidae	Pteromalus	sp.	KF444823.1
Hymenoptera	Chalcidoidea	Pteromalidae	Pteromalus	sp.	KM556999.1
Hymenoptera	Chalcidoidea	Pteromalidae	Pteromalus	sp.	KM557318.1
Hymenoptera	Chalcidoidea	Pteromalidae	Pteromalus	sp.	KM557443.1
Hymenoptera	Chalcidoidea	Pteromalidae	Pteromalus	sp.	KM557529.1
Hymenoptera	Chalcidoidea	Pteromalidae	Pteromalus	sp.	KM557816.1
Hymenoptera	Chalcidoidea	Pteromalidae	Pteromalus	sp.	KM557974.1
Hymenoptera	Chalcidoidea	Pteromalidae	Pteromalus	sp.	KM558930.1
Hymenoptera	Chalcidoidea	Pteromalidae	Pteromalus	sp.	KM559986.1
Hymenoptera	Chalcidoidea	Pteromalidae	Pteromalus	sp.	KM561153.1
Hymenoptera	Chalcidoidea	Pteromalidae	Pteromalus	sp.	KM562344.1
Hymenoptera	Chalcidoidea	Pteromalidae	Pteromalus	sp.	KM562487.1
Hymenoptera	Chalcidoidea	Pteromalidae	Pteromalus	sp.	KM563201.1
Hymenoptera	Chalcidoidea	Pteromalidae	Pteromalus	sp.	KM563460.1
Hymenoptera	Chalcidoidea	Pteromalidae	Pteromalus	sp.	KM563621.1
Hymenoptera	Chalcidoidea	Pteromalidae	Pteromalus	sp.	KM564434.1
Hymenoptera	Chalcidoidea	Pteromalidae	Pteromalus	sp.	KM566159.1
Hymenoptera	Chalcidoidea	Pteromalidae	Pteromalus	sp.	KM567020.1
Hymenoptera	Chalcidoidea	Pteromalidae	Pteromalus	sp.	KR369019.1
Hymenoptera	Chalcidoidea	Pteromalidae	Pteromalus	sp.	KR782810.1
Hymenoptera	Chalcidoidea	Pteromalidae	Pteromalus	sp.	KR783431.1
Hymenoptera	Chalcidoidea	Pteromalidae	Pteromalus	sp.	KR783477.1
Hymenoptera	Chalcidoidea	Pteromalidae	Pteromalus	sp.	KR784950.1
Hymenoptera	Chalcidoidea	Pteromalidae	Pteromalus	sp.	KR786227.1
Hymenoptera	Chalcidoidea	Pteromalidae	Pteromalus	sp.	KR786452.1
Hymenoptera	Chalcidoidea	Pteromalidae	Pteromalus	sp.	KR786457.1
Hymenoptera	Chalcidoidea	Pteromalidae	Pteromalus	sp.	KR786957.1
Hymenoptera	Chalcidoidea	Pteromalidae	Pteromalus	sp.	KR787171.1
Hymenoptera	Chalcidoidea	Pteromalidae	Pteromalus	sp.	KR787402.1
Hymenoptera	Chalcidoidea	Pteromalidae	Pteromalus	sp.	KR788952.1
Hymenoptera	Chalcidoidea	Pteromalidae	Pteromalus	sp.	KR791754.1
Hymenoptera	Chalcidoidea	Pteromalidae	Pteromalus	sp.	KR792124.1
Hymenoptera	Chalcidoidea	Pteromalidae	Pteromalus	sp.	KR792200.1
Hymenoptera	Chalcidoidea	Pteromalidae	Pteromalus	sp.	KR792375.1
Hymenoptera	Chalcidoidea	Pteromalidae	Pteromalus	sp.	KR792384.1
Hymenoptera	Chalcidoidea	Pteromalidae	Pteromalus	sp.	KR792890.1
Hymenoptera	Chalcidoidea	Pteromalidae	Pteromalus	sp.	KR795134.1
Hymenoptera	Chalcidoidea	Pteromalidae	Pteromalus	sp.	KR795137.1
Hymenoptera	Chalcidoidea	Pteromalidae	Pteromalus	sp.	KR795278.1
Hymenoptera	Chalcidoidea	Pteromalidae	Pteromalus	sp.	KR795468.1
Hymenoptera	Chalcidoidea	Pteromalidae	Pteromalus	sp.	KR795658.1
Hymenoptera	Chalcidoidea	Pteromalidae	Pteromalus	sp.	KR795960.1
Hymenoptera	Chalcidoidea	Pteromalidae	Pteromalus	sp.	KR796600.1
Hymenoptera	Chalcidoidea	Pteromalidae	Pteromalus	sp.	KR796754.1
Hymenoptera	Chalcidoidea	Pteromalidae	Pteromalus	sp.	KR798016.1

Hymenoptera	Chalcidoidea	Pteromalidae	Pteromalus	sp.	KR800729.1
Hymenoptera	Chalcidoidea	Pteromalidae	Pteromalus	sp.	KR801016.1
Hymenoptera	Chalcidoidea	Pteromalidae	Pteromalus	sp.	KR804622.1
Hymenoptera	Chalcidoidea	Pteromalidae	Pteromalus	sp.	KR805240.1
Hymenoptera	Chalcidoidea	Pteromalidae	Pteromalus	sp.	KR809274.1
Hymenoptera	Chalcidoidea	Pteromalidae	Pteromalus	sp.	KR874803.1
Hymenoptera	Chalcidoidea	Pteromalidae	Pteromalus	sp.	KR878421.1
Hymenoptera	Chalcidoidea	Pteromalidae	Pteromalus	sp.	KR883983.1
Hymenoptera	Chalcidoidea	Pteromalidae	Pteromalus	sp.	KR889930.1
Hymenoptera	Chalcidoidea	Pteromalidae	Pteromalus	sp.	KR890781.1
Hymenoptera	Chalcidoidea	Pteromalidae	Pteromalus	sp.	KR892787.1
Hymenoptera	Chalcidoidea	Pteromalidae	Pteromalus	sp.	KR894550.1
Hymenoptera	Chalcidoidea	Pteromalidae	Pteromalus	sp.	KR898157.1
Hymenoptera	Chalcidoidea	Pteromalidae	Pteromalus	sp.	KR923384.1
Hymenoptera	Chalcidoidea	Pteromalidae	Pteromalus	sp.	KX281772.1
Hymenoptera	Chalcidoidea	Pteromalidae	Pteromalus	sp.	MG373791.1
Hymenoptera	Chalcidoidea	Pteromalidae	Pteromalus	sp.	MG375804.1
Hymenoptera	Chalcidoidea	Pteromalidae	Pteromalus	sp.	MG375812.1
Hymenoptera	Chalcidoidea	Pteromalidae	Pteromalus	sp.	MG377689.1
Hymenoptera	Chalcidoidea	Pteromalidae	Pteromalus	sp.	MG378772.1
Hymenoptera	Chalcidoidea	Pteromalidae	Pteromalus	sp.	MG381342.1
Hymenoptera	Chalcidoidea	Pteromalidae	Pteromalus	sp.	MG382962.1
Hymenoptera	Chalcidoidea	Pteromalidae	Pteromalus	sp.	MG497854.1
Hymenoptera	Chalcidoidea	Pteromalidae	Pteromalus	sp.	MG499508.1
Hymenoptera	Chalcidoidea	Pteromalidae	Pteromalus	sp.	MG499784.1
Hymenoptera	Chalcidoidea	Pteromalidae	Pteromalus	sp.	MG501621.1
Hymenoptera	Chalcidoidea	Pteromalidae	Pteromalus	sp.	MG506065.1
Hymenoptera	Chalcidoidea	Pteromalidae	Pteromalus	sp.	MG784020.1
Hymenoptera	Chalcidoidea	Pteromalidae	Pteromalus	sp.	MN665480.1
Hymenoptera	Chalcidoidea	Pteromalidae	Pteromalus	sp.	MN666128.1
Hymenoptera	Chalcidoidea	Pteromalidae	Pteromalus	sp.	MN667487.1
Hymenoptera	Chalcidoidea	Pteromalidae	Pteromalus	sp.	MN668016.1
Hymenoptera	Chalcidoidea	Pteromalidae	Pteromalus	sp.	MN671019.1
Hymenoptera	Chalcidoidea	Pteromalidae	Pteromalus	sp.	MN671898.1
Hymenoptera	Chalcidoidea	Pteromalidae	Pteromalus	sp.	MN672816.1
Hymenoptera	Chalcidoidea	Pteromalidae	Pteromalus	sp.	MN673752.1
Hymenoptera	Chalcidoidea	Pteromalidae	Pteromalus	sp.	MN677873.1
Hymenoptera	Chalcidoidea	Pteromalidae	Pteromalus	sp.	MN678226.1
Hymenoptera	Chalcidoidea	Pteromalidae	Pteromalus	sp.	MN678395.1
Hymenoptera	Chalcidoidea	Pteromalidae	Pteromalus	sp.	MN680163.1
Hymenoptera	Chalcidoidea	Pteromalidae	Pteromalus	sp.	MN680392.1
Hymenoptera	Chalcidoidea	Pteromalidae	Pteromalus	sp.	MN681967.1
Hymenoptera	Chalcidoidea	Pteromalidae	Pteromalus	sp.	MN682043.1
Hymenoptera	Chalcidoidea	Pteromalidae	Pteromalus	sp.	MN682988.1
Hymenoptera	Chalcidoidea	Pteromalidae	Pteromalus	sp.	MN683041.1
Hymenoptera	Chalcidoidea	Pteromalidae	Pteromalus	temporalis	MG784016.1
Hymenoptera	Chalcidoidea	Pteromalidae	Rhicnocoelia	impar	OL538055.1
Hymenoptera	Chalcidoidea	Pteromalidae	Rhicnocoelia	impar	OL538084.1
Hymenoptera	Chalcidoidea	Pteromalidae	Rohatina	inermis	OL538068.1
Hymenoptera	Chalcidoidea	Pteromalidae	Rohatina	inermis	OL538095.1
Hymenoptera	Chalcidoidea	Pteromalidae	Rohatina	inermis	OL538148.1
Hymenoptera	Chalcidoidea	Pteromalidae	Scutellista	caerulea	MH456766.1
Hymenoptera	Chalcidoidea	Pteromalidae	Seres	solweziensis	JQ756569.1
Hymenoptera	Chalcidoidea	Pteromalidae	Seres	wardi	JQ756551.1

Hymenoptera	Chalcidoidea	Pteromalidae	Sphegigaster	hamugurivora	LC542884.1
Hymenoptera	Chalcidoidea	Pteromalidae	Spintherus	sp.	MW982617.1
Hymenoptera	Chalcidoidea	Pteromalidae	Spintherus	sp.	MW982885.1
Hymenoptera	Chalcidoidea	Pteromalidae	Stichocrepis	armata	OL538062.1
Hymenoptera	Chalcidoidea	Pteromalidae	Sycobia	sp.	MK530721.1
Hymenoptera	Chalcidoidea	Pteromalidae	Sycobia	sp.	MK530722.1
Hymenoptera	Chalcidoidea	Pteromalidae	Sycobia	sp.	MK530723.1
Hymenoptera	Chalcidoidea	Pteromalidae	Sycobia	sp.	MK530724.1
Hymenoptera	Chalcidoidea	Pteromalidae	Sycobia	sp.	MK530725.1
Hymenoptera	Chalcidoidea	Pteromalidae	Sycobia	sp.	MK530727.1
Hymenoptera	Chalcidoidea	Pteromalidae	Sycoecus	sp.	JQ756553.1
Hymenoptera	Chalcidoidea	Pteromalidae	Sycoscapter	sp.	KM555753.1
Hymenoptera	Chalcidoidea	Pteromalidae	Sycoscapter	sp.	KM559658.1
Hymenoptera	Chalcidoidea	Pteromalidae	Sycoscapter	sp.	KM559793.1
Hymenoptera	Chalcidoidea	Pteromalidae	Sycoscapter	sp.	KM565776.1
Hymenoptera	Chalcidoidea	Pteromalidae	Sycoscapter	sp.	KM565975.1
Hymenoptera	Chalcidoidea	Pteromalidae	Sycoscapter	sp.	KM566008.1
Hymenoptera	Chalcidoidea	Pteromalidae	Sycoscapter	sp.	KM568612.1
Hymenoptera	Chalcidoidea	Pteromalidae	Sycoscapter	sp.	KR365323.1
Hymenoptera	Chalcidoidea	Pteromalidae	Sycoscapter	sp.	KR802916.1
Hymenoptera	Chalcidoidea	Pteromalidae	Sycoscapter	sp.	MG375533.1
Hymenoptera	Chalcidoidea	Pteromalidae	Sycoscapter	sp.	MG378144.1
Hymenoptera	Chalcidoidea	Pteromalidae	Sycoscapter	sp.	MH095556.1
Hymenoptera	Chalcidoidea	Pteromalidae	Theocolax	americanus	KJ451421.1
Hymenoptera	Chalcidoidea	Pteromalidae	Theocolax	americanus	KJ451423.1
Hymenoptera	Chalcidoidea	Pteromalidae	Theocolax	elegans	KJ451419.1
Hymenoptera	Chalcidoidea	Pteromalidae	Theocolax	sp.	KX809932.1
Hymenoptera	Chalcidoidea	Pteromalidae	Theocolax	sp.	KX809934.1
Hymenoptera	Chalcidoidea	Pteromalidae	Thinodytes	sp.	KM556640.1
Hymenoptera	Chalcidoidea	Pteromalidae	Thinodytes	sp.	KM566399.1
Hymenoptera	Chalcidoidea	Pteromalidae	Thinodytes	sp.	MG376348.1
Hymenoptera	Chalcidoidea	Pteromalidae	Thinodytes	sp.	MG378206.1
Hymenoptera	Chalcidoidea	Pteromalidae	Toxeuma	discretum	OL538101.1
Hymenoptera	Chalcidoidea	Pteromalidae	Toxeuma	discretum	OL538112.1
Hymenoptera	Chalcidoidea	Pteromalidae	Toxeuma	discretum	OL538116.1
Hymenoptera	Chalcidoidea	Pteromalidae	Tricyclomischus	celticus	OL538053.1
Hymenoptera	Chalcidoidea	Pteromalidae	Tricyclomischus	celticus	OL538077.1
Hymenoptera	Chalcidoidea	Pteromalidae	Trychnosoma	punctipleura	OL538099.1
Hymenoptera	Chalcidoidea	Pteromalidae	Walkerella	benjamini	MK530768.1
Hymenoptera	Chalcidoidea	Pteromalidae	Walkerella	benjamini	MK530769.1
Hymenoptera	Chalcidoidea	Pteromalidae	Walkerella	curtipedis	MK530761.1
Hymenoptera	Chalcidoidea	Pteromalidae	Walkerella	microcarpae	MK530770.1
Hymenoptera	Chalcidoidea	Pteromalidae	Walkerella	microcarpae	MK530772.1
Hymenoptera	Chalcidoidea	Pteromalidae	Walkerella	nigrabdomina	MK530764.1
Hymenoptera	Chalcidoidea	Pteromalidae	Walkerella	nigrabdomina	MK530765.1
Hymenoptera	Chalcidoidea	Pteromalidae	Walkerella	nigrabdomina	MK530766.1
Hymenoptera	Chalcidoidea	Pteromalidae	Walkerella	nigrabdomina	MK543433.1
Hymenoptera	Chalcidoidea	Pteromalidae	Walkerella	sp.	MK530762.1
Hymenoptera	Chalcidoidea	Pteromalidae	Walkerella	sp.	MK530763.1
Hymenoptera	Chalcidoidea	Signiphoridae	Chartocerus	niger	MH456579.1
Hymenoptera	Chalcidoidea	Signiphoridae	Chartocerus	sp.	KR809410.1
Hymenoptera	Chalcidoidea	Signiphoridae	Chartocerus	sp.	MH407238.1
Hymenoptera	Chalcidoidea	Signiphoridae	Chartocerus	sp.	MH407241.1
Hymenoptera	Chalcidoidea	Signiphoridae	Chartocerus	sp.	MH407242.1

Hymenoptera	Chalcidoidea	Signiphoridae	Chartocerus	sp.	MN698735.1
Hymenoptera	Chalcidoidea	Signiphoridae	Signiphora	bifasciata	MH456703.1
Hymenoptera	Chalcidoidea	Signiphoridae	Signiphora	flavella	MH456540.1
Hymenoptera	Chalcidoidea	Signiphoridae	Signiphora	perpauca	MH456745.1
Hymenoptera	Chalcidoidea	Signiphoridae	Signiphora	sp.	MH456674.1
Hymenoptera	Chalcidoidea	Signiphoridae	Signiphora	sp.	MH456758.1
Hymenoptera	Chalcidoidea	Signiphoridae	Signiphoridae	sp.	KR875663.1
Hymenoptera	Chalcidoidea	Signiphoridae	Signiphoridae	sp.	KR896603.1
Hymenoptera	Chalcidoidea	Signiphoridae	Signiphoridae	sp.	KR900496.1
Hymenoptera	Chalcidoidea	Signiphoridae	Signiphoridae	sp.	MH407234.1
Hymenoptera	Chalcidoidea	Signiphoridae	Signiphoridae	sp.	MH407235.1
Hymenoptera	Chalcidoidea	Signiphoridae	Signiphoridae	sp.	MH407236.1
Hymenoptera	Chalcidoidea	Signiphoridae	Signiphoridae	sp.	MH407239.1
Hymenoptera	Chalcidoidea	Signiphoridae	Signiphoridae	sp.	MH456749.1
Hymenoptera	Chalcidoidea	Signiphoridae	Signiphoridae	sp.	MH456754.1
Hymenoptera	Chalcidoidea	Signiphoridae	Signiphoridae	sp.	MH456785.1
Hymenoptera	Chalcidoidea	Signiphoridae	Signiphoridae	sp.	MH456786.1
Hymenoptera	Chalcidoidea	Tanaostigmatidae	Tanaostigmatidae	sp.	MG497447.1
Hymenoptera	Chalcidoidea	Tetracampidae	Foersterella	reptans	KC213238.1
Hymenoptera	Chalcidoidea	Tetracampidae	Tetracampe	sp.	HM365027.1
Hymenoptera	Chalcidoidea	Torymidae	Glyphomerus	stigma	KJ012452.1
Hymenoptera	Chalcidoidea	Torymidae	Monodontomerus	sp.	KR897915.1
Hymenoptera	Chalcidoidea	Torymidae	Monodontomerus	sp.	MG380891.1
Hymenoptera	Chalcidoidea	Torymidae	Torymidae	sp.	HM374832.1
Hymenoptera	Chalcidoidea	Torymidae	Torymidae	sp.	HM414490.1
Hymenoptera	Chalcidoidea	Torymidae	Torymidae	sp.	HQ929595.1
Hymenoptera	Chalcidoidea	Torymidae	Torymidae	sp.	HQ929600.1
Hymenoptera	Chalcidoidea	Torymidae	Torymidae	sp.	HQ930254.1
Hymenoptera	Chalcidoidea	Torymidae	Torymidae	sp.	HQ930256.1
Hymenoptera	Chalcidoidea	Torymidae	Torymidae	sp.	HQ930257.1
Hymenoptera	Chalcidoidea	Torymidae	Torymidae	sp.	JN292241.1
Hymenoptera	Chalcidoidea	Torymidae	Torymidae	sp.	JN292309.1
Hymenoptera	Chalcidoidea	Torymidae	Torymidae	sp.	KM560622.1
Hymenoptera	Chalcidoidea	Torymidae	Torymidae	sp.	KM561407.1
Hymenoptera	Chalcidoidea	Torymidae	Torymidae	sp.	KM563247.1
Hymenoptera	Chalcidoidea	Torymidae	Torymidae	sp.	KM566174.1
Hymenoptera	Chalcidoidea	Torymidae	Torymidae	sp.	KM567248.1
Hymenoptera	Chalcidoidea	Torymidae	Torymidae	sp.	KR366608.1
Hymenoptera	Chalcidoidea	Torymidae	Torymidae	sp.	KR369011.1
Hymenoptera	Chalcidoidea	Torymidae	Torymidae	sp.	KR370017.1
Hymenoptera	Chalcidoidea	Torymidae	Torymidae	sp.	KR372963.1
Hymenoptera	Chalcidoidea	Torymidae	Torymidae	sp.	KR786974.1
Hymenoptera	Chalcidoidea	Torymidae	Torymidae	sp.	KR787257.1
Hymenoptera	Chalcidoidea	Torymidae	Torymidae	sp.	KR788474.1
Hymenoptera	Chalcidoidea	Torymidae	Torymidae	sp.	KR794178.1
Hymenoptera	Chalcidoidea	Torymidae	Torymidae	sp.	KR797316.1
Hymenoptera	Chalcidoidea	Torymidae	Torymidae	sp.	KR798030.1
Hymenoptera	Chalcidoidea	Torymidae	Torymidae	sp.	KR806867.1
Hymenoptera	Chalcidoidea	Torymidae	Torymidae	sp.	KR806931.1
Hymenoptera	Chalcidoidea	Torymidae	Torymidae	sp.	KR883204.1
Hymenoptera	Chalcidoidea	Torymidae	Torymidae	sp.	KR885158.1
Hymenoptera	Chalcidoidea	Torymidae	Torymidae	sp.	KR887110.1
Hymenoptera	Chalcidoidea	Torymidae	Torymidae	sp.	KR890820.1
Hymenoptera	Chalcidoidea	Torymidae	Torymidae	sp.	KR891502.1

Hymenoptera	Chalcidoidea	Torymidae	Torymidae	sp.	KR894344.1
Hymenoptera	Chalcidoidea	Torymidae	Torymidae	sp.	KR924088.1
Hymenoptera	Chalcidoidea	Torymidae	Torymidae	sp.	KR924673.1
Hymenoptera	Chalcidoidea	Torymidae	Torymidae	sp.	KR929433.1
Hymenoptera	Chalcidoidea	Torymidae	Torymidae	sp.	KR932671.1
Hymenoptera	Chalcidoidea	Torymidae	Torymidae	sp.	MG375765.1
Hymenoptera	Chalcidoidea	Torymidae	Torymidae	sp.	MG376472.1
Hymenoptera	Chalcidoidea	Torymidae	Torymidae	sp.	MG377356.1
Hymenoptera	Chalcidoidea	Torymidae	Torymidae	sp.	MG377769.1
Hymenoptera	Chalcidoidea	Torymidae	Torymidae	sp.	MG378086.1
Hymenoptera	Chalcidoidea	Torymidae	Torymidae	sp.	MG379576.1
Hymenoptera	Chalcidoidea	Torymidae	Torymidae	sp.	MG379872.1
Hymenoptera	Chalcidoidea	Torymidae	Torymidae	sp.	MG380086.1
Hymenoptera	Chalcidoidea	Torymidae	Torymidae	sp.	MG497241.1
Hymenoptera	Chalcidoidea	Torymidae	Torymidae	sp.	MG498708.1
Hymenoptera	Chalcidoidea	Torymidae	Torymidae	sp.	MG498784.1
Hymenoptera	Chalcidoidea	Torymidae	Torymidae	sp.	MG502680.1
Hymenoptera	Chalcidoidea	Torymidae	Torymidae	sp.	MG503077.1
Hymenoptera	Chalcidoidea	Torymidae	Torymidae	sp.	MG503118.1
Hymenoptera	Chalcidoidea	Torymidae	Torymidae	sp.	MG503128.1
Hymenoptera	Chalcidoidea	Torymidae	Torymidae	sp.	MG504367.1
Hymenoptera	Chalcidoidea	Torymidae	Torymidae	sp.	MG504630.1
Hymenoptera	Chalcidoidea	Torymidae	Torymidae	sp.	MG506112.1
Hymenoptera	Chalcidoidea	Torymidae	Torymidae	sp.	MG511928.1
Hymenoptera	Chalcidoidea	Torymidae	Torymidae	sp.	MH928811.1
Hymenoptera	Chalcidoidea	Torymidae	Torymidae	sp.	MW784478.1
Hymenoptera	Chalcidoidea	Torymidae	Toryminae	sp.	KM560008.1
Hymenoptera	Chalcidoidea	Torymidae	Toryminae	sp.	KR365015.1
Hymenoptera	Chalcidoidea	Torymidae	Toryminae	sp.	KR365970.1
Hymenoptera	Chalcidoidea	Torymidae	Toryminae	sp.	KR368304.1
Hymenoptera	Chalcidoidea	Torymidae	Toryminae	sp.	KR368750.1
Hymenoptera	Chalcidoidea	Torymidae	Toryminae	sp.	KR369149.1
Hymenoptera	Chalcidoidea	Torymidae	Toryminae	sp.	KR369997.1
Hymenoptera	Chalcidoidea	Torymidae	Toryminae	sp.	KR369999.1
Hymenoptera	Chalcidoidea	Torymidae	Toryminae	sp.	KR370385.1
Hymenoptera	Chalcidoidea	Torymidae	Toryminae	sp.	KR370829.1
Hymenoptera	Chalcidoidea	Torymidae	Toryminae	sp.	KR370859.1
Hymenoptera	Chalcidoidea	Torymidae	Toryminae	sp.	KR373826.1
Hymenoptera	Chalcidoidea	Torymidae	Toryminae	sp.	KR374658.1
Hymenoptera	Chalcidoidea	Torymidae	Toryminae	sp.	KR877795.1
Hymenoptera	Chalcidoidea	Torymidae	Toryminae	sp.	KR882134.1
Hymenoptera	Chalcidoidea	Torymidae	Toryminae	sp.	MG381541.1
Hymenoptera	Chalcidoidea	Torymidae	Toryminae	sp.	MG499820.1
Hymenoptera	Chalcidoidea	Torymidae	Torymus	azureus	KM558635.1
Hymenoptera	Chalcidoidea	Torymidae	Torymus	cecidomyae	KU496852.1
Hymenoptera	Chalcidoidea	Torymidae	Torymus	chrysochlorus	KM557679.1
Hymenoptera	Chalcidoidea	Torymidae	Torymus	chrysochlorus	MG379837.1
Hymenoptera	Chalcidoidea	Torymidae	Torymus	geranii	MZ629775.1
Hymenoptera	Chalcidoidea	Torymidae	Torymus	sp.	HM374742.1
Hymenoptera	Chalcidoidea	Torymidae	Torymus	sp.	JN292346.1
Hymenoptera	Chalcidoidea	Torymidae	Torymus	sp.	KM555441.1
Hymenoptera	Chalcidoidea	Torymidae	Torymus	sp.	KM556224.1
Hymenoptera	Chalcidoidea	Torymidae	Torymus	sp.	KM556756.1
Hymenoptera	Chalcidoidea	Torymidae	Torymus	sp.	KM557077.1

Hymenoptera	Chalcidoidea	Torymidae	Torymus	sp.	KM558962.1
Hymenoptera	Chalcidoidea	Torymidae	Torymus	sp.	KM559010.1
Hymenoptera	Chalcidoidea	Torymidae	Torymus	sp.	KM561396.1
Hymenoptera	Chalcidoidea	Torymidae	Torymus	sp.	KM561410.1
Hymenoptera	Chalcidoidea	Torymidae	Torymus	sp.	KM563276.1
Hymenoptera	Chalcidoidea	Torymidae	Torymus	sp.	KM566071.1
Hymenoptera	Chalcidoidea	Torymidae	Torymus	sp.	KM569308.1
Hymenoptera	Chalcidoidea	Torymidae	Torymus	sp.	KR365100.1
Hymenoptera	Chalcidoidea	Torymidae	Torymus	sp.	KR366010.1
Hymenoptera	Chalcidoidea	Torymidae	Torymus	sp.	KR370131.1
Hymenoptera	Chalcidoidea	Torymidae	Torymus	sp.	KR370726.1
Hymenoptera	Chalcidoidea	Torymidae	Torymus	sp.	KR371879.1
Hymenoptera	Chalcidoidea	Torymidae	Torymus	sp.	KR372972.1
Hymenoptera	Chalcidoidea	Torymidae	Torymus	sp.	KR373422.1
Hymenoptera	Chalcidoidea	Torymidae	Torymus	sp.	KR374163.1
Hymenoptera	Chalcidoidea	Torymidae	Torymus	sp.	KR791499.1
Hymenoptera	Chalcidoidea	Torymidae	Torymus	sp.	KR793581.1
Hymenoptera	Chalcidoidea	Torymidae	Torymus	sp.	KR808465.1
Hymenoptera	Chalcidoidea	Torymidae	Torymus	sp.	KR876075.1
Hymenoptera	Chalcidoidea	Torymidae	Torymus	sp.	KR887886.1
Hymenoptera	Chalcidoidea	Torymidae	Torymus	sp.	KR933101.1
Hymenoptera	Chalcidoidea	Torymidae	Torymus	sp.	KR933739.1
Hymenoptera	Chalcidoidea	Torymidae	Torymus	sp.	KT266710.1
Hymenoptera	Chalcidoidea	Torymidae	Torymus	sp.	KT599308.1
Hymenoptera	Chalcidoidea	Torymidae	Torymus	sp.	MG373925.1
Hymenoptera	Chalcidoidea	Torymidae	Torymus	sp.	MG374738.1
Hymenoptera	Chalcidoidea	Torymidae	Torymus	sp.	MG378799.1
Hymenoptera	Chalcidoidea	Torymidae	Torymus	sp.	MG379140.1
Hymenoptera	Chalcidoidea	Torymidae	Torymus	sp.	MG379272.1
Hymenoptera	Chalcidoidea	Torymidae	Torymus	sp.	MG380497.1
Hymenoptera	Chalcidoidea	Torymidae	Torymus	sp.	MG504813.1
Hymenoptera	Chalcidoidea	Torymidae	Torymus	sp.	MG505868.1
Hymenoptera	Chalcidoidea	Torymidae	Torymus	sp.	MG506313.1
Hymenoptera	Chalcidoidea	Torymidae	Torymus	tubicola	KR108713.1
Hymenoptera	Chalcidoidea	Torymidae	Torymus	tubicola	KR108715.1
Hymenoptera	Chalcidoidea	Torymidae	Torymus	tubicola	KR108717.1
Hymenoptera	Chalcidoidea	Torymidae	Torymus	tubicola	KR108730.1
Hymenoptera	Chalcidoidea	Torymidae	Torymus	tubicola	KR108731.1
Hymenoptera	Chalcidoidea	Torymidae	Torymus	tubicola	KR108732.1
Hymenoptera	Chalcidoidea	Trichogrammatidae	Oligosita	sp.	MG904893.1
Hymenoptera	Chalcidoidea	Trichogrammatidae	Oligosita	sp.	MG904896.1
Hymenoptera	Chalcidoidea	Trichogrammatidae	Oligosita	sp.	MG904897.1
Hymenoptera	Chalcidoidea	Trichogrammatidae	Oligosita	sp.	MG904914.1
Hymenoptera	Chalcidoidea	Trichogrammatidae	Oligosita	sp.	MG904917.1
Hymenoptera	Chalcidoidea	Trichogrammatidae	Paracentrobia	sp.	MG904920.1
Hymenoptera	Chalcidoidea	Trichogrammatidae	Paracentrobia	sp.	MG904934.1
Hymenoptera	Chalcidoidea	Trichogrammatidae	Trichogramma	achaeae	KP994548.1
Hymenoptera	Chalcidoidea	Trichogrammatidae	Trichogramma	brassicae	FM210196.1
Hymenoptera	Chalcidoidea	Trichogrammatidae	Trichogramma	brassicae	FM210197.1
Hymenoptera	Chalcidoidea	Trichogrammatidae	Trichogramma	brassicae	FM210198.1
Hymenoptera	Chalcidoidea	Trichogrammatidae	Trichogramma	brassicae	GU975842.2
Hymenoptera	Chalcidoidea	Trichogrammatidae	Trichogramma	brassicae	JX131627.1
Hymenoptera	Chalcidoidea	Trichogrammatidae	Trichogramma	brassicae	JX442923.1
Hymenoptera	Chalcidoidea	Trichogrammatidae	Trichogramma	brassicae	KM220523.1

Hymenoptera	Chalcidoidea	Trichogrammatidae	Trichogramma	brassicae	KM242284.1
Hymenoptera	Chalcidoidea	Trichogrammatidae	Trichogramma	brassicae	MG850863.1
Hymenoptera	Chalcidoidea	Trichogrammatidae	Trichogramma	brassicae	MG850864.1
Hymenoptera	Chalcidoidea	Trichogrammatidae	Trichogramma	brassicae	MG850865.1
Hymenoptera	Chalcidoidea	Trichogrammatidae	Trichogramma	brassicae	MG850866.1
Hymenoptera	Chalcidoidea	Trichogrammatidae	Trichogramma	brassicae	MG850867.1
Hymenoptera	Chalcidoidea	Trichogrammatidae	Trichogramma	brassicae	MG850868.1
Hymenoptera	Chalcidoidea	Trichogrammatidae	Trichogramma	brassicae	MG850870.1
Hymenoptera	Chalcidoidea	Trichogrammatidae	Trichogramma	brassicae	MG850871.1
Hymenoptera	Chalcidoidea	Trichogrammatidae	Trichogramma	brassicae	MG850872.1
Hymenoptera	Chalcidoidea	Trichogrammatidae	Trichogramma	brassicae	MG850873.1
Hymenoptera	Chalcidoidea	Trichogrammatidae	Trichogramma	brassicae	MG850875.1
Hymenoptera	Chalcidoidea	Trichogrammatidae	Trichogramma	cacaeciae	KM559290.1
Hymenoptera	Chalcidoidea	Trichogrammatidae	Trichogramma	cacaeciae	KM566734.1
Hymenoptera	Chalcidoidea	Trichogrammatidae	Trichogramma	cacoeciae	KM242285.1
Hymenoptera	Chalcidoidea	Trichogrammatidae	Trichogramma	chilonis	KM259632.1
Hymenoptera	Chalcidoidea	Trichogrammatidae	Trichogramma	chilonis	KP994546.1
Hymenoptera	Chalcidoidea	Trichogrammatidae	Trichogramma	chilonis	KU575095.1
Hymenoptera	Chalcidoidea	Trichogrammatidae	Trichogramma	chilonis	KY836727.1
Hymenoptera	Chalcidoidea	Trichogrammatidae	Trichogramma	chilonis	KY872646.1
Hymenoptera	Chalcidoidea	Trichogrammatidae	Trichogramma	chilonis	MK611825.1
Hymenoptera	Chalcidoidea	Trichogrammatidae	Trichogramma	chilonis	MK611826.1
Hymenoptera	Chalcidoidea	Trichogrammatidae	Trichogramma	chilonis	MK611827.1
Hymenoptera	Chalcidoidea	Trichogrammatidae	Trichogramma	chilonis	MK611830.1
Hymenoptera	Chalcidoidea	Trichogrammatidae	Trichogramma	chilonis	MK611831.1
Hymenoptera	Chalcidoidea	Trichogrammatidae	Trichogramma	chilotraeae	KP090266.1
Hymenoptera	Chalcidoidea	Trichogrammatidae	Trichogramma	cordubensis	KM232610.1
Hymenoptera	Chalcidoidea	Trichogrammatidae	Trichogramma	danaidiphaga	KP994545.1
Hymenoptera	Chalcidoidea	Trichogrammatidae	Trichogramma	danaidiphaga	KT305958.1
Hymenoptera	Chalcidoidea	Trichogrammatidae	Trichogramma	danausicida	JF776380.1
Hymenoptera	Chalcidoidea	Trichogrammatidae	Trichogramma	danausicida	KM105168.1
Hymenoptera	Chalcidoidea	Trichogrammatidae	Trichogramma	dendrolimi	MK611824.1
Hymenoptera	Chalcidoidea	Trichogrammatidae	Trichogramma	embryophagum	KC488685.1
Hymenoptera	Chalcidoidea	Trichogrammatidae	Trichogramma	embryophagum	KM105169.1
Hymenoptera	Chalcidoidea	Trichogrammatidae	Trichogramma	evanescens	GQ367960.1
Hymenoptera	Chalcidoidea	Trichogrammatidae	Trichogramma	evanescens	JX131626.1
Hymenoptera	Chalcidoidea	Trichogrammatidae	Trichogramma	evanescens	KM105170.1
Hymenoptera	Chalcidoidea	Trichogrammatidae	Trichogramma	evanescens	KP127627.1
Hymenoptera	Chalcidoidea	Trichogrammatidae	Trichogramma	hebbalensis	KP090265.1
Hymenoptera	Chalcidoidea	Trichogrammatidae	Trichogramma	japonicum	KM220522.1
Hymenoptera	Chalcidoidea	Trichogrammatidae	Trichogramma	japonicum	KP994544.1
Hymenoptera	Chalcidoidea	Trichogrammatidae	Trichogramma	mwanzai	KP142716.1
Hymenoptera	Chalcidoidea	Trichogrammatidae	Trichogramma	pintoi	MN668169.1
Hymenoptera	Chalcidoidea	Trichogrammatidae	Trichogramma	pintoi	MN668755.1
Hymenoptera	Chalcidoidea	Trichogrammatidae	Trichogramma	pintoi	MN669480.1
Hymenoptera	Chalcidoidea	Trichogrammatidae	Trichogramma	pintoi	MN675231.1
Hymenoptera	Chalcidoidea	Trichogrammatidae	Trichogramma	pintoi	MN678844.1
Hymenoptera	Chalcidoidea	Trichogrammatidae	Trichogramma	pintoi	MN680701.1
Hymenoptera	Chalcidoidea	Trichogrammatidae	Trichogramma	pintoi	MN681679.1
Hymenoptera	Chalcidoidea	Trichogrammatidae	Trichogramma	platneri	KJ083959.1
Hymenoptera	Chalcidoidea	Trichogrammatidae	Trichogramma	platneri	KJ084938.1
Hymenoptera	Chalcidoidea	Trichogrammatidae	Trichogramma	platneri	KJ088919.1
Hymenoptera	Chalcidoidea	Trichogrammatidae	Trichogramma	platneri	KJ090108.1
Hymenoptera	Chalcidoidea	Trichogrammatidae	Trichogramma	platneri	KJ092512.1

Hymenoptera	Chalcidoidea	Trichogrammatidae	Trichogramma	platneri	KR365401.1
Hymenoptera	Chalcidoidea	Trichogrammatidae	Trichogramma	platneri	KR366150.1
Hymenoptera	Chalcidoidea	Trichogrammatidae	Trichogramma	platneri	KR367409.1
Hymenoptera	Chalcidoidea	Trichogrammatidae	Trichogramma	platneri	KR368680.1
Hymenoptera	Chalcidoidea	Trichogrammatidae	Trichogramma	platneri	KR375382.1
Hymenoptera	Chalcidoidea	Trichogrammatidae	Trichogramma	platneri	KR794241.1
Hymenoptera	Chalcidoidea	Trichogrammatidae	Trichogramma	platneri	KR797366.1
Hymenoptera	Chalcidoidea	Trichogrammatidae	Trichogramma	platneri	KR808681.1
Hymenoptera	Chalcidoidea	Trichogrammatidae	Trichogramma	platneri	KR894563.1
Hymenoptera	Chalcidoidea	Trichogrammatidae	Trichogramma	platneri	KR935092.1
Hymenoptera	Chalcidoidea	Trichogrammatidae	Trichogramma	platneri	KT614906.1
Hymenoptera	Chalcidoidea	Trichogrammatidae	Trichogramma	platneri	KT616690.1
Hymenoptera	Chalcidoidea	Trichogrammatidae	Trichogramma	platneri	KT617717.1
Hymenoptera	Chalcidoidea	Trichogrammatidae	Trichogramma	platneri	KT617750.1
Hymenoptera	Chalcidoidea	Trichogrammatidae	Trichogramma	platneri	KT618154.1
Hymenoptera	Chalcidoidea	Trichogrammatidae	Trichogramma	platneri	KX512837.1
Hymenoptera	Chalcidoidea	Trichogrammatidae	Trichogramma	platneri	KX512838.1
Hymenoptera	Chalcidoidea	Trichogrammatidae	Trichogramma	platneri	KX512839.1
Hymenoptera	Chalcidoidea	Trichogrammatidae	Trichogramma	platneri	KX512840.1
Hymenoptera	Chalcidoidea	Trichogrammatidae	Trichogramma	platneri	KX512841.1
Hymenoptera	Chalcidoidea	Trichogrammatidae	Trichogramma	platneri	KX512842.1
Hymenoptera	Chalcidoidea	Trichogrammatidae	Trichogramma	platneri	MG498198.1
Hymenoptera	Chalcidoidea	Trichogrammatidae	Trichogramma	platneri	MG498267.1
Hymenoptera	Chalcidoidea	Trichogrammatidae	Trichogramma	platneri	MG498401.1
Hymenoptera	Chalcidoidea	Trichogrammatidae	Trichogramma	platneri	MG499709.1
Hymenoptera	Chalcidoidea	Trichogrammatidae	Trichogramma	platneri	MG500705.1
Hymenoptera	Chalcidoidea	Trichogrammatidae	Trichogramma	platneri	MG501174.1
Hymenoptera	Chalcidoidea	Trichogrammatidae	Trichogramma	platneri	MG501415.1
Hymenoptera	Chalcidoidea	Trichogrammatidae	Trichogramma	platneri	MG501607.1
Hymenoptera	Chalcidoidea	Trichogrammatidae	Trichogramma	platneri	MG502032.1
Hymenoptera	Chalcidoidea	Trichogrammatidae	Trichogramma	platneri	MG503855.1
Hymenoptera	Chalcidoidea	Trichogrammatidae	Trichogramma	platneri	MG505203.1
Hymenoptera	Chalcidoidea	Trichogrammatidae	Trichogramma	platneri	MG506190.1
Hymenoptera	Chalcidoidea	Trichogrammatidae	Trichogramma	platneri	MG506632.1
Hymenoptera	Chalcidoidea	Trichogrammatidae	Trichogramma	pretiosum	KF573370.1
Hymenoptera	Chalcidoidea	Trichogrammatidae	Trichogramma	pretiosum	KF573371.1
Hymenoptera	Chalcidoidea	Trichogrammatidae	Trichogramma	pretiosum	KF573372.1
Hymenoptera	Chalcidoidea	Trichogrammatidae	Trichogramma	pretiosum	KF573373.1
Hymenoptera	Chalcidoidea	Trichogrammatidae	Trichogramma	pretiosum	KF573374.1
Hymenoptera	Chalcidoidea	Trichogrammatidae	Trichogramma	pretiosum	KF573375.1
Hymenoptera	Chalcidoidea	Trichogrammatidae	Trichogramma	pretiosum	KF573376.1
Hymenoptera	Chalcidoidea	Trichogrammatidae	Trichogramma	pretiosum	KF573378.1
Hymenoptera	Chalcidoidea	Trichogrammatidae	Trichogramma	pretiosum	KF573379.1
Hymenoptera	Chalcidoidea	Trichogrammatidae	Trichogramma	pretiosum	KF573380.1
Hymenoptera	Chalcidoidea	Trichogrammatidae	Trichogramma	pretiosum	KF573382.1
Hymenoptera	Chalcidoidea	Trichogrammatidae	Trichogramma	pretiosum	KF573393.1
Hymenoptera	Chalcidoidea	Trichogrammatidae	Trichogramma	pretiosum	KF573403.1
Hymenoptera	Chalcidoidea	Trichogrammatidae	Trichogramma	pretiosum	KF573404.1
Hymenoptera	Chalcidoidea	Trichogrammatidae	Trichogramma	pretiosum	KF573406.1
Hymenoptera	Chalcidoidea	Trichogrammatidae	Trichogramma	pretiosum	KM232609.1
Hymenoptera	Chalcidoidea	Trichogrammatidae	Trichogramma	pretiosum	KU702640.2
Hymenoptera	Chalcidoidea	Trichogrammatidae	Trichogramma	pretiosum	KU702644.2
Hymenoptera	Chalcidoidea	Trichogrammatidae	Trichogramma	pretiosum	KU702648.2
Hymenoptera	Chalcidoidea	Trichogrammatidae	Trichogramma	semblidis	KM259631.1

Hymenoptera	Chalcidoidea	Trichogrammatidae	Trichogramma	sp.	HM374093.1
Hymenoptera	Chalcidoidea	Trichogrammatidae	Trichogramma	sp.	HM374094.1
Hymenoptera	Chalcidoidea	Trichogrammatidae	Trichogramma	sp.	JN292899.1
Hymenoptera	Chalcidoidea	Trichogrammatidae	Trichogramma	sp.	JN293529.1
Hymenoptera	Chalcidoidea	Trichogrammatidae	Trichogramma	sp.	KJ444952.1
Hymenoptera	Chalcidoidea	Trichogrammatidae	Trichogramma	sp.	KM555556.1
Hymenoptera	Chalcidoidea	Trichogrammatidae	Trichogramma	sp.	KM555673.1
Hymenoptera	Chalcidoidea	Trichogrammatidae	Trichogramma	sp.	KM556376.1
Hymenoptera	Chalcidoidea	Trichogrammatidae	Trichogramma	sp.	KM556538.1
Hymenoptera	Chalcidoidea	Trichogrammatidae	Trichogramma	sp.	KM556608.1
Hymenoptera	Chalcidoidea	Trichogrammatidae	Trichogramma	sp.	KM557019.1
Hymenoptera	Chalcidoidea	Trichogrammatidae	Trichogramma	sp.	KM557061.1
Hymenoptera	Chalcidoidea	Trichogrammatidae	Trichogramma	sp.	KM558125.1
Hymenoptera	Chalcidoidea	Trichogrammatidae	Trichogramma	sp.	KM558312.1
Hymenoptera	Chalcidoidea	Trichogrammatidae	Trichogramma	sp.	KM559031.1
Hymenoptera	Chalcidoidea	Trichogrammatidae	Trichogramma	sp.	KM559414.1
Hymenoptera	Chalcidoidea	Trichogrammatidae	Trichogramma	sp.	KM559536.1
Hymenoptera	Chalcidoidea	Trichogrammatidae	Trichogramma	sp.	KM560032.1
Hymenoptera	Chalcidoidea	Trichogrammatidae	Trichogramma	sp.	KM560249.1
Hymenoptera	Chalcidoidea	Trichogrammatidae	Trichogramma	sp.	KM560267.1
Hymenoptera	Chalcidoidea	Trichogrammatidae	Trichogramma	sp.	KM560281.1
Hymenoptera	Chalcidoidea	Trichogrammatidae	Trichogramma	sp.	KM560511.1
Hymenoptera	Chalcidoidea	Trichogrammatidae	Trichogramma	sp.	KM561788.1
Hymenoptera	Chalcidoidea	Trichogrammatidae	Trichogramma	sp.	KM561935.1
Hymenoptera	Chalcidoidea	Trichogrammatidae	Trichogramma	sp.	KM562032.1
Hymenoptera	Chalcidoidea	Trichogrammatidae	Trichogramma	sp.	KM562343.1
Hymenoptera	Chalcidoidea	Trichogrammatidae	Trichogramma	sp.	KM563126.1
Hymenoptera	Chalcidoidea	Trichogrammatidae	Trichogramma	sp.	KM563528.1
Hymenoptera	Chalcidoidea	Trichogrammatidae	Trichogramma	sp.	KM563877.1
Hymenoptera	Chalcidoidea	Trichogrammatidae	Trichogramma	sp.	KM564152.1
Hymenoptera	Chalcidoidea	Trichogrammatidae	Trichogramma	sp.	KM564232.1
Hymenoptera	Chalcidoidea	Trichogrammatidae	Trichogramma	sp.	KM564449.1
Hymenoptera	Chalcidoidea	Trichogrammatidae	Trichogramma	sp.	KM565757.1
Hymenoptera	Chalcidoidea	Trichogrammatidae	Trichogramma	sp.	KM566250.1
Hymenoptera	Chalcidoidea	Trichogrammatidae	Trichogramma	sp.	KM566472.1
Hymenoptera	Chalcidoidea	Trichogrammatidae	Trichogramma	sp.	KM567360.1
Hymenoptera	Chalcidoidea	Trichogrammatidae	Trichogramma	sp.	KM568100.1
Hymenoptera	Chalcidoidea	Trichogrammatidae	Trichogramma	sp.	KM568606.1
Hymenoptera	Chalcidoidea	Trichogrammatidae	Trichogramma	sp.	KM568990.1
Hymenoptera	Chalcidoidea	Trichogrammatidae	Trichogramma	sp.	KM569080.1
Hymenoptera	Chalcidoidea	Trichogrammatidae	Trichogramma	sp.	KM569155.1
Hymenoptera	Chalcidoidea	Trichogrammatidae	Trichogramma	sp.	KM569416.1
Hymenoptera	Chalcidoidea	Trichogrammatidae	Trichogramma	sp.	KR365785.1
Hymenoptera	Chalcidoidea	Trichogrammatidae	Trichogramma	sp.	KR365851.1
Hymenoptera	Chalcidoidea	Trichogrammatidae	Trichogramma	sp.	KR366258.1
Hymenoptera	Chalcidoidea	Trichogrammatidae	Trichogramma	sp.	KR366847.1
Hymenoptera	Chalcidoidea	Trichogrammatidae	Trichogramma	sp.	KR366992.1
Hymenoptera	Chalcidoidea	Trichogrammatidae	Trichogramma	sp.	KR367510.1
Hymenoptera	Chalcidoidea	Trichogrammatidae	Trichogramma	sp.	KR367934.1
Hymenoptera	Chalcidoidea	Trichogrammatidae	Trichogramma	sp.	KR368713.1
Hymenoptera	Chalcidoidea	Trichogrammatidae	Trichogramma	sp.	KR369688.1
Hymenoptera	Chalcidoidea	Trichogrammatidae	Trichogramma	sp.	KR370205.1
Hymenoptera	Chalcidoidea	Trichogrammatidae	Trichogramma	sp.	KR370295.1
Hymenoptera	Chalcidoidea	Trichogrammatidae	Trichogramma	sp.	KR371366.1

Hymenoptera	Chalcidoidea	Trichogrammatidae	Trichogramma	sp.	KR371973.1
Hymenoptera	Chalcidoidea	Trichogrammatidae	Trichogramma	sp.	KR374035.1
Hymenoptera	Chalcidoidea	Trichogrammatidae	Trichogramma	sp.	KR374048.1
Hymenoptera	Chalcidoidea	Trichogrammatidae	Trichogramma	sp.	KR375253.1
Hymenoptera	Chalcidoidea	Trichogrammatidae	Trichogramma	sp.	KR375568.1
Hymenoptera	Chalcidoidea	Trichogrammatidae	Trichogramma	sp.	KR783220.1
Hymenoptera	Chalcidoidea	Trichogrammatidae	Trichogramma	sp.	KR783415.1
Hymenoptera	Chalcidoidea	Trichogrammatidae	Trichogramma	sp.	KR786157.1
Hymenoptera	Chalcidoidea	Trichogrammatidae	Trichogramma	sp.	KR787135.1
Hymenoptera	Chalcidoidea	Trichogrammatidae	Trichogramma	sp.	KR787812.1
Hymenoptera	Chalcidoidea	Trichogrammatidae	Trichogramma	sp.	KR788123.1
Hymenoptera	Chalcidoidea	Trichogrammatidae	Trichogramma	sp.	KR788872.1
Hymenoptera	Chalcidoidea	Trichogrammatidae	Trichogramma	sp.	KR790513.1
Hymenoptera	Chalcidoidea	Trichogrammatidae	Trichogramma	sp.	KR794810.1
Hymenoptera	Chalcidoidea	Trichogrammatidae	Trichogramma	sp.	KR798819.1
Hymenoptera	Chalcidoidea	Trichogrammatidae	Trichogramma	sp.	KR806665.1
Hymenoptera	Chalcidoidea	Trichogrammatidae	Trichogramma	sp.	KR806716.1
Hymenoptera	Chalcidoidea	Trichogrammatidae	Trichogramma	sp.	KR808638.1
Hymenoptera	Chalcidoidea	Trichogrammatidae	Trichogramma	sp.	KR875418.1
Hymenoptera	Chalcidoidea	Trichogrammatidae	Trichogramma	sp.	KR880801.1
Hymenoptera	Chalcidoidea	Trichogrammatidae	Trichogramma	sp.	KR884798.1
Hymenoptera	Chalcidoidea	Trichogrammatidae	Trichogramma	sp.	KR886567.1
Hymenoptera	Chalcidoidea	Trichogrammatidae	Trichogramma	sp.	KR892236.1
Hymenoptera	Chalcidoidea	Trichogrammatidae	Trichogramma	sp.	KR894089.1
Hymenoptera	Chalcidoidea	Trichogrammatidae	Trichogramma	sp.	KR897205.1
Hymenoptera	Chalcidoidea	Trichogrammatidae	Trichogramma	sp.	KR897227.1
Hymenoptera	Chalcidoidea	Trichogrammatidae	Trichogramma	sp.	KR897725.1
Hymenoptera	Chalcidoidea	Trichogrammatidae	Trichogramma	sp.	KR899467.1
Hymenoptera	Chalcidoidea	Trichogrammatidae	Trichogramma	sp.	KR900765.1
Hymenoptera	Chalcidoidea	Trichogrammatidae	Trichogramma	sp.	KT614217.1
Hymenoptera	Chalcidoidea	Trichogrammatidae	Trichogramma	sp.	KT615930.1
Hymenoptera	Chalcidoidea	Trichogrammatidae	Trichogramma	sp.	KT617331.1
Hymenoptera	Chalcidoidea	Trichogrammatidae	Trichogramma	sp.	KY934085.1
Hymenoptera	Chalcidoidea	Trichogrammatidae	Trichogramma	sp.	MG374029.1
Hymenoptera	Chalcidoidea	Trichogrammatidae	Trichogramma	sp.	MG375690.1
Hymenoptera	Chalcidoidea	Trichogrammatidae	Trichogramma	sp.	MG376062.1
Hymenoptera	Chalcidoidea	Trichogrammatidae	Trichogramma	sp.	MG377678.1
Hymenoptera	Chalcidoidea	Trichogrammatidae	Trichogramma	sp.	MG378465.1
Hymenoptera	Chalcidoidea	Trichogrammatidae	Trichogramma	sp.	MG379041.1
Hymenoptera	Chalcidoidea	Trichogrammatidae	Trichogramma	sp.	MG379289.1
Hymenoptera	Chalcidoidea	Trichogrammatidae	Trichogramma	sp.	MG380740.1
Hymenoptera	Chalcidoidea	Trichogrammatidae	Trichogramma	sp.	MG381057.1
Hymenoptera	Chalcidoidea	Trichogrammatidae	Trichogramma	sp.	MG381886.1
Hymenoptera	Chalcidoidea	Trichogrammatidae	Trichogramma	sp.	MG382270.1
Hymenoptera	Chalcidoidea	Trichogrammatidae	Trichogramma	sp.	MG497445.1
Hymenoptera	Chalcidoidea	Trichogrammatidae	Trichogramma	sp.	MG497948.1
Hymenoptera	Chalcidoidea	Trichogrammatidae	Trichogramma	sp.	MG499129.1
Hymenoptera	Chalcidoidea	Trichogrammatidae	Trichogramma	sp.	MG501159.1
Hymenoptera	Chalcidoidea	Trichogrammatidae	Trichogramma	sp.	MG501380.1
Hymenoptera	Chalcidoidea	Trichogrammatidae	Trichogramma	sp.	MG501557.1
Hymenoptera	Chalcidoidea	Trichogrammatidae	Trichogramma	sp.	MG501841.1
Hymenoptera	Chalcidoidea	Trichogrammatidae	Trichogramma	sp.	MG501971.1
Hymenoptera	Chalcidoidea	Trichogrammatidae	Trichogramma	sp.	MG502421.1
Hymenoptera	Chalcidoidea	Trichogrammatidae	Trichogramma	sp.	MG504175.1

Hymenoptera	Chalcidoidea	Trichogrammatidae	Trichogramma	sp.	MG506433.1
Hymenoptera	Chalcidoidea	Trichogrammatidae	Trichogramma	sp.	MG506811.1
Hymenoptera	Chalcidoidea	Trichogrammatidae	Trichogramma	sp.	MG510363.1
Hymenoptera	Chalcidoidea	Trichogrammatidae	Trichogramma	sp.	MG510624.1
Hymenoptera	Chalcidoidea	Trichogrammatidae	Trichogramma	sp.	MK611821.1
Hymenoptera	Chalcidoidea	Trichogrammatidae	Trichogramma	sp.	MK611822.1
Hymenoptera	Chalcidoidea	Trichogrammatidae	Trichogrammatidae	sp.	KJ085507.1
Hymenoptera	Chalcidoidea	Trichogrammatidae	Trichogrammatidae	sp.	KJ088077.1
Hymenoptera	Chalcidoidea	Trichogrammatidae	Trichogrammatidae	sp.	KJ089309.1
Hymenoptera	Chalcidoidea	Trichogrammatidae	Trichogrammatidae	sp.	KJ164706.1
Hymenoptera	Chalcidoidea	Trichogrammatidae	Trichogrammatidae	sp.	KJ165511.1
Hymenoptera	Chalcidoidea	Trichogrammatidae	Trichogrammatidae	sp.	KJ166341.1
Hymenoptera	Chalcidoidea	Trichogrammatidae	Trichogrammatidae	sp.	KJ166648.1
Hymenoptera	Chalcidoidea	Trichogrammatidae	Trichogrammatidae	sp.	KJ209465.1
Hymenoptera	Chalcidoidea	Trichogrammatidae	Trichogrammatidae	sp.	KM556285.1
Hymenoptera	Chalcidoidea	Trichogrammatidae	Trichogrammatidae	sp.	KM556381.1
Hymenoptera	Chalcidoidea	Trichogrammatidae	Trichogrammatidae	sp.	KM556388.1
Hymenoptera	Chalcidoidea	Trichogrammatidae	Trichogrammatidae	sp.	KM556502.1
Hymenoptera	Chalcidoidea	Trichogrammatidae	Trichogrammatidae	sp.	KM556586.1
Hymenoptera	Chalcidoidea	Trichogrammatidae	Trichogrammatidae	sp.	KM557299.1
Hymenoptera	Chalcidoidea	Trichogrammatidae	Trichogrammatidae	sp.	KM557616.1
Hymenoptera	Chalcidoidea	Trichogrammatidae	Trichogrammatidae	sp.	KM557821.1
Hymenoptera	Chalcidoidea	Trichogrammatidae	Trichogrammatidae	sp.	KM558505.1
Hymenoptera	Chalcidoidea	Trichogrammatidae	Trichogrammatidae	sp.	KM559034.1
Hymenoptera	Chalcidoidea	Trichogrammatidae	Trichogrammatidae	sp.	KM559108.1
Hymenoptera	Chalcidoidea	Trichogrammatidae	Trichogrammatidae	sp.	KM559119.1
Hymenoptera	Chalcidoidea	Trichogrammatidae	Trichogrammatidae	sp.	KM559206.1
Hymenoptera	Chalcidoidea	Trichogrammatidae	Trichogrammatidae	sp.	KM559767.1
Hymenoptera	Chalcidoidea	Trichogrammatidae	Trichogrammatidae	sp.	KM559768.1
Hymenoptera	Chalcidoidea	Trichogrammatidae	Trichogrammatidae	sp.	KM559776.1
Hymenoptera	Chalcidoidea	Trichogrammatidae	Trichogrammatidae	sp.	KM560057.1
Hymenoptera	Chalcidoidea	Trichogrammatidae	Trichogrammatidae	sp.	KM560115.1
Hymenoptera	Chalcidoidea	Trichogrammatidae	Trichogrammatidae	sp.	KM560313.1
Hymenoptera	Chalcidoidea	Trichogrammatidae	Trichogrammatidae	sp.	KM560726.1
Hymenoptera	Chalcidoidea	Trichogrammatidae	Trichogrammatidae	sp.	KM561411.1
Hymenoptera	Chalcidoidea	Trichogrammatidae	Trichogrammatidae	sp.	KM561493.1
Hymenoptera	Chalcidoidea	Trichogrammatidae	Trichogrammatidae	sp.	KM562836.1
Hymenoptera	Chalcidoidea	Trichogrammatidae	Trichogrammatidae	sp.	KM563130.1
Hymenoptera	Chalcidoidea	Trichogrammatidae	Trichogrammatidae	sp.	KM563286.1
Hymenoptera	Chalcidoidea	Trichogrammatidae	Trichogrammatidae	sp.	KM563615.1
Hymenoptera	Chalcidoidea	Trichogrammatidae	Trichogrammatidae	sp.	KM564103.1
Hymenoptera	Chalcidoidea	Trichogrammatidae	Trichogrammatidae	sp.	KM564740.1
Hymenoptera	Chalcidoidea	Trichogrammatidae	Trichogrammatidae	sp.	KM564841.1
Hymenoptera	Chalcidoidea	Trichogrammatidae	Trichogrammatidae	sp.	KM565094.1
Hymenoptera	Chalcidoidea	Trichogrammatidae	Trichogrammatidae	sp.	KM565160.1
Hymenoptera	Chalcidoidea	Trichogrammatidae	Trichogrammatidae	sp.	KM565466.1
Hymenoptera	Chalcidoidea	Trichogrammatidae	Trichogrammatidae	sp.	KM565524.1
Hymenoptera	Chalcidoidea	Trichogrammatidae	Trichogrammatidae	sp.	KM566035.1
Hymenoptera	Chalcidoidea	Trichogrammatidae	Trichogrammatidae	sp.	KM566043.1
Hymenoptera	Chalcidoidea	Trichogrammatidae	Trichogrammatidae	sp.	KM566185.1
Hymenoptera	Chalcidoidea	Trichogrammatidae	Trichogrammatidae	sp.	KM566230.1
Hymenoptera	Chalcidoidea	Trichogrammatidae	Trichogrammatidae	sp.	KM566284.1
Hymenoptera	Chalcidoidea	Trichogrammatidae	Trichogrammatidae	sp.	KM566309.1
Hymenoptera	Chalcidoidea	Trichogrammatidae	Trichogrammatidae	sp.	KM566624.1

Hymenoptera	Chalcidoidea	Trichogrammatidae	Trichogrammatidae	sp.	KM566850.1
Hymenoptera	Chalcidoidea	Trichogrammatidae	Trichogrammatidae	sp.	KM567214.1
Hymenoptera	Chalcidoidea	Trichogrammatidae	Trichogrammatidae	sp.	KM567884.1
Hymenoptera	Chalcidoidea	Trichogrammatidae	Trichogrammatidae	sp.	KM568138.1
Hymenoptera	Chalcidoidea	Trichogrammatidae	Trichogrammatidae	sp.	KM569583.1
Hymenoptera	Chalcidoidea	Trichogrammatidae	Trichogrammatidae	sp.	KR365127.1
Hymenoptera	Chalcidoidea	Trichogrammatidae	Trichogrammatidae	sp.	KR365158.1
Hymenoptera	Chalcidoidea	Trichogrammatidae	Trichogrammatidae	sp.	KR365792.1
Hymenoptera	Chalcidoidea	Trichogrammatidae	Trichogrammatidae	sp.	KR365864.1
Hymenoptera	Chalcidoidea	Trichogrammatidae	Trichogrammatidae	sp.	KR365867.1
Hymenoptera	Chalcidoidea	Trichogrammatidae	Trichogrammatidae	sp.	KR365954.1
Hymenoptera	Chalcidoidea	Trichogrammatidae	Trichogrammatidae	sp.	KR366116.1
Hymenoptera	Chalcidoidea	Trichogrammatidae	Trichogrammatidae	sp.	KR366625.1
Hymenoptera	Chalcidoidea	Trichogrammatidae	Trichogrammatidae	sp.	KR366810.1
Hymenoptera	Chalcidoidea	Trichogrammatidae	Trichogrammatidae	sp.	KR366825.1
Hymenoptera	Chalcidoidea	Trichogrammatidae	Trichogrammatidae	sp.	KR367098.1
Hymenoptera	Chalcidoidea	Trichogrammatidae	Trichogrammatidae	sp.	KR367801.1
Hymenoptera	Chalcidoidea	Trichogrammatidae	Trichogrammatidae	sp.	KR368199.1
Hymenoptera	Chalcidoidea	Trichogrammatidae	Trichogrammatidae	sp.	KR368948.1
Hymenoptera	Chalcidoidea	Trichogrammatidae	Trichogrammatidae	sp.	KR369006.1
Hymenoptera	Chalcidoidea	Trichogrammatidae	Trichogrammatidae	sp.	KR369974.1
Hymenoptera	Chalcidoidea	Trichogrammatidae	Trichogrammatidae	sp.	KR370358.1
Hymenoptera	Chalcidoidea	Trichogrammatidae	Trichogrammatidae	sp.	KR370568.1
Hymenoptera	Chalcidoidea	Trichogrammatidae	Trichogrammatidae	sp.	KR370674.1
Hymenoptera	Chalcidoidea	Trichogrammatidae	Trichogrammatidae	sp.	KR370815.1
Hymenoptera	Chalcidoidea	Trichogrammatidae	Trichogrammatidae	sp.	KR371253.1
Hymenoptera	Chalcidoidea	Trichogrammatidae	Trichogrammatidae	sp.	KR371545.1
Hymenoptera	Chalcidoidea	Trichogrammatidae	Trichogrammatidae	sp.	KR372266.1
Hymenoptera	Chalcidoidea	Trichogrammatidae	Trichogrammatidae	sp.	KR372268.1
Hymenoptera	Chalcidoidea	Trichogrammatidae	Trichogrammatidae	sp.	KR372412.1
Hymenoptera	Chalcidoidea	Trichogrammatidae	Trichogrammatidae	sp.	KR372822.1
Hymenoptera	Chalcidoidea	Trichogrammatidae	Trichogrammatidae	sp.	KR373261.1
Hymenoptera	Chalcidoidea	Trichogrammatidae	Trichogrammatidae	sp.	KR373299.1
Hymenoptera	Chalcidoidea	Trichogrammatidae	Trichogrammatidae	sp.	KR373382.1
Hymenoptera	Chalcidoidea	Trichogrammatidae	Trichogrammatidae	sp.	KR373546.1
Hymenoptera	Chalcidoidea	Trichogrammatidae	Trichogrammatidae	sp.	KR373796.1
Hymenoptera	Chalcidoidea	Trichogrammatidae	Trichogrammatidae	sp.	KR373996.1
Hymenoptera	Chalcidoidea	Trichogrammatidae	Trichogrammatidae	sp.	KR374099.1
Hymenoptera	Chalcidoidea	Trichogrammatidae	Trichogrammatidae	sp.	KR374591.1
Hymenoptera	Chalcidoidea	Trichogrammatidae	Trichogrammatidae	sp.	KR374870.1
Hymenoptera	Chalcidoidea	Trichogrammatidae	Trichogrammatidae	sp.	KR375070.1
Hymenoptera	Chalcidoidea	Trichogrammatidae	Trichogrammatidae	sp.	KR375105.1
Hymenoptera	Chalcidoidea	Trichogrammatidae	Trichogrammatidae	sp.	KR375134.1
Hymenoptera	Chalcidoidea	Trichogrammatidae	Trichogrammatidae	sp.	KR786906.1
Hymenoptera	Chalcidoidea	Trichogrammatidae	Trichogrammatidae	sp.	KR788468.1
Hymenoptera	Chalcidoidea	Trichogrammatidae	Trichogrammatidae	sp.	KR789015.1
Hymenoptera	Chalcidoidea	Trichogrammatidae	Trichogrammatidae	sp.	KR789712.1
Hymenoptera	Chalcidoidea	Trichogrammatidae	Trichogrammatidae	sp.	KR789759.1
Hymenoptera	Chalcidoidea	Trichogrammatidae	Trichogrammatidae	sp.	KR790611.1
Hymenoptera	Chalcidoidea	Trichogrammatidae	Trichogrammatidae	sp.	KR791984.1
Hymenoptera	Chalcidoidea	Trichogrammatidae	Trichogrammatidae	sp.	KR792521.1
Hymenoptera	Chalcidoidea	Trichogrammatidae	Trichogrammatidae	sp.	KR794079.1
Hymenoptera	Chalcidoidea	Trichogrammatidae	Trichogrammatidae	sp.	KR794517.1
Hymenoptera	Chalcidoidea	Trichogrammatidae	Trichogrammatidae	sp.	KR797508.1

Hymenoptera	Chalcidoidea	Trichogrammatidae	Trichogrammatidae	sp.	KR797825.1
Hymenoptera	Chalcidoidea	Trichogrammatidae	Trichogrammatidae	sp.	KR798089.1
Hymenoptera	Chalcidoidea	Trichogrammatidae	Trichogrammatidae	sp.	KR799851.1
Hymenoptera	Chalcidoidea	Trichogrammatidae	Trichogrammatidae	sp.	KR801568.1
Hymenoptera	Chalcidoidea	Trichogrammatidae	Trichogrammatidae	sp.	KR802394.1
Hymenoptera	Chalcidoidea	Trichogrammatidae	Trichogrammatidae	sp.	KR802758.1
Hymenoptera	Chalcidoidea	Trichogrammatidae	Trichogrammatidae	sp.	KR806181.1
Hymenoptera	Chalcidoidea	Trichogrammatidae	Trichogrammatidae	sp.	KR873999.1
Hymenoptera	Chalcidoidea	Trichogrammatidae	Trichogrammatidae	sp.	KR874609.1
Hymenoptera	Chalcidoidea	Trichogrammatidae	Trichogrammatidae	sp.	KR875952.1
Hymenoptera	Chalcidoidea	Trichogrammatidae	Trichogrammatidae	sp.	KR876102.1
Hymenoptera	Chalcidoidea	Trichogrammatidae	Trichogrammatidae	sp.	KR877676.1
Hymenoptera	Chalcidoidea	Trichogrammatidae	Trichogrammatidae	sp.	KR878973.1
Hymenoptera	Chalcidoidea	Trichogrammatidae	Trichogrammatidae	sp.	KR880091.1
Hymenoptera	Chalcidoidea	Trichogrammatidae	Trichogrammatidae	sp.	KR880272.1
Hymenoptera	Chalcidoidea	Trichogrammatidae	Trichogrammatidae	sp.	KR881809.1
Hymenoptera	Chalcidoidea	Trichogrammatidae	Trichogrammatidae	sp.	KR881963.1
Hymenoptera	Chalcidoidea	Trichogrammatidae	Trichogrammatidae	sp.	KR882595.1
Hymenoptera	Chalcidoidea	Trichogrammatidae	Trichogrammatidae	sp.	KR882744.1
Hymenoptera	Chalcidoidea	Trichogrammatidae	Trichogrammatidae	sp.	KR882991.1
Hymenoptera	Chalcidoidea	Trichogrammatidae	Trichogrammatidae	sp.	KR884089.1
Hymenoptera	Chalcidoidea	Trichogrammatidae	Trichogrammatidae	sp.	KR884267.1
Hymenoptera	Chalcidoidea	Trichogrammatidae	Trichogrammatidae	sp.	KR884726.1
Hymenoptera	Chalcidoidea	Trichogrammatidae	Trichogrammatidae	sp.	KR884901.1
Hymenoptera	Chalcidoidea	Trichogrammatidae	Trichogrammatidae	sp.	KR886296.1
Hymenoptera	Chalcidoidea	Trichogrammatidae	Trichogrammatidae	sp.	KR886700.1
Hymenoptera	Chalcidoidea	Trichogrammatidae	Trichogrammatidae	sp.	KR886869.1
Hymenoptera	Chalcidoidea	Trichogrammatidae	Trichogrammatidae	sp.	KR887161.1
Hymenoptera	Chalcidoidea	Trichogrammatidae	Trichogrammatidae	sp.	KR887521.1
Hymenoptera	Chalcidoidea	Trichogrammatidae	Trichogrammatidae	sp.	KR888870.1
Hymenoptera	Chalcidoidea	Trichogrammatidae	Trichogrammatidae	sp.	KR889598.1
Hymenoptera	Chalcidoidea	Trichogrammatidae	Trichogrammatidae	sp.	KR889971.1
Hymenoptera	Chalcidoidea	Trichogrammatidae	Trichogrammatidae	sp.	KR890510.1
Hymenoptera	Chalcidoidea	Trichogrammatidae	Trichogrammatidae	sp.	KR890825.1
Hymenoptera	Chalcidoidea	Trichogrammatidae	Trichogrammatidae	sp.	KR891774.1
Hymenoptera	Chalcidoidea	Trichogrammatidae	Trichogrammatidae	sp.	KR892114.1
Hymenoptera	Chalcidoidea	Trichogrammatidae	Trichogrammatidae	sp.	KR893694.1
Hymenoptera	Chalcidoidea	Trichogrammatidae	Trichogrammatidae	sp.	KR894230.1
Hymenoptera	Chalcidoidea	Trichogrammatidae	Trichogrammatidae	sp.	KR894714.1
Hymenoptera	Chalcidoidea	Trichogrammatidae	Trichogrammatidae	sp.	KR895396.1
Hymenoptera	Chalcidoidea	Trichogrammatidae	Trichogrammatidae	sp.	KR895996.1
Hymenoptera	Chalcidoidea	Trichogrammatidae	Trichogrammatidae	sp.	KR897501.1
Hymenoptera	Chalcidoidea	Trichogrammatidae	Trichogrammatidae	sp.	KR897774.1
Hymenoptera	Chalcidoidea	Trichogrammatidae	Trichogrammatidae	sp.	KR898051.1
Hymenoptera	Chalcidoidea	Trichogrammatidae	Trichogrammatidae	sp.	KR898894.1
Hymenoptera	Chalcidoidea	Trichogrammatidae	Trichogrammatidae	sp.	KR899548.1
Hymenoptera	Chalcidoidea	Trichogrammatidae	Trichogrammatidae	sp.	KR900428.1
Hymenoptera	Chalcidoidea	Trichogrammatidae	Trichogrammatidae	sp.	KR900863.1
Hymenoptera	Chalcidoidea	Trichogrammatidae	Trichogrammatidae	sp.	KR900875.1
Hymenoptera	Chalcidoidea	Trichogrammatidae	Trichogrammatidae	sp.	KR901055.1
Hymenoptera	Chalcidoidea	Trichogrammatidae	Trichogrammatidae	sp.	KR901481.1
Hymenoptera	Chalcidoidea	Trichogrammatidae	Trichogrammatidae	sp.	KR902104.1
Hymenoptera	Chalcidoidea	Trichogrammatidae	Trichogrammatidae	sp.	KR902109.1
Hymenoptera	Chalcidoidea	Trichogrammatidae	Trichogrammatidae	sp.	KR923885.1

Hymenoptera	Chalcidoidea	Trichogrammatidae	Trichogrammatidae	sp.	KR928355.1
Hymenoptera	Chalcidoidea	Trichogrammatidae	Trichogrammatidae	sp.	KY831015.1
Hymenoptera	Chalcidoidea	Trichogrammatidae	Trichogrammatidae	sp.	KY831044.1
Hymenoptera	Chalcidoidea	Trichogrammatidae	Trichogrammatidae	sp.	KY831132.1
Hymenoptera	Chalcidoidea	Trichogrammatidae	Trichogrammatidae	sp.	KY831635.1
Hymenoptera	Chalcidoidea	Trichogrammatidae	Trichogrammatidae	sp.	KY835361.1
Hymenoptera	Chalcidoidea	Trichogrammatidae	Trichogrammatidae	sp.	KY835695.1
Hymenoptera	Chalcidoidea	Trichogrammatidae	Trichogrammatidae	sp.	KY836097.1
Hymenoptera	Chalcidoidea	Trichogrammatidae	Trichogrammatidae	sp.	KY837521.1
Hymenoptera	Chalcidoidea	Trichogrammatidae	Trichogrammatidae	sp.	KY838358.1
Hymenoptera	Chalcidoidea	Trichogrammatidae	Trichogrammatidae	sp.	KY838875.1
Hymenoptera	Chalcidoidea	Trichogrammatidae	Trichogrammatidae	sp.	KY840109.1
Hymenoptera	Chalcidoidea	Trichogrammatidae	Trichogrammatidae	sp.	KY841443.1
Hymenoptera	Chalcidoidea	Trichogrammatidae	Trichogrammatidae	sp.	KY841743.1
Hymenoptera	Chalcidoidea	Trichogrammatidae	Trichogrammatidae	sp.	KY842613.1
Hymenoptera	Chalcidoidea	Trichogrammatidae	Trichogrammatidae	sp.	KY842896.1
Hymenoptera	Chalcidoidea	Trichogrammatidae	Trichogrammatidae	sp.	KY843143.1
Hymenoptera	Chalcidoidea	Trichogrammatidae	Trichogrammatidae	sp.	KY844347.1
Hymenoptera	Chalcidoidea	Trichogrammatidae	Trichogrammatidae	sp.	KY844518.1
Hymenoptera	Chalcidoidea	Trichogrammatidae	Trichogrammatidae	sp.	KY845406.1
Hymenoptera	Chalcidoidea	Trichogrammatidae	Trichogrammatidae	sp.	KY846226.1
Hymenoptera	Chalcidoidea	Trichogrammatidae	Trichogrammatidae	sp.	KY846975.1
Hymenoptera	Chalcidoidea	Trichogrammatidae	Trichogrammatidae	sp.	MG373889.1
Hymenoptera	Chalcidoidea	Trichogrammatidae	Trichogrammatidae	sp.	MG373997.1
Hymenoptera	Chalcidoidea	Trichogrammatidae	Trichogrammatidae	sp.	MG375491.1
Hymenoptera	Chalcidoidea	Trichogrammatidae	Trichogrammatidae	sp.	MG376674.1
Hymenoptera	Chalcidoidea	Trichogrammatidae	Trichogrammatidae	sp.	MG377918.1
Hymenoptera	Chalcidoidea	Trichogrammatidae	Trichogrammatidae	sp.	MG380162.1
Hymenoptera	Chalcidoidea	Trichogrammatidae	Trichogrammatidae	sp.	MG380200.1
Hymenoptera	Chalcidoidea	Trichogrammatidae	Trichogrammatidae	sp.	MG380532.1
Hymenoptera	Chalcidoidea	Trichogrammatidae	Trichogrammatidae	sp.	MG380911.1
Hymenoptera	Chalcidoidea	Trichogrammatidae	Trichogrammatidae	sp.	MG381446.1
Hymenoptera	Chalcidoidea	Trichogrammatidae	Trichogrammatidae	sp.	MG382543.1
Hymenoptera	Chalcidoidea	Trichogrammatidae	Trichogrammatidae	sp.	MG497061.1
Hymenoptera	Chalcidoidea	Trichogrammatidae	Trichogrammatidae	sp.	MG497355.1
Hymenoptera	Chalcidoidea	Trichogrammatidae	Trichogrammatidae	sp.	MG498534.1
Hymenoptera	Chalcidoidea	Trichogrammatidae	Trichogrammatidae	sp.	MG500174.1
Hymenoptera	Chalcidoidea	Trichogrammatidae	Trichogrammatidae	sp.	MG500421.1
Hymenoptera	Chalcidoidea	Trichogrammatidae	Trichogrammatidae	sp.	MG500525.1
Hymenoptera	Chalcidoidea	Trichogrammatidae	Trichogrammatidae	sp.	MG502743.1
Hymenoptera	Chalcidoidea	Trichogrammatidae	Trichogrammatidae	sp.	MG506142.1
Hymenoptera	Chalcidoidea	Trichogrammatidae	Trichogrammatidae	sp.	MG509363.1
Hymenoptera	Chalcidoidea	Trichogrammatidae	Trichogrammatidae	sp.	MG512241.1
Hymenoptera	Chalcidoidea	Trichogrammatidae	Trichogrammatidae	sp.	MG514624.1
Hymenoptera	Chalcidoidea	Trichogrammatidae	Trichogrammatidae	sp.	MN673489.1
Hymenoptera	Chalcidoidea	Trichogrammatidae	Trichogrammatidae	sp.	MN678822.1
Hymenoptera	Chalcidoidea	Trichogrammatidae	Trichogrammatidae	sp.	MN679022.1
Hymenoptera	Chalcidoidea	Trichogrammatidae	Trichogrammatinae	sp.	KM556716.1
Hymenoptera	Chalcidoidea	Trichogrammatidae	Trichogrammatoidea	armigera	KP994541.1
Hymenoptera	Chalcidoidea	Trichogrammatidae	Trichogrammatoidea	armigera	KP994542.1
Hymenoptera	Chalcidoidea	Trichogrammatidae	Trichogrammatoidea	armigera	MF673622.1
Hymenoptera	Chalcidoidea	Trichogrammatidae	Trichogrammatoidea	armigera	MF673623.1
Hymenoptera	Chalcidoidea	Trichogrammatidae	Trichogrammatoidea	armigera	MF673624.1
Hymenoptera	Chalcidoidea	Trichogrammatidae	Trichogrammatoidea	armigera	MF673625.1

Hymenoptera	Chalcidoidea	Trichogrammatidae	Trichogrammatoidea	armigera	MF673626.1
Hymenoptera	Chalcidoidea	Trichogrammatidae	Trichogrammatoidea	armigera	MF673627.1
Hymenoptera	Chalcidoidea	Trichogrammatidae	Trichogrammatoidea	bactrae	KP142715.1
Hymenoptera	Chalcidoidea	Trichogrammatidae	Trichogrammatoidea	bactrae	KP994547.1
Hymenoptera	Chalcidoidea	Trichogrammatidae	Trichogrammatoidea	robusta	KP233826.1
