# Supplementary material for: Exploration for Asian longhorned beetle parasitoids in Korea using an improved sentinel log trap
Source: Parasite. 2023 Dec 12;30:57. doi: 10.1051/parasite/2023062 (PMC10714676; doi:10.1051/parasite/2023062)
Supplement: Supplementary file 1 — Table S1. Dates and number of materials used in this study. Table S2. List of primers used in this study. Table S3. Information of specimens used for sequencing. Table S4. Species information and GenBank accession number. [file parasite-30-57-s1.zip › Supplementary tables S1-S3.pdf]

Table S1. Dates and number of materials used in this study

Year	Locality	Date	No. of logs				No. of insects			
							ALB		Parasitoid	
			AM	AS	AT	SK	Larvae	Eggs	Unidentified parasitoid	*Spathius ibarakius*
2019	Gapyeong	15. Jul. ~ 27. Jul.			5	10	15	135		n/c
2019	Gapyeong	27. Jul. ~ 22. Aug.	9	6	5*	4	292	62	2	n/c
2019	Gapyeong	22. Aug. ~ 04. Sep.	3	5		7	13	73		n/c
**2019 Total**			**12**	**11**	**10**	**21**	**320**	**270**	**2**	n/c
2020	Gapyeong	21. Jul. ~ 07. Aug.		8		15	12	89		n/c
2020	Gapyeong	7. Aug. ~ 22. Aug.		4		18	74	143		n/c
2020	Gapyeong	22. Aug. ~ 04. Sep.				12	8	54		n/c
**2020 Total**			**0**	**12**	**0**	**45**	**94**	**286**	**0**	n/c
2021	Gapyeong	7. Jul. ~ 28. Jul.			31		n/c	n/c		n/c
2021	Gapyeong	13. Jul. ~ 28. Jul.			40		n/c	n/c		n/c
2021	Gapyeong	16. Jul. ~ 12. Aug.			41		107	213		n/c
2021	Gapyeong	21. Jul. ~ 25. Aug.				48	150	262		n/c
2021	Gapyeong	28. Jul. ~ 25. Aug.			34					
2021	Gapyeong	4. Jul. ~ 31. Aug.			31		223	736		n/c
2021	Gapyeong	11. Aug. ~ 31. Aug.			33					
**2021 Total**			**0**	**0**	**210**	**48**	**480+n/c**	**1211+n/c**	**0**	n/c
2022	Gapyeong	19. Jul. ~ 22. Jul.			19		18	293		n/c
2022	Busan	17. Jul ~ 26. Jul.				16	49	2		21
**2022 Total**			**0**	**0**	**19**	**16**	**67**	**293**	**0**	**21+ n/c**
**TOTAL**			**12**	**23**	**239**	**130**	**961+n/c**	**2,060+n/c**	**2**	**21+ n/c**

*: trap where the parasitoid larvae were found

n/c: no count

Table S2. List of primers used in this study

Gene	Direction	Primer	Sequences (5' – 3')	Reference
**COI**	F	SLEPF	CCTGGTTCTTTRATTGGTAATGATC	Li et al., 2010
	R	LEPR	TAAACTTCTGGATGTCCAAAAA	Hebert et al., 2004
	F	LEPF	ATTCAACCAATCATAAAGATAT	Hebert et al., 2004
	R	LEPR	TAAACTTCTGGATGTCCAAAAA	Hebert et al., 2004
	F	CLepF	ATTCAACCAATCATAAAGATATTGG	Hebert et al., 2004
	R	CLepR	TAAACTTCTGGATGTCCAAAAAATCA	Hebert et al., 2004
	F	HCO	TAAACTTCAGGGTGACCAAAAAATCA	Folmer et al. 1994
	R	LCO	GGTCAACAAATCATAAAGATATTGG	Folmer et al. 1994

Table S3. Information of specimens used for sequencing

Specimen code	Family	Genbank Accession Number	Locality	GPS	Date	Voucher deposit
**S2019**	Chalcidoidea	OQ134171	Jeongmok-ri, Buk-myeon, Gapyeong-gun, Gyeonggi-do, S. Korea	38°00'13.7"N 127°26'19.0"E	2019.07.27–08.22	SNU
**A2**	Torymidae	OQ134174	B-4, Millak-dong, Uijeongbu-si, Gyeonggi-do, Gyeonggi-do, S. Korea	N37°45'19.1" E127°09'14.8"	2018.07.30–08.16	KNAE
**A5**	Eupelmidae	OQ134175	Soribong, Millak-dong, Uijeongbu-si, Gyeonggi-do, S. Korea.	N37°45'1.1" E127°8'32.1"	2018.06.01–06.15	KNAE
**A6**	Eupelmidae	OQ134177	Soribong, Millak-dong, Uijeongbu-si, Gyeonggi-do, S. Korea.	N37°45'1.1" E127°8'32.1"	2018.06.01–06.15	KNAE
**A7**	Eupelmidae	OQ134181	Nat. Arb., Gwangneungsumogwon-ro, Soheul-eup, Pocheon-gun, Gyeonggi-do, S. Korea.	N37°45'32.2" E127°9'42"	2018.07.30–08.16	KNAE
**A9**	Eupelmidae	OQ134368	Loose-flower hornbea, Millak-dong, Uijeongbu-si, Gyeonggi-do, Gyeonggi-do, S. Korea.	N37°44'49.2" E127°8'34.9"	2018.06.29–07.16	KNAE
**A10**	Eupelmidae	OQ134180	Loose-flower hornbea, Millak-dong, Uijeongbu-si, Gyeonggi-do, Gyeonggi-do, S. Korea.	N37°44'49.2" E127°8'34.9"	2018.06.29–07.16	KNAE
**A11**	Eupelmidae	OQ134178	Loose-flower hornbea, Millak-dong, Uijeongbu-si, Gyeonggi-do, Gyeonggi-do, S. Korea.	N37°44'49.2" E127°8'34.9"	2018.06.29–07.16	KNAE
**A12**	Eupelmidae	OQ134179	Loose-flower hornbea, Millak-dong, Uijeongbu-si, Gyeonggi-do, Gyeonggi-do, S. Korea.	N37°44'49.2" E127°8'34.9"	2018.06.29–07.16	KNAE

**B1**	Eulophidae	OQ134269	DMZ Arb., Mandae-ri, Haean-myeon, Yanggu-gun, Gangwon-do, S. Korea.	N38°15'9.3" E128°6'40.6"	2017.08.21–09.05	KNAE
**B2**	Eulophidae	OQ134351	DMZ Arb., Mandae-ri, Haean-myeon, Yanggu-gun, Gangwon-do, S. Korea.	N38°15'9.3" E128°6'40.6"	2017.08.21–09.05	KNAE
**B3**	Eulophidae	OQ134270	DMZ Arb., Mandae-ri, Haean-myeon, Yanggu-gun, Gangwon-do, S. Korea.	N38°15'9.3" E128°6'40.6"	2017.09.19–10.01	KNAE
**B4**	Eulophidae	OQ134273	DMZ Arb., Mandae-ri, Haean-myeon, Yanggu-gun, Gangwon-do, S. Korea.	N38°15'9.3" E128°6'40.6"	2017.09.19–10.01	KNAE
**B5**	Eulophidae	OQ134280	DMZ Arb., Mandae-ri, Haean-myeon, Yanggu-gun, Gangwon-do, S. Korea.	N38°15'9.3" E128°6'40.6"	2018.07.18–08.02	KNAE
**B6**	Eulophidae	OQ134275	DMZ Arb., Mandae-ri, Haean-myeon, Yanggu-gun, Gangwon-do, S. Korea.	N38°15'9.3" E128°6'40.6"	2018.08.13–09.14	KNAE
**C1**	Pteromalidae	OQ134345	Mongolian oak, Jikdong-ri, Soheul-eup, Pocheon-gun, Gyeonggi-do, S. Korea.	N37°45'8.8" E127°9'30.6"	2020.05.29–06.15	KNAE
**D1**	Pteromalidae	OQ134208	Daedeukbong, Munhye-ri, Galmal-eup, Cheorwon-gun, Gangwon-do, S. Korea.	N38°11'49.3" E127°23'35.36"	2020.05.19–06.19	KNAE
**D2**	Pteromalidae	OQ134209	Daedeukbong, Munhye-ri, Galmal-eup, Cheorwon-gun, Gangwon-do, S. Korea.	N38°11'49.3" E127°23'35.36"	2020.05.19–06.19	KNAE
**D3**	Pteromalidae	OQ134190	Daedeukbong, Munhye-ri, Galmal-eup, Cheorwon-gun, Gangwon-do, S. Korea.	N38°11'49.3" E127°23'35.36"	2020.08.31–09.24	KNAE
**D4**	Pteromalidae	OQ134350	Daedeukbong, Munhye-ri, Galmal-eup, Cheorwon-gun, Gangwon-do, S. Korea.	N38°11'49.3" E127°23'35.36"	2020.08.31–09.24	KNAE
**C4**	Pteromalidae	OQ134353	Soribong, Millak-dong, Uijeongbu-si, Gyeonggi-do, S. Korea.	N37°45'2" E127°8'33.5"	2020.07.31–08.14	KNAE
**C5**	Pteromalidae	OQ134251	Soribong, Millak-dong, Uijeongbu-si, Gyeonggi-do, S. Korea.	N37°45'2" E127°8'33.5"	2020.08.31–09.14	KNAE
**C6**	Eupelmidae	OQ134176	Soribong, Millak-dong, Uijeongbu-si, Gyeonggi-do, S. Korea.	N37°45'2" E127°8'33.5"	2020.06.29–07.13	KNAE
**C8**	Eulophidae	OQ134319	Nat. Arb., Gwangneungsumogwon-ro, Soheul-eup, Pocheon-gun, Gyeonggi-do, S. Korea.	N37°45'32.6" E127°9.4'1.9"	2020.05.29–06.15	KNAE
**C9**	Eulophidae	OQ134235	Observatory, Igok-ri, Soheul-eup, Pocheon-gun, Gyeonggi-do, S. Korea.	N37°45'19.6" E127°9'1.6"	2020.07.13–07.31	KNAE
**C10**	Eurytomidae	OQ134358	Observatory, Igok-ri, Soheul-eup, Pocheon-gun, Gyeonggi-do, S. Korea.	N37°45'19.6" E127°9'1.6"	2020.05.15–05.29	KNAE
**C11**	Pteromalidae	OQ134376	Observatory, Igok-ri, Soheul-eup, Pocheon-gun, Gyeonggi-do, S. Korea.	N37°45'19.6" E127°9'1.6"	2020.05.15–05.29	KNAE
**C12**	Pteromalidae	OQ134265	Observatory, Igok-ri, Soheul-eup, Pocheon-gun, Gyeonggi-do, S. Korea.	N37°45'19.6" E127°9'1.6"	2020.05.15–05.29	KNAE

**C13**	Pteromalidae	OQ134366	Observatory, Igok-ri, Soheul-eup, Pocheon-gun, Gyeonggi-do, S. Korea.	N37°45'19.6" E127°9'1.6"	2020.08.14–08.31	KNAE
**C14**	Pteromalidae	OQ134240	Observatory, Igok-ri, Soheul-eup, Pocheon-gun, Gyeonggi-do, S. Korea.	N37°45'19.6" E127°9'1.6"	2020.05.29–06.15	KNAE
**c16**	Pteromalidae	OQ134188	Mongolian oak, Jikdong-ri, Soheul-eup, Pocheon-gun, Gyeonggi-do, S. Korea.	N37°45'8.8" E127°9'30.6"	2020.06.29–07.13	KNAE
**D6**	Pteromalidae	OQ134298	Daedeukbong, Munhye-ri, Galmal-eup, Cheorwon-gun, Gangwon-do, S. Korea.	N38°11'49.3" E127°23'35.36"	2020.09.24–10.27	KNAE
**D7**	Pteromalidae	OQ134257	Daedeukbong, Munhye-ri, Galmal-eup, Cheorwon-gun, Gangwon-do, S. Korea.	N38°11'49.3" E127°23'35.36"	2020.07.14–08.31	KNAE
**D12**	Pteromalidae	OQ134300	Daedeukbong, Munhye-ri, Galmal-eup, Cheorwon-gun, Gangwon-do, S. Korea.	N38°11'49.3" E127°23'35.36"	2020.07.14–08.31	KNAE
**D13**	Pteromalidae	OQ134369	Mt. Baekjeok, Guun-ri, Sangseo-myeon, Hwacheon-gun, Gangwon-do, S. Korea.	N38°8'33.8" E127°36'23.33"	2020.08.31–09.24	KNAE
**D17**	Pteromalidae	OQ134324	Mt. Baekjeok, Guun-ri, Sangseo-myeon, Hwacheon-gun, Gangwon-do, S. Korea.	N38°8'33.8" E127°36'23.33"	2020.08.31–09.24	KNAE
**D19**	Pteromalidae	OQ134374	Mt. Baekjeok, Guun-ri, Sangseo-myeon, Hwacheon-gun, Gangwon-do, S. Korea.	N38°8'33.8" E127°36'23.33"	2020.08.31–09.24	KNAE
**D20**	Pteromalidae	OQ134331	Mt. Baekjeok, Guun-ri, Sangseo-myeon, Hwacheon-gun, Gangwon-do, S. Korea.	N38°8'33.8" E127°36'23.33"	2020.08.31–09.24	KNAE
**D21**	Pteromalidae	OQ134325	Mt. Baekjeok, Guun-ri, Sangseo-myeon, Hwacheon-gun, Gangwon-do, S. Korea.	N38°8'33.8" E127°36'23.33"	2020.08.31–09.24	KNAE
**D22**	Pteromalidae	OQ134370	Mt. Baekjeok, Guun-ri, Sangseo-myeon, Hwacheon-gun, Gangwon-do, S. Korea.	N38°8'33.8" E127°36'23.33"	2020.06.19–07.14	KNAE
**D23**	Pteromalidae	OQ134258	Mt. Baekjeok, Guun-ri, Sangseo-myeon, Hwacheon-gun, Gangwon-do, S. Korea.	N38°8'33.8" E127°36'23.33"	2020.06.19–07.14	KNAE
**D24**	Pteromalidae	OQ134371	Mt. Baekjeok, Guun-ri, Sangseo-myeon, Hwacheon-gun, Gangwon-do, S. Korea.	N38°8'33.8" E127°36'23.33"	2020.06.19–07.14	KNAE
**D26**	Pteromalidae	OQ134320	Mt. Baekjeok, Guun-ri, Sangseo-myeon, Hwacheon-gun, Gangwon-do, S. Korea.	N38°8'33.8" E127°36'23.33"	2020.07.14–08.31	KNAE
**D27**	Pteromalidae	OQ134191	Mt. Baekjeok, Guun-ri, Sangseo-myeon, Hwacheon-gun, Gangwon-do, S. Korea.	N38°8'33.8" E127°36'23.33"	2020.07.14–08.31	KNAE
**D29**	Pteromalidae	OQ134337	Mt. Baekjeok, Guun-ri, Sangseo-myeon, Hwacheon-gun, Gangwon-do, S. Korea.	N38°8'33.8" E127°36'23.33"	2020.07.14–08.31	KNAE
**D30**	Pteromalidae	OQ134359	Mt. Baekjeok, Guun-ri, Sangseo-myeon, Hwacheon-gun, Gangwon-do, S. Korea.	N38°8'33.8" E127°36'23.33"	2020.07.14–08.31	KNAE
**D34**	Pteromalidae	OQ134339	Mt. Baekjeok, Guun-ri, Sangseo-myeon, Hwacheon-gun, Gangwon-do, S. Korea.	N38°8'33.8" E127°36'23.33"	2020.07.14–08.31	KNAE

**D36**	Pteromalidae	OQ134286	Mt. Baekjeok, Guun-ri, Sangseo-myeon, Hwacheon-gun, Gangwon-do, S. Korea.	N38°8'33.8" E127°36'23.33"	2020.07.14–08.31	KNAE
**D37**	Pteromalidae	OQ134380	Mt. Baekjeok, Guun-ri, Sangseo-myeon, Hwacheon-gun, Gangwon-do, S. Korea.	N38°8'33.8" E127°36'23.33"	2020.07.14–08.31	KNAE
**D38**	Pteromalidae	OQ134294	Mt. Baekjeok, Guun-ri, Sangseo-myeon, Hwacheon-gun, Gangwon-do, S. Korea.	N38°8'33.8" E127°36'23.33"	2020.07.14–08.31	KNAE
**D39**	Pteromalidae	OQ134360	Mt. Baekjeok, Guun-ri, Sangseo-myeon, Hwacheon-gun, Gangwon-do, S. Korea.	N38°8'33.8" E127°36'23.33"	2020.07.14–08.31	KNAE
**D41**	Pteromalidae	OQ134211	Hyangnobong, Jinbu-ri, Ganseong-eup, Goseong-gun, Gangwon-do, S. Korea.	N38°15'55.99" E128°20'46.86"	2020.05.18–06.22	KNAE
**D42**	Pteromalidae	OQ134327	Hyangnobong, Jinbu-ri, Ganseong-eup, Goseong-gun, Gangwon-do, S. Korea.	N38°15'55.99" E128°20'46.86"	2020.05.18–06.22	KNAE
**D43**	Pteromalidae	OQ134212	Hyangnobong, Jinbu-ri, Ganseong-eup, Goseong-gun, Gangwon-do, S. Korea.	N38°15'55.99" E128°20'46.86"	2020.05.18–06.22	KNAE
**D44**	Pteromalidae	OQ134364	Hyangnobong, Jinbu-ri, Ganseong-eup, Goseong-gun, Gangwon-do, S. Korea.	N38°15'55.99" E128°20'46.86"	2020.05.18–06.22	KNAE
**D45**	Pteromalidae	OQ134237	Hyangnobong, Jinbu-ri, Ganseong-eup, Goseong-gun, Gangwon-do, S. Korea.	N38°15'55.99" E128°20'46.86"	2020.05.18–06.22	KNAE
**D48**	Pteromalidae	OQ134332	Hyangnobong, Jinbu-ri, Ganseong-eup, Goseong-gun, Gangwon-do, S. Korea.	N38°15'55.99" E128°20'46.86"	2020.07.14–08.31	KNAE
**D49**	Pteromalidae	OQ134247	Hyangnobong, Jinbu-ri, Ganseong-eup, Goseong-gun, Gangwon-do, S. Korea.	N38°15'55.99" E128°20'46.86"	2020.07.14–08.31	KNAE
**D50**	Pteromalidae	OQ134308	Hyangnobong, Jinbu-ri, Ganseong-eup, Goseong-gun, Gangwon-do, S. Korea.	N38°15'55.99" E128°20'46.86"	2020.07.14–08.31	KNAE
**D51**	Pteromalidae	OQ134248	Hyangnobong, Jinbu-ri, Ganseong-eup, Goseong-gun, Gangwon-do, S. Korea.	N38°15'55.99" E128°20'46.86"	2020.07.14–08.31	KNAE
**D52**	Pteromalidae	OQ134215	Hyangnobong, Jinbu-ri, Ganseong-eup, Goseong-gun, Gangwon-do, S. Korea.	N38°15'55.99" E128°20'46.86"	2020.07.14–08.31	KNAE
**D53**	Pteromalidae	OQ134243	Hyangnobong, Jinbu-ri, Ganseong-eup, Goseong-gun, Gangwon-do, S. Korea.	N38°15'55.99" E128°20'46.86"	2020.07.14–08.31	KNAE
**D54**	Pteromalidae	OQ134219	Hyangnobong, Jinbu-ri, Ganseong-eup, Goseong-gun, Gangwon-do, S. Korea.	N38°15'55.99" E128°20'46.86"	2020.07.14–08.31	KNAE
**D55**	Pteromalidae	OQ134314	Hyangnobong, Jinbu-ri, Ganseong-eup, Goseong-gun, Gangwon-do, S. Korea.	N38°15'55.99" E128°20'46.86"	2020.06.22–07.14	KNAE
**D56**	Pteromalidae	OQ134217	Hyangnobong, Jinbu-ri, Ganseong-eup, Goseong-gun, Gangwon-do, S. Korea.	N38°15'55.99" E128°20'46.86"	2020.06.18–07.14	KNAE
**D57**	Pteromalidae	OQ134266	Mt. Gaemyeong, Seokhyeon-ri, Jangheung-myeon, Yangju-gun, Gyeonggi-do, S. Korea.	N37°45'2.51" E126°56'24.48"	2020.06.18–07.14	KNAE

**D58**	Pteromalidae	OQ134362	Mt. Gaemyeong, Seokhyeon-ri, Jangheung-myeon, Yangju-gun, Gyeonggi-do, S. Korea.	N37°45'2.51" E126°56'24.48"	2020.06.18–07.14	KNAE
**D59**	Pteromalidae	OQ134267	Mt. Gaemyeong, Seokhyeon-ri, Jangheung-myeon, Yangju-gun, Gyeonggi-do, S. Korea.	N37°45'2.51" E126°56'24.48"	2020.06.18–07.14	KNAE
**D62**	Pteromalidae	OQ134367	Mt. Goseong, Eocheon-ri, Ganseong-eup, Goseong-gun, Gyeonggi-do, S. Korea.	N38°21'14.48" E128°26'37.06"	2020.05.18–06.22	KNAE
**D63**	Pteromalidae	OQ134214	Mt. Goseong, Eocheon-ri, Ganseong-eup, Goseong-gun, Gyeonggi-do, S. Korea.	N38°21'14.48" E128°26'37.06"	2020.05.18–06.22	KNAE
**D64**	Pteromalidae	OQ134218	Mt. Goseong, Eocheon-ri, Ganseong-eup, Goseong-gun, Gyeonggi-do, S. Korea.	N38°21'14.48" E128°26'37.06"	2020.05.18–06.22	KNAE
**D65**	Pteromalidae	OQ134305	Mt. Baekjeok, Guun-ri, Sangseo-myeon, Hwacheon-gun, Gangwon-do, S. Korea.	N38°8'33.8" E127°36'23.33"	2020.06.22–07.14	KNAE
**D66**	Pteromalidae	OQ134338	Mt. Baekjeok, Guun-ri, Sangseo-myeon, Hwacheon-gun, Gangwon-do, S. Korea.	N38°8'33.8" E127°36'23.33"	2020.06.22–07.14	KNAE
**D67**	Pteromalidae	OQ134276	Mt. Baekjeok, Guun-ri, Sangseo-myeon, Hwacheon-gun, Gangwon-do, S. Korea.	N38°8'33.8" E127°36'23.33"	2020.06.22–07.14	KNAE
**D68**	Pteromalidae	OQ134299	Mt. Baekjeok, Guun-ri, Sangseo-myeon, Hwacheon-gun, Gangwon-do, S. Korea.	N38°8'33.8" E127°36'23.33"	2020.06.22–07.14	KNAE
**D70**	Pteromalidae	OQ134322	Mt. Baekjeok, Guun-ri, Sangseo-myeon, Hwacheon-gun, Gangwon-do, S. Korea.	N38°8'33.8" E127°36'23.33"	2020.07.14–08.31	KNAE
**D71**	Pteromalidae	OQ134306	Mt. Baekjeok, Guun-ri, Sangseo-myeon, Hwacheon-gun, Gangwon-do, S. Korea.	N38°8'33.8" E127°36'23.33"	2020.07.14–08.31	KNAE
**D72**	Pteromalidae	OQ134361	Mt. Baekjeok, Guun-ri, Sangseo-myeon, Hwacheon-gun, Gangwon-do, S. Korea.	N38°8'33.8" E127°36'23.33"	2020.07.14–08.31	KNAE
**D73**	Pteromalidae	OQ134301	Mt. Baekjeok, Guun-ri, Sangseo-myeon, Hwacheon-gun, Gangwon-do, S. Korea.	N38°8'33.8" E127°36'23.33"	2020.07.14–08.31	KNAE
**D74**	Pteromalidae	OQ134323	Mt. Baekjeok, Guun-ri, Sangseo-myeon, Hwacheon-gun, Gangwon-do, S. Korea.	N38°8'33.8" E127°36'23.33"	2020.07.14–08.31	KNAE
**D75**	Pteromalidae	OQ134354	Mt. Baekjeok, Guun-ri, Sangseo-myeon, Hwacheon-gun, Gangwon-do, S. Korea.	N38°8'33.8" E127°36'23.33"	2020.09.24–10.27	KNAE
**D76**	Pteromalidae	OQ134197	Mt. Baekjeok, Guun-ri, Sangseo-myeon, Hwacheon-gun, Gangwon-do, S. Korea.	N38°8'33.8" E127°36'23.33"	2020.09.24–10.27	KNAE
**D77**	Pteromalidae	OQ134182	Mt. Baekjeok, Guun-ri, Sangseo-myeon, Hwacheon-gun, Gangwon-do, S. Korea.	N38°8'33.8" E127°36'23.33"	2020.09.24–10.27	KNAE
**D78**	Pteromalidae	OQ134340	Mt. Baekjeok, Guun-ri, Sangseo-myeon, Hwacheon-gun, Gangwon-do, S. Korea.	N38°8'33.8" E127°36'23.33"	2020.09.24–10.27	KNAE
**D79**	Pteromalidae	OQ134326	Mt. Baekjeok, Guun-ri, Sangseo-myeon, Hwacheon-gun, Gangwon-do, S. Korea.	N38°8'33.8" E127°36'23.33"	2020.09.24–10.27	KNAE

**D81**	Pteromalidae	OQ134262	Mt. Baekjeok, Guun-ri, Sangseo-myeon, Hwacheon-gun, Gangwon-do, S. Korea.	N38°8'33.8" E127°36'23.33"	2020.09.24–10.27	KNAE
**D82**	Pteromalidae	OQ134201	Mt. Baekjeok, Guun-ri, Sangseo-myeon, Hwacheon-gun, Gangwon-do, S. Korea.	N38°8'33.8" E127°36'23.33"	2020.09.24–10.27	KNAE
**D83**	Pteromalidae	OQ134363	Mt. Baekjeok, Guun-ri, Sangseo-myeon, Hwacheon-gun, Gangwon-do, S. Korea.	N38°8'33.8" E127°36'23.33"	2020.09.24–10.27	KNAE
**D86**	Pteromalidae	OQ134172	Mt. Toemo, Naega-myeon, Ganghwa-gun, Incheon, S. Korea.	N37°42'56.31" E126°24'52.43"	2020.08.31–09.24	KNAE
**D87**	Pteromalidae	OQ134303	Mt. Toemo, Naega-myeon, Ganghwa-gun, Incheon, S. Korea.	N37°42'56.31" E126°24'52.43"	2020.08.31–09.24	KNAE
**D88**	Pteromalidae	OQ134352	Mt. Toemo, Naega-myeon, Ganghwa-gun, Incheon, S. Korea.	N37°42'56.31" E126°24'52.43"	2020.08.31–09.24	KNAE
**D90**	Pteromalidae	OQ134348	Mt. Papyeong, Beobwon-eup, Paju-si, Gyeonggi-do, S. Korea.	N37°54'36.7" E126°52'58.27"	2020.05.19–06.19	KNAE
**D92**	Pteromalidae	OQ134346	Mt. Papyeong, Beobwon-eup, Paju-si, Gyeonggi-do, S. Korea.	N37°54'36.7" E126°52'58.27"	2020.05.19–06.19	KNAE
**D93**	Pteromalidae	OQ134236	Mt. Papyeong, Beobwon-eup, Paju-si, Gyeonggi-do, S. Korea.	N37°54'36.7" E126°52'58.27"	2020.05.19–06.19	KNAE
**D95**	Pteromalidae	OQ134263	Mt. Papyeong, Beobwon-eup, Paju-si, Gyeonggi-do, S. Korea.	N37°54'36.7" E126°52'58.27"	2020.07.14–08.31	KNAE
**D96**	Pteromalidae	OQ134312	Mt. Papyeong, Beobwon-eup, Paju-si, Gyeonggi-do, S. Korea.	N37°54'36.7" E126°52'58.27"	2020.07.14–08.31	KNAE
**D97**	Pteromalidae	OQ134229	Dutayeon, Satae-ri, Dong-myeon, Yanggu-gun, Gangwon-do, S. Korea.	N38°23'13.89" E127°58'28.58"	2020.09.24–10.27	KNAE
**D99**	Pteromalidae	OQ134241	Dutayeon, Satae-ri, Dong-myeon, Yanggu-gun, Gangwon-do, S. Korea.	N38°23'13.89" E127°58'28.58"	2020.09.24–10.27	KNAE
**D100**	Pteromalidae	OQ134318	Dutayeon, Satae-ri, Dong-myeon, Yanggu-gun, Gangwon-do, S. Korea.	N38°23'13.89" E127°58'28.58"	2020.08.31–09.24	KNAE
**D101**	Pteromalidae	OQ134194	Dutayeon, Satae-ri, Dong-myeon, Yanggu-gun, Gangwon-do, S. Korea.	N38°23'13.89" E127°58'28.58"	2020.08.31–09.24	KNAE
**D102**	Pteromalidae	OQ134347	Dutayeon, Satae-ri, Dong-myeon, Yanggu-gun, Gangwon-do, S. Korea.	N38°23'13.89" E127°58'28.58"	2020.08.31–09.24	KNAE
**D103**	Pteromalidae	OQ134239	Dutayeon, Satae-ri, Dong-myeon, Yanggu-gun, Gangwon-do, S. Korea.	N38°23'13.89" E127°58'28.58"	2020.08.31–09.24	KNAE
**D104**	Pteromalidae	OQ134227	Dutayeon, Satae-ri, Dong-myeon, Yanggu-gun, Gangwon-do, S. Korea.	N38°23'13.89" E127°58'28.58"	2020.07.14–08.31	KNAE
**D105**	Pteromalidae	OQ134228	Dutayeon, Satae-ri, Dong-myeon, Yanggu-gun, Gangwon-do, S. Korea.	N38°23'13.89" E127°58'28.58"	2020.07.14–08.31	KNAE

**D106**	Pteromalidae	OQ134261	Dutayeon, Satae-ri, Dong-myeon, Yanggu-gun, Gangwon-do, S. Korea.	N38°23'13.89" E127°58'28.58"	2020.07.14–08.31	KNAE
**E1**	Pteromalidae	OQ134375	Mt. Gariwang, Hoedong-ri, Jeongseon-eup, Jeongseon-gun, Gangwon-do, S. Korea.	N37°25'28.65" E128°31'43.86"	2020.07.01–08.18	KNAE
**E2**	Pteromalidae	OQ134317	Mt. Gariwang, Hoedong-ri, Jeongseon-eup, Jeongseon-gun, Gangwon-do, S. Korea.	N37°25'28.65" E128°31'43.86"	2020.07.01–08.18	KNAE
**E3**	Pteromalidae	OQ134302	Mt. Hanseok, Deokjeok-ri, Inje-eup, Inje-gun, Gangwon-do, S. Korea.	N38°3'51.37" E128°17'14.7"	2020.06.02–07.01	KNAE
**E4**	Pteromalidae	OQ134315	Mt. Hanseok, Deokjeok-ri, Inje-eup, Inje-gun, Gangwon-do, S. Korea.	N38°3'51.37" E128°17'14.7"	2020.06.02–07.01	KNAE
**E5**	Pteromalidae	OQ134189	Mt. Hanseok, Deokjeok-ri, Inje-eup, Inje-gun, Gangwon-do, S. Korea.	N38°3'51.37" E128°17'14.7"	2020.07.01–08.18	KNAE
**E7**	Pteromalidae	OQ134316	Mt. Hanseok, Deokjeok-ri, Inje-eup, Inje-gun, Gangwon-do, S. Korea.	N38°3'51.37" E128°17'14.7"	2020.07.01–08.18	KNAE
**E9**	Pteromalidae	OQ134290	DMZ Arb., Mandae-ri, Haean-myeon, Yanggu-gun, Gangwon-do, S. Korea.	N38°15'9.3" E128°6'40.06"	2019.08.15–080.3	KNAE
**E10**	Pteromalidae	OQ134381	DMZ Arb., Mandae-ri, Haean-myeon, Yanggu-gun, Gangwon-do, S. Korea.	N38°15'9.3" E128°6'40.06"	2019.08.15–08.03	KNAE
**E11**	Pteromalidae	OQ134297	DMZ Arb., Mandae-ri, Haean-myeon, Yanggu-gun, Gangwon-do, S. Korea.	N38°15'9.3" E128°6'40.06"	2019.08.15–08.03	KNAE
**E12**	Pteromalidae	OQ134222	DMZ Arb., Mandae-ri, Haean-myeon, Yanggu-gun, Gangwon-do, S. Korea.	N38°15'9.3" E128°6'40.06"	2019.08.15–08.03	KNAE
**E14**	Pteromalidae	OQ134307	DMZ Arb., Mandae-ri, Haean-myeon, Yanggu-gun, Gangwon-do, S. Korea.	N38°15'9.3" E128°6'40.06"	2019.09.25	KNAE
**E15**	Pteromalidae	OQ134382	DMZ Arb., Mandae-ri, Haean-myeon, Yanggu-gun, Gangwon-do, S. Korea.	N38°15'9.3" E128°6'40.06"	2019.09.25	KNAE
**E16**	Pteromalidae	OQ134383	DMZ Arb., Mandae-ri, Haean-myeon, Yanggu-gun, Gangwon-do, S. Korea.	N38°15'9.3" E128°6'40.06"	2019.09.25	KNAE
**E17**	Pteromalidae	OQ134246	DMZ Arb., Mandae-ri, Haean-myeon, Yanggu-gun, Gangwon-do, S. Korea.	N38°15'9.3" E128°6'40.06"	2019.08.14	KNAE
**E21**	Pteromalidae	OQ134372	DMZ Arb., Mandae-ri, Haean-myeon, Yanggu-gun, Gangwon-do, S. Korea.	N38°15'9.3" E128°6'40.06"	2019.09.26	KNAE
**E22**	Pteromalidae	OQ134198	DMZ Arb., Mandae-ri, Haean-myeon, Yanggu-gun, Gangwon-do, S. Korea.	N38°15'9.3" E128°6'40.06"	2019.09.26	KNAE
**E23**	Pteromalidae	OQ134268	Baekbongnyeong, Namyang-ri, Okgye-myeon, Gangneung-si, S. Korea.	N37°32'32.74" E128°57'52.37"	2020.06.02–07.01	KNAE
**E24**	Pteromalidae	OQ134329	Baekbongnyeong, Namyang-ri, Okgye-myeon, Gangneung-si, S. Korea.	N37°32'32.74" E128°57'52.37"	2020.06.02–07.01	KNAE

**E25**	Pteromalidae	OQ134220	Baekbongnyeong, Namyang-ri, Okgye-myeon, Gangneung-si, S. Korea.	N37°32'32.74" E128°57'52.37"	2020.06.02–07.01	KNAE
**E26**	Pteromalidae	OQ134282	Baekbongnyeong, Namyang-ri, Okgye-myeon, Gangneung-si, S. Korea.	N37°32'32.74" E128°57'52.37"	2020.06.02–07.01	KNAE
**E28**	Pteromalidae	OQ134334	Baekbongnyeong, Namyang-ri, Okgye-myeon, Gangneung-si, S. Korea.	N37°32'32.74" E128°57'52.37"	2020.06.02–07.01	KNAE
**E29**	Pteromalidae	OQ134281	Mt. Bangtae, Gwangwon-ri, Nae-myeon, Hongcheon-gun, Gangwon-do, S. Korea.	N37°52'18.02" E128°25'38.64"	2020.07.01–08.18	KNAE
**E30**	Pteromalidae	OQ134213	Mt. Bangtae, Gwangwon-ri, Nae-myeon, Hongcheon-gun, Gangwon-do, S. Korea.	N37°52'18.02" E128°25'38.64"	2020.07.01–08.18	KNAE
**F81**	Eulophidae	OQ134278	Dutayeon, Bangsan-myeon, Yanggu-gun, Gangwon-do, S. Korea	N38°13'7330" E127°508'78"	2019.08.09–09.06	KNAE
**F82**	Eulophidae	OQ134277	Dutayeon, Bangsan-myeon, Yanggu-gun, Gangwon-do, S. Korea	N38°13'7330" E127°508'78"	2019.08.09–09.06	KNAE
**F83**	Eulophidae	OQ134271	Dutayeon, Bangsan-myeon, Yanggu-gun, Gangwon-do, S. Korea	N38°13'7330" E127°508'78"	2019.09.06–10.02	KNAE
**F85**	Eulophidae	OQ134274	Soribong, Millak-dong, Uijeongbu-si, Gyeonggi-do, S. Korea.	N37°45'1.6" E127°8'34.9"	2016.04.29–05.15	KNAE
**F87**	Eulophidae	OQ134272	Mt. Goseong, Eocheon-ri, Ganseong-eup, Goseong-gun, Gyeonggi-do, S. Korea.	N38°21'2540" E128°26'2.9"	2019.09.06–10.02	KNAE
**F88**	Eulophidae	OQ134279	Mt. Goseong, Eocheon-ri, Ganseong-eup, Goseong-gun, Gyeonggi-do, S. Korea.	N38°21'2540" E128°26'2.9"	2019.09.06–10.02	KNAE
**F2**	Pteromalidae	OQ134287	Mt. Duta, Yupyeong-ri, Cheongil-myeon, Hoengseong-gun, Gangwon-do, S. Korea.	N37°34'17.85" E128°9'7.2"	2020.08.18–09.15	KNAE
**F3**	Pteromalidae	OQ134202	Mt. Duta, Yupyeong-ri, Cheongil-myeon, Hoengseong-gun, Gangwon-do, S. Korea.	N37°34'17.85" E128°9'7.2"	2020.07.01–08.18	KNAE
**F4**	Pteromalidae	OQ134204	Mt. Duta, Yupyeong-ri, Cheongil-myeon, Hoengseong-gun, Gangwon-do, S. Korea.	N37°34'17.85" E128°9'7.2"	2020.07.01–08.18	KNAE
**F5**	Pteromalidae	OQ134288	Mt. Duta, Yupyeong-ri, Cheongil-myeon, Hoengseong-gun, Gangwon-do, S. Korea.	N37°34'17.85" E128°9'7.2"	2020.07.01–08.18	KNAE
**F6**	Pteromalidae	OQ134343	Mt. Duta, Yupyeong-ri, Cheongil-myeon, Hoengseong-gun, Gangwon-do, S. Korea.	N37°34'17.85" E128°9'7.2"	2020.07.01–08.18	KNAE
**F7**	Pteromalidae	OQ134252	Mt. Duta, Yupyeong-ri, Cheongil-myeon, Hoengseong-gun, Gangwon-do, S. Korea.	N37°34'17.85" E128°9'7.2"	2020.09.15–10.19	KNAE
**F8**	Pteromalidae	OQ134295	Mt. Duta, Yupyeong-ri, Cheongil-myeon, Hoengseong-gun, Gangwon-do, S. Korea.	N37°34'17.85" E128°9'7.2"	2020.09.15–10.19	KNAE
**F9**	Pteromalidae	OQ134296	Mt. Duta, Yupyeong-ri, Cheongil-myeon, Hoengseong-gun, Gangwon-do, S. Korea.	N37°34'17.85" E128°9'7.2"	2020.09.15–10.19	KNAE

**F10**	Pteromalidae	OQ134206	Mt. Duta, Yupyeong-ri, Cheongil-myeon, Hoengseong-gun, Gangwon-do, S. Korea.	N37°34'17.85" E128°9'7.2"	2020.09.15–10.19	KNAE
**F11**	Pteromalidae	OQ134259	Mt. Duta, Yupyeong-ri, Cheongil-myeon, Hoengseong-gun, Gangwon-do, S. Korea.	N37°34'17.85" E128°9'7.2"	2020.06.02–07.01	KNAE
**F12**	Pteromalidae	OQ134200	Mt. Cheongtae, Yupo-ri, Bongpyeong-myeon, Pyeongchang-gun, Gangwon-do, S. Korea.	N37°31'58.03" E128°18'26.79"	2020.06.02–07.01	KNAE
**F14**	Pteromalidae	OQ134249	Mt. Cheongtae, Yupo-ri, Bongpyeong-myeon, Pyeongchang-gun, Gangwon-do, S. Korea.	N37°31'58.03" E128°18'26.79"	2020.06.02–07.01	KNAE
**F15**	Pteromalidae	OQ134365	Mt. Cheongtae, Yupo-ri, Bongpyeong-myeon, Pyeongchang-gun, Gangwon-do, S. Korea.	N37°31'58.03" E128°18'26.79"	2020.07.01–08.18	KNAE
**F16**	Pteromalidae	OQ134341	Mt. Cheongtae, Yupo-ri, Bongpyeong-myeon, Pyeongchang-gun, Gangwon-do, S. Korea.	N37°31'58.03" E128°18'26.79"	2020.07.01–08.18	KNAE
**F17**	Pteromalidae	OQ134289	Mt. Cheongtae, Yupo-ri, Bongpyeong-myeon, Pyeongchang-gun, Gangwon-do, S. Korea.	N37°31'58.03" E128°18'26.79"	2020.07.01–08.18	KNAE
**F18**	Pteromalidae	OQ134199	Baekbongnyeong, Imgye-myeon, Jeongseon-gun, Gangwon-do, S. Korea.	N37°32'32.74" E128°57'52.37"	2020.09.15–10.19	KNAE
**F20**	Pteromalidae	OQ134205	Baekbongnyeong, Imgye-myeon, Jeongseon-gun, Gangwon-do, S. Korea.	N37°32'32.74" E128°57'52.37"	2020.09.15–10.19	KNAE
**F21**	Pteromalidae	OQ134309	Baekbongnyeong, Imgye-myeon, Jeongseon-gun, Gangwon-do, S. Korea.	N37°32'32.74" E128°57'52.37"	2020.09.15–10.19	KNAE
**F23**	Pteromalidae	OQ134232	Baekbongnyeong, Imgye-myeon, Jeongseon-gun, Gangwon-do, S. Korea.	N37°32'32.74" E128°57'52.37"	2020.08.18–09.15	KNAE
**F24**	Pteromalidae	OQ134183	Baekbongnyeong, Imgye-myeon, Jeongseon-gun, Gangwon-do, S. Korea.	N37°32'32.74" E128°57'52.37"	2020.08.18–09.15	KNAE
**F25**	Pteromalidae	OQ134250	Baekbongnyeong, Imgye-myeon, Jeongseon-gun, Gangwon-do, S. Korea.	N37°32'32.74" E128°57'52.37"	2020.08.18–09.15	KNAE
**F26**	Pteromalidae	OQ134244	Baekbongnyeong, Imgye-myeon, Jeongseon-gun, Gangwon-do, S. Korea.	N37°32'32.74" E128°57'52.37"	2020.08.18–09.15	KNAE
**F27**	Pteromalidae	OQ134313	Mt. Baekdeok, Pyeongchang-eup, Pyeongchang-gun, Gangwon-do, S. Korea.	N37°24'6.33" E128°19'13.9"	2020.08.18–09.15	KNAE
**F28**	Pteromalidae	OQ134233	DMZ Arb., Mandae-ri, Haean-myeon, Yanggu-gun, Gangwon-do, S. Korea.	N38°15'9.3" E128°6'40.06"	2018.08.17–08.31	KNAE
**F29**	Pteromalidae	OQ134253	DMZ Arb., Mandae-ri, Haean-myeon, Yanggu-gun, Gangwon-do, S. Korea.	N38°15'9.3" E128°6'40.06"	2018.08.17–08.31	KNAE
**F30**	Pteromalidae	OQ134254	DMZ Arb., Mandae-ri, Haean-myeon, Yanggu-gun, Gangwon-do, S. Korea.	N38°15'9.3" E128°6'40.06"	2018.08.17–08.31	KNAE
**F31**	Pteromalidae	OQ134344	DMZ Arb., Mandae-ri, Haean-myeon, Yanggu-gun, Gangwon-do, S. Korea.	N38°15'9.3" E128°6'40.06"	2018.08.17–08.31	KNAE

**F32**	Pteromalidae	OQ134291	DMZ Arb., Mandae-ri, Haean-myeon, Yanggu-gun, Gangwon-do, S. Korea.	N38°15'9.3" E128°6'40.06"	2018.08.31–09.14	KNAE
**F33**	Pteromalidae	OQ134234	DMZ Arb., Mandae-ri, Haean-myeon, Yanggu-gun, Gangwon-do, S. Korea.	N38°15'9.3" E128°6'40.06"	2018.08.31–09.14	KNAE
**F34**	Pteromalidae	OQ134187	DMZ Arb., Mandae-ri, Haean-myeon, Yanggu-gun, Gangwon-do, S. Korea.	N38°15'9.3" E128°6'40.06"	2018.09.14–09.28	KNAE
**F35**	Pteromalidae	OQ134173	DMZ Arb., Mandae-ri, Haean-myeon, Yanggu-gun, Gangwon-do, S. Korea.	N38°15'9.3" E128°6'40.06"	2018.09.14–09.28	KNAE
**F36**	Pteromalidae	OQ134335	DMZ Arb., Mandae-ri, Haean-myeon, Yanggu-gun, Gangwon-do, S. Korea.	N38°15'9.3" E128°6'40.06"	2018.09.14–09.28	KNAE
**F37**	Pteromalidae	OQ134207	DMZ Arb., Mandae-ri, Haean-myeon, Yanggu-gun, Gangwon-do, S. Korea.	N38°15'9.3" E128°6'40.06"	2018.09.14–09.28	KNAE
**F38**	Pteromalidae	OQ134223	DMZ Arb., Mandae-ri, Haean-myeon, Yanggu-gun, Gangwon-do, S. Korea.	N38°15'9.3" E128°6'40.06"	2018.05.21–06.05	KNAE
**F39**	Pteromalidae	OQ134195	DMZ Arb., Mandae-ri, Haean-myeon, Yanggu-gun, Gangwon-do, S. Korea.	N38°15'9.3" E128°6'40.06"	2018.05.21–06.05	KNAE
**F40**	Pteromalidae	OQ134330	DMZ Arb., Mandae-ri, Haean-myeon, Yanggu-gun, Gangwon-do, S. Korea.	N38°15'9.3" E128°6'40.06"	2018.05.21–06.05	KNAE
**F42**	Pteromalidae	OQ134377	DMZ Arb., Mandae-ri, Haean-myeon, Yanggu-gun, Gangwon-do, S. Korea.	N38°15'9.3" E128°6'40.06"	2018.06.05–06.02	KNAE
**F43**	Pteromalidae	OQ134184	DMZ Arb., Mandae-ri, Haean-myeon, Yanggu-gun, Gangwon-do, S. Korea.	N38°15'9.3" E128°6'40.06"	2018.06.05–06.02	KNAE
**F44**	Pteromalidae	OQ134293	DMZ Arb., Mandae-ri, Haean-myeon, Yanggu-gun, Gangwon-do, S. Korea.	N38°15'9.3" E128°6'40.06"	2018.06.05–06.02	KNAE
**F45**	Pteromalidae	OQ134203	DMZ Arb., Mandae-ri, Haean-myeon, Yanggu-gun, Gangwon-do, S. Korea.	N38°15'9.3" E128°6'40.06"	2018.06.05–06.02	KNAE
**F46**	Pteromalidae	OQ134378	DMZ Arb., Mandae-ri, Haean-myeon, Yanggu-gun, Gangwon-do, S. Korea.	N38°15'9.3" E128°6'40.06"	2018.07.04–07.18	KNAE
**F47**	Pteromalidae	OQ134225	DMZ Arb., Mandae-ri, Haean-myeon, Yanggu-gun, Gangwon-do, S. Korea.	N38°15'9.3" E128°6'40.06"	2018.07.04–07.18	KNAE
**F48**	Pteromalidae	OQ134264	DMZ Arb., Mandae-ri, Haean-myeon, Yanggu-gun, Gangwon-do, S. Korea.	N38°15'9.3" E128°6'40.06"	2018.07.04–07.18	KNAE
**F50**	Pteromalidae	OQ134355	DMZ Arb., Mandae-ri, Haean-myeon, Yanggu-gun, Gangwon-do, S. Korea.	N38°15'9.3" E128°6'40.06"	2018.06.20–07.04	KNAE
**F51**	Pteromalidae	OQ134226	DMZ Arb., Mandae-ri, Haean-myeon, Yanggu-gun, Gangwon-do, S. Korea.	N38°15'9.3" E128°6'40.06"	2018.06.20–07.04	KNAE
**F52**	Pteromalidae	OQ134379	DMZ Arb., Mandae-ri, Haean-myeon, Yanggu-gun, Gangwon-do, S. Korea.	N38°15'9.3" E128°6'40.06"	2018.06.20–07.04	KNAE

**F53**	Pteromalidae	OQ134284	DMZ Arb., Mandae-ri, Haean-myeon, Yanggu-gun, Gangwon-do, S. Korea.	N38°15'9.3" E128°6'40.06"	2018.06.20–07.04	KNAE
**F54**	Pteromalidae	OQ134230	DMZ Arb., Mandae-ri, Haean-myeon, Yanggu-gun, Gangwon-do, S. Korea.	N38°15'9.3" E128°6'40.06"	2018.06.20–07.04	KNAE
**F55**	Pteromalidae	OQ134285	DMZ Arb., Mandae-ri, Haean-myeon, Yanggu-gun, Gangwon-do, S. Korea.	N38°15'9.3" E128°6'40.06"	2018.06.20–07.04	KNAE
**F56**	Pteromalidae	OQ134260	DMZ Arb., Mandae-ri, Haean-myeon, Yanggu-gun, Gangwon-do, S. Korea.	N38°15'9.3" E128°6'40.06"	2018.08.02–08.17	KNAE
**F57**	Pteromalidae	OQ134193	DMZ Arb., Mandae-ri, Haean-myeon, Yanggu-gun, Gangwon-do, S. Korea.	N38°15'9.3" E128°6'40.06"	2018.08.02–08.17	KNAE
**F58**	Pteromalidae	OQ134356	DMZ Arb., Mandae-ri, Haean-myeon, Yanggu-gun, Gangwon-do, S. Korea.	N38°15'9.3" E128°6'40.06"	2018.08.02–08.17	KNAE
**F59**	Pteromalidae	OQ134185	DMZ Arb., Mandae-ri, Haean-myeon, Yanggu-gun, Gangwon-do, S. Korea.	N38°15'9.3" E128°6'40.06"	2018.08.17–08.31	KNAE
**F60**	Pteromalidae	OQ134357	DMZ Arb., Mandae-ri, Haean-myeon, Yanggu-gun, Gangwon-do, S. Korea.	N38°15'9.3" E128°6'40.06"	2018.08.17–08.31	KNAE
**F61**	Pteromalidae	OQ134192	DMZ Arb., Mandae-ri, Haean-myeon, Yanggu-gun, Gangwon-do, S. Korea.	N38°15'9.3" E128°6'40.06"	2018.08.17–08.31	KNAE
**F62**	Pteromalidae	OQ134242	DMZ Arb., Mandae-ri, Haean-myeon, Yanggu-gun, Gangwon-do, S. Korea.	N38°15'9.3" E128°6'40.06"	2018.08.17–08.31	KNAE
**F63**	Pteromalidae	OQ134328	DMZ Arb., Mandae-ri, Haean-myeon, Yanggu-gun, Gangwon-do, S. Korea.	N38°15'9.3" E128°6'40.6"	2019.06.12–07.04	KNAE
**F65**	Pteromalidae	OQ134333	B-4, Millak-dong, Uijeongbu-si, Gyeonggi-do, Gyeonggi-do, S. Korea	N37°45'19.1" E127°09'14.8"	2017.10.16–10.03	KNAE
**F66**	Pteromalidae	OQ134349	Mt. Gaein, Inje-gun, Gangwon-do, S. Korea.	-	–	KNAE
**F67**	Pteromalidae	OQ134255	Mt. Gaein, Inje-gun, Gangwon-do, S. Korea.	-	–	KNAE
**F68**	Pteromalidae	OQ134221	Mt. Gaein, Inje-gun, Gangwon-do, S. Korea.	-	–	KNAE
**F69**	Pteromalidae	OQ134373	Mt. Gaein, Inje-gun, Gangwon-do, S. Korea.	-	–	KNAE
**F70**	Pteromalidae	OQ134238	Mt. Goseong, Eocheon-ri, Ganseong-eup, Goseong-gun, Gyeonggi-do, S. Korea.	N38°21'2540" E128°26'62.9"	2019.05.27	KNAE
**F71**	Pteromalidae	OQ134321	Dutayeon, Bangsan-myeon, Yanggu-gun, Gangwon-do, S. Korea	N38°13'7330" E127°508'78"	2019.08.09	KNAE
**F72**	Pteromalidae	OQ134342	Dutayeon, Bangsan-myeon, Yanggu-gun, Gangwon-do, S. Korea	N38°13'7330" E127°508'78"	2019.08.09	KNAE
**F73**	Pteromalidae	OQ134231	Hyangnobong, Sudong-myeon, Goseong-gun, Gangwon-do, S. Korea.	N38°16'1910" E128°19'23.7"	2019.08.09	KNAE

**F74**	Pteromalidae	OQ134216	Hyangnobong, Sudong-myeon, Goseong-gun, Gangwon-do, S. Korea.	N38°16'1910" E128°19'23.7"	2019.08.09	KNAE
**F75**	Pteromalidae	OQ134245	Hyangnobong, Sudong-myeon, Goseong-gun, Gangwon-do, S. Korea.	N38°16'1910" E128°19'23.7"	2019.08.09	KNAE
**F76**	Pteromalidae	OQ134210	Hyangnobong, Sudong-myeon, Goseong-gun, Gangwon-do, S. Korea.	N38°16'1910" E128°19'23.7"	2019.08.09	KNAE
**F77**	Pteromalidae	OQ134304	Hyangnobong, Sudong-myeon, Goseong-gun, Gangwon-do, S. Korea.	N38°16'1910" E128°19'23.7"	2019.09.06	KNAE
**F78**	Pteromalidae	OQ134186	Mt. Toemo, Naega-myeon, Ganghwa-gun, Incheon, S. Korea.	N37°42'9380" E126°24'88.7"	2019.10.02	KNAE
**F79**	Pteromalidae	OQ134310	Mt. Toemo, Naega-myeon, Ganghwa-gun, Incheon, S. Korea.	N37°42'9380" E126°24'88.7"	2019.10.02	KNAE
**F89**	Pteromalidae	OQ134196	DMZ Arb., Mandae-ri, Haean-myeon, Yanggu-gun, Gangwon-do, S. Korea.	N38°15'9.3" E128°6'40.06"	2017.08.21	KNAE
**F90**	Pteromalidae	OQ134292	DMZ Arb., Mandae-ri, Haean-myeon, Yanggu-gun, Gangwon-do, S. Korea.	N38°15'9.3" E128°6'40.06"	2017.08.21	KNAE
**F91**	Pteromalidae	OQ134256	DMZ Arb., Mandae-ri, Haean-myeon, Yanggu-gun, Gangwon-do, S. Korea.	N38°15'9.3" E128°6'40.06"	2017.08.21	KNAE
**F92**	Pteromalidae	OQ134336	DMZ Arb., Mandae-ri, Haean-myeon, Yanggu-gun, Gangwon-do, S. Korea.	N38°15'9.3" E128°6'40.06"	2017.08.21	KNAE
**F93**	Pteromalidae	OQ134311	DMZ Arb., Mandae-ri, Haean-myeon, Yanggu-gun, Gangwon-do, S. Korea.	N38°15'9.3" E128°6'40.06"	2017.09.19	KNAE
**F95**	Pteromalidae	OQ134283	DMZ Arb., Mandae-ri, Haean-myeon, Yanggu-gun, Gangwon-do, S. Korea.	N38°15'9.3" E128°6'40.06"	2017.09.19	KNAE
**F96**	Pteromalidae	OQ134224	DMZ Arb., Mandae-ri, Haean-myeon, Yanggu-gun, Gangwon-do, S. Korea.	N38°15'9.3" E128°6'40.06"	2017.05.27	KNAE
